# Biodegradation of Benzo[a]pyrene by a White-Rot Fungus *Phlebia acerina*: Surfactant-Enhanced Degradation and Possible Genes Involved

**DOI:** 10.3390/jof9100978

**Published:** 2023-09-28

**Authors:** Wenquan Zhang, Qiaoyu Li, Jianqiao Wang, Ziyu Wang, Hongjie Zhan, Xiaolong Yu, Yan Zheng, Tangfu Xiao, Li-Wei Zhou

**Affiliations:** 1 Key Laboratory for Water Quality and Conservation of the Pearl River Delta, Ministry of Education, School of Environmental Science and Engineering, Guangzhou University, Guangzhou 510006, China; 2 School of Environmental Science and Engineering, Southern University of Science and Technology, Shenzhen 518055, China; 3 State Key Laboratory of Geohazard Prevention and Geoenvironment Protection, Chengdu University of Technology, Chengdu 610059, China; 4 State Key Laboratory of Mycology, Institute of Microbiology, Chinese Academy of Sciences, Beijing 100101, China

**Keywords:** benzo[a]pyrene, white-rot fungus, degradation, surfactants

## Abstract

Polycyclic aromatic hydrocarbons (PAHs) are persistent environmental pollutants that pose a threat to human health. Among these PAHs, benzo[a]pyrene (BaP), a five-ring compound, exhibits high resistance to biodegradation. White-rot fungus *Phlebia acerina* S-LWZ20190614-6 has demonstrated higher BaP degradation capabilities compared with *Phanerochaete chrysosporium* and *P. sordida* YK-624, achieving a degradation rate of 57.7% after 32 days of incubation under a ligninolytic condition. To further enhance the biodegradation rate, three nonionic surfactants were used, and the addition of 1 or 2 g·L^−1^ of polyethylene glycol monododecyl ether (Brij 30) resulted in nearly complete BaP biodegradation by *P. acerina* S-LWZ20190614-6. Interestingly, Brij 30 did not significantly affect the activity of manganese peroxidase and lignin peroxidase, but it did decrease laccase activity. Furthermore, the impact of cytochrome P450 on BaP degradation by *P. acerina* S-LWZ20190614-6 was found to be relatively mild. Transcriptomic analysis provided insights into the degradation mechanism of BaP, revealing the involvement of genes related to energy production and the synthesis of active enzymes crucial for BaP degradation. The addition of Brij 30 significantly upregulated various transferase and binding protein genes in *P. acerina* S-LWZ20190614-6. Hence, the bioremediation potential of BaP by the white-rot fungus *P. acerina* S-LWZ20190614-6 holds promise and warrants further exploration.

## 1. Introduction

Polycyclic aromatic hydrocarbons (PAHs) are organic compounds comprised of two or more fused benzene rings arranged in various configurations [1,2]. These compounds are commonly found in the environment and are produced through incomplete combustion of organic substances, such as vehicle exhaust, trash incineration, coal and petroleum combustion, cigarette smoke, and wood treatment [3,4,5]. In China, the most significant sources of PAH emissions are organic combustion, coal combustion, and the coking industry [6,7,8]. Benzo[a]pyrene (BaP), a high molecular weight PAH with five rings, is one of the most typical PAHs. The formation mechanism of BaP was first discovered in 1960 and it was reported that hydrocarbons with relatively small molecular weight form BaP through a series of reactions at high temperatures [9]. The United States Environmental Protection Agency has listed BaP as a priority pollutant [10]. BaP is a major factor in food pollution, often produced during food processing, and poses a significant threat to food safety [11]. Long-term exposure to indoor burning bituminous coal will significantly increase the risk of lung cancer for residents, and there is a significant positive correlation between the concentration of BaP and the mortality of lung cancer [12]. Additionally, BaP can be adsorbed onto atmospheric particles, which can then pollute surface water through sedimentation and precipitation washing [13,14]. BaP accumulates in water bodies and increases in concentration as aquatic organisms grow and develop. The pollution can then spread through biological migration and amplification, ultimately endangering human health.

Currently, there are several methods to remove PAHs, which can be roughly divided into chemical, physical, and biological methods. Ultrasonic elimination, adsorption, extraction, oxidation, and ultraviolet light decomposition are relatively mature methods, but they all have problems such as high cost, complex processes, high technical difficulty, and serious secondary pollution, which can harm human health and the public environment [15,16,17,18]. As a result, the microbial degradation method has been proposed as a viable approach for eliminating PAHs. This method is characterized by its environmental friendliness, low cost, and high efficiency, rendering it an ideal and effective means for eliminating PAHs from the environment [19]. Various microorganisms, including bacteria, algae, and fungi, have been shown to play an essential role in the biodegradation of PAHs [20,21,22]. Many studies have investigated the biodegradation and metabolism of PAHs containing two to four rings by bacteria and fungi [23,24,25,26]. However, studies on BaP have been limited due to its low solubility, high toxicity, and the inability of most bacteria to utilize it as the sole carbon source. Fungal degradation of high molecular weight PAHs has been widely examined, and *Phanerochaete chrysosporium* was the first white-rot fungus reported to possess the ability to biodegrade BaP [27].

The degradation of PAHs by white-rot fungi mainly occurs through the transformation of lignin-degrading enzymes [28]. The enzyme system of white-rot fungi comprises laccase, manganese peroxidase (MnP), and lignin peroxidase (LiP). Laccase, which belongs to the multi-copper oxidase family, possesses a unique redox potential and can both oxidize and reduce [29]. Studies have shown that laccase isolated from *Trametes versicolor* could degrade various PAHs, and the degradation rate of PAHs was close to 100% [30]. Additionally, Cho et al. [31] also showed that laccase secreted by white-rot fungus *Coriolus hirsutus* can catalyze the oxidation of five PAHs under the condition of redox mediator. The role of cytochrome P450 monooxygenases in fungal metabolism of high molecular weight PAHs also have been demonstrated. P450-mediated BaP hydroxylation was found in microsomal fractions from *P. chrysosporium* [32]. Six recombinant P450 monooxygenases from *P. chrysosporium* showed PAH (3~5 rings)-oxidizing activity [33]. Moreover, surfactants have the potential to enhance the solubility and biodegradability of PAHs. Our previous research demonstrated that the white-rot fungus *P. sordida* was capable of degrading PAHs with three, four, and five rings, and the addition of surfactants has been shown to double the biodegradation rate of BaP [34]. However, it could not completely degrade high concentrations (50 mg·L^−1^) of BaP, and underlying mechanisms of BaP degradation by white-rot fungi remain unclear.

Surpassing the above issues, highly efficient white-rot fungi capable of degrading PAHs were screened. Previous studies have reported on the significant potential for PAH biodegradation by the genera *Phanerochaete* and *Phlebia* [34,35]. While *Phlebia acerina* has been shown to degrade three- and four-ring PAHs [35]; its ability to degrade five-ring PAHs remains unclear. In the present study, *P. acerina* S-LWZ20190614-6 has a higher capacity for degrading BaP compared with *P. chrysosporium* and *P. sordida*. Piperonyl butoxide (PB), a common inhibitor of cytochrome P450s, was used to demonstrate that cytochrome P450s were involved in the degradation of BaP by *P. acerina* S-LWZ20190614-6. LiP, MnP, and laccase enzyme activity were assayed to determine their role in BaP biodegradation. To enhance the rate of BaP biodegradation, three nonionic surfactants, polyethylene glycol monododecyl ether (Brij 30), polyethylene glycol mono-4-octylphenyl ether (Triton X-100), and polyoxyethylene sorbitan monooleate (Tween 80), were tested. The addition of 1 or 2 g·L^−1^ of Brij 30 nearly completely degraded BaP by *P. acerina* S-LWZ20190614-6. Additionally, transcriptome analysis utilizing RNA-Seq was used to identify potential genes involved in BaP degradation in *P. acerina* S-LWZ20190614-6. To the best of our knowledge, this study represents the pioneering investigation into the ability of *P. acerina* to achieve near-complete degradation of BaP with the addition of nonionic surfactants. Furthermore, we examined the potential genes involved in this degradation process.

## 2. Materials and Methods

### 2.1. Fungus and Chemicals

In this study, three white-rot fungi *P. chrysosporium* ME-446 (ATCC 34541), *P. sordida* YK-624 (ATCC 90872), and *P. acerina* S-LWZ20190614-6 (CGMCC 40196) were used. These strains were initially cultured on potato dextrose agar (PDA) and maintained on the same slants at 4 °C. Kirk medium, which contained 10 g·L^−1^ glucose and trace elements, was used in the degradation experiment [36]. BaP with 96.0% analytical standards was purchased from Macklin, China. A solution of BaP in acetone (5000 mg·L^−1^) was prepared before to use. PB and three nonionic surfactants were purchased from Tokyo Chemical Industry Co. (Tokyo, Japan). High-performance liquid chromatography (HPLC)-grade methanol was used for HPLC analyses, and all other reagents were of analytical reagent grade.

### 2.2. BaP Degradation in Liquid Media by White-Rot Fungi

White-rot fungi were incubated on PDA plates and incubated at 30 °C. The Kirk medium was prepared and dispensed into a 100 mL conical flask at 10 mL and then sterilized at 121 °C for 15 min. Two fungal disks, each with a diameter of 10 mm, were inoculated into the liquid media and subsequently incubated at 30 °C for 3 days. After that, 100 μL of BaP solution was added to each flask, resulting in a final concentration of 50 mg·L^−1^. All experiments were carried out in triplicate. Samples were collected with the addition of twice the volume of acetonitrile in the culture every 4 days during the 32-day incubation period. The sample handling method after cultivation and the HPLC testing method were consistent with our previously published paper [34].

### 2.3. Cytochrome P450s Inhibition and Surfactant-Enhanced Degradation Experiments

To investigate the involvement of cytochrome P450 in the degradation of BaP, different concentrations of cytochrome P450 inhibitor PB were used. Pre-incubated cultures were supplemented with BaP and PB at concentrations of 50 and 500 mg·L^−1^, respectively. The subsequent experimental procedures were carried out as described in Section 2.2. Each experiment was performed in triplicate.

To enhance the biodegradation of BaP, three nonionic surfactants, polyethylene glycol monododecyl ether (Brij 30), polyethylene glycol mono-4-octylphenyl ether (Triton X-100), and polyoxyethylene sorbitan monooleate (Tween 80) were used. The pre-incubation of *P. acerina* S-LWZ20190614-6 was performed as above. BaP at a concentration of 50 mg·L^−1^ and different concentrations (1 or 2 g·L^−l^) of Brij 30, Triton X-100, or Tween 80 were added to the cultures, which were then incubated at 30 °C for 8, 16, and 32 days. The extraction and quantification of BaP were performed as described in Section 2.2. All experiments were conducted in triplicate, and the statistical analyses were performed using Microsoft Office Excel 2019 and GraphPad 9 software.

### 2.4. Biomass and Enzyme Activity Estimation

The cultures were obtained as described above. The mycelia were separated from the culture fluid via filtration using a 0.2 μm filter and subsequently dried at 105 °C after removing the PDA disc and then weighed. The resulting filtrate was used for enzymatic assay of LiP and MnP activity according to the method described previously [37]. Laccase activity was measured based on the method reported by [38]; the tartaric acid buffer was replaced with citric acid/disodium hydrogen phosphate in this study. The activity of LiP was assayed based on the oxidization of VA to veratraldehyde (ε310 = 9300 M^−1^ cm^−1^). While the activity of MnP was the oxidation of 2,6-dimethoxyphenol to coerulignone (ε470 = 49.6 mM^−1^ cm^−1^). Laccase activity was determined by monitoring the oxidation of ABTS to its cation radical ABTS^·+^ (*ε*420 = 36,000 M^−1^ cm^−1^).

### 2.5. RNA-Sequencing Analysis and Quantitative Real-Time PCR (qPCR)

RNA-Seq was applied to elucidate the functional genes of *P. acerina* S-LWZ20190614-6 in the degradation of BaP. Cultures containing BaP, as well as those with BaP and 2 g·L^−1^ of Brij 30, which were incubated for 16 days, were collected. Media lacking BaP were used as the control. The fungal mycelia were separated from the solution via suction filtration and stored at −80 °C. All experiments were performed in triplicate. RNA extraction, sequencing, and transcriptomic analysis were carried out following the methodology outlined in our previously published paper [39]. Clean bases of all samples were greater than 5.8 GB, and other more detailed information is shown in Table S1. The samples for differential expression analysis were described as **NoBaP** (control, without BaP), **BaP** (with BaP), and **Brij30_BaP** (with BaP and 2 g·L^−1^ of Brij 30). The details of differential expression genes (DEGs) are shown in Tables S2–S4.

qPCR was carried out using an Applied Biosystems ViiA 7 Real-Time PCR System (Thermo Fisher Scientific, Waltham, MA, USA). The reaction solution preparation and PCR amplification reaction were described in our previous studies [37]. Each reaction contained 3 μL of cDNAs, 2 μL of PCR primers, 10 μL of water, and 5 μL of SYBR Green Master Mix (Yeasen, Guangzhou, China). The amplification reaction program was set as follows: predenaturation, 95 °C for 5 min; amplification, 40 cycles at 95 °C for 10 s and 60 °C for 30 s. After amplification, the temperature was raised to 90 °C at a rate of 0.05 °C/s, and the melting curve was generated by the software automatically. The relative quantification of DEG expression was determined using the 2^−ΔΔCT^ method. Actin was used as a reference gene for normalization. The details of these genes are shown in Table S5.

## 3. Results

### 3.1. BaP Degradation by White-Rot Fungi and Surfactant-Enhanced BaP Degradation

**Figure 1:**
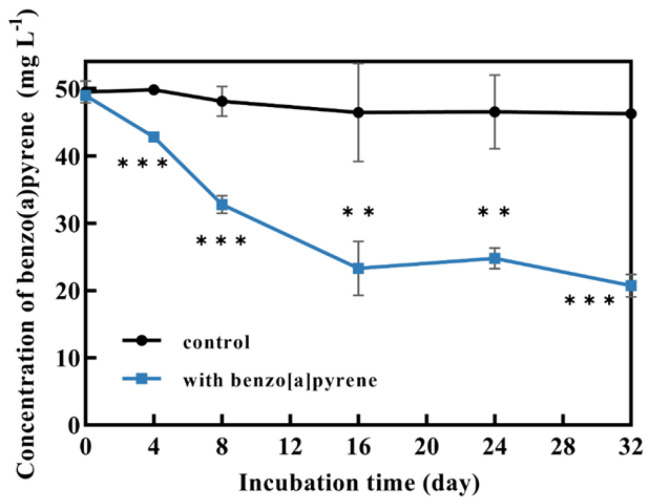
Degradation of BaP by *P. acerina* S-LWZ20190614-6 for 32 days of incubation. The control was Kirk media without fungus. **, *p* < 0.01, ***, *p* < 0.001, compared with the control.

**Figure 2:**
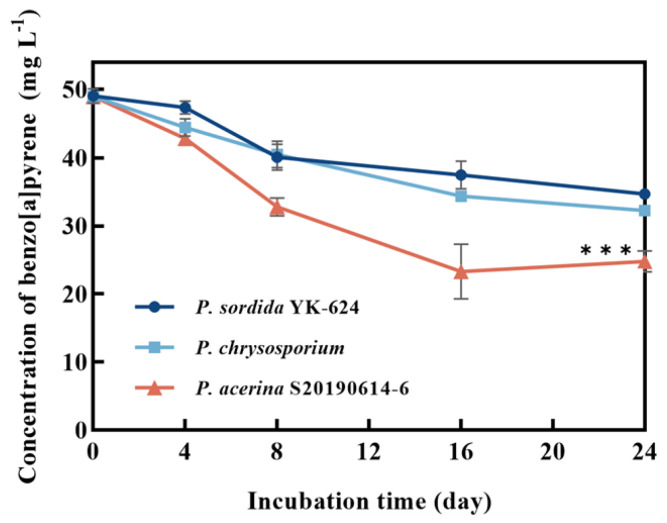
Degradation of BaP by white-rot fungi. Dark blue circle: *P. sordida* YK-624; light blue square: *P. chrysosporium*; red triangle: *P. acerina* S-LWZ20190614-6. ***, *p* < 0.001, compared with the *P. chrysosporium* samples.

*P. acerina* S-LWZ20190614-6 showed a highly efficient capability for BaP degradation, with 57.7% of BaP being degraded after 32 days of incubation, and a volatility rate of 6.5% was observed in the control (Figure 1). Moreover, white-rot fungi *P. chrysosporium* and *P. sordida* YK-624 were used to compare the degradation capacity for BaP. After 24 days of incubation in this study. *P. sordida* YK-624 exhibited the lowest BaP degradation rate of 29.3%, whereas *P. chrysosporium* and *P. acerina* S-LWZ20190614-6 degraded 34.2% and 52% of BaP, respectively (Figure 2). These results suggested that *P. acerina* S-LWZ20190614-6 has a highly effective degradation capacity for BaP among these white-rot fungi, warranting further study.

**Figure 3:**
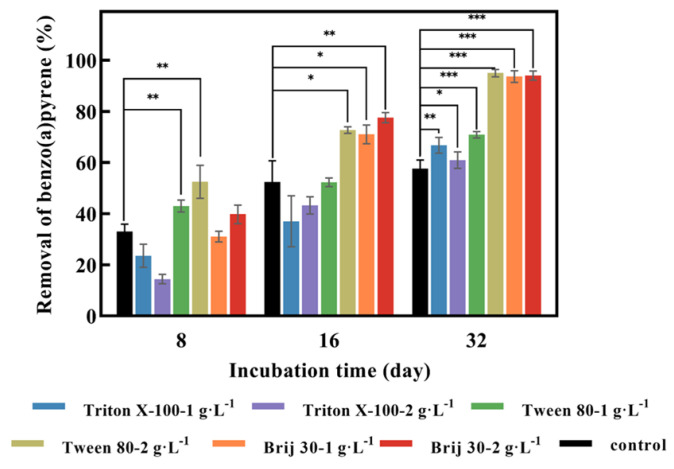
Effect of three surfactants on the degradation of BaP by *P. acerina* S-LWZ20190614-6. The control was without surfactants. Black: control; blue: Triton X-100-1 g·L^−1^; purple: Triton X-100-2 g·L^−1^; green: Tween 80-1 g·L^−1^; khaki: Tween 80-2 g·L^−1^; orange: Brij 30-1 g·L^−1^; red: Brij 30-2 g·L^−1^. *, *p* < 0.05, **, *p* < 0.01, ***, *p* < 0.001, compared with the control.

As can be seen from Figure 3, with the addition of Triton X-100 at concentrations of 1 g·L^−1^ and 2 g·L^−1^, the removal values of BaP by *P. acerina* S-LWZ20190614-6 after 32 days of incubation were 66.8% and 61.0%, respectively. Whereas, the degradation rate at 8 days and 16 days of incubation was lower than the control (Figure 3). These results suggest that Triton X-100 had an inhibitory effect on BaP degradation, possibly due to Triton X-100 inhibiting fungal growth. Triton X-100 had a toxic effect on *Pseudomonas aeruginosa* when it degraded pyrene, due to the toxic metabolites formed from the utilization of Triton X-100 [40]. Moreover, both Tween 80 and Brij 30 enhanced the removal of BaP. At Tween 80 concentrations of 1 g·L^−1^ and 2 g·L^−1^, the degradation of BaP by *P. acerina* S-LWZ20190614-6 after 32 days of incubation increased by 13.2% and 37.3% compared with the control. Brij 30 showed a significant result for BaP degradation, which enhanced the removal efficiencies from 57.7% in the control condition to 93.7% and 94.0% for 1 g·L^−1^ and 2 g·L^−1^ of Brij 30 after 32 days of incubation (Figure 3).

### 3.2. Effect of Surfactant Brij 30 on Biomass and Enzyme Production

**Figure 4:**
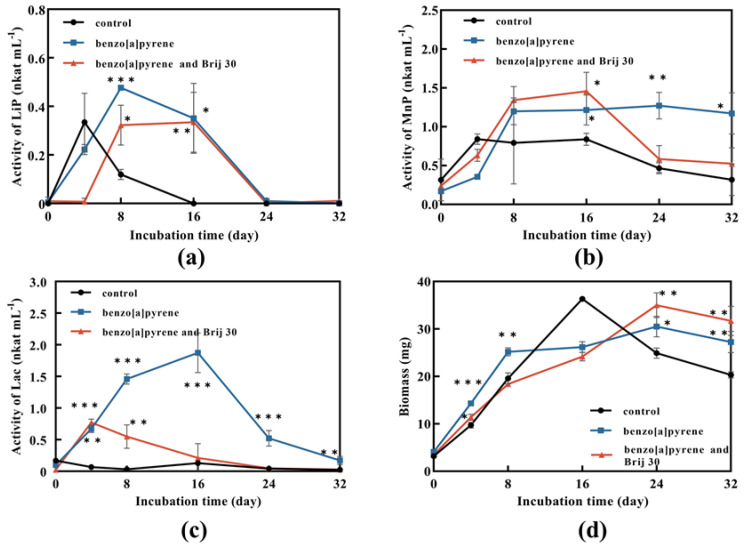
Enzyme activity and biomass of *P. acerina* S-LWZ20190614-6 in degrading BaP process. (**a**) LiP activity; (**b**) MnP activity; (**c**) Lac activity; (**d**) biomass. The control was *P. acerina* S-LWZ20190614-6 without BaP. Black circle: control; blue square: with BaP; red triangle: with BaP and Brij 30. *, *p* < 0.05, **, *p* < 0.01, ***, *p* < 0.001, compared with the control.

Fungal biomass without BaP and Brij 30 reached the maximum of 36.3 mg after 16 days of incubation and then decreased (Figure 4d). It can be assumed that the carbon source was not enough for the growth of *P. acerina* S-LWZ20190614-6. On the other hand, fungal biomass was continuously increased with the addition of BaP and Brij 30 (Figure 4d), indicating that the presence of BaP and Brij 30 supported cell growth.

Ligninolytic peroxidases and laccases can degrade PAHs [41,42,43]. The results showed that the highest enzyme activity was obtained in laccase, followed by MnP and LiP. LiP activity of the samples with BaP was lower than that of the control at the first 4 days, and then exceeded the control rapidly and reached the highest activity on day 8 (Figure 4a). Therefore, it can be assumed that *P. acerina* S-LWZ20190614-6 needed adaptation time for the presence of BaP to have an effect. LiP activity played a role in the degradation process and Brij 30 had no enhanced effect on LiP activity (Figure 4a). A similar trend was also seen in MnP activity (Figure 4b). MnP was also involved in the degradation of BaP and Brij 30 did not significantly affect MnP activity (Figure 4b). Laccase showed the highest enzyme activity in this study. However, laccase activity was decreased by the addition of Brij 30 (Figure 4c).

### 3.3. BaP Degradation Effects of Cytochrome P450s on BaP Degradation by P. acerina S-LWZ20190614-6

**Figure 5:**
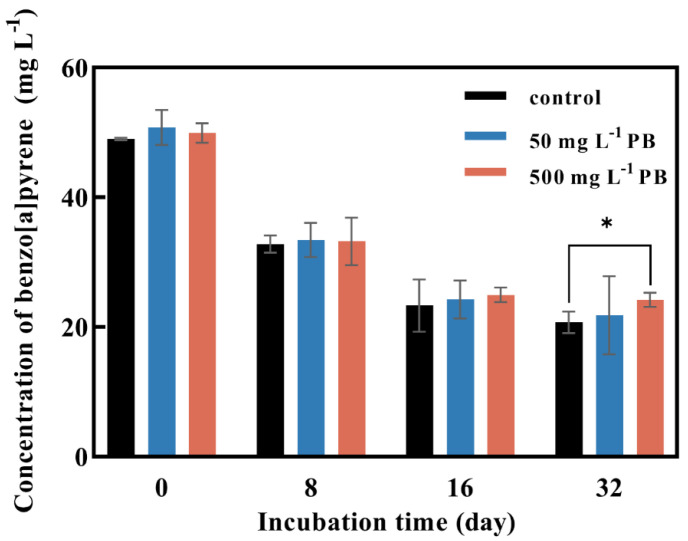
Effect of cytochrome P450 inhibitor PB concentration on BaP degradation by *P. acerina* S-LWZ20190614-6. The control was without PB. Black: control; blue: with 50 mg L^−1^ of PB; red: with 500 mg L^−1^ of PB. *, *p* < 0.05, compared with the control.

Three families of oxidoreductases, including ligninolytic enzymes, cytochrome P450s, and dioxygenases, are critical for fungal bioremediation [44,45]. Fungi can oxidize PAHs to quinones by secreting ligninolytic enzymes into the extracellular compartment, and then PAHs are degraded by hydrogenation and dehydration [46]. PB is a common cytochrome P450s inhibitor that has been used in many studies to determine if cytochrome P450s is involved in the degradation of organic pollutants [37,47,48,49]. After 8 days of incubation, the degradation rate of BaP was about 33% and PB had no effect on the degradation of BaP (Figure 5). The degradation of BaP reached 50% after 16 days of incubation and it decreased by 2% with the addition of 500 mg·L^−1^ of PB. The degradation of BaP was decreased by 6% with the addition of 500 mg·L^−1^ of PB after 32 days of incubation (Figure 5).

### 3.4. Transcriptome Analysis for BaP Degradation Mechanism by P. acerina S-LWZ20190614-6

In this study, RNA-Seq was used to identify the DEGs in *P. acerina* S-LWZ20190614-6 that responded to BaP degradation, as well as the enhanced degradation facilitated using the surfactant Brij 30. DEGs were obtained by comparing samples with BaP degradation (**BaP**), samples with Brij 30-enhanced BaP degradation (**Brij30_BaP**), and samples without BaP (**NoBaP**). The comparisons revealed 1170 upregulated and 1197 downregulated DEGs in the **BaP** and **NoBaP** comparisons, 282 upregulated and 557 downregulated DEGs in the **Brij30_BaP** and **BaP** comparisons, and 393 upregulated and 833 downregulated DEGs in the **Brij30_BaP** and **NoBaP** comparisons (Figure S1). Moreover, a total of 9472 DEGs were found in all comparisons, with 473, 236, and 88 DEGs identified uniquely in **NoBaP**, **BaP,** and **Brij30_BaP**, respectively (Figure S2). GO analysis revealed that the upregulated DEGs in the **BaP** and **NoBaP** comparisons were significantly enriched in the biological process categories, such as the nucleobase-containing compound metabolic process, the nucleic acid metabolic process, the ribonucleic acid metabolic process, the ncRNA metabolic process, and the cellular macromolecular biosynthetic process (Figure S3a). DEGs were also associated with various cellular component categories, including organelles, nuclei, and protein-containing complexes. In terms of molecular functions, the upregulated DEGs were found to be enriched in RNA binding, catalytic activity, translation factor activity, DNA binding, microtubule binding, and ligase activity (Figure S3a). Furthermore, the comparison between **Brij30_BaP** and **BaP** revealed that the upregulated DEGs were enriched in cellular components such as the cell wall and membrane (Figure S3b). Additionally, the upregulated DEGs in the **Brij30_BaP** and **NoBaP** comparisons were found to be associated with biological processes such as protein deubiquitination, modification, and dephosphorylation. In terms of cellular components, these DEGs were enriched in organelle subcompartments and the endomembrane system, specifically the endoplasmic reticulum. Moreover, the molecular functions of these DEGs were related to protease activity, hydrolase activity, and phosphatase activity (Figure S3c). Furthermore, the addition of Brij 30 primarily affected the components and intrinsic constituents of the cell wall and membrane. Fungal cell membranes are known to contain a variety of lipids that play a role in the synthesis and transport of enzyme transporter proteins [50].

To further investigate the DEGs associated with the degradation of BaP by *P. acerina* S-LWZ20190614-6, KEGG functional annotation and pathway enrichment analysis were performed. In the **BaP** and **NoBaP** comparisons, enriched KEGG pathways included eukaryotic ribosome biogenesis, DNA replication, biosynthesis of amino acid, cysteine and methionine metabolism, alanine, aspartate and glutamate metabolism, nucleocytoplasmic transport, and nucleotide excision repair (Figure S4a). The degradation of BaP by *P. acerina* S-LWZ20190614-6 with 2 g·L^−1^ of Brij 30 and without Brij 30 showed that the enriched KEGG pathways included the biosynthesis of secondary metabolites, vitamin B6 metabolism, carbon metabolism, amino acid biosynthesis, and cofactor biosynthesis (Figure S4b). Additionally, enriched KEGG pathways in the **Brij30_BaP** and **NoBaP** comparisons included nucleotide excision repair, 2-oxocarboxylic acid metabolism, terpenoid backbone biosynthesis, nucleocytoplasmic transport, biosynthesis of unsulfurated fatty acids, fatty acid metabolism, and biosynthesis of amino acids (Figure S4c).

### 3.5. qPCR Analysis

**Figure 6:**
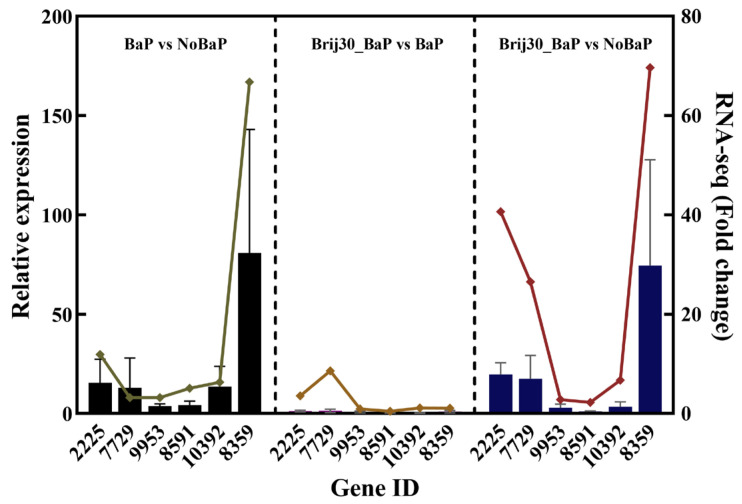
Correlation analysis between qPCR and RNA-seq results for upregulated DEGs (**left**: BaP vs. NoBaP; **middle**: Brij30_BaP vs. BaP; **right**: Brij30_BaP vs. NoBaP). Bars: qPCR results; line: RNA-seq results. The details of these genes are shown in Table S5.

To verify the reliability of the expression profile obtained from RNA-Seq, gene expression was analyzed using the qPCR method. The results showed that the expression levels of six candidate genes from DEGs were consistent with those obtained by RNA-Seq (Figure 6).

## 4. Discussion

Since the 1970s, there have been reports on the ability of bacterial strains to degrade BaP [51]. The first white-rot fungus reported to possess the ability to biodegrade BaP was *P. chrysosporium* [27]. *P. sordida* YK-624 has been shown to have a lignin degradation rate twice that of *P. chrysosporium* [52]. Our previous studies have demonstrated that *P. sordida* YK-624 can degrade a wide range of organic pollutants, including aflatoxin B1, bisphenol compounds, and neonicotinoid insecticides [53,54,55,56]. Our results suggested that *P. acerina* S-LWZ20190614-6 has a higher degradation capacity for BaP than the other two white-rot fungi, with 57.7% of BaP being degraded after 32 days of incubation (Figure 1 and Figure 2). Among various surfactants, nonionic surfactants have high stability, strong solubilization ability, and are not susceptible to strong electrolytes, acids, and bases [57]. Nonionic surfactants are always used for the enhancement of PAH bioremediation [58]. Recent results indicate that the degradation of BaP by *P. sordida* YK-624 is increased approximately two-fold by the addition of nonionic surfactant Brij 30 [34]. In this study, three nonionic surfactants increased the degradation rate of BaP; *P. acerina* S-LWZ20190614-6 almost completely degraded BaP with the addition of Brij 30 whether it was 1 g·L^−1^ or 2 g·L^−1^ (Figure 3). In previous studies, it has been found that Brij 30 can improve biomass and ligninolytic enzyme production [59,60,61]. Furthermore, biomass production and the LiP, MnP, and laccase activity of *P. acerina* S-LWZ20190614-6 during the degradation period after the addition of 2 g·L^−1^ of Brij 30 were detected (Figure 4). From these results, it was observed that ligninolytic enzymes are involved in the degradation of BaP, but are not the key factors responsible for the degradation.

White-rot fungi had more than 150 kinds of cytochrome P450s, and they showed degradation capacity for a wide range of organic pollutants [56,62,63,64]. The cytochrome P450 enzymes catalyzed the PAH benzene ring to form aromatic epoxides by the addition of oxygen, and unstable catalytic aromatic epoxides were further reacted to generate phenol derivatives, which are highly water-soluble, have low toxicity, and are more easily further degraded [46]. The effect of cytochrome P450s in the degradation of BaP by *P. acerina* S-LWZ20190614-6 was inhibited by PB, in which 50 mg·L^−1^ or 500 mg·L^−1^ of PB was added to the culture (Figure 5).

**Figure 7:**
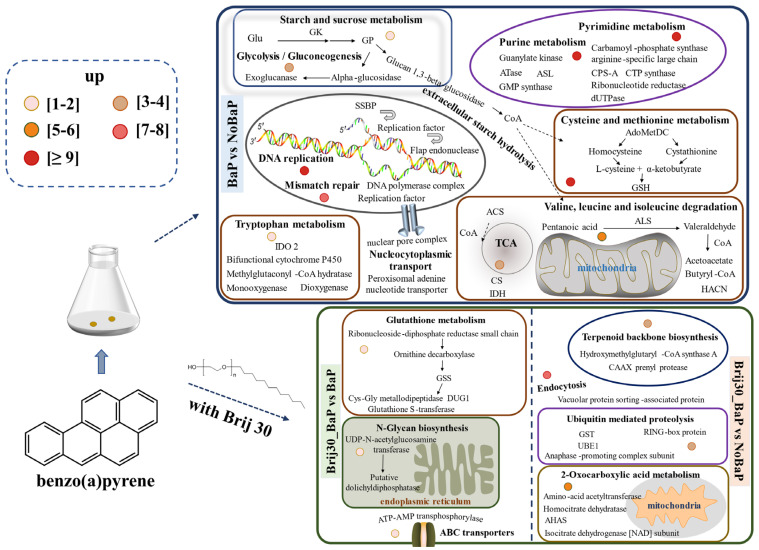
Proposed functional genes of *P. acerina* S-LWZ20190614-6 for BaP degradation with or without surfactant Brij 30. The numbers of upregulated differentially expressed genes are depicted as circles of varying colors. Glu, glucose; GK, glucokinase; GP, glycogen phosphorylase; HK, homoserine kinase; CSE, cystathionine gamma-lyase; CBS, cystathionine beta-synthase; HisHF, omidazole glycerol phosphate synthase hisHF; BACT, branched-chain-amino-acid aminotransferase; GoGAT[NADPH], putative glutamate synthase (NADPH); SSBP, single-stranded DNA-binding protein; AdoMetDC, S-adenosylmethionine decarboxylase proenzyme; GSH, glutathione; IDO 2, indoleamine 2,3-dioxygenase 2; ACS, A synthetase; CS, citrate synthase; IDH, isocitric dehydrogenase; ALS, acetolactate synthase; HACN, homoaconitase; GSS, glutathione synthetase; CPO, oxygen-dependent coproporphyrinogen-III oxidase; GST, glutathione S-transferase; UBE1, ubiquitin-activating enzyme; AHAS, acetohydroxyacid synthase.

Based on the transcriptomic analysis, this study discussed the genes involved in the biodegradation of BaP by *P. acerina* S-LWZ20190614-6 (Figure 7). The KEGG enrichment analysis of the upregulated DEGs in BaP degradation revealed their significant involvement in various metabolic pathways, including starch and sucrose metabolism, tryptophan metabolism, purine metabolism, pyrimidine metabolism, cysteine and methionine metabolism, and valine, leucine, and isoleucine degradation. Previous studies have demonstrated the key role of these pathways in fungal growth [65,66]. Additionally, the enrichment of DNA replication and mismatch repair pathways suggest that the presence of BaP affected the DNA/RNA binding and metabolic process in *P. acerina* S-LWZ20190614-6. These pathways are associated with the production of energy and active enzymes involved in BaP degradation. The degradation pathway of PAHs in fungi involves several processes: (i) initial activation of organic pollutant degradation through redox reactions; (ii) subsequent detoxification; and (iii) mineralization of metabolic intermediates [67]. Each of these bioremediation pathways relies on a specific family of redox enzymes, such as cytochrome P450s and lignin-degrading enzymes. There have been numerous studies reporting the ability of white-rot fungi to degrade PAHs in the environment. However, the details of the gene-based metabolic pathways in the degradation process remain poorly understood, and the transcriptomic analyses of PAH degradation by fungi are rare. Transcriptome analysis of PAH degradation by *Dentipellis* sp. KUC8613 revealed that it primarily utilizes non-lignin-degrading enzymes to remove various PAHs, rather than relying on the typical lignin-degrading enzymes [45]. In our study, only a limited number of lignin-degrading enzymes were found in the upregulated DEGs. However, cytochrome P450s and carbohydrate-active enzymes were upregulated during the degradation of BaP by *P. acerina* S-LWZ20190614-6. Wood-rotting fungi possess an exceptionally large pool of cytochrome P450s in their genomes, which play a crucial role in the degradation of diverse organic pollutants [68,69]. The genome-scale identification of cytochrome P450 from *Trichoderma flavus* suggests a specific oxidative activity towards PAHs [33]. The fungus *Dentipellis* sp. KUC8613 initiated the removal of PAHs through the upregulation of common cytochrome P450s [44]. Our findings indicate that BaP degradation in *P. acerina* S-LWZ20190614-6 occurred through ring-opening oxidation facilitated by epoxide hydrolases, including dehydrogenase, monooxygenase, dioxygenase, reductase, and dehalogenase. Furthermore, genomic analysis of PAH degradation by *Rhodococcus* sp. P14 revealed that PAHs were degraded to acetyl coenzyme A and succinyl coenzyme A, which were further mineralized to CO_2_ via the tricarboxylic acid (TCA) cycle [70]. It is anticipated that after the ring-opening of BaP, mineralization will occur through the TCA cycle.

Treatment with Brij 30 significantly enhanced BaP degradation by *P. acerina* S-LWZ20190614-6. The enriched KEGG pathways were glutathione metabolism, N-glycan biosynthesis, terpenoid backbone biosynthesis, ubiquitin-mediated proteolysis, 2-oxocarboxylic acid metabolism, and ATP-binding cassette (ABC) transporters (Figure 7). The moderate addition of surfactant can help the uptake and degradation of PAHs by cells without affecting cell activity [71,72]. This facilitates the transport of PAH molecules in the micellar phase of surfactants to the vicinity of microbial cells, where they can be utilized by microorganisms. As a result, the solubility and bioavailability of PAHs are increased [59,73]. The Carbohydrate-Active Enzymes Database contains information on enzymes involved in complex carbohydrate assembly (glycosyltransferases) and catabolism (glycoside hydrolases, polysaccharide lyases, and carbohydrate esterases). It provides comprehensive information on carbohydrate-active enzymes involved in the catabolism, modification, and synthesis of glycosidic bonds [74]. Enzymes from the glycoside hydrolase and carbohydrate esterase families are particularly abundant in the degradation of lignocellulosic plant cell wall materials [75,76]. Table S5 presents the upregulated DEGs belonging to carbohydrate-active enzymes in *P. acerina* S-LWZ20190614-6 during the degradation of BaP. In the **BaP** and **NoBaP** comparisons, several genes were found to be upregulated, including the glycosyl hydrolase family, glycosyltransferase, endo-beta-1,4-galactanase, and polysaccharide biosynthesis. In the **Brij30_BaP** and **BaP** comparisons, the upregulated DEGs included the glycosyl hydrolase family and β-glucuronidase. The genes in the glycosyl hydrolase family, glycosyltransferase, and hexosaminidase were upregulated in the **Brij30_BaP** and **NoBaP** comparisons. Furthermore, the comparison between **Brij30_BaP** and **BaP** revealed significant upregulation of genes such as the glycosyl hydrolase family, α/β fold hydrolase, dioxygenase, reductase, dehydrogenase, polypeptide protease, and salicylate hydroxylase. Additionally, it is well known that ABC transporters mediate the import and export of xenobiotics [77]. Therefore, the addition of surfactant Brij 30 led to a significant upregulation of various transferase and binding protein genes in *P. acerina* S-LWZ20190614-6. However, further research is required to elucidate the BaP degradation mechanisms associated with the key enzymes identified in this study.

## 5. Conclusions

The white-rot fungus *P. acerina* S-LWZ20190614-6 demonstrated effective degradation of BaP. The addition of surfactants Brij 30 and Tween 80 promoted the degradation of BaP, but Triton X-100 was counterproductive. The addition of either 1 or 2 g·L^−1^ of the surfactants resulted in near-complete degradation of BaP. The presence of BaP and Brij 30 could increase fungal biomass after 24 days of incubation. Nonetheless, the addition of Brij 30 did not significantly affect the activity of LiP and MnP, although it did reduce laccase activity. Furthermore, the degradation of BaP by *P. acerina* S-LWZ20190614-6 was found to be minimally influenced by cytochrome P450. Additionally, the expressions of cytochrome P450s and carbohydrate-active enzymes were upregulated during the degradation process. GO analysis indicated that the addition of Brij 30 primarily impacted the components and intrinsic constituents of the cell wall and membrane. KEGG enrichment analysis revealed a significant upregulation of various transferase and binding protein genes in *P. acerina* S-LWZ20190614-6 when the surfactant Brij 30 was present. To the best of our knowledge, this study is the first to investigate the potential genes involved in the degradation of BaP by *P. acerina*.

## Data Availability

The sequence information that supports the findings of this study is available in the National Microbiology Data Center (NMDC) with the accession number NMDC 10018312.

## Supplementary Materials

The following supporting information can be downloaded at https://www.mdpi.com/article/10.3390/jof9100978/s1, Figure S1: DEGs in the degradation of BaP by *P. acerina* S-LWZ20190614-6. Blue spots represent genes without significant expression. Red spots mean two-fold upregulated genes. Green spots represent significantly two-fold downregulated genes. a: BaP vs. NoBaP; b: Brij30_BaP vs. BaP; c: Brij30_BaP vs. NoBaP; Figure S2: Venn diagram of the numbers of DEGs found in the comparisons of BaP, NoBaP, and Brij30_BaP in the degradation of BaP by *P. acerina* S-LWZ20190614-6; Figure S3: GO enrichment analysis of upregulated DEGs. a: BaP vs. NoBaP; b: Brij30_BaP vs. BaP; c: Brij30_BaP vs. NoBaP. BP: biological process; CC: cellular component; MF: molecular function; Figure S4: KEGG pathways of upregulated DEGs. a: BaP vs. NoBaP; b: Brij30_BaP vs. BaP; c: Brij30_BaP vs. NoBaP; Table S1: Basic data results of transcriptome analysis; Table S2: Expression of genes in the BaP and NoBaP comparisons; Table S3: Expression of genes in the Brij30_BaP and BaP comparisons; Table S4: Expression of genes in the Brij30_BaP and NoBaP comparisons; Table S5: Primers used for qPCR.
