# Supplementary material for: Biodegradation of Benzo[a]pyrene by a White-Rot Fungus Phlebia acerina: Surfactant-Enhanced Degradation and Possible Genes Involved
Source: J Fungi (Basel). 2023 Sep 28;9(10):978. doi: 10.3390/jof9100978 (PMC10607704; doi:10.3390/jof9100978)
Supplement: Supplementary file 1 [file jof-09-00978-s001.zip › jof-2565652-supplementary.pdf]

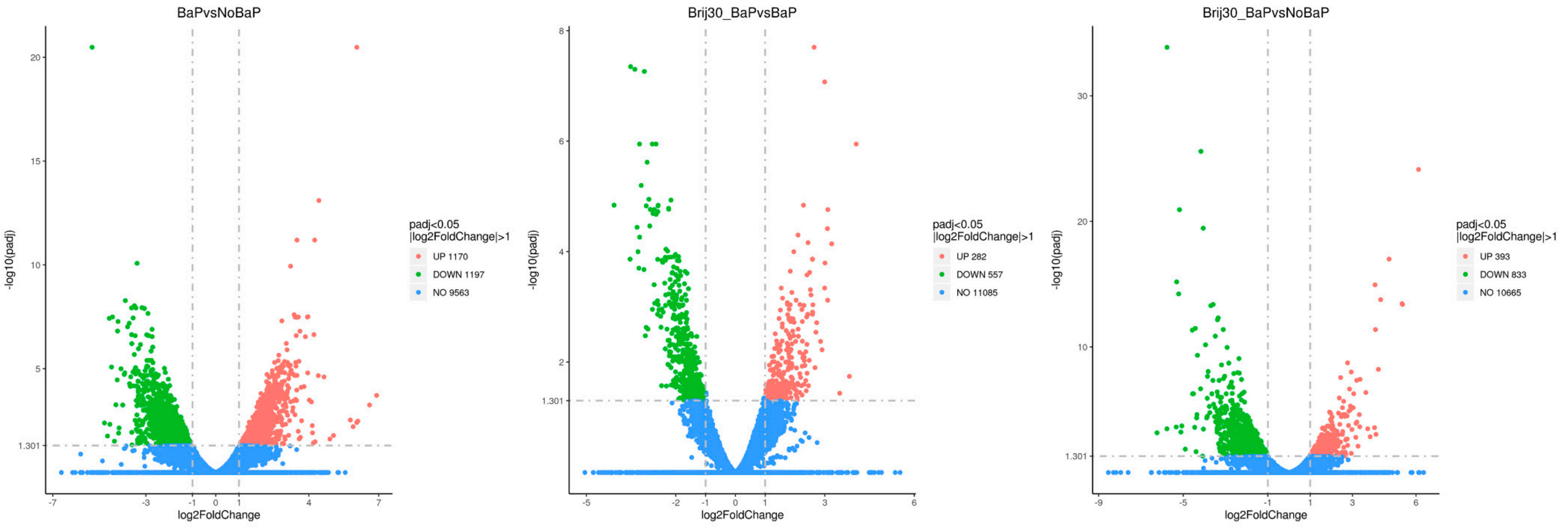

a **b** **c**

Figure S1. DEGs in the degradation of BaP by *P. acerina* S-LWZ20190614-6. Blue spots represent genes without significant expression. Red spots mean 2-fold up-regulated genes. Green spots represent significantly 2-fold down-regulated genes. **a**: BaP vs NoBaP; **b**: Brij30_BaP vs BaP; **c**: Brij30_BaP vs NoBaP.

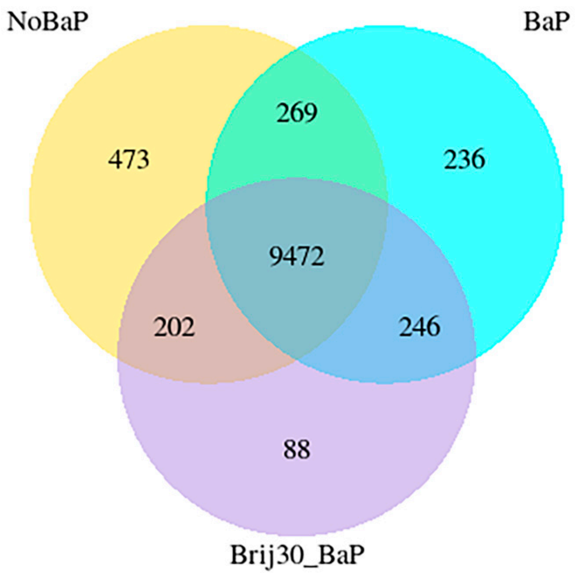

Figure S2. Venn diagram of the numbers of DEGs found in the comparisons of BaP, NoBaP, and Brij30_BaP in the degradation of BaP by *P. acerina* S-LWZ20190614-6.

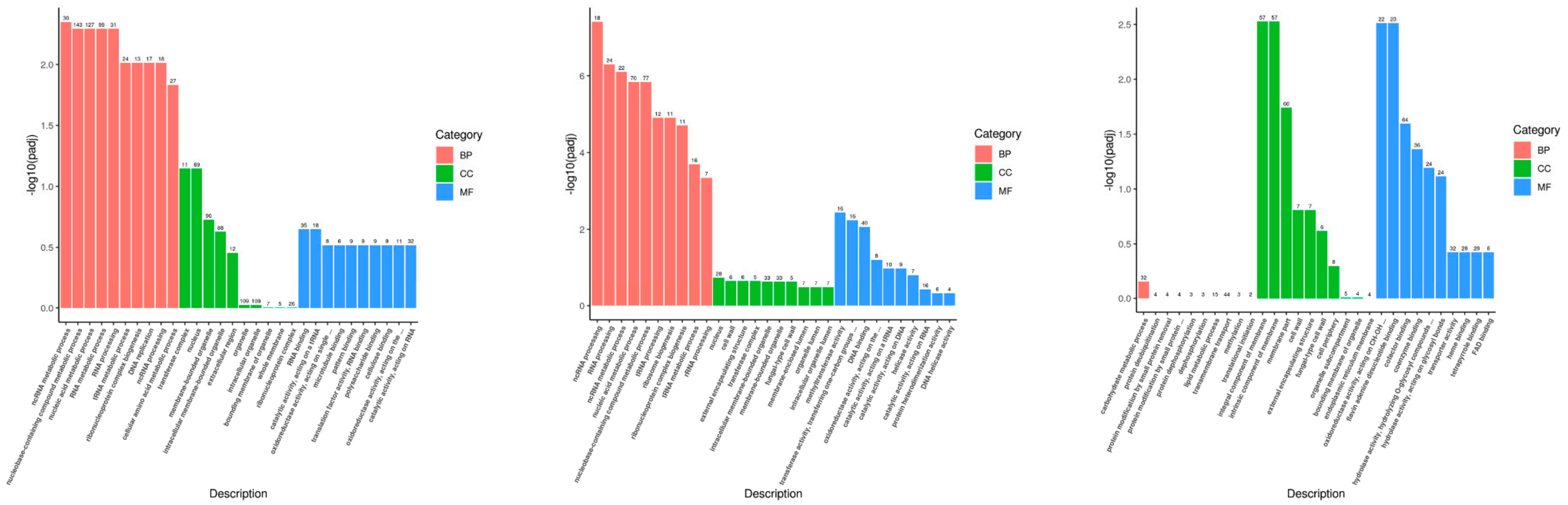

a b c

Figure S3. GO enrichment analysis of upregulated DEGs. **a**: BaP vs NoBaP; **b**: Brij30_BaP vs BaP; **c**: Brij30_BaP vs NoBaP. BP: biological process; CC: cellular component; MF: molecular function.

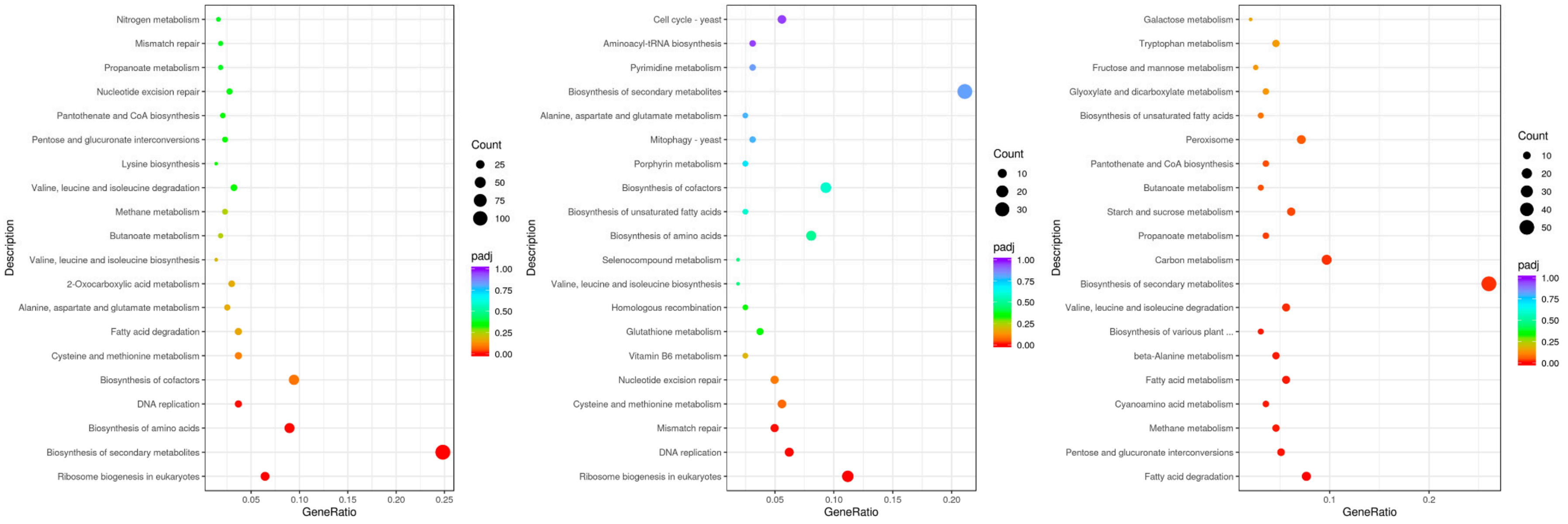

a **b** **c**

Figure S4. Enriched KEGG pathways of upregulated DEGs. **a**: BaP vs NoBaP; **b**: Brij30_BaP vs BaP; **c**: Brij30_BaP vs NoBaP.

Table S1. Basic data results of transcriptome analysis

Sample	Raw Reads	Clean Reads	Clean Bases	Error Rate (%)	Q20 (%)	Q30 (%)	GC Content (%)
NoBaP_1	41273312	39516912	5.93 G	0.03	96.04	90.34	59.66
NoBaP_2	45199146	43880020	6.58 G	0.03	96.03	90.33	59.15
NoBaP_3	41897470	40713928	6.11 G	0.03	95.93	90.09	59.73
BaP_1	43506490	42351378	6.35 G	0.03	96.01	90.28	59.54
BaP_2	40949626	39725090	5.96 G	0.03	95.96	90.19	59.64
BaP_3	42111774	41252706	6.19 G	0.03	96.00	90.24	59.42
Brij30_BaP_1	39584900	38571448	5.79 G	0.03	95.88	89.95	59.54
Brij30_BaP_2	43074160	41917706	6.29 G	0.03	95.97	90.20	59.74
Brij30_BaP_3	42068172	41090796	6.16 G	0.03	95.91	89.96	59.63

Table S2 Expression of genes in the BaP and NoBaP comparisons

gene_id	BaP	NoBaP	$\log_2$FoldChange	*P*value	padj	gene_length	gene_description
Lachesis_group0__13_contigs__l.g8359	1835.348	27.59259	6.060938	3.91E-25	3.30E-21	789	- && Q91XV4.1 RecName: Full=L-xylulose reductase; Short=XR; AltName: Full=Dicarbonyl/L-xylulose reductase; AltName: Full=Sperm antigen P26h && PF00106:short chain dehydrogenase
Lachesis_group0__13_contigs__l.g7710	23.5419	0.358443	5.781563	0.000203	0.002956	933	- && O94480.1 RecName: Full=25S rRNA (uridine-N(3))-methyltransferase && PF10354:Domain of unknown function (DUF2431)
Lachesis_group0__13_contigs__l.g5207	205.9655	11.05565	4.217337	6.61E-10	2.28E-07	954	- && Q5U209.1 RecName: Full=Ribonuclease H2 subunit A; Short=RNase H2 subunit A; AltName: Full=Ribonuclease HI large subunit; Short=RNase HI large subunit; AltName: Full=Ribonuclease HI subunit A && PF01351:Ribonuclease HII
Lachesis_group0__13_contigs__l.g7799	70.87416	3.942875	4.112041	1.26E-05	0.000399	576	- && - && PF05207:CSL zinc finger|PF06912:Protein of unknown function (DUF1275)
Lachesis_group0__13_contigs__l.g10188	223.1278	14.26844	3.951414	1.38E-07	1.59E-05	1290	- && Q5BK68.1 RecName: Full=snRNA-activating protein complex subunit 3; Short=SNAPc subunit 3; AltName: Full=Small nuclear RNA-activating complex polypeptide 3 && PF12251:snRNA-activating protein of 50kDa MW C terminal
Lachesis_group0__13_contigs__l.g8566	302.504	19.81682	3.93838	5.96E-11	3.26E-08	1047	- && - && PF07574:Nse1 non-SMC component of SMC5-6 complex|PF08746:RING-like domain
Lachesis_group0__13_contigs__l.g10926	101.0314	7.153265	3.795126	1.16E-06	7.27E-05	1524	- && P36146.1 RecName: Full=Protein LAS1 && PF04031:Las1-like

Lachesis_group0__13_contigs__l.g5502	233.822	18.95925	3.616171	4.27E-10	1.56E-07	1629	- && Q09887.1 RecName: Full=Uncharacterized amino-acid permease C584.13 && PF13520:Amino acid permease
Lachesis_group0__13_contigs__l.g2225	1060.176	88.81395	3.574578	2.15E-08	4.44E-06	1257	- && Q01752.1 RecName: Full=Aryl-alcohol dehydrogenase [NADP(+)]; Short=AAD && PF00248:Aldo/keto reductase family
Lachesis_group0__13_contigs__l.g4640	583.7243	49.77651	3.548383	5.30E-11	3.23E-08	1881	- && - && PF12937:F-box-like
Lachesis_group0__13_contigs__l.g9363	26.85916	2.226847	3.543837	0.001187	0.010357	1479	- && O69763.1 RecName: Full=Vanillin dehydrogenase && PF00171:Aldehyde dehydrogenase family
Lachesis_group0__13_contigs__l.g4699	27593.83	2456.891	3.489495	2.92E-15	6.40E-12	1041	- && - && PF00702:haloacid dehalogenase-like hydrolase
Lachesis_group0__13_contigs__l.g4744	186.6332	16.73651	3.487963	2.07E-07	2.11E-05	2280	- && Q9BY08.1 RecName: Full=Emopamil-binding protein-like; AltName: Full=Emopamil-binding-related protein && PF05241:Emopamil binding protein
Lachesis_group0__13_contigs__l.g3554	165.046	14.82331	3.48065	2.04E-06	0.000107	753	- && Q9P7C8.1 RecName: Full=DNA replication complex GINS protein sld5 && PF16922:DNA replication complex GINS protein SLD5 C-terminus
Lachesis_group0__13_contigs__l.g4308	68.07137	6.227718	3.474926	9.98E-06	0.000342	630	- && Q8K396.1 RecName: Full=Meiotic nuclear division protein 1 homolog && PF03962:Mnd1 family
Lachesis_group0__13_contigs__l.g5326	926.5655	83.7782	3.467792	6.50E-11	3.39E-08	1788	- && Q870L3.1 RecName: Full=Siderophore iron transporter mirC; AltName: Full=Major facilitator iron-regulated transporter C && -
Lachesis_group0__13_contigs__l.g5556	329.0505	30.16654	3.447901	4.42E-08	7.56E-06	732	- && - && PF03070:TENA/THI-4/PQQC family
Lachesis_group0__13_contigs__l.g5709	133.9542	12.45338	3.432895	2.60E-08	5.01E-06	2832	- && - && PF00533:BRCA1 C Terminus (BRCT) domain|PF12738:twin BRCT domain

Lachesis_group0__13_contigs__l.g4605	130.0551	11.92731	3.427271	8.21E-06	0.000299	1251	- && Q24168.2 RecName: Full=Origin recognition complex subunit 2; Short=DmORC2 && PF04084:Origin recognition complex subunit 2
Lachesis_group0__13_contigs__l.g1296	1341.261	129.7398	3.368592	3.43E-11	2.51E-08	1386	- && C5PEI9.1 RecName: Full=Aspartic protease PEP3; Flags: Precursor && PF00026:Eukaryotic aspartyl protease
Lachesis_group0__13_contigs__l.g11694	98.0378	9.494393	3.350437	1.04E-05	0.000351	741	- && - && PF00808:Histone-like transcription factor (CBF/NF-Y) and archaeal histone
Lachesis_group0__13_contigs__l.g10724	3277.617	326.2902	3.328591	1.64E-07	1.81E-05	1872	- && P11182.3 RecName: Full=Lipoamide acyltransferase component of branched-chain alpha-keto acid dehydrogenase complex, mitochondrial; AltName: Full=52 kDa mitochondrial autoantigen of primary biliary cirrhosis; AltName: Full=Branched chain 2-oxo-acid dehydrogenase complex component E2; Short=BCOADC-E2; AltName: Full=Branched-chain alpha-keto acid dehydrogenase complex component E2; Short=BCKAD-E2; Short=BCKADE2; AltName: Full=Dihydrolipoamide acetyltransferase component of branched-chain alpha-keto acid dehydrogenase complex; AltName: Full=Dihydrolipoamide branched chain transacylase; AltName: Full=Dihydrolipoyllysine-residue (2-methylpropanoyl)transferase; Flags: Precursor && PF00364:Biotin-requiring enzyme|PF00198:2-oxoacid dehydrogenases

							acyltransferase (catalytic domain)|PF02817:e3 binding domain
Lachesis_group0__13_contigs__l.g6383	72.3975	7.115171	3.32028	3.09E-05	0.000754	1746	- && - && PF12937:F-box-like
Lachesis_group0__13_contigs__l.g1968	5604.234	592.9833	3.240111	8.40E-08	1.16E-05	1593	- && G3Y416.1 RecName: Full=Cytochrome P450 monooxygenase yanC; AltName: Full=Yanuthone D synthesis protein C; Flags: Precursor && PF00067:Cytochrome P450
Lachesis_group0__13_contigs__l.g6385	132.9916	14.31714	3.217261	1.22E-05	0.000393	1836	- && - && PF09507:DNA polymerase subunit Cdc27
Lachesis_group0__13_contigs__l.g8222	2100.67	226.0516	3.21489	7.38E-14	1.16E-10	2214	- && Q8TB72.2 RecName: Full=Pumilio homolog 2; Short=Pumilio-2 && -
Lachesis_group0__13_contigs__l.g2419	263.2053	29.43699	3.152931	1.88E-07	2.00E-05	1119	- && P0CS31.1 RecName: Full=Probable cytosolic iron-sulfur protein assembly protein 1 >P0CS30.1 RecName: Full=Probable cytosolic iron-sulfur protein assembly protein 1 && PF00400:WD domain, G-beta repeat
Lachesis_group0__13_contigs__l.g8360	101.9323	11.48358	3.134795	1.47E-06	8.49E-05	699	- && Q6NUE2.1 RecName: Full=Carbonyl reductase family member 4; AltName: Full=3-ketoacyl-[acyl-carrier-protein] reductase beta subunit; Short=KAR beta subunit; AltName: Full=3-oxoacyl-[acyl-carrier-protein] reductase; AltName: Full=Quinone reductase CBR4 && PF00106:short chain dehydrogenase
Lachesis_group0__13_contigs__l.g1944	3101.123	353.9786	3.130156	3.44E-07	3.04E-05	1350	- && Q55DV9.1 RecName: Full=Cystathionine gamma-lyase; AltName: Full=Gamma-cystathionase && PF01053:Cys/Met metabolism PLP-dependent enzyme

Lachesis_group0__13_contigs__l.g10956	249.8367	29.0847	3.104122	3.76E-08	6.78E-06	1035	- && - && PF06775:Putative adipose-regulatory protein (Seipin)
Lachesis_group0__13_contigs__l.g5341	132.83	15.56509	3.088315	6.70E-06	0.000258	903	- && Q9P4T7.1 RecName: Full=Thymidylate synthase; Short=TS; Short=TSase && PF00303:Thymidylate synthase
Lachesis_group0__13_contigs__l.g9052	206.9132	24.16362	3.085894	9.69E-05	0.001772	1140	- && - && PF13878:zinc-finger of acetyl-transferase ESCO|PF13880:ESCO1/2 acetyl-transferase
Lachesis_group0__13_contigs__l.g9890	445.469	52.69428	3.081877	2.60E-08	5.01E-06	1824	- && Q9C0V0.1 RecName: Full=Probable amino-acid permease PB1C11.02 && PF00324:Amino acid permease
Lachesis_group0__13_contigs__l.g6805	132.6019	15.66172	3.077456	2.40E-06	0.000121	906	- && - && PF08167:rRNA processing/ribosome biogenesis
Lachesis_group0__13_contigs__l.g5558	1132.719	138.7008	3.030905	2.16E-09	6.08E-07	2619	- && Q9C1X1.1 RecName: Full=Periodic tryptophan protein 2 homolog && PF00400:WD domain, G-beta repeat|PF04003:Dip2/Utp12 Family
Lachesis_group0__13_contigs__l.g3665	93.1149	11.56388	3.024615	2.69E-05	0.000688	978	- && Q99145.1 RecName: Full=ATP phosphoribosyltransferase; Short=ATP-PRT; Short=ATP-PRTase && PF08029:HisG, C-terminal domain|PF01634:ATP phosphoribosyltransferase
Lachesis_group0__13_contigs__l.g7754	54.49373	6.653595	3.003825	0.000394	0.004729	3285	- && P0CQ67.1 RecName: Full=DNA repair protein RAD5 && PF00271:Helicase conserved C-terminal domain|PF00176:SNF2 family N-terminal domain|PF08797:HIRAN domain
Lachesis_group0__13_contigs__l.g6363	75.22285	9.40911	2.993515	1.59E-05	0.000484	3243	- && O14343.1 RecName: Full=Kinesin-like protein 5 && PF00225:Kinesin motor domain
Lachesis_group0__13_contigs__l.g6668	110.9872	14.17146	2.977285	8.31E-06	0.0003	1770	- && - && PF12937:F-box-like

Lachesis_group0__13_contigs__l.g5609	452.3297	57.37736	2.973489	4.12E-06	0.000184	2427	- && A6QPL4.2 RecName: Full=Kinesin-like protein KIF22 && PF00225:Kinesin motor domain
Lachesis_group0__13_contigs__l.g3366	510.9987	65.49202	2.968314	1.33E-07	1.57E-05	963	- && Q6BDR8.1 RecName: Full=Non-structural maintenance of chromosome element 4; Short=Non-SMC element 4; AltName: Full=DNA repair protein rad62 && PF08743:Nse4 C-terminal|PF15412:Binding domain of Nse4/EID3 to Nse3-MAGE
Lachesis_group0__13_contigs__l.g7937	1857.48	239.1627	2.958372	2.22E-08	4.51E-06	1032	- && A8N2Z9.1 RecName: Full=Elongation factor Ts, mitochondrial; Short=EF-Ts; Short=EF-TsMt; Flags: Precursor && PF00889:Elongation factor TS
Lachesis_group0__13_contigs__l.g7696	489.312	63.64049	2.944399	4.69E-07	3.99E-05	1758	- && Q0UG00.1 RecName: Full=ATP-dependent RNA helicase MSS116, mitochondrial; Flags: Precursor && PF00271:Helicase conserved C-terminal domain|PF00270:DEAD/DEAH box helicase
Lachesis_group0__13_contigs__l.g9084	146.8231	19.31304	2.920176	8.23E-06	0.000299	3285	- && - && PF08457:Sfi1 spindle body protein
Lachesis_group0__13_contigs__l.g5348	409.5437	54.79667	2.906774	7.69E-06	0.000286	3462	- && Q06680.2 RecName: Full=Condensin complex subunit 3; AltName: Full=CAPG homolog && PF13646:HEAT repeats|PF12719:Nuclear condensing complex subunits, C-term domain
Lachesis_group0__13_contigs__l.g4696	12324.72	1647.556	2.903274	4.33E-08	7.53E-06	798	- && O23522.2 RecName: Full=Phospholipase A1-Ibeta2, chloroplastic; Flags: Precursor && PF01764:Lipase (class 3)
Lachesis_group0__13_contigs__l.g5221	189.096	25.36848	2.88958	2.16E-05	0.000592	3504	- && O94751.1 RecName: Full=Checkpoint serine/threonine-protein kinase bub1 && PF00069:Protein kinase domain|PF08311:Mad3/BUB1 homology region 1

Lachesis_group0__13_contigs__l.g9851	122.1368	16.62223	2.884063	4.77E-05	0.001035	1155	- && Q14AI0.1 RecName: Full=Sister chromatid cohesion protein DCC1; AltName: Full=Defective in sister chromatid cohesion protein 1 homolog && PF09724:Uncharacterized conserved protein (DUF2036)
Lachesis_group0__13_contigs__l.g3306	114.469	15.54949	2.882176	3.05E-06	0.000146	306	- && Q4P8G2.1 RecName: Full=Diphthamide biosynthesis protein 3 && PF05207:CSL zinc finger
Lachesis_group0__13_contigs__l.g1086	2169.552	296.7473	2.870944	3.32E-08	6.16E-06	2319	- && Q80Z60.2 RecName: Full=Endothelin-converting enzyme 2; Short=ECE-2; Includes: RecName: Full=Methyltransferase-like region; Includes: RecName: Full=Endothelin-converting enzyme 2 region && PF08241:Methyltransferase domain
Lachesis_group0__13_contigs__l.g2237	690.3075	94.61281	2.86404	1.16E-07	1.45E-05	1740	- && - && PF12937:F-box-like
Lachesis_group0__13_contigs__l.g4067	922.7464	127.1798	2.860961	1.16E-07	1.45E-05	2370	- && O13956.1 RecName: Full=Ribosome biogenesis protein tsr1 && PF04950:40S ribosome biogenesis protein Tsr1 and BMS1 C-terminal|PF08142:AARP2CN (NUC121) domain
Lachesis_group0__13_contigs__l.g2258	347.0618	47.60296	2.86032	6.41E-07	4.95E-05	3666	- && P0CR92.1 RecName: Full=Topoisomerase 1-associated factor 1 && PF05029:Timeless protein C terminal region|PF04821:Timeless protein
Lachesis_group0__13_contigs__l.g9771	22.49095	3.058015	2.857078	0.007801	0.039844	1323	- && Q91727.1 RecName: Full=Cyclin-dependent kinase 4; AltName: Full=Cell division protein kinase 4 && PF00069:Protein kinase domain
Lachesis_group0__13_contigs__l.g9344	360.9619	50.31784	2.844853	5.42E-07	4.31E-05	1707	- && - && PF09739:Mini-chromosome maintenance replisome factor
Lachesis_group0__13_contigs__l.g5251	176.4204	24.6557	2.837668	0.000179	0.002674	1077	- && Q8X082.1 RecName: Full=Replication factor C subunit 5; Short=Replication factor C5; AltName:

							Full=Probable activator 1 subunit 5 && PF08542:Replication factor C C-terminal domain|PF13177:DNA polymerase III, delta subunit
Lachesis_group0__13_contigs__l.g951	143.3845	20.14182	2.837009	9.13E-07	6.18E-05	1287	- && Q10498.1 RecName: Full=Protein HGH1 homolog && PF04063:Domain of unknown function (DUF383)|PF04064:Domain of unknown function (DUF384)
Lachesis_group0__13_contigs__l.g4034	615.0993	86.34085	2.834361	6.87E-08	1.00E-05	1011	- && Q10248.2 RecName: Full=Uncharacterized mitochondrial carrier C4G9.20c && PF00153:Mitochondrial carrier protein
Lachesis_group0__13_contigs__l.g9173	222.3818	31.13482	2.832851	2.31E-06	0.000118	1332	- && Q6C656.1 RecName: Full=Chromosome segregation in meiosis protein 3 && PF07962:Replication Fork Protection Component Swi3
Lachesis_group0__13_contigs__l.g7345	129.3869	18.20666	2.816263	0.000302	0.003879	336	- && - && PF07019:Rab5-interacting protein (Rab5ip)
Lachesis_group0__13_contigs__l.g2754	82.84506	11.68965	2.808974	0.000143	0.002318	1233	- && - && PF13414:TPR repeat
Lachesis_group0__13_contigs__l.g6835	2536.905	362.2934	2.808595	1.25E-07	1.50E-05	1665	- && P28745.2 RecName: Full=Protein pim1; AltName: Full=Poly(A)+ RNA transport protein 2 && PF00415:Regulator of chromosome condensation (RCC1) repeat
Lachesis_group0__13_contigs__l.g8691	429.9706	61.66128	2.799023	7.65E-07	5.57E-05	1098	- && Q54S38.2 RecName: Full=Probable RNA 3'-terminal phosphate cyclase-like protein && PF01137:RNA 3'-terminal phosphate cyclase|PF05189:RNA 3'-terminal phosphate cyclase (RTC), insert domain

Lachesis_group0__13_contigs__l.g3389	1384.37	199.2006	2.7955	4.74E-08	7.86E-06	1875	- && O94528.1 RecName: Full=Caffeine resistance protein 5 && PF07690:Major Facilitator Superfamily
Lachesis_group0__13_contigs__l.g6499	9048.043	1309.316	2.788974	0.000121	0.002062	1197	- && Q55E72.1 RecName: Full=Probable polyketide synthase 1; Short=dipks1; Includes: RecName: Full=Polyketide synthase stlA; Includes: RecName: Full=Chalcone synthase stlA; AltName: Full=Steely1 && PF02797:Chalcone and stilbene synthases, C-terminal domain|PF00195:Chalcone and stilbene synthases, N-terminal domain
Lachesis_group0__13_contigs__l.g10646	94.97398	13.74593	2.786002	5.57E-05	0.001164	750	- && B6JGU7.1 RecName: Full=ATP-dependent Clp protease proteolytic subunit; AltName: Full=Endopeptidase Clp && PF00574:Clp protease
Lachesis_group0__13_contigs__l.g8807	195.7033	28.36371	2.782586	5.38E-06	0.000223	2754	- && - && PF12253:Chromatin assembly factor 1 subunit A
Lachesis_group0__13_contigs__l.g7346	1842.914	275.1056	2.744166	6.51E-08	9.64E-06	1116	- && P78780.2 RecName: Full=Probable aspartate-semialdehyde dehydrogenase; Short=ASA dehydrogenase; Short=ASADH; AltName: Full=Aspartate-beta-semialdehyde dehydrogenase && PF01118:Semialdehyde dehydrogenase, NAD binding domain|PF02774:Semialdehyde dehydrogenase, dimerisation domain
Lachesis_group0__13_contigs__l.g7501	60.14427	8.945481	2.728351	0.001297	0.011073	294	- && - && PF05730:CFEM domain
Lachesis_group0__13_contigs__l.g11359	795.5687	120.4317	2.724598	1.18E-07	1.45E-05	2445	- && Q9P7D4.3 RecName: Full=Homocitrate dehydratase, mitochondrial; AltName: Full=Aconitase 2; Flags: Precursor && PF00694:Aconitase C-terminal

							domain|PF00330:Aconitase family (aconitate hydratase)
Lachesis_group0__13_contigs__l.g4951	176.8857	26.72646	2.721072	1.24E-05	0.000397	690	- && - && PF09725:Folate-sensitive fragile site protein Fra10Ac1
Lachesis_group0__13_contigs__l.g10555	131.7805	19.93815	2.720859	9.07E-06	0.000322	3834	- && Q96GN5.2 RecName: Full=Cell division cycle-associated 7-like protein; AltName: Full=Protein JPO2; AltName: Full=Transcription factor RAM2 && PF10497:Zinc-finger domain of monoamine-oxidase A repressor R1
Lachesis_group0__13_contigs__l.g6547	95.78278	14.62654	2.718073	6.45E-05	0.001306	1080	- && - && PF00011:Hsp20/alpha crystallin family
Lachesis_group0__13_contigs__l.g4546	2001.609	306.198	2.709012	9.58E-09	2.23E-06	1956	- && Q9UT32.1 RecName: Full=Putative ribosome biogenesis protein C8F11.04; AltName: Full=U3 snoRNP-associated protein C8F11.04 && PF00687:Ribosomal protein L1p/L10e family
Lachesis_group0__13_contigs__l.g9214	2411.74	369.2458	2.707418	8.56E-07	5.90E-05	1455	- && P38680.2 RecName: Full=N amino acid transport system protein; AltName: Full=Methyltryptophan resistance protein && PF01490:Transmembrane amino acid transporter protein
Lachesis_group0__13_contigs__l.g9227	1137.408	174.303	2.706898	1.16E-07	1.45E-05	1623	- && P87041.3 RecName: Full=UDP-galactose transporter; AltName: Full=Golgi UDP-Gal transporter && PF04142:Nucleotide-sugar transporter
Lachesis_group0__13_contigs__l.g10832	1512.405	235.4435	2.683336	1.95E-08	4.12E-06	1794	- && P78750.3 RecName: Full=Probable U3 small nucleolar RNA-associated protein 18; Short=U3 snoRNA-associated protein 18 && -
Lachesis_group0__13_contigs__l.g3907	381.2808	59.50524	2.678041	2.34E-07	2.27E-05	1695	- && - && PF12937:F-box-like

Lachesis_group0__13_contigs__l.g344	1550.619	243.5228	2.670875	2.88E-06	0.00014	1665	- && P53388.1 RecName: Full=Dicarboxylic amino acid permease && PF00324:Amino acid permease
Lachesis_group0__13_contigs__l.g9619	75.65426	12.0721	2.670699	0.001182	0.01035	876	- && - && PF12328:Rpp20 subunit of nuclear RNase MRP and P
Lachesis_group0__13_contigs__l.g11157	364.2258	57.5447	2.664493	6.15E-08	9.49E-06	1179	- && Q8Z8W3.1 RecName: Full=2-dehydropantoate 2-reductase; AltName: Full=Ketopantoate reductase; Short=KPA reductase; Short=KPR && PF02558:Ketopantoate reductase PanE/ApbA|PF08546:Ketopantoate reductase PanE/ApbA C terminal
Lachesis_group0__13_contigs__l.g4688	840.6323	132.71	2.664394	8.14E-07	5.69E-05	822	- && Q96DG6.1 RecName: Full=Carboxymethylenebutenolidase homolog && PF01738:Dienelactone hydrolase family
Lachesis_group0__13_contigs__l.g148	386.8719	61.25437	2.659553	8.87E-07	6.07E-05	1245	- && P49321.2 RecName: Full=Nuclear autoantigenic sperm protein; Short=NASP && PF10516:SHNi-TPR
Lachesis_group0__13_contigs__l.g10392	458.3329	72.51041	2.656731	0.000164	0.002534	3291	- && Q04336.1 RecName: Full=Uncharacterized protein YMR196W && PF03200:Glycosyl hydrolase family 63 C-terminal domain
Lachesis_group0__13_contigs__l.g9486	331.0712	52.64166	2.651585	3.57E-06	0.000163	1944	- && O94481.2 RecName: Full=Transcription factor TFIIIB component B''; AltName: Full=Transcription factor TFIIIB complex subunit bdp1 && PF15963:Myb DNA-binding like
Lachesis_group0__13_contigs__l.g4398	432.3211	69.21015	2.640352	1.73E-06	9.57E-05	1752	- && D7PHY8.1 RecName: Full=Efflux pump vrtL; AltName: Full=Viridicatumtoxin synthesis protein L && PF07690:Major Facilitator Superfamily
Lachesis_group0__13_contigs__l.g6392	741.8378	119.0702	2.637274	0.000274	0.003654	1167	- && O13991.3 RecName: Full=Uncharacterized oxidoreductase C26H5.09c &&

							PF01408:Oxidoreductase family, NAD-binding Rossmann fold
Lachesis_group0__13_contigs__l.g8861	1189.567	192.1538	2.629223	1.36E-06	8.11E-05	1833	- && Q92372.1 RecName: Full=Replication factor A protein 1; AltName: Full=Single-stranded DNA-binding protein p68 subunit && PF01336:OB-fold nucleic acid binding domain|PF04057:Replication factor-A protein 1, N-terminal domain|PF08646:Replication factor-A C terminal domain|PF16900:Replication protein A OB domain
Lachesis_group0__13_contigs__l.g2514	1283.549	207.6275	2.628834	2.44E-07	2.33E-05	1356	- && B0DWM8.1 RecName: Full=Ribosome biogenesis protein YTM1 && PF08154:NLE (NUC135) domain
Lachesis_group0__13_contigs__l.g6441	131.9298	21.36111	2.616033	0.002503	0.017661	1149	- && Q9C167.2 RecName: Full=Ribonucleoside-diphosphate reductase small chain; AltName: Full=Ribonucleotide reductase small subunit && PF00268:Ribonucleotide reductase, small chain
Lachesis_group0__13_contigs__l.g6847	1945.191	317.1988	2.615569	0.00016	0.002502	771	- && Q12335.1 RecName: Full=Protoplast secreted protein 2; Flags: Precursor && PF03358:NADPH-dependent FMN reductase
Lachesis_group0__13_contigs__l.g6885	947.7798	155.7639	2.604541	1.98E-06	0.000105	1770	- && P40186.3 RecName: Full=PHO85 cyclin-7; AltName: Full=PHO85-associated protein 1 && PF08613:Cyclin
Lachesis_group0__13_contigs__l.g8694	148.3837	24.58402	2.603171	0.000149	0.002381	1278	- && O08760.2 RecName: Full=N-glycosylase/DNA lyase; Includes: RecName: Full=8-oxoguanine DNA glycosylase; Includes: RecName: Full=DNA-(apurinic or apyrimidinic site) lyase; Short=AP lyase && PF07934:8-oxoguanine DNA glycosylase,

							N-terminal domain|PF00730:HhH-GPD superfamily base excision DNA repair protein
Lachesis_group0__13_contigs__l.g3189	233.6559	38.88536	2.592989	6.52E-06	0.000253	1437	- && B5FXE5.1 RecName: Full=Apoptosis-inducing factor 2 && PF07992:Pyridine nucleotide-disulphide oxidoreductase
Lachesis_group0__13_contigs__l.g10487	27.77236	4.529881	2.592101	0.008636	0.042825	561	- && - && PF01753:MYND finger
Lachesis_group0__13_contigs__l.g11644	183.3396	30.60117	2.584014	3.33E-05	0.000793	1089	- && O94449.1 RecName: Full=Replication factor C subunit 4; Short=Replication factor C4 && PF00004:ATPase family associated with various cellular activities (AAA)|PF08542:Replication factor C C-terminal domain
Lachesis_group0__13_contigs__l.g6500	424.6058	71.0361	2.582547	2.92E-05	0.000728	1239	- && P48407.1 RecName: Full=Pinosylvin synthase 1; AltName: Full=Dihydropinosylvin synthase 1; AltName: Full=Stilbene synthase 1; Short=STS 1 && PF00195:Chalcone and stilbene synthases, N-terminal domain|PF02797:Chalcone and stilbene synthases, C-terminal domain
Lachesis_group0__13_contigs__l.g4738	37.287	6.192217	2.579608	0.006846	0.036162	3234	- && B4J3F1.1 RecName: Full=Serine/threonine-protein kinase PLK4; AltName: Full=Polo-like kinase 4; Short=PLK-4; AltName: Full=Serine/threonine-protein kinase SAK && PF00069:Protein kinase domain
Lachesis_group0__13_contigs__l.g1957	1298.38	217.184	2.579183	2.19E-07	2.16E-05	1656	- && Q9USK3.1 RecName: Full=Uncharacterized transporter C4B3.13 && PF01554:MatE
Lachesis_group0__13_contigs__l.g6908	716.1454	120.2783	2.573301	3.30E-07	2.94E-05	1035	- && - && PF02777:Iron/manganese superoxide dismutases, C-terminal domain
Lachesis_group0__13_contigs__l.g7753	1466.049	247.0228	2.570429	5.78E-06	0.000234	1677	- && P87153.1 RecName: Full=Probable T-complex protein 1 subunit eta; Short=TCP-1-eta; AltName:

							Full=CCT-eta && PF00118:TCP-1/cpn60 chaperonin family
Lachesis_group0__13_contigs__l.g10719	20.98632	3.514944	2.569135	0.009556	0.04566	903	- && - && PF00069:Protein kinase domain
Lachesis_group0__13_contigs__l.g10133	490.8132	82.77515	2.56814	8.07E-07	5.69E-05	960	- && Q09704.1 RecName: Full=Exosome complex component rrp4; AltName: Full=Ribosomal RNA-processing protein 4 && PF14382:Exosome complex exonuclease RRP4 N-terminal region|PF15985:KH domain
Lachesis_group0__13_contigs__l.g10511	4920.251	831.8664	2.564613	7.77E-07	5.57E-05	1890	- && P87142.3 RecName: Full=Heat shock protein 70 homolog C57A7.12 && PF00012:Hsp70 protein|PF10712:NAD-specific glutamate dehydrogenase
Lachesis_group0__13_contigs__l.g10180	158.2567	26.68558	2.564365	0.000217	0.003104	1005	- && - && PF09468:Ydr279p protein family (RNase H2 complex component)
Lachesis_group0__13_contigs__l.g4468	1009.385	170.8594	2.56342	5.39E-07	4.31E-05	1083	- && O13648.3 RecName: Full=Ribosomal protein arginine N-methyltransferase rmt3 && PF06325:Ribosomal protein L11 methyltransferase (PrmA)
Lachesis_group0__13_contigs__l.g9031	1342.986	227.5912	2.561946	1.55E-06	8.89E-05	1389	- && P50514.1 RecName: Full=Probable argininosuccinate lyase; Short=ASAL; AltName: Full=Arginosuccinase && PF00206:Lyase|PF14698:Argininosuccinate lyase C-terminal
Lachesis_group0__13_contigs__l.g5796	3262.543	553.9272	2.558689	1.69E-06	9.49E-05	1803	- && Q05506.1 RecName: Full=Arginine--tRNA ligase, cytoplasmic; AltName: Full=Arginyl-tRNA synthetase; Short=ArgRS && PF00750:tRNA synthetases class I (R)|PF05746:DALR anticodon binding domain

Lachesis_group0__13_contigs__l.g3446	41.01635	6.909103	2.556389	0.004901	0.028534	636	- && - && PF12937:F-box-like
Lachesis_group0__13_contigs__l.g3417	203.3038	34.86034	2.547843	1.88E-05	0.000544	630	- && - && PF08641:Kinetochore protein Mis14 like
Lachesis_group0__13_contigs__l.g2428	6365.295	1098.262	2.535209	3.91E-07	3.37E-05	1437	- && O23254.1 RecName: Full=Serine hydroxymethyltransferase 4; Short=AtSHMT4; AltName: Full=Glycine hydroxymethyltransferase 4; AltName: Full=Serine methylase 4 && PF00464:Serine hydroxymethyltransferase
Lachesis_group0__13_contigs__l.g2226	1383.165	239.9825	2.528469	4.92E-07	4.05E-05	1158	- && Q01752.1 RecName: Full=Aryl-alcohol dehydrogenase [NADP(+)]; Short=AAD && PF00248:Aldo/keto reductase family
Lachesis_group0__13_contigs__l.g8804	1477.205	256.2661	2.527914	3.18E-06	0.00015	1506	- && O94582.1 RecName: Full=Probable anthranilate synthase component 1; AltName: Full=Anthranilate synthase component I && PF04715:Anthranilate synthase component I, N terminal region|PF00425:chorismate binding enzyme
Lachesis_group0__13_contigs__l.g5734	6489.737	1126.98	2.525743	2.54E-08	5.01E-06	1470	- && Q9UQW9.1 RecName: Full=Ornithine decarboxylase; Short=ODC && PF02784:Pyridoxal-dependent decarboxylase, pyridoxal binding domain|PF00278:Pyridoxal-dependent decarboxylase, C-terminal sheet domain
Lachesis_group0__13_contigs__l.g4764	1557.059	272.3287	2.516256	9.21E-07	6.19E-05	2367	- && O74879.1 RecName: Full=Ribosome biogenesis protein enp2 homolog && PF08159:NUC153 domain
Lachesis_group0__13_contigs__l.g2706	119.6346	20.7741	2.513731	0.004318	0.026026	645	- && - && PF16015:Promethin
Lachesis_group0__13_contigs__l.g7204	1356.619	238.1187	2.511062	8.21E-07	5.69E-05	1422	- && Q9UTK0.1 RecName: Full=20S-pre-rRNA D-site endonuclease nob1; AltName: Full=Pre-rRNA-

							processing endonuclease nob1 && PF08772:Nin one binding (NOB1) Zn-ribbon like
Lachesis_group0__13_contigs__l.g11397	586.3343	103.9905	2.497319	3.30E-06	0.000153	300	- && P0CR99.1 RecName: Full=Mitochondrial import inner membrane translocase subunit TIM10 >P0CR98.1 RecName: Full=Mitochondrial import inner membrane translocase subunit TIM10 && PF02953:Tim10/DDP family zinc finger
Lachesis_group0__13_contigs__l.g3687	296.8936	52.77477	2.497172	1.83E-05	0.000536	372	- && Q09723.1 RecName: Full=Multifunctional methyltransferase subunit trm112; AltName: Full=eRF1 methyltransferase subunit trm112; Short=eRF1 MTase subunit trm112; AltName: Full=tRNA methyltransferase 112 homolog && PF03966:Trm112p-like protein
Lachesis_group0__13_contigs__l.g113	71.18328	12.63715	2.492582	9.71E-05	0.001772	1209	- && - && PF10294:Lysine methyltransferase
Lachesis_group0__13_contigs__l.g1025	884.6977	157.6804	2.489437	6.14E-06	0.000243	6777	- && Q7S1P9.3 RecName: Full=DNA repair protein rad5; AltName: Full=Mutagen-sensitive protein 41 && PF00271:Helicase conserved C-terminal domain|PF00145:C-5 cytosine-specific DNA methylase|PF00176:SNF2 family N-terminal domain
Lachesis_group0__13_contigs__l.g2350	302.4759	54.00467	2.488308	5.84E-06	0.000235	1329	- && P53011.1 RecName: Full=Nucleoporin SEH1; AltName: Full=Nuclear pore protein SEH1; AltName: Full=SEC13 homolog 1 && -
Lachesis_group0__13_contigs__l.g3747	92.25084	16.43249	2.485046	0.00049	0.005531	450	- && Q8HYI9.1 RecName: Full=Prefoldin subunit 5 && PF02996:Prefoldin subunit
Lachesis_group0__13_contigs__l.g10984	1625.207	291.1908	2.481601	1.82E-06	9.87E-05	972	- && O74919.1 RecName: Full=RNA-binding protein rnc1; AltName: Full=RNA-binding protein

							that suppresses calcineurin deletion 1 && PF00013:KH domain
Lachesis_group0__13_contigs__l.g7977	598.7635	107.4099	2.480898	1.89E-06	0.000102	1389	- && O74731.1 RecName: Full=tRNA-dihydrouridine(20) synthase [NAD(P)+]; AltName: Full=tRNA-dihydrouridine synthase 2 && PF01207:Dihydrouridine synthase (Dus)
Lachesis_group0__13_contigs__l.g466	611.8194	110.3067	2.473566	9.26E-06	0.000324	1467	- && Q4PHV3.1 RecName: Full=Pre-rRNA-processing protein IPI3 && -
Lachesis_group0__13_contigs__l.g1201	105.2085	18.9678	2.471741	0.000331	0.00415	1233	- && Q9UTK3.1 RecName: Full=Probable nicotinate phosphoribosyltransferase; Short=NAPRTase && PF04095:Nicotinate phosphoribosyltransferase (NAPRTase) family
Lachesis_group0__13_contigs__l.g11522	52.93536	9.433803	2.464389	0.008321	0.041733	2631	- && Q96Q89.3 RecName: Full=Kinesin-like protein KIF20B; AltName: Full=Cancer/testis antigen 90; Short=CT90; AltName: Full=Kinesin family member 20B; AltName: Full=Kinesin-related motor interacting with PIN1; AltName: Full=M-phase phosphoprotein 1; Short=MPP1 && PF00225:Kinesin motor domain
Lachesis_group0__13_contigs__l.g11437	526.1124	95.64892	2.462523	2.96E-05	0.000731	2559	- && Q9W032.1 RecName: Full=Protein ecdysoneless && PF07093:SGT1 protein
Lachesis_group0__13_contigs__l.g248	1279.921	235.1769	2.444593	2.43E-07	2.33E-05	2244	- && - && PF12767:Transcriptional regulator of RNA polII, SAGA, subunit|PF05132:RNA polymerase III RPC4
Lachesis_group0__13_contigs__l.g5955	85.90041	15.67732	2.442131	0.000153	0.002426	1554	- && P49374.1 RecName: Full=High-affinity glucose transporter && PF00083:Sugar (and other) transporter

Lachesis_group0__13_contigs__l.g9457	1614.481	297.2786	2.442045	5.10E-06	0.000215	3606	- && - && PF03177:Non-repetitive/WGA-negative nucleoporin C-terminal|PF08801:Nup133 N terminal like
Lachesis_group0__13_contigs__l.g5672	710.0869	131.4299	2.433514	2.87E-06	0.00014	801	- && P0CS81.1 RecName: Full=tRNA (guanine-N(7)-)-methyltransferase; AltName: Full=Transfer RNA methyltransferase 8; AltName: Full=tRNA (guanine(46)-N(7))-methyltransferase; AltName: Full=tRNA(m7G46)-methyltransferase >P0CS80.1 RecName: Full=tRNA (guanine-N(7)-)-methyltransferase; AltName: Full=Transfer RNA methyltransferase 8; AltName: Full=tRNA (guanine(46)-N(7))-methyltransferase; AltName: Full=tRNA(m7G46)-methyltransferase && PF02390:Putative methyltransferase
Lachesis_group0__13_contigs__l.g10934	625.1385	116.1922	2.428281	1.60E-07	1.80E-05	3729	- && Q04217.1 RecName: Full=Probable ATP-dependent RNA helicase DHR1; AltName: Full=DEAH box RNA helicase DHR1; AltName: Full=Extracellular mutant protein 16 && PF07717:Oligonucleotide/oligosaccharide-binding (OB)-fold|PF00270:DEAD/DEAH box helicase|PF00271:Helicase conserved C-terminal domain|PF04408:Helicase associated domain (HA2)
Lachesis_group0__13_contigs__l.g3864	261.8435	48.6934	2.42824	4.17E-06	0.000186	1710	- && - && PF12937:F-box-like
Lachesis_group0__13_contigs__l.g9037	328.2249	61.08449	2.423093	1.95E-05	0.000552	1494	- && P0CS57.1 RecName: Full=DNA damage-binding protein CMR1 >P0CS56.1 RecName: Full=DNA damage-binding protein CMR1 && -

Lachesis_group0__13_contigs__l.g339	106.5062	19.8822	2.411533	0.000117	0.002006	1032	- && Q96RS0.3 RecName: Full=Trimethylguanosine synthase; AltName: Full=CLL-associated antigen KW-2; AltName: Full=Cap-specific guanine-N2 methyltransferase; AltName: Full=Hepatocellular carcinoma-associated antigen 137; AltName: Full=Nuclear receptor coactivator 6-interacting protein; AltName: Full=PRIP-interacting protein with methyltransferase motif; Short=PIMT; Short=PIPMT && PF09445:RNA cap guanine-N2 methyltransferase
Lachesis_group0__13_contigs__l.g4250	661.2622	124.4959	2.409053	1.31E-05	0.000411	1431	- && O94636.2 RecName: Full=tRNA (guanine(10)-N2)-methyltransferase; AltName: Full=tRNA guanosine-2'-O-methyltransferase TRM11; Short=tRNA methylase 11 && PF13659:Methyltransferase domain
Lachesis_group0__13_contigs__l.g135	158.2412	30.08145	2.40121	2.80E-05	0.000707	1794	- && - && PF00270:DEAD/DEAH box helicase
Lachesis_group0__13_contigs__l.g10160	114.7667	21.76865	2.39781	0.000175	0.002639	390	- && B8PBQ6.1 RecName: Full=Transcription activator of gluconeogenesis ERT1 && -
Lachesis_group0__13_contigs__l.g7510	1473.434	280.1696	2.395218	3.62E-06	0.000164	1689	- && O94365.1 RecName: Full=U3 small nucleolar RNA-associated protein 15; Short=U3 snoRNA-associated protein 15; AltName: Full=U3 protein 15 required for transcription && PF09384:UTP15 C terminal
Lachesis_group0__13_contigs__l.g5623	1420.698	270.7019	2.392009	5.73E-05	0.001192	3048	- && Q01371.2 RecName: Full=White collar 1 protein; Short=WC1 && PF08447:PAS fold|PF13426:PAS domain

Lachesis_group0__13_contigs__l.g5950	125.491	23.81652	2.389426	0.005834	0.032219	2322	- && Q6BQU8.2 RecName: Full=Nitrogen permease regulator 3; AltName: Full=Required for meiotic nuclear division protein 11; Flags: Precursor && PF03666:Nitrogen Permease regulator of amino acid transport activity 3
Lachesis_group0__13_contigs__l.g2215	7719.644	1475.059	2.387854	0.000261	0.003498	1509	- && Q70LM3.1 RecName: Full=Manganese peroxidase 2; Short=MnP2; AltName: Full=Manganese peroxidase isozyme 2; Flags: Precursor && PF11895:Domain of unknown function (DUF3415)|PF00141:Peroxidase
Lachesis_group0__13_contigs__l.g6436	744.0688	142.2982	2.386372	0.000131	0.002187	849	- && Q0VD01.1 RecName: Full=U3 small nucleolar ribonucleoprotein protein IMP4; Short=U3 snoRNP protein IMP4 && PF04427:Brix domain
Lachesis_group0__13_contigs__l.g2446	140.6135	26.80226	2.382914	0.000544	0.00594	1794	- && Q9UUA2.1 RecName: Full=ATP-dependent DNA helicase pfh1; AltName: Full=DNA repair and recombination helicase pfh1; AltName: Full=PIF1 helicase homolog; AltName: Full=RRM3/PIF1 homolog 1; Flags: Precursor && PF05970:PIF1-like helicase
Lachesis_group0__13_contigs__l.g8820	1434.583	275.3593	2.381708	3.13E-06	0.000148	543	- && Q9D187.1 RecName: Full=Mitotic spindle-associated MMXD complex subunit MIP18; AltName: Full=Protein FAM96B && PF01883:Domain of unknown function DUF59
Lachesis_group0__13_contigs__l.g997	683.968	131.425	2.379132	5.95E-06	0.000238	1095	- && O14180.1 RecName: Full=Brix domain-containing protein C4F8.04 && PF04427:Brix domain
Lachesis_group0__13_contigs__l.g5328	89.17354	17.33261	2.368933	0.000135	0.00222	2970	- && Q10213.1 RecName: Full=ATP-dependent DNA helicase srs2 && PF13361:UvrD-like helicase

							C-terminal domain|PF00580:UvrD/REP helicase N-terminal domain
Lachesis_group0__13_contigs__l.g10027	402.3039	78.20682	2.364611	0.000526	0.005794	2718	- && P40377.1 RecName: Full=DNA replication licensing factor mcm2; AltName: Full=Cell division control protein 19; AltName: Full=Minichromosome maintenance protein 2 && PF12619:Mini-chromosome maintenance protein 2|PF00493:MCM2/3/5 family|PF14551:MCM N-terminal domain
Lachesis_group0__13_contigs__l.g8591	1093.397	213.2431	2.358716	1.19E-06	7.30E-05	2670	- && A5PJS6.1 RecName: Full=Ubiquitin carboxyl-terminal hydrolase 10; AltName: Full=Deubiquitinating enzyme 10; AltName: Full=Ubiquitin thioesterase 10; AltName: Full=Ubiquitin-specific-processing protease 10 && PF00443:Ubiquitin carboxyl-terminal hydrolase
Lachesis_group0__13_contigs__l.g8683	2870.885	560.2254	2.357569	1.30E-06	7.91E-05	759	- && P38523.1 RecName: Full=GrpE protein homolog, mitochondrial; Flags: Precursor && PF01025:GrpE
Lachesis_group0__13_contigs__l.g5212	6009.024	1175.952	2.353464	1.90E-06	0.000102	1251	- && O13982.1 RecName: Full=Uncharacterized WD repeat-containing protein C25H1.08c && PF00400:WD domain, G-beta repeat
Lachesis_group0__13_contigs__l.g6922	618.2401	122.0463	2.341875	2.55E-05	0.000668	2373	- && A8P7F7.1 RecName: Full=Nucleolar protein 9; AltName: Full=Pumilio domain-containing protein NOP9 && -
Lachesis_group0__13_contigs__l.g129	299.525	59.43487	2.337331	6.49E-06	0.000253	1779	- && P0CS53.1 RecName: Full=tRNA (guanine-N(7)-)-methyltransferase non-catalytic subunit TRM82; AltName: Full=Transfer RNA methyltransferase 82 && -

Lachesis_group0__13_contigs__l.g10384	438.6577	86.9718	2.333636	0.000182	0.002706	1980	- && Q99741.1 RecName: Full=Cell division control protein 6 homolog; AltName: Full=CDC6-related protein; AltName: Full=Cdc18-related protein; Short=HsCdc18; AltName: Full=p62(cdc6); Short=HsCDC6 && PF13401:AAA domain
Lachesis_group0__13_contigs__l.g545	4868.308	968.9872	2.329195	5.50E-06	0.000227	1437	- && P54872.2 RecName: Full=Hydroxymethylglutaryl-CoA synthase A; Short=HMG-CoA synthase A; AltName: Full=3-hydroxy-3-methylglutaryl coenzyme A synthase A && PF01154:Hydroxymethylglutaryl-coenzyme A synthase N terminal|PF08540:Hydroxymethylglutaryl-coenzyme A synthase C terminal
Lachesis_group0__13_contigs__l.g9563	374.4793	74.56514	2.328994	0.000276	0.003668	837	- && - && PF08208:DNA-directed RNA polymerase I subunit RPA34.5
Lachesis_group0__13_contigs__l.g11087	339.965	68.16407	2.319975	2.81E-05	0.000707	891	- && - && PF07890:Rrp15p
Lachesis_group0__13_contigs__l.g5539	448.5628	89.85741	2.318673	5.89E-05	0.001215	594	- && Q4X0T4.1 RecName: Full=Required for respiratory growth protein 9, mitochondrial; Flags: Precursor >B0XSW5.1 RecName: Full=Required for respiratory growth protein 9, mitochondrial; Flags: Precursor && PF06413:Neugrin
Lachesis_group0__13_contigs__l.g6690	535.4862	107.6062	2.314964	2.91E-06	0.000141	1764	- && P94547.1 RecName: Full=Long-chain-fatty-acid--CoA ligase; AltName: Full=Long-chain acyl-CoA synthetase && PF13193:AMP-binding enzyme C-terminal domain|PF00501:AMP-binding enzyme
Lachesis_group0__13_contigs__l.g4690	730.5487	147.6399	2.308155	1.31E-05	0.000411	981	- && - && PF01738:Dienelactone hydrolase family

Lachesis_group0__13_contigs__l.g7107	77.75996	15.78456	2.307258	0.000788	0.007768	1170	- && Q29RY4.1 RecName: Full=Zinc phosphodiesterase ELAC protein 1; AltName: Full=ElaC homolog protein 1; AltName: Full=Ribonuclease Z 1; Short=RNase Z 1; AltName: Full=tRNA 3 endonuclease 1; AltName: Full=tRNase Z 1 && PF12706:Beta-lactamase superfamily domain
Lachesis_group0__13_contigs__l.g27	176.318	35.48353	2.307133	4.52E-05	0.001	918	- && - && PF06221:Putative zinc finger motif, C2HC5-type
Lachesis_group0__13_contigs__l.g4741	89.49948	18.04744	2.305977	0.000107	0.001904	1152	- && - && PF00326:Prolyl oligopeptidase family|PF07859:alpha/beta hydrolase fold
Lachesis_group0__13_contigs__l.g5164	770.7655	156.7492	2.298682	3.23E-06	0.000151	627	- && Q09191.1 RecName: Full=DNA-directed RNA polymerases I, II, and III subunit RPABC1; Short=RNA polymerases I, II, and III subunit ABC1; AltName: Full=RPC24B && PF01191:RNA polymerase Rpb5, C-terminal domain|PF03871:RNA polymerase Rpb5, N-terminal domain
Lachesis_group0__13_contigs__l.g8685	842.1214	171.4984	2.296853	2.86E-07	2.61E-05	1188	- && - && PF13324:Grap2 and cyclin-D-interacting
Lachesis_group0__13_contigs__l.g11544	1650.804	336.465	2.295409	2.60E-05	0.000673	3324	- && O13282.1 RecName: Full=Transcription initiation factor TFIID subunit 5; AltName: Full=Transcription initiation factor TFIID 72 kDa subunit; Short=TAFII-72 && PF00400:WD domain, G-beta repeat|PF04494:WD40 associated region in TFIID subunit, NTD2 domain
Lachesis_group0__13_contigs__l.g2416	621.3865	126.8127	2.294625	4.00E-05	0.000923	3228	- && Q9P7S5.1 RecName: Full=Probable ubiquitin carboxyl-terminal hydrolase 7; AltName: Full=Deubiquitinating enzyme 7; AltName:

							Full=Ubiquitin thioesterase 7; AltName: Full=Ubiquitin-specific-processing protease 7 && PF00443:Ubiquitin carboxyl-terminal hydrolase
Lachesis_group0__13_contigs__l.g554	357.9818	73.02876	2.293832	2.23E-05	0.000604	2505	- && Q9Y7R3.1 RecName: Full=Condensin complex subunit 2; AltName: Full=Barren homolog; AltName: Full=CAPH homolog; AltName: Full=p105 && PF05786:Condensin complex subunit 2
Lachesis_group0__13_contigs__l.g11370	203.3816	41.54738	2.293007	4.52E-05	0.001	1788	- && O74397.1 RecName: Full=Asparagine synthetase domain-containing protein C4F6.11c && PF00733:Asparagine synthase
Lachesis_group0__13_contigs__l.g5967	13356.3	2731.788	2.289669	0.00108	0.009782	474	- && - && PF01185:Fungal hydrophobin
Lachesis_group0__13_contigs__l.g676	128.0679	26.19261	2.287457	0.000308	0.003931	2580	- && - && PF11559:Afadin- and alpha -actinin-Binding
Lachesis_group0__13_contigs__l.g8472	2967.258	608.61	2.285576	1.11E-05	0.000368	1500	- && Q51342.3 RecName: Full=Amidophosphoribosyltransferase; Short=ATase; AltName: Full=Glutamine phosphoribosylpyrophosphate amidotransferase; Short=GPATase && PF00156:Phosphoribosyl transferase domain|PF13522:Glutamine amidotransferase domain
Lachesis_group0__13_contigs__l.g8217	39.03397	8.058027	2.284147	0.002986	0.019947	849	- && - && PF06544:Protein of unknown function (DUF1115)
Lachesis_group0__13_contigs__l.g10512	7226.211	1484.1	2.283819	8.02E-07	5.69E-05	3609	- && Q11119.2 RecName: Full=Ubiquitin carboxyl-terminal hydrolase 14; AltName: Full=Deubiquitinating enzyme 14; AltName: Full=UBA domain-containing protein 2; AltName: Full=Ubiquitin thioesterase 14; AltName:

							Full=Ubiquitin-specific-processing protease 14 && PF00956:Nucleosome assembly protein (NAP)|PF00627:UBA/TS-N domain|PF02148:Zn-finger in ubiquitin-hydrolases and other protein|PF00443:Ubiquitin carboxyl-terminal hydrolase
Lachesis_group0__13_contigs__l.g10381	269.5999	55.66886	2.278656	1.22E-05	0.000393	1665	- && - && PF07535:DBF zinc finger|PF08630:Dfp1/Him1, central region
Lachesis_group0__13_contigs__l.g9169	419.7513	87.16966	2.271859	7.99E-05	0.001546	1494	- && - && PF00226:DnaJ domain
Lachesis_group0__13_contigs__l.g6442	1805.489	374.4955	2.269481	3.57E-05	0.000835	1218	- && Q9C167.2 RecName: Full=Ribonucleoside-diphosphate reductase small chain; AltName: Full=Ribonucleotide reductase small subunit && PF00268:Ribonucleotide reductase, small chain
Lachesis_group0__13_contigs__l.g476	103.9026	21.51779	2.267942	0.00033	0.004137	1641	- && - && PF12937:F-box-like
Lachesis_group0__13_contigs__l.g7294	132.9519	27.46804	2.265998	0.001022	0.009423	4167	- && - && PF12770:CHAT domain
Lachesis_group0__13_contigs__l.g11551	1602.671	333.7052	2.263972	6.17E-06	0.000243	1401	- && P40510.1 RecName: Full=D-3-phosphoglycerate dehydrogenase 2; Short=3-PGDH 2 && PF00389:D-isomer specific 2-hydroxyacid dehydrogenase, catalytic domain|PF02826:D-isomer specific 2-hydroxyacid dehydrogenase, NAD binding domain
Lachesis_group0__13_contigs__l.g4274	203.3295	42.24485	2.259848	0.003578	0.022774	3114	- && Q9W689.1 RecName: Full=Ataxin-3; AltName: Full=Machado-Joseph disease protein 1 homolog && PF02099:Josephin|PF03595:Voltage-dependent anion channel
Lachesis_group0__13_contigs__l.g2229	1388.397	290.3733	2.257931	1.74E-05	0.000515	1362	- && Q7ZYQ6.1 RecName: Full=DDB1- and CUL4-associated factor 13; AltName: Full=WD repeat and SOF domain-containing protein 1 &&

							PF04158:Sof1-like domain|PF00400:WD domain, G-beta repeat
Lachesis_group0__13_contigs__l.g11550	1295.34	271.3919	2.255482	1.13E-06	7.12E-05	1680	- && O94303.3 RecName: Full=Imidazole glycerol phosphate synthase hisHF; Short=IGP synthase; Short=IGPS; Short=ImGP synthase; Includes: RecName: Full=Glutamine amidotransferase; Includes: RecName: Full=Cyclase && PF00117:Glutamine amidotransferase class-I|PF00977:Histidine biosynthesis protein
Lachesis_group0__13_contigs__l.g9241	419.8764	88.06464	2.25367	9.79E-05	0.001778	2349	- && O75001.1 RecName: Full=DNA replication licensing factor mcm7; AltName: Full=Minichromosome maintenance protein 7 && PF00493:MCM2/3/5 family|PF14551:MCM N-terminal domain
Lachesis_group0__13_contigs__l.g8476	758.7456	159.4427	2.251466	2.00E-05	0.000561	975	- && Q08235.2 RecName: Full=Ribosome biogenesis protein BRX1 && PF04427:Brix domain
Lachesis_group0__13_contigs__l.g6400	236.2583	49.67749	2.248577	5.12E-06	0.000215	990	- && P0CL97.1 RecName: Full=Pre-rRNA-processing protein ESF2; AltName: Full=18S rRNA factor 2 >P0CL96.1 RecName: Full=Pre-rRNA-processing protein ESF2; AltName: Full=18S rRNA factor 2 && -
Lachesis_group0__13_contigs__l.g6398	1085.83	229.0041	2.24639	5.29E-06	0.000221	4278	- && P87323.1 RecName: Full=Response regulator mcs4; AltName: Full=Mitotic catastrophe suppressor 4 && PF00072:Response regulator receiver domain
Lachesis_group0__13_contigs__l.g7105	56.90866	11.96281	2.24443	0.00788	0.04012	513	- && - && PF04430:Protein of unknown function (DUF498/DUF598)

Lachesis_group0__13_contigs__l.g5596	313.5047	66.16852	2.243503	9.39E-05	0.001741	1395	- && O13632.1 RecName: Full=Tyrosine-protein phosphatase yvh1; Short=PTPase yvh1 && PF00782:Dual specificity phosphatase, catalytic domain
Lachesis_group0__13_contigs__l.g2141	751.7058	159.1094	2.241823	1.93E-05	0.00055	1203	- && - && PF15459:60S ribosome biogenesis protein Rrp14|PF04935:Surfeit locus protein 6
Lachesis_group0__13_contigs__l.g4542	226.4742	47.98693	2.240872	0.001123	0.010042	1317	- && O59790.2 RecName: Full=Serine/threonine-protein kinase ark1; AltName: Full=Aurora-related kinase 1 && PF00069:Protein kinase domain
Lachesis_group0__13_contigs__l.g4540	225.7752	48.1908	2.233141	0.000415	0.004886	921	- && Q6ME51.1 RecName: Full=50S ribosomal protein L5 && PF00673:ribosomal L5P family C-terminus
Lachesis_group0__13_contigs__l.g11128	1598.96	340.5939	2.231193	2.52E-06	0.000126	4425	- && O74835.1 RecName: Full=rRNA biogenesis protein rrp5; AltName: Full=Ribosomal RNA-processing protein 5; AltName: Full=U3 small nucleolar RNA-associated protein rrp5; Short=U3 snoRNA-associated protein rrp5 && PF00575:S1 RNA binding domain
Lachesis_group0__13_contigs__l.g7938	886.8023	189.0762	2.230602	7.73E-05	0.001504	1764	- && O14360.1 RecName: Full=H/ACA ribonucleoprotein complex non-core subunit NAF1; AltName: Full=Nuclear assembly factor 1 && PF04410:Gar1/Naf1 RNA binding region
Lachesis_group0__13_contigs__l.g1212	395.6761	84.66623	2.225856	2.98E-05	0.000733	663	- && Q7S5W9.1 RecName: Full=Phosphatidyl-N-methylethanolamine N-methyltransferase; AltName: Full=Cholineless-2; AltName: Full=Phospholipid methyltransferase; Short=PLMT && PF04191:Phospholipid methyltransferase

Lachesis_group0__13_contigs__l.g3156	103.8233	22.02616	2.225193	0.001925	0.014704	1335	- && Q6NU95.1 RecName: Full=RNA polymerase II-associated protein 3 && PF13414:TPR repeat
Lachesis_group0__13_contigs__l.g9080	307.491	65.86469	2.221756	2.19E-05	0.000597	693	- && - && PF04930:FUN14 family
Lachesis_group0__13_contigs__l.g10727	744.1999	160.3011	2.21478	5.32E-06	0.000222	1383	- && P08465.2 RecName: Full=Homoserine O-acetyltransferase; AltName: Full=Homoserine O-trans-acetylase && PF00561:alpha/beta hydrolase fold
Lachesis_group0__13_contigs__l.g8181	320.7479	69.10593	2.214624	0.00037	0.004501	1275	- && P17898.3 RecName: Full=Cholinephosphotransferase 1; AltName: Full=Aminoalcohol phosphotransferase CPT1; AltName: Full=Diacylglycerol cholinephosphotransferase 1; AltName: Full=Sn-1,2-diacylglycerol cholinephosphotransferase; Short=CHOPT && PF01066:CDP-alcohol phosphatidyltransferase
Lachesis_group0__13_contigs__l.g7939	142.5364	30.88308	2.214352	0.000265	0.003555	1812	- && - && PF15275:PEHE domain
Lachesis_group0__13_contigs__l.g5797	217.1785	46.96549	2.212424	0.000301	0.003877	1071	- && - && PF06966:Protein of unknown function (DUF1295)
Lachesis_group0__13_contigs__l.g11214	258.7309	56.0342	2.211286	0.000112	0.00195	1137	- && P42023.1 RecName: Full=Actin-2; AltName: Full=Actin II; AltName: Full=Centractin-like protein [Pneumocystis carinii] && PF00022:Actin
Lachesis_group0__13_contigs__l.g2197	2418.567	524.3511	2.205521	1.74E-06	9.58E-05	2337	- && - && PF13813:Membrane bound O-acyl transferase family
Lachesis_group0__13_contigs__l.g10100	5370.293	1168.343	2.200472	1.75E-07	1.90E-05	1488	- && Q9LK73.1 RecName: Full=UDP-glycosyltransferase 88A1 && PF00201:UDP-glucoronosyl and UDP-glucosyl transferase
Lachesis_group0__13_contigs__l.g10132	1719.248	374.7093	2.198579	2.61E-05	0.000674	2502	- && P0CQ95.1 RecName: Full=ATP-dependent RNA helicase DBP7 >P0CQ94.1 RecName:

<table>
<tr><td></td><td></td><td></td><td></td><td></td><td></td><td></td><td>Full=ATP-dependent RNA helicase DBP7 && PF13959:Domain of unknown function (DUF4217)|PF00270:DEAD/DEAH box helicase|PF00271:Helicase conserved C-terminal domain</td></tr>
<tr><td>Lachesis_group0__13_contigs__l.g9594</td><td>768.8381</td><td>167.5451</td><td>2.198322</td><td>0.001869</td><td>0.014411</td><td>1008</td><td>- && Q5XGR8.1 RecName: Full=Endoribonuclease LACTB2; AltName: Full=Beta-lactamase-like protein 2 && PF00753:Metallo-beta-lactamase superfamily</td></tr>
<tr><td>Lachesis_group0__13_contigs__l.g11075</td><td>4872.271</td><td>1062.577</td><td>2.197202</td><td>2.03E-06</td><td>0.000107</td><td>1599</td><td>- && G5EB89.1 RecName: Full=Importin subunit alpha; AltName: Full=Karyopherin alpha && PF16186:Atypical Arm repeat|PF01749:Importin beta binding domain|PF00514:Armadillo/beta-catenin-like repeat</td></tr>
<tr><td>Lachesis_group0__13_contigs__l.g11619</td><td>154.7944</td><td>34.13156</td><td>2.186548</td><td>0.001807</td><td>0.013998</td><td>2634</td><td>- && P52958.1 RecName: Full=Cutinase transcription factor 1 alpha && PF04082:Fungal specific transcription factor domain</td></tr>
<tr><td>Lachesis_group0__13_contigs__l.g10711</td><td>102.2928</td><td>22.70427</td><td>2.182104</td><td>0.000528</td><td>0.0058</td><td>750</td><td>- && P0CR67.1 RecName: Full=Probable kinetochore protein SPC25 >P0CR66.1 RecName: Full=Probable kinetochore protein SPC25 && PF08234:Chromosome segregation protein Spc25</td></tr>
<tr><td>Lachesis_group0__13_contigs__l.g975</td><td>762.1244</td><td>168.3173</td><td>2.179627</td><td>5.21E-07</td><td>4.22E-05</td><td>612</td><td>- && Q64520.2 RecName: Full=Guanylate kinase; AltName: Full=GMP kinase && PF00625:Guanylate kinase</td></tr>
<tr><td>Lachesis_group0__13_contigs__l.g6146</td><td>6168.782</td><td>1363.522</td><td>2.177811</td><td>2.18E-05</td><td>0.000596</td><td>1731</td><td>- && P38861.3 RecName: Full=60S ribosomal export protein NMD3; AltName: Full=Nonsense-mediated mRNA decay protein 3 && PF04981:NMD3 family</td></tr>
</table>

Lachesis_group0__13_contigs__l.g9957	4923.022	1088.222	2.177229	0.00057	0.006132	2034	- && O34355.1 RecName: Full=Putative amidohydrolase YtcJ && PF07969:Amidohydrolase family
Lachesis_group0__13_contigs__l.g11376	405.7006	89.84886	2.173863	0.001122	0.010042	1725	- && - && PF02194:PXA domain|PF08628:Sorting nexin C terminal
Lachesis_group0__13_contigs__l.g8819	3641.215	807.1017	2.173418	1.10E-05	0.000367	1038	- && A1D3T4.1 RecName: Full=Probable arabinogalactan endo-beta-1,4-galactanase A; AltName: Full=Endo-1,4-beta-galactanase A; Short=Galactanase A; Flags: Precursor && PF07745:Glycosyl hydrolase family 53
Lachesis_group0__13_contigs__l.g5006	123.1404	27.23914	2.171116	5.76E-05	0.001195	825	- && - && PF01138:3' exoribonuclease family, domain 1
Lachesis_group0__13_contigs__l.g11297	1407.155	313.9018	2.164503	3.11E-05	0.000757	729	- && Q4P4X6.2 RecName: Full=Eukaryotic translation initiation factor 3 subunit K; Short=eIF3k; AltName: Full=eIF-3 p25 && PF10075:CSN8/PSMD8/EIF3K family
Lachesis_group0__13_contigs__l.g8303	463.9817	103.7458	2.164027	2.82E-05	0.000707	3084	- && P87137.1 RecName: Full=Uncharacterized protein C57A7.06 && PF04615:Utp14 protein
Lachesis_group0__13_contigs__l.g4172	243.4452	54.57197	2.159314	5.12E-05	0.001087	1209	- && Q8XJL8.1 RecName: Full=Probable serine/threonine-protein kinase CPE1738 && PF00069:Protein kinase domain
Lachesis_group0__13_contigs__l.g6808	357.6911	80.26497	2.158721	4.18E-05	0.000948	1047	- && - && PF17104:Domain of unknown function (DUF5102)
Lachesis_group0__13_contigs__l.g4599	138.1183	30.9912	2.157132	0.000385	0.004651	1476	- && P38121.1 RecName: Full=DNA polymerase alpha subunit B; AltName: Full=DNA polymerase I subunit B; AltName: Full=DNA polymerase alpha:primase complex p86 subunit; Short=Pol alpha-primase complex p86 subunit; AltName:

							Full=DNA polymerase-primase complex p74 subunit && PF04042:DNA polymerase alpha/epsilon subunit B|PF08418:DNA polymerase alpha subunit B N-terminal
Lachesis_group0__13_contigs__l.g1011	1214.087	272.4091	2.156543	2.11E-05	0.000583	3585	- && O94372.1 RecName: Full=Uncharacterized protein C1604.06c && PF03914:CBF/Mak21 family
Lachesis_group0__13_contigs__l.g2684	278.5482	62.68946	2.153278	0.000978	0.009115	1461	- && - && PF01612:3'-5' exonuclease
Lachesis_group0__13_contigs__l.g6635	1202.599	270.3676	2.15321	5.60E-06	0.000228	2850	- && Q9LYG9.1 RecName: Full=Mechanosensitive ion channel protein 10; AltName: Full=Mechanosensitive channel of small conductance-like 10; AltName: Full=MscS-Like protein 10; Short=AtMSL10 && PF00924:Mechanosensitive ion channel
Lachesis_group0__13_contigs__l.g4718	90.89977	20.43317	2.152078	0.001281	0.010975	1413	- && Q54DL6.1 RecName: Full=SET and MYND domain-containing protein DDB_G0292140 && PF01753:MYND finger|PF00856:SET domain
Lachesis_group0__13_contigs__l.g7426	434.237	97.86888	2.151635	0.00018	0.002681	1203	- && - && PF12923:Ribosomal RNA-processing protein 7 (RRP7)
Lachesis_group0__13_contigs__l.g6208	881.9539	198.6466	2.150659	9.73E-06	0.000335	1023	- && A8P7Y3.1 RecName: Full=S-methyl-5'-thioadenosine phosphorylase; AltName: Full=5'-methylthioadenosine phosphorylase; Short=MTA phosphorylase; Short=MTAP; Short=MTAPase && PF01048:Phosphorylase superfamily
Lachesis_group0__13_contigs__l.g8950	53.77453	12.33206	2.144676	0.007727	0.039635	711	- && Q503L4.2 RecName: Full=Isoamyl acetate-hydrolyzing esterase 1 homolog && PF00657:GDSL-like Lipase/Acylhydrolase
Lachesis_group0__13_contigs__l.g10881	765.1999	173.0625	2.144298	2.99E-05	0.000733	1215	- && Q9HE11.2 RecName: Full=U3 small nucleolar RNA-associated protein 5; Short=U3 snoRNA-

							associated protein 5; AltName: Full=U3 protein 5 required for transcription && PF04003:Dip2/Utp12 Family
Lachesis_group0__13_contigs__l.g9807	1208.244	274.2723	2.139899	4.19E-05	0.000949	1260	- && - && PF14976:FAM72 protein
Lachesis_group0__13_contigs__l.g5790	400.6897	90.81522	2.138745	0.000459	0.005278	1764	- && O13345.1 RecName: Full=O-methylsterigmatocystin oxidoreductase; Short=OMST oxidoreductase; AltName: Full=Aflatoxin B synthase; AltName: Full=Aflatoxin biosynthesis protein Q; AltName: Full=Cytochrome P450 64 && PF00067:Cytochrome P450
Lachesis_group0__13_contigs__l.g10960	1196.742	272.3823	2.135711	0.002065	0.015461	1830	- && A1CFL2.1 RecName: Full=Dehydrogenase patE; AltName: Full=Patulin synthesis protein E; Flags: Precursor && PF00732:GMC oxidoreductase|PF05199:GMC oxidoreductase
Lachesis_group0__13_contigs__l.g11136	1422.771	324.0445	2.134713	7.57E-06	0.000285	3372	- && Q10190.1 RecName: Full=Large subunit GTPase 1 && PF01926:50S ribosome-binding GTPase|PF00150:Cellulase (glycosyl hydrolase family 5)|PF00734:Fungal cellulose binding domain
Lachesis_group0__13_contigs__l.g11301	1754.935	400.964	2.130549	2.74E-05	0.000696	1869	- && A8N1X3.1 RecName: Full=Pescadillo homolog; AltName: Full=Nucleolar protein 7 homolog && PF16589:BRCT domain, a BRCA1 C-terminus domain|PF06732:Pescadillo N-terminus
Lachesis_group0__13_contigs__l.g9064	683.2468	156.5292	2.126978	1.80E-05	0.000528	1929	- && Q4P9E5.2 RecName: Full=ATP-dependent rRNA helicase SPB4 && PF00270:DEAD/DEAH box helicase|PF00271:Helicase conserved C-

							terminal domain|PF13959:Domain of unknown function (DUF4217)
Lachesis_group0__13_contigs__l.g10677	2601.015	595.8889	2.126377	1.20E-05	0.00039	1863	- && P0CQ78.1 RecName: Full=ATP-dependent RNA helicase DBP3 && PF00270:DEAD/DEAH box helicase|PF00271:Helicase conserved C-terminal domain
Lachesis_group0__13_contigs__l.g7907	1906.692	437.3521	2.123474	0.000105	0.001875	1212	- && Q9SAJ7.2 RecName: Full=Metal tolerance protein 9; Short=AtMTP9 && PF01545:Cation efflux family|PF16916:Dimerisation domain of Zinc Transporter
Lachesis_group0__13_contigs__l.g8975	196.1272	44.94633	2.120618	0.000224	0.003166	876	- && Q9UT16.2 RecName: Full=Protein atp12, mitochondrial; Flags: Precursor && PF07542:ATP12 chaperone protein
Lachesis_group0__13_contigs__l.g6389	454.6752	104.633	2.120017	0.000746	0.007463	3042	- && Q8K2V6.1 RecName: Full=Importin-11; Short=Imp11; AltName: Full=Ran-binding protein 11; Short=RanBP11 && -
Lachesis_group0__13_contigs__l.g4736	1300.757	299.6291	2.118475	1.35E-05	0.000421	1344	- && Q99078.2 RecName: Full=Dual specificity protein kinase FUZ7 && PF00069:Protein kinase domain
Lachesis_group0__13_contigs__l.g6921	1164.815	269.9503	2.109674	3.47E-05	0.000818	1242	- && O60071.1 RecName: Full=Uncharacterized protein C13G1.09 && PF05291:Bystin
Lachesis_group0__13_contigs__l.g235	420.4578	97.63865	2.109061	0.000123	0.002081	2604	- && Q9DE25.1 RecName: Full=Forkhead box C1-A && PF00250:Forkhead domain
Lachesis_group0__13_contigs__l.g165	876.2315	203.7314	2.106537	6.59E-05	0.00133	3171	- && O36021.1 RecName: Full=Uncharacterized protein C4F10.09c && PF03914:CBF/Mak21 family
Lachesis_group0__13_contigs__l.g1565	1946.207	452.3483	2.105398	2.96E-05	0.000731	1563	- && Q9ZV56.1 RecName: Full=Choline-phosphate cytidylyltransferase 1; Short=AtCCT1; AltName: Full=CTP:phosphocholine cytidylyltransferase 1;

								AltName: Full=Phosphorylcholine transferase 1 && PF01467:Cytidylyltransferase-like
Lachesis_group0__13_contigs__l.g11358	234.8465	54.90889	2.101269		0.001054	0.009635	3282	- && O13921.2 RecName: Full=MutS protein homolog 1 && PF00488:MutS domain V|PF05192:MutS domain III|PF01624:MutS domain I|PF05188:MutS domain II
Lachesis_group0__13_contigs__l.g8808	103.4951	24.19433	2.0977		0.000822	0.008043	1119	- && Q09843.1 RecName: Full=Replication factor C subunit 2; Short=Replication factor C2; AltName: Full=Activator 1 41 kDa subunit && PF08542:Replication factor C C-terminal domain|PF00004:ATPase family associated with various cellular activities (AAA)
Lachesis_group0__13_contigs__l.g8469	428.9265	100.3831	2.092663		5.94E-06	0.000238	2790	- && Q9Y804.1 RecName: Full=Fanconi-associated nuclease 1 homolog && PF08774:VRR-NUC domain
Lachesis_group0__13_contigs__l.g8965	1263.726	296.875	2.09053		3.03E-05	0.000742	3585	- && O60094.2 RecName: Full=DNA polymerase V; Short=POL V && PF04931:DNA polymerase phi
Lachesis_group0__13_contigs__l.g6965	950.5941	223.6147	2.089093		0.000402	0.004791	3237	- && P33520.1 RecName: Full=Cell division cycle-related protein res1/sct1; AltName: Full=p72sct1 && PF13637:Ankyrin repeats (many copies)|PF13857:Ankyrin repeats (many copies)
Lachesis_group0__13_contigs__l.g7800	69.3134	16.23273	2.087036		0.00161	0.012793	864	- && - && PF06912:Protein of unknown function (DUF1275)
Lachesis_group0__13_contigs__l.g5314	345.3183	81.58318	2.085579		0.000116	0.002	2082	- && Q76PD3.1 RecName: Full=ATP-dependent RNA helicase dbp6 && PF00270:DEAD/DEAH box helicase|PF00271:Helicase conserved C-terminal domain

Lachesis_group0__13_contigs__l.g10436	223.6542	52.72826	2.085011	0.000146	0.002354	3405	- && Q9C107.1 RecName: Full=Minichromosome loss protein 1; AltName: Full=DNA polymerase alpha accessory factor Mcl1 && PF12341:Minichromosome loss protein, Mcl1, middle region
Lachesis_group0__13_contigs__l.g4302	1745.814	412.2585	2.082674	8.15E-05	0.001568	3756	- && Q12754.1 RecName: Full=Ribosomal RNA-processing protein 12 && PF08161:NUC173 domain
Lachesis_group0__13_contigs__l.g6658	1004.426	237.2776	2.082039	9.68E-06	0.000335	795	- && Q1MTR2.2 RecName: Full=RNA-binding protein with serine-rich domain 1 homolog && PF00076:RNA recognition motif. (a.k.a. RRM, RBD, or RNP domain)
Lachesis_group0__13_contigs__l.g10868	4875.021	1151.771	2.08154	1.37E-05	0.000426	1350	- && Q9USW3.1 RecName: Full=Probable glycosidase C21B10.07 && PF00722:Glycosyl hydrolases family 16
Lachesis_group0__13_contigs__l.g8802	732.4436	173.4616	2.080051	9.18E-05	0.00171	993	- && Q9Y6Z9.1 RecName: Full=Sorbose reductase sou1; AltName: Full=Sorbitol utilization protein sou1 && PF00106:short chain dehydrogenase
Lachesis_group0__13_contigs__l.g1293	1117.363	264.5697	2.079655	3.25E-05	0.000782	1245	- && Q92925.3 RecName: Full=SWI/SNF-related matrix-associated actin-dependent regulator of chromatin subfamily D member 2; AltName: Full=60 kDa BRG-1/Brm-associated factor subunit B; AltName: Full=BRG1-associated factor 60B; Short=BAF60B && PF02201:SWIB/MDM2 domain
Lachesis_group0__13_contigs__l.g5204	840.9052	199.522	2.076449	0.000134	0.002217	1143	- && Q1MTN8.2 RecName: Full=Probable assembly chaperone of rpl4 && PF14559:Tetratricopeptide repeat

Lachesis_group0__13_contigs__l.g2040	144.6974	34.37627	2.075676	0.000706	0.007166	1035	- && O94505.1 RecName: Full=3'(2'),5'-bisphosphate nucleotidase; AltName: Full=3'(2'),5-bisphosphonucleoside 3'(2')-phosphohydrolase; AltName: Full=DPNPase; AltName: Full=Halotolerance protein tol1; AltName: Full=Target of lithium protein 1 && PF00459:Inositol monophosphatase family
Lachesis_group0__13_contigs__l.g10811	5773.553	1370.222	2.075123	1.03E-05	0.000351	1239	- && P47176.1 RecName: Full=Branched-chain-amino-acid aminotransferase, cytosolic; Short=BCAT; AltName: Full=Protein TWT2 && PF01063:Amino-transferase class IV
Lachesis_group0__13_contigs__l.g4665	329.0092	77.99435	2.074201	3.24E-05	0.000781	5991	- && Q92355.1 RecName: Full=Helicase sen1 && PF12726:SEN1 N terminal|PF13087:AAA domain|PF13086:AAA domain
Lachesis_group0__13_contigs__l.g9128	271.196	64.55546	2.073344	0.000876	0.008412	1182	- && Q601X8.2 RecName: Full=Chaperone protein DnaJ && PF00226:DnaJ domain
Lachesis_group0__13_contigs__l.g7875	629.68	150.172	2.068572	8.39E-06	0.000302	1470	- && Q8C2P3.1 RecName: Full=tRNA-dihydrouridine(16/17) synthase [NAD(P)(+)]-like; AltName: Full=tRNA-dihydrouridine synthase 1-like && PF01207:Dihydrouridine synthase (Dus)
Lachesis_group0__13_contigs__l.g11609	114.177	27.31943	2.066756	0.001394	0.011589	1293	- && P0CO35.1 RecName: Full=Inositol-pentakisphosphate 2-kinase; AltName: Full=Inositol-1,3,4,5,6-pentakisphosphate 2-kinase; AltName: Full=Ins(1,3,4,5,6)P5 2-kinase; Short=InsP5 2-kinase >P0CO34.1 RecName: Full=Inositol-pentakisphosphate 2-kinase; AltName: Full=Inositol-1,3,4,5,6-pentakisphosphate 2-kinase; AltName:

							Full=Ins(1,3,4,5,6)P5 2-kinase; Short=InsP5 2-kinase && PF06090:Inositol-pentakisphosphate 2-kinase
Lachesis_group0__13_contigs__l.g4022	920.786	220.0157	2.065946	8.07E-05	0.001559	1518	- && - && PF02845:CUE domain
Lachesis_group0__13_contigs__l.g8669	543.9879	130.1503	2.065903	0.000253	0.003419	1170	- && - && PF10332:Protein of unknown function (DUF2418)
Lachesis_group0__13_contigs__l.g9116	122.4487	29.19767	2.064857	0.002421	0.017292	936	- && Q2KIB9.1 RecName: Full=Ribonuclease P protein subunit p29 && PF01868:Domain of unknown function UPF0086
Lachesis_group0__13_contigs__l.g3511	3969.591	949.6661	2.063859	5.61E-05	0.001169	6525	- && O93937.2 RecName: Full=Protein pyrABCN; Includes: RecName: Full=Glutamine-dependent carbamoyl-phosphate; Includes: RecName: Full=Aspartate carbamoyltransferase && PF00117:Glutamine amidotransferase class-I|PF02787:Carbamoyl-phosphate synthetase large chain, oligomerisation domain|PF00185:Aspartate/ornithine carbamoyltransferase, Asp/Orn binding domain|PF02729:Aspartate/ornithine carbamoyltransferase, carbamoyl-P binding domain|PF02786:Carbamoyl-phosphate synthase L chain, ATP binding domain|PF02142:MGS-like domain|PF00988:Carbamoyl-phosphate synthase small chain, CPSase domain
Lachesis_group0__13_contigs__l.g4768	18973.28	4538.546	2.063711	4.29E-06	0.00019	1335	- && P59668.1 RecName: Full=Delta(12) fatty acid desaturase; AltName: Full=Delta-12 fatty acid desaturase && PF00487:Fatty acid desaturase
Lachesis_group0__13_contigs__l.g362	282.0556	67.67681	2.059505	0.000223	0.003166	1620	- && Q9HFE6.1 RecName: Full=Uncharacterized RNA-binding protein P16F5.06 && -

Lachesis_group0__13_contigs__l.g6869	154.4526	37.32151	2.055345	0.000473	0.005385	978	- && Q7XA87.1 RecName: Full=Folate transporter 1, chloroplastic; Short=AtFOLT1 && PF00153:Mitochondrial carrier protein
Lachesis_group0__13_contigs__l.g978	474.2344	114.2454	2.054856	4.38E-05	0.000986	948	- && Q9UTE6.1 RecName: Full=Protein mak16 && PF01778:Ribosomal L28e protein family|PF04874:Mak16 protein C-terminal region
Lachesis_group0__13_contigs__l.g6672	295.8226	71.34398	2.054601	0.000143	0.002314	1632	- && Q3UFB2.2 RecName: Full=Box C/D snoRNA protein 1; AltName: Full=Zinc finger HIT domain-containing protein 6 && PF04438:HIT zinc finger
Lachesis_group0__13_contigs__l.g3848	836.191	201.5179	2.053504	1.26E-05	0.000399	3603	- && - && PF12937:F-box-like
Lachesis_group0__13_contigs__l.g5168	158.8586	38.30985	2.051735	0.000421	0.004933	1347	- && P20397.3 RecName: Full=Nucleolin; AltName: Full=Protein C23 && PF14259:RNA recognition motif (a.k.a. RRM, RBD, or RNP domain)|PF00076:RNA recognition motif. (a.k.a. RRM, RBD, or RNP domain)
Lachesis_group0__13_contigs__l.g983	703.1863	169.8307	2.051031	1.13E-05	0.000372	1497	- && O13724.1 RecName: Full=Zinc finger protein zpr1 && PF03367:ZPR1 zinc-finger domain
Lachesis_group0__13_contigs__l.g8180	529.0597	127.8777	2.050469	8.84E-05	0.001664	1734	- && O43011.2 RecName: Full=Histidine--tRNA ligase, mitochondrial; AltName: Full=Histidyl-tRNA synthetase; Short=HisRS; Flags: Precursor && PF03129:Anticodon binding domain|PF13393:Histidyl-tRNA synthetase
Lachesis_group0__13_contigs__l.g5723	2516.485	607.4962	2.050336	6.20E-06	0.000243	1374	- && B3EAE0.1 RecName: Full=8-amino-7-oxononanoate synthase; Short=AONS; AltName: Full=7-keto-8-amino-pelargonic acid synthase; Short=7-KAP synthase; Short=KAPA synthase; AltName: Full=8-amino-7-ketopelargonate

							synthase && PF00155:Aminotransferase class I and II
Lachesis_group0__13_contigs__l.g7191	2015.194	487.6358	2.047492	7.24E-05	0.001443	894	- && Q4PI88.1 RecName: Full=Eukaryotic translation initiation factor 3 subunit F; Short=eIF3f && PF01398:JAB1/Mov34/MPN/PAD-1 ubiquitin protease|PF13012:Maintenance of mitochondrial structure and function
Lachesis_group0__13_contigs__l.g5290	3152.928	763.035	2.04696	1.75E-05	0.000516	2961	- && Q01371.2 RecName: Full=White collar 1 protein; Short=WC1 && PF00320:GATA zinc finger
Lachesis_group0__13_contigs__l.g1956	627.5514	151.9528	2.046385	2.85E-05	0.000713	1587	- && Q7ZWS5.1 RecName: Full=Sorting and assembly machinery component 50 homolog A && PF01103:Surface antigen
Lachesis_group0__13_contigs__l.g10875	719.6512	174.3431	2.045682	4.84E-05	0.001045	1773	- && Q75AE1.1 RecName: Full=ATP-dependent RNA helicase ROK1 && PF00270:DEAD/DEAH box helicase|PF00271:Helicase conserved C-terminal domain
Lachesis_group0__13_contigs__l.g1191	207.1723	50.25725	2.044785	0.000234	0.003247	1887	- && - && PF12937:F-box-like
Lachesis_group0__13_contigs__l.g1731	2473.765	599.6254	2.044263	7.69E-05	0.001499	2442	- && O88833.2 RecName: Full=Cytochrome P450 4A10; AltName: Full=CYPIVA10; AltName: Full=Cytochrome P450-LA-omega 1; AltName: Full=Cytochrome P452; AltName: Full=Lauric acid omega-hydroxylase; AltName: Full=Long-chain fatty acid omega-monooxygenase && PF00067:Cytochrome P450
Lachesis_group0__13_contigs__l.g1459	84.54798	20.5428	2.041128	0.001433	0.011803	1134	- && O94564.1 RecName: Full=Zinc-type alcohol dehydrogenase-like protein C1773.06c && PF08240:Alcohol dehydrogenase GroES-like domain|PF00107:Zinc-binding dehydrogenase

Lachesis_group0__13_contigs__l.g7347	452.4862	110.0076	2.040023	0.000251	0.003402	957	- && Q96B26.1 RecName: Full=Exosome complex component RRP43; AltName: Full=Exosome component 8; AltName: Full=Opa-interacting protein 2; Short=OIP-2; AltName: Full=Ribosomal RNA-processing protein 43; AltName: Full=p9 && PF03725:3' exoribonuclease family, domain 2|PF01138:3' exoribonuclease family, domain 1
Lachesis_group0__13_contigs__l.g11030	106.1756	25.99735	2.039266	0.000936	0.008827	1248	- && Q9CX47.1 RecName: Full=DNA repair protein XRCC2; AltName: Full=X-ray repair cross-complementing protein 2 && -
Lachesis_group0__13_contigs__l.g3351	432.0371	105.3228	2.039135	0.000314	0.003984	1011	- && Q10205.1 RecName: Full=Exosome complex component rrp43; AltName: Full=Ribosomal RNA-processing protein 43 && PF01138:3' exoribonuclease family, domain 1
Lachesis_group0__13_contigs__l.g8999	109.8097	26.71458	2.038051	0.00093	0.008794	2754	- && Q6NU40.1 RecName: Full=Chromosome transmission fidelity protein 18 homolog && PF00004:ATPase family associated with various cellular activities (AAA)
Lachesis_group0__13_contigs__l.g8137	342.6569	83.4558	2.03803	4.41E-05	0.000989	1389	- && P0CS07.1 RecName: Full=tRNA (adenine(58)-N(1))-methyltransferase non-catalytic subunit TRM6; AltName: Full=tRNA(m1A58)-methyltransferase subunit TRM6; Short=tRNA(m1A58)MTase subunit TRM6 >P0CS06.1 RecName: Full=tRNA (adenine(58)-N(1))-methyltransferase non-catalytic subunit TRM6; AltName: Full=tRNA(m1A58)-methyltransferase subunit TRM6;

							Short=tRNA(m1A58)MTase subunit TRM6 && PF04189:Gcd10p family
Lachesis_group0__13_contigs__l.g1721	1141.268	279.4801	2.030079	0.000162	0.002523	1092	- && C3K630.1 RecName: Full=L-amino acid amidase && PF12697:Alpha/beta hydrolase family
Lachesis_group0__13_contigs__l.g9187	1158.631	285.1021	2.023781	0.000107	0.001905	528	- && Q759D1.1 RecName: Full=Ribosome biogenesis protein RLP24 && PF01246:Ribosomal protein L24e
Lachesis_group0__13_contigs__l.g4161	965.1519	237.5286	2.023666	5.98E-05	0.001231	1437	- && Q4P4G8.1 RecName: Full=NKAP family protein UM04995 && PF06047:Ras-induced vulval development antagonist
Lachesis_group0__13_contigs__l.g11308	408.7138	100.5977	2.022542	3.46E-05	0.000816	903	- && B8PL20.1 RecName: Full=Maintenance of mitochondrial morphology protein 1 && PF10296:Maintenance of mitochondrial morphology protein 1
Lachesis_group0__13_contigs__l.g9167	600.6376	148.2347	2.0196	0.000239	0.003289	732	- && Q9CY21.1 RecName: Full=Probable 18S rRNA (guanine-N(7))-methyltransferase; AltName: Full=Bud site selection protein 23 homolog; AltName: Full=Williams-Beuren syndrome chromosomal region 22 protein homolog && PF12589:Methyltransferase involved in Williams-Beuren syndrome
Lachesis_group0__13_contigs__l.g6932	51.13244	12.72654	2.018929	0.004313	0.026026	1344	- && - && PF07699:GCC2 and GCC3
Lachesis_group0__13_contigs__l.g9135	131.7357	32.44596	2.016812	0.001397	0.011602	825	- && B4NBL6.1 RecName: Full=Protein phosphatase PTC7 homolog fig; AltName: Full=Fos intronic gene protein && PF07228:Stage II sporulation protein E (SpoIIE)
Lachesis_group0__13_contigs__l.g3350	513.7788	127.1553	2.015498	0.000118	0.002029	666	- && O94285.1 RecName: Full=DNA-directed RNA polymerase III subunit rpc8; Short=RNA

							polymerase III subunit C8; AltName: Full=RNA polymerase III subunit C25 && PF03876:SHS2 domain found in N terminus of Rpb7p/Rpc25p/MJ0397|PF08292:RNA polymerase III subunit Rpc25
Lachesis_group0__13_contigs__l.g8445	455.4487	112.826	2.015397	0.000112	0.001957	1857	- && - && PF12937:F-box-like
Lachesis_group0__13_contigs__l.g5505	411.6273	102.2683	2.010562	7.29E-05	0.001446	2007	- && A7EZP8.1 RecName: Full=U3 small nucleolar RNA-associated protein 25; Short=U3 snoRNA-associated protein 25; AltName: Full=U three protein 25 && PF06862:Utp25, U3 small nucleolar RNA-associated SSU processome protein 25
Lachesis_group0__13_contigs__l.g5708	618.1106	153.6837	2.010399	0.000422	0.00494	2418	- && O14325.1 RecName: Full=Uncharacterized AAA domain-containing protein C16E9.10c && PF00004:ATPase family associated with various cellular activities (AAA)
Lachesis_group0__13_contigs__l.g11345	3269.464	812.7833	2.008445	0.000109	0.001925	1173	- && P38997.1 RecName: Full=Saccharopine dehydrogenase [NAD(+), L-lysine-forming]; Short=SDH; AltName: Full=Lysine--2-oxoglutarate reductase && PF05222:Alanine dehydrogenase/PNT, N-terminal domain
Lachesis_group0__13_contigs__l.g11312	159.9948	39.69047	2.00729	0.000139	0.002276	471	- && Q92346.2 RecName: Full=Uncharacterized protein C6G9.01c && PF08576:Eukaryotic protein of unknown function (DUF1764)
Lachesis_group0__13_contigs__l.g7164	211.2959	52.72102	2.004344	0.000556	0.006033	1656	- && Q8R1Q8.1 RecName: Full=Cytoplasmic dynein 1 light intermediate chain 1; AltName: Full=Dynein light chain A; Short=DLC-A; AltName: Full=Dynein light intermediate chain 1, cytosolic

							&& PF05783:Dynein light intermediate chain (DLIC)
Lachesis_group0__13_contigs__l.g4773	820.6118	204.7597	2.004319	0.00023	0.003207	1614	- && Q9ZWB3.1 RecName: Full=Casein kinase 1-like protein 9; AltName: Full=Dual specificity kinase 1; AltName: Full=Protein CASEIN KINASE I-LIKE 9; AltName: Full=Protein CASEIN KINASE I-LIKE 9 ALPHA; AltName: Full=Protein CASEIN KINASE I-LIKE 9 BETA && PF00069:Protein kinase domain
Lachesis_group0__13_contigs__l.g5532	1370.411	342.0641	2.002762	1.91E-05	0.000549	2313	- && P0CQ91.1 RecName: Full=ATP-dependent RNA helicase MAK5 && PF00270:DEAD/DEAH box helicase|PF00271:Helicase conserved C-terminal domain
Lachesis_group0__13_contigs__l.g7690	556.256	138.998	2.001905	0.000105	0.001883	1392	- && Q4P553.2 RecName: Full=Histone acetyltransferase type B subunit 2 && PF12265:Histone-binding protein RBBP4 or subunit C of CAF1 complex
Lachesis_group0__13_contigs__l.g4177	254.1168	63.53066	2.00127	6.74E-05	0.001355	1722	- && B7F9G7.1 RecName: Full=Heptahelical transmembrane protein ADIPOR1; AltName: Full=PAQR family protein ADIPOR1 && PF03006:Haemolysin-III related
Lachesis_group0__13_contigs__l.g6036	475.0291	119.1283	1.995778	7.55E-06	0.000285	3510	- && P28706.2 RecName: Full=DNA repair protein rad13 && PF00752:XPG N-terminal domain|PF00867:XPG I-region
Lachesis_group0__13_contigs__l.g6892	2940.923	738.4431	1.994151	0.000167	0.002563	2034	- && P40010.1 RecName: Full=Nuclear GTP-binding protein NUG1; AltName: Full=Nuclear GTPase 1 && PF01926:50S ribosome-binding GTPase|PF08701:GNL3L/Grn1 putative GTPase

Lachesis_group0__13_contigs__l.g4266	1490.213	375.132	1.99083	9.58E-05	0.001759	2304	- && Q9USL4.2 RecName: Full=Nucleoporin nup61; AltName: Full=Nuclear pore protein nup61 && PF00638:RanBP1 domain|PF08911:NUP50 (Nucleoporin 50 kDa)
Lachesis_group0__13_contigs__l.g11566	514.6342	129.5567	1.990326	0.00086	0.008306	1155	- && P53871.1 RecName: Full=Probable glutamine amidotransferase DUG3; AltName: Full=Deficient in utilization of glutathione protein 3; AltName: Full=GSH degradosomal complex subunit DUG3 && PF13230:Glutamine amidotransferases class-II
Lachesis_group0__13_contigs__l.g6517	527.4825	132.838	1.988908	0.000732	0.00736	540	- && Q6MVL2.1 RecName: Full=Deoxyuridine 5'-triphosphate nucleotidohydrolase; Short=dUTPase; AltName: Full=dUTP pyrophosphatase && PF00692:dUTPase
Lachesis_group0__13_contigs__l.g2759	1943.321	490.9477	1.98509	1.55E-05	0.000475	966	- && Q9UUI8.1 RecName: Full=Uncharacterized transporter C22F8.04 && PF03151:Triose-phosphate Transporter family
Lachesis_group0__13_contigs__l.g2089	750.0039	189.7177	1.98369	0.000131	0.002182	570	- && B0D4L0.1 RecName: Full=Signal peptidase complex catalytic subunit SEC11; AltName: Full=Signal peptidase I && PF00717:Peptidase S24-like
Lachesis_group0__13_contigs__l.g6008	51.0997	12.90826	1.979825	0.003387	0.021894	633	- && - && PF01185:Fungal hydrophobin
Lachesis_group0__13_contigs__l.g5385	531.8056	135.1487	1.977598	0.000285	0.003755	2628	- && P52948.4 RecName: Full=Nuclear pore complex protein Nup98-Nup96; Contains: RecName: Full=Nuclear pore complex protein Nup98; AltName: Full=98 kDa nucleoporin; AltName: Full=Nucleoporin Nup98; Short=Nup98; Contains: RecName: Full=Nuclear pore complex protein Nup96; AltName: Full=96 kDa nucleoporin;

							AltName: Full=Nucleoporin Nup96; Short=Nup96; Flags: Precursor && PF12110:Nuclear protein 96
Lachesis_group0__13_contigs__l.g8029	18649.12	4757.6	1.970845	0.0001	0.001812	375	- && P29429.2 RecName: Full=Thioredoxin; Short=Trx && PF00085:Thioredoxin
Lachesis_group0__13_contigs__l.g3003	468.4316	119.6174	1.970409	0.000253	0.003419	4332	- && O13816.3 RecName: Full=Cohesin subunit psc3; AltName: Full=SCC3 homolog && PF08514:STAG domain
Lachesis_group0__13_contigs__l.g4900	946.9913	242.0298	1.968944	4.64E-05	0.001016	1722	- && Q9P775.1 RecName: Full=Uncharacterized WD repeat-containing protein C17D11.16 && -
Lachesis_group0__13_contigs__l.g3442	2175.61	556.378	1.967666	2.72E-05	0.000692	2256	- && O42945.1 RecName: Full=Probable pre-mRNA-splicing factor ATP-dependent RNA helicase prp43 && PF07717:Oligonucleotide/oligosaccharide-binding (OB)-fold|PF00271:Helicase conserved C-terminal domain|PF00270:DEAD/DEAH box helicase|PF04408:Helicase associated domain (HA2)
Lachesis_group0__13_contigs__l.g6430	1534.548	392.6081	1.966097	0.000148	0.002374	2172	- && O43000.2 RecName: Full=Pantothenate transporter liz1 && -
Lachesis_group0__13_contigs__l.g3400	633.7788	162.4181	1.963891	4.06E-05	0.000929	1140	- && Q6FM05.1 RecName: Full=37S ribosomal protein S10, mitochondrial; AltName: Full=Mitochondrial ribosomal small subunit protein 10; Flags: Precursor && PF00338:Ribosomal protein S10p/S20e
Lachesis_group0__13_contigs__l.g3885	2387.435	612.0461	1.963789	0.000231	0.003211	1755	- && Q9UUE6.1 RecName: Full=Lysine--tRNA ligase, cytoplasmic; AltName: Full=Lysyl-tRNA synthetase; Short=LysRS && PF01336:OB-fold

							nucleic acid binding domain|PF00152:tRNA synthetases class II (D, K and N)
Lachesis_group0__13_contigs__l.g778	188.9749	48.65531	1.958877	7.44E-05	0.001463	1626	- && - && PF02636:Putative S-adenosyl-L-methionine-dependent methyltransferase
Lachesis_group0__13_contigs__l.g375	891.3307	229.4924	1.957647	0.000237	0.00327	1593	- && Q7ZXK9.1 RecName: Full=Notchless protein homolog 1 && PF08154:NLE (NUC135) domain|PF00400:WD domain, G-beta repeat
Lachesis_group0__13_contigs__l.g1360	3499.031	901.8792	1.956329	7.68E-05	0.001499	1701	- && P87058.1 RecName: Full=La protein homolog; AltName: Full=La autoantigen homolog; AltName: Full=La ribonucleoprotein && PF05383:La domain|PF00076:RNA recognition motif. (a.k.a. RRM, RBD, or RNP domain)
Lachesis_group0__13_contigs__l.g4098	3204.84	826.7561	1.954924	0.00176	0.013724	1161	- && Q7SDV9.1 RecName: Full=Cytochrome c peroxidase, mitochondrial; Short=CCP; Flags: Precursor && PF00141:Peroxidase
Lachesis_group0__13_contigs__l.g270	9776.803	2524.562	1.95343	0.0013	0.011073	3219	- && Q4WY82.2 RecName: Full=Linoleate 10R-lipoxygenase; AltName: Full=Cyclooxygenase-like fatty acid oxygenase; AltName: Full=Fatty acid oxygenase ppoC; AltName: Full=Linoleate 10R-dioxygenase; Short=10R-DOX; AltName: Full=Psi-producing oxygenase C; Short=AfPpoC && PF03098:Animal haem peroxidase
Lachesis_group0__13_contigs__l.g365	565.2541	146.2815	1.950275	2.30E-05	0.000615	1506	- && Q6TNS2.1 RecName: Full=p21-activated protein kinase-interacting protein 1-like; AltName: Full=PAK1-interacting protein 1-like && PF00400:WD domain, G-beta repeat

Lachesis_group0__13_contigs__l.g9571	15388.41	3987.383	1.94829	0.0002	0.002924	1854	- && - && PF04082:Fungal specific transcription factor domain|PF00172:Fungal Zn(2)-Cys(6) binuclear cluster domain
Lachesis_group0__13_contigs__l.g1479	195.8971	50.83032	1.948192	0.000809	0.007933	1263	- && O94725.1 RecName: Full=Protein-glutamate O-methyltransferase C1393.13 && PF01937:Protein of unknown function DUF89
Lachesis_group0__13_contigs__l.g8058	1912.325	496.7711	1.945251	0.000411	0.004847	2850	- && Q5U2W5.1 RecName: Full=Transducin beta-like protein 3 && PF08625:Utp13 specific WD40 associated domain
Lachesis_group0__13_contigs__l.g7479	392.9171	101.9844	1.943712	0.001525	0.012345	1509	- && - && PF02205:WH2 motif
Lachesis_group0__13_contigs__l.g1506	342.5267	89.32237	1.943253	0.000908	0.008631	1632	- && Q9BZE2.3 RecName: Full=tRNA pseudouridine(38/39) synthase; AltName: Full=tRNA pseudouridine synthase 3; AltName: Full=tRNA pseudouridylate synthase 3; AltName: Full=tRNA-uridine isomerase 3 && PF01416:tRNA pseudouridine synthase
Lachesis_group0__13_contigs__l.g4004	5082.865	1322.177	1.942696	3.41E-06	0.000158	663	- && O94255.2 RecName: Full=Carbonic anhydrase; AltName: Full=Carbonate dehydratase && PF00484:Carbonic anhydrase
Lachesis_group0__13_contigs__l.g6666	252.1063	65.51442	1.942517	0.000858	0.008295	1092	- && Q6FLQ5.1 RecName: Full=Altered inheritance of mitochondria protein 32 && PF06999:Sucrase/ferredoxin-like
Lachesis_group0__13_contigs__l.g1537	436.0405	113.5463	1.940364	0.000106	0.001894	2820	- && Q17RS7.2 RecName: Full=Flap endonuclease GEN homolog 1 && PF00867:XPG I-region
Lachesis_group0__13_contigs__l.g3894	7989.432	2087.018	1.936782	9.62E-05	0.001763	2352	- && O59838.2 RecName: Full=Heat shock protein homolog pss1 && PF00012:Hsp70 protein|PF10712:NAD-specific glutamate dehydrogenase

Lachesis_group0__13_contigs__l.g2705	3147.424	823.6473	1.934316	0.000225	0.003166	1137	- && Q8K0C9.1 RecName: Full=GDP-mannose 4,6 dehydratase; AltName: Full=GDP-D-mannose dehydratase; Short=GMD && PF16363:GDP-mannose 4,6 dehydratase
Lachesis_group0__13_contigs__l.g9220	373.7996	97.93983	1.934122	0.000115	0.002	2082	- && Q03188.2 RecName: Full=Centromere protein C; Short=CENP-C; AltName: Full=Centromere autoantigen C; AltName: Full=Centromere protein C 1; Short=CENP-C 1; AltName: Full=Interphase centromere complex protein 7 && PF11699:Mif2/CENP-C like
Lachesis_group0__13_contigs__l.g8419	58.49357	15.37037	1.933434	0.009219	0.044634	363	- && P0CM85.1 RecName: Full=Cytochrome c oxidase assembly protein COX16, mitochondrial; Flags: Precursor >P0CM84.1 RecName: Full=Cytochrome c oxidase assembly protein COX16, mitochondrial; Flags: Precursor && PF14138:Cytochrome c oxidase assembly protein COX16
Lachesis_group0__13_contigs__l.g4588	1199.962	314.3711	1.93241	9.55E-05	0.001758	2169	- && Q03319.2 RecName: Full=Probable ATP-dependent RNA helicase prh1 && PF00270:DEAD/DEAH box helicase|PF07717:Oligonucleotide/oligosaccharide-binding (OB)-fold|PF04408:Helicase associated domain (HA2)|PF00271:Helicase conserved C-terminal domain
Lachesis_group0__13_contigs__l.g7158	1960.626	514.0794	1.931861	0.000241	0.003296	1272	- && Q4PEV8.1 RecName: Full=Eukaryotic translation initiation factor 3 subunit M; Short=eIF3m && PF01399:PCI domain

Lachesis_group0__13_contigs__l.g3724	677.3961	177.6688	1.930742	2.40E-05	0.000636	1962	- && - && PF03235:Protein of unknown function DUF262
Lachesis_group0__13_contigs__l.g10350	168.6426	44.2514	1.929886	0.000222	0.003164	2184	- && - && PF12348:CLASP N terminal
Lachesis_group0__13_contigs__l.g9548	2128.42	558.6214	1.929319	0.000176	0.002648	990	- && Q5XGI0.1 RecName: Full=Ribose-phosphate pyrophosphokinase 2; AltName: Full=Phosphoribosyl pyrophosphate synthase II; Short=PRS-II && PF14572:Phosphoribosyl synthetase-associated domain|PF13793:N-terminal domain of ribose phosphate pyrophosphokinase
Lachesis_group0__13_contigs__l.g2646	1021.554	268.6908	1.926584	0.000784	0.007729	2985	- && O13671.2 RecName: Full=Importin-alpha re-exporter; AltName: Full=Cellular apoptosis susceptibility protein homolog && PF03378:CAS/CSE protein, C-terminus|PF08506:Cse1|PF03810:Importin-beta N-terminal domain
Lachesis_group0__13_contigs__l.g8421	152.4151	40.22344	1.925738	0.001475	0.012077	1836	- && - && PF12937:F-box-like
Lachesis_group0__13_contigs__l.g4167	130.7256	34.41881	1.925355	0.001154	0.01017	567	- && C1A349.1 RecName: Full=Peptidyl-tRNA hydrolase; Short=PTH && PF01195:Peptidyl-tRNA hydrolase
Lachesis_group0__13_contigs__l.g6809	2197.367	579.2583	1.924124	0.000167	0.00256	5298	- && P15398.2 RecName: Full=DNA-directed RNA polymerase I subunit rpa1; AltName: Full=DNA-directed RNA polymerase I 190 kDa polypeptide; AltName: Full=DNA-directed RNA polymerase I largest subunit && PF04998:RNA polymerase Rpb1, domain 5|PF04983:RNA polymerase Rpb1, domain 3|PF04997:RNA polymerase Rpb1, domain 1|PF00623:RNA polymerase Rpb1, domain 2|PF05000:RNA polymerase Rpb1, domain 4

Lachesis_group0__13_contigs__l.g7689	207.5478	54.6353	1.92367	0.000598	0.006327	954	- && O94480.1 RecName: Full=25S rRNA (uridine-N(3))-methyltransferase && PF10354:Domain of unknown function (DUF2431)
Lachesis_group0__13_contigs__l.g9910	4115.25	1085.478	1.922634	7.69E-06	0.000286	1863	- && - && PF04577:Protein of unknown function (DUF563)
Lachesis_group0__13_contigs__l.g11458	644.4433	170.3272	1.921373	0.001165	0.010252	2301	- && P41389.2 RecName: Full=DNA replication licensing factor mcm5; AltName: Full=Cell division control protein nda4; AltName: Full=Minichromosome maintenance protein 5 && PF14551:MCM N-terminal domain|PF00493:MCM2/3/5 family
Lachesis_group0__13_contigs__l.g6106	1378.221	364.0166	1.921059	0.00016	0.002503	741	- && P33281.1 RecName: Full=C-8 sterol isomerase; AltName: Full=Delta-8--delta-7 sterol isomerase && PF04622:ERG2 and Sigma1 receptor like protein
Lachesis_group0__13_contigs__l.g5996	1183.603	312.6466	1.920936	0.000117	0.002006	1068	- && O74978.1 RecName: Full=Uncharacterized RNA-binding protein C1827.05c && PF00076:RNA recognition motif. (a.k.a. RRM, RBD, or RNP domain)
Lachesis_group0__13_contigs__l.g1508	1349.056	356.7121	1.920243	0.000375	0.004552	2034	- && Q3SYU1.1 RecName: Full=Nucleolar complex protein 2 homolog; Short=Protein NOC2 homolog; AltName: Full=NOC2-like protein; AltName: Full=Novel INHAT repressor && PF03715:Noc2p family
Lachesis_group0__13_contigs__l.g2817	46.82519	12.40414	1.919434	0.004615	0.0273	2400	- && - && PF04082:Fungal specific transcription factor domain
Lachesis_group0__13_contigs__l.g7163	872.797	231.2386	1.916798	0.000247	0.003347	1977	- && Q2KIH4.1 RecName: Full=Ribosome biogenesis regulatory protein homolog &&

							PF04176:TIP41-like family|PF04939:Ribosome biogenesis regulatory protein (RRS1)
Lachesis_group0__13_contigs__l.g6033	2418.465	641.1302	1.915674	2.54E-05	0.000668	1203	- && Q6AYD3.1 RecName: Full=Proliferation-associated protein 2G4 && PF00557:Metallopeptidase family M24
Lachesis_group0__13_contigs__l.g11400	1565.495	415.0967	1.915616	0.0001	0.001813	1701	- && Q4P6N0.2 RecName: Full=ATP-dependent RNA helicase HAS1 && PF13959:Domain of unknown function (DUF4217)|PF00270:DEAD/DEAH box helicase|PF00271:Helicase conserved C-terminal domain
Lachesis_group0__13_contigs__l.g2180	1296.785	344.0339	1.915415	0.001536	0.012385	1635	- && O74341.1 RecName: Full=T-complex protein 1 subunit gamma; Short=TCP-1-gamma; AltName: Full=CCT-gamma && PF00118:TCP-1/cpn60 chaperonin family
Lachesis_group0__13_contigs__l.g10819	134.9394	35.7028	1.914726	0.001202	0.010456	1578	- && A8MLB5.1 RecName: Full=Glutamate--tRNA ligase; AltName: Full=Glutamyl-tRNA synthetase; Short=GluRS && PF00749:tRNA synthetases class I (E and Q), catalytic domain
Lachesis_group0__13_contigs__l.g7775	1132.005	300.3404	1.914199	0.001046	0.009583	1590	- && Q1JQD2.1 RecName: Full=Glutamate-rich WD repeat-containing protein 1 && PF12265:Histone-binding protein RBBP4 or subunit C of CAF1 complex
Lachesis_group0__13_contigs__l.g2412	1251.239	332.6954	1.911107	1.03E-05	0.00035	1833	- && P10815.1 RecName: Full=G2/mitotic-specific cyclin cdc13 && PF02984:Cyclin, C-terminal domain|PF00134:Cyclin, N-terminal domain

Lachesis_group0__13_contigs__l.g662	1028.211	273.612	1.910126	5.55E-05	0.001163	2802	- && P38127.1 RecName: Full=Mitochondrial carrier protein RIM2 && PF12937:F-box-like|PF00153:Mitochondrial carrier protein
Lachesis_group0__13_contigs__l.g1359	5742.069	1528.128	1.909958	0.001381	0.011516	2835	- && - && PF04082:Fungal specific transcription factor domain
Lachesis_group0__13_contigs__l.g178	320.3064	85.25988	1.909592	0.002288	0.01659	2280	- && P28739.3 RecName: Full=Kinesin-like protein klpA && PF00225:Kinesin motor domain
Lachesis_group0__13_contigs__l.g6623	295.5461	78.59065	1.908678	0.000165	0.002539	1038	- && - && PF10180:Uncharacterised conserved protein (DUF2373)
Lachesis_group0__13_contigs__l.g8805	572.9554	152.8091	1.906999	4.31E-05	0.000975	912	- && O13970.2 RecName: Full=37S ribosomal protein mrp4, mitochondrial; Flags: Precursor && PF00318:Ribosomal protein S2
Lachesis_group0__13_contigs__l.g7195	966.5949	258.0285	1.906264	0.001442	0.011839	2949	- && P49731.2 RecName: Full=DNA replication licensing factor mcm6; AltName: Full=Minichromosome maintenance protein 6 && PF00493:MCM2/3/5 family|PF14551:MCM N-terminal domain
Lachesis_group0__13_contigs__l.g4415	235.3338	62.86301	1.905985	0.000755	0.00751	1533	- && Q08220.1 RecName: Full=Glutathione synthetase; Short=GSH synthetase; Short=GSH-S; AltName: Full=Glutathione synthase && PF03917:Eukaryotic glutathione synthase, ATP binding domain|PF03199:Eukaryotic glutathione synthase
Lachesis_group0__13_contigs__l.g9413	1024.639	273.9467	1.90368	0.000601	0.006354	3297	- && Q4P2U5.1 RecName: Full=FACT complex subunit SPT16; AltName: Full=Facilitates chromatin transcription complex subunit SPT16 && PF08644:FACT complex subunit (SPT16/CDC68)|PF14826:FACT complex subunit

							SPT16 N-terminal lobe domain|PF00557:Metallopeptidase family M24|PF08512:Histone chaperone Rttp106-like
Lachesis_group0__13_contigs__l.g8957	454.2887	121.6208	1.90364	0.000225	0.003166	2202	- && O60070.1 RecName: Full=Set1 complex component ash2; Short=Set1C component ash2; AltName: Full=COMPASS component ash2; AltName: Full=Complex proteins associated with set1 protein ash2; AltName: Full=Lid2 complex component ash2; Short=Lid2C component ash2 && PF00622:SPRY domain
Lachesis_group0__13_contigs__l.g11152	85.87378	23.0876	1.900049	0.007301	0.037999	1710	- && - && PF12937:F-box-like|PF13414:TPR repeat
Lachesis_group0__13_contigs__l.g3516	2856.33	765.7704	1.899096	0.000403	0.004796	1794	- && O94300.1 RecName: Full=Putative xanthine/uracil permease C887.17 && PF00860:Permease family
Lachesis_group0__13_contigs__l.g5673	6931.286	1858.634	1.898896	0.000137	0.002257	1401	- && P34085.2 RecName: Full=Citrate synthase, mitochondrial; Flags: Precursor && PF00285:Citrate synthase
Lachesis_group0__13_contigs__l.g6402	2255.508	605.1364	1.898018	0.000151	0.002406	4827	- && Q09878.2 RecName: Full=Probable sulfite reductase [NADPH] flavoprotein component && PF00175:Oxidoreductase NAD-binding domain|PF00667:FAD binding domain|PF00759:Glycosyl hydrolase family 9
Lachesis_group0__13_contigs__l.g2096	372.5781	100.3518	1.894476	0.000299	0.003864	3864	- && - && PF00498:FHA domain
Lachesis_group0__13_contigs__l.g11288	239.7963	64.5166	1.89408	0.000309	0.003937	681	- && Q8VY97.1 RecName: Full=ER membrane protein complex subunit 7 homolog; Flags: Precursor && PF09430:Protein of unknown function (DUF2012)

Lachesis_group0__13_contigs__l.g10617	1324.318	357.8364	1.887616	2.96E-05	0.000731	1941	- && Q0CJV3.1 RecName: Full=Vacuolar protein sorting-associated protein 27 && PF01363:FYVE zinc finger|PF03127:GAT domain|PF00790:VHS domain
Lachesis_group0__13_contigs__l.g4241	1174.983	318.8632	1.880809	0.000415	0.004886	2073	- && Q09768.1 RecName: Full=Glutamate--cysteine ligase; AltName: Full=Gamma-ECS; Short=GCS; AltName: Full=Gamma-glutamylcysteine synthetase && PF03074:Glutamate-cysteine ligase
Lachesis_group0__13_contigs__l.g11078	1115.428	302.9334	1.880467	2.29E-05	0.000615	1563	- && Q9D119.1 RecName: Full=Protein phosphatase 1 regulatory subunit 27; AltName: Full=Dysferlin-interacting protein 1 && PF13857:Ankyrin repeats (many copies)|PF00887:Acyl CoA binding protein|PF14622:Ribonuclease-III-like
Lachesis_group0__13_contigs__l.g2127	4486.986	1218.891	1.880273	0.000156	0.002463	993	- && P47818.1 RecName: Full=Protein CCC1; AltName: Full=Cross-complementer of CSG1 protein 1 && PF01988:VIT family
Lachesis_group0__13_contigs__l.g1154	134.0972	36.41079	1.88019	0.000494	0.005547	1797	- && - && PF12937:F-box-like
Lachesis_group0__13_contigs__l.g5871	2717.579	739.3765	1.877793	9.75E-05	0.001778	999	- && P20654.1 RecName: Full=Serine/threonine-protein phosphatase PP1 && PF16891:Serine-threonine protein phosphatase N-terminal domain|PF00149:Calcineurin-like phosphoesterase
Lachesis_group0__13_contigs__l.g3566	547.9635	149.7278	1.874009	0.004237	0.025687	1176	- && P50125.2 RecName: Full=Homocysteine synthase; AltName: Full=O-acetylhomoserine sulfhydrylase; Short=OAH SHL; Short=OAH sulfhydrylase && PF01053:Cys/Met metabolism PLP-dependent enzyme

Lachesis_group0__13_contigs__l.g4645	638.7232	174.4356	1.872518	0.000229	0.003206	759	- && Q7S910.2 RecName: Full=54S ribosomal protein L4, mitochondrial; Flags: Precursor && PF06984:Mitochondrial 39-S ribosomal protein L47 (MRP-L47)
Lachesis_group0__13_contigs__l.g9340	322.0726	87.93638	1.870757	0.000314	0.003984	888	- && Q9Y7M5.2 RecName: Full=54S ribosomal protein L10, mitochondrial; Flags: Precursor && PF00828:Ribosomal protein L18e/L15
Lachesis_group0__13_contigs__l.g2013	221.3382	60.51738	1.869275	0.000811	0.007948	2442	- && - && PF07814:Wings apart-like protein regulation of heterochromatin
Lachesis_group0__13_contigs__l.g342	270.8359	74.18937	1.867962	0.000292	0.003794	2730	- && Q24K12.1 RecName: Full=G patch domain-containing protein 1 && PF07713:Protein of unknown function (DUF1604)|PF01585:G-patch domain
Lachesis_group0__13_contigs__l.g11036	858.0803	235.3609	1.866681	0.000414	0.004879	1863	- && - && PF10297:Minimal binding motif of Hap4 for binding to Hap2/3/5
Lachesis_group0__13_contigs__l.g1348	142.5953	39.2397	1.86393	0.000335	0.004181	282	- && P53733.1 RecName: Full=37S ribosomal protein S19, mitochondrial && PF00203:Ribosomal protein S19
Lachesis_group0__13_contigs__l.g3983	42.83879	11.74559	1.863441	0.010776	0.049881	606	- && P36088.1 RecName: Full=Free methionine-R-sulfoxide reductase; Short=fRMsr; AltName: Full=GAF domain-containing protein YKL069W && -
Lachesis_group0__13_contigs__l.g8645	2220.441	610.5067	1.863214	0.000184	0.002723	381	- && P0CQ53.1 RecName: Full=13 kDa ribonucleoprotein-associated protein >P0CQ52.1 RecName: Full=13 kDa ribonucleoprotein-associated protein && PF01248:Ribosomal protein L7Ae/L30e/S12e/Gadd45 family
Lachesis_group0__13_contigs__l.g6386	585.0568	161.0828	1.860515	9.34E-05	0.001734	2019	- && - && PF13041:PPR repeat family

Lachesis_group0__13_contigs__l.g675	224.3808	61.93208	1.860426	0.002535	0.017806	1080	- && O14242.2 RecName: Full=Putative pyridoxal kinase C6F6.11c && PF08543:Phosphomethylpyrimidine kinase
Lachesis_group0__13_contigs__l.g9117	159.0604	43.83257	1.859051	0.000678	0.006966	1122	- && O74627.1 RecName: Full=Cyclin pch1; AltName: Full=Pombe cyclin C homolog 1 && PF00134:Cyclin, N-terminal domain
Lachesis_group0__13_contigs__l.g10287	676.3667	186.5099	1.858733	0.000126	0.002118	1104	- && Q6FL25.1 RecName: Full=37S ribosomal protein S9, mitochondrial; Flags: Precursor && PF00380:Ribosomal protein S9/S16
Lachesis_group0__13_contigs__l.g6369	438.7747	121.0079	1.858644	0.000143	0.002314	1926	- && O13432.3 RecName: Full=Phenylalanine--tRNA ligase beta subunit; AltName: Full=Phenylalanyl-tRNA synthetase beta subunit; Short=PheRS && PF03484:tRNA synthetase B5 domain|PF03483:B3/4 domain
Lachesis_group0__13_contigs__l.g2166	13562.02	3742.721	1.857368	0.000215	0.003089	1437	- && Q12618.1 RecName: Full=Acyl-CoA desaturase; AltName: Full=Delta(9)-desaturase; Short=Delta-9 desaturase; AltName: Full=Fatty acid desaturase; AltName: Full=Stearoyl-CoA desaturase && PF00487:Fatty acid desaturase|PF00173:Cytochrome b5-like Heme/Steroid binding domain
Lachesis_group0__13_contigs__l.g10059	1288.587	355.6427	1.857145	0.000466	0.005323	1542	- && P33886.1 RecName: Full=Protein kinase wis1; AltName: Full=Protein kinase sty2 && PF00069:Protein kinase domain
Lachesis_group0__13_contigs__l.g10632	516.922	143.041	1.855319	0.000212	0.00306	1974	- && Q1ZXE8.1 RecName: Full=Signal recognition particle subunit SRP68; AltName: Full=Signal recognition particle 68 kDa protein homolog &&

							PF16969:RNA-binding signal recognition particle 68
Lachesis_group0__13_contigs__l.g11209	584.1246	161.7816	1.853855	0.000147	0.002357	2340	- && P0CQ93.1 RecName: Full=ATP-dependent RNA helicase DRS1 >P0CQ92.1 RecName: Full=ATP-dependent RNA helicase DRS1 && PF00270:DEAD/DEAH box helicase|PF00271:Helicase conserved C-terminal domain
Lachesis_group0__13_contigs__l.g5933	526.1263	145.8415	1.853174	0.000204	0.002963	1854	- && - && PF08202:Mis12-Mtw1 protein family
Lachesis_group0__13_contigs__l.g1963	597.6126	165.6374	1.851445	7.32E-05	0.001449	1452	- && - && PF04577:Protein of unknown function (DUF563)
Lachesis_group0__13_contigs__l.g8822	654.3406	181.4755	1.851011	0.000162	0.002523	1575	- && Q09692.1 RecName: Full=Tryptophan--tRNA ligase, cytoplasmic; AltName: Full=Tryptophanyl-tRNA synthetase; Short=TrpRS && PF00579:tRNA synthetases class I (W and Y)
Lachesis_group0__13_contigs__l.g3425	1083.88	300.8631	1.849284	0.000237	0.00327	1185	- && P36421.3 RecName: Full=Tyrosine--tRNA ligase, cytoplasmic; AltName: Full=Tyrosyl-tRNA synthetase; Short=TyrRS && PF00579:tRNA synthetases class I (W and Y)
Lachesis_group0__13_contigs__l.g262	973.6605	270.7176	1.847164	0.000294	0.003816	978	- && Q6C7A6.1 RecName: Full=Cytosolic Fe-S cluster assembly factor NBP35; AltName: Full=Nucleotide-binding protein 35 && PF10609:NUBPL iron-transfer P-loop NTPase
Lachesis_group0__13_contigs__l.g2415	260.7332	72.66147	1.84683	0.002421	0.017292	1263	- && P36605.1 RecName: Full=Histidinol-phosphate aminotransferase; AltName: Full=Imidazole acetol-phosphate transaminase && PF00155:Aminotransferase class I and II

Lachesis_group0__13_contigs__l.g8652	1144.867	318.9687	1.844621	0.000156	0.002463	2853	- && O14053.1 RecName: Full=U3 small nucleolar RNA-associated protein 21 homolog; Short=U3 snoRNA-associated protein 21 && PF04192:Utp21 specific WD40 associated putative domain
Lachesis_group0__13_contigs__l.g5334	2361.401	659.2841	1.840998	0.000138	0.002264	1833	- && G0SEV9.2 RecName: Full=Translation initiation factor RLI1; AltName: Full=ATP-binding cassette sub-family E member RLI1 && PF00037:4Fe-4S binding domain|PF00005:ABC transporter|PF04068:Possible Fer4-like domain in RNase L inhibitor, RLI|PF07673:Protein of unknown function (DUF1602)
Lachesis_group0__13_contigs__l.g6979	141.7028	39.43031	1.840962	0.001133	0.010067	3636	- && D3YWJ0.1 RecName: Full=Nuclear GTPase SLIP-GC; AltName: Full=Speckled-like pattern in the germinal center && PF00350:Dynamin family
Lachesis_group0__13_contigs__l.g2014	578.2933	161.9539	1.83831	0.001363	0.011409	2946	- && - && PF00653:Inhibitor of Apoptosis domain
Lachesis_group0__13_contigs__l.g5549	112.4542	31.49327	1.833005	0.002295	0.01663	807	- && - && PF10197:N-terminal domain of CBF1 interacting co-repressor CIR
Lachesis_group0__13_contigs__l.g6760	445.8485	125.5551	1.829113	0.000222	0.003164	786	- && Q9CQI7.1 RecName: Full=U2 small nuclear ribonucleoprotein B''; Short=U2 snRNP B'' && PF00076:RNA recognition motif. (a.k.a. RRM, RBD, or RNP domain)|PF13893:RNA recognition motif. (a.k.a. RRM, RBD, or RNP domain)
Lachesis_group0__13_contigs__l.g7901	2107.221	593.8808	1.827615	0.000116	0.002	3480	- && Q14562.1 RecName: Full=ATP-dependent RNA helicase DHX8; AltName: Full=DEAH box protein 8; AltName: Full=RNA helicase HRH1 && PF00271:Helicase conserved C-terminal domain|PF07717:Oligonucleotide/oligosaccharide-binding (OB)-fold|PF00575:S1 RNA binding

							domain|PF04408:Helicase associated domain (HA2)
Lachesis_group0__13_contigs__l.g10159	2408.977	678.7159	1.827583	1.86E-05	0.000543	1725	- && B0D0T8.1 RecName: Full=Transcription activator of gluconeogenesis ERT1 && -
Lachesis_group0__13_contigs__l.g8543	304.74	85.90708	1.826631	0.007111	0.037259	3525	- && P17120.2 RecName: Full=Kinesin-like protein bimC && PF00225:Kinesin motor domain
Lachesis_group0__13_contigs__l.g11530	2402.455	678.0553	1.825296	1.32E-05	0.000411	1911	- && A8N2M6.1 RecName: Full=Amino-acid acetyltransferase, mitochondrial; AltName: Full=Arginine-requiring protein 2; AltName: Full=Glutamate N-acetyltransferase; AltName: Full=N-acetylglutamate synthase; Short=AGS; Short=NAGS; Flags: Precursor && PF04768:NAT, N-acetyltransferase, of N-acetylglutamate synthase
Lachesis_group0__13_contigs__l.g8529	7832.235	2211.822	1.824325	0.000167	0.00256	1185	- && P48466.1 RecName: Full=S-adenosylmethionine synthase; Short=AdoMet synthase; AltName: Full=Ethionine resistance protein 1; AltName: Full=Methionine adenosyltransferase; Short=MAT && PF02773:S-adenosylmethionine synthetase, C-terminal domain|PF02772:S-adenosylmethionine synthetase, central domain|PF00438:S-adenosylmethionine synthetase, N-terminal domain
Lachesis_group0__13_contigs__l.g114	541.8554	153.0775	1.823116	2.99E-05	0.000733	1272	- && - && PF01694:Rhomboid family
Lachesis_group0__13_contigs__l.g8088	226.9297	64.02211	1.822807	0.00032	0.004041	1734	- && O94725.1 RecName: Full=Protein-glutamate O-methyltransferase C1393.13 && PF01937:Protein of unknown function DUF89

Lachesis_group0__13_contigs__l.g6920	1995.133	564.1367	1.822381	7.33E-05	0.001449	1299	- && Q6GMI9.2 RecName: Full=UDP-glucuronic acid decarboxylase 1; AltName: Full=UDP-glucuronate decarboxylase 1; Short=UXS-1 && PF16363:GDP-mannose 4,6 dehydratase
Lachesis_group0__13_contigs__l.g9051	2977.695	842.1539	1.822018	2.23E-05	0.000603	6465	- && Q9C102.1 RecName: Full=Putative glutamate synthase [NADPH]; AltName: Full=NADPH-GOGAT && PF04898:Glutamate synthase central domain|PF00310:Glutamine amidotransferases class-II|PF01493:GXGXG motif|PF14691:Dihydroprymidine dehydrogenase domain II, 4Fe-4S cluster|PF01645:Conserved region in glutamate synthase|PF07992:Pyridine nucleotide-disulphide oxidoreductase
Lachesis_group0__13_contigs__l.g2651	3226.83	913.5943	1.820824	0.000342	0.004236	1155	- && Q9Y7I8.2 RecName: Full=Zuotin; AltName: Full=DnaJ-related protein zuo1; Short=J protein zuo1; AltName: Full=Ribosome-associated complex subunit zuo1 && PF00226:DnaJ domain
Lachesis_group0__13_contigs__l.g8240	591.5434	167.357	1.820079	0.001061	0.009685	1209	- && - && PF00046:Homeobox domain
Lachesis_group0__13_contigs__l.g5291	650.6706	184.4884	1.819255	0.000307	0.003919	2406	- && P78963.2 RecName: Full=Protein arginine N-methyltransferase skb1; AltName: Full=Shk1 kinase-binding protein 1; AltName: Full=Type II protein arginine N-methyltransferase; Short=Type II PRMT && PF05185:PRMT5 arginine-N-methyltransferase|PF13857:Ankyrin repeats (many copies)
Lachesis_group0__13_contigs__l.g8905	3821.695	1083.262	1.818865	0.005516	0.030999	1686	- && Q01772.1 RecName: Full=Aldehyde oxidase GLOX; AltName: Full=Glyoxal oxidase; Short=GLOX; Flags: Precursor && PF07250:Glyoxal

							oxidase N-terminus|PF09118:Domain of unknown function (DUF1929)
Lachesis_group0__13_contigs__l.g297	6363.444	1810.05	1.813981	0.000335	0.004181	4278	- && P46056.1 RecName: Full=Carbamoyl-phosphate synthase arginine-specific large chain; AltName: Full=Arginine-specific carbamoyl-phosphate synthetase, ammonia chain && PF02786:Carbamoyl-phosphate synthase L chain, ATP binding domain|PF02787:Carbamoyl-phosphate synthetase large chain, oligomerisation domain
Lachesis_group0__13_contigs__l.g9977	796.4991	227.0551	1.810473	0.000348	0.004287	1269	- && - && PF07766:LETM1-like protein
Lachesis_group0__13_contigs__l.g138	1593.445	454.6594	1.809429	0.000376	0.004553	4479	- && Q4P7M1.1 RecName: Full=ATP-dependent RNA helicase DBP9 && PF00270:DEAD/DEAH box helicase|PF00271:Helicase conserved C-terminal domain
Lachesis_group0__13_contigs__l.g5382	269.8056	76.97443	1.809192	0.000323	0.004078	1485	- && Q06137.1 RecName: Full=Putative 6-phosphofructo-2-kinase/fructose-2,6-bisphosphatase YLR345W; Includes: RecName: Full=6-phosphofructo-2-kinase; Includes: RecName: Full=Fructose-2,6-bisphosphatase && PF00300:Histidine phosphatase superfamily (branch 1)|PF01591:6-phosphofructo-2-kinase
Lachesis_group0__13_contigs__l.g10764	1656.576	472.9616	1.808888	0.000554	0.006024	3171	- && Q09833.1 RecName: Full=tRNA (uracil(54)-C(5))-methyltransferase && PF05958:tRNA (Uracil-5-)-methyltransferase
Lachesis_group0__13_contigs__l.g2110	1963.135	561.5347	1.806398	0.00023	0.003211	1356	- && - && PF11735:Cryptococcal mannosyltransferase 1

Lachesis_group0__13_contigs__l.g6391	567.4263	162.4308	1.806146	0.000557	0.006033	1281	- && Q54CP1.1 RecName: Full=Ribosomal RNA-processing protein 8 && PF05148:Hypothetical methyltransferase
Lachesis_group0__13_contigs__l.g8893	313.3811	89.85617	1.804392	0.002471	0.017509	648	- && Q9UVT8.1 RecName: Full=6,7-dimethyl-8-ribityllumazine synthase; Short=DMRL synthase; Short=LS; Short=Lumazine synthase && PF00885:6,7-dimethyl-8-ribityllumazine synthase
Lachesis_group0__13_contigs__l.g10621	618.9008	177.5313	1.802217	0.00018	0.002681	1290	- && O14341.2 RecName: Full=54S ribosomal protein L35, mitochondrial; Flags: Precursor && PF01161:Phosphatidylethanolamine-binding protein
Lachesis_group0__13_contigs__l.g11289	252.6057	72.42783	1.802117	0.001847	0.01427	837	- && O74862.1 RecName: Full=rRNA-processing protein utp23; AltName: Full=U three protein 23 && PF04900:Fcf1
Lachesis_group0__13_contigs__l.g176	449.6046	129.417	1.798134	0.00021	0.003031	1380	- && - && PF10236:Mitochondrial ribosomal death-associated protein 3
Lachesis_group0__13_contigs__l.g8723	82.22636	23.8019	1.796785	0.006269	0.033969	3117	- && - && PF10193:Telomere length regulation protein
Lachesis_group0__13_contigs__l.g3982	1411.928	406.9817	1.795549	0.001766	0.013753	2346	- && P53852.1 RecName: Full=Cysteine--tRNA ligase; AltName: Full=Cysteinyl-tRNA synthetase; Short=CysRS && PF01406:tRNA synthetases class I (C) catalytic domain
Lachesis_group0__13_contigs__l.g6468	942.1258	271.5066	1.793969	0.000631	0.006583	876	- && O13774.1 RecName: Full=GTP cyclohydrolase 1; AltName: Full=GTP cyclohydrolase I; Short=GTP-CH-I && PF01227:GTP cyclohydrolase I
Lachesis_group0__13_contigs__l.g8920	645.4573	186.2135	1.792842	0.000782	0.007721	1437	- && P0CL91.1 RecName: Full=Protein BFR2 >P0CL90.1 RecName: Full=Protein BFR2 &&

							PF13339:Apoptosis antagonizing transcription factor|PF08164:Apoptosis-antagonizing transcription factor, C-terminal
Lachesis_group0__13_contigs__l.g2709	357.361	103.5226	1.790291	0.005517	0.030999	2334	- && Q10332.1 RecName: Full=Uncharacterized ATP-dependent helicase C582.10c && PF00271:Helicase conserved C-terminal domain|PF00176:SNF2 family N-terminal domain
Lachesis_group0__13_contigs__l.g267	1680.723	487.953	1.784412	7.99E-05	0.001546	1896	- && P0C155.1 PUTATIVE PSEUDOGENE: RecName: Full=Putative carboxypeptidase YOL153C && PF07687:Peptidase dimerisation domain|PF01546:Peptidase family M20/M25/M40
Lachesis_group0__13_contigs__l.g8301	505.4413	147.0811	1.782117	0.000878	0.008412	1839	- && O74468.1 RecName: Full=Probable uroporphyrinogen-III C-methyltransferase && PF14823:Sirohaem biosynthesis protein C-terminal|PF13241:Putative NAD(P)-binding|PF00590:Tetrapyrrole (Corrin/Porphyrin) Methylases|PF14824:Sirohaem biosynthesis protein central
Lachesis_group0__13_contigs__l.g5685	466.1749	135.705	1.77977	0.000292	0.003794	987	- && P0C1I9.1 RecName: Full=Peptidyl-prolyl cis-trans isomerase cyp11; Short=PPIase cyp11; AltName: Full=Cyclophilin cyp11; AltName: Full=Rotamase cyp11 && PF00160:Cyclophilin type peptidyl-prolyl cis-trans isomerase/CLD
Lachesis_group0__13_contigs__l.g6624	3133.151	913.4994	1.778309	0.000324	0.004081	549	- && P38116.4 RecName: Full=ADP-ribosylation factor-like protein 1; AltName: Full=Arf-like GTPase 1 && PF00025:ADP-ribosylation factor family

Lachesis_group0__13_contigs__l.g10614	317.4135	92.7358	1.777381	0.0006	0.006341	858	- && O74907.2 RecName: Full=Protein bcp1 && PF13862:p21-C-terminal region-binding protein
Lachesis_group0__13_contigs__l.g6486	565.243	165.0379	1.77714	0.000418	0.004901	1359	- && Q9N4Z0.2 RecName: Full=L-2-hydroxyglutarate dehydrogenase, mitochondrial; Flags: Precursor && PF01266:FAD dependent oxidoreductase
Lachesis_group0__13_contigs__l.g7071	124.6929	36.3344	1.775923	0.000807	0.007926	2349	- && P42935.1 RecName: Full=Elongator complex protein 2; AltName: Full=Gamma-toxin target 2 && -
Lachesis_group0__13_contigs__l.g4911	23689.79	6948.974	1.769388	0.000108	0.001909	2979	- && - && PF11951:Fungal specific transcription factor domain
Lachesis_group0__13_contigs__l.g9238	630.3279	185.0097	1.768474	0.000175	0.002639	1980	- && A8NVW1.2 RecName: Full=Mitochondrial outer membrane protein IML2 && PF10300:Protein of unknown function (DUF3808)
Lachesis_group0__13_contigs__l.g11077	256.0863	75.34021	1.768256	0.000969	0.009066	342	- && O43020.2 RecName: Full=Probable 37S ribosomal protein S16, mitochondrial && PF00886:Ribosomal protein S16
Lachesis_group0__13_contigs__l.g7194	235.91	69.54624	1.766991	0.006199	0.033705	870	- && Q05918.1 RecName: Full=Tyrosine-protein phosphatase; Flags: Precursor && PF13350:Tyrosine phosphatase family|PF13348:Tyrosine phosphatase family C-terminal region
Lachesis_group0__13_contigs__l.g5875	2341.271	688.5745	1.76575	0.000183	0.002718	1533	- && A1CJ18.1 RecName: Full=ATP-dependent RNA helicase dhh1 && PF00270:DEAD/DEAH box helicase|PF00271:Helicase conserved C-terminal domain
Lachesis_group0__13_contigs__l.g9079	2220.792	653.4676	1.765162	0.000901	0.008604	2244	- && O94268.1 RecName: Full=25S rRNA (cytosine-C(5))-methyltransferase nop2; AltName:

							Full=Nucleolar protein 2 && PF01189:16S rRNA methyltransferase RsmF|PF17125:N-terminal domain of 16S rRNA methyltransferase RsmF
Lachesis_group0__13_contigs__l.g2733	2180.724	641.796	1.764898	0.001119	0.010032	1989	- && Q5AKA5.1 RecName: Full=Cys-Gly metallodipeptidase DUG1; AltName: Full=Deficient in utilization of glutathione protein 1; AltName: Full=GSH degradosomal complex subunit DUG1 && PF07687:Peptidase dimerisation domain|PF01546:Peptidase family M20/M25/M40
Lachesis_group0__13_contigs__l.g10153	1666.883	491.3302	1.762674	6.24E-05	0.001272	1701	- && P0CR53.1 RecName: Full=Pre-mRNA-splicing factor SLU7 >P0CR52.1 RecName: Full=Pre-mRNA-splicing factor SLU7 && PF11708:Pre-mRNA splicing Prp18-interacting factor
Lachesis_group0__13_contigs__l.g2950	4743.282	1398.564	1.762088	2.70E-05	0.00069	1500	- && O13639.1 RecName: Full=Adenosylhomocysteinase; Short=AdoHcyase; AltName: Full=S-adenosyl-L-homocysteine hydrolase && PF00670:S-adenosyl-L-homocysteine hydrolase, NAD binding domain|PF05221:S-adenosyl-L-homocysteine hydrolase
Lachesis_group0__13_contigs__l.g7407	3678.253	1084.749	1.761916	0.000157	0.002469	2235	- && Q4P6G4.1 RecName: Full=Eukaryotic translation initiation factor 3 subunit B; Short=eIF3b; AltName: Full=Eukaryotic translation initiation factor 3 90 kDa subunit homolog; Short=eIF3 p90; AltName: Full=Translation initiation factor eIF3 p90 subunit homolog && PF00076:RNA recognition motif. (a.k.a. RRM, RBD, or RNP domain)|PF08662:Eukaryotic translation initiation factor eIF2A

Lachesis_group0__13_contigs__l.g6823	72.47468	21.41857	1.759798	0.010369	0.048444	1062	- && - && PF00561:alpha/beta hydrolase fold
Lachesis_group0__13_contigs__l.g1294	1237.826	366.0075	1.758846	0.001338	0.011258	2220	- && Q9UUD0.1 RecName: Full=Uncharacterized protein C19C2.10 && PF03114:BAR domain|PF14604:Variant SH3 domain
Lachesis_group0__13_contigs__l.g4606	313.3839	92.69321	1.758033	0.000759	0.007536	2973	- && - && PF14197:Centrosome localisation domain of PPC89|PF06657:Centrosome microtubule-binding domain of Cep57
Lachesis_group0__13_contigs__l.g1021	129.1452	38.35205	1.754896	0.005184	0.029691	1074	- && O14003.2 RecName: Full=Replication factor C subunit 3; Short=Replication factor C3 && PF00004:ATPase family associated with various cellular activities (AAA)|PF08542:Replication factor C C-terminal domain
Lachesis_group0__13_contigs__l.g1585	251.7937	74.86303	1.753639	0.000903	0.008618	5808	- && P24686.2 RecName: Full=Negative regulator of mitosis; AltName: Full=Anaphase-promoting complex subunit 1 && PF12859:Anaphase-promoting complex subunit 1
Lachesis_group0__13_contigs__l.g1536	1224.326	363.404	1.753144	0.000208	0.00301	4551	- && Q9US40.1 RecName: Full=Uncharacterized amino-acid permease C1039.01 && PF13086:AAA domain|PF13520:Amino acid permease|PF13087:AAA domain
Lachesis_group0__13_contigs__l.g1228	443.5494	131.8135	1.752677	0.000418	0.004901	1425	- && Q05911.1 RecName: Full=Adenylosuccinate lyase; Short=ASL; AltName: Full=Adenylosuccinase; Short=ASase && PF00206:Lyase|PF10397:Adenylosuccinate lyase C-terminus
Lachesis_group0__13_contigs__l.g5919	3152.117	935.5719	1.752629	0.000133	0.002208	3033	- && Q4P763.1 RecName: Full=GMP synthase [glutamine-hydrolyzing]; AltName: Full=GMP synthetase; AltName: Full=Glutamine

							amidotransferase && PF02540:NAD synthase|PF00958:GMP synthase C terminal domain|PF00117:Glutamine amidotransferase class-I|PF04303:PrpF protein
Lachesis_group0__13_contigs__l.g10605	846.7354	251.8931	1.75014	0.000333	0.00416	1161	- && Q9UT34.1 RecName: Full=Uncharacterized protein C824.09c && PF01412:Putative GTPase activating protein for Arf
Lachesis_group0__13_contigs__l.g3102	833.9385	248.1197	1.749823	0.003231	0.021154	1188	- && Q03392.1 RecName: Full=Proliferating cell nuclear antigen; Short=PCNA && PF02747:Proliferating cell nuclear antigen, C-terminal domain|PF00705:Proliferating cell nuclear antigen, N-terminal domain
Lachesis_group0__13_contigs__l.g6933	410.3656	122.6566	1.745462	0.002959	0.019804	1146	- && O74777.1 RecName: Full=KRR1 small subunit processome component homolog; AltName: Full=KRR-R motif-containing protein 1; AltName: Full=Ribosomal RNA assembly protein mis3 && -
Lachesis_group0__13_contigs__l.g601	1226.737	366.1073	1.744209	0.001854	0.014318	1674	- && P25340.2 RecName: Full=Delta(24(24(1)))-sterol reductase; AltName: Full=C-24(28) sterol reductase; AltName: Full=Sterol Delta(24(28))-reductase && PF01222:Ergosterol biosynthesis ERG4/ERG24 family
Lachesis_group0__13_contigs__l.g6912	584.0218	174.5342	1.744082	0.004052	0.024911	4092	- && - && PF11715:Nucleoporin Nup120/160
Lachesis_group0__13_contigs__l.g5070	1008.369	301.2711	1.743861	0.000707	0.007174	2193	- && P0CM02.1 RecName: Full=Homoaconitase, mitochondrial; AltName: Full=Homoaconitate hydratase; Flags: Precursor && PF00694:Aconitase C-terminal domain|PF00330:Aconitase family (aconitate hydratase)

Lachesis_group0__13_contigs__l.g6821	1187.595	355.3261	1.741116	0.001364	0.011409	3051	- && Q5AT15.1 RecName: Full=Serine O-acetyltransferase; Flags: Precursor && PF00561:alpha/beta hydrolase fold
Lachesis_group0__13_contigs__l.g10371	3808.913	1140.851	1.739342	0.000123	0.002081	3510	- && O59811.1 RecName: Full=Cytoplasmic 60S subunit biogenesis factor SPCC550.15c; AltName: Full=pre-60S factor REI1 homolog && PF00481:Protein phosphatase 2C|PF12756:C2H2 type zinc-finger (2 copies)|PF12874:Zinc-finger of C2H2 type
Lachesis_group0__13_contigs__l.g8549	742.4138	222.432	1.739297	0.000972	0.009074	948	- && Q7ZV50.1 RecName: Full=Magnesium transporter protein 1; Short=MagT1; Flags: Precursor && PF04756:OST3 / OST6 family
Lachesis_group0__13_contigs__l.g1827	378.8118	114.1849	1.732728	0.001014	0.009373	2994	- && P37202.1 RecName: Full=Exosome complex exonuclease dis3; AltName: Full=Chromosome disjunction protein 3; AltName: Full=Mitotic control protein dis3; AltName: Full=Ribosomal RNA-processing protein 44 && PF13638:PIN domain|PF00773:RNB domain
Lachesis_group0__13_contigs__l.g811	594.1301	178.7431	1.731658	0.000515	0.00571	1677	- && C8V3W5.1 RecName: Full=NAD-dependent protein deacetylase hst1; AltName: Full=Homologous to SIR2 protein 1; AltName: Full=Regulatory protein SIR2 homolog 1 && PF02146:Sir2 family
Lachesis_group0__13_contigs__l.g5564	249.0734	75.01932	1.727178	0.010175	0.047784	1839	- && Q8NK72.2 RecName: Full=DNA repair and recombination protein rhm52; AltName: Full=RAD52 homolog && PF04098:Rad52/22 family double-strand break repair protein

Lachesis_group0__13_contigs__l.g8731	238.4596	72.09466	1.726638	0.001248	0.010759	1605	- && O48520.2 RecName: Full=DNA polymerase delta small subunit && PF04042:DNA polymerase alpha/epsilon subunit B
Lachesis_group0__13_contigs__l.g1078	361.7352	109.5034	1.726285	0.001948	0.014818	1770	- && - && PF12937:F-box-like
Lachesis_group0__13_contigs__l.g5930	5246.927	1587.09	1.724968	0.000596	0.006325	1317	- && P00046.3 RecName: Full=Cytochrome c && PF02270:Transcription initiation factor IIF, beta subunit|PF00034:Cytochrome c
Lachesis_group0__13_contigs__l.g10991	249.8229	75.87635	1.724045	0.006976	0.03662	2340	- && A3M0B1.2 RecName: Full=Pre-rRNA-processing protein IPI1 && PF12333:Rix1 complex component involved in 60S ribosome maturation
Lachesis_group0__13_contigs__l.g1461	3231.269	978.3328	1.724005	0.001329	0.011227	609	- && - && PF10342:Ser-Thr-rich glycosyl-phosphatidyl-inositol-anchored membrane family
Lachesis_group0__13_contigs__l.g8187	1057.908	320.9486	1.720906	0.001227	0.010624	1179	- && Q9Y7P4.2 RecName: Full=54S ribosomal protein rml2, mitochondrial; Short=L2 && PF00181:Ribosomal Proteins L2, RNA binding domain|PF03947:Ribosomal Proteins L2, C-terminal domain
Lachesis_group0__13_contigs__l.g9351	499.2294	151.7799	1.718666	0.00037	0.004501	2529	- && - && PF05622:HOOK protein
Lachesis_group0__13_contigs__l.g8892	1918.143	583.0601	1.718582	0.000697	0.007118	1521	- && Q9P785.1 RecName: Full=LisH domain-containing protein C1711.05 && PF05022:SRP40, C-terminal domain
Lachesis_group0__13_contigs__l.g10377	2359.608	717.1395	1.718391	0.000316	0.004001	1317	- && P17707.2 RecName: Full=S-adenosylmethionine decarboxylase proenzyme; Short=AdoMetDC; Short=SAMDC; Contains: RecName: Full=S-adenosylmethionine decarboxylase alpha chain; Contains: RecName: Full=S-adenosylmethionine decarboxylase beta

							chain; Flags: Precursor && PF01536:Adenosylmethionine decarboxylase
Lachesis_group0__13_contigs__l.g7917	443.2055	134.8545	1.71723	0.000767	0.007613	1338	- && Q9HD43.3 RecName: Full=Receptor-type tyrosine-protein phosphatase H; Short=R-PTP-H; AltName: Full=Stomach cancer-associated protein tyrosine phosphatase 1; Short=SAP-1; AltName: Full=Transmembrane-type protein-tyrosine phosphatase type H; Flags: Precursor && PF00102:Protein-tyrosine phosphatase
Lachesis_group0__13_contigs__l.g2280	11990.61	3648.901	1.716377	0.003092	0.020481	2280	- && A8PDE3.1 RecName: Full=Acetyl-coenzyme A synthetase; AltName: Full=Acetate--CoA ligase; AltName: Full=Acyl-activating enzyme && PF13193:AMP-binding enzyme C-terminal domain|PF16177:Acetyl-coenzyme A synthetase N-terminus|PF00501:AMP-binding enzyme
Lachesis_group0__13_contigs__l.g5015	836.4814	255.1385	1.713203	0.000156	0.002463	1899	- && Q9US44.1 RecName: Full=Uncharacterized transporter C1002.16c && PF07690:Major Facilitator Superfamily
Lachesis_group0__13_contigs__l.g6437	284.9951	87.11665	1.712706	0.001601	0.012759	1407	- && O36014.1 RecName: Full=Aspartyl aminopeptidase 1 && PF02127:Aminopeptidase I zinc metalloprotease (M18)
Lachesis_group0__13_contigs__l.g9042	251.5773	76.86166	1.712056	0.001767	0.013753	2076	- && O74113.1 RecName: Full=Cell division control protein 45 homolog; AltName: Full=Suppressor of nda4 protein && PF02724:CDC45-like protein
Lachesis_group0__13_contigs__l.g6749	350.8445	107.3113	1.711978	0.003054	0.02029	2934	- && P0CN25.1 RecName: Full=DNA polymerase epsilon subunit B; AltName: Full=DNA polymerase II subunit 2 >P0CN24.1 RecName: Full=DNA polymerase epsilon subunit B; AltName: Full=DNA

							polymerase II subunit 2 && PF00583:Acetyltransferase (GNAT) family|PF04042:DNA polymerase alpha/epsilon subunit B
Lachesis_group0__13_contigs__l.g2342	458.4177	140.3433	1.709387	0.001543	0.012415	1785	- && O74999.1 RecName: Full=DNA repair protein rhp7; AltName: Full=RAD7 homolog && -
Lachesis_group0__13_contigs__l.g3182	266.9903	81.82543	1.707583	0.003323	0.021578	2385	- && - && PF04121:Nuclear pore protein 84 / 107
Lachesis_group0__13_contigs__l.g8947	137.3819	41.96265	1.707321	0.009463	0.045451	627	- && - && PF07264:Etoposide-induced protein 2.4 (EI24)
Lachesis_group0__13_contigs__l.g5966	8816.209	2703.251	1.70552	0.004263	0.025803	324	- && - && PF01185:Fungal hydrophobin
Lachesis_group0__13_contigs__l.g7764	84.13832	25.68253	1.704893	0.009037	0.044047	1269	- && - && PF12937:F-box-like
Lachesis_group0__13_contigs__l.g6925	4470.293	1371.713	1.704611	0.000972	0.009074	1698	- && Q4PBF2.1 RecName: Full=Nucleolar protein 58 && PF01798:snoRNA binding domain, fibrillarin|PF08156:NOP5NT (NUC127) domain
Lachesis_group0__13_contigs__l.g32	9011.58	2770.012	1.702009	0.000462	0.005292	3222	- && O60100.1 RecName: Full=Probable importin subunit beta-4; AltName: Full=Importin-123; AltName: Full=Karyopherin subunit beta-4; AltName: Full=Karyopherin-123 && PF03810:Importin-beta N-terminal domain
Lachesis_group0__13_contigs__l.g7405	4303.136	1323.039	1.701831	0.00072	0.007266	1146	- && P0CP98.1 RecName: Full=FK506-binding protein 4; AltName: Full=Histone proline isomerase; AltName: Full=Peptidyl-prolyl cis-trans isomerase; Short=PPIase; AltName: Full=Rotamase >P0CP99.1 RecName: Full=FK506-binding protein 4; AltName: Full=Histone proline isomerase; AltName: Full=Peptidyl-prolyl cis-trans isomerase; Short=PPIase; AltName: Full=Rotamase

							&& PF00254:FKBP-type peptidyl-prolyl cis-trans isomerase
Lachesis_group0__13_contigs__l.g2257	742.1595	228.7213	1.69944	0.000484	0.0055	2394	- && Q4P5U4.2 RecName: Full=ATP-dependent RNA helicase DBP4 && PF00270:DEAD/DEAH box helicase|PF13959:Domain of unknown function (DUF4217)|PF00271:Helicase conserved C-terminal domain
Lachesis_group0__13_contigs__l.g6674	110.8773	34.17465	1.698824	0.002738	0.018808	1935	- && - && PF04129:Vps52 / Sac2 family
Lachesis_group0__13_contigs__l.g8696	272.2063	83.90768	1.697312	0.000473	0.005382	1659	- && P34809.3 RecName: Full=Glycylpeptide N-tetradecanoyltransferase; AltName: Full=Myristoyl-CoA:protein N-myristoyltransferase; Short=NMT; AltName: Full=Peptide N-myristoyltransferase && PF01233:Myristoyl-CoA:protein N-myristoyltransferase, N-terminal domain|PF02799:Myristoyl-CoA:protein N-myristoyltransferase, C-terminal domain
Lachesis_group0__13_contigs__l.g136	10339.28	3193.306	1.695015	0.000341	0.004226	1302	- && - && PF12937:F-box-like
Lachesis_group0__13_contigs__l.g10438	372.9219	115.2605	1.694317	0.003883	0.024048	1665	- && O74187.1 RecName: Full=Aldehyde dehydrogenase; Short=ALDDH; Short=ALDH && PF00171:Aldehyde dehydrogenase family
Lachesis_group0__13_contigs__l.g9814	821.2003	254.2661	1.692916	0.000875	0.00841	1032	- && A8N6B4.1 RecName: Full=Very-long-chain 3-oxoacyl-CoA reductase; AltName: Full=3-ketoacyl-CoA reductase; Short=3-ketoreductase; Short=KAR; AltName: Full=Microsomal beta-keto-reductase && PF00106:short chain dehydrogenase
Lachesis_group0__13_contigs__l.g9876	1099.264	340.5128	1.690997	0.00097	0.009066	1125	- && - && PF01040:UbiA prenyltransferase family

Lachesis_group0__13_contigs__l.g2072	1020.933	316.4513	1.690253	0.000431	0.00503	879	- && Q5BC69.1 RecName: Full=Pre-mRNA-splicing factor syf2 && PF08231:SYF2 splicing factor
Lachesis_group0__13_contigs__l.g7313	1060.55	328.9333	1.689259	0.000603	0.006366	2718	- && O43051.1 RecName: Full=Probable nucleolar complex protein 14 && PF04147:Nop14-like family
Lachesis_group0__13_contigs__l.g7729	217.9637	67.55656	1.687976	0.004518	0.026829	1071	- && G3XMC6.1 RecName: Full=Dehydrogenase azaJ; AltName: Full=Azaphilone biosynthesis cluster protein azaJ && PF08240:Alcohol dehydrogenase GroES-like domain|PF00107:Zinc-binding dehydrogenase
Lachesis_group0__13_contigs__l.g11334	2726.519	846.4598	1.687949	0.000371	0.004503	1569	- && O14007.1 RecName: Full=H/ACA ribonucleoprotein complex subunit 4; AltName: Full=Centromere-binding factor 5 homolog && PF08068:DKCLD (NUC011) domain|PF01472:PUA domain|PF16198:tRNA pseudouridylate synthase B C-terminal domain|PF01509:TruB family pseudouridylate synthase (N terminal domain)
Lachesis_group0__13_contigs__l.g9953	2448.48	761.5227	1.684869	0.000242	0.003312	1542	- && Q5AR55.1 RecName: Full=Cyrochrome P450 monooxygenase asqL; AltName: Full=4'-methoxyviridicatin/aspoquinolone biosynthesis cluster protein asqL; AltName: Full=Aspoquinolone biosynthesis protein L && PF00067:Cytochrome P450
Lachesis_group0__13_contigs__l.g6508	936.7787	291.5621	1.684363	0.001923	0.014702	468	- && Q8K933.1 RecName: Full=Single-stranded DNA-binding protein; Short=SSB && PF00436:Single-strand binding protein family
Lachesis_group0__13_contigs__l.g3422	1694.983	527.7308	1.683956	0.000598	0.006327	1098	- && P78605.1 RecName: Full=Ornithine carbamoyltransferase, mitochondrial; AltName: Full=Ornithine transcarbamylase; Short=OTCase;

							Flags: Precursor [Trametes hirsuta] && PF02729:Aspartate/ornithine carbamoyltransferase, carbamoyl-P binding domain|PF00185:Aspartate/ornithine carbamoyltransferase, Asp/Orn binding domain
Lachesis_group0__13_contigs__l.g1936	1901.293	591.9322	1.683895	0.000349	0.004291	1920	- && Q4P7G1.1 RecName: Full=Eukaryotic translation initiation factor 3 subunit G; Short=eIF3g; AltName: Full=Eukaryotic translation initiation factor 3 RNA-binding subunit; Short=eIF-3 RNA-binding subunit; AltName: Full=Translation initiation factor eIF3 p33 subunit homolog; Short=eIF3 p33 homolog && PF00076:RNA recognition motif. (a.k.a. RRM, RBD, or RNP domain)|PF12353:Eukaryotic translation initiation factor 3 subunit G|PF01370:NAD dependent epimerase/dehydratase family
Lachesis_group0__13_contigs__l.g10005	71.87706	22.34431	1.682541	0.007403	0.038296	882	- && - && PF12695:Alpha/beta hydrolase family
Lachesis_group0__13_contigs__l.g9329	206.7925	64.46382	1.682472	0.009899	0.046904	1620	- && Q9UT75.1 RecName: Full=tRNA dimethylallyltransferase, mitochondrial; AltName: Full=Isopentenyl-diphosphate: tRNA isopentenyltransferase; Short=IPP transferase; Short=IPPT; AltName: Full=tRNA isopentenyltransferase; Short=IPTase && PF01715:IPP transferase
Lachesis_group0__13_contigs__l.g732	654.6615	204.3294	1.680658	0.000323	0.004078	4482	- && Q10264.3 RecName: Full=DNA cross-link repair protein pso2/snm1 && PF07522:DNA repair metallo-beta-lactamase

Lachesis_group0__13_contigs__l.g8593	3773.505	1178.191	1.679576	0.000695	0.007101	978	- && P35551.1 RecName: Full=rRNA 2'-O-methyltransferase fibrillarin; AltName: Full=Histone-glutamine methyltransferase && PF01269:Fibrillarin
Lachesis_group0__13_contigs__l.g7746	241.5505	75.55186	1.679285	0.002934	0.019684	843	- && - && PF04000:Sas10/Utp3/C1D family
Lachesis_group0__13_contigs__l.g9485	336.5963	104.9456	1.679194	0.006323	0.034179	720	- && - && PF02410:Ribosomal silencing factor during starvation
Lachesis_group0__13_contigs__l.g5895	120.7316	37.80872	1.677202	0.006472	0.034658	798	- && - && PF05018:Protein of unknown function (DUF667)
Lachesis_group0__13_contigs__l.g4120	107.7751	33.75117	1.676037	0.002908	0.01963	681	- && Q8R040.2 RecName: Full=Ribonuclease P protein subunit p21; Short=RNaseP protein p21; AltName: Full=Ribonucleoprotein V && PF04032:RNAse P Rpr2/Rpp21/SNM1 subunit domain
Lachesis_group0__13_contigs__l.g3985	1423.426	446.2449	1.673878	0.000956	0.008972	1209	- && - && PF09729:Gti1/Pac2 family
Lachesis_group0__13_contigs__l.g4233	285.8216	89.85785	1.671456	0.001902	0.014595	1113	- && - && PF06220:U1 zinc finger
Lachesis_group0__13_contigs__l.g6814	647.7899	203.6132	1.670339	0.000617	0.006458	1560	- && Q9FG01.1 RecName: Full=Splicing factor SF3a60 homolog; AltName: Full=Protein ATROPOS; AltName: Full=Splicing factor ATO && PF16837:Pre-mRNA-splicing factor SF3A3, of SF3a complex, Prp9|PF11931:Domain of unknown function (DUF3449)|PF12171:Zinc-finger double-stranded RNA-binding|PF12108:Splicing factor SF3a60 binding domain
Lachesis_group0__13_contigs__l.g11521	130.6107	40.98282	1.66738	0.007309	0.038006	4284	- && - && PF08317:Spc7 kinetochore protein|PF15577:Spc7_C2
Lachesis_group0__13_contigs__l.g1552	256.9589	81.21261	1.663644	0.000993	0.009224	993	- && Q6K9N1.1 RecName: Full=Casein kinase 1; Short=OsCKI1; AltName: Full=Protein HYBRID

							BREAKDOWN 2; AltName: Full=Protein LOW TEMPERATURE GROWTH 1 && PF00069:Protein kinase domain
Lachesis_group0__13_contigs__l.g5292	734.8448	232.4059	1.661486	0.001405	0.011644	1389	- && Q6NUA1.1 RecName: Full=Protein arginine N-methyltransferase 5; AltName: Full=Histone synthetic lethal 7 protein; Short=Hsl7; AltName: Full=Histone-arginine N-methyltransferase PRMT5 && PF05185:PRMT5 arginine-N-methyltransferase
Lachesis_group0__13_contigs__l.g8482	102.6521	32.48351	1.660456	0.006173	0.033595	468	- && Q8R344.2 RecName: Full=Coiled-coil domain-containing protein 12 && PF08315:cwf18 pre-mRNA splicing factor
Lachesis_group0__13_contigs__l.g11479	1245.823	394.3784	1.659891	0.000437	0.005091	1512	- && O22785.3 RecName: Full=Pre-mRNA-processing factor 19 homolog 2; AltName: Full=MOS4-associated complex protein 3B; Short=MAC protein 3B; AltName: Full=Plant U-box protein 60; AltName: Full=U-box domain-containing protein 60 && PF04564:U-box domain|PF08606:Prp19/Pso4-like
Lachesis_group0__13_contigs__l.g8484	255.8246	81.41238	1.653717	0.001359	0.0114	963	- && - && PF06881:RNA polymerase II transcription factor SIII (Elongin) subunit A
Lachesis_group0__13_contigs__l.g2649	429.537	136.6848	1.652503	0.001524	0.012345	1830	- && O14059.1 RecName: Full=Probable histidinol-phosphatase; Short=HolPase && PF00111:2Fe-2S iron-sulfur cluster binding domain|PF02811:PHP domain
Lachesis_group0__13_contigs__l.g9168	1232.346	392.6969	1.650401	0.000442	0.005122	927	- && Q9USM4.1 RecName: Full=U1 snRNP-associated protein usp106; AltName: Full=Protein luc7 && PF03194:LUC7 N_terminus

Lachesis_group0__13_contigs__l.g11045	978.6273	312.3375	1.648561	0.000606	0.006377	3510	- && Q1MTQ9.1 RecName: Full=60S ribosome subunit biogenesis protein nip7 && PF08314:Secretory pathway protein Sec39|PF03657:Uncharacterised protein family (UPF0113)
Lachesis_group0__13_contigs__l.g1729	2428.733	775.1295	1.647972	0.000618	0.00647	2592	- && O13864.1 RecName: Full=Importin subunit beta-1; AltName: Full=Importin-95; AltName: Full=Karyopherin subunit beta-1; AltName: Full=Karyopherin-95 && PF13513:HEAT-like repeat|PF03810:Importin-beta N-terminal domain
Lachesis_group0__13_contigs__l.g10606	871.4651	278.4885	1.646411	0.000169	0.002583	996	- && - && PF08690:GET complex subunit GET2
Lachesis_group0__13_contigs__l.g10060	975.9093	312.0976	1.645667	0.000213	0.003068	720	- && - && PF00240:Ubiquitin family|PF02179:BAG domain
Lachesis_group0__13_contigs__l.g3642	1457.163	465.8408	1.64501	0.001361	0.011408	1053	- && - && PF00153:Mitochondrial carrier protein
Lachesis_group0__13_contigs__l.g9583	200.3492	64.08196	1.644257	0.000527	0.0058	1017	- && A8WFS8.1 RecName: Full=Post-GPI attachment to proteins factor 3; AltName: Full=PER1-like domain-containing protein 1; Flags: Precursor && PF04080:Per1-like
Lachesis_group0__13_contigs__l.g5333	403.6899	129.4485	1.641319	0.00203	0.015266	2001	- && Q9UUF2.1 RecName: Full=Probable methionine--tRNA ligase, cytoplasmic; AltName: Full=Methionyl-tRNA synthetase; Short=MetRS && PF09334:tRNA synthetases class I (M)|PF00458:WHEP-TRS domain
Lachesis_group0__13_contigs__l.g6344	920.6581	296.3433	1.63618	0.000801	0.007867	552	- && Q9P7H0.1 RecName: Full=H/ACA ribonucleoprotein complex subunit 2; AltName: Full=H/ACA snoRNP protein NHP2; AltName: Full=High mobility group-like nuclear protein 2;

							AltName: Full=P17-nhp2 && PF01248:Ribosomal protein L7Ae/L30e/S12e/Gadd45 family
Lachesis_group0__13_contigs__l.g5410	155.9781	49.99613	1.635681	0.004856	0.028391	573	- && - && PF11705:DNA-directed RNA polymerase III subunit Rpc31
Lachesis_group0__13_contigs__l.g11438	232.0569	74.77379	1.634694	0.003524	0.022533	354	- && A1DH48.1 RecName: Full=V-type proton ATPase subunit F; Short=V-ATPase subunit F; AltName: Full=V-ATPase 14 kDa subunit; AltName: Full=Vacuolar proton pump subunit F && PF01990:ATP synthase (F/14-kDa) subunit
Lachesis_group0__13_contigs__l.g8189	6564.424	2114.755	1.634191	0.002552	0.017889	1266	- && - && PF10294:Lysine methyltransferase
Lachesis_group0__13_contigs__l.g5228	865.3044	280.0539	1.628671	0.00108	0.009782	3360	- && Q5C9Z4.1 RecName: Full=Nucleolar MIF4G domain-containing protein 1; AltName: Full=SGD1 homolog && PF02854:MIF4G domain|PF02847:MA3 domain
Lachesis_group0__13_contigs__l.g8859	554.4389	179.4164	1.627667	0.001016	0.009388	1593	- && P0CR91.1 RecName: Full=Mitochondrial import inner membrane translocase subunit TIM54 >P0CR90.1 RecName: Full=Mitochondrial import inner membrane translocase subunit TIM54 && PF11711:Inner membrane protein import complex subunit Tim54
Lachesis_group0__13_contigs__l.g9222	468.3667	152.0586	1.623932	0.001028	0.009457	2100	- && Q9UTN3.2 RecName: Full=Poly(A) RNA polymerase cid14; Short=PAP; AltName: Full=Caffeine-induced death protein 14; AltName: Full=Polynucleotide adenylyltransferase cid14 && PF03828:Cid1 family poly A polymerase|PF01909:Nucleotidyltransferase domain

Lachesis_group0__13_contigs__l.g6367	1146.641	372.2692	1.623606	0.000798	0.007854	1989	- && F4I2J8.1 RecName: Full=Cactin && PF09732:Cactus-binding C-terminus of cactin protein|PF10312:Conserved mid region of cactin
Lachesis_group0__13_contigs__l.g2375	2356.299	766.1169	1.621255	0.000935	0.008827	1932	- && O42644.1 RecName: Full=CTP synthase; AltName: Full=CTP synthetase; AltName: Full=UTP--ammonia ligase && PF06418:CTP synthase N-terminus|PF00117:Glutamine amidotransferase class-I
Lachesis_group0__13_contigs__l.g7233	305.8842	99.3185	1.620727	0.006677	0.035476	477	- && Q9UTI3.1 RecName: Full=N-terminal acetyltransferase A complex catalytic subunit ard1; Short=NatA complex subunit ARD1 && PF00583:Acetyltransferase (GNAT) family
Lachesis_group0__13_contigs__l.g3664	1320.196	430.7264	1.615869	0.000579	0.006202	1077	- && Q92368.1 RecName: Full=Cell differentiation protein rcd1 && PF04078:Cell differentiation family, Rcd1-like
Lachesis_group0__13_contigs__l.g8703	966.6281	315.2794	1.615845	0.003562	0.0227	972	- && O14027.1 RecName: Full=Probable pyridoxal 5'-phosphate synthase subunit PDX1; Short=PLP synthase subunit PDX1 && PF01680:SOR/SNZ family
Lachesis_group0__13_contigs__l.g11194	887.7728	290.2686	1.613399	0.004952	0.028708	2298	- && O13910.1 RecName: Full=U3 small nucleolar ribonucleoprotein protein mpp10 && PF04006:Mpp10 protein
Lachesis_group0__13_contigs__l.g5840	329.5223	107.5886	1.613129	0.001501	0.012223	4677	- && Q94BM7.1 RecName: Full=Protein SPA1-RELATED 4 && PF00400:WD domain, G-beta repeat|PF05729:NACHT domain|PF00168:C2 domain

Lachesis_group0__13_contigs__l.g9041	334.8341	109.7949	1.611236	0.001207	0.010484	516	- && O94153.1 RecName: Full=Imidazoleglycerol-phosphate dehydratase; Short=IGPD && PF00475:Imidazoleglycerol-phosphate dehydratase
Lachesis_group0__13_contigs__l.g9026	1218.585	399.164	1.61107	0.00192	0.014691	3831	- && O74490.1 RecName: Full=Vacuolar protein sorting-associated protein ist1 && PF03398:Regulator of Vps4 activity in the MVB pathway|PF00566:Rab-GTPase-TBC domain
Lachesis_group0__13_contigs__l.g9624	483.5856	158.5626	1.610174	0.001663	0.013113	1380	- && - && PF07690:Major Facilitator Superfamily
novel.808	73.93977	24.27981	1.607838	0.009857	0.046827	370	- && P0CM71.1 RecName: Full=Cytochrome c oxidase-assembly factor COX23, mitochondrial; Flags: Precursor >P0CM70.1 RecName: Full=Cytochrome c oxidase-assembly factor COX23, mitochondrial; Flags: Precursor && PF08991:Mature-T-Cell Proliferation I type
Lachesis_group0__13_contigs__l.g377	1376.454	451.8667	1.606998	0.003364	0.021772	1059	- && Q54KL5.1 RecName: Full=WD repeat-containing protein 5 homolog && PF00400:WD domain, G-beta repeat
Lachesis_group0__13_contigs__l.g3560	1747.732	574.0572	1.606949	0.004541	0.026921	615	- && Q7T3C6.1 RecName: Full=Cleavage and polyadenylation specificity factor subunit 5 && PF13869:Nucleotide hydrolase
Lachesis_group0__13_contigs__l.g5273	1925.796	632.4938	1.606415	0.00029	0.003786	2817	- && P73177.1 RecName: Full=Uncharacterized ribonuclease sll1290 && PF00773:RNB domain
Lachesis_group0__13_contigs__l.g9606	201.4129	66.17409	1.605786	0.008532	0.042511	810	- && Q9Y7L1.2 RecName: Full=Phosducin-like protein C2A9.09 && PF02114:Phosducin
Lachesis_group0__13_contigs__l.g4862	168.7012	55.49008	1.604737	0.007344	0.038127	966	- && P74369.1 RecName: Full=UPF0014 membrane protein slr1647 && PF03649:Uncharacterised protein family (UPF0014)

Lachesis_group0__13_contigs__l.g4701	384.175	126.3256	1.603814	0.000755	0.00751	2019	- && P0CS09.1 RecName: Full=tRNA (adenine(58)-N(1))-methyltransferase catalytic subunit TRM61; AltName: Full=tRNA(m1A58)-methyltransferase subunit TRM61; Short=tRNA(m1A58)MTase subunit TRM61 >P0CS08.1 RecName: Full=tRNA (adenine(58)-N(1))-methyltransferase catalytic subunit TRM61; AltName: Full=tRNA(m1A58)-methyltransferase subunit TRM61; Short=tRNA(m1A58)MTase subunit TRM61 && PF08704:tRNA methyltransferase complex GCD14 subunit
Lachesis_group0__13_contigs__l.g5906	1192.904	392.7651	1.60341	0.002523	0.01774	834	- && - && PF05817:Oligosaccharyltransferase subunit Ribophorin II
Lachesis_group0__13_contigs__l.g3875	3049.31	1003.807	1.603145	0.000508	0.005674	891	- && P38988.1 RecName: Full=Mitochondrial GTP/GDP carrier protein 1 && PF00153:Mitochondrial carrier protein
Lachesis_group0__13_contigs__l.g4316	163.6717	53.78164	1.602658	0.004328	0.026067	5580	- && - && PF02902:Ulp1 protease family, C-terminal catalytic domain
Lachesis_group0__13_contigs__l.g251	478.2239	157.5426	1.602513	0.002189	0.016056	546	- && O60171.1 RecName: Full=Transcription initiation factor TFIID subunit 10; AltName: Full=TBP-associated factor 10 && PF03540:Transcription initiation factor TFIID 23-30kDa subunit
Lachesis_group0__13_contigs__l.g11483	832.5134	274.3722	1.602419	0.00164	0.012979	3534	- && Q8L638.1 RecName: Full=Dol-P-Glc:Glc(2)Man(9)GlcNAc(2)-PP-Dol alpha-1,2-glucosyltransferase; AltName: Full=Alpha-1,2-glucosyltransferase ALG10 homolog; AltName:

							Full=Protein HOMOLOG OF YEAST ALG10 && PF04922:DIE2/ALG10 family
Lachesis_group0__13_contigs__l.g8737	873.2607	287.7848	1.601968	0.003657	0.023131	3054	- && Q9URY8.1 RecName: Full=Probable sulfate permease C869.05c && PF00916:Sulfate permease family|PF01740:STAS domain
Lachesis_group0__13_contigs__l.g10558	412.2658	136.0365	1.601958	0.008067	0.040843	747	- && - && PF13409:Glutathione S-transferase, N-terminal domain
Lachesis_group0__13_contigs__l.g5090	1039.402	343.7267	1.59608	0.000127	0.002123	915	- && O94511.1 RecName: Full=Putative enoyl reductase C646.07c && PF02544:3-oxo-5-alpha-steroid 4-dehydrogenase
Lachesis_group0__13_contigs__l.g9367	511.7513	169.5534	1.593863	0.002228	0.016244	1314	- && Q8BVE3.1 RecName: Full=V-type proton ATPase subunit H; Short=V-ATPase subunit H; AltName: Full=Vacuolar proton pump subunit H && PF03224:V-ATPase subunit H|PF11698:V-ATPase subunit H
Lachesis_group0__13_contigs__l.g9677	5150.03	1706.824	1.593457	0.001976	0.014961	1839	- && O74965.1 RecName: Full=Eukaryotic translation initiation factor 2A; Short=eIF-2A && PF08662:Eukaryotic translation initiation factor eIF2A
Lachesis_group0__13_contigs__l.g11050	697.49	231.3445	1.593038	0.000404	0.004797	1614	- && O14156.2 RecName: Full=Protein phosphatase 2C homolog 4; Short=PP2C-4 && PF00481:Protein phosphatase 2C
Lachesis_group0__13_contigs__l.g249	2063.76	684.2843	1.59294	0.001125	0.010043	669	- && O13917.1 RecName: Full=Hypoxanthine-guanine phosphoribosyltransferase; Short=HGPRT; Short=HGPRTase && -
Lachesis_group0__13_contigs__l.g10179	187.0603	62.04667	1.58981	0.008639	0.042825	3297	- && Q82IY3.1 RecName: Full=Pentalenene oxygenase && PF00067:Cytochrome P450

Lachesis_group0__13_contigs__l.g10264	964.1552	320.527	1.589414	0.001152	0.01017	1050	- && P25605.2 RecName: Full=Acetolactate synthase small subunit, mitochondrial; AltName: Full=Acetohydroxy-acid synthase small subunit; Short=AHAS; Short=ALS; Flags: Precursor && PF10369:Small subunit of acetolactate synthase|PF01842:ACT domain
Lachesis_group0__13_contigs__l.g4114	232.3907	77.50275	1.586245	0.007291	0.037983	1482	- && Q10475.1 RecName: Full=Eukaryotic translation initiation factor 4 gamma; Short=eIF-4-gamma; Short=eIF-4G && PF02854:MIF4G domain|PF12152:Eukaryotic translation initiation factor 4G1
Lachesis_group0__13_contigs__l.g10328	857.0059	285.7596	1.585277	0.000267	0.003573	2832	- && P53973.1 RecName: Full=Histone deacetylase HDA1 && PF00850:Histone deacetylase domain|PF09757:Arb2 domain|PF01201:Ribosomal protein S8e
Lachesis_group0__13_contigs__l.g9267	410.0242	137.048	1.582596	0.000546	0.005955	2049	- && Q9H5Z1.2 RecName: Full=Probable ATP-dependent RNA helicase DHX35; AltName: Full=DEAH box protein 35 && PF07717:Oligonucleotide/oligosaccharide-binding (OB)-fold|PF00271:Helicase conserved C-terminal domain|PF04408:Helicase associated domain (HA2)
Lachesis_group0__13_contigs__l.g252	399.0875	133.2546	1.581356	0.00217	0.015959	1122	- && A0A024SMV2.1 RecName: Full=D-xylose 1-dehydrogenase (NADP(+)); Short=XDH; AltName: Full=D-xylose-NADP dehydrogenase; AltName: Full=NADP(+)-dependent D-xylose dehydrogenase && PF01408:Oxidoreductase family, NAD-binding Rossmann fold

Lachesis_group0__13_contigs__l.g10126	2901.622	972.3914	1.577293	0.000486	0.005513	1332	- && Q6C9Y4.1 RecName: Full=Carbamoyl-phosphate synthase arginine-specific small chain; Short=CPS-A; AltName: Full=Arginine-specific carbamoyl-phosphate synthetase, glutamine chain && PF00117:Glutamine amidotransferase class-I|PF00988:Carbamoyl-phosphate synthase small chain, CPSase domain
Lachesis_group0__13_contigs__l.g11571	930.1456	312.1912	1.575611	0.001988	0.015041	873	- && P78603.2 RecName: Full=Vesicular-fusion protein SEC17 && PF14938:Soluble NSF attachment protein, SNAP
Lachesis_group0__13_contigs__l.g5821	410.4887	137.8287	1.574878	0.004823	0.028229	669	- && Q9P6N2.1 RecName: Full=Pdp3-interacting factor 1 && PF12710:haloacid dehalogenase-like hydrolase
Lachesis_group0__13_contigs__l.g403	464.696	156.1934	1.573487	0.003717	0.023358	3453	- && P38360.2 RecName: Full=P-type cation-transporting ATPase; AltName: Full=Cadmium resistance protein 2; AltName: Full=Cadmium-translocating P-type ATPase; AltName: Full=Cd(2+)-exporting ATPase && PF00122:E1-E2 ATPase|PF00702:haloacid dehalogenase-like hydrolase
Lachesis_group0__13_contigs__l.g2106	1591.387	536.1457	1.569997	0.001382	0.011516	1431	- && Q9UUI4.1 RecName: Full=Uncharacterized protein C22F8.09 && PF07767:Nop53 (60S ribosomal biogenesis)
Lachesis_group0__13_contigs__l.g11395	2161.117	728.5012	1.568732	0.005184	0.029691	1200	- && O13940.1 RecName: Full=Probable low-specificity L-threonine aldolase && PF01212:Beta-eliminating lyase

Lachesis_group0__13_contigs__l.g152	351.3786	118.7302	1.568268	0.002992	0.019975	1329	- && Q08980.1 RecName: Full=Probable transport protein YPL264C && PF00892:EamA-like transporter family
Lachesis_group0__13_contigs__l.g1103	679.181	229.5241	1.565525	0.008018	0.040631	1083	- && P49349.1 RecName: Full=Farnesyl pyrophosphate synthase; Short=FPP synthase; Short=FPS; AltName: Full=(2E,6E)-farnesyl diphosphate synthase; AltName: Full=Dimethylallyltranstransferase; AltName: Full=Farnesyl diphosphate synthase; AltName: Full=Geranyltranstransferase && PF00348:Polyprenyl synthetase
Lachesis_group0__13_contigs__l.g3258	548.4974	185.5124	1.564448	0.008117	0.041	2571	- && Q74ZS2.2 RecName: Full=GPI ethanolamine phosphate transferase 3; AltName: Full=Glycosylphosphatidylinositol-anchor biosynthesis protein 13 && PF01663:Type I phosphodiesterase / nucleotide pyrophosphatase
Lachesis_group0__13_contigs__l.g9418	332.373	112.5911	1.562914	0.00174	0.013576	2577	- && Q9P7X5.1 RecName: Full=Protein kinase domain-containing protein ppk32 && PF00069:Protein kinase domain
Lachesis_group0__13_contigs__l.g11652	636.7166	215.5814	1.562558	0.000586	0.00624	504	- && P36595.1 RecName: Full=DNA-directed RNA polymerases I, II, and III subunit RPABC2; Short=RNA polymerases I, II, and III subunit ABC2; AltName: Full=DNA-directed RNA polymerases I, II, and III 15 kDa polypeptide; AltName: Full=RPC16 && PF01192:RNA polymerase Rpb6
Lachesis_group0__13_contigs__l.g4624	88.04312	29.87128	1.55954	0.009002	0.043933	534	- && O07513.1 RecName: Full=Protein hit && PF01230:HIT domain

Lachesis_group0__13_contigs__l.g9881	406.4774	137.7223	1.559139	0.002131	0.015779	1173	- && O74995.1 RecName: Full=TFIIH basal transcription factor complex p47 subunit; AltName: Full=Suppressor of stem-loop protein 1 homolog; Short=SSL1 homolog && PF04056:Ssl1-like
Lachesis_group0__13_contigs__l.g976	1513.682	514.3605	1.557002	0.001678	0.013188	1311	- && Q8WU08.2 RecName: Full=Serine/threonine-protein kinase 32A; AltName: Full=Yet another novel kinase 1 && PF00069:Protein kinase domain
Lachesis_group0__13_contigs__l.g10296	2947.785	1002.94	1.555572	0.000556	0.006033	2952	- && Q4P3W3.1 RecName: Full=ATP-dependent RNA helicase DBP10 && PF00270:DEAD/DEAH box helicase|PF08147:DBP10CT (NUC160) domain|PF00271:Helicase conserved C-terminal domain
Lachesis_group0__13_contigs__l.g7167	1388.469	472.631	1.554873	0.000907	0.008631	1275	- && Q4WVE5.1 RecName: Full=Survival factor 1 && PF08622:Svf1-like
Lachesis_group0__13_contigs__l.g9847	35106.19	11958.65	1.553675	0.001024	0.009429	2037	- && A7BHQ9.1 RecName: Full=Tyrosinase; Flags: Precursor && PF00264:Common central domain of tyrosinase
Lachesis_group0__13_contigs__l.g6694	190.3588	64.7676	1.553383	0.008342	0.041801	768	- && B4LPP8.1 RecName: Full=Protein crossbronx-like && PF00179:Ubiquitin-conjugating enzyme
Lachesis_group0__13_contigs__l.g982	695.8929	237.6158	1.551016	0.003722	0.023367	1245	- && - && PF05172:Nup53/35/40-type RNA recognition motif
Lachesis_group0__13_contigs__l.g8681	4682.422	1600.099	1.549225	0.000366	0.004474	2808	- && Q8WZJ7.1 RecName: Full=C-1-tetrahydrofolate synthase, cytoplasmic; Short=C1-THF synthase; Includes: RecName: Full=Methylenetetrahydrofolate dehydrogenase; Includes: RecName: Full=Methenyltetrahydrofolate cyclohydrolase; Includes: RecName: Full=Formyltetrahydrofolate

							synthetase && PF01268:Formate--tetrahydrofolate ligase|PF02882:Tetrahydrofolate dehydrogenase/cyclohydrolase, NAD(P)-binding domain|PF00763:Tetrahydrofolate dehydrogenase/cyclohydrolase, catalytic domain
Lachesis_group0__13_contigs__l.g563	996.6448	340.651	1.549188	0.000304	0.003896	1563	- && Q52KV5.1 RecName: Full=RNA polymerase-associated protein LEO1 && PF04004:Leo1-like protein
Lachesis_group0__13_contigs__l.g3075	317.1304	108.3733	1.549088	0.00236	0.01699	3054	- && - && PF00320:GATA zinc finger
Lachesis_group0__13_contigs__l.g5195	371.2629	126.8236	1.547963	0.002016	0.015214	2784	- && A7UX13.1 RecName: Full=Hercynylcysteine sulfoxide lyase; AltName: Full=Ergothioneine biosynthesis protein 2; AltName: Full=PLP-binding cysteine desulfurase; AltName: Full=PLP-dependent C-S lyase && PF10288:Cytoplasmic tRNA 2-thiolation protein 2|PF00266:Aminotransferase class-V
Lachesis_group0__13_contigs__l.g309	299.8263	102.6457	1.546885	0.001261	0.010829	1866	- && Q6FV05.1 RecName: Full=tRNA pseudouridine synthase 1; AltName: Full=tRNA pseudouridylate synthase 1; AltName: Full=tRNA-uridine isomerase 1 && PF01416:tRNA pseudouridine synthase
Lachesis_group0__13_contigs__l.g9364	348.634	119.5248	1.546457	0.009304	0.044925	1035	- && Q10480.2 RecName: Full=Nuclease 1, mitochondrial; AltName: Full=SpNUC1; Flags: Precursor && PF01223:DNA/RNA non-specific endonuclease
Lachesis_group0__13_contigs__l.g2710	1640.683	562.2097	1.545241	0.000406	0.004813	3306	- && Q92540.2 RecName: Full=Protein SMG7; AltName: Full=EST1-like protein C; AltName: Full=SMG-7 homolog; Short=hSMG-7 &&

							PF10373:Est1 DNA/RNA binding domain|PF10374:Telomerase activating protein Est1
Lachesis_group0__13_contigs__l.g6630	365.2233	125.049	1.544803	0.002308	0.01669	924	- && - && PF10406:Transcription factor TFIID complex subunit 8 C-term|PF07524:Bromodomain associated
Lachesis_group0__13_contigs__l.g7350	239.8861	82.30354	1.544549	0.002098	0.015603	471	- && Q12487.1 RecName: Full=54S ribosomal protein L23, mitochondrial; AltName: Full=YmL23; Flags: Precursor && PF00572:Ribosomal protein L13
Lachesis_group0__13_contigs__l.g6692	732.2781	250.9069	1.5442	0.003688	0.023277	3762	- && Q04656.3 RecName: Full=Copper-transporting ATPase 1; AltName: Full=Copper pump 1; AltName: Full=Menkes disease-associated protein && PF00122:E1-E2 ATPase|PF00702:haloacid dehalogenase-like hydrolase|PF00403:Heavy-metal-associated domain
Lachesis_group0__13_contigs__l.g3663	384.6581	131.7291	1.544	0.00977	0.04656	831	- && D4AK18.2 RecName: Full=Uncharacterized secreted protein ARB_06907; Flags: Precursor && -
Lachesis_group0__13_contigs__l.g2737	311.5944	107.1523	1.543743	0.007307	0.038006	2064	- && A8NZY7.1 RecName: Full=tRNA-dihydrouridine(47) synthase [NAD(P)(+)]; AltName: Full=tRNA-dihydrouridine synthase 3 && PF01207:Dihydrouridine synthase (Dus)
Lachesis_group0__13_contigs__l.g9965	448.8509	154.0825	1.542841	0.001186	0.010357	1008	- && Q09923.1 RecName: Full=Aldo-keto reductase yakc [NADP(+)] && PF00248:Aldo/keto reductase family
Lachesis_group0__13_contigs__l.g2208	711.8411	244.4641	1.542274	0.001873	0.014419	300	- && Q6Q560.1 RecName: Full=Protein ISD11; AltName: Full=Iron-sulfur protein biogenesis,

							desulfurase-interacting protein 11 && PF13232:Complex1_LYR-like
Lachesis_group0__13_contigs__l.g6183	820.6325	281.9483	1.542247	0.001431	0.011796	1503	- && P0CR27.1 RecName: Full=RuvB-like helicase 1 >P0CR26.1 RecName: Full=RuvB-like helicase 1 && PF06068:TIP49 C-terminus
Lachesis_group0__13_contigs__l.g2760	1880.913	646.0812	1.541916	0.001392	0.011577	1041	- && Q6ING7.1 RecName: Full=FAD synthase; AltName: Full=FAD pyrophosphorylase; AltName: Full=FMN adenylyltransferase; AltName: Full=Flavin adenine dinucleotide synthase; Includes: RecName: Full=Molybdenum cofactor biosynthesis protein-like region; Includes: RecName: Full=FAD synthase region && PF01507:Phosphoadenosine phosphosulfate reductase family
Lachesis_group0__13_contigs__l.g8140	343.945	118.0372	1.541849	0.002549	0.017876	1428	- && Q10209.1 RecName: Full=Uncharacterized J domain-containing protein C4H3.01 && PF00226:DnaJ domain|PF14308:X-domain of DnaJ-containing
Lachesis_group0__13_contigs__l.g8698	515.8999	177.286	1.54164	0.002221	0.016226	1617	- && P50999.2 RecName: Full=T-complex protein 1 subunit delta; Short=TCP-1-delta; AltName: Full=CCT-delta && PF00118:TCP-1/cpn60 chaperonin family
Lachesis_group0__13_contigs__l.g1141	613.5137	210.9479	1.541446	0.001004	0.009308	1890	- && P0CP42.1 RecName: Full=Oxidation resistance protein 1 && PF07534:TLD
Lachesis_group0__13_contigs__l.g5547	8690.369	2986.067	1.541309	0.010748	0.049789	1065	- && O14321.1 RecName: Full=Sterol 24-C-methyltransferase erg6; AltName: Full=Delta(24)-sterol C-methyltransferase erg6; AltName: Full=Ergosterol biosynthesis protein 6 &&

							PF13847:Methyltransferase domain|PF08498:Sterol methyltransferase C-terminal
Lachesis_group0__13_contigs__l.g9109	189.7579	65.18199	1.539252	0.001541	0.012402	7764	- && P04323.1 RecName: Full=Retrovirus-related Pol polyprotein from transposon 17.6; Includes: RecName: Full=Protease; Includes: RecName: Full=Reverse transcriptase; Includes: RecName: Full=Endonuclease && PF00078:Reverse transcriptase (RNA-dependent DNA polymerase)|PF00385:Chromo (CHRromatin Organisation MOdifier) domain|PF12862:Anaphase-promoting complex subunit 5|PF00665:Integrase core domain
Lachesis_group0__13_contigs__l.g10872	249.8156	86.05595	1.537982	0.006973	0.03662	630	- && O42881.1 RecName: Full=Acyl-protein thioesterase 1 && PF02230:Phospholipase/Carboxylesterase
Lachesis_group0__13_contigs__l.g4936	363.9095	125.5084	1.536403	0.002208	0.016162	1479	- && - && PF13634:Nucleoporin FG repeat region
Lachesis_group0__13_contigs__l.g10267	1734.305	599.3083	1.533274	0.000631	0.006583	3429	- && Q4P3S3.1 RecName: Full=Histone acetyltransferase ESA1 && PF01853:MOZ/SAS family|PF11717:RNA binding activity-knot of a chromodomain
Lachesis_group0__13_contigs__l.g5259	756.2151	261.1563	1.533254	0.007202	0.03759	972	- && P40387.2 RecName: Full=Alpha,alpha-trehalose-phosphate synthase [UDP-forming]; AltName: Full=Trehalose-6-phosphate synthase; AltName: Full=UDP-glucose-glucosephosphate glucosyltransferase && PF00982:Glycosyltransferase family 20
Lachesis_group0__13_contigs__l.g2544	1193.889	412.5882	1.533071	0.000828	0.008088	1911	- && - && PF01544:CorA-like Mg2+ transporter protein

Lachesis_group0__13_contigs__l.g5592	259.3208	89.83535	1.53122		0.003265	0.021331	339	- && Q09177.1 RecName: Full=DNA-directed RNA polymerases I and III subunit RPAC2; Short=RNA polymerases I and III subunit AC2; AltName: Full=AC19; AltName: Full=DNA-directed RNA polymerases I and III 14 kDa polypeptide && PF13656:RNA polymerase Rpb3/Rpb11 dimerisation domain
Lachesis_group0__13_contigs__l.g9396	523.5026	181.3533	1.53028		0.002845	0.019334	927	- && P16451.1 RecName: Full=Pyruvate dehydrogenase complex protein X component, mitochondrial; AltName: Full=Dihydrolipoamide dehydrogenase-binding protein of pyruvate dehydrogenase complex; AltName: Full=E3-binding protein; AltName: Full=Pyruvate dehydrogenase complex component E3BP; Flags: Precursor && PF02817:e3 binding domain|PF00364:Biotin-requiring enzyme
Lachesis_group0__13_contigs__l.g9547	636.6397	220.6349	1.52914		0.003699	0.023293	1863	- && O13298.1 RecName: Full=Histone deacetylase phd1 && PF00850:Histone deacetylase domain
Lachesis_group0__13_contigs__l.g11369	8762.088	3036.059	1.529129		0.001137	0.010099	1920	- && Q9P7H3.1 RecName: Full=Prohibitin-1 && PF00076:RNA recognition motif. (a.k.a. RRM, RBD, or RNP domain)|PF01145:SPFH domain / Band 7 family
Lachesis_group0__13_contigs__l.g10220	389.3244	135.1736	1.526243		0.001425	0.011784	1284	- && Q90YA8.1 RecName: Full=Glutaminyl-peptide cyclotransferase; AltName: Full=Glutaminyl cyclase; Short=QC; AltName: Full=Glutaminyl-tRNA cyclotransferase; Flags: Precursor && PF04389:Peptidase family M28

Lachesis_group0__13_contigs__l.g3533	466.9444	163.0375	1.519274	0.002804	0.019128	906	- && Q9UTM0.1 RecName: Full=INO80 complex subunit 1 && PF00096:Zinc finger, C2H2 type
Lachesis_group0__13_contigs__l.g9096	404.0743	140.8876	1.519201	0.002058	0.015424	894	- && P38747.1 RecName: Full=OTU domain-containing protein 2 && PF02338:OTU-like cysteine protease
Lachesis_group0__13_contigs__l.g10098	444.7878	155.3973	1.5189	0.002872	0.019457	1011	- && P87055.1 RecName: Full=Uncharacterized protein C57A10.07 && -
Lachesis_group0__13_contigs__l.g2025	91.34896	31.85376	1.518777	0.008594	0.042736	1167	- && Q9LVH5.1 RecName: Full=Outer envelope protein 64, chloroplastic; AltName: Full=Translocon at the outer membrane of chloroplasts 64-III && PF13414:TPR repeat|PF07719:Tetratricopeptide repeat|PF13877:Potential Monad-binding region of RPAP3
Lachesis_group0__13_contigs__l.g11453	2356.158	822.5679	1.518705	0.006669	0.035453	876	- && Q55ED1.1 RecName: Full=Probable nucleosome assembly protein && PF00956:Nucleosome assembly protein (NAP)
Lachesis_group0__13_contigs__l.g2486	390.6328	136.3447	1.517979	0.002108	0.015667	2322	- && Q9D0N7.1 RecName: Full=Chromatin assembly factor 1 subunit B; Short=CAF-1 subunit B; AltName: Full=Chromatin assembly factor I p60 subunit; Short=CAF-I 60 kDa subunit; Short=CAF-I p60 && -
Lachesis_group0__13_contigs__l.g4470	180.164	62.9033	1.517383	0.003299	0.021453	3276	- && Q70LM4.1 RecName: Full=Linear gramicidin synthase subunit D; Includes: RecName: Full=ATP-dependent D-leucine adenylase; Short=D-LeuA; AltName: Full=D-leucine activase; Includes: RecName: Full=Leucine racemase [ATP-hydrolyzing]; Includes: RecName: Full=ATP-

							dependent tryptophan adenylase; Short=TrpA; AltName: Full=Tryptophan activase; Includes: RecName: Full=ATP-dependent glycine adenylase; Short=GlyA; AltName: Full=Glycine activase; Includes: RecName: Full=Linear gramicidin--PCP reductase && PF00501:AMP-binding enzyme|PF07993:Male sterility protein
Lachesis_group0__13_contigs__l.g6656	182.3389	64.01624	1.514206	0.008873	0.043652	1707	- && P0CN19.1 RecName: Full=Diphthamide biosynthesis protein 1 >P0CN18.1 RecName: Full=Diphthamide biosynthesis protein 1 && PF01866:Putative diphthamide synthesis protein
Lachesis_group0__13_contigs__l.g9555	277.8795	97.43087	1.513542	0.006936	0.036474	456	- && O94520.1 RecName: Full=ER membrane protein complex subunit 4 && PF06417:Protein of unknown function (DUF1077)
Lachesis_group0__13_contigs__l.g5783	581.0167	203.6357	1.512958	0.001689	0.013251	717	- && P35521.1 RecName: Full=Methylosome subunit pICln; AltName: Full=Chloride channel, nucleotide sensitive 1A; AltName: Full=Chloride conductance regulatory protein ICln; Short=I(Cln) && PF03517:Regulator of volume decrease after cellular swelling
Lachesis_group0__13_contigs__l.g10093	1026.128	359.9569	1.512414	0.006542	0.034946	1659	- && O94515.1 RecName: Full=T-complex protein 1 subunit zeta; Short=TCP-1-zeta; AltName: Full=CCT-zeta && PF00118:TCP-1/cpn60 chaperonin family
Lachesis_group0__13_contigs__l.g7980	15818.93	5552.427	1.510459	0.001736	0.013556	1278	- && - && PF12937:F-box-like
Lachesis_group0__13_contigs__l.g1211	985.5228	346.3226	1.509443	0.003885	0.024048	789	- && Q8AVU2.1 RecName: Full=Ubiquitin-conjugating enzyme E2 S-B; AltName: Full=E2 ubiquitin-conjugating enzyme S-B; AltName:

							Full=Ubiquitin carrier protein S-B; AltName: Full=Ubiquitin-protein ligase S-B && PF00179:Ubiquitin-conjugating enzyme
Lachesis_group0__13_contigs__l.g11270	2048.339	719.9356	1.508592	0.003956	0.024443	1644	- && O74407.1 RecName: Full=Aspartate--tRNA ligase, cytoplasmic; AltName: Full=Aspartyl-tRNA synthetase; Short=AspRS && PF01336:OB-fold nucleic acid binding domain|PF00152:tRNA synthetases class II (D, K and N)
Lachesis_group0__13_contigs__l.g11401	947.3125	333.3717	1.506964	0.003779	0.023563	876	- && P0CS65.1 RecName: Full=mRNA 3'-end-processing protein YTH1 >P0CS64.1 RecName: Full=mRNA 3'-end-processing protein YTH1 && -
Lachesis_group0__13_contigs__l.g4220	3586.994	1263.415	1.505583	0.000352	0.004325	1239	- && O74803.1 RecName: Full=UV excision repair protein rhp23; AltName: Full=RAD23 homolog && PF09280:XPC-binding domain|PF00627:UBA/TS-N domain|PF00240:Ubiquitin family
Lachesis_group0__13_contigs__l.g2007	155.9403	54.81907	1.505531	0.004805	0.028136	1695	- && Q7XJS0.2 RecName: Full=Histone-lysine N-methyltransferase ASHR1; AltName: Full=ASH1-related protein 1; AltName: Full=Protein SET DOMAIN GROUP 37 && PF00856:SET domain|PF01753:MYND finger
Lachesis_group0__13_contigs__l.g10349	775.9914	273.3211	1.504912	0.004012	0.024706	393	- && Q9P7L9.1 RecName: Full=Probable 54S ribosomal protein L34, mitochondrial; Short=L34mt; Flags: Precursor && PF00468:Ribosomal protein L34
Lachesis_group0__13_contigs__l.g11306	444.7503	157.1076	1.503233	0.006448	0.034563	471	- && P52434.4 RecName: Full=DNA-directed RNA polymerases I, II, and III subunit RPABC3; Short=RNA polymerases I, II, and III subunit ABC3; AltName: Full=DNA-directed RNA polymerase II

							subunit H; AltName: Full=DNA-directed RNA polymerases I, II, and III 17.1 kDa polypeptide; AltName: Full=RPB17; AltName: Full=RPB8 homolog; Short=hRPB8 >Q923G2.3 RecName: Full=DNA-directed RNA polymerases I, II, and III subunit RPABC3; Short=RNA polymerases I, II, and III subunit ABC3; AltName: Full=DNA-directed RNA polymerase II subunit H; AltName: Full=RPB17; AltName: Full=RPB8 homolog && PF03870:RNA polymerase Rpb8
Lachesis_group0__13_contigs__l.g11302	345.9473	122.0702	1.503151	0.003021	0.020134	231	- && O14036.1 RecName: Full=Small nuclear ribonucleoprotein Sm D2; Short=Sm-D2; AltName: Full=Complexed with cdc5 protein 9; AltName: Full=snRNP core protein D2 && PF01423:LSM domain
Lachesis_group0__13_contigs__l.g5017	338.8097	119.5256	1.503036	0.001499	0.012223	1770	- && - && PF00566:Rab-GTPase-TBC domain
Lachesis_group0__13_contigs__l.g10873	343.6053	121.3828	1.501249	0.003451	0.022173	1665	- && P21269.1 RecName: Full=CCA tRNA nucleotidyltransferase, mitochondrial; AltName: Full=CCA-adding enzyme; AltName: Full=tRNA CCA-pyrophosphorylase; AltName: Full=tRNA adenylyltransferase; AltName: Full=tRNA nucleotidyltransferase; Flags: Precursor && PF01743:Poly A polymerase head domain
Lachesis_group0__13_contigs__l.g8667	334.6427	118.3194	1.500241	0.001208	0.010488	1887	- && - && PF00096:Zinc finger, C2H2 type
Lachesis_group0__13_contigs__l.g2253	1388.456	490.9945	1.499418	0.001331	0.011227	1779	- && O59682.1 RecName: Full=Serine palmitoyltransferase 1; Short=SPT 1; Short=SPT1; AltName: Full=Long chain base biosynthesis

								protein 1 && PF00155:Aminotransferase class I and II
Lachesis_group0__13_contigs__l.g5959	1575.252	558.2841	1.49693		0.001078	0.009781	2553	- && Q9UTM4.1 RecName: Full=T-complex protein 1 subunit epsilon; Short=TCP-1-epsilon; AltName: Full=CCT-epsilon && PF00118:TCP-1/cpn60 chaperonin family|PF00149:Calcineurin-like phosphoesterase
Lachesis_group0__13_contigs__l.g1198	380.2647	134.9224	1.495578		0.002133	0.015781	378	- && O04438.1 RecName: Full=Signal recognition particle 9 kDa protein; Short=SRP9 && PF05486:Signal recognition particle 9 kDa protein (SRP9)
Lachesis_group0__13_contigs__l.g6583	1046.688	372.3132	1.491653		0.000672	0.00694	1107	- && P33333.1 RecName: Full=Probable 1-acyl-sn-glycerol-3-phosphate acyltransferase; Short=1-AGP acyltransferase; Short=1-AGPAT; AltName: Full=Lysophosphatidic acid acyltransferase; Short=LPAAT && PF01553:Acyltransferase
Lachesis_group0__13_contigs__l.g3666	438.814	156.1935	1.491563		0.00236	0.01699	1815	- && O94336.1 RecName: Full=Uncharacterized FCP1 homology domain-containing protein C1271.03c && PF03031:NLI interacting factor-like phosphatase
Lachesis_group0__13_contigs__l.g348	552.5878	196.6143	1.489484		0.009225	0.044634	1776	- && Q9HE13.1 RecName: Full=Uncharacterized MFS-type transporter C1399.02 && PF07690:Major Facilitator Superfamily
Lachesis_group0__13_contigs__l.g1116	568.4572	202.9623	1.486195		0.000934	0.008823	1038	- && - && PF13383:Methyltransferase domain
Lachesis_group0__13_contigs__l.g4203	1492.938	534.1017	1.483456		0.001226	0.010623	1122	- && O43036.1 RecName: Full=DNA-directed RNA polymerase I subunit rpa43; Short=RNA polymerase I subunit A43; AltName: Full=DNA-dependent RNA polymerase 19 kDa polypeptide

								&& PF03876:SHS2 domain found in N terminus of Rpb7p/Rpc25p/MJ0397
Lachesis_group0__13_contigs__l.g2011	1000.888	358.3565	1.482346		0.001132	0.010067	2781	- && Q10155.1 RecName: Full=Ribonuclease Z 1; Short=RNase Z 1; AltName: Full=tRNA 3 endonuclease 1; AltName: Full=tRNase Z 1 && PF12706:Beta-lactamase superfamily domain|PF13691:tRNase Z endonuclease
Lachesis_group0__13_contigs__l.g11118	654.9982	234.6398	1.482226		0.005577	0.031189	789	- && A1CXQ2.1 RecName: Full=37S ribosomal protein S25, mitochondrial && PF13741:Mitochondrial ribosomal protein S25
Lachesis_group0__13_contigs__l.g9837	170.3176	61.06429	1.481788		0.007566	0.038934	1227	- && Q9P7G5.1 RecName: Full=Protein transport protein bos1 && PF12352:Snare region anchored in the vesicle membrane C-terminus
Lachesis_group0__13_contigs__l.g8815	455.2267	162.8537	1.480869		0.004489	0.026711	2661	- && Q54YS0.2 RecName: Full=DNA-binding protein DDB_G0278111 && PF04082:Fungal specific transcription factor domain|PF01984:Double-stranded DNA-binding domain
Lachesis_group0__13_contigs__l.g11398	2825.28	1012.584	1.480523		0.007353	0.038127	2682	- && O60161.1 RecName: Full=U3 small nucleolar RNA-associated protein 4; Short=U3 snoRNA-associated protein 4; AltName: Full=U3 protein 4 required for transcription && -
Lachesis_group0__13_contigs__l.g10199	537.8547	192.8173	1.480158		0.002145	0.015826	549	- && Q6DBY2.1 RecName: Full=N-alpha-acetyltransferase 50; AltName: Full=N-acetyltransferase NAT13; AltName: Full=NatE catalytic subunit && PF00583:Acetyltransferase (GNAT) family

Lachesis_group0__13_contigs__l.g10608	201.6557	72.3931	1.479887	0.003042	0.020237	1326	- && - && PF08265:YL1 nuclear protein C-terminal domain|PF05764:YL1 nuclear protein
Lachesis_group0__13_contigs__l.g1008	1470.668	527.3777	1.479723	0.001558	0.012504	1380	- && Q01852.1 RecName: Full=Mitochondrial import inner membrane translocase subunit TIM44; AltName: Full=Inner membrane import site protein 45; Short=ISP45; AltName: Full=Membrane import machinery protein MIM44; AltName: Full=Mitochondrial protein import protein 1; Flags: Precursor && PF04280:Tim44-like domain
Lachesis_group0__13_contigs__l.g6309	1285.703	461.4939	1.478536	0.000657	0.006808	1812	- && Q6DRJ9.1 RecName: Full=Synembryn-B; AltName: Full=Protein Ric-8B; AltName: Full=Synembryn-like && PF10165:Guanine nucleotide exchange factor synembryn
Lachesis_group0__13_contigs__l.g2479	1504.115	539.9707	1.478201	0.001272	0.010914	1074	- && P56329.2 RecName: Full=Probable eukaryotic translation initiation factor 2 subunit beta; Short=eIF-2-beta && PF01873:Domain found in IF2B/IF5
Lachesis_group0__13_contigs__l.g3902	1630.394	585.5587	1.477897	0.002535	0.017806	4077	- && O14228.1 RecName: Full=Probable phosphoribosylformylglycinamidine synthase; Short=FGAM synthase; Short=FGAMS; AltName: Full=Formylglycinamide ribonucleotide amidotransferase; Short=FGAR amidotransferase; Short=FGAR-AT; AltName: Full=Formylglycinamide ribotide amidotransferase && PF13507:CobB/CobQ-like glutamine amidotransferase domain|PF02769:AIR synthase related protein, C-terminal domain

Lachesis_group0__13_contigs__l.g4207	506.0264	181.7187	1.477034	0.001334	0.011237	798	- && O14346.2 RecName: Full=Probable very-long-chain (3R)-3-hydroxyacyl-CoA dehydratase; AltName: Full=3-hydroxyacyl-CoA dehydratase; Short=HACD && PF04387:Protein tyrosine phosphatase-like protein, PTPLA
Lachesis_group0__13_contigs__l.g8420	2544.992	914.414	1.476738	0.001186	0.010357	1299	- && B0DQB9.1 RecName: Full=Adenylosuccinate synthetase 1; Short=AMPSase 1; Short=AdSS 1; AltName: Full=IMP--aspartate ligase 1 && PF00709:Adenylosuccinate synthetase
Lachesis_group0__13_contigs__l.g5569	405.4839	145.6286	1.476701	0.001113	0.010014	1248	- && B8N8Q9.1 RecName: Full=NADPH dehydrogenase afvA; AltName: Full=Aflavarin synthesis protein A && PF00724:NADH:flavin oxidoreductase / NADH oxidase family
Lachesis_group0__13_contigs__l.g6764	262.4217	94.34679	1.47567	0.002912	0.01963	1494	- && P53960.1 RecName: Full=Putative alanyl-tRNA editing protein alaX; Short=AlaX; Short=AlaXp; Short=AlaXp-II; AltName: Full=Alanyl-tRNA deacylase alaX && PF01411:tRNA synthetases class II (A)
Lachesis_group0__13_contigs__l.g9618	3162.247	1137.696	1.474956	0.000633	0.006596	1314	- && O59948.1 RecName: Full=Eukaryotic peptide chain release factor subunit 1; Short=Eukaryotic release factor 1; Short=eRF1 && PF03463:eRF1 domain 1|PF03464:eRF1 domain 2|PF03465:eRF1 domain 3
Lachesis_group0__13_contigs__l.g5845	2109.667	759.3373	1.474006	0.001952	0.014841	3162	- && P08092.2 RecName: Full=Negative regulator of sexual conjugation and meiosis && PF00069:Protein kinase domain
Lachesis_group0__13_contigs__l.g9244	1347.581	485.2552	1.473387	0.003749	0.023456	2022	- && Q02953.2 RecName: Full=Heat shock factor protein; Short=HSF; AltName: Full=Heat shock

							transcription factor; Short=HSTF && PF00447:HSF-type DNA-binding
Lachesis_group0__13_contigs__l.g2767	1827.256	658.1541	1.473265	0.003541	0.022582	1815	- && Q9P7C0.1 RecName: Full=Uncharacterized WD repeat-containing protein C2E1P5.05 && -
Lachesis_group0__13_contigs__l.g6775	682.1389	246.1168	1.471987	0.00547	0.030845	606	- && O14225.1 RecName: Full=Mitochondrial import receptor subunit tom20; AltName: Full=Mitochondrial 20 kDa outer membrane protein; AltName: Full=Translocase of outer membrane 20 kDa subunit && PF02064:MAS20 protein import receptor
Lachesis_group0__13_contigs__l.g5606	681.9103	245.9975	1.471655	0.002362	0.01699	375	- && Q9VU02.1 RecName: Full=Probable small nuclear ribonucleoprotein Sm D1; Short=Sm-D1; AltName: Full=snRNP core protein D1 && PF01423:LSM domain
Lachesis_group0__13_contigs__l.g4122	1370.285	494.3632	1.471507	0.002204	0.016142	3642	- && Q4PGM6.1 RecName: Full=Pre-mRNA-splicing factor RSE1 && PF03178:CPSF A subunit region|PF10433:Mono-functional DNA-alkylating methyl methanesulfonate N-term
Lachesis_group0__13_contigs__l.g82	9393.064	3389.518	1.470521	0.00062	0.006482	1479	- && - && PF01823:MAC/Perforin domain
Lachesis_group0__13_contigs__l.g1282	2112.563	762.6562	1.470263	0.0015	0.012223	4227	- && P13433.2 RecName: Full=DNA-directed RNA polymerase, mitochondrial; Flags: Precursor && PF00940:DNA-dependent RNA polymerase|PF14700:DNA-directed RNA polymerase N-terminal
Lachesis_group0__13_contigs__l.g1746	2919.946	1054.328	1.469746	0.000777	0.007679	1431	- && Q09130.1 RecName: Full=Eukaryotic translation initiation factor 2 subunit gamma; Short=eIF-2-gamma && PF03144:Elongation factor Tu domain 2|PF09173:Initiation factor eIF2 gamma,

							C terminal|PF00009:Elongation factor Tu GTP binding domain
Lachesis_group0__13_contigs__l.g1503	3428.851	1238.35	1.469263	0.001507	0.012257	1323	- && Q4PBZ9.2 RecName: Full=Non-histone chromosomal protein 6 && PF00505:HMG (high mobility group) box|PF01975:Survival protein SurE
Lachesis_group0__13_contigs__l.g6569	4022.067	1453.547	1.468407	0.001385	0.011532	1725	- && Q4PEZ2.1 RecName: Full=Eukaryotic translation initiation factor 3 subunit D; Short=eIF3d && PF05091:Eukaryotic translation initiation factor 3 subunit 7 (eIF-3)
Lachesis_group0__13_contigs__l.g6813	1759.133	636.4425	1.467217	0.001534	0.012385	1248	- && P0CQ73.1 RecName: Full=ATP-dependent RNA helicase FAL1 >P0CQ72.1 RecName: Full=ATP-dependent RNA helicase FAL1 && PF00271:Helicase conserved C-terminal domain|PF00270:DEAD/DEAH box helicase
Lachesis_group0__13_contigs__l.g2816	230.0809	83.5757	1.463906	0.007703	0.039531	1509	- && - && PF03151:Triose-phosphate Transporter family
Lachesis_group0__13_contigs__l.g2403	6780.885	2458.408	1.463857	0.002613	0.018153	2076	- && Q5ZBH5.1 RecName: Full=DEAD-box ATP-dependent RNA helicase 25 && PF00271:Helicase conserved C-terminal domain|PF00270:DEAD/DEAH box helicase
Lachesis_group0__13_contigs__l.g4147	257.2061	93.1696	1.46322	0.002113	0.015693	2001	- && Q5ZLG0.1 RecName: Full=Acetoacetyl-CoA synthetase && PF00501:AMP-binding enzyme
Lachesis_group0__13_contigs__l.g7460	176.0632	63.79076	1.462107	0.003883	0.024048	597	- && P0CR81.1 RecName: Full=Translation machinery-associated protein 22 >P0CR80.1 RecName: Full=Translation machinery-associated protein 22 && PF01253:Translation initiation factor SUI1

Lachesis_group0__13_contigs__l.g4806	808.5277	294.1818	1.459098	0.003264	0.021331	2127	- && P0CL92.1 RecName: Full=Iron-sulfur clusters transporter ATM1, mitochondrial; Flags: Precursor && PF00664:ABC transporter transmembrane region|PF00005:ABC transporter|PF07673:Protein of unknown function (DUF1602)
Lachesis_group0__13_contigs__l.g616	729.3939	265.4848	1.458912	0.00436	0.026191	864	- && A3RCV9.1 RecName: Full=Eukaryotic translation initiation factor NCBP; AltName: Full=Novel cap-binding protein; Short=nCBP; AltName: Full=mRNA cap-binding protein && PF01652:Eukaryotic initiation factor 4E
Lachesis_group0__13_contigs__l.g7393	1222.99	445.2188	1.458525	0.004377	0.026243	3720	- && Q9P7X8.2 RecName: Full=Probable DNA-directed RNA polymerase I subunit RPA2; AltName: Full=DNA-directed RNA polymerase I polypeptide 2; Short=RNA polymerase I subunit 2 && PF06883:RNA polymerase I, Rpa2 specific domain|PF04563:RNA polymerase beta subunit|PF04565:RNA polymerase Rpb2, domain 3|PF00562:RNA polymerase Rpb2, domain 6|PF04561:RNA polymerase Rpb2, domain 2|PF04560:RNA polymerase Rpb2, domain 7
Lachesis_group0__13_contigs__l.g6296	548.5413	199.7772	1.457488	0.005963	0.032745	2349	- && P0CM79.1 RecName: Full=Polynucleotide 5'-hydroxyl-kinase GRC3 >P0CM78.1 RecName: Full=Polynucleotide 5'-hydroxyl-kinase GRC3 && PF16575:mRNA cleavage and polyadenylation factor CLP1 P-loop
Lachesis_group0__13_contigs__l.g815	1268.453	462.2841	1.4565	0.001516	0.01231	504	- && Q9CWZ3.3 RecName: Full=RNA-binding protein 8A; AltName: Full=RNA-binding motif protein 8A; AltName: Full=Ribonucleoprotein

							RBM8A && PF00076:RNA recognition motif. (a.k.a. RRM, RBD, or RNP domain)
Lachesis_group0__13_contigs__l.g7157	2438.335	888.6737	1.456068	0.003419	0.022031	1887	- && Q54BM8.1 RecName: Full=UPF0652 protein && PF09418:Protein of unknown function (DUF2009)
Lachesis_group0__13_contigs__l.g1351	1476.889	538.5739	1.455772	0.000541	0.005917	2631	- && Q5AP95.1 RecName: Full=Suppressor of ferric uptake 1 && PF08550:Fungal protein of unknown function (DUF1752)|PF00320:GATA zinc finger
Lachesis_group0__13_contigs__l.g8559	1754.102	639.6204	1.455656	0.002314	0.016722	756	- && Q9UUA4.1 RecName: Full=Translocation protein sec66 && PF09802:Preprotein translocase subunit Sec66
Lachesis_group0__13_contigs__l.g7059	741.8494	271.0971	1.452257	0.004153	0.025317	1581	- && O42997.1 RecName: Full=Uncharacterized protein C27B12.07 && PF07798:Protein of unknown function (DUF1640)
Lachesis_group0__13_contigs__l.g6964	546.4527	199.9445	1.451808	0.00668	0.035476	2184	- && Q9P7H1.1 RecName: Full=Tyrosine-protein phosphatase CDC14 homolog; AltName: Full=CDC fourteen-like phosphatase 1 && PF00782:Dual specificity phosphatase, catalytic domain|PF14671:Dual specificity protein phosphatase, N-terminal half
Lachesis_group0__13_contigs__l.g6505	245.4308	89.8684	1.450482	0.005786	0.03202	1197	- && Q10659.1 RecName: Full=Membrane glycoprotein spo14; AltName: Full=SEC12-like protein; AltName: Full=Sporulation-specific protein 14 && -
Lachesis_group0__13_contigs__l.g7406	471.4309	173.1382	1.445382	0.00243	0.017323	510	- && P59998.3 RecName: Full=Actin-related protein 2/3 complex subunit 4; AltName: Full=Arp2/3 complex 20 kDa subunit; Short=p20-ARC >P59999.3 RecName: Full=Actin-related protein 2/3 complex

							subunit 4; AltName: Full=Arp2/3 complex 20 kDa subunit; Short=p20-ARC >Q148J6.3 RecName: Full=Actin-related protein 2/3 complex subunit 4; AltName: Full=Arp2/3 complex 20 kDa subunit; Short=p20-ARC && PF05856:ARP2/3 complex 20 kDa subunit (ARPC4)
Lachesis_group0__13_contigs__l.g9005	1403.215	515.5304	1.445095	0.003232	0.021154	2283	- && O13775.1 RecName: Full=Probable glutamate--tRNA ligase, cytoplasmic; AltName: Full=Glutamyl-tRNA synthetase; Short=GluRS && PF14497:Glutathione S-transferase, C-terminal domain|PF00749:tRNA synthetases class I (E and Q), catalytic domain|PF03950:tRNA synthetases class I (E and Q), anti-codon binding domain
Lachesis_group0__13_contigs__l.g4703	1599.614	587.746	1.444459	0.001646	0.013017	1890	- && - && PF16561:Glycogen recognition site of AMP-activated protein kinase|PF04739:5'-AMP-activated protein kinase beta subunit, interaction domain
Lachesis_group0__13_contigs__l.g1001	485.8348	178.5527	1.444086	0.009504	0.045548	450	- && Q2K2Y1.1 RecName: Full=50S ribosomal protein L21 && PF00829:Ribosomal prokaryotic L21 protein
Lachesis_group0__13_contigs__l.g4622	1818.285	670.4227	1.439758	0.000945	0.008901	2739	- && P56286.1 RecName: Full=Eukaryotic translation initiation factor 2 subunit alpha; Short=eIF-2-alpha && PF00575:S1 RNA binding domain|PF07541:Eukaryotic translation initiation factor 2 alpha subunit
Lachesis_group0__13_contigs__l.g2481	909.4155	335.8319	1.437962	0.007103	0.037236	1515	- && Q5XJY5.2 RecName: Full=Coatomer subunit delta; AltName: Full=Archain; AltName: Full=Delta-coat protein; Short=Delta-COP &&

							PF00928:Adaptor complexes medium subunit family|PF01217:Clathrin adaptor complex small chain
Lachesis_group0__13_contigs__l.g8374	313.9949	116.2107	1.436696	0.010437	0.04864	3924	- && P36619.2 RecName: Full=Leptomycin B resistance protein pmd1 && PF00664:ABC transporter transmembrane region|PF00005:ABC transporter|PF07673:Protein of unknown function (DUF1602)
Lachesis_group0__13_contigs__l.g5552	5358.569	1980.25	1.436355	0.002913	0.01963	1521	- && O94260.1 RecName: Full=Putative G3BP-like protein && PF02136:Nuclear transport factor 2 (NTF2) domain|PF00076:RNA recognition motif. (a.k.a. RRM, RBD, or RNP domain)
Lachesis_group0__13_contigs__l.g6054	490.0532	180.9692	1.436274	0.006795	0.03593	1248	- && O74480.1 RecName: Full=Uncharacterized protein C1840.07c; Flags: Precursor && PF00149:Calcineurin-like phosphoesterase
Lachesis_group0__13_contigs__l.g3735	594.8803	219.8842	1.436124	0.001322	0.011204	1905	- && Q9UNZ2.2 RecName: Full=NSFL1 cofactor p47; AltName: Full=UBX domain-containing protein 2C; AltName: Full=p97 cofactor p47 >Q5RBG3.1 RecName: Full=NSFL1 cofactor p47; AltName: Full=p97 cofactor p47 && PF00789:UBX domain|PF08059:SEP domain
Lachesis_group0__13_contigs__l.g4462	178.3419	65.75095	1.436006	0.005673	0.031596	5823	- && Q3UMC0.2 RecName: Full=Spermatogenesis-associated protein 5; AltName: Full=Spermatogenesis-associated factor protein && PF00004:ATPase family associated with various cellular activities (AAA)
Lachesis_group0__13_contigs__l.g6629	990.7869	366.4815	1.435266	0.001973	0.01495	483	- && O74866.1 RecName: Full=Riboflavin kinase; AltName: Full=ATP:riboflavin 5'-

							phosphotransferase; AltName: Full=Flavin mononucleotide kinase 1; AltName: Full=Flavokinase && PF01687:Riboflavin kinase
Lachesis_group0__13_contigs__l.g5626	1825.809	676.1563	1.4331	0.008218	0.041303	2238	- && Q02799.1 RecName: Full=Zinc finger protein LEE1 && -
Lachesis_group0__13_contigs__l.g9537	563.6266	209.0341	1.431432	0.002379	0.017083	1092	- && O94505.1 RecName: Full=3'(2'),5'-bisphosphate nucleotidase; AltName: Full=3'(2'),5-bisphosphonucleoside 3'(2')-phosphohydrolase; AltName: Full=DPNPase; AltName: Full=Halotolerance protein tol1; AltName: Full=Target of lithium protein 1 && PF00459:Inositol monophosphatase family
Lachesis_group0__13_contigs__l.g8182	1174.739	435.5054	1.431377	0.00075	0.007473	2562	- && - && PF04082:Fungal specific transcription factor domain
Lachesis_group0__13_contigs__l.g11714	351.8659	130.6195	1.429952	0.008968	0.043868	567	- && - && PF09812:Mitochondrial ribosomal protein L28
Lachesis_group0__13_contigs__l.g5573	222.7955	82.83592	1.429228	0.005633	0.031467	1773	- && Q6BLF4.2 RecName: Full=Flap endonuclease 1; Short=FEN-1; AltName: Full=Flap structure-specific endonuclease 1 && PF00867:XPG I-region
Lachesis_group0__13_contigs__l.g2177	1890.993	703.2076	1.427243	0.000614	0.006441	3603	- && Q8RWR2.2 RecName: Full=La-related protein 1B; Short=AtLARP1b && PF05383:La domain
Lachesis_group0__13_contigs__l.g3921	390.4868	145.4134	1.427143	0.008122	0.041009	696	- && Q5M9I6.2 RecName: Full=Multiple myeloma tumor-associated protein 2 homolog && PF10159:Kinase phosphorylation protein
Lachesis_group0__13_contigs__l.g6946	267.8245	99.88813	1.424522	0.009894	0.046904	1797	- && - && PF09734:RNA polymerase III transcription factor (TF)IIIC subunit
Lachesis_group0__13_contigs__l.g3532	263.08	97.94775	1.424514	0.004399	0.026321	855	- && Q9US54.1 RecName: Full=Transcription initiation factor TFIID subunit 11; AltName:

							Full=TBP-associated factor 11 && PF04719:hTAFII28-like protein conserved region
Lachesis_group0__13_contigs__l.g6950	701.8531	262.0621	1.421486	0.002577	0.017985	1848	- && O94547.1 RecName: Full=Serine/threonine-protein kinase srk1; AltName: Full=Sty1-regulated kinase 1 && PF00069:Protein kinase domain
Lachesis_group0__13_contigs__l.g1374	10784.09	4029.091	1.420429	0.001496	0.012219	2376	- && P29547.2 RecName: Full=Elongation factor 1-gamma 1; Short=EF-1-gamma 1; AltName: Full=Calcium and membrane-binding protein 1; AltName: Full=Calcium phospholipid-binding protein; Short=CPBP; AltName: Full=Eukaryotic elongation factor 1Bgamma 1; Short=eEF1Bgamma 1; AltName: Full=Translation elongation factor 1B gamma 1 && PF02798:Glutathione S-transferase, N-terminal domain|PF00043:Glutathione S-transferase, C-terminal domain|PF00647:Elongation factor 1 gamma, conserved domain
Lachesis_group0__13_contigs__l.g1703	503.912	188.4505	1.420029	0.003634	0.02305	1122	- && Q01080.2 RecName: Full=DNA-directed RNA polymerase I subunit RPA49; Short=A49; AltName: Full=DNA-directed RNA polymerase I 49 kDa polypeptide && PF06870:A49-like RNA polymerase I associated factor
Lachesis_group0__13_contigs__l.g6693	643.0513	240.57	1.419637	0.00495	0.028708	3240	- && O74429.1 RecName: Full=Inositol hexakisphosphate and diphosphoinositol-pentakisphosphate kinase; AltName: Full=Cortical actin cytoskeleton protein asp1; AltName: Full=InsP6 and PP-IP5 kinase &&

							PF00328:Histidine phosphatase superfamily (branch 2)
Lachesis_group0__13_contigs__l.g2358	413.9114	154.5618	1.419499	0.005311	0.03018	2211	- && - && PF11696:Protein of unknown function (DUF3292)
Lachesis_group0__13_contigs__l.g7968	199.2697	74.57516	1.418954	0.00579	0.03202	1731	- && Q96MW5.2 RecName: Full=Conserved oligomeric Golgi complex subunit 8; Short=COG complex subunit 8; AltName: Full=Component of oligomeric Golgi complex 8 && PF04124:Dor1-like family
Lachesis_group0__13_contigs__l.g8796	7142.658	2672.372	1.418338	0.001773	0.013777	3939	- && Q7PPA5.5 RecName: Full=Calcium-transporting ATPase sarcoplasmic/endoplasmic reticulum type; AltName: Full=Calcium pump && PF00122:E1-E2 ATPase|PF00690:Cation transporter/ATPase, N-terminus|PF00689:Cation transporting ATPase, C-terminus|PF00702:haloacid dehalogenase-like hydrolase|PF00248:Aldo/keto reductase family
Lachesis_group0__13_contigs__l.g5974	822.047	308.3207	1.415338	0.001726	0.013497	3204	- && B0DAD3.1 RecName: Full=Exportin-T; AltName: Full=Exportin(tRNA); AltName: Full=Karyopherin-beta; AltName: Full=tRNA exportin && PF08389:Exportin 1-like protein
Lachesis_group0__13_contigs__l.g7941	1708.912	640.9697	1.414847	0.001902	0.014595	2829	- && P23955.2 RecName: Full=Mitochondrial-processing peptidase subunit alpha; AltName: Full=Alpha-MPP; Flags: Precursor && PF00675:Insulinase (Peptidase family M16)|PF05193:Peptidase M16 inactive domain
Lachesis_group0__13_contigs__l.g8480	1420.355	533.85	1.41203	0.003702	0.023299	3213	- && A8NEP3.2 RecName: Full=Tryptophan synthase && PF01008:Initiation factor 2 subunit

							family|PF00291:Pyridoxal-phosphate dependent enzyme|PF00290:Tryptophan synthase alpha chain
Lachesis_group0__13_contigs__l.g2330	859.5443	323.127	1.411731	0.004116	0.025204	1503	- && Q874L4.1 RecName: Full=Vitamin B6 transporter TPN1; AltName: Full=Transport of pyridoxine protein 1 && PF02133:Permease for cytosine/purines, uracil, thiamine, allantoin
Lachesis_group0__13_contigs__l.g9391	224.9884	84.65303	1.411413	0.007793	0.039821	1275	- && P0CO07.1 RecName: Full=Histone acetyltransferase type B catalytic subunit >P0CO06.1 RecName: Full=Histone acetyltransferase type B catalytic subunit && PF10394:Histone acetyl transferase HAT1 N-terminus
Lachesis_group0__13_contigs__l.g6435	1040.731	391.3687	1.411332	0.004134	0.025271	2898	- && P87177.1 RecName: Full=Uncharacterized WD repeat-containing protein C3D6.12 && PF00400:WD domain, G-beta repeat|PF04003:Dip2/Utp12 Family
Lachesis_group0__13_contigs__l.g5703	152.3942	57.57091	1.40802	0.008725	0.043156	1719	- && Q5F1R6.2 RecName: Full=DnaJ homolog subfamily C member 21; AltName: Full=DnaJ homolog subfamily A member 5; AltName: Full=Protein GS3 && PF00226:DnaJ domain|PF12171:Zinc-finger double-stranded RNA-binding
Lachesis_group0__13_contigs__l.g10437	374.4256	141.3179	1.407099	0.003227	0.021145	1173	- && - && PF00398:Ribosomal RNA adenine dimethylase
Lachesis_group0__13_contigs__l.g8053	530.4256	199.9908	1.407095	0.001597	0.012737	1485	- && A9IMS2.1 RecName: Full=30S ribosomal protein S15 && PF00312:Ribosomal protein S15
Lachesis_group0__13_contigs__l.g8795	3218.287	1213.849	1.406573	0.00098	0.009126	2028	- && Q2TZ06.1 RecName: Full=Protein sip5 && -
Lachesis_group0__13_contigs__l.g4275	360.164	135.7463	1.405733	0.006347	0.034271	492	- && - && PF09451:Autophagy-related protein 27

Lachesis_group0__13_contigs__l.g9033	175.5333	66.4116	1.404317	0.006395	0.03443	1290	- && O82244.1 RecName: Full=Dol-P-Man:Man(5)GlcNAc(2)-PP-Dol alpha-1,3-mannosyltransferase; AltName: Full=Alpha-1,3-mannosyltransferase ALG3; AltName: Full=Asparagine-linked glycosylation protein 3; Short=AtALG3; AltName: Full=Not56-like protein && PF05208:ALG3 protein
Lachesis_group0__13_contigs__l.g3649	486.9022	183.966	1.404217	0.00217	0.015959	1656	- && B2AFW1.1 RecName: Full=Probable Xaa-Pro aminopeptidase PEPP; AltName: Full=Aminoacylproline aminopeptidase; AltName: Full=Prolidase && PF00557:Metallopeptidase family M24|PF05195:Aminopeptidase P, N-terminal domain
Lachesis_group0__13_contigs__l.g8894	917.4035	346.7812	1.404161	0.007621	0.039165	2220	- && P17423.4 RecName: Full=Homoserine kinase; Short=HK; Short=HSK && PF04670:Gtr1/RagA G protein conserved region|PF00288:GHMP kinases N terminal domain
Lachesis_group0__13_contigs__l.g4870	382.3122	144.741	1.401927	0.002022	0.015225	2730	- && P78810.2 RecName: Full=Vacuolar transporter chaperone 4 && PF02656:Domain of unknown function (DUF202)|PF09359:VTC domain|PF03105:SPX domain
Lachesis_group0__13_contigs__l.g5369	1009.263	382.7828	1.399571	0.010363	0.048436	1095	- && O94671.1 RecName: Full=Probable homoserine dehydrogenase; Short=HDH && PF03447:Homoserine dehydrogenase, NAD binding domain|PF00742:Homoserine dehydrogenase

Lachesis_group0__13_contigs__l.g8083	954.4625	361.8891	1.398945	0.007472	0.038558	978	- && Q9UU99.1 RecName: Full=CRAL-TRIO domain-containing protein C23B6.04c && PF00650:CRAL/TRIO domain
Lachesis_group0__13_contigs__l.g6492	544.5803	206.5221	1.398427	0.006009	0.032965	1443	- && O94673.2 RecName: Full=Uncharacterized membrane protein C776.05 && PF10998:Protein of unknown function (DUF2838)
Lachesis_group0__13_contigs__l.g6314	10306.79	3915.128	1.396506	0.005	0.028905	1725	- && P27476.1 RecName: Full=Nuclear localization sequence-binding protein; AltName: Full=p67 && PF00076:RNA recognition motif. (a.k.a. RRM, RBD, or RNP domain)
Lachesis_group0__13_contigs__l.g4405	540.052	205.5696	1.394474	0.005706	0.03172	1095	- && O60122.1 RecName: Full=Anthranilate phosphoribosyltransferase && PF02885:Glycosyl transferase family, helical bundle domain|PF00591:Glycosyl transferase family, a/b domain
Lachesis_group0__13_contigs__l.g6002	951.4706	362.074	1.394396	0.001184	0.010357	2526	- && P87122.1 RecName: Full=Putative cation exchanger C3A12.06c && PF01699:Sodium/calcium exchanger protein
Lachesis_group0__13_contigs__l.g10688	1178.107	448.7507	1.392434	0.008965	0.043868	690	- && Q9C8J2.1 RecName: Full=NifU-like protein 5, mitochondrial; Short=AtNfu-I; Short=AtNfu5; Flags: Precursor && PF08712:Scaffold protein Nfu/NifU N terminal|PF01106:NifU-like domain
Lachesis_group0__13_contigs__l.g5681	989.5185	377.2632	1.391027	0.005575	0.031189	897	- && O14241.1 RecName: Full=Actin-related protein 2/3 complex subunit 2; AltName: Full=Arp2/3 complex 34 kDa subunit; Short=p34-ARC && PF04045:Arp2/3 complex, 34 kD subunit p34-Arc

Lachesis_group0__13_contigs__l.g5726	557.2454	212.7263	1.390232	0.002042	0.015335	3099	- && - && PF01494:FAD binding domain|PF04424:Protein of unknown function (DUF544)
Lachesis_group0__13_contigs__l.g10319	1332.126	509.7316	1.386114	0.000608	0.00639	684	- && Q09176.2 RecName: Full=Splicing factor U2AF 23 kDa subunit; AltName: Full=U2 auxiliary factor 23 kDa subunit; Short=U2AF23; AltName: Full=U2 snRNP auxiliary factor small subunit && PF00642:Zinc finger C-x8-C-x5-C-x3-H type (and similar)
Lachesis_group0__13_contigs__l.g11390	3831.644	1467.593	1.384566	0.002924	0.019647	2985	- && O74359.1 RecName: Full=Meiotically up-regulated gene 60 protein && PF00013:KH domain
Lachesis_group0__13_contigs__l.g6956	853.6714	327.2603	1.384136	0.007909	0.04021	1941	- && P40996.1 RecName: Full=Protein scd2/ral3 && PF00018:SH3 domain|PF00787:PX domain
Lachesis_group0__13_contigs__l.g7552	381.9483	146.5813	1.383526	0.005244	0.029891	1671	- && P32561.1 RecName: Full=Histone deacetylase RPD3; AltName: Full=Transcriptional regulatory protein RPD3 && PF00850:Histone deacetylase domain
Lachesis_group0__13_contigs__l.g11560	1192.248	457.9105	1.380583	0.001233	0.010666	2190	- && O74828.1 RecName: Full=Pre-rRNA-processing protein esf1 && PF08159:NUC153 domain
Lachesis_group0__13_contigs__l.g381	284.3605	109.3301	1.379488	0.002683	0.018535	936	- && Q96VP6.1 RecName: Full=Phosphoribosylaminoimidazole-succinocarboxamide synthase; AltName: Full=SAICAR synthetase && PF01259:SAICAR synthetase
Lachesis_group0__13_contigs__l.g3423	1631.756	627.6895	1.378638	0.004356	0.026179	1800	- && - && PF11735:Cryptococcal mannosyltransferase 1

Lachesis_group0__13_contigs__l.g4537	1002.351	385.7281	1.378105	0.002502	0.017661	3513	- && - && PF14523:Syntaxin-like protein|PF05739:SNARE domain
Lachesis_group0__13_contigs__l.g283	436.0728	168.0918	1.376899	0.004535	0.026902	954	- && Q5RFR8.1 RecName: Full=Coatomer subunit epsilon; AltName: Full=Epsilon-coat protein; Short=Epsilon-COP && PF04733:Coatomer epsilon subunit
Lachesis_group0__13_contigs__l.g11442	849.1652	327.2452	1.375734	0.00163	0.012911	1032	- && Q8TG13.1 RecName: Full=Casein kinase II subunit alpha; Short=CK II subunit alpha && PF00069:Protein kinase domain
Lachesis_group0__13_contigs__l.g7102	336.0858	129.9644	1.371609	0.001802	0.013975	471	- && P40046.3 RecName: Full=Vacuolar transporter chaperone 1; AltName: Full=Negative regulator of CDC42 protein 1; AltName: Full=Phosphate metabolism protein 4 && PF02656:Domain of unknown function (DUF202)
Lachesis_group0__13_contigs__l.g341	360.1876	139.5478	1.369445	0.005457	0.030786	1212	- && Q08641.3 RecName: Full=tRNA(Thr) (cytosine(32)-N(3))-methyltransferase; AltName: Full=Actin-binding protein of 140 kDa; AltName: Full=tRNA methyltransferase of 140 kDa && PF13489:Methyltransferase domain
Lachesis_group0__13_contigs__l.g456	2428.581	940.552	1.368791	0.002097	0.015603	1110	- && O59785.1 RecName: Full=Uncharacterized solute carrier family 35 member C320.08 && PF06027:Solute carrier family 35
Lachesis_group0__13_contigs__l.g2946	2895.796	1123.812	1.365809	0.005003	0.028907	4347	- && Q4P2B6.1 RecName: Full=Protein transport protein SEC31 && PF07304:Steroid receptor RNA activator (SRA1)
Lachesis_group0__13_contigs__l.g6447	760.3479	295.4538	1.36465	0.002501	0.017661	2241	- && Q9P787.1 RecName: Full=ER membrane protein complex subunit 3 && PF01956:Integral membrane protein DUF106

Lachesis_group0__13_contigs__l.g11156	2331.134	905.8833	1.363914	0.001625	0.012884	2838	- && Q6DFC6.1 RecName: Full=WD repeat-containing protein 75 && -
Lachesis_group0__13_contigs__l.g2879	2124.945	827.5176	1.360692	0.006554	0.034956	1704	- && O94512.1 RecName: Full=Oxysterol-binding protein-like protein 1 && PF01237:Oxysterol-binding protein
Lachesis_group0__13_contigs__l.g5614	809.8643	315.5126	1.360376	0.001574	0.012618	3336	- && - && PF00651:BTB/POZ domain
Lachesis_group0__13_contigs__l.g11379	1507.031	587.7316	1.358478	0.004102	0.025135	4032	- && Q6NZN0.2 RecName: Full=RNA-binding protein 26; AltName: Full=Protein expressed in male leptotene and zygotene spermatocytes 393; Short=MLZ-393; AltName: Full=RNA-binding motif protein 26 && PF01480:PWI domain|PF12171:Zinc-finger double-stranded RNA-binding|PF01585:G-patch domain|PF14259:RNA recognition motif (a.k.a. RRM, RBD, or RNP domain)
Lachesis_group0__13_contigs__l.g5866	187.9194	73.3099	1.357536	0.008886	0.043675	1068	- && - && PF07961:MBA1-like protein
Lachesis_group0__13_contigs__l.g9366	783.0663	305.8543	1.35716	0.004622	0.027331	879	- && - && PF11788:39S mitochondrial ribosomal protein L46
Lachesis_group0__13_contigs__l.g10822	11551.27	4518.132	1.3543	0.001478	0.012096	1983	- && P35190.1 RecName: Full=PHO85 cyclin CLG1; AltName: Full=Cyclin-like G1 protein 1 && PF08613:Cyclin
Lachesis_group0__13_contigs__l.g1546	731.7096	286.4812	1.353688	0.007547	0.038889	1779	- && Q55EC7.1 RecName: Full=RasGEF domain-containing serine/threonine-protein kinase X; AltName: Full=Ras guanine nucleotide exchange factor X; AltName: Full=RasGEF domain-containing protein X && PF00130:Phorbol esters/diacylglycerol binding domain (C1 domain)|PF07714:Protein tyrosine kinase

Lachesis_group0__13_contigs__l.g3643	817.4222	319.7963	1.353613	0.005225	0.029829	1389	- && P47154.1 RecName: Full=CAAX prenyl protease 1; AltName: Full=A-factor-converting enzyme; AltName: Full=Prenyl protein-specific endoprotease 1; Short=PPSEP 1 && PF16491:CAAX prenyl protease N-terminal, five membrane helices|PF01435:Peptidase family M48
Lachesis_group0__13_contigs__l.g3675	5197.201	2034.516	1.353197	0.003083	0.020446	3057	- && Q4P358.2 RecName: Full=Eukaryotic translation initiation factor 3 subunit A; Short=eIF3a; AltName: Full=Eukaryotic translation initiation factor 3 110 kDa subunit homolog; Short=eIF3 p110; AltName: Full=Translation initiation factor eIF3, p110 subunit homolog && -
Lachesis_group0__13_contigs__l.g6074	1592.541	624.0891	1.35181	0.009295	0.044899	2304	- && Q4PE39.1 RecName: Full=Protein transport protein SEC23 && PF04810:Sec23/Sec24 zinc finger|PF08033:Sec23/Sec24 beta-sandwich domain|PF00626:Gelsolin repeat|PF04811:Sec23/Sec24 trunk domain|PF04815:Sec23/Sec24 helical domain
Lachesis_group0__13_contigs__l.g11420	753.5268	295.6057	1.350303	0.004931	0.028645	681	- && Q5RKQ0.1 RecName: Full=Pre-mRNA-splicing factor SPF27; AltName: Full=Protein BCAS2 homolog && PF05700:Breast carcinoma amplified sequence 2 (BCAS2)
Lachesis_group0__13_contigs__l.g11643	394.0388	154.7557	1.348867	0.008622	0.042781	2010	- && Q8CI33.2 RecName: Full=CWF19-like protein 1 && PF13696:Zinc knuckle|PF04677:Protein similar to CwfJ C-terminus 1|PF04676:Protein similar to CwfJ C-terminus 2
Lachesis_group0__13_contigs__l.g5229	414.5989	163.2039	1.345097	0.007737	0.039667	975	- && Q9P7Z3.1 RecName: Full=Protein-lysine N-methyltransferase efm4; AltName: Full=Elongation

							factor methyltransferase 4; AltName: Full=Secretion and early endocytosis protein 1 homolog && PF12847:Methyltransferase domain
Lachesis_group0__13_contigs__l.g9201	231.2931	91.29068	1.343429	0.008955	0.043868	1194	- && - && PF02213:GYF domain
Lachesis_group0__13_contigs__l.g4653	363.9559	143.6005	1.343165	0.004178	0.025376	1362	- && O36018.1 RecName: Full=Protein bud22 && PF09073:BUD22
Lachesis_group0__13_contigs__l.g11538	861.0826	340.1081	1.340887	0.009545	0.045626	4530	- && Q9URX8.3 RecName: Full=Probable nucleoporin C890.06 && PF03177:Non-repetitive/WGA-negative nucleoporin C-terminal|PF08801:Nup133 N terminal like
Lachesis_group0__13_contigs__l.g3934	166.2078	65.7666	1.337696	0.007585	0.039013	1008	- && Q93K00.1 RecName: Full=Haloalkane dehalogenase >Q73Y99.1 RecName: Full=Haloalkane dehalogenase && PF00561:alpha/beta hydrolase fold
Lachesis_group0__13_contigs__l.g4895	573.4539	227.499	1.335121	0.005861	0.03233	1428	- && Q09747.1 RecName: Full=ATP-dependent RNA helicase dbp5 && PF00271:Helicase conserved C-terminal domain|PF00270:DEAD/DEAH box helicase
Lachesis_group0__13_contigs__l.g154	294.5747	116.7158	1.334745	0.005229	0.029829	2223	- && O74846.2 RecName: Full=Exocyst complex component sec6 && PF06046:Exocyst complex component Sec6
Lachesis_group0__13_contigs__l.g6632	432.1606	171.7234	1.332669	0.010249	0.048026	561	- && - && PF05620:Protein of unknown function (DUF788)
Lachesis_group0__13_contigs__l.g7902	401.8148	159.4821	1.332509	0.004867	0.028424	471	- && - && PF02136:Nuclear transport factor 2 (NTF2) domain
Lachesis_group0__13_contigs__l.g9111	743.3764	295.383	1.331102	0.005746	0.031875	891	- && Q5ZIM5.1 RecName: Full=Methionine aminopeptidase 1; Short=MAP 1; Short=MetAP 1;

							AltName: Full=Peptidase M 1 && PF00557:Metallopeptidase family M24
Lachesis_group0__13_contigs__l.g6373	591.1159	235.3266	1.330025	0.01038	0.048454	1047	- && O14295.1 RecName: Full=Pyridoxal reductase; Short=PL reductase; Short=PL-red && PF00248:Aldo/keto reductase family
Lachesis_group0__13_contigs__l.g2128	2139.155	851.9373	1.328372	0.002771	0.018953	1437	- && P0CO37.1 RecName: Full=Pre-mRNA-splicing factor ISY1 >P0CO36.1 RecName: Full=Pre-mRNA-splicing factor ISY1 && PF06246:Isy1-like splicing family
Lachesis_group0__13_contigs__l.g614	403.5583	160.7669	1.327324	0.003463	0.022215	1326	- && - && PF12937:F-box-like
Lachesis_group0__13_contigs__l.g4056	1246.27	497.1901	1.325974	0.002025	0.01524	939	- && - && PF08790:LYAR-type C2HC zinc finger
Lachesis_group0__13_contigs__l.g5714	1318.897	526.5298	1.325472	0.008885	0.043675	1650	- && Q9HGM9.1 RecName: Full=DnaJ homolog subfamily C member 7 homolog && PF00226:DnaJ domain|PF13414:TPR repeat
Lachesis_group0__13_contigs__l.g8057	4590.489	1836.979	1.321331	0.00232	0.016755	2493	- && J9VQ03.2 RecName: Full=Nucleolar GTP-binding protein 2 && PF01926:50S ribosome-binding GTPase|PF01778:Ribosomal L28e protein family|PF08153:NGP1NT (NUC091) domain
Lachesis_group0__13_contigs__l.g8818	737.0466	294.9099	1.321228	0.004067	0.024976	1068	- && Q9BTE7.1 RecName: Full=DCN1-like protein 5; AltName: Full=DCUN1 domain-containing protein 5; AltName: Full=Defective in cullin neddylation protein 1-like protein 5 && PF03556:Cullin binding
Lachesis_group0__13_contigs__l.g7697	4866.481	1950.967	1.318629	0.003747	0.023456	4458	- && P41820.1 RecName: Full=Brefeldin A resistance protein && PF14510:ABC-transporter extracellular N-terminal|PF00005:ABC transporter|PF06422:CDR ABC transporter|PF01061:ABC-2 type transporter

Lachesis_group0__13_contigs__l.g1561	511.2893	205.4037	1.317245	0.008182	0.041232	1740	- && - && PF08512:Histone chaperone Rttp106-like
Lachesis_group0__13_contigs__l.g11343	2549.717	1025.264	1.31435	0.002418	0.017292	852	- && Q5UNW1.1 RecName: Full=Uncharacterized protein R707 && PF01501:Glycosyl transferase family 8
Lachesis_group0__13_contigs__l.g7115	783.58	316.4892	1.308241	0.008755	0.043238	3408	- && - && PF00249:Myb-like DNA-binding domain
Lachesis_group0__13_contigs__l.g10205	3801.579	1541.949	1.302023	0.004633	0.027347	3258	- && O14134.1 RecName: Full=mRNA export factor elf1 && PF00385:Chromo (CHRromatin Organisation MOdifier) domain|PF00005:ABC transporter
Lachesis_group0__13_contigs__l.g1484	1095.904	444.7764	1.301547	0.003758	0.023501	2811	- && Q9USU2.1 RecName: Full=Dimethyladenosine transferase; AltName: Full=18S rRNA (adenine(1779)-N(6)/adenine(1780)-N(6))-dimethyltransferase; AltName: Full=18S rRNA dimethylase; AltName: Full=S-adenosylmethionine-6-N', N'-adenosyl(rRNA) dimethyltransferase && PF00398:Ribosomal RNA adenine dimethylase
Lachesis_group0__13_contigs__l.g11556	362.4929	146.9479	1.301489	0.007788	0.039816	2472	- && - && PF16589:BRCT domain, a BRCA1 C-terminus domain
Lachesis_group0__13_contigs__l.g2918	928.5543	377.2121	1.299934	0.007539	0.038888	3672	- && P22579.2 RecName: Full=Transcriptional regulatory protein SIN3 && PF16879:C-terminal domain of Sin3a protein|PF08295:Sin3 family co-repressor|PF02671:Paired amphipathic helix repeat
Lachesis_group0__13_contigs__l.g11711	725.5318	294.9137	1.298952	0.00991	0.046904	1611	- && P34078.2 RecName: Full=Protein LTV1; AltName: Full=Low-temperature viability protein 1 && PF04180:Low temperature viability protein
Lachesis_group0__13_contigs__l.g2997	251.6024	102.3956	1.298946	0.0073	0.037999	1302	- && A8NZM5.2 RecName: Full=WD repeat-containing protein JIP5 && -

Lachesis_group0__13_contigs__l.g4265	3458.014	1410.717	1.293666	0.003116	0.020551	3723	- && P35210.2 RecName: Full=Protein SPT23 && PF01833:IPT/TIG domain|PF12796:Ankyrin repeats (3 copies)
Lachesis_group0__13_contigs__l.g9657	210.0297	85.7778	1.293588	0.009931	0.046978	4182	- && P0CT89.1 RecName: Full=4-O-methyltransferase 1; Short=Mtrase 1 && PF00481:Protein phosphatase 2C|PF00891:O-methyltransferase
Lachesis_group0__13_contigs__l.g2942	391.4077	159.6765	1.292324	0.009799	0.046639	471	- && O23310.1 RecName: Full=Nuclear transcription factor Y subunit B-3; Short=AtNF-YB-3; AltName: Full=Transcriptional activator HAP3C && PF00808:Histone-like transcription factor (CBF/NF-Y) and archaeal histone
Lachesis_group0__13_contigs__l.g7227	505.6776	206.9134	1.290346	0.006206	0.033729	1647	- && P0CR02.1 RecName: Full=ATP-dependent RNA helicase DBP8 && PF00270:DEAD/DEAH box helicase|PF00271:Helicase conserved C-terminal domain
Lachesis_group0__13_contigs__l.g5716	600.7409	245.987	1.289374	0.006609	0.035167	807	- && Q8TG11.1 RecName: Full=Casein kinase II subunit beta-2; Short=CK II beta-2 && PF01214:Casein kinase II regulatory subunit
Lachesis_group0__13_contigs__l.g11536	500.5176	205.0108	1.288976	0.008209	0.041293	1548	- && O13681.2 RecName: Full=Integral inner nuclear membrane protein ima1 && PF09779:Ima1 N-terminal domain
Lachesis_group0__13_contigs__l.g5802	6183.914	2530.921	1.288956	0.007391	0.038268	1218	- && P78827.2 RecName: Full=Probable ketol-acid reductoisomerase, mitochondrial; AltName: Full=Acetohydroxy-acid reductoisomerase; AltName: Full=Alpha-keto-beta-hydroxylacyl reductoisomerase; Flags: Precursor && PF07991:Acetohydroxy acid isomeroreductase,

							NADPH-binding domain|PF01450:Acetohydroxy acid isomeroreductase, catalytic domain
Lachesis_group0__13_contigs__l.g10015	181.8455	74.54097	1.288234	0.006585	0.035079	1134	- && Q54WW4.1 RecName: Full=Putative isoaspartyl peptidase/L-asparaginase; AltName: Full=Beta-aspartyl-peptidase; AltName: Full=Isoaspartyl dipeptidase; AltName: Full=L-asparagine amidohydrolase; Contains: RecName: Full=Putative isoaspartyl peptidase/L-asparaginase alpha chain; Contains: RecName: Full=Putative isoaspartyl peptidase/L-asparaginase beta chain; Flags: Precursor && PF01112:Asparaginase
Lachesis_group0__13_contigs__l.g7293	1319.386	540.3149	1.28816	0.003188	0.02093	1956	- && Q9USP8.2 RecName: Full=Isocitrate dehydrogenase [NAD] subunit 2, mitochondrial; AltName: Full=Isocitric dehydrogenase; AltName: Full=NAD(+)-specific ICDH; Flags: Precursor && PF03357:Snf7|PF00180:Isocitrate/isopropylmalate dehydrogenase
Lachesis_group0__13_contigs__l.g5846	1007.195	412.8948	1.287193	0.00842	0.042102	1311	- && Q10234.1 RecName: Full=Probable 37S ribosomal protein S5, mitochondrial && PF00333:Ribosomal protein S5, N-terminal domain|PF03719:Ribosomal protein S5, C-terminal domain
Lachesis_group0__13_contigs__l.g11314	325.7577	133.6241	1.286101	0.008205	0.041292	1281	- && Q7XUF4.2 RecName: Full=Cyclin-dependent kinase G-2; Short=CDKG;2 >A2XUW1.1 RecName: Full=Cyclin-dependent kinase G-2; Short=CDKG;2 && PF00069:Protein kinase domain
Lachesis_group0__13_contigs__l.g4714	202.2851	82.89329	1.285663	0.008559	0.042587	1575	- && Q86XW9.1 RecName: Full=Thioredoxin domain-containing protein 6; AltName:

							Full=Thioredoxin-like protein 2; Short=Txl-2 && PF00334:Nucleoside diphosphate kinase
Lachesis_group0__13_contigs__l.g11127	1183.514	486.5131	1.282635	0.007122	0.037281	663	- && Q6AXT8.1 RecName: Full=Splicing factor 3A subunit 2 && PF12874:Zinc-finger of C2H2 type|PF16835:Pre-mRNA-splicing factor SF3a complex subunit 2 (Prp11)
Lachesis_group0__13_contigs__l.g7292	4151.705	1708.09	1.281235	0.002379	0.017083	4416	- && Q5AK62.1 RecName: Full=Virulence protein SSD1 && PF00773:RNB domain
Lachesis_group0__13_contigs__l.g4823	666.0084	274.3788	1.2804	0.006275	0.033969	1032	- && Q12000.2 RecName: Full=Translation machinery-associated protein 46; AltName: Full=DRG family-regulatory protein 1 && PF16543:DRG Family Regulatory Proteins, Tma46|PF00642:Zinc finger C-x8-C-x5-C-x3-H type (and similar)
Lachesis_group0__13_contigs__l.g7248	1824.74	751.6193	1.279888	0.003401	0.021953	1542	- && Q09100.2 RecName: Full=RNA-binding protein rnp24 && PF14259:RNA recognition motif (a.k.a. RRM, RBD, or RNP domain)
Lachesis_group0__13_contigs__l.g6099	964.0793	397.2718	1.279571	0.006443	0.034563	2862	- && Q52KI8.2 RecName: Full=Serine/arginine repetitive matrix protein 1; AltName: Full=Plenty-of-prolines 101 && PF03443:Glycosyl hydrolase family 61|PF00734:Fungal cellulose binding domain|PF01480:PWI domain
Lachesis_group0__13_contigs__l.g9189	1783.076	734.6053	1.279288	0.009206	0.044634	4656	- && Q92373.1 RecName: Full=Replication factor A protein 2; AltName: Full=Single-stranded DNA-binding protein P30 subunit && PF09797:N-acetyltransferase B complex (NatB) non catalytic subunit|PF08784:Replication protein A C terminal

Lachesis_group0__13_contigs__l.g7070	296.5335	122.2508	1.279195	0.00535	0.030339	1314	- && Q4P2E8.1 RecName: Full=Actin-related protein 4; AltName: Full=Actin-like protein ARP4; Short=Actin-like protein 4 && PF00022:Actin
Lachesis_group0__13_contigs__l.g6030	1980.194	816.8565	1.277297	0.006448	0.034563	1266	- && O60158.2 RecName: Full=Bouquet formation protein 4 && -
Lachesis_group0__13_contigs__l.g929	287.2331	118.7158	1.276203	0.007195	0.037588	708	- && - && PF13414:TPR repeat
Lachesis_group0__13_contigs__l.g1509	6303.114	2603.762	1.275408	0.004129	0.025256	3957	- && Q90YK3.1 RecName: Full=L-gulonolactone oxidase; Short=LGO; AltName: Full=L-gulono-gamma-lactone oxidase; Short=GLO && PF04030:D-arabinono-1,4-lactone oxidase|PF01565:FAD binding domain
Lachesis_group0__13_contigs__l.g9479	443.5792	183.5248	1.27442	0.005278	0.030054	2001	- && P42698.2 RecName: Full=DNA-damage-repair/toleration protein DRT111, chloroplastic; AltName: Full=REQUIRED FOR SNC4-1D protein 2; Flags: Precursor && PF01585:G-patch domain
Lachesis_group0__13_contigs__l.g442	1500.765	622.2336	1.270318	0.002575	0.017985	1125	- && P46794.2 RecName: Full=Cystathionine beta-synthase; AltName: Full=Beta-thionase; AltName: Full=Serine sulfhydrase && PF00291:Pyridoxal-phosphate dependent enzyme
Lachesis_group0__13_contigs__l.g6034	1417.544	588.989	1.266993	0.00246	0.017484	1566	- && B6H9U8.1 RecName: Full=Secondary metabolism regulator laeA; AltName: Full=Methyltransferase laeA; AltName: Full=Velvet complex subunit laeA && PF12847:Methyltransferase domain
Lachesis_group0__13_contigs__l.g4804	414.7341	172.5284	1.266421	0.010344	0.048392	843	- && P22143.1 RecName: Full=Cyanamide hydratase; AltName: Full=Urea hydro-lyase && PF01966:HD domain

Lachesis_group0__13_contigs__l.g10136	365.5546	152.431	1.263756	0.009476	0.045497	1980	- && Q8WZM0.1 RecName: Full=Histone acetyltransferase GCN5 && PF00439:Bromodomain
Lachesis_group0__13_contigs__l.g5936	2871.471	1199.451	1.259472	0.009426	0.045354	3354	- && P0CQ17.1 RecName: Full=Lon protease homolog, mitochondrial; Flags: Precursor && PF05362:Lon protease (S16) C-terminal proteolytic domain|PF02190:ATP-dependent protease La (LON) substrate-binding domain|PF00004:ATPase family associated with various cellular activities (AAA)
Lachesis_group0__13_contigs__l.g10030	1484.406	620.6555	1.258028	0.002273	0.01651	3561	- && P07527.1 RecName: Full=Mitosis inhibitor protein kinase wee1; AltName: Full=P107 protein kinase homolog && PF00069:Protein kinase domain
Lachesis_group0__13_contigs__l.g4840	694.8952	290.8347	1.25709	0.005554	0.031189	1068	- && Q8FKG1.1 RecName: Full=S-(hydroxymethyl)glutathione dehydrogenase; AltName: Full=Alcohol dehydrogenase class-3; AltName: Full=Alcohol dehydrogenase class-III; AltName: Full=Glutathione-dependent formaldehyde dehydrogenase; Short=FALDH; Short=FDH; Short=GSH-FDH && PF00107:Zinc-binding dehydrogenase|PF08240:Alcohol dehydrogenase GroES-like domain
Lachesis_group0__13_contigs__l.g5520	1456.548	609.1912	1.257063	0.009024	0.044017	2559	- && G3XMC9.1 RecName: Full=Efflux pump azaL; AltName: Full=Azaphilone biosynthesis cluster protein azaL && PF07690:Major Facilitator Superfamily
Lachesis_group0__13_contigs__l.g9032	530.9165	222.2539	1.255626	0.00346	0.022215	684	- && - && PF04750:FAR-17a/AIG1-like protein

Lachesis_group0__13_contigs__l.g10345	7505.918	3144.762	1.255143	0.005432	0.030694	2631	- && Q4P0P0.1 RecName: Full=Eukaryotic translation initiation factor 3 subunit C; Short=eIF3c; AltName: Full=Eukaryotic translation initiation factor 3 93 kDa subunit homolog; Short=eIF3 p93; AltName: Full=Translation initiation factor eIF3, p93 subunit homolog && PF01399:PCI domain|PF05470:Eukaryotic translation initiation factor 3 subunit 8 N-terminus
Lachesis_group0__13_contigs__l.g11275	1206.869	506.5056	1.252421	0.009228	0.044634	1038	- && Q6DHC3.1 RecName: Full=Solute carrier family 25 member 40 && PF00153:Mitochondrial carrier protein
Lachesis_group0__13_contigs__l.g5574	1329.938	559.1093	1.25035	0.008494	0.042379	3603	- && P53621.2 RecName: Full=Coatomer subunit alpha; AltName: Full=Alpha-coat protein; Short=Alpha-COP; AltName: Full=HEP-COP; Short=HEPCOP; Contains: RecName: Full=Xenin; AltName: Full=Xenopsin-related peptide; Contains: RecName: Full=Proxenin && PF04053:Coatomer WD associated region|PF00400:WD domain, G-beta repeat|PF06957:Coatomer (COPI) alpha subunit C-terminus
Lachesis_group0__13_contigs__l.g8806	18255.56	7673.926	1.250348	0.010373	0.048444	2385	- && Q10284.2 RecName: Full=Heat shock protein sks2; AltName: Full=Heat shock cognate protein hsc1 && PF08286:Spc24 subunit of Ndc80|PF00012:Hsp70 protein|PF10712:NAD-specific glutamate dehydrogenase

Lachesis_group0__13_contigs__l.g1284	1280.486	538.7214	1.248721	0.006053	0.033109	768	- && Q4PHE4.1 RecName: Full=Histone H2A.Z && PF16211:C-terminus of histone H2A|PF00125:Core histone H2A/H2B/H3/H4
Lachesis_group0__13_contigs__l.g172	331.3675	139.7971	1.244916	0.005567	0.031189	2793	- && - && PF04082:Fungal specific transcription factor domain
Lachesis_group0__13_contigs__l.g2477	679.611	288.4727	1.235818	0.004863	0.028413	4005	- && D7PI00.1 RecName: Full=Transcription factor vrtR1; AltName: Full=Viridicatumtoxin synthesis protein R1 && PF04082:Fungal specific transcription factor domain
Lachesis_group0__13_contigs__l.g5380	760.1328	323.1382	1.234313	0.004166	0.025344	1911	- && Q6PD26.3 RecName: Full=GPI transamidase component PIG-S; AltName: Full=Phosphatidylinositol-glycan biosynthesis class S protein && PF10510:Phosphatidylinositol-glycan biosynthesis class S protein
Lachesis_group0__13_contigs__l.g1758	660.2894	280.8307	1.232745	0.008976	0.043868	1032	- && Q09923.1 RecName: Full=Aldo-keto reductase yakc [NADP(+)] && PF00248:Aldo/keto reductase family
Lachesis_group0__13_contigs__l.g9043	761.1279	324.3585	1.230694	0.007783	0.039807	1659	- && Q9UTB4.1 RecName: Full=Protein mss51 && PF13824:Zinc-finger of mitochondrial splicing suppressor 51
Lachesis_group0__13_contigs__l.g3987	8607.012	3671.194	1.229254	0.006018	0.032985	987	- && - && PF07716:Basic region leucine zipper
Lachesis_group0__13_contigs__l.g649	1109.751	474.3147	1.226964	0.006407	0.034442	1986	- && P10961.1 RecName: Full=Heat shock factor protein; Short=HSF; AltName: Full=Heat shock transcription factor; Short=HSTF && PF00447:HSF-type DNA-binding
Lachesis_group0__13_contigs__l.g952	755.6612	322.6724	1.226819	0.005707	0.03172	2928	- && Q3ZLR7.2 RecName: Full=Transcription factor SPT20 homolog-like 1 && PF12090:Spt20 family

Lachesis_group0__13_contigs__l.g11446	5447.182	2340.643	1.218672	0.004507	0.026781	2397	- && P26651.1 RecName: Full=mRNA decay activator protein ZFP36; AltName: Full=G0/G1 switch regulatory protein 24; AltName: Full=Growth factor-inducible nuclear protein NUP475; AltName: Full=Tristetraprolin; AltName: Full=Zinc finger protein 36; Short=Zfp-36 && PF00642:Zinc finger C-x8-C-x5-C-x3-H type (and similar)
Lachesis_group0__13_contigs__l.g8611	936.2173	402.7384	1.217379	0.006452	0.034567	495	- && P53937.1 RecName: Full=37S ribosomal protein SWS2, mitochondrial; AltName: Full=Sick without securin protein 2 && PF00416:Ribosomal protein S13/S18
Lachesis_group0__13_contigs__l.g7496	1632.119	704.1209	1.213108	0.009321	0.044968	1458	- && - && PF09350:Domain of unknown function (DUF1992)
Lachesis_group0__13_contigs__l.g5848	4158.701	1796.159	1.211172	0.004352	0.026166	1026	- && Q3E6T0.1 RecName: Full=Probable sugar phosphate/phosphate translocator At5g25400 && PF03151:Triose-phosphate Transporter family
Lachesis_group0__13_contigs__l.g9217	3832.553	1665.911	1.202161	0.009455	0.045433	3567	- && Q5KPJ5.1 RecName: Full=Acetolactate synthase, mitochondrial; AltName: Full=AHAS; AltName: Full=ALS; AltName: Full=Acetohydroxy-acid synthase; Flags: Precursor && PF02153:Prephenate dehydrogenase|PF02776:Thiamine pyrophosphate enzyme, N-terminal TPP binding domain|PF00205:Thiamine pyrophosphate enzyme, central domain|PF02775:Thiamine pyrophosphate enzyme, C-terminal TPP binding domain

Lachesis_group0__13_contigs__l.g7152	1949.243	851.2817	1.195099	0.009693	0.04623	720	- && O42901.3 RecName: Full=Uncharacterized protein C119.09c && PF04061:ORMDL family
Lachesis_group0__13_contigs__l.g7095	2441.035	1066.895	1.194195	0.005771	0.031968	4932	- && A8NWR2.2 RecName: Full=Ribosome biogenesis protein ERB1; AltName: Full=Eukaryotic ribosome biogenesis protein 1 && PF08145:BOP1NT (NUC169) domain
Lachesis_group0__13_contigs__l.g3770	1423.937	624.7756	1.188443	0.009907	0.046904	2328	- && A8P1W0.1 RecName: Full=Elongation factor G, mitochondrial; Short=EF-Gmt; AltName: Full=Elongation factor G 1, mitochondrial; Short=mEF-G 1; AltName: Full=Elongation factor G1; Flags: Precursor && PF03144:Elongation factor Tu domain 2|PF03764:Elongation factor G, domain IV|PF00009:Elongation factor Tu GTP binding domain|PF00679:Elongation factor G C-terminus|PF14492:Elongation Factor G, domain II
Lachesis_group0__13_contigs__l.g6232	337.6014	148.5303	1.186398	0.009482	0.0455	6039	- && - && PF11707:Ribosome 60S biogenesis N-terminal|PF16201:Nucleolar pre-ribosomal-associated protein 1
Lachesis_group0__13_contigs__l.g6407	3213.182	1413.093	1.185416	0.010134	0.047671	1485	- && P49605.2 RecName: Full=cAMP-dependent protein kinase regulatory subunit; Short=PKA regulatory subunit && PF02197:Regulatory subunit of type II PKA R-subunit|PF00027:Cyclic nucleotide-binding domain
Lachesis_group0__13_contigs__l.g5167	1601.921	704.7741	1.184887	0.009452	0.045433	3771	- && Q1K8G0.1 RecName: Full=Cystathionine beta-lyase && PF01053:Cys/Met metabolism PLP-dependent enzyme|PF00288:GHMP kinases N terminal domain

Lachesis_group0__13_contigs__l.g6530	752.587	331.72	1.182165	0.003967	0.024486	1506	- && B0CXQ2.1 RecName: Full=Velvet complex subunit B && PF11754:Velvet factor
Lachesis_group0__13_contigs__l.g11291	2578.652	1139.366	1.178291	0.003445	0.022173	1440	- && C4YR54.1 RecName: Full=G1/S-specific cyclin CCN1 >P0CY18.1 RecName: Full=G1/S-specific cyclin CLN1 && PF00134:Cyclin, N-terminal domain|PF02984:Cyclin, C-terminal domain
Lachesis_group0__13_contigs__l.g5233	884.2545	391.2824	1.176272	0.006484	0.034697	3033	- && Q09825.1 RecName: Full=Spindle pole body-associated protein sad1 && PF07738:Sad1 / UNC-like C-terminal
Lachesis_group0__13_contigs__l.g11049	14464.23	6402.931	1.175674	0.005077	0.029215	4146	- && Q9HGM3.1 RecName: Full=Mitochondrial respiratory chain complexes assembly protein rca1 && PF01434:Peptidase family M41|PF00004:ATPase family associated with various cellular activities (AAA)|PF00328:Histidine phosphatase superfamily (branch 2)|PF06480:FtsH Extracellular
Lachesis_group0__13_contigs__l.g5900	3725.08	1660.585	1.165589	0.005289	0.030102	2928	- && Q03516.1 RecName: Full=Uncharacterized protein RSN1; AltName: Full=Rescuer of SRO7 at high Nacl protein 1 && PF02714:Calcium-dependent channel, 7TM region, putative phosphate|PF13967:Late exocytosis, associated with Golgi transport|PF12621:Extracellular tail, of 10TM putative phosphate transporter|PF14703:Cytosolic domain of 10TM putative phosphate transporter
Lachesis_group0__13_contigs__l.g1162	428.1837	190.989	1.164346	0.006356	0.034286	4830	- && Q9UTL9.1 RecName: Full=Uncharacterized ATP-dependent helicase C144.05 && PF00176:SNF2 family N-terminal

								domain|PF13920:Zinc finger, C3HC4 type (RING finger)
Lachesis_group0__13_contigs__l.g6229	1095.663	489.9244	1.161528		0.007754	0.039697	3714	- && O14306.2 RecName: Full=Probable inositol polyphosphate 5-phosphatase C9G1.10c && PF03372:Endonuclease/Exonuclease/phosphatase family
Lachesis_group0__13_contigs__l.g2914	380.772	170.3286	1.160841		0.008105	0.040977	942	- && Q9RA05.1 RecName: Full=(-)-trans-carveol dehydrogenase; AltName: Full=(4R,6S)-carveol dehydrogenase; Short=CDH; Short=Carveol dehydrogenase && PF00106:short chain dehydrogenase
Lachesis_group0__13_contigs__l.g2245	1379.396	618.8326	1.156556		0.008114	0.041	669	- && Q08971.1 RecName: Full=Protein PBDC1 homolog && PF04669:Polysaccharide biosynthesis
Lachesis_group0__13_contigs__l.g1579	7584.908	3408.159	1.154214		0.010029	0.047319	486	- && O94083.1 RecName: Full=Eukaryotic translation initiation factor 5A; Short=eIF-5A; AltName: Full=eIF-4D && PF01287:Eukaryotic elongation factor 5A hypusine, DNA-binding OB fold
Lachesis_group0__13_contigs__l.g10985	2583.272	1174.538	1.137275		0.008977	0.043868	603	- && Q9P720.1 RecName: Full=60S ribosomal protein L16; AltName: Full=Cytoplasmic ribosomal protein 46 && PF00572:Ribosomal protein L13
Lachesis_group0__13_contigs__l.g8860	2189.901	1003.017	1.126641		0.01017	0.047778	1764	- && P0CR57.1 RecName: Full=Pre-mRNA-processing protein 45 && PF02731:SKIP/SNW domain
Lachesis_group0__13_contigs__l.g5528	2758.601	1273.448	1.115378		0.006258	0.033923	2847	- && Q4P9K4.1 RecName: Full=Protein transport protein SEC24 && PF04810:Sec23/Sec24 zinc finger|PF00626:Gelsolin repeat|PF04811:Sec23/Sec24 trunk

							domain|PF00397:WW domain|PF04815:Sec23/Sec24 helical domain|PF08033:Sec23/Sec24 beta-sandwich domain
Lachesis_group0__13_contigs__l.g10253	1175.637	543.418	1.113552	0.007359	0.038137	3396	- && O60111.2 RecName: Full=ABC1 family protein MCP2 homolog; Flags: Precursor && PF03109:ABC1 family|PF00566:Rab-GTPase-TBC domain
Lachesis_group0__13_contigs__l.g243	2684.74	1240.949	1.113417	0.005517	0.030999	546	- && P34737.1 RecName: Full=40S ribosomal protein S15; AltName: Full=S12 && PF00203:Ribosomal protein S19
Lachesis_group0__13_contigs__l.g4547	399.5754	184.8637	1.112303	0.008839	0.043564	1866	- && Q9HGM6.1 RecName: Full=Putative transporter C543.05c && PF00955:HCO3-transporter family
Lachesis_group0__13_contigs__l.g5546	1315.197	613.9041	1.099109	0.009356	0.045097	3516	- && Q10097.1 RecName: Full=Uncharacterized transporter C11D3.18C && PF00076:RNA recognition motif. (a.k.a. RRM, RBD, or RNP domain)|PF07690:Major Facilitator Superfamily
Lachesis_group0__13_contigs__l.g2552	354.8182	167.2204	1.085015	0.0106	0.049229	1320	- && Q8VYR0.1 RecName: Full=Calcium uniporter protein 5, mitochondrial; Flags: Precursor && PF04678:Mitochondrial calcium uniporter
Lachesis_group0__13_contigs__l.g4085	1791.793	850.5205	1.075186	0.008718	0.043156	2712	- && P04786.2 RecName: Full=DNA topoisomerase 1; AltName: Full=DNA topoisomerase I; AltName: Full=Maintenance of killer protein 1 && PF01028:Eukaryotic DNA topoisomerase I, catalytic core|PF02919:Eukaryotic DNA topoisomerase I, DNA binding

							fragment|PF14370:C-terminal topoisomerase domain
Lachesis_group0__13_contigs__l.g9247	3942.627	1873.574	1.073353	0.009798	0.046639	1197	- && Q10489.1 RecName: Full=Pyruvate dehydrogenase E1 component subunit alpha, mitochondrial; Short=PDHE1-A; Flags: Precursor && PF00676:Dehydrogenase E1 component
Lachesis_group0__13_contigs__l.g9001	10445.46	5040.054	1.05138	0.007546	0.038889	1695	- && Q9C0U9.1 RecName: Full=Uncharacterized transporter PB1C11.03 && PF07690:Major Facilitator Superfamily
Lachesis_group0__13_contigs__l.g4676	1368.678	2905.35	-1.08598	0.005892	0.032456	1284	- && P24280.3 RecName: Full=SEC14 cytosolic factor; AltName: Full=Phosphatidylinositol/phosphatidylcholine transfer protein; Short=PI/PC TP && PF03765:CRAL/TRIO, N-terminal domain|PF00650:CRAL/TRIO domain
Lachesis_group0__13_contigs__l.g8949	5375.228	11449.27	-1.09088	0.010142	0.047691	3288	- && O75003.1 RecName: Full=Trehalose phosphorylase; AltName: Full=Trehalose synthase; Short=TSase; Flags: Precursor && PF00534:Glycosyl transferases group 1
Lachesis_group0__13_contigs__l.g9368	3320.763	7123.435	-1.10109	0.010471	0.048753	3288	- && P13860.1 RecName: Full=Exoglucanase 1; AltName: Full=1,4-beta-cellobiohydrolase; AltName: Full=Exocellobiohydrolase I; AltName: Full=Exoglucanase I; Flags: Precursor && PF00840:Glycosyl hydrolase family 7|PF00734:Fungal cellulose binding domain
Lachesis_group0__13_contigs__l.g6742	578.9254	1257.214	-1.11891	0.007916	0.040225	798	- && O14351.1 RecName: Full=Uncharacterized oxidoreductase C30D10.05c && PF00106:short chain dehydrogenase

Lachesis_group0__13_contigs__l.g9432	7572.525	16491.88	-1.12293	0.007754	0.039697	1062	- && O06179.1 RecName: Full=Putative monooxygenase Rv1533 && PF03060:Nitronate monooxygenase
Lachesis_group0__13_contigs__l.g678	2057.14	4492.705	-1.12698	0.005443	0.030742	1725	- && P15245.3 RecName: Full=Phenol 2-monooxygenase; AltName: Full=Phenol hydroxylase && PF07976:Phenol hydroxylase, C-terminal dimerisation domain|PF01494:FAD binding domain
Lachesis_group0__13_contigs__l.g954	142.9647	313.2921	-1.13207	0.009058	0.044086	750	- && B8M9K0.1 RecName: Full=Hydrolase tropI; AltName: Full=Tropolone synthesis protein I && PF01738:Dienelactone hydrolase family
Lachesis_group0__13_contigs__l.g2601	2188.881	4805.585	-1.13457	0.010352	0.048406	1920	- && Q9UTJ7.1 RecName: Full=Probable succinate dehydrogenase [ubiquinone] flavoprotein subunit, mitochondrial; AltName: Full=Flavoprotein subunit of complex II; Short=FP; Flags: Precursor && PF00890:FAD binding domain|PF02910:Fumarate reductase flavoprotein C-term
Lachesis_group0__13_contigs__l.g4251	4026.553	8847.143	-1.13565	0.007699	0.039529	2322	- && - && PF10497:Zinc-finger domain of monoamine-oxidase A repressor R1
Lachesis_group0__13_contigs__l.g2242	834.0684	1837.064	-1.13934	0.004904	0.028534	4188	- && O80327.1 RecName: Full=Thaumatin-like protein 1; Flags: Precursor && PF00314:Thaumatin family|PF08386:TAP-like protein|PF00561:alpha/beta hydrolase fold
Lachesis_group0__13_contigs__l.g5700	105.7635	234.0366	-1.1454	0.009454	0.045433	906	- && Q0IHF9.2 RecName: Full=Store-operated calcium entry-associated regulatory factor; Short=SARAF; Short=SOCE-associated regulatory factor; AltName: Full=Transmembrane protein 66;

							Flags: Precursor && PF06682:SOCE-associated regulatory factor of calcium homoeostasis
Lachesis_group0__13_contigs__l.g6553	417.6558	929.8012	-1.15494	0.006111	0.033324	10668	- && P38811.1 RecName: Full=Transcription-associated protein 1; AltName: Full=p400 kDa component of SAGA && PF02259:FAT domain|PF00454:Phosphatidylinositol 3- and 4-kinase
Lachesis_group0__13_contigs__l.g2842	1672.627	3736.902	-1.1598	0.008065	0.040843	1167	- && Q9Y7N4.1 RecName: Full=D-amino-acid oxidase; Short=DAAO; Short=DAMOX; Short=DAO && PF01266:FAD dependent oxidoreductase
Lachesis_group0__13_contigs__l.g8786	469.6773	1051.332	-1.16235	0.009851	0.046825	1053	- && O42909.1 RecName: Full=Zinc-type alcohol dehydrogenase-like protein C16A3.02c && PF13602:Zinc-binding dehydrogenase
Lachesis_group0__13_contigs__l.g7505	265.2037	599.4959	-1.17711	0.005061	0.02915	831	- && - && PF00651:BTB/POZ domain
Lachesis_group0__13_contigs__l.g1641	58498.23	132501.1	-1.17954	0.003525	0.022533	1311	- && Q01982.1 RecName: Full=Glyceraldehyde-3-phosphate dehydrogenase; Short=GAPDH && PF02800:Glyceraldehyde 3-phosphate dehydrogenase, C-terminal domain|PF00044:Glyceraldehyde 3-phosphate dehydrogenase, NAD binding domain
Lachesis_group0__13_contigs__l.g6803	134.7169	306.4316	-1.18621	0.00641	0.034442	933	- && Q9JLZ3.1 RecName: Full=Methylglutaconyl-CoA hydratase, mitochondrial; AltName: Full=AU-specific RNA-binding enoyl-CoA hydratase; Short=AU-binding enoyl-CoA hydratase; Short=muAUH; Flags: Precursor && PF00378:Enoyl-CoA hydratase/isomerase

Lachesis_group0__13_contigs__l.g6734	3108.621	7083.352	-1.18814	0.005226	0.029829	1275	- && O59735.2 RecName: Full=Sphingosine N-acyltransferase lac1; AltName: Full=Meiotically up-regulated gene 83 protein && PF03798:TLC domain|PF08390:TRAM1-like protein
Lachesis_group0__13_contigs__l.g3650	131.9081	301.0579	-1.19116	0.006486	0.034697	1347	- && A2QBQ3.1 RecName: Full=Probable endo-1,3(4)-beta-glucanase An02g00850; AltName: Full=Mixed-linked glucanase An02g00850; Flags: Precursor && PF00652:Ricin-type beta-trefoil lectin domain
Lachesis_group0__13_contigs__l.g1600	407.6898	934.4788	-1.19718	0.005432	0.030694	1395	- && Q9P7L5.1 RecName: Full=Ornithine aminotransferase car2; AltName: Full=Ornithine--oxo-acid aminotransferase && PF00202:Aminotransferase class-III
Lachesis_group0__13_contigs__l.g10317	265.3583	610.9545	-1.20384	0.006243	0.033862	2376	- && O94556.2 RecName: Full=Anaphase-promoting complex subunit 8; AltName: Full=20S cyclosome/APC complex protein apc8; AltName: Full=Cell untimely torn protein 23 && PF04049:Anaphase promoting complex subunit 8 / Cdc23|PF13414:TPR repeat
Lachesis_group0__13_contigs__l.g10550	211.4196	487.2438	-1.205	0.005796	0.03203	2910	- && P29064.1 RecName: Full=Alpha-glucosidase; AltName: Full=Maltase; Contains: RecName: Full=Alpha-glucosidase subunit 1; Contains: RecName: Full=Alpha-glucosidase subunit 2; Flags: Precursor && PF16863:N-terminal barrel of NtMGAM and CtMGAM, maltase-glucoamylase|PF01055:Glycosyl hydrolases family 31

Lachesis_group0__13_contigs__l.g6911	164.4791	380.4918	-1.21085	0.010593	0.04922	246	- && - && PF06522:NADH-ubiquinone reductase complex 1 MLRQ subunit
Lachesis_group0__13_contigs__l.g7742	1040.985	2424.548	-1.21999	0.009744	0.046456	2382	- && Q96AX1.1 RecName: Full=Vacuolar protein sorting-associated protein 33A; Short=hVPS33A && PF00995:Sec1 family
Lachesis_group0__13_contigs__l.g11052	11191.36	26117.57	-1.22265	0.006065	0.033142	1653	- && A0A0B5EMG9.1 RecName: Full=Efflux pump FUBT; AltName: Full=Fusaric acid biosynthesis protein T; AltName: Full=Fusaric acid transporter && PF07690:Major Facilitator Superfamily
Lachesis_group0__13_contigs__l.g8157	3175.926	7415.227	-1.22331	0.003725	0.023372	3306	- && - && PF03370:Carbohydrate/starch-binding module (family 21)
Lachesis_group0__13_contigs__l.g11147	2894.821	6765.66	-1.22472	0.003272	0.021353	1140	- && Q56WD9.2 RecName: Full=3-ketoacyl-CoA thiolase 2, peroxisomal; AltName: Full=Acetyl-CoA acyltransferase 2; AltName: Full=Beta-ketothiolase 2; AltName: Full=Peroxisomal 3-oxoacyl-CoA thiolase 2; AltName: Full=Peroxisome defective protein 1; Flags: Precursor && PF02803:Thiolase, C-terminal domain|PF00108:Thiolase, N-terminal domain
Lachesis_group0__13_contigs__l.g1697	3740.456	8825.505	-1.23845	0.002167	0.015953	2661	- && Q7ZUW2.1 RecName: Full=Hypoxia up-regulated protein 1; Flags: Precursor && PF00012:Hsp70 protein
Lachesis_group0__13_contigs__l.g4651	2286.384	5408.565	-1.24225	0.003539	0.022582	3114	- && P40317.2 RecName: Full=Protein SOK1 && PF05794:T-complex protein 11
Lachesis_group0__13_contigs__l.g6356	3170.642	7506.778	-1.24345	0.00388	0.024048	2613	- && Q17RB8.2 RecName: Full=LON peptidase N-terminal domain and RING finger protein 1; AltName: Full=RING finger protein 191 &&

							PF02190:ATP-dependent protease La (LON) substrate-binding domain
Lachesis_group0__13_contigs__l.g2738	851.7496	2018.94	-1.24513	0.009221	0.044634	1974	- && - && PF00443:Ubiquitin carboxyl-terminal hydrolase
Lachesis_group0__13_contigs__l.g8784	158.8234	376.4083	-1.24584	0.00688	0.036309	4407	- && - && PF05729:NACHT domain
Lachesis_group0__13_contigs__l.g6607	4255.424	10093.66	-1.24609	0.008344	0.041801	657	- && O13737.2 RecName: Full=Uncharacterized endoplasmic reticulum membrane protein C16E8.02 && PF06127:Protein of unknown function (DUF962)
Lachesis_group0__13_contigs__l.g1113	2019.863	4810.68	-1.25199	0.010155	0.047729	1857	- && Q2HXK9.2 RecName: Full=Respiratory burst oxidase homolog protein D; AltName: Full=NADPH oxidase RBOHD; AltName: Full=StRBOHD && PF08030:Ferric reductase NAD binding domain|PF01794:Ferric reductase like transmembrane component|PF08022:FAD-binding domain
Lachesis_group0__13_contigs__l.g9021	101.7582	243.2012	-1.2565	0.009545	0.045626	879	- && Q5F4C0.1 RecName: Full=Voltage-gated hydrogen channel 1; AltName: Full=Hydrogen voltage-gated channel 1; Short=HV1 && -
Lachesis_group0__13_contigs__l.g6534	4141.554	9897.471	-1.2569	0.001041	0.009549	1761	- && P26585.1 RecName: Full=HMG1/2-like protein; AltName: Full=Protein SB11 && PF09011:HMG-box domain|PF00505:HMG (high mobility group) box
Lachesis_group0__13_contigs__l.g3391	140.1299	335.8862	-1.26177	0.005635	0.031467	765	- && - && PF11807:Domain of unknown function (DUF3328)
Lachesis_group0__13_contigs__l.g4610	768.9759	1855.875	-1.27107	0.002867	0.019448	918	- && P43069.1 RecName: Full=Cell division control protein 25 && PF00617:RasGEF domain

Lachesis_group0__13_contigs__l.g11286	285.0414	688.4189	-1.27255	0.004524	0.026852	1224	- && Q59ZX3.1 RecName: Full=Repressed by EFG1 protein 1; AltName: Full=PRY family cell wall protein 2; Flags: Precursor && PF00188:Cysteine-rich secretory protein family
Lachesis_group0__13_contigs__l.g9100	69.68355	168.8631	-1.27579	0.008895	0.043683	687	- && Q00719.1 RecName: Full=O-methyltransferase MdmC && PF01596:O-methyltransferase
Lachesis_group0__13_contigs__l.g8150	294.2243	712.9446	-1.27703	0.003137	0.020661	1389	- && Q96V64.1 RecName: Full=Glucan 1,3-beta-glucosidase; AltName: Full=Exo-1,3-beta-glucanase; Flags: Precursor && PF00150:Cellulase (glycosyl hydrolase family 5)
Lachesis_group0__13_contigs__l.g2117	123.1471	298.3861	-1.27813	0.007287	0.037981	1416	- && J9MJK9.1 RecName: Full=Transaldolase; Short=FoTal && PF00923:Transaldolase
Lachesis_group0__13_contigs__l.g8960	2357.926	5720.471	-1.27868	0.004075	0.025013	1308	- && - && PF10021:Uncharacterized protein conserved in bacteria (DUF2263)
Lachesis_group0__13_contigs__l.g4977	1191.175	2893.305	-1.28049	0.007409	0.038309	1548	- && Q5A220.2 RecName: Full=Repressor of filamentous growth 1 && PF00505:HMG (high mobility group) box
Lachesis_group0__13_contigs__l.g7156	121605	295580.7	-1.28135	0.001064	0.009701	3507	- && Q9P7Q4.1 RecName: Full=Vesicular-fusion protein sec18 && PF02933:Cell division protein 48 (CDC48), domain 2|PF00004:ATPase family associated with various cellular activities (AAA)
Lachesis_group0__13_contigs__l.g1942	441.422	1073.422	-1.28198	0.003579	0.022774	1155	- && - && PF14249:Tocopherol cyclase
Lachesis_group0__13_contigs__l.g8852	1147.712	2792.55	-1.28293	0.001299	0.011073	1977	- && Q8K194.1 RecName: Full=U4/U6.U5 small nuclear ribonucleoprotein 27 kDa protein; Short=U4/U6.U5 snRNP 27 kDa protein; Short=U4/U6.U5-27K; AltName: Full=U4/U6.U5 tri-snRNP-associated protein 3 && PF08648:Protein of unknown function (DUF1777)

Lachesis_group0__13_contigs__l.g11231	337.5121	828.6786	-1.29628	0.004968	0.028786	9282	- && Q07878.1 RecName: Full=Vacuolar protein sorting-associated protein 13; AltName: Full=Suppression of the onset of impotence protein 1; AltName: Full=Vacuolar protein-targeting protein 2 && PF09333:Autophagy-related protein C terminal domain|PF06650:SHR-binding domain of vacuolar-sorting associated protein 13|PF12624:N-terminal region of Chorein or VPS13|PF16910:Repeating coiled region of VPS13|PF16909:Vacuolar-sorting-associated 13 protein C-terminal|PF16908:Vacuolar sorting-associated protein 13, N-terminal
Lachesis_group0__13_contigs__l.g6900	1398.04	3433.581	-1.29633	0.000799	0.007856	1515	- && Q92407.1 RecName: Full=Glucokinase; AltName: Full=Glucose kinase; Short=GLK && PF00349:Hexokinase|PF03727:Hexokinase
Lachesis_group0__13_contigs__l.g9387	290.0497	713.2543	-1.29799	0.009649	0.046043	4206	- && Q13423.3 RecName: Full=NAD(P) transhydrogenase, mitochondrial; AltName: Full=Nicotinamide nucleotide transhydrogenase; AltName: Full=Pyridine nucleotide transhydrogenase; Flags: Precursor && PF02233:NAD(P) transhydrogenase beta subunit|PF00701:Dihydrodipicolinate synthetase family|PF05222:Alanine dehydrogenase/PNT, N-terminal domain|PF01262:Alanine dehydrogenase/PNT, C-terminal domain|PF12769:4TM region of pyridine nucleotide transhydrogenase, mitoch

Lachesis_group0__13_contigs__l.g4517	670.6601	1652.581	-1.30128	0.008501	0.042391	1770	- && P54857.1 RecName: Full=Lipase 2; AltName: Full=Triacylglycerol lipase 2 && -
Lachesis_group0__13_contigs__l.g8291	21494.64	53456.38	-1.31439	0.00066	0.006823	5076	- && Q0DV32.2 RecName: Full=4-coumarate--CoA ligase-like 1 && PF00501:AMP-binding enzyme|PF00441:Acyl-CoA dehydrogenase, C-terminal domain|PF02353:Mycolic acid cyclopropane synthetase|PF02770:Acyl-CoA dehydrogenase, middle domain|PF13193:AMP-binding enzyme C-terminal domain
Lachesis_group0__13_contigs__l.g4990	1071.231	2665.32	-1.31506	0.008597	0.042736	1596	- && - && PF12937:F-box-like
Lachesis_group0__13_contigs__l.g6187	1640.43	4091.885	-1.31877	0.010214	0.047898	2751	- && - && PF00400:WD domain, G-beta repeat
Lachesis_group0__13_contigs__l.g1157	89.80105	224.6208	-1.32264	0.009325	0.044968	2130	- && A8DZE7.1 RecName: Full=Short-chain dehydrogenase/reductase family 42E member 1 && PF01073:3-beta hydroxysteroid dehydrogenase/isomerase family
Lachesis_group0__13_contigs__l.g1698	67.9899	170.2299	-1.32474	0.008395	0.041996	1695	- && - && PF00651:BTB/POZ domain
Lachesis_group0__13_contigs__l.g11628	995.4142	2495.128	-1.32584	0.003026	0.020143	4080	- && Q9Y810.1 RecName: Full=Cytochrome oxidase assembly protein shy1; AltName: Full=SURF1-like protein && PF02104:SURF1 family
Lachesis_group0__13_contigs__l.g5096	140.1547	351.9784	-1.32877	0.004319	0.026026	1293	- && - && PF00339:Arrestin (or S-antigen), N-terminal domain
Lachesis_group0__13_contigs__l.g10953	373.1148	937.1542	-1.32924	0.008832	0.043564	1617	- && Q7KW39.1 RecName: Full=Probable methylmalonate-semialdehyde dehydrogenase [acylating], mitochondrial; Short=MMSDH; Short=Malonate-semialdehyde dehydrogenase [acylating]; Flags: Precursor && PF00171:Aldehyde dehydrogenase family

Lachesis_group0__13_contigs__l.g9938	8570.134	21556.4	-1.33072	0.005271	0.030032	3204	- && Q9Y8G7.1 RecName: Full=Bifunctional cytochrome P450/NADPH--P450 reductase; AltName: Full=Cytochrome P450foxy; AltName: Full=Fatty acid omega-hydroxylase; AltName: Full=P450foxy; Includes: RecName: Full=Cytochrome P450 505; Includes: RecName: Full=NADPH--cytochrome P450 reductase && PF00067:Cytochrome P450|PF00258:Flavodoxin|PF00667:FAD binding domain|PF00175:Oxidoreductase NAD-binding domain
Lachesis_group0__13_contigs__l.g11426	713.7168	1806.629	-1.34005	0.002131	0.015779	927	- && - && PF13622:Thioesterase-like superfamily
Lachesis_group0__13_contigs__l.g10294	1934.113	4898.247	-1.34064	0.002918	0.019635	699	- && Q9UUM7.1 RecName: Full=Protein hob3; AltName: Full=Homolog of Bin3 && PF03114:BAR domain
Lachesis_group0__13_contigs__l.g10201	250.8697	635.7417	-1.34212	0.002835	0.019301	633	- && - && PF13883:Pyridoxamine 5'-phosphate oxidase
Lachesis_group0__13_contigs__l.g7791	5183.248	13158.39	-1.34408	0.00572	0.031764	5385	- && Q9P792.1 RecName: Full=ZZ-type zinc finger-containing protein P35G2.11c && PF16158:Ig-like domain from next to BRCA1 gene|PF00569:Zinc finger, ZZ type
Lachesis_group0__13_contigs__l.g10191	43763.85	111293.4	-1.34656	0.005353	0.030341	2163	- && P87049.3 RecName: Full=G1/S-specific cyclin pas1 && -
Lachesis_group0__13_contigs__l.g10858	691.9108	1766.263	-1.35208	0.010008	0.047261	1041	- && - && PF01180:Dihydroorotate dehydrogenase
Lachesis_group0__13_contigs__l.g6864	30162.72	77166.66	-1.35522	0.002925	0.019647	543	- && - && PF00397:WW domain
Lachesis_group0__13_contigs__l.g8441	294.1157	752.6999	-1.35544	0.003716	0.023358	1602	- && - && PF01699:Sodium/calcium exchanger protein

Lachesis_group0__13_contigs__l.g9601	143.756	368.0468	-1.35643	0.004458	0.026558	765	- && O74758.1 RecName: Full=Uncharacterized protein C17D11.03c && -
Lachesis_group0__13_contigs__l.g8076	2502.706	6442.579	-1.36419	0.002416	0.017292	1332	- && P42894.1 RecName: Full=Enolase; AltName: Full=2-phospho-D-glycerate hydro-lyase; AltName: Full=2-phosphoglycerate dehydratase && PF00113:Enolase, C-terminal TIM barrel domain|PF03952:Enolase, N-terminal domain
Lachesis_group0__13_contigs__l.g4151	347.0612	893.7447	-1.36484	0.008702	0.043122	1299	- && Q8NBN7.2 RecName: Full=Retinol dehydrogenase 13; AltName: Full=Short chain dehydrogenase/reductase family 7C member 3 && PF00106:short chain dehydrogenase|PF02566:OsmC-like protein
Lachesis_group0__13_contigs__l.g4940	3640.226	9410.051	-1.37019	0.003222	0.021127	1140	- && Q06497.1 RecName: Full=Peroxisomal adenine nucleotide transporter 1 && PF00153:Mitochondrial carrier protein
Lachesis_group0__13_contigs__l.g7712	178.6678	463.229	-1.37499	0.001429	0.011791	852	- && G4V4T7.1 RecName: Full=Enoyl-CoA-hydratase && PF00378:Enoyl-CoA hydratase/isomerase
Lachesis_group0__13_contigs__l.g10883	112.5601	291.8914	-1.37565	0.007761	0.039715	1593	- && Q54PK9.1 RecName: Full=3-phosphoinositide-dependent protein kinase B; AltName: Full=Pdk-class protein kinase b && PF00069:Protein kinase domain
Lachesis_group0__13_contigs__l.g6679	413.5621	1074.782	-1.37811	0.001991	0.015048	1143	- && - && PF03227:Gamma interferon inducible lysosomal thiol reductase (GILT)|PF09774:Caffeine-induced death protein 2
Lachesis_group0__13_contigs__l.g5756	617.7663	1606.011	-1.37846	0.006273	0.033969	837	- && - && PF00293:NUDIX domain
Lachesis_group0__13_contigs__l.g530	522.0167	1357.235	-1.37882	0.002501	0.017661	1044	- && Q9UT59.1 RecName: Full=Putative uncharacterized oxidoreductase C513.07 &&

							PF01370:NAD dependent epimerase/dehydratase family
Lachesis_group0__13_contigs__l.g6418	340.019	884.0811	-1.37889	0.008721	0.043156	1641	- && Q07982.2 RecName: Full=Glucose--fructose oxidoreductase; Short=GFOR; Flags: Precursor && PF02894:Oxidoreductase family, C-terminal alpha/beta domain|PF01408:Oxidoreductase family, NAD-binding Rossmann fold
Lachesis_group0__13_contigs__l.g2048	8838.848	23017.42	-1.38081	0.003508	0.022484	1947	- && P32949.1 RecName: Full=Lipase 5; Flags: Precursor && PF00135:Carboxylesterase family
Lachesis_group0__13_contigs__l.g1187	1576.706	4122.739	-1.38674	0.001091	0.009858	2616	- && P06738.4 RecName: Full=Glycogen phosphorylase && PF00343:Carbohydrate phosphorylase
Lachesis_group0__13_contigs__l.g458	242.6757	635.5279	-1.38903	0.002665	0.018455	6915	- && P9WQE7.1 RecName: Full=Phthiocerol synthesis polyketide synthase type I PpsA; AltName: Full=Beta-ketoacyl-acyl-carrier-protein synthase I && PF00698:Acyl transferase domain|PF02801:Beta-ketoacyl synthase, C-terminal domain|PF08659:KR domain|PF07993:Male sterility protein|PF14765:Polyketide synthase dehydratase|PF00109:Beta-ketoacyl synthase, N-terminal domain
Lachesis_group0__13_contigs__l.g6053	1731.154	4541.656	-1.39143	0.000826	0.008073	660	- && P40581.1 RecName: Full=Peroxiredoxin HYR1; AltName: Full=Glutathione peroxidase 3; AltName: Full=Hydrogen peroxide resistance protein 1; AltName: Full=Oxidant receptor peroxidase 1; AltName: Full=Phospholipid hydroperoxide glutathione peroxidase 3;

							Short=PHGPx3 && PF00255:Glutathione peroxidase
Lachesis_group0__13_contigs__l.g8451	123.0174	323.2958	-1.395	0.00433	0.026067	4047	- && - && PF13191:AAA ATPase domain
Lachesis_group0__13_contigs__l.g8399	2569.974	6771.641	-1.39783	0.00269	0.018568	3954	- && Q6LPK6.1 RecName: Full=Lipid A export ATP-binding/permease protein MsbA && -
Lachesis_group0__13_contigs__l.g5810	710.4505	1871.986	-1.398	0.00893	0.043802	3000	- && Q9HDZ2.2 RecName: Full=Protein cwh43 && PF10277:Frag1/DRAM/Sfk1 family
Lachesis_group0__13_contigs__l.g7258	4717.184	12441.49	-1.39919	0.000406	0.004813	3924	- && P53721.1 RecName: Full=Respiratory supercomplex factor 2, mitochondrial; AltName: Full=Altered inheritance of mitochondria protein 38 && -
Lachesis_group0__13_contigs__l.g5018	107.5631	284.0091	-1.40093	0.002185	0.016047	1641	- && Q5A744.1 RecName: Full=Protein SDS23 && -
Lachesis_group0__13_contigs__l.g959	402.615	1063.403	-1.40182	0.004685	0.027574	585	- && - && PF05180:DNL zinc finger
Lachesis_group0__13_contigs__l.g5110	6459.193	17080.51	-1.40294	0.001326	0.011221	744	- && - && PF13430:Domain of unknown function (DUF4112)
Lachesis_group0__13_contigs__l.g6108	129.5828	343.7123	-1.40714	0.001132	0.010067	2496	- && P55487.1 RecName: Full=Uncharacterized monooxygenase y4iD && PF13738:Pyridine nucleotide-disulphide oxidoreductase
Lachesis_group0__13_contigs__l.g7796	2224.284	5903.372	-1.40828	0.002521	0.01774	1215	- && Q58EJ9.1 RecName: Full=Mitochondrial amidoxime-reducing component 1; Short=mARC1 && PF03476:MOSC N-terminal beta barrel domain|PF03473:MOSC domain
Lachesis_group0__13_contigs__l.g4889	104.3974	277.8331	-1.41386	0.009911	0.046904	3765	- && P34211.3 RecName: Full=Putative hydrolase YuaR; Flags: Precursor && PF00561:alpha/beta hydrolase fold|PF08386:TAP-like protein
Lachesis_group0__13_contigs__l.g7762	143.9002	384.1034	-1.41662	0.007862	0.040065	1095	- && Q5AUY5.1 RecName: Full=Zinc-binding alcohol dehydrogenase domain-containing protein cipB; AltName: Full=Concanamycin-induced

								protein B && PF08240:Alcohol dehydrogenase GroES-like domain|PF00107:Zinc-binding dehydrogenase
Lachesis_group0__13_contigs__l.g4574	638.7761	1710.303	-1.42126		0.006051	0.033109	2949	- && Q5E9V4.1 RecName: Full=Protein RRNAD1 && PF13679:Methyltransferase domain
Lachesis_group0__13_contigs__l.g8571	43.77659	117.1315	-1.42221		0.008999	0.043933	1551	- && P0CO49.1 RecName: Full=Kynurenine 3-monooxygenase; AltName: Full=Biosynthesis of nicotinic acid protein 4; AltName: Full=Kynurenine 3-hydroxylase && PF01494:FAD binding domain
Lachesis_group0__13_contigs__l.g11060	332.6549	892.4689	-1.4241		0.004373	0.026243	1650	- && P49374.1 RecName: Full=High-affinity glucose transporter && PF00083:Sugar (and other) transporter
Lachesis_group0__13_contigs__l.g2033	160.7094	431.645	-1.4266		0.00556	0.031189	5091	- && Q9P7V2.1 RecName: Full=ATP-binding cassette transporter abc4; Short=ABC transporter abc4; AltName: Full=ATP-energized glutathione S-conjugate pump abc4; AltName: Full=Glutathione S-conjugate-transporting ATPase abc4 && PF00005:ABC transporter|PF00664:ABC transporter transmembrane region
Lachesis_group0__13_contigs__l.g11011	3782.841	10196.55	-1.43055		0.006437	0.034551	1710	- && - && PF13805:Eisosome component PIL1
Lachesis_group0__13_contigs__l.g2083	88.88895	239.8967	-1.4324		0.010217	0.047898	1254	- && - && PF00010:Helix-loop-helix DNA-binding domain
Lachesis_group0__13_contigs__l.g7442	194.0115	524.2408	-1.4353		0.005049	0.029131	1104	- && Q148L6.1 RecName: Full=Trans-1,2-dihydrobenzene-1,2-diol dehydrogenase; AltName: Full=D-xylose 1-dehydrogenase; AltName: Full=D-xylose-NADP dehydrogenase; AltName: Full=Dimeric dihydrodiol

							dehydrogenase && PF01408:Oxidoreductase family, NAD-binding Rossmann fold
Lachesis_group0__13_contigs__l.g638	707.142	1922.321	-1.44295	0.002093	0.015588	1788	- && - && PF15786:PET assembly of cytochrome c oxidase, mitochondrial
Lachesis_group0__13_contigs__l.g9399	679.7225	1857.037	-1.45013	0.00213	0.015779	1464	- && Q9RC92.1 RecName: Full=Unsaturated glucuronyl hydrolase; Short=UGL; AltName: Full=Glycosaminoglycan hydrolase; AltName: Full=Glycuronidase; AltName: Full=Unsaturated uronic acid hydrolase && PF07470:Glycosyl Hydrolase Family 88
Lachesis_group0__13_contigs__l.g6761	200.7278	548.8104	-1.45066	0.001119	0.010032	1542	- && O43000.2 RecName: Full=Pantothenate transporter liz1 && PF07690:Major Facilitator Superfamily
Lachesis_group0__13_contigs__l.g7783	1098.955	3005.704	-1.45164	0.001331	0.011227	1992	- && O96904.1 RecName: Full=Drainin; AltName: Full=TBC1 domain family member 12 homolog && PF00566:Rab-GTPase-TBC domain
Lachesis_group0__13_contigs__l.g8262	234.9166	642.7735	-1.45244	0.001053	0.009627	5529	- && - && PF13191:AAA ATPase domain
Lachesis_group0__13_contigs__l.g10472	2150.091	5884.373	-1.45255	0.00024	0.003294	2484	- && A3KMV8.1 RecName: Full=Ran-binding protein 10; Short=RanBP10 && PF00622:SPRY domain|PF10607:CTLH/CRA C-terminal to LisH motif domain
Lachesis_group0__13_contigs__l.g6029	5014.38	13737.45	-1.454	0.002056	0.015422	2454	- && O42938.1 RecName: Full=ATP-dependent 6-phosphofructokinase; Short=ATP-PFK; Short=Phosphofructokinase; AltName: Full=Phosphohexokinase && PF00365:Phosphofructokinase
Lachesis_group0__13_contigs__l.g5303	147.057	403.05	-1.45472	0.001206	0.010484	2649	- && Q01373.1 RecName: Full=Peroxisomal hydratase-dehydrogenase-epimerase; Short=HDE;

							AltName: Full=Multifunctional beta-oxidation protein; Short=MFP; Includes: RecName: Full=2-enoyl-CoA hydratase; Includes: RecName: Full=(3R)-3-hydroxyacyl-CoA dehydrogenase && PF00106:short chain dehydrogenase|PF01575:MaoC like domain
Lachesis_group0__13_contigs__l.g2075	339.9873	933.2751	-1.4569	0.007239	0.037747	921	- && Q03219.1 RecName: Full=Uncharacterized protein YMR178W && PF00994:Probable molybdopterin binding domain
Lachesis_group0__13_contigs__l.g9922	727.6441	1997.339	-1.45692	0.004684	0.027574	1575	- && - && PF01753:MYND finger
Lachesis_group0__13_contigs__l.g9692	338.8713	930.4167	-1.4571	0.002794	0.019073	435	- && - && PF00067:Cytochrome P450
Lachesis_group0__13_contigs__l.g2892	656.799	1804.584	-1.45794	0.003699	0.023293	1668	- && P0CU32.1 RecName: Full=FAD-linked oxidoreductase CHGG_01242-2; AltName: Full=Chaetoglobosin biosynthesis protein CHGG_01242-2 && PF01565:FAD binding domain
Lachesis_group0__13_contigs__l.g7429	11106.09	30716.83	-1.46768	0.003364	0.021772	1053	- && Q5UP73.1 RecName: Full=Putative band 7 family protein R614 && PF01145:SPFH domain / Band 7 family
Lachesis_group0__13_contigs__l.g11363	1170.449	3249.138	-1.47309	0.005181	0.029691	2277	- && Q9H4L5.1 RecName: Full=Oxysterol-binding protein-related protein 3; Short=ORP-3; Short=OSBP-related protein 3 && PF15409:Pleckstrin homology domain|PF01237:Oxysterol-binding protein
Lachesis_group0__13_contigs__l.g3515	144.5009	401.1618	-1.4733	0.008867	0.043642	1536	- && Q6UEF1.1 RecName: Full=Oxidoreductase AflY; AltName: Full=Aflatoxin biosynthesis protein Y && PF14027:Protein of unknown function (DUF4243)

Lachesis_group0__13_contigs__l.g5773	93.88881	260.6784	-1.47461	0.010391	0.048468	2262	- && Q7Z9M8.1 RecName: Full=Xyloglucanase; Short=XG; AltName: Full=Cel74a; Flags: Precursor && -
Lachesis_group0__13_contigs__l.g360	79.04213	220.7297	-1.48185	0.004741	0.027853	2511	- && Q5B6Q3.1 RecName: Full=Glucan endo-1,6-beta-glucosidase B; AltName: Full=Beta-1,6-glucanase B; AltName: Full=Endo-1,6-beta-D-glucanase B; AltName: Full=Endo-1,6-beta-glucanase B; Flags: Precursor && -
Lachesis_group0__13_contigs__l.g5886	333.4815	932.0632	-1.483	0.004029	0.024801	999	- && Q6F4M7.1 RecName: Full=Hydroxyquinol 1,2-dioxygenase && PF00775:Dioxygenase|PF04444:Catechol dioxygenase N terminus
Lachesis_group0__13_contigs__l.g8755	173.9798	488.9122	-1.49127	0.000849	0.008258	507	- && Q9Y6Z9.1 RecName: Full=Sorbose reductase sou1; AltName: Full=Sorbitol utilization protein sou1 && -
Lachesis_group0__13_contigs__l.g9104	68.14695	191.5716	-1.49237	0.005495	0.030953	2031	- && - && PF12862:Anaphase-promoting complex subunit 5
Lachesis_group0__13_contigs__l.g273	4022.967	11330.75	-1.49392	0.000128	0.002148	1488	- && - && PF05057:Putative serine esterase (DUF676)
Lachesis_group0__13_contigs__l.g2571	194.814	549.3854	-1.49594	0.000745	0.007461	4692	- && - && PF00400:WD domain, G-beta repeat
Lachesis_group0__13_contigs__l.g5310	218.0529	615.7051	-1.49804	0.003655	0.023131	1560	- && P30284.1 RecName: Full=G2/mitotic-specific cyclin-B && PF00134:Cyclin, N-terminal domain|PF02984:Cyclin, C-terminal domain
Lachesis_group0__13_contigs__l.g5795	67.07184	189.1421	-1.49868	0.005887	0.032456	954	- && Q54GH4.1 RecName: Full=Inositol oxygenase; AltName: Full=Myo-inositol oxygenase; Short=MI oxygenase && PF05153:Myo-inositol oxygenase
Lachesis_group0__13_contigs__l.g11150	1929.683	5459.876	-1.50054	0.002768	0.018944	876	- && - && PF00622:SPRY domain

Lachesis_group0__13_contigs__l.g8622	2340.618	6630.038	-1.50216	0.005571	0.031189	858	- && - && PF11274:Protein of unknown function (DUF3074)
Lachesis_group0__13_contigs__l.g2720	4986.176	14127.01	-1.50247	0.000353	0.004332	1416	- && O02373.1 RecName: Full=UDP-glucose 6-dehydrogenase; Short=UDP-Glc dehydrogenase; Short=UDP-GlcDH; Short=UDPGDH; AltName: Full=Protein sugarless; AltName: Full=Protein suppenkasper && PF03721:UDP-glucose/GDP-mannose dehydrogenase family, NAD binding domain|PF00984:UDP-glucose/GDP-mannose dehydrogenase family, central domain|PF03720:UDP-glucose/GDP-mannose dehydrogenase family, UDP binding domain
Lachesis_group0__13_contigs__l.g11575	700.2748	1989.945	-1.50668	0.002608	0.018142	1557	- && - && PF09994:Uncharacterized alpha/beta hydrolase domain (DUF2235)
Lachesis_group0__13_contigs__l.g5020	938.7133	2667.652	-1.50705	0.002433	0.017335	1728	- && Q47944.1 RecName: Full=L-sorbose 1-dehydrogenase; Short=SDH && PF00732:GMC oxidoreductase|PF05199:GMC oxidoreductase
Lachesis_group0__13_contigs__l.g9078	1086.94	3090.957	-1.50799	0.001624	0.012881	705	- && - && PF06687:SUR7/PalI family
Lachesis_group0__13_contigs__l.g571	136.9108	389.2017	-1.50831	0.001145	0.010132	1833	- && O13902.1 RecName: Full=Dihydroxyacetone kinase 1; Short=DHA kinase 1; AltName: Full=Glycerone kinase 1; AltName: Full=Triokinase 1; AltName: Full=Triose kinase 1 && PF02734:DAK2 domain|PF02733:Dak1 domain
Lachesis_group0__13_contigs__l.g2585	88.01787	252.099	-1.52036	0.010577	0.049186	1092	- && Q7LL04.2 RecName: Full=UPF0676 protein C1494.01 && PF03171:2OG-Fe(II) oxygenase superfamily|PF14226:non-haem dioxygenase in morphine synthesis N-terminal

Lachesis_group0__13_contigs__l.g109	116.5478	335.3026	-1.52355	0.000613	0.006434	1419	- && Q12436.1 RecName: Full=Zinc-regulated transporter 2; AltName: Full=Low-affinity zinc transport protein ZRT2 && PF02535:ZIP Zinc transporter
Lachesis_group0__13_contigs__l.g4086	196.492	565.4383	-1.52487	0.008389	0.041988	1626	- && O42615.1 RecName: Full=Threonine dehydratase, mitochondrial; AltName: Full=Threonine deaminase; Flags: Precursor && PF00291:Pyridoxal-phosphate dependent enzyme|PF00585:C-terminal regulatory domain of Threonine dehydratase
Lachesis_group0__13_contigs__l.g1320	438.489	1264.483	-1.52817	0.005668	0.031585	1848	- && - && PF03007:Wax ester synthase-like Acyl-CoA acyltransferase domain|PF06974:Protein of unknown function (DUF1298)
Lachesis_group0__13_contigs__l.g10682	10602.69	30664.98	-1.53217	0.000126	0.002118	1206	- && Q9UUA1.2 RecName: Full=Oxysterol-binding protein homolog C23B6.01c && PF01237:Oxysterol-binding protein
Lachesis_group0__13_contigs__l.g5236	21614.72	62528.49	-1.5325	0.005734	0.031823	3210	- && - && PF04082:Fungal specific transcription factor domain
Lachesis_group0__13_contigs__l.g2526	68.23822	197.228	-1.53294	0.009849	0.046825	1137	- && O31463.1 RecName: Full=Homocysteine S-methyltransferase YbgG; AltName: Full=S-methylmethionine:homocysteine methyltransferase && PF02574:Homocysteine S-methyltransferase
Lachesis_group0__13_contigs__l.g8530	2413.385	7012.377	-1.53887	0.00382	0.023794	1479	- && B7STY2.1 RecName: Full=L-tyrosine:2-oxoglutarate aminotransferase atrD; AltName: Full=Atromentin biosynthesis protein D && PF00155:Aminotransferase class I and II

Lachesis_group0__13_contigs__l.g1311	87.46576	254.2656	-1.5397	0.010551	0.049085	2817	- && Q9URY6.1 RecName: Full=Probable urea active transporter 3 && PF00474:Sodium:solute symporter family|PF00011:Hsp20/alpha crystallin family
Lachesis_group0__13_contigs__l.g7958	1922.78	5633.404	-1.55092	0.001766	0.013753	1944	- && P87035.1 RecName: Full=Guanine nucleotide-binding protein alpha-4 subunit && PF00503:G-protein alpha subunit
Lachesis_group0__13_contigs__l.g1239	123.246	361.2849	-1.55158	0.000589	0.006271	4284	- && Q5UQ40.1 RecName: Full=Probable bifunctional E2/E3 enzyme R795; Includes: RecName: Full=E3 ubiquitin-protein ligase; AltName: Full=RING-type E3 ubiquitin transferase; Includes: RecName: Full=Ubiquitin-conjugating enzyme E2; AltName: Full=E2 ubiquitin-conjugating enzyme && PF00179:Ubiquitin-conjugating enzyme
Lachesis_group0__13_contigs__l.g5152	178.2769	522.7128	-1.55295	0.002718	0.018714	1971	- && Q9TT38.1 RecName: Full=Cytosolic phospholipase A2; Short=cPLA2; AltName: Full=Phospholipase A2 group IVA; Includes: RecName: Full=Phospholipase A2; AltName: Full=Phosphatidylcholine 2-acylhydrolase; Includes: RecName: Full=Lysophospholipase && PF01735:Lysophospholipase catalytic domain
Lachesis_group0__13_contigs__l.g7314	123.0522	361.4581	-1.55576	0.003661	0.023143	5442	- && - && PF12770:CHAT domain
Lachesis_group0__13_contigs__l.g8402	4461.032	13123.16	-1.55668	0.004657	0.027475	4155	- && O14283.2 RecName: Full=Transcription factor prr1; AltName: Full=Pombe response regulator 1 && PF00447:HSF-type DNA-binding|PF00072:Response regulator receiver domain

Lachesis_group0__13_contigs__l.g4834	1154.306	3395.573	-1.55686	0.00336	0.021772	1062	- && Q9P7F4.1 RecName: Full=Zinc-type alcohol dehydrogenase-like protein C2E1P3.01 && PF08240:Alcohol dehydrogenase GroES-like domain|PF00107:Zinc-binding dehydrogenase
Lachesis_group0__13_contigs__l.g4228	383.5028	1128.082	-1.55706	0.006082	0.033212	3066	- && Q556W1.1 RecName: Full=D-aspartate oxidase; Short=DASOX; AltName: Full=DDO && PF01266:FAD dependent oxidoreductase
Lachesis_group0__13_contigs__l.g5820	50.93301	150.0069	-1.55834	0.003511	0.022484	1401	- && D4AUF4.1 RecName: Full=WSC domain-containing protein ARB_07870; Flags: Precursor && PF09362:Domain of unknown function (DUF1996)|PF01822:WSC domain
Lachesis_group0__13_contigs__l.g6317	2978.019	8785.554	-1.56078	0.000701	0.007147	1269	- && Q9DBL1.1 RecName: Full=Short/branched chain specific acyl-CoA dehydrogenase, mitochondrial; Short=SBCAD; AltName: Full=2-methyl branched chain acyl-CoA dehydrogenase; Short=2-MEBCAD; AltName: Full=2-methylbutyryl-coenzyme A dehydrogenase; Short=2-methylbutyryl-CoA dehydrogenase; Flags: Precursor && PF00441:Acyl-CoA dehydrogenase, C-terminal domain|PF02770:Acyl-CoA dehydrogenase, middle domain|PF02771:Acyl-CoA dehydrogenase, N-terminal domain
Lachesis_group0__13_contigs__l.g2906	220.9357	653.1955	-1.56407	0.000244	0.003325	1617	- && P17549.1 RecName: Full=Benzoate 4-monooxygenase; AltName: Full=Benzoate-para-hydroxylase; Short=BpH; AltName: Full=Cytochrome P450 53 && PF00067:Cytochrome P450

Lachesis_group0__13_contigs__l.g3145	799.2189	2365.328	-1.56541	0.002707	0.018665	1869	- && A1CPX0.1 RecName: Full=Probable quinate permease; AltName: Full=Quinate transporter && PF00083:Sugar (and other) transporter
Lachesis_group0__13_contigs__l.g6702	2406.147	7138.924	-1.569	0.001156	0.010181	2061	- && O94913.3 RecName: Full=Pre-mRNA cleavage complex 2 protein Pcf11; AltName: Full=Pre-mRNA cleavage complex II protein Pcf11 && PF04818:RNA polymerase II-binding domain.
Lachesis_group0__13_contigs__l.g9927	66.09025	196.3473	-1.57012	0.007095	0.037212	2304	- && Q9C1X1.1 RecName: Full=Periodic tryptophan protein 2 homolog && PF00400:WD domain, G-beta repeat
Lachesis_group0__13_contigs__l.g2168	1062.8	3157.317	-1.5708	0.00035	0.004295	1887	- && Q9N2V6.3 RecName: Full=Guanine nucleotide-binding protein alpha-16 subunit && PF00503:G-protein alpha subunit
Lachesis_group0__13_contigs__l.g9683	4429.684	13160.84	-1.57098	0.004198	0.025478	1131	- && P07250.1 RecName: Full=Inositol polyphosphate multikinase; Short=IPMK; AltName: Full=Arginine metabolism regulation protein III && PF03770:Inositol polyphosphate kinase
Lachesis_group0__13_contigs__l.g717	386.9264	1152.481	-1.57504	0.009865	0.046827	2310	- && Q942X8.1 RecName: Full=Probable potassium transporter 2; AltName: Full=OsHAK2 && PF02705:K+ potassium transporter
Lachesis_group0__13_contigs__l.g10260	74.32044	222.4516	-1.58219	0.001609	0.012792	2925	- && Q804S5.1 RecName: Full=E3 ubiquitin-protein ligase mib1; AltName: Full=Protein mind bomb && PF00569:Zinc finger, ZZ type
Lachesis_group0__13_contigs__l.g11062	2507.165	7555.846	-1.59154	0.000301	0.003877	2691	- && - && PF13886:Domain of unknown function (DUF4203)
Lachesis_group0__13_contigs__l.g3381	49.51954	149.0501	-1.59196	0.002471	0.017509	591	- && B6CHA3.1 RecName: Full=E3 ubiquitin-protein ligase UHRF1; AltName: Full=Nuclear zinc

							finger protein Np95; Short=XNp95; AltName: Full=Ubiquitin-like PHD and RING finger domain-containing protein 1; AltName: Full=Ubiquitin-like-containing PHD and RING finger domains protein 1 && PF02182:SAD/SRA domain
Lachesis_group0__13_contigs__l.g11378	269.0555	813.0034	-1.59564	0.002742	0.01881	1128	- && - && PF01485:IBR domain, a half RING-finger domain
Lachesis_group0__13_contigs__l.g9829	441.2462	1333.865	-1.59591	0.000184	0.002724	1815	- && Q08970.2 RecName: Full=Mitochondrial metal transporter 2; Flags: Precursor && PF01545:Cation efflux family
Lachesis_group0__13_contigs__l.g8714	350.0073	1058.13	-1.59632	0.003727	0.023372	918	- && Q8K370.1 RecName: Full=Acyl-CoA dehydrogenase family member 10; Short=ACAD-10 && PF13419:Haloacid dehalogenase-like hydrolase
Lachesis_group0__13_contigs__l.g6186	336.7162	1023.976	-1.60502	0.001563	0.012533	4623	- && - && PF00400:WD domain, G-beta repeat|PF07971:Glycosyl hydrolase family 92
Lachesis_group0__13_contigs__l.g11441	70.44141	214.3188	-1.60515	0.001956	0.014841	1599	- && P29026.1 RecName: Full=Chitinase 1; Flags: Precursor && PF02839:Carbohydrate binding domain|PF00704:Glycosyl hydrolases family 18
Lachesis_group0__13_contigs__l.g6883	1062.539	3234.223	-1.60591	0.000423	0.004942	3471	- && P0CT24.1 RecName: Full=Glucosidase 2 subunit beta; AltName: Full=Alpha-glucosidase 2 subunit beta; Flags: Precursor && PF12999:Glucosidase II beta subunit-like|PF13015:Glucosidase II beta subunit-like protein
Lachesis_group0__13_contigs__l.g11656	894.3152	2736.21	-1.61338	0.002404	0.01724	1923	- && P10281.2 RecName: Full=Ribonuclease T2; Short=RNase T2; Flags: Precursor && PF00445:Ribonuclease T2 family
Lachesis_group0__13_contigs__l.g11123	33596.81	102844.2	-1.61406	0.000501	0.005613	2772	- && - && PF00564:PB1 domain

Lachesis_group0__13_contigs__l.g11466	1789.514	5486.031	-1.6163	0.00169	0.013251	3792	- && Q9V3C5.1 RecName: Full=Guanine nucleotide exchange factor subunit Rich; AltName: Full=Protein RIC1 homolog && PF04707:PRELI-like family|PF07064:RIC1
Lachesis_group0__13_contigs__l.g3214	25.13045	76.88332	-1.61893	0.010259	0.048054	2151	- && F4JTP5.1 RecName: Full=Serine/threonine-protein kinase STY46; AltName: Full=Serine/threonine/tyrosine-protein kinase 46 && PF07714:Protein tyrosine kinase
Lachesis_group0__13_contigs__l.g8897	828.1351	2550.938	-1.62318	0.005946	0.032688	2439	- && O94289.2 RecName: Full=Ubiquitin homeostasis protein lub1 && PF00400:WD domain, G-beta repeat|PF08324:PUL domain|PF09070:PFU (PLAA family ubiquitin binding)
Lachesis_group0__13_contigs__l.g3078	340.324	1048.759	-1.62344	0.000199	0.002924	2298	- && - && PF00294:pfkB family carbohydrate kinase
Lachesis_group0__13_contigs__l.g2807	24643.94	75982.09	-1.62443	0.002142	0.015825	570	- && - && PF00011:Hsp20/alpha crystallin family
Lachesis_group0__13_contigs__l.g3569	201.8461	622.8931	-1.62636	0.000244	0.003323	3426	- && P34422.2 RecName: Full=Dipeptidyl peptidase family member 6 && PF00326:Prolyl oligopeptidase family
Lachesis_group0__13_contigs__l.g4144	92.63812	286.139	-1.62738	0.004898	0.028534	1809	- && - && PF00651:BTB/POZ domain
Lachesis_group0__13_contigs__l.g128	31.82036	98.52048	-1.6309	0.004909	0.028545	1398	- && P23262.4 RecName: Full=Salicylate hydroxylase; AltName: Full=Salicylate 1-monooxygenase && PF01494:FAD binding domain
Lachesis_group0__13_contigs__l.g1929	1268.34	3929.656	-1.63165	0.006892	0.036335	804	- && P42058.1 RecName: Full=Minor allergen Alt a 7; AltName: Full=Allergen Alt a VII; AltName: Allergen=Alt a 7 && PF03358:NADPH-dependent FMN reductase
Lachesis_group0__13_contigs__l.g11374	26.36975	81.67678	-1.63446	0.006586	0.035079	1695	- && - && PF01485:IBR domain, a half RING-finger domain

Lachesis_group0__13_contigs__l.g3044	134.3459	416.99	-1.63448	0.003835	0.023875	1395	- && Q00023.1 RecName: Full=Cellulose-growth-specific protein; Flags: Precursor && PF00734:Fungal cellulose binding domain|PF03443:Glycosyl hydrolase family 61
Lachesis_group0__13_contigs__l.g3063	1750.931	5437.637	-1.63491	0.000141	0.002307	708	- && Q7ZV68.1 RecName: Full=Vacuolar protein sorting-associated protein 29; AltName: Full=Vesicle protein sorting 29 && PF12850:Calcineurin-like phosphoesterase superfamily domain
Lachesis_group0__13_contigs__l.g1534	535.0431	1661.87	-1.63549	0.000304	0.003896	5208	- && Q9UUG9.1 RecName: Full=Tuberous sclerosis 2 protein homolog && PF03542:Tuberin|PF02145:Rap/ran-GAP
Lachesis_group0__13_contigs__l.g1860	495.9817	1543.494	-1.63786	0.00194	0.014784	1386	- && Q4WAZ6.1 RecName: Full=Multifunctional cytochrome P450 monooxygenase af510; AltName: Full=Fumagillin bioynthesis cluster P450 monooxygenase; Short=Fma-P450 && PF00067:Cytochrome P450
Lachesis_group0__13_contigs__l.g4565	51.98305	162.2434	-1.64179	0.001197	0.010422	1416	- && Q99252.1 RecName: Full=Protein ECM3; AltName: Full=Extracellular mutant protein 3 && PF03547:Membrane transport protein
Lachesis_group0__13_contigs__l.g5745	294.2804	918.9096	-1.6428	0.006499	0.034749	1056	- && - && PF01753:MYND finger
Lachesis_group0__13_contigs__l.g2884	996.4632	3121.04	-1.6472	0.000773	0.00765	1236	- && Q9Y7K9.3 RecName: Full=PARP-type zinc finger-containing protein C2A9.07c && PF00645:Poly(ADP-ribose) polymerase and DNA-Ligase Zn-finger region
Lachesis_group0__13_contigs__l.g5954	386.6316	1215.974	-1.65309	0.000338	0.004198	702	- && Q6PAV8.1 RecName: Full=O-acetyl-ADP-ribose deacetylase MACROD2; AltName: Full=MACRO domain-containing protein 2;

							AltName: Full=[Protein ADP-ribosylglutamate] hydrolase && PF01661:Macro domain
Lachesis_group0__13_contigs__l.g306	2362.886	7436.523	-1.65411	0.006211	0.033738	4530	- && Q54YW1.1 RecName: Full=ELMO domain-containing protein A && PF04727:ELMO/CED-12 family|PF16457:Pleckstrin homology domain|PF11841:Domain of unknown function (DUF3361)
Lachesis_group0__13_contigs__l.g8411	48.22369	151.4908	-1.65417	0.003106	0.020536	2010	- && P54718.1 RecName: Full=Uncharacterized ABC transporter ATP-binding protein YfiB && PF00005:ABC transporter
Lachesis_group0__13_contigs__l.g2059	109.8145	347.2882	-1.66247	0.000566	0.006111	1734	- && P70786.1 RecName: Full=Putative tartrate transporter && PF07690:Major Facilitator Superfamily
Lachesis_group0__13_contigs__l.g9589	30.87511	97.73225	-1.66336	0.003284	0.021404	819	- && B5ZA76.1 RecName: Full=Peptidoglycan deacetylase; Short=PG deacetylase; AltName: Full=Acetylxylan esterase && PF01522:Polysaccharide deacetylase
Lachesis_group0__13_contigs__l.g5928	1702.076	5402.421	-1.66641	0.000173	0.002621	525	- && B0DX25.1 RecName: Full=Type 1 phosphatases regulator YPI1 && PF07491:Protein phosphatase inhibitor
Lachesis_group0__13_contigs__l.g599	158.1833	501.8577	-1.66676	0.003239	0.021183	765	- && O14283.2 RecName: Full=Transcription factor prr1; AltName: Full=Pombe response regulator 1 && PF00447:HSF-type DNA-binding
Lachesis_group0__13_contigs__l.g10701	65.65139	208.8641	-1.66999	0.001505	0.012249	2664	- && Q09803.1 RecName: Full=Suppressor protein of bem1/bed5 double mutants && PF04212:MIT (microtubule interacting and transport) domain|PF07714:Protein tyrosine kinase

Lachesis_group0__13_contigs__l.g6778	29.85971	94.85418	-1.67027	0.006322	0.034179	813	- && - && PF13527:Acetyltransferase (GNAT) domain
Lachesis_group0__13_contigs__l.g147	329.7295	1050.044	-1.67091	0.00054	0.005915	1377	- && Q9UT44.1 RecName: Full=Probable gamma-glutamyl phosphate reductase; Short=GPR; AltName: Full=Glutamate-5-semialdehyde dehydrogenase; Short=GSA dehydrogenase; AltName: Full=Glutamyl-gamma-semialdehyde dehydrogenase && PF00171:Aldehyde dehydrogenase family
Lachesis_group0__13_contigs__l.g6783	51.03863	162.7201	-1.67245	0.001023	0.009423	1344	- && - && PF13472:GDSL-like Lipase/Acylhydrolase family
Lachesis_group0__13_contigs__l.g9163	88.36513	281.9524	-1.67544	0.000856	0.008291	4614	- && - && PF02373:JmjC domain, hydroxylase
Lachesis_group0__13_contigs__l.g2380	80.95633	258.4653	-1.67738	0.006737	0.035676	1374	- && O34714.1 RecName: Full=Oxalate decarboxylase OxdC && PF00190:Cupin
Lachesis_group0__13_contigs__l.g4553	398.2497	1280.51	-1.68498	0.000143	0.002314	786	- && D2WKD9.2 RecName: Full=Farnesol dehydrogenase; AltName: Full=NADP+-dependent farnesol dehydrogenase 1; Short=AaSDR-1 && PF00106:short chain dehydrogenase
Lachesis_group0__13_contigs__l.g8868	311.3524	1001.358	-1.68558	0.005855	0.032318	1788	- && P49075.1 RecName: Full=Exoglucanase 3; AltName: Full=1,4-beta-cellobiohydrolase 3; AltName: Full=Exocellobiohydrolase 3; Flags: Precursor && PF00734:Fungal cellulose binding domain|PF01341:Glycosyl hydrolases family 6
Lachesis_group0__13_contigs__l.g2608	28.44312	91.73695	-1.68857	0.005203	0.029761	969	- && B0Y081.2 RecName: Full=Metacaspase-1B; Flags: Precursor >Q4WYT0.2 RecName: Full=Metacaspase-1B; Flags: Precursor && PF00656:Caspase domain

Lachesis_group0__13_contigs__l.g4367	34.67506	111.8782	-1.69235	0.002843	0.019333	3351	- && B8NHY4.1 RecName: Full=O-methylsterigmatocystin oxidoreductase; Short=OMST oxidoreductase; AltName: Full=Aflatoxin B synthase; AltName: Full=Aflatoxin biosynthesis protein Q; AltName: Full=Cytochrome P450 64 >P0CT93.1 RecName: Full=O-methylsterigmatocystin oxidoreductase; Short=OMST oxidoreductase; AltName: Full=Aflatoxin B synthase; AltName: Full=Aflatoxin biosynthesis protein Q; AltName: Full=Cytochrome P450 64 && PF00067:Cytochrome P450
Lachesis_group0__13_contigs__l.g5941	123.6543	400.6971	-1.69694	0.001347	0.011328	1608	- && Q4U3U4.1 RecName: Full=Quinate permease; AltName: Full=Quinate transporter && PF00083:Sugar (and other) transporter
Lachesis_group0__13_contigs__l.g11140	258.7725	839.9504	-1.69891	7.67E-05	0.001499	2277	- && - && PF02838:Glycosyl hydrolase family 20, domain 2
Lachesis_group0__13_contigs__l.g8161	224.2273	728.1661	-1.69949	0.005577	0.031189	3516	- && F4JTP5.1 RecName: Full=Serine/threonine-protein kinase STY46; AltName: Full=Serine/threonine/tyrosine-protein kinase 46 && PF07714:Protein tyrosine kinase
Lachesis_group0__13_contigs__l.g8302	34.54365	112.0839	-1.70044	0.002912	0.01963	1815	- && Q92341.1 RecName: Full=Siderophore iron transporter 3 && PF07690:Major Facilitator Superfamily
Lachesis_group0__13_contigs__l.g11329	309.7604	1006.628	-1.70102	0.000439	0.005097	1542	- && P09437.2 RecName: Full=Cytochrome b2, mitochondrial; AltName: Full=L-lactate dehydrogenase [Cytochrome]; AltName: Full=L-lactate ferricytochrome C oxidoreductase; Short=L-

							LCR; Flags: Precursor && PF00173:Cytochrome b5-like Heme/Steroid binding domain|PF01070:FMN-dependent dehydrogenase
Lachesis_group0__13_contigs__l.g8618	36.09146	117.1632	-1.70232	0.002403	0.01724	1419	- && P49374.1 RecName: Full=High-affinity glucose transporter && PF00083:Sugar (and other) transporter
Lachesis_group0__13_contigs__l.g640	369.4147	1204.059	-1.70493	0.005057	0.029147	1101	- && Q4WR80.1 RecName: Full=Probable endopolygalacturonase AFUA_1G17220; AltName: Full=Pectinase AFUA_1G17220; AltName: Full=Polygalacturonase AFUA_1G17220; Flags: Precursor >B0XPA1.1 RecName: Full=Probable endopolygalacturonase AFUB_016610; AltName: Full=Pectinase AFUB_016610; AltName: Full=Polygalacturonase AFUB_016610; Flags: Precursor && PF00295:Glycosyl hydrolases family 28
Lachesis_group0__13_contigs__l.g7082	799.2323	2607.005	-1.70592	0.00252	0.01774	1368	- && Q8RWZ3.1 RecName: Full=Probable acyl-CoA dehydrogenase IBR3; AltName: Full=Protein INDOLE-3-BUTYRIC ACID RESPONSE 3 && PF00441:Acyl-CoA dehydrogenase, C-terminal domain|PF02770:Acyl-CoA dehydrogenase, middle domain|PF02771:Acyl-CoA dehydrogenase, N-terminal domain
Lachesis_group0__13_contigs__l.g2364	47.07221	153.4196	-1.70654	0.002827	0.019264	2409	- && - && PF12937:F-box-like
Lachesis_group0__13_contigs__l.g1403	280.6081	916.0659	-1.70679	0.002694	0.018588	1425	- && P50080.1 RecName: Full=Azole resistance protein 1 && PF07690:Major Facilitator Superfamily

Lachesis_group0__13_contigs__l.g765	238.9406	781.3715	-1.7094	0.000847	0.008255	1395	- && P25390.2 RecName: Full=Serine/threonine-protein kinase SSK22; AltName: Full=MAP kinase kinase kinase SSK22; AltName: Full=Suppressor of sensor kinase 22 && PF07714:Protein tyrosine kinase
Lachesis_group0__13_contigs__l.g7993	1759.614	5757.148	-1.71017	0.00016	0.002507	582	- && - && PF01476:LysM domain
Lachesis_group0__13_contigs__l.g8715	10212.33	33421.77	-1.71048	0.002204	0.016142	3030	- && Q9ULC5.1 RecName: Full=Long-chain-fatty-acid--CoA ligase 5; AltName: Full=Long-chain acyl-CoA synthetase 5; Short=LACS 5 && PF13499:EF-hand domain pair|PF00501:AMP-binding enzyme
Lachesis_group0__13_contigs__l.g654	150.1421	492.7679	-1.71384	0.002888	0.019541	2025	- && Q54DY9.1 RecName: Full=Probable mitochondrial chaperone BCS1-B; AltName: Full=BCS1-like protein 2 && PF00004:ATPase family associated with various cellular activities (AAA)|PF08740:BCS1 N terminal
Lachesis_group0__13_contigs__l.g2600	1065.86	3500.331	-1.7155	0.001093	0.009864	2562	- && Q54DU5.1 RecName: Full=von Willebrand factor A domain-containing protein DDB_G0292028 && PF13768:von Willebrand factor type A domain|PF08487:Vault protein inter-alpha-trypsin domain
Lachesis_group0__13_contigs__l.g11654	2965.687	9765.142	-1.7193	0.001669	0.013139	1098	- && Q9ZWQ9.1 RecName: Full=Flavonol synthase/flavanone 3-hydroxylase; AltName: Full=CitFLS; Short=FLS && PF03171:2OG-Fe(II) oxygenase superfamily|PF14226:non-haem dioxygenase in morphine synthesis N-terminal
Lachesis_group0__13_contigs__l.g7954	115.2407	380.6274	-1.72442	0.000148	0.002366	3099	- && Q13546.3 RecName: Full=Receptor-interacting serine/threonine-protein kinase 1; AltName: Full=Cell death protein RIP; AltName:

							Full=Receptor-interacting protein 1; Short=RIP-1; AltName: Full=Serine/threonine-protein kinase RIP && PF07714:Protein tyrosine kinase
Lachesis_group0__13_contigs__l.g1026	150.3457	496.8954	-1.72507	4.57E-05	0.001008	1722	- && Q86UW6.2 RecName: Full=NEDD4-binding protein 2; Short=N4BP2; AltName: Full=BCL-3-binding protein && PF08590:Domain of unknown function (DUF1771)|PF01713:Smr domain
Lachesis_group0__13_contigs__l.g9920	170.4277	563.4348	-1.72602	0.00144	0.011839	1443	- && A2QEQ6.1 RecName: Full=Beta-glucuronidase; Short=GlcAase; AltName: Full=Beta-D-glucuronoside glucuronosohydrolase; Flags: Precursor && PF16862:Glycosyl hydrolase family 79 C-terminal beta domain
Lachesis_group0__13_contigs__l.g721	217.2972	719.0553	-1.72631	0.002079	0.015527	2298	- && O22881.2 RecName: Full=Potassium transporter 2; Short=AtKT2; Short=AtKUP2; Short=AtPOT2 && PF02705:K+ potassium transporter
Lachesis_group0__13_contigs__l.g1471	364.5472	1206.151	-1.7264	0.000295	0.003822	1383	- && - && PF00107:Zinc-binding dehydrogenase|PF16884:N-terminal domain of oxidoreductase
Lachesis_group0__13_contigs__l.g10687	2169.058	7183.051	-1.72752	0.000749	0.007473	1050	- && Q8YVU6.2 RecName: Full=Porphobilinogen deaminase; Short=PBG; AltName: Full=Hydroxymethylbilane synthase; Short=HMBS; AltName: Full=Pre-uroporphyrinogen synthase && PF01379:Porphobilinogen deaminase, dipyromethane cofactor binding domain|PF03900:Porphobilinogen deaminase, C-terminal domain

Lachesis_group0__13_contigs__l.g612	153.9941	510.0957	-1.7284	0.002426	0.017307	4908	- && Q9USW3.1 RecName: Full=Probable glycosidase C21B10.07 && PF00501:AMP-binding enzyme|PF07993:Male sterility protein
Lachesis_group0__13_contigs__l.g1287	116.9258	388.5931	-1.73316	0.000225	0.003174	615	- && - && PF01822:WSC domain
Lachesis_group0__13_contigs__l.g10407	51343.9	170785.5	-1.73392	0.000224	0.003166	432	- && D4ALV6.1 RecName: Full=Allergen Asp f 15 homolg && PF07249:Cerato-platanin
Lachesis_group0__13_contigs__l.g11243	1289.256	4298.225	-1.73728	0.000581	0.006202	552	- && - && PF13424:Tetratricopeptide repeat|PF01753:MYND finger
Lachesis_group0__13_contigs__l.g8405	411.9513	1373.782	-1.73777	0.000292	0.003794	3213	- && O14283.2 RecName: Full=Transcription factor prr1; AltName: Full=Pombe response regulator 1 && PF00447:HSF-type DNA-binding
Lachesis_group0__13_contigs__l.g3882	320.6456	1070.666	-1.74005	0.000516	0.005715	1389	- && O15975.1 RecName: Full=Guanine nucleotide-binding protein G(q) subunit alpha; AltName: Full=Guanine nucleotide-binding protein alpha-q && PF00503:G-protein alpha subunit
Lachesis_group0__13_contigs__l.g2222	183.0485	611.4348	-1.74077	0.00173	0.01352	1158	- && Q01752.1 RecName: Full=Aryl-alcohol dehydrogenase [NADP(+)]; Short=AAD && PF00248:Aldo/keto reductase family
Lachesis_group0__13_contigs__l.g11048	33.30329	111.0554	-1.74091	0.003585	0.022785	1527	- && Q9P6J0.1 RecName: Full=Uncharacterized transporter C1683.12 && PF07690:Major Facilitator Superfamily
Lachesis_group0__13_contigs__l.g7662	91.95722	307.6533	-1.74263	0.00051	0.005684	2667	- && P08159.2 RecName: Full=6-hydroxy-D-nicotine oxidase; Short=6-HDNO && PF08031:Berberine and berberine like|PF01565:FAD binding domain
Lachesis_group0__13_contigs__l.g10893	118.131	396.2802	-1.7472	0.002617	0.018161	1092	- && Q4X084.1 RecName: Full=Probable endo-1,3(4)-beta-glucanase AFUA_2G14360; AltName: Full=Mixed-linked glucanase AFUA_2G14360;

							Flags: Precursor >B0XTU6.1 RecName: Full=Probable endo-1,3(4)-beta-glucanase AFUB_029980; AltName: Full=Mixed-linked glucanase AFUB_029980; Flags: Precursor && -
Lachesis_group0__13_contigs__l.g990	161.729	543.014	-1.74807	0.001934	0.014751	2844	- && Q4WAZ6.1 RecName: Full=Multifunctional cytochrome P450 monooxygenase af510; AltName: Full=Fumagillin bioynthesis cluster P450 monooxygenase; Short=Fma-P450 && PF00067:Cytochrome P450
Lachesis_group0__13_contigs__l.g5160	1672.295	5619.229	-1.7486	0.000171	0.002595	990	- && O23300.1 RecName: Full=Enoyl-CoA delta isomerase 3; AltName: Full=3-hydroxyacyl-CoA dehydratase 1; AltName: Full=Delta(3),Delta(2)-enoyl CoA isomerase 3; Short=AtECI3 && PF00378:Enoyl-CoA hydratase/isomerase
Lachesis_group0__13_contigs__l.g4424	3446.692	11592.64	-1.74994	0.0059	0.032481	3249	- && S0DXJ2.1 RecName: Full=Non-canonical non-ribosomal peptide synthetase FUB8; AltName: Full=Fusaric acid biosynthesis protein 8 && PF07993:Male sterility protein|PF00501:AMP-binding enzyme
Lachesis_group0__13_contigs__l.g1982	2277.492	7669.161	-1.75163	0.000146	0.002354	330	- && - && PF12585:Protein of unknown function (DUF3759)
Lachesis_group0__13_contigs__l.g2202	76.16464	256.2304	-1.75201	0.001497	0.012219	1566	- && O60020.1 RecName: Full=Aspartic protease; Flags: Precursor && PF00026:Eukaryotic aspartyl protease
Lachesis_group0__13_contigs__l.g8672	21.29457	71.69382	-1.75349	0.009522	0.045567	1674	- && P42251.3 RecName: Full=Alkaline phosphatase D; Short=APaseD; Flags: Precursor && PF09423:PhoD-like

							phosphatase|PF16655:PhoD-like phosphatase, N-terminal domain
Lachesis_group0__13_contigs__l.g4981	14868.46	50149.94	-1.754	6.86E-05	0.001374	4215	- && Q7S4N5.2 RecName: Full=Metacaspase-1B; Flags: Precursor && PF00656:Caspase domain
Lachesis_group0__13_contigs__l.g10410	2017.249	6807.035	-1.75468	0.000907	0.008631	564	- && - && PF06687:SUR7/PalI family
Lachesis_group0__13_contigs__l.g11203	267.3413	904.0662	-1.75763	0.000162	0.002523	681	- && - && PF01975:Survival protein SurE
Lachesis_group0__13_contigs__l.g9149	1191.161	4030.808	-1.75872	0.000716	0.007231	2418	- && Q9CPY6.1 RecName: Full=Glucose-induced degradation protein 4 homolog; AltName: Full=Vacuolar import and degradation protein 24 homolog && PF09783:Vacuolar import and degradation protein
Lachesis_group0__13_contigs__l.g11236	3802.636	12870.01	-1.75896	0.009569	0.045703	2700	- && O94641.1 RecName: Full=Heat shock protein 104; AltName: Full=Protein aggregation-remodeling factor hsp104 && PF07724:AAA domain (Cdc48 subfamily)|PF00004:ATPase family associated with various cellular activities (AAA)|PF10431:C-terminal, D2-small domain, of ClpB protein|PF02861:Clp amino terminal domain, pathogenicity island component
Lachesis_group0__13_contigs__l.g107	700.0187	2370.923	-1.76008	0.000527	0.0058	933	- && - && PF04479:RTA1 like protein
Lachesis_group0__13_contigs__l.g590	1742.484	5907.971	-1.76156	4.49E-05	0.001	5571	- && Q5B4Z3.2 RecName: Full=Cytokinesis protein sepH; AltName: Full=Serine/threonine-protein kinase sepH && PF00069:Protein kinase domain|PF00307:Calponin homology (CH) domain|PF00130:Phorbol esters/diacylglycerol binding domain (C1 domain)

Lachesis_group0__13_contigs__l.g7705	263.5242	896.0849	-1.76582	0.005757	0.031902	1035	- && Q568Z6.1 RecName: Full=IST1 homolog && PF03398:Regulator of Vps4 activity in the MVB pathway
Lachesis_group0__13_contigs__l.g6467	530.2749	1805.231	-1.76784	0.001516	0.01231	1848	- && Q3KJ31.1 RecName: Full=Lipid A export ATP-binding/permease protein MsbA && PF00005:ABC transporter
Lachesis_group0__13_contigs__l.g4384	45.56565	154.9898	-1.76837	0.002946	0.019738	1506	- && Q4WAZ6.1 RecName: Full=Multifunctional cytochrome P450 monooxygenase af510; AltName: Full=Fumagillin bioynthesis cluster P450 monooxygenase; Short=Fma-P450 && PF00067:Cytochrome P450
Lachesis_group0__13_contigs__l.g8968	57.57635	196.5692	-1.77233	0.000296	0.003824	1074	- && Q96HA8.2 RecName: Full=Protein N-terminal glutamine amidohydrolase; AltName: Full=Protein NH2-terminal glutamine deamidase; Short=N-terminal Gln amidase; Short=Nt(Q)-amidase; AltName: Full=WDYHV motif-containing protein 1 && PF09764:N-terminal glutamine amidase
Lachesis_group0__13_contigs__l.g3623	481.9576	1646.493	-1.77279	0.001008	0.009329	3006	- && Q7XSA2.3 RecName: Full=Protein argonaute 1B; Short=OsAGO1b && PF16488:Argonaute linker 2 domain|PF08699:Argonaute linker 1 domain|PF02170:PAZ domain|PF16486:N-terminal domain of argonaute|PF02171:Piwi domain
Lachesis_group0__13_contigs__l.g4410	28.28577	96.642	-1.77622	0.001465	0.012009	2118	- && - && PF00076:RNA recognition motif. (a.k.a. RRM, RBD, or RNP domain)
Lachesis_group0__13_contigs__l.g10183	1175.401	4032.768	-1.77857	8.57E-06	0.000305	1815	- && Q7SEY9.1 RecName: Full=Endoplasmic reticulum oxidoreductin-1; Flags: Precursor &&

							PF04137:Endoplasmic Reticulum Oxidoreductin 1 (ERO1)
Lachesis_group0__13_contigs__l.g11122	126.046	432.7533	-1.77934	0.000115	0.001995	1677	- && P47013.1 RecName: Full=Dihydrosphingosine 1-phosphate phosphatase LCB3; AltName: Full=Long-chain base protein 3; AltName: Full=Sphingolipid resistance protein 2 && PF01569:PAP2 superfamily
Lachesis_group0__13_contigs__l.g10242	72.85642	249.7439	-1.77959	0.001144	0.010132	1716	- && P39992.1 RecName: Full=Uncharacterized protein YEL023C && PF09994:Uncharacterized alpha/beta hydrolase domain (DUF2235)
Lachesis_group0__13_contigs__l.g8721	4836.358	16607.18	-1.77982	0.000463	0.005295	1617	- && P25390.2 RecName: Full=Serine/threonine-protein kinase SSK22; AltName: Full=MAP kinase kinase kinase SSK22; AltName: Full=Suppressor of sensor kinase 22 && PF00069:Protein kinase domain
Lachesis_group0__13_contigs__l.g716	187.9779	645.2224	-1.77986	0.001917	0.014684	1473	- && - && PF01266:FAD dependent oxidoreductase
Lachesis_group0__13_contigs__l.g1248	107.6739	369.6749	-1.78154	0.00131	0.011127	2037	- && - && PF05592:Bacterial alpha-L-rhamnosidase
Lachesis_group0__13_contigs__l.g2843	6888.139	23705.37	-1.78304	0.000289	0.003783	1518	- && Q9URW6.1 RecName: Full=SH3 domain-containing protein PJ696.02 && PF04366:Las17-binding protein actin regulator|PF00018:SH3 domain
Lachesis_group0__13_contigs__l.g8835	7480.133	25744.65	-1.78315	0.00104	0.009549	849	- && O74631.1 RecName: Full=Protein FDD123; AltName: Full=CvHSP30/1 && PF01036:Bacteriorhodopsin-like protein
Lachesis_group0__13_contigs__l.g568	17911.03	61681.03	-1.78399	0.000237	0.00327	1626	- && Q12300.1 RecName: Full=High-affinity glucose transporter RGT2 && PF00083:Sugar (and other) transporter

Lachesis_group0__13_contigs__l.g8885	1569.196	5416.661	-1.78751	0.001587	0.012681	1230	- && P43066.1 RecName: Full=D-arabinitol 2-dehydrogenase [ribulose-forming]; Short=ARDH && PF00106:short chain dehydrogenase
Lachesis_group0__13_contigs__l.g5514	155.8169	537.8118	-1.78825	0.00386	0.02399	840	- && - && PF09458:H-type lectin domain
Lachesis_group0__13_contigs__l.g7481	1609.748	5564.417	-1.78939	0.000126	0.002118	2475	- && - && PF04082:Fungal specific transcription factor domain
Lachesis_group0__13_contigs__l.g3662	14.38419	49.67729	-1.79012	0.006933	0.036474	798	- && - && PF11807:Domain of unknown function (DUF3328)
Lachesis_group0__13_contigs__l.g11176	4616.09	15971.71	-1.79078	0.000535	0.005862	627	- && Q0JL44.1 RecName: Full=Protein SGT1 homolog; Short=OsSGT1; AltName: Full=Suppressor of G2 allele of SKP1 homolog && PF04969:CS domain|PF05002:SGS domain
Lachesis_group0__13_contigs__l.g6780	656.1662	2271.081	-1.79159	0.009375	0.045147	1959	- && P30608.1 RecName: Full=Cytochrome P450 52A6; AltName: Full=Alkane-inducible P450-ALK3; AltName: Full=CYPLIIA6 && PF00067:Cytochrome P450
Lachesis_group0__13_contigs__l.g5914	3374.728	11685.59	-1.7919	0.000227	0.003183	3783	- && Q5BGR2.2 RecName: Full=Protein mesA && PF07792:Docking domain of Afi1 for Arf3 in vesicle trafficking|PF00656:Caspase domain
Lachesis_group0__13_contigs__l.g5624	1067.04	3708.105	-1.79714	0.000604	0.006368	1332	- && Q9P6P9.1 RecName: Full=[Pyruvate dehydrogenase (acetyl-transferring)] kinase, mitochondrial; Short=Pyruvate dehydrogenase kinase; Flags: Precursor && PF10436:Mitochondrial branched-chain alpha-ketoacid dehydrogenase kinase|PF02518:Histidine kinase-, DNA gyrase B-, and HSP90-like ATPase
Lachesis_group0__13_contigs__l.g2699	82.39893	286.9797	-1.8016	0.002225	0.016226	1923	- && Q8NK92.2 RecName: Full=Aorsin; Flags: Precursor && PF00082:Subtilase

							family|PF09286:Pro-kumamolisin, activation domain
Lachesis_group0__13_contigs__l.g1720	39.69642	138.8362	-1.80571	0.001124	0.010042	885	- && B8NM67.1 RecName: Full=Oxidase ustYa; AltName: Full=Ustiloxin B biosynthesis protein Ya && PF11807:Domain of unknown function (DUF3328)
Lachesis_group0__13_contigs__l.g3604	927.4681	3286.048	-1.82501	0.004827	0.028238	1461	- && P53322.1 RecName: Full=High-affinity nicotinic acid transporter; AltName: Full=Nicotinic acid permease && PF07690:Major Facilitator Superfamily
Lachesis_group0__13_contigs__l.g3589	42.75671	151.2488	-1.82516	0.003573	0.02276	3213	- && A0A0D2YG01.1 RecName: Full=Non-canonical non-ribosomal peptide synthetase FUB8; AltName: Full=Fusaric acid biosynthesis protein 8 && PF07993:Male sterility protein|PF00501:AMP-binding enzyme
Lachesis_group0__13_contigs__l.g4534	191.4885	679.0501	-1.82705	2.98E-05	0.000733	1710	- && P45598.1 RecName: Full=Arabinose-proton symporter; AltName: Full=Arabinose transporter && PF00083:Sugar (and other) transporter
Lachesis_group0__13_contigs__l.g5452	4143.429	14732.66	-1.83015	8.21E-05	0.001572	1326	- && - && PF03547:Membrane transport protein
Lachesis_group0__13_contigs__l.g993	59.54099	213.8768	-1.84645	0.001286	0.011008	1593	- && B8NHY4.1 RecName: Full=O-methylsterigmatocystin oxidoreductase; Short=OMST oxidoreductase; AltName: Full=Aflatoxin B synthase; AltName: Full=Aflatoxin biosynthesis protein Q; AltName: Full=Cytochrome P450 64 >P0CT93.1 RecName: Full=O-methylsterigmatocystin oxidoreductase; Short=OMST oxidoreductase; AltName: Full=Aflatoxin B synthase; AltName:

							Full=Aflatoxin biosynthesis protein Q; AltName: Full=Cytochrome P450 64 && PF00067:Cytochrome P450
Lachesis_group0__13_contigs__l.g2685	231.6845	833.303	-1.84723	0.000119	0.002032	897	- && Q1ZXF1.1 RecName: Full=Probable enoyl-CoA hydratase, mitochondrial; AltName: Full=Enoyl-CoA hydratase 1; AltName: Full=Short-chain enoyl-CoA hydratase; Short=SCEH; Flags: Precursor && PF00378:Enoyl-CoA hydratase/isomerase
Lachesis_group0__13_contigs__l.g239	37.96665	136.7035	-1.8473	0.000868	0.008363	2805	- && Q9P3U4.1 RecName: Full=E3 ubiquitin-protein ligase dbl4; AltName: Full=DNA-break-localizing protein 4; AltName: Full=Histone E3 ligase 1 && PF01485:IBR domain, a half RING-finger domain|PF13445:RING-type zinc-finger
Lachesis_group0__13_contigs__l.g7299	2733.998	9842.929	-1.8481	0.000237	0.00327	1920	- && P56859.3 RecName: Full=Phosphoadenosine phosphosulfate reductase; AltName: Full=3'-phosphoadenylylsulfate reductase; AltName: Full=PAPS reductase, thioredoxin dependent; AltName: Full=PAdoPS reductase && PF01507:Phosphoadenosine phosphosulfate reductase family|PF14622:Ribonuclease-III-like|PF00035:Double-stranded RNA binding motif
Lachesis_group0__13_contigs__l.g5617	82.30664	296.0294	-1.8485	0.00061	0.006407	939	- && P02723.1 RecName: Full=ADP,ATP carrier protein; AltName: Full=ADP/ATP translocase; AltName: Full=Adenine nucleotide translocator; Short=ANT && PF00153:Mitochondrial carrier protein
Lachesis_group0__13_contigs__l.g5278	1698.928	6124.647	-1.85007	0.000257	0.003454	1635	- && - && PF10277:Frag1/DRAM/Sfk1 family

Lachesis_group0__13_contigs__l.g8800	124.7994	451.3357	-1.85481	0.004491	0.026711	1242	- && Q8NK50.1 RecName: Full=NADP-dependent mannitol dehydrogenase; Short=MtDH; AltName: Full=Mannitol 2-dehydrogenase [NADP(+)] && PF00106:short chain dehydrogenase
Lachesis_group0__13_contigs__l.g5508	167.479	605.7852	-1.85518	0.002879	0.019492	423	- && P33207.2 RecName: Full=3-oxoacyl-[acyl-carrier-protein] reductase, chloroplastic; AltName: Full=3-ketoacyl-acyl carrier protein reductase; Flags: Precursor && PF00106:short chain dehydrogenase
Lachesis_group0__13_contigs__l.g4525	4332.66	15675.92	-1.85525	0.000877	0.008412	3144	- && P00365.4 RecName: Full=NAD-specific glutamate dehydrogenase; Short=NAD-GDH && PF00208:Glutamate/Leucine/Phenylalanine/Valine dehydrogenase|PF05088:Bacterial NAD-glutamate dehydrogenase
Lachesis_group0__13_contigs__l.g6118	134.8196	487.2507	-1.85536	0.002439	0.017366	2061	- && A1CFL2.1 RecName: Full=Dehydrogenase patE; AltName: Full=Patulin synthesis protein E; Flags: Precursor && PF05199:GMC oxidoreductase|PF00732:GMC oxidoreductase
Lachesis_group0__13_contigs__l.g1789	2543.464	9213.51	-1.85691	9.20E-06	0.000324	396	- && - && PF03330:Rare lipoprotein A (RlpA)-like double-psi beta-barrel
Lachesis_group0__13_contigs__l.g8747	167.2373	605.7955	-1.85798	0.000143	0.002314	2403	- && P31434.2 RecName: Full=Alpha-xylosidase && PF13802:Galactose mutarotase-like|PF01055:Glycosyl hydrolases family 31
Lachesis_group0__13_contigs__l.g5618	248.7932	913.5809	-1.87683	0.002303	0.016668	1179	- && P77316.1 RecName: Full=Uncharacterized zinc-type alcohol dehydrogenase-like protein YbdR && PF08240:Alcohol dehydrogenase GroES-like domain

Lachesis_group0__13_contigs__l.g3352	155.5561	571.8441	-1.87815	4.78E-05	0.001036	1416	- && A1CTI3.1 RecName: Full=Probable glucan 1,3-beta-glucosidase D; AltName: Full=Exo-1,3-beta-glucanase D && PF00150:Cellulase (glycosyl hydrolase family 5)
Lachesis_group0__13_contigs__l.g6708	149.6527	549.9739	-1.87847	2.59E-05	0.000673	1128	- && A1CFY8.2 RecName: Full=Probable D-xylulose reductase A; AltName: Full=Xylitol dehydrogenase A && PF08240:Alcohol dehydrogenase GroES-like domain|PF00107:Zinc-binding dehydrogenase
Lachesis_group0__13_contigs__l.g2771	135.5901	498.2812	-1.87893	0.002222	0.016226	1935	- && Q8NK92.2 RecName: Full=Aorsin; Flags: Precursor && PF09286:Pro-kumamolisin, activation domain
Lachesis_group0__13_contigs__l.g11221	49.29165	181.1953	-1.88072	0.001407	0.011654	1029	- && Q8Y014.1 RecName: Full=Transaldolase && PF00923:Transaldolase
Lachesis_group0__13_contigs__l.g3733	19013.08	70136.46	-1.88318	5.29E-05	0.001121	3861	- && O74969.1 RecName: Full=High-affinity glucose transporter ght2; AltName: Full=Hexose transporter 2 && PF00083:Sugar (and other) transporter
Lachesis_group0__13_contigs__l.g4969	60.9153	224.8246	-1.88549	0.000572	0.006149	1077	- && - && PF05368:NmrA-like family
Lachesis_group0__13_contigs__l.g7330	62.98146	232.9647	-1.88852	5.50E-05	0.001155	1320	- && A1DDK1.1 RecName: Full=Aspartic protease pep1; AltName: Full=Aspergillopepsin A; AltName: Full=Aspergillopepsin I; Flags: Precursor && PF00026:Eukaryotic aspartyl protease
Lachesis_group0__13_contigs__l.g1568	35.96981	133.2865	-1.89158	0.004141	0.025271	894	- && - && PF08719:Domain of unknown function (DUF1768)
Lachesis_group0__13_contigs__l.g483	32.67339	121.5727	-1.89299	0.001429	0.011791	1689	- && - && PF12937:F-box-like

Lachesis_group0__13_contigs__l.g520	741.41	2768.952	-1.90115	0.000687	0.007031	957	- && O06179.1 RecName: Full=Putative monooxygenase Rv1533 && PF03060:Nitronate monooxygenase
Lachesis_group0__13_contigs__l.g5294	31.5608	117.8237	-1.90239	0.006646	0.035345	975	- && - && PF05577:Serine carboxypeptidase S28
Lachesis_group0__13_contigs__l.g10111	1256.738	4705.777	-1.9048	0.00247	0.017509	3831	- && - && PF00004:ATPase family associated with various cellular activities (AAA)
Lachesis_group0__13_contigs__l.g4931	123.2609	461.9363	-1.90568	0.000229	0.003201	834	- && - && PF12695:Alpha/beta hydrolase family
Lachesis_group0__13_contigs__l.g845	2472.127	9270.988	-1.90698	0.001142	0.010125	4587	- && - && PF08229:ER membrane protein SH3
Lachesis_group0__13_contigs__l.g7326	27470.31	103090.3	-1.90797	0.004377	0.026243	1152	- && C5DGQ7.1 RecName: Full=Glucose transport transcription regulator RGT1; AltName: Full=Restores glucose transport protein 1 && PF00172:Fungal Zn(2)-Cys(6) binuclear cluster domain
Lachesis_group0__13_contigs__l.g6578	3711.361	13937.14	-1.90896	0.000116	0.002001	3000	- && - && PF06011:Transient receptor potential (TRP) ion channel
Lachesis_group0__13_contigs__l.g677	117.1036	444.3199	-1.9248	0.002506	0.01767	1878	- && P15245.3 RecName: Full=Phenol 2-monooxygenase; AltName: Full=Phenol hydroxylase && PF07976:Phenol hydroxylase, C-terminal dimerisation domain|PF01494:FAD binding domain
novel.1009	33.17595	126.0126	-1.92842	0.00146	0.011971	2302	- && P15365.1 RecName: Full=Allantoate permease && PF07690:Major Facilitator Superfamily
Lachesis_group0__13_contigs__l.g5495	34.41778	131.3241	-1.93283	0.00259	0.018043	903	- && P14065.1 RecName: Full=Glycerol 2-dehydrogenase (NADP(+)); AltName: Full=Galactose-inducible crystallin-like protein 1 && PF00248:Aldo/keto reductase family

Lachesis_group0__13_contigs__l.g268	361.8112	1383.513	-1.93496	8.85E-05	0.001664	945	- && O74959.1 RecName: Full=Uncharacterized oxidoreductase C736.13 && PF00106:short chain dehydrogenase
Lachesis_group0__13_contigs__l.g710	42.12905	161.1	-1.93681	0.003106	0.020536	2427	- && - && PF00069:Protein kinase domain
Lachesis_group0__13_contigs__l.g5913	90.33187	346.4283	-1.9389	0.002461	0.017484	1671	- && Q0D076.1 RecName: Full=Probable mannosyl-oligosaccharide alpha-1,2-mannosidase 1B; AltName: Full=Class I alpha-mannosidase 1B; AltName: Full=Man(9)-alpha-mannosidase 1B; Flags: Precursor && PF01532:Glycosyl hydrolase family 47
Lachesis_group0__13_contigs__l.g10730	85.44555	327.3894	-1.93931	0.000443	0.005129	2748	- && Q2U4L7.2 RecName: Full=Glutaminase A; Flags: Precursor && PF08760:Domain of unknown function (DUF1793)|PF16335:Domain of unknown function (DUF4965)
Lachesis_group0__13_contigs__l.g2499	13.28075	50.70783	-1.94022	0.006352	0.034284	765	- && Q9FSG7.1 RecName: Full=Thaumatin-like protein 1a; AltName: Full=Mdtl1; AltName: Full=Pathogenesis-related protein 5a; Short=PR-5a; AltName: Allergen=Mal d 2; Flags: Precursor && PF00314:Thaumatin family
Lachesis_group0__13_contigs__l.g9307	14414.07	55343.5	-1.94094	6.35E-05	0.001294	1296	- && O94123.1 RecName: Full=Phosphoglycerate kinase && PF00162:Phosphoglycerate kinase
Lachesis_group0__13_contigs__l.g722	1290.288	4959.946	-1.94266	0.007935	0.040302	2160	- && Q3L245.1 RecName: Full=Pyranose dehydrogenase 1; Short=PDH 1; AltName: Full=Pyranose:quinone oxidoreductase 1; Flags: Precursor && PF00732:GMC oxidoreductase|PF05199:GMC oxidoreductase
Lachesis_group0__13_contigs__l.g6980	1247.316	4797.317	-1.94347	4.53E-06	0.000196	1572	- && Q0CJ61.1 RecName: Full=Efflux pump atB; AltName: Full=Terreic acid biosynthesis cluster

							protein B && PF07690:Major Facilitator Superfamily
Lachesis_group0__13_contigs__l.g1146	236.4269	909.5683	-1.94372	7.06E-06	0.00027	2244	- && P32816.1 RecName: Full=Glycerol dehydrogenase; Short=GDH; Short=GLDH; Short=GlyDH && PF00465:Iron-containing alcohol dehydrogenase
Lachesis_group0__13_contigs__l.g11192	81.95433	315.0571	-1.94549	0.002848	0.019342	2703	- && - && PF08124:Polysaccharide lyase family 8, N terminal alpha-helical domain|PF02278:Polysaccharide lyase family 8, super-sandwich domain|PF02884:Polysaccharide lyase family 8, C-terminal beta-sandwich domain
Lachesis_group0__13_contigs__l.g10680	3328.394	12877.46	-1.95199	5.39E-05	0.001135	1899	- && Q12581.1 RecName: Full=Cytochrome P450 52A5; AltName: Full=Alkane-inducible P450-ALK2-A; AltName: Full=CYPLIIA5 [Candida maltosa] && PF00067:Cytochrome P450
Lachesis_group0__13_contigs__l.g521	9502.178	36883.88	-1.95667	0.005315	0.030185	783	- && - && PF13920:Zinc finger, C3HC4 type (RING finger)
Lachesis_group0__13_contigs__l.g8408	669.9196	2601.617	-1.95764	0.000999	0.009272	1881	- && O14283.2 RecName: Full=Transcription factor prr1; AltName: Full=Pombe response regulator 1 && PF00447:HSF-type DNA-binding
Lachesis_group0__13_contigs__l.g7165	23.04094	89.4924	-1.9621	0.008895	0.043683	1479	- && - && PF07690:Major Facilitator Superfamily
Lachesis_group0__13_contigs__l.g825	861.6066	3360.719	-1.96368	5.03E-05	0.00107	2187	- && P53933.1 RecName: Full=Phosphatidate phosphatase APP1; Short=PAP; AltName: Full=Actin patch protein 1 && PF09949:Uncharacterized conserved protein (DUF2183)
Lachesis_group0__13_contigs__l.g8190	179.6164	702.0642	-1.96713	0.001354	0.011375	1329	- && - && PF00652:Ricin-type beta-trefoil lectin domain

novel.536	17.82694	69.64424	-1.96961	0.002563	0.017942	1339	- && Q0CJ60.1 RecName: Full=Cyclase atC; AltName: Full=Terreic acid biosynthesis cluster protein C; Flags: Precursor && PF00732:GMC oxidoreductase|PF05199:GMC oxidoreductase
Lachesis_group0__13_contigs__l.g10091	319.0115	1251.735	-1.97261	0.00034	0.004224	2415	- && Q59KI0.1 RecName: Full=UTP--glucose-1-phosphate uridylyltransferase; AltName: Full=UDP-glucose pyrophosphorylase; Short=UDPGP; Short=UGPase && PF00106:short chain dehydrogenase|PF01704:UTP--glucose-1-phosphate uridylyltransferase
Lachesis_group0__13_contigs__l.g3103	187.4344	736.193	-1.97385	0.000167	0.002564	3453	- && Q9Y6Z9.1 RecName: Full=Sorbose reductase sou1; AltName: Full=Sorbitol utilization protein sou1 && PF00106:short chain dehydrogenase
Lachesis_group0__13_contigs__l.g10156	15.1062	59.32802	-1.97448	0.009043	0.044051	960	- && A7J2C6.1 RecName: Full=Acetylesterase; AltName: Full=Carbohydrate esterase family 16 protein; Flags: Precursor && PF00657:GDSL-like Lipase/Acylhydrolase
Lachesis_group0__13_contigs__l.g11360	14558.79	57275.47	-1.97603	1.00E-06	6.53E-05	1017	- && A1CFL1.1 RecName: Full=Alcohol dehydrogenase patD; AltName: Full=Patulin synthesis protein D && PF08240:Alcohol dehydrogenase GroES-like domain|PF00107:Zinc-binding dehydrogenase
Lachesis_group0__13_contigs__l.g11459	61.32586	240.8051	-1.9763	0.002599	0.018088	912	- && - && PF02602:Uroporphyrinogen-III synthase HemD
Lachesis_group0__13_contigs__l.g7741	266.1997	1049.119	-1.97895	0.000887	0.008483	1686	- && - && PF08241:Methyltransferase domain
Lachesis_group0__13_contigs__l.g4498	4772.167	18829.75	-1.98031	0.001117	0.010032	1056	- && Q9USW3.1 RecName: Full=Probable glycosidase C21B10.07 && PF00722:Glycosyl hydrolases family 16

Lachesis_group0__13_contigs__l.g5100	662.795	2617.37	-1.9817	8.52E-05	0.001621	537	- && B0DN41.1 RecName: Full=Cyanate hydratase; Short=Cyanase; AltName: Full=Cyanate hydrolase; AltName: Full=Cyanate lyase && PF02560:Cyanate lyase C-terminal domain
Lachesis_group0__13_contigs__l.g10262	7496.762	29765.9	-1.98934	8.34E-05	0.00159	2103	- && Q8VZI2.1 RecName: Full=DUF21 domain-containing protein At4g33700; AltName: Full=CBS domain-containing protein CBSDUF6 && PF01595:Domain of unknown function DUF21
Lachesis_group0__13_contigs__l.g5534	607.2576	2415.416	-1.99215	1.20E-05	0.00039	1626	- && P07921.1 RecName: Full=Lactose permease && PF00083:Sugar (and other) transporter
Lachesis_group0__13_contigs__l.g3045	70.94332	282.9307	-1.99745	0.006408	0.034442	975	- && Q00023.1 RecName: Full=Cellulose-growth-specific protein; Flags: Precursor && PF03443:Glycosyl hydrolase family 61|PF00734:Fungal cellulose binding domain
Lachesis_group0__13_contigs__l.g4178	206.2931	824.736	-2.00013	0.000525	0.00579	1650	- && P81406.1 RecName: Full=NADP-dependent glyceraldehyde-3-phosphate dehydrogenase; AltName: Full=Glyceraldehyde-3-phosphate dehydrogenase [NADP(+)]; AltName: Full=Non-phosphorylating glyceraldehyde 3-phosphate dehydrogenase; AltName: Full=Triosephosphate dehydrogenase && PF00171:Aldehyde dehydrogenase family
Lachesis_group0__13_contigs__l.g4273	16.43372	65.922	-2.00342	0.007887	0.040136	1374	- && - && PF03595:Voltage-dependent anion channel
Lachesis_group0__13_contigs__l.g8274	27.95664	112.6847	-2.01379	0.001675	0.013173	1395	- && Q9YI66.1 RecName: Full=Tyrosine-protein kinase receptor TYRO3; AltName: Full=Tyrosine-protein kinase DTK; Flags: Precursor && PF07714:Protein tyrosine kinase

Lachesis_group0__13_contigs__l.g8279	24.84682	100.4192	-2.01821	0.003158	0.020771	1026	- && Q6PAD2.1 RecName: Full=Serine/threonine-protein kinase PLK4; AltName: Full=Polo-like kinase 4; Short=PLK-4; AltName: Full=Serine/threonine-protein kinase Sak && PF07714:Protein tyrosine kinase
Lachesis_group0__13_contigs__l.g838	598.883	2431.7	-2.02174	0.000316	0.004001	1323	- && Q01284.1 RecName: Full=Nitronate monooxygenase; AltName: Full=2-nitropropane dioxygenase; Short=2-NPD; AltName: Full=Nitroalkane oxidase; Flags: Precursor && PF03060:Nitronate monooxygenase
Lachesis_group0__13_contigs__l.g451	382.6615	1564.883	-2.03209	2.59E-05	0.000673	1038	- && Q9UT59.1 RecName: Full=Putative uncharacterized oxidoreductase C513.07 && PF01370:NAD dependent epimerase/dehydratase family
Lachesis_group0__13_contigs__l.g8991	106.3194	436.6139	-2.03989	0.000206	0.003	1092	- && Q6GBM4.1 RecName: Full=Alcohol dehydrogenase; Short=ADH >Q6GJ63.1 RecName: Full=Alcohol dehydrogenase; Short=ADH >Q8NXU1.1 RecName: Full=Alcohol dehydrogenase; Short=ADH >Q5HI63.1 RecName: Full=Alcohol dehydrogenase; Short=ADH >Q2G0G1.1 RecName: Full=Alcohol dehydrogenase; Short=ADH >Q2FJ31.1 RecName: Full=Alcohol dehydrogenase; Short=ADH >Q2YSX0.1 RecName: Full=Alcohol dehydrogenase; Short=ADH && PF00107:Zinc-binding dehydrogenase

Lachesis_group0__13_contigs__l.g2540	52.08229	214.3842	-2.04404	0.000464	0.005305	1722	- && P59668.1 RecName: Full=Delta(12) fatty acid desaturase; AltName: Full=Delta-12 fatty acid desaturase && PF00487:Fatty acid desaturase
Lachesis_group0__13_contigs__l.g1952	47.53739	197.0588	-2.05004	0.000522	0.005763	444	- && - && PF16850:Peptidase inhibitor I66
Lachesis_group0__13_contigs__l.g7494	41.32524	171.0409	-2.05114	0.007348	0.038127	1917	- && A7MFA8.1 RecName: Full=Oxygen-dependent choline dehydrogenase; Short=CDH; Short=CHD; AltName: Full=Betaine aldehyde dehydrogenase; Short=BADH && PF00732:GMC oxidoreductase|PF05199:GMC oxidoreductase
Lachesis_group0__13_contigs__l.g6866	47.50446	197.6544	-2.05767	0.000134	0.00222	1893	- && - && PF13578:Methyltransferase domain|PF01501:Glycosyl transferase family 8
Lachesis_group0__13_contigs__l.g3651	205.8933	856.716	-2.058	0.000208	0.003011	1317	- && P75791.1 RecName: Full=Uncharacterized protein YbiU && PF07350:Protein of unknown function (DUF1479)
Lachesis_group0__13_contigs__l.g7087	388.7436	1619.373	-2.05882	0.001072	0.009748	2643	- && B7GFR1.1 RecName: Full=Aspartate--tRNA(Asp/Asn) ligase; AltName: Full=Aspartyl-tRNA synthetase; Short=AspRS; AltName: Full=Non-discriminating aspartyl-tRNA synthetase; Short=ND-AspRS && PF00152:tRNA synthetases class II (D, K and N)
Lachesis_group0__13_contigs__l.g11461	204.74	855.3072	-2.06377	0.000115	0.001998	2325	- && Q9C168.2 RecName: Full=Catalase-1 && PF01965:DJ-1/PfpI family|PF06628:Catalase-related immune-responsive|PF00199:Catalase
Lachesis_group0__13_contigs__l.g9060	307.1044	1284.556	-2.06497	1.43E-05	0.000443	1677	- && P55809.1 RecName: Full=Succinyl-CoA:3-ketoacid coenzyme A transferase 1, mitochondrial; AltName: Full=3-oxoacid CoA-transferase 1; AltName: Full=Somatic-type succinyl-CoA:3-

							oxoacid CoA-transferase; Short=SCOT-s; Flags: Precursor && PF01144:Coenzyme A transferase
Lachesis_group0__13_contigs__l.g1062	2574.037	10780.5	-2.06636	9.22E-06	0.000324	4593	- && P55201.2 RecName: Full=Peregrin; AltName: Full=Bromodomain and PHD finger-containing protein 1; AltName: Full=Protein Br140 && PF10513:Enhancer of polycomb-like|PF13831:PHD-finger|PF00439:Bromodomain|PF13832:PHD-zinc-finger like domain|PF00855:PWWP domain
Lachesis_group0__13_contigs__l.g5591	76.24353	319.8883	-2.0694	0.000101	0.001813	1479	- && Q0QWS4.1 RecName: Full=L-galactonate dehydratase && PF13378:Enolase C-terminal domain-like|PF02746:Mandelate racemase / muconate lactonizing enzyme, N-terminal domain
Lachesis_group0__13_contigs__l.g3172	19.47783	81.58993	-2.07138	0.008991	0.043915	2478	- && - && PF02816:Alpha-kinase family
Lachesis_group0__13_contigs__l.g9495	2409.824	10222.19	-2.08476	4.75E-06	0.000204	738	- && - && PF00646:F-box domain
Lachesis_group0__13_contigs__l.g6790	12617.75	53542.2	-2.08523	0.003819	0.023794	840	- && - && PF11563:Protoglobin
Lachesis_group0__13_contigs__l.g5392	7629.078	32447.98	-2.08856	1.69E-06	9.49E-05	1080	- && P07754.1 RecName: Full=Alcohol dehydrogenase 3; AltName: Full=Alcohol dehydrogenase III; Short=ADH III && PF08240:Alcohol dehydrogenase GroES-like domain|PF00107:Zinc-binding dehydrogenase
Lachesis_group0__13_contigs__l.g10346	1921.167	8180.664	-2.0903	2.61E-05	0.000674	1302	- && A8N4R7.1 RecName: Full=1,2-dihydroxy-3-keto-5-methylthiopentene dioxygenase 1; AltName: Full=Acireductone dioxygenase (Fe(2+)-requiring) 1; Short=ARD 1; Short=Fe-ARD 1 && PF03079:ARD/ARD' family
Lachesis_group0__13_contigs__l.g9091	552.7375	2363.67	-2.09645	3.30E-05	0.000789	1698	- && P53388.1 RecName: Full=Dicarboxylic amino acid permease && PF00324:Amino acid permease

Lachesis_group0__13_contigs__l.g4906	14659.49	62717.78	-2.09705	2.21E-05	0.000601	2271	- && Q9P326.1 RecName: Full=Transcriptional regulatory protein pro1; AltName: Full=Arrested development protein 1 && PF11951:Fungal specific transcription factor domain
Lachesis_group0__13_contigs__l.g836	114.9621	491.7835	-2.09866	0.001128	0.010065	717	- && - && PF03060:Nitronate monooxygenase
Lachesis_group0__13_contigs__l.g10551	49.30142	211.4597	-2.1026	0.002162	0.015937	1887	- && Q9FVQ0.1 RecName: Full=Probable indole-3-pyruvate monooxygenase YUCCA10; AltName: Full=Flavin-containing monooxygenase YUCCA10 && PF13738:Pyridine nucleotide-disulphide oxidoreductase
Lachesis_group0__13_contigs__l.g6589	37.88435	162.5522	-2.10579	0.00059	0.006271	1104	- && O13629.1 RecName: Full=Putative lipoate-protein ligase A && -
Lachesis_group0__13_contigs__l.g5019	202.4207	871.8454	-2.10771	2.62E-05	0.000675	1689	- && Q47944.1 RecName: Full=L-sorbose 1-dehydrogenase; Short=SDH && PF00732:GMC oxidoreductase|PF05199:GMC oxidoreductase
Lachesis_group0__13_contigs__l.g6641	980.9361	4263.88	-2.1201	8.32E-05	0.001588	2220	- && Q95M17.1 RecName: Full=Acidic mammalian chitinase; Short=AMCase; AltName: Full=Chitin-binding protein b04; Short=CBPb04; Flags: Precursor && PF00704:Glycosyl hydrolases family 18
Lachesis_group0__13_contigs__l.g3393	762.3102	3315.943	-2.12078	9.45E-05	0.001742	2451	- && Q0CEF3.1 RecName: Full=Probable beta-glucosidase L; AltName: Full=Beta-D-glucoside glucohydrolase L; AltName: Full=Cellobiase L; AltName: Full=Gentiobiase L; Flags: Precursor && PF01915:Glycosyl hydrolase family 3 C-terminal domain|PF14310:Fibronectin type III-like domain|PF00933:Glycosyl hydrolase family 3 N terminal domain

Lachesis_group0__13_contigs__l.g7707	4301.528	18739.47	-2.12317	0.00449	0.026711	2277	- && P04821.1 RecName: Full=Cell division control protein 25 && PF00617:RasGEF domain|PF00618:RasGEF N-terminal motif|PF00018:SH3 domain
Lachesis_group0__13_contigs__l.g6318	159.6216	695.8524	-2.12422	4.86E-05	0.001045	645	- && B0DK57.1 RecName: Full=Solute carrier family 25 member 38 homolog && PF00153:Mitochondrial carrier protein
Lachesis_group0__13_contigs__l.g4962	20.15691	88.12168	-2.12991	0.003527	0.022533	909	- && Q9HEZ1.1 RecName: Full=Endo-1,4-beta-xylanase A; Short=Xylanase A; AltName: Full=1,4-beta-D-xylan xylanohydrolase A; Flags: Precursor && PF00331:Glycosyl hydrolase family 10
Lachesis_group0__13_contigs__l.g4667	1193.295	5238.372	-2.13423	9.44E-05	0.001742	534	- && O14368.1 RecName: Full=Heat shock protein 16; AltName: Full=16 kDa heat shock protein && PF00011:Hsp20/alpha crystallin family
Lachesis_group0__13_contigs__l.g10337	126.7511	557.7497	-2.13733	2.71E-06	0.000133	555	- && Q9UTI7.1 RecName: Full=Probable thymidylate synthase; Short=TS; Short=TSase && PF02441:Flavoprotein
Lachesis_group0__13_contigs__l.g2888	370.1243	1630.411	-2.13925	2.17E-06	0.000112	3771	- && P0CU32.1 RecName: Full=FAD-linked oxidoreductase CHGG_01242-2; AltName: Full=Chaetoglobosin biosynthesis protein CHGG_01242-2 && PF08031:Berberine and berberine like|PF01565:FAD binding domain|PF00069:Protein kinase domain
Lachesis_group0__13_contigs__l.g3240	1505.968	6640.654	-2.14069	9.13E-05	0.001706	8106	- && Q3S2U6.1 RecName: Full=Lovastatin diketide synthase mokB; AltName: Full=Monacolin K biosynthesis protein B && PF00975:Thioesterase domain|PF02801:Beta-ketoacyl synthase, C-terminal domain|PF00698:Acyl transferase

							domain|PF14765:Polyketide synthase dehydratase|PF00550:Phosphopantetheine attachment site|PF13489:Methyltransferase domain|PF08659:KR domain|PF00109:Beta-ketoacyl synthase, N-terminal domain
Lachesis_group0__13_contigs__l.g11175	626.4884	2767.64	-2.14354	2.71E-06	0.000133	1080	- && - && PF13417:Glutathione S-transferase, N-terminal domain
Lachesis_group0__13_contigs__l.g5507	537.7125	2380.27	-2.14641	0.000226	0.003183	708	- && P37769.2 RecName: Full=2-dehydro-3-deoxy-D-gluconate 5-dehydrogenase; AltName: Full=2-deoxy-D-gluconate 3-dehydrogenase; AltName: Full=2-keto-3-deoxygluconate 5-dehydrogenase; AltName: Full=2-keto-3-deoxygluconate oxidoreductase; Short=KDG oxidoreductase; AltName: Full=20-ketosteroid reductase && PF00106:short chain dehydrogenase
Lachesis_group0__13_contigs__l.g305	7474.663	33248.01	-2.15321	1.07E-06	6.81E-05	1650	- && Q3MIF4.1 RecName: Full=Xylulose kinase; Short=Xylulokinase && PF00370:FGGY family of carbohydrate kinases, N-terminal domain|PF02782:FGGY family of carbohydrate kinases, C-terminal domain
Lachesis_group0__13_contigs__l.g1014	134.1753	596.5193	-2.15358	1.69E-05	0.000506	1542	- && Q9US44.1 RecName: Full=Uncharacterized transporter C1002.16c && PF07690:Major Facilitator Superfamily
Lachesis_group0__13_contigs__l.g4886	516.4569	2305.876	-2.15872	1.48E-06	8.53E-05	1851	- && O23461.1 RecName: Full=L-arabinokinase; Short=AtISA1 && PF13528:Glycosyl transferase family 1
Lachesis_group0__13_contigs__l.g2503	63.99089	286.0462	-2.16106	5.82E-05	0.001204	969	- && P84675.2 RecName: Full=Putative fungistatic metabolite && PF01822:WSC domain

Lachesis_group0__13_contigs__l.g2714	329.1786	1472.975	-2.16177	4.05E-05	0.000929	3603	- && Q9USG8.1 RecName: Full=Meiotically up-regulated gene 190 protein && PF00168:C2 domain
Lachesis_group0__13_contigs__l.g7994	54.81834	244.9386	-2.1626	0.000117	0.002014	708	- && P77460.2 PUTATIVE PSEUDOGENE: RecName: Full=Putative uncharacterized protein YbcY && PF08241:Methyltransferase domain
Lachesis_group0__13_contigs__l.g2907	130.2684	583.4242	-2.16332	8.81E-05	0.001662	1653	- && - && PF00011:Hsp20/alpha crystallin family
Lachesis_group0__13_contigs__l.g11178	1025.571	4594.898	-2.16377	0.000331	0.00415	936	- && O13780.1 RecName: Full=Uncharacterized protein C17G6.02c && PF04479:RTA1 like protein
Lachesis_group0__13_contigs__l.g9821	1081.798	4852.402	-2.16535	1.29E-07	1.53E-05	4662	- && - && PF00069:Protein kinase domain
Lachesis_group0__13_contigs__l.g7337	10.48371	47.48223	-2.17724	0.002522	0.01774	1815	- && - && PF00076:RNA recognition motif. (a.k.a. RRM, RBD, or RNP domain)
Lachesis_group0__13_contigs__l.g6432	98.31608	444.6304	-2.17838	1.72E-06	9.57E-05	621	- && - && PF00076:RNA recognition motif. (a.k.a. RRM, RBD, or RNP domain)
Lachesis_group0__13_contigs__l.g3645	1859.875	8445.992	-2.18318	0.000149	0.00238	423	- && - && PF14200:Ricin-type beta-trefoil lectin domain-like
Lachesis_group0__13_contigs__l.g6719	60.36739	273.5793	-2.18344	0.002139	0.015815	1449	- && A1CFY8.2 RecName: Full=Probable D-xylulose reductase A; AltName: Full=Xylitol dehydrogenase A && PF00107:Zinc-binding dehydrogenase|PF08240:Alcohol dehydrogenase GroES-like domain
Lachesis_group0__13_contigs__l.g4617	24.88362	113.4431	-2.18452	0.001607	0.012787	624	- && - && PF01822:WSC domain
Lachesis_group0__13_contigs__l.g11168	46.95781	213.2653	-2.18591	0.000163	0.002525	1026	- && Q8ZTE1.1 RecName: Full=Ketol-acid reductoisomerase (NADP(+)); Short=KARI; AltName: Full=Acetohydroxy-acid isomeroreductase; Short=AHIR; AltName: Full=Alpha-keto-beta-hydroxylacyl reductoisomerase; AltName: Full=Ketol-acid reductoisomerase type 1; AltName: Full=Ketol-acid

							reductoisomerase type I && PF07991:Acetohydroxy acid isomeroreductase, NADPH-binding domain|PF01450:Acetohydroxy acid isomeroreductase, catalytic domain
Lachesis_group0__13_contigs__l.g531	26.65806	121.5215	-2.1909	0.001298	0.011073	2076	- && - && PF12937:F-box-like
Lachesis_group0__13_contigs__l.g2264	380.683	1737.818	-2.19096	5.38E-05	0.001135	2058	- && E9R876.1 RecName: Full=MFS gliotoxin efflux transporter gliA; AltName: Full=Gliotoxin biosynthesis protein A && PF07690:Major Facilitator Superfamily
Lachesis_group0__13_contigs__l.g317	94.29011	430.5406	-2.19136	4.83E-06	0.000207	1626	- && - && PF00172:Fungal Zn(2)-Cys(6) binuclear cluster domain
Lachesis_group0__13_contigs__l.g6078	40.71414	185.9064	-2.19394	0.002544	0.017853	1692	- && G3Y416.1 RecName: Full=Cytochrome P450 monooxygenase yanC; AltName: Full=Yanuthone D synthesis protein C; Flags: Precursor && PF00067:Cytochrome P450
Lachesis_group0__13_contigs__l.g8031	614.4758	2833.244	-2.20498	6.48E-08	9.64E-06	2988	- && Q06681.1 RecName: Full=Membrane-anchored lipid-binding protein YSP2; AltName: Full=Lipid transfer at contact site protein 4; AltName: Full=Lipid transfer protein anchored at membrane contact sites 3; AltName: Full=Yeast suicide protein 2 && PF16016:Domain of unknown function (DUF4782)|PF02893:GRAM domain
Lachesis_group0__13_contigs__l.g1318	2383.072	11007.54	-2.20765	0.000104	0.001864	1995	- && A2QEQ6.1 RecName: Full=Beta-glucuronidase; Short=GlcAase; AltName: Full=Beta-D-glucuronoside glucuronosohydrolase; Flags: Precursor && PF16862:Glycosyl hydrolase family 79 C-terminal beta domain

Lachesis_group0__13_contigs__l.g1268	220.8968	1024.263	-2.21361	0.001656	0.013082	960	- && O74959.1 RecName: Full=Uncharacterized oxidoreductase C736.13 && PF00106:short chain dehydrogenase
Lachesis_group0__13_contigs__l.g8548	98.5007	456.9991	-2.21611	0.000307	0.003923	2457	- && D4ATR3.1 RecName: Full=Uncharacterized secreted glycosidase ARB_07629; Flags: Precursor && PF07971:Glycosyl hydrolase family 92
Lachesis_group0__13_contigs__l.g11056	88.73756	412.5106	-2.21804	3.62E-05	0.000843	1233	- && Q4WAZ6.1 RecName: Full=Multifunctional cytochrome P450 monooxygenase af510; AltName: Full=Fumagillin bioynthesis cluster P450 monooxygenase; Short=Fma-P450 && PF00067:Cytochrome P450
Lachesis_group0__13_contigs__l.g11362	89.08577	414.6468	-2.2191	0.00059	0.006271	2502	- && Q0CEF3.1 RecName: Full=Probable beta-glucosidase L; AltName: Full=Beta-D-glucoside glucohydrolase L; AltName: Full=Cellobiase L; AltName: Full=Gentiobiase L; Flags: Precursor && PF01915:Glycosyl hydrolase family 3 C-terminal domain|PF14310:Fibronectin type III-like domain|PF00734:Fungal cellulose binding domain|PF00933:Glycosyl hydrolase family 3 N terminal domain
Lachesis_group0__13_contigs__l.g2810	21.75702	101.515	-2.22557	0.001833	0.014195	882	- && P24665.1 RecName: Full=Aspergillopepsin-2; AltName: Full=Acid protease A; AltName: Full=Aspergillopepsin II; AltName: Full=Proctase A; Contains: RecName: Full=Aspergillopepsin-2 light chain; AltName: Full=Aspergillopepsin II light chain; Contains: RecName: Full=Aspergillopepsin-2 heavy chain; AltName: Full=Aspergillopepsin II

							heavy chain; Flags: Precursor && PF01828:Peptidase A4 family
Lachesis_group0__13_contigs__l.g1173	325.7532	1534.685	-2.23653	0.000126	0.002118	1239	- && O43050.1 RecName: Full=Sterol-4-alpha-carboxylate 3-dehydrogenase, decarboxylating && PF01073:3-beta hydroxysteroid dehydrogenase/isomerase family
Lachesis_group0__13_contigs__l.g1947	16.22388	76.16291	-2.23894	0.007856	0.040062	1215	- && A6TBU6.1 RecName: Full=2-keto-3-deoxy-L-rhamnonate aldolase; Short=KDR aldolase; AltName: Full=2-dehydro-3-deoxyrhamnonate aldolase && PF03328:HpcH/HpaI aldolase/citrate lyase family
Lachesis_group0__13_contigs__l.g3872	30.42588	143.818	-2.24338	2.40E-05	0.000636	2193	- && Q54PX0.1 RecName: Full=Probable serine/threonine-protein kinase DDB_G0284251 && PF07714:Protein tyrosine kinase
Lachesis_group0__13_contigs__l.g600	16896.89	80175.69	-2.24642	0.000771	0.007639	2517	- && O14283.2 RecName: Full=Transcription factor prr1; AltName: Full=Pombe response regulator 1 && PF00447:HSF-type DNA-binding|PF00072:Response regulator receiver domain
Lachesis_group0__13_contigs__l.g275	126.6653	602.4719	-2.24971	0.000289	0.003783	2625	- && - && PF17107:N-terminal domain on NACHT_NTPase and P-loop NTPases
Lachesis_group0__13_contigs__l.g5372	6.517702	31.08338	-2.25269	0.009137	0.044356	1296	- && B8NHY4.1 RecName: Full=O-methylsterigmatocystin oxidoreductase; Short=OMST oxidoreductase; AltName: Full=Aflatoxin B synthase; AltName: Full=Aflatoxin biosynthesis protein Q; AltName: Full=Cytochrome P450 64 >P0CT93.1 RecName: Full=O-methylsterigmatocystin oxidoreductase;

							Short=OMST oxidoreductase; AltName: Full=Aflatoxin B synthase; AltName: Full=Aflatoxin biosynthesis protein Q; AltName: Full=Cytochrome P450 64 && PF00067:Cytochrome P450
Lachesis_group0__13_contigs__l.g10514	64.37297	306.4995	-2.25301	1.17E-06	7.27E-05	1143	- && Q9HTE3.1 RecName: Full=Glutathione-independent formaldehyde dehydrogenase; Short=FALDH; Short=FDH && PF08240:Alcohol dehydrogenase GroES-like domain|PF00107:Zinc-binding dehydrogenase
Lachesis_group0__13_contigs__l.g808	72.12488	346.1207	-2.26429	0.000182	0.002703	1584	- && - && PF12937:F-box-like
Lachesis_group0__13_contigs__l.g5099	52.10272	250.4409	-2.26721	6.45E-05	0.001306	1377	- && P75791.1 RecName: Full=Uncharacterized protein YbiU && PF07350:Protein of unknown function (DUF1479)
Lachesis_group0__13_contigs__l.g4295	80.48439	387.6046	-2.26971	3.16E-05	0.000764	1776	- && B2SH83.1 RecName: Full=Dihydroxy-acid dehydratase; Short=DAD && PF00920:Dehydratase family
Lachesis_group0__13_contigs__l.g11148	110.2416	532.7614	-2.27294	6.44E-06	0.000252	882	- && Q6P8M1.1 RecName: Full=Putative deoxyribonuclease TATDN1 && PF01026:TatD related DNase
Lachesis_group0__13_contigs__l.g3789	1014.434	4910.915	-2.27533	0.000222	0.003164	3030	- && - && PF02265:S1/P1 Nuclease
Lachesis_group0__13_contigs__l.g1724	2614.048	12682.18	-2.27848	0.001683	0.013221	1755	- && - && PF02179:BAG domain
Lachesis_group0__13_contigs__l.g5375	35.42042	171.9027	-2.28236	0.005507	0.030989	1350	- && G3Y416.1 RecName: Full=Cytochrome P450 monooxygenase yanC; AltName: Full=Yanuthone D synthesis protein C; Flags: Precursor && PF00067:Cytochrome P450

Lachesis_group0__13_contigs__l.g8710	719.5909	3500.695	-2.28247	1.96E-05	0.000555	1542	- && Q800A0.1 RecName: Full=Cathepsin E; Flags: Precursor && PF00026:Eukaryotic aspartyl protease
Lachesis_group0__13_contigs__l.g10566	28.43887	138.8325	-2.28825	0.004805	0.028136	798	- && - && PF13417:Glutathione S-transferase, N-terminal domain
Lachesis_group0__13_contigs__l.g7816	14.61933	71.20794	-2.29174	0.001062	0.009691	1404	- && - && PF09810:Exonuclease V - a 5' deoxyribonuclease
Lachesis_group0__13_contigs__l.g446	96.30514	473.2794	-2.29741	0.000458	0.005272	3243	- && A0A0D2YG01.1 RecName: Full=Non-canonical non-ribosomal peptide synthetase FUB8; AltName: Full=Fusaric acid biosynthesis protein 8 && PF07993:Male sterility protein|PF00501:AMP-binding enzyme
Lachesis_group0__13_contigs__l.g1788	297.3684	1478.636	-2.31449	1.14E-05	0.000373	399	- && - && PF03330:Rare lipoprotein A (RlpA)-like double-psi beta-barrel
Lachesis_group0__13_contigs__l.g11460	301.4491	1500.852	-2.31638	2.97E-06	0.000143	651	- && - && PF00385:Chromo (CHRromatin Organisation MOdifier) domain
Lachesis_group0__13_contigs__l.g4968	16.90702	84.47214	-2.32336	0.002274	0.01651	597	- && - && PF05368:NmrA-like family
Lachesis_group0__13_contigs__l.g62	22.42076	113.2615	-2.34344	0.000908	0.008631	954	- && B7SIW2.1 RecName: Full=Endo-1,4-beta-xylanase C; Short=Xylanase C; AltName: Full=1,4-beta-D-xylan xylanohydrolase C; Flags: Precursor && PF00331:Glycosyl hydrolase family 10|PF00734:Fungal cellulose binding domain
Lachesis_group0__13_contigs__l.g4937	12.55043	63.42151	-2.3435	0.010319	0.048314	417	- && - && PF00172:Fungal Zn(2)-Cys(6) binuclear cluster domain
Lachesis_group0__13_contigs__l.g11029	398.3199	2033.957	-2.35204	0.002375	0.017076	1695	- && - && PF00501:AMP-binding enzyme
Lachesis_group0__13_contigs__l.g7745	298.8893	1528.427	-2.35462	2.09E-05	0.00058	3006	- && - && PF12937:F-box-like
Lachesis_group0__13_contigs__l.g11055	52.13098	266.6215	-2.3574	3.88E-05	0.0009	1554	- && Q4WAZ6.1 RecName: Full=Multifunctional cytochrome P450 monooxygenase af510; AltName:

							Full=Fumagillin bioynthesis cluster P450 monooxygenase; Short=Fma-P450 && PF00067:Cytochrome P450
Lachesis_group0__13_contigs__l.g7972	48.16269	246.8213	-2.35889	4.31E-06	0.00019	963	- && P18631.1 RecName: Full=Low-affinity glucose transporter; AltName: Full=Hexose transporter 1 && PF00083:Sugar (and other) transporter
Lachesis_group0__13_contigs__l.g10038	13.03966	67.05729	-2.36379	0.000287	0.003765	2580	- && - && PF00069:Protein kinase domain
Lachesis_group0__13_contigs__l.g10128	15.61008	80.82645	-2.37147	0.000679	0.006966	2388	- && Q551H4.1 RecName: Full=Serine/threonine-protein kinase fray2; AltName: Full=STE20-like kinase fray2 && PF00069:Protein kinase domain
Lachesis_group0__13_contigs__l.g8792	230.217	1193.076	-2.37381	0.000125	0.002118	1074	- && Q84V25.1 RecName: Full=2-methylene-furan-3-one reductase; AltName: Full=Enone oxidoreductase; Short=FaEO; AltName: Full=Quinone oxidoreductase; Short=FaQR && PF13602:Zinc-binding dehydrogenase
Lachesis_group0__13_contigs__l.g3062	4031.651	20956.38	-2.37797	1.22E-05	0.000393	306	- && - && PF05254:Uncharacterised protein family (UPF0203)
Lachesis_group0__13_contigs__l.g4512	207.6648	1082.447	-2.38302	1.20E-05	0.00039	1791	- && Q9SYK0.1 RecName: Full=Beta-hexosaminidase 2; AltName: Full=Beta-GlcNAcase 2; AltName: Full=Beta-N-acetylhexosaminidase 2; AltName: Full=Beta-hexosaminidase 3; Short=AtHEX3; AltName: Full=N-acetyl-beta-glucosaminidase 2; Flags: Precursor && PF00728:Glycosyl hydrolase family 20, catalytic domain|PF14845:beta-acetyl hexosaminidase like
Lachesis_group0__13_contigs__l.g540	1066.824	5611.574	-2.39514	0.00111	0.009997	1020	- && - && PF00651:BTB/POZ domain
Lachesis_group0__13_contigs__l.g7290	53.1343	281.2161	-2.4044	4.13E-05	0.000941	1614	- && P51687.2 RecName: Full=Sulfite oxidase, mitochondrial; Flags: Precursor && PF03404:Mo-co

							oxidoreductase dimerisation domain|PF00174:Oxidoreductase molybdopterin binding domain
Lachesis_group0__13_contigs__l.g4961	12.72818	67.94748	-2.42691	0.003094	0.020482	1107	- && Q9HEZ1.1 RecName: Full=Endo-1,4-beta-xylanase A; Short=Xylanase A; AltName: Full=1,4-beta-D-xylan xylanohydrolase A; Flags: Precursor && PF00331:Glycosyl hydrolase family 10
Lachesis_group0__13_contigs__l.g992	19.44786	105.4685	-2.4405	0.000203	0.002956	1593	- && B8NHY4.1 RecName: Full=O-methylsterigmatocystin oxidoreductase; Short=OMST oxidoreductase; AltName: Full=Aflatoxin B synthase; AltName: Full=Aflatoxin biosynthesis protein Q; AltName: Full=Cytochrome P450 64 >P0CT93.1 RecName: Full=O-methylsterigmatocystin oxidoreductase; Short=OMST oxidoreductase; AltName: Full=Aflatoxin B synthase; AltName: Full=Aflatoxin biosynthesis protein Q; AltName: Full=Cytochrome P450 64 && PF00067:Cytochrome P450
Lachesis_group0__13_contigs__l.g541	563.9717	3071.92	-2.44566	4.36E-06	0.000192	2256	- && O74396.3 RecName: Full=Vacuolar protein sorting-associated protein 9a; AltName: Full=Vacuolar protein-targeting protein 9a && PF02204:Vacuolar sorting protein 9 (VPS9) domain|PF02845:CUE domain
Lachesis_group0__13_contigs__l.g11063	1702.756	9397.178	-2.46446	1.95E-07	2.03E-05	2937	- && O74954.1 RecName: Full=Transcription factor cbf11; AltName: Full=C-promoter element-binding factor-like protein 11 && PF09271:LAG1, DNA

							binding丨PF09270:Beta-trefoil DNA-binding domain
Lachesis_group0__13_contigs__l.g8282	61.69746	340.6034	-2.46613	1.60E-05	0.000484	1662	- && F4JY37.1 RecName: Full=Serine/threonine-protein kinase RUNKEL; AltName: Full=Protein EMBRYO DEFECTIVE 3013; AltName: Full=Protein RUNKEL && PF07714:Protein tyrosine kinase
Lachesis_group0__13_contigs__l.g7176	489.7301	2708.683	-2.46776	1.12E-05	0.00037	432	- && - && PF01172:Shwachman-Bodian-Diamond syndrome (SBDS) protein
Lachesis_group0__13_contigs__l.g2363	20.23132	111.8072	-2.47054	0.000387	0.004668	1194	- && Q25BW5.1 RecName: Full=Beta-glucosidase 1A; AltName: Full=Cellobiase 1A && PF00232:Glycosyl hydrolase family 1
Lachesis_group0__13_contigs__l.g7661	21.78782	120.6334	-2.4759	0.000302	0.003878	1476	- && P25737.5 RecName: Full=Lysine-specific permease && PF00324:Amino acid permease
Lachesis_group0__13_contigs__l.g597	2419.639	13473.17	-2.47729	4.98E-06	0.000211	966	- && O14283.2 RecName: Full=Transcription factor prr1; AltName: Full=Pombe response regulator 1 && PF00447:HSF-type DNA-binding
Lachesis_group0__13_contigs__l.g715	13828.39	77310.43	-2.48304	0.000279	0.00369	2142	- && Q0R4L2.1 RecName: Full=Pyranose dehydrogenase 3; Short=PDH 3; AltName: Full=Pyranose:quinone oxidoreductase 3; Flags: Precursor && PF00732:GMC oxidoreductase丨PF05199:GMC oxidoreductase
Lachesis_group0__13_contigs__l.g10375	2599.885	14591.79	-2.48865	4.52E-05	0.001	510	- && - && PF02755:RPEL repeat
Lachesis_group0__13_contigs__l.g6301	170.7923	961.2931	-2.49321	1.40E-06	8.18E-05	831	- && O13871.1 RecName: Full=Uncharacterized methyltransferase C1B3.06c && PF13847:Methyltransferase domain
Lachesis_group0__13_contigs__l.g1953	20.01893	112.1206	-2.49389	0.000544	0.00594	1104	- && - && PF00149:Calcineurin-like phosphoesterase

Lachesis_group0__13_contigs__l.g1530	5567.918	31726.38	-2.5105	3.12E-05	0.000757	4059	- && P40406.1 RecName: Full=Beta-hexosaminidase; AltName: Full=Beta-N-acetylhexosaminidase; AltName: Full=N-acetyl-beta-glucosaminidase; AltName: Full=N-acetylglucosaminidase; AltName: Full=ORF1; Flags: Precursor && PF03127:GAT domain|PF00790:VHS domain|PF00933:Glycosyl hydrolase family 3 N terminal domain
Lachesis_group0__13_contigs__l.g9792	45.46637	258.9314	-2.51184	0.000127	0.002134	1719	- && Q756A9.2 RecName: Full=Acyl-coenzyme A oxidase; Short=Acyl-CoA oxidase && -
Lachesis_group0__13_contigs__l.g5712	33.27713	189.7772	-2.51407	0.000223	0.003166	2346	- && - && PF00069:Protein kinase domain
Lachesis_group0__13_contigs__l.g4634	2575.897	14785.83	-2.5211	3.54E-06	0.000162	1077	- && Q8NKE2.2 RecName: Full=Alternative oxidase, mitochondrial; Flags: Precursor && PF01786:Alternative oxidase
Lachesis_group0__13_contigs__l.g7223	3526.044	20286.21	-2.52438	0.001187	0.010357	546	- && P16934.1 RecName: Full=Fruiting body protein SC4; AltName: Full=Hydrophobin SC4; Flags: Precursor && PF01185:Fungal hydrophobin
Lachesis_group0__13_contigs__l.g5150	484.8153	2814.191	-2.5374	1.69E-05	0.000507	2967	- && P0CS23.1 RecName: Full=Urease; AltName: Full=Urea amidohydrolase >P0CS22.1 RecName: Full=Urease; AltName: Full=Urea amidohydrolase && PF00547:Urease, gamma subunit|PF01979:Amidohydrolase family|PF00449:Urease alpha-subunit, N-terminal domain|PF00699:Urease beta subunit
Lachesis_group0__13_contigs__l.g3904	273.8779	1614.594	-2.55988	9.35E-07	6.24E-05	2031	- && - && PF00561:alpha/beta hydrolase fold
Lachesis_group0__13_contigs__l.g2038	5.910284	34.58698	-2.56288	0.001931	0.014746	810	- && - && PF12697:Alpha/beta hydrolase family

Lachesis_group0__13_contigs__l.g6565	27.07326	160.7314	-2.57141	0.000158	0.002478	2814	- && Q0CI48.2 RecName: Full=Beta-mannosidase A; AltName: Full=Mannanase A; Short=Mannase A; Flags: Precursor && -
Lachesis_group0__13_contigs__l.g7749	52.44716	312.4622	-2.57543	8.21E-07	5.69E-05	1068	- && P0CU27.1 RecName: Full=Protein-lysine N-methyltransferase EFM3; AltName: Full=Elongation factor methyltransferase 3 && PF10294:Lysine methyltransferase
Lachesis_group0__13_contigs__l.g4973	2421.53	14531.77	-2.58521	2.24E-05	0.000604	741	- && - && PF12766:Pyridoxamine 5'-phosphate oxidase
Lachesis_group0__13_contigs__l.g8558	496.9343	3002.835	-2.5954	1.37E-06	8.14E-05	1398	- && Q50I20.1 RecName: Full=2-methylcitrate synthase, mitochondrial; Short=Methylcitrate synthase; AltName: Full=(2S,3S)-2-methylcitrate synthase; AltName: Full=Citrate synthase 1; Flags: Precursor >B0YD89.1 RecName: Full=2-methylcitrate synthase, mitochondrial; Short=Methylcitrate synthase; AltName: Full=(2S,3S)-2-methylcitrate synthase; AltName: Full=Citrate synthase 2; Flags: Precursor && PF00285:Citrate synthase
Lachesis_group0__13_contigs__l.g4253	2980.776	18052.61	-2.59851	7.91E-05	0.001537	1395	- && Q01358.1 RecName: Full=Protein bli-3 && PF16242:Pyridoxamine 5'-phosphate oxidase like
Lachesis_group0__13_contigs__l.g9198	10.47774	63.23759	-2.60034	6.84E-05	0.001373	432	- && D4ALV6.1 RecName: Full=Allergen Asp f 15 homolg && PF07249:Cerato-platanin
Lachesis_group0__13_contigs__l.g3509	55.9726	341.1296	-2.60861	0.000778	0.007684	1569	- && Q01679.2 RecName: Full=Laccase; AltName: Full=Benzenediol:oxygen oxidoreductase; AltName: Full=Diphenol oxidase; AltName: Full=Ligninolytic phenoloxidase; AltName: Full=Urishiol oxidase; Flags: Precursor &&

							PF00394:Multicopper oxidase|PF07732:Multicopper oxidase|PF07731:Multicopper oxidase
Lachesis_group0__13_contigs__l.g4409	22.79304	138.9694	-2.61034	0.000286	0.003755	4737	- && G3Y416.1 RecName: Full=Cytochrome P450 monooxygenase yanC; AltName: Full=Yanuthone D synthesis protein C; Flags: Precursor && PF00067:Cytochrome P450|PF07993:Male sterility protein|PF00501:AMP-binding enzyme
Lachesis_group0__13_contigs__l.g9971	416.9352	2553.677	-2.61509	9.86E-05	0.001789	1428	- && - && PF03595:Voltage-dependent anion channel
Lachesis_group0__13_contigs__l.g7430	55.20419	340.2286	-2.62432	0.00041	0.004847	1614	- && Q9C0V8.1 RecName: Full=Uncharacterized transporter PB10D8.01 && PF07690:Major Facilitator Superfamily
novel.1164	235.2885	1475.231	-2.64871	3.65E-07	3.20E-05	2041	- && - && PF10164:Uncharacterized conserved protein (DUF2367)
Lachesis_group0__13_contigs__l.g1814	27.25119	172.3694	-2.66358	3.98E-05	0.00092	2169	- && P94593.2 RecName: Full=Uncharacterized ATP-dependent helicase YwqA && PF00176:SNF2 family N-terminal domain
Lachesis_group0__13_contigs__l.g9472	37.42259	239.0129	-2.67713	9.78E-05	0.001778	1485	- && A1CFL6.2 RecName: Full=Cytochrome P450 monooxygenase patI; AltName: Full=Patulin synthesis protein I; AltName: Full=m-hydroxybenzyl alcohol hydroxylase; Flags: Precursor && PF00067:Cytochrome P450
Lachesis_group0__13_contigs__l.g2517	21.14896	135.7462	-2.68322	1.08E-05	0.000363	1395	- && O34371.1 RecName: Full=Putative oxidoreductase YteT; Flags: Precursor && PF01408:Oxidoreductase family, NAD-binding Rossmann fold|PF02894:Oxidoreductase family, C-terminal alpha/beta domain

Lachesis_group0__13_contigs__l.g6756	7041.781	45493.15	-2.69165	0.000108	0.001905	552	- && P16081.3 RecName: Full=Nitrate reductase [NADH] 1; Short=NR1 && PF00173:Cytochrome b5-like Heme/Steroid binding domain
Lachesis_group0__13_contigs__l.g4353	146.9404	950.0891	-2.69363	2.23E-06	0.000115	630	- && - && PF08534:Redoxin
Lachesis_group0__13_contigs__l.g6191	52.25646	339.0432	-2.69779	2.71E-08	5.12E-06	1002	- && Q9UTA1.2 RecName: Full=Zinc finger protein C25B8.19c && -
Lachesis_group0__13_contigs__l.g281	77.62053	506.8882	-2.70883	7.64E-05	0.001498	2409	- && - && PF17107:N-terminal domain on NACHT_NTPase and P-loop NTPases
Lachesis_group0__13_contigs__l.g6312	1437.268	9402.098	-2.70968	7.78E-08	1.09E-05	339	- && - && PF10937:Protein of unknown function (DUF2638)
Lachesis_group0__13_contigs__l.g598	623.595	4117.359	-2.72315	0.000179	0.002674	807	- && O14283.2 RecName: Full=Transcription factor prr1; AltName: Full=Pombe response regulator 1 && PF00447:HSF-type DNA-binding
Lachesis_group0__13_contigs__l.g9752	403.1238	2676.42	-2.7313	9.66E-10	2.86E-07	375	- && O95563.1 RecName: Full=Mitochondrial pyruvate carrier 2; AltName: Full=Brain protein 44 && PF03650:Uncharacterised protein family (UPF0041)
Lachesis_group0__13_contigs__l.g8242	228.7681	1523.428	-2.73565	3.25E-10	1.27E-07	1185	- && - && PF00652:Ricin-type beta-trefoil lectin domain
Lachesis_group0__13_contigs__l.g10522	292.8221	1965.715	-2.74743	0.00011	0.001934	1503	- && O74187.1 RecName: Full=Aldehyde dehydrogenase; Short=ALDDH; Short=ALDH && PF00171:Aldehyde dehydrogenase family
Lachesis_group0__13_contigs__l.g2276	59.37432	398.8576	-2.75133	9.54E-07	6.33E-05	1551	- && Q25BW4.1 RecName: Full=Beta-glucosidase 1B; AltName: Full=Cellobiase 1B && PF00232:Glycosyl hydrolase family 1
Lachesis_group0__13_contigs__l.g8727	100.2195	678.1872	-2.76	1.86E-08	3.99E-06	2337	- && Q9URY8.1 RecName: Full=Probable sulfate permease C869.05c && PF01740:STAS domain|PF00916:Sulfate permease family

Lachesis_group0__13_contigs__l.g4541	17.30946	117.1432	-2.76077	6.08E-05	0.001246	1155	- && Q8RXK7.2 RecName: Full=Probable pectinesterase/pectinesterase inhibitor 41; Includes: RecName: Full=Pectinesterase inhibitor 41; AltName: Full=Pectin methylesterase inhibitor 41; Includes: RecName: Full=Pectinesterase 41; Short=PE 41; AltName: Full=AtPMEpcrB; AltName: Full=Pectin methylesterase 41; Short=AtPME41; Flags: Precursor && PF01095:Pectinesterase
Lachesis_group0__13_contigs__l.g3973	23.26589	157.7457	-2.76095	1.75E-06	9.60E-05	723	- && O94255.2 RecName: Full=Carbonic anhydrase; AltName: Full=Carbonate dehydratase && PF00484:Carbonic anhydrase
Lachesis_group0__13_contigs__l.g4960	6.67418	44.95734	-2.76108	0.00052	0.00575	1203	- && Q9HEZ1.1 RecName: Full=Endo-1,4-beta-xylanase A; Short=Xylanase A; AltName: Full=1,4-beta-D-xylan xylanohydrolase A; Flags: Precursor && PF00331:Glycosyl hydrolase family 10|PF00734:Fungal cellulose binding domain
Lachesis_group0__13_contigs__l.g9763	3347.452	22944.08	-2.77699	9.03E-08	1.24E-05	2370	- && O95563.1 RecName: Full=Mitochondrial pyruvate carrier 2; AltName: Full=Brain protein 44 && PF03650:Uncharacterised protein family (UPF0041)
Lachesis_group0__13_contigs__l.g9768	55.0673	376.7959	-2.77701	1.73E-06	9.57E-05	2799	- && Q2U4L7.2 RecName: Full=Glutaminase A; Flags: Precursor && PF16335:Domain of unknown function (DUF4965)|PF08760:Domain of unknown function (DUF1793)
Lachesis_group0__13_contigs__l.g5245	874.8145	5996.985	-2.77718	5.81E-09	1.41E-06	2412	- && G0SGU4.1 RecName: Full=Formate dehydrogenase; Short=FDH; AltName: Full=NAD-dependent formate dehydrogenase && PF00389:D-isomer specific 2-hydroxyacid dehydrogenase,

							catalytic domain¦PF02826:D-isomer specific 2-hydroxyacid dehydrogenase, NAD binding domain
Lachesis_group0__13_contigs__l.g6438	43.96429	302.5472	-2.78534	6.97E-05	0.001393	1077	- && Q76NT9.1 RecName: Full=1-aminocyclopropane-1-carboxylate oxidase; Short=ACC oxidase; Short=Ddaco; AltName: Full=Ethylene-forming enzyme; Short=EFE >A6BM06.1 RecName: Full=1-aminocyclopropane-1-carboxylate oxidase; Short=ACC oxidase; Short=Dmaco; AltName: Full=Ethylene-forming enzyme; Short=EFE && PF03171:2OG-Fe(II) oxygenase superfamily¦PF14226:non-haem dioxygenase in morphine synthesis N-terminal
Lachesis_group0__13_contigs__l.g3548	57.85675	398.3266	-2.78618	1.87E-05	0.000543	3453	- && A0A0D2YG01.1 RecName: Full=Non-canonical non-ribosomal peptide synthetase FUB8; AltName: Full=Fusaric acid biosynthesis protein 8 && PF00501:AMP-binding enzyme¦PF07993:Male sterility protein
Lachesis_group0__13_contigs__l.g7950	23.55884	163.5909	-2.79672	3.37E-05	0.000801	1371	- && A9JPE2.1 RecName: Full=Cytochrome P450 monooxygenase atmQ; AltName: Full=Aflatrem synthesis protein Q && PF00067:Cytochrome P450
Lachesis_group0__13_contigs__l.g4108	24.23135	168.9261	-2.80466	2.58E-05	0.000673	657	- && - && PF00583:Acetyltransferase (GNAT) family
Lachesis_group0__13_contigs__l.g9972	1606.2	11224.42	-2.80495	1.91E-05	0.000549	1602	- && - && PF03595:Voltage-dependent anion channel
Lachesis_group0__13_contigs__l.g9767	62.89084	439.6502	-2.80609	6.96E-06	0.000267	2613	- && D4AMT2.2 RecName: Full=Probable glutaminase ARB_05535/05536; Flags: Precursor

							&& PF08760:Domain of unknown function (DUF1793)|PF16335:Domain of unknown function (DUF4965)
Lachesis_group0__13_contigs__l.g8030	446.8137	3166.306	-2.82527	6.38E-05	0.001296	330	- && P29429.2 RecName: Full=Thioredoxin; Short=Trx && PF00085:Thioredoxin
Lachesis_group0__13_contigs__l.g10523	599.3889	4250.144	-2.82623	6.92E-07	5.19E-05	1503	- && O74187.1 RecName: Full=Aldehyde dehydrogenase; Short=ALDDH; Short=ALDH && PF00171:Aldehyde dehydrogenase family
Lachesis_group0__13_contigs__l.g10102	6.549378	46.83855	-2.84136	0.000438	0.005095	1599	- && Q9M156.1 RecName: Full=UDP-glycosyltransferase 72B1; AltName: Full=Arbutin synthase; AltName: Full=Probable hydroquinone glucosyltransferase && PF00201:UDP-glucoronosyl and UDP-glucosyl transferase
Lachesis_group0__13_contigs__l.g9674	38.48242	275.7183	-2.84345	2.81E-05	0.000707	3369	- && Q4WRH9.1 RecName: Full=Probable alpha/beta-glucosidase agdC; Flags: Precursor >B0XNL6.1 RecName: Full=Probable alpha/beta-glucosidase agdC; Flags: Precursor && PF01055:Glycosyl hydrolases family 31|PF13802:Galactose mutarotase-like|PF16863:N-terminal barrel of NtMGAM and CtMGAM, maltase-glucoamylase
Lachesis_group0__13_contigs__l.g282	8.628678	61.98294	-2.85304	0.000338	0.004201	1587	- && O60020.1 RecName: Full=Aspartic protease; Flags: Precursor && PF00026:Eukaryotic aspartyl protease
Lachesis_group0__13_contigs__l.g9562	807.0415	5858.317	-2.86002	1.80E-08	3.99E-06	423	- && D4ALV6.1 RecName: Full=Allergen Asp f 15 homolg && PF07249:Cerato-platanin
Lachesis_group0__13_contigs__l.g10899	375.3244	2750.875	-2.87356	1.84E-08	3.99E-06	1500	- && Q54HD2.1 RecName: Full=Probable serine/threonine-protein kinase ndrD; AltName:

							Full=Nuclear DBF2-related kinase D && PF00069:Protein kinase domain
Lachesis_group0__13_contigs__l.g8704	117.7508	862.3373	-2.87403	5.73E-06	0.000232	1488	- && - && PF04616:Glycosyl hydrolases family 43
Lachesis_group0__13_contigs__l.g8376	390.1342	2863.372	-2.87603	9.56E-05	0.001758	339	- && - && PF01185:Fungal hydrophobin
Lachesis_group0__13_contigs__l.g10552	20.86255	153.6198	-2.88562	3.32E-05	0.000792	1032	- && Q9UVP6.1 RecName: Full=Versatile peroxidase VPS1; AltName: Full=Versatile solid phase peroxidase 1; Flags: Precursor && PF00141:Peroxidase|PF11895:Domain of unknown function (DUF3415)
Lachesis_group0__13_contigs__l.g6193	27.85097	206.4064	-2.89722	1.75E-05	0.000516	1662	- && A2QEQ6.1 RecName: Full=Beta-glucuronidase; Short=GlcAase; AltName: Full=Beta-D-glucuronoside glucuronosohydrolase; Flags: Precursor && PF16862:Glycosyl hydrolase family 79 C-terminal beta domain
Lachesis_group0__13_contigs__l.g10515	40.96971	307.756	-2.91168	4.02E-05	0.000926	1662	- && - && PF00501:AMP-binding enzyme
Lachesis_group0__13_contigs__l.g7178	139.0624	1046.726	-2.91252	2.15E-06	0.000112	2577	- && A1CA51.1 RecName: Full=Probable beta-glucosidase I; AltName: Full=Beta-D-glucoside glucohydrolase I; AltName: Full=Cellobiase I; AltName: Full=Gentiobiase I && PF00933:Glycosyl hydrolase family 3 N terminal domain|PF01915:Glycosyl hydrolase family 3 C-terminal domain|PF14310:Fibronectin type III-like domain
Lachesis_group0__13_contigs__l.g6878	357.1863	2714.202	-2.92605	4.78E-07	4.00E-05	1164	- && O74750.1 RecName: Full=UDP-N-acetylglucosamine transporter yea4 && PF08449:UAA transporter family

Lachesis_group0__13_contigs__l.g4414	6.461149	49.03659	-2.93928	0.00012	0.002043	1326	- && O94343.1 RecName: Full=Uncharacterized MFS-type transporter C1271.10c && PF07690:Major Facilitator Superfamily
Lachesis_group0__13_contigs__l.g8595	2263.769	17583.32	-2.95749	4.30E-08	7.53E-06	882	- && A3PXS9.1 RecName: Full=Uncharacterized oxidoreductase Mjls_1918 && PF00248:Aldo/keto reductase family
Lachesis_group0__13_contigs__l.g1711	33.74045	265.5457	-2.97527	4.96E-05	0.00106	1452	- && B7ID58.1 RecName: Full=8-amino-7-oxononanoate synthase; Short=AONS; AltName: Full=7-keto-8-amino-pelargonic acid synthase; Short=7-KAP synthase; Short=KAPA synthase; AltName: Full=8-amino-7-ketopelargonate synthase; AltName: Full=Alpha-oxoamine synthase && PF00155:Aminotransferase class I and II
Lachesis_group0__13_contigs__l.g3871	26.13948	208.1346	-2.98986	5.85E-07	4.61E-05	2310	- && C5PFX0.2 RecName: Full=Nuclear distribution protein PAC1; AltName: Full=Lissencephaly-1 homolog; Short=LIS-1; AltName: Full=nudF homolog && PF00400:WD domain, G-beta repeat
Lachesis_group0__13_contigs__l.g10697	15.41554	121.7612	-2.99068	4.49E-05	0.001	330	- && - && PF12770:CHAT domain
Lachesis_group0__13_contigs__l.g10011	45.08053	363.8037	-3.01242	4.05E-05	0.000929	2391	- && D4B0V1.1 RecName: Full=Probable glucan endo-1,3-beta-glucosidase ARB_02077; AltName: Full=(1->3)-beta-glucan endohydrolase ARB_02077; Short=(1->3)-beta-glucanase ARB_02077; Flags: Precursor && PF12708:Pectate lyase superfamily protein
Lachesis_group0__13_contigs__l.g4685	832.5773	6812.08	-3.03246	9.40E-08	1.26E-05	549	- && P0AE54.1 RecName: Full=Putative peroxiredoxin bcp; AltName: Full=Bacterioferritin comigratory protein; AltName: Full=Thioredoxin

							reductase >P0AE53.1 RecName: Full=Putative peroxiredoxin bcp; AltName: Full=Bacterioferritin comigratory protein; AltName: Full=Thioredoxin reductase >P0AE52.1 RecName: Full=Putative peroxiredoxin bcp; AltName: Full=Bacterioferritin comigratory protein; AltName: Full=Thioredoxin reductase >P0AE55.1 RecName: Full=Putative peroxiredoxin bcp; AltName: Full=Bacterioferritin comigratory protein; AltName: Full=Thioredoxin reductase && PF00578:AhpC/TSA family
Lachesis_group0__13_contigs__l.g10661	9.081972	74.91966	-3.03795	2.35E-06	0.000119	6573	- && Q8BV79.3 RecName: Full=TPR and ankyrin repeat-containing protein 1; AltName: Full=Lupus brain antigen 1 && PF13361:UvrD-like helicase C-terminal domain|PF00580:UvrD/REP helicase N-terminal domain
Lachesis_group0__13_contigs__l.g5121	883.8805	7395.751	-3.0649	1.49E-11	1.26E-08	813	- && Q9ZNN8.1 RecName: Full=L-2,3-butanediol dehydrogenase; Short=L-BDH; AltName: Full=(S,S)-butanediol dehydrogenase; AltName: Full=Diacetyl reductase [(S)-acetoin forming] && PF00106:short chain dehydrogenase
Lachesis_group0__13_contigs__l.g3254	84.49643	711.2741	-3.07419	1.82E-06	9.87E-05	3033	- && - && PF00004:ATPase family associated with various cellular activities (AAA)
Lachesis_group0__13_contigs__l.g60	60.18585	507.9476	-3.07766	2.36E-06	0.000119	1077	- && Q4WBW4.1 RecName: Full=Probable acetylxylan esterase A; Flags: Precursor && PF10503:Esterase PHB depolymerase
Lachesis_group0__13_contigs__l.g797	255.7724	2237.054	-3.12905	2.93E-07	2.65E-05	2097	- && - && PF00004:ATPase family associated with various cellular activities (AAA)

Lachesis_group0__13_contigs__l.g5403	28136.55	247393.8	-3.1363	7.38E-06	0.000281	1074	- && P43067.1 RecName: Full=Alcohol dehydrogenase 1; AltName: Full=40 kDa allergen; AltName: Full=Allergen Can a 1; AltName: Full=Allergen Can a I; AltName: Allergen=Cand a 1 && PF08240:Alcohol dehydrogenase GroES-like domain|PF00107:Zinc-binding dehydrogenase
Lachesis_group0__13_contigs__l.g2012	19.39473	171.9361	-3.14861	6.66E-07	5.10E-05	2025	- && Q0CTV2.1 RecName: Full=Probable alpha-L-arabinofuranosidase A; Short=ABF A; Short=Arabinosidase A; Flags: Precursor && PF06964:Alpha-L-arabinofuranosidase C-terminus
Lachesis_group0__13_contigs__l.g8862	42.79032	383.5093	-3.16353	4.48E-06	0.000195	1383	- && Q8R0V5.3 RecName: Full=Indoleamine 2,3-dioxygenase 2; Short=IDO-2; AltName: Full=Indoleamine 2,3-dioxygenase-like protein 1; AltName: Full=Indoleamine-pyrrole 2,3-dioxygenase-like protein 1 && PF01231:Indoleamine 2,3-dioxygenase
Lachesis_group0__13_contigs__l.g150	25.75253	230.8453	-3.16473	2.05E-06	0.000108	1164	- && Q04835.1 RecName: Full=Uncharacterized membrane protein YMR253C && PF00892:EamA-like transporter family
Lachesis_group0__13_contigs__l.g5942	158.6916	1425.759	-3.16838	1.05E-11	1.15E-08	1047	- && P43549.1 RecName: Full=Uncharacterized membrane protein YFL054C && PF00230:Major intrinsic protein
Lachesis_group0__13_contigs__l.g3054	6304.308	57234.26	-3.18248	8.37E-06	0.000302	3528	- && P05023.1 RecName: Full=Sodium/potassium-transporting ATPase subunit alpha-1; Short=Na(+)/K(+) ATPase alpha-1 subunit; AltName: Full=Sodium pump subunit alpha-1; Flags: Precursor && PF13246:Cation transport ATPase (P-type)|PF00702:haloacid dehalogenase-

							like hydrolase|PF00122:E1-E2 ATPase|PF00690:Cation transporter/ATPase, N-terminus|PF00689:Cation transporting ATPase, C-terminus
Lachesis_group0__13_contigs__l.g5530	130.7982	1209.49	-3.20988	8.48E-06	0.000304	1578	- && P07921.1 RecName: Full=Lactose permease && PF00083:Sugar (and other) transporter
Lachesis_group0__13_contigs__l.g1739	172.6872	1617.421	-3.22859	1.94E-07	2.03E-05	1803	- && Q00922.1 RecName: Full=Alcohol oxidase; Short=AO; Short=AOX; AltName: Full=Methanol oxidase; Short=MOX && PF05199:GMC oxidoreductase|PF00732:GMC oxidoreductase
Lachesis_group0__13_contigs__l.g552	34.76921	327.3336	-3.23993	1.83E-07	1.97E-05	1968	- && Q5G234.1 RecName: Full=Pyranose 2-oxidase; Short=P2Ox; Short=POD; Short=POx; Short=PROD; Short=Pyranose oxidase; AltName: Full=FAD-oxidoreductase; AltName: Full=Glucose 2-oxidase; AltName: Full=Pyranose:oxygen 2-oxidoreductase; Flags: Precursor && PF05199:GMC oxidoreductase
Lachesis_group0__13_contigs__l.g3661	5.132983	50.00194	-3.28247	0.000202	0.002949	792	- && - && PF11807:Domain of unknown function (DUF3328)
Lachesis_group0__13_contigs__l.g2185	25.29586	252.7126	-3.31928	4.30E-09	1.10E-06	1935	- && - && PF00856:SET domain
Lachesis_group0__13_contigs__l.g9559	1181.206	11865.42	-3.32844	2.71E-06	0.000133	8046	- && P04323.1 RecName: Full=Retrovirus-related Pol polyprotein from transposon 17.6; Includes: RecName: Full=Protease; Includes: RecName: Full=Reverse transcriptase; Includes: RecName: Full=Endonuclease && PF00665:Integrase core domain|PF00078:Reverse transcriptase (RNA-dependent DNA polymerase)
Lachesis_group0__13_contigs__l.g5078	5166.296	51955.64	-3.33011	1.73E-05	0.000515	2307	- && O74770.1 RecName: Full=Probable phosphoketolase && PF09363:XFP C-terminal

							domain|PF09364:XFP N-terminal domain|PF03894:D-xylulose 5-phosphate/D-fructose 6-phosphate phosphoketolase
Lachesis_group0__13_contigs__l.g5077	604.3613	6097.503	-3.33504	2.29E-06	0.000117	1395	- && Q7SH17.3 RecName: Full=Probable acetate kinase; AltName: Full=Acetokinase >Q5B3G6.2 RecName: Full=Probable acetate kinase; AltName: Full=Acetokinase && PF00871:Acetokinase family
Lachesis_group0__13_contigs__l.g6766	256.8489	2600.56	-3.34015	4.75E-07	4.00E-05	2499	- && I2C092.1 RecName: Full=Beta-mannosidase B; AltName: Full=Mannanase B; Short=Mannase B && -
Lachesis_group0__13_contigs__l.g8160	22.49133	230.1008	-3.35651	9.73E-06	0.000335	1380	- && P23262.4 RecName: Full=Salicylate hydroxylase; AltName: Full=Salicylate 1-monooxygenase && PF13450:NAD(P)-binding Rossmann-like domain|PF01494:FAD binding domain
Lachesis_group0__13_contigs__l.g8241	4.071437	42.70868	-3.39569	0.000401	0.00479	885	- && - && PF00652:Ricin-type beta-trefoil lectin domain
Lachesis_group0__13_contigs__l.g5319	310.8334	3313.944	-3.41496	1.00E-05	0.000342	2592	- && Q00922.1 RecName: Full=Alcohol oxidase; Short=AO; Short=AOX; AltName: Full=Methanol oxidase; Short=MOX && PF05199:GMC oxidoreductase|PF00732:GMC oxidoreductase
Lachesis_group0__13_contigs__l.g4683	47.52527	515.5346	-3.44343	5.68E-08	8.89E-06	2316	- && Q9Y6Z9.1 RecName: Full=Sorbose reductase sou1; AltName: Full=Sorbitol utilization protein sou1 && PF13561:Enoyl-(Acyl carrier protein) reductase|PF00106:short chain dehydrogenase
Lachesis_group0__13_contigs__l.g7748	978.4852	10758.4	-3.4588	8.44E-10	2.57E-07	849	- && - && PF08325:WLM domain
Lachesis_group0__13_contigs__l.g9406	79.79915	900.7947	-3.49909	8.73E-09	2.08E-06	2289	- && Q4WFI6.1 RecName: Full=Probable exo-1,4-beta-xylosidase bxlB; AltName: Full=1,4-beta-D-

							xylan xylohydrolase bxlB; AltName: Full=Beta-xylosidase bxlB; AltName: Full=Xylobiase bxlB; Flags: Precursor >B0Y0I4.1 RecName: Full=Probable exo-1,4-beta-xylosidase bxlB; AltName: Full=1,4-beta-D-xylan xylohydrolase bxlB; AltName: Full=Beta-xylosidase bxlB; AltName: Full=Xylobiase bxlB; Flags: Precursor && PF01915:Glycosyl hydrolase family 3 C-terminal domain|PF14310:Fibronectin type III-like domain|PF00933:Glycosyl hydrolase family 3 N terminal domain
Lachesis_group0__13_contigs__l.g10950	2595.911	30343.71	-3.54716	7.26E-11	3.62E-08	981	- && Q876L8.1 RecName: Full=NAD(P)H-dependent D-xylose reductase xyl1; Short=XR && PF00248:Aldo/keto reductase family
Lachesis_group0__13_contigs__l.g8243	226.0125	2642.785	-3.54806	1.05E-06	6.75E-05	882	- && - && PF00652:Ricin-type beta-trefoil lectin domain
Lachesis_group0__13_contigs__l.g11663	1095.751	12866.86	-3.55377	1.17E-07	1.45E-05	1266	- && P20368.2 RecName: Full=Alcohol dehydrogenase 1; AltName: Full=Alcohol dehydrogenase I; Short=ADH I && PF08240:Alcohol dehydrogenase GroES-like domain|PF00107:Zinc-binding dehydrogenase
Lachesis_group0__13_contigs__l.g10079	1.074307	13.04019	-3.59567	0.009871	0.046838	1515	- && P87035.1 RecName: Full=Guanine nucleotide-binding protein alpha-4 subunit && PF00503:G-protein alpha subunit
Lachesis_group0__13_contigs__l.g8740	1856.669	22754.05	-3.61529	1.28E-11	1.18E-08	1641	- && - && PF01936:NYN domain
Lachesis_group0__13_contigs__l.g7267	60.34654	766.8509	-3.66899	1.12E-06	7.11E-05	1887	- && P36619.2 RecName: Full=Leptomycin B resistance protein pmd1 && PF00005:ABC transporter

Lachesis_group0__13_contigs__l.g837	2763.38	35941.77	-3.7012	7.07E-07	5.27E-05	1053	- && Q01284.1 RecName: Full=Nitronate monooxygenase; AltName: Full=2-nitropropane dioxygenase; Short=2-NPD; AltName: Full=Nitroalkane oxidase; Flags: Precursor && PF03060:Nitronate monooxygenase
Lachesis_group0__13_contigs__l.g8159	67.79935	889.0704	-3.71485	1.54E-10	6.51E-08	1365	- && P23262.4 RecName: Full=Salicylate hydroxylase; AltName: Full=Salicylate 1-monooxygenase && PF01494:FAD binding domain
Lachesis_group0__13_contigs__l.g4059	20.46562	281.5701	-3.78713	2.38E-10	9.64E-08	564	- && - && PF07883:Cupin domain
Lachesis_group0__13_contigs__l.g10575	692.1531	10220.03	-3.88445	3.84E-12	5.26E-09	3297	- && Q9XF67.1 RecName: Full=3-phosphoinositide-dependent protein kinase 1; Short=AtPDK1 && PF00069:Protein kinase domain
Lachesis_group0__13_contigs__l.g4400	3.467628	55.56105	-3.996	2.01E-05	0.000561	1845	- && Q47944.1 RecName: Full=L-sorbose 1-dehydrogenase; Short=SDH && PF05199:GMC oxidoreductase|PF00732:GMC oxidoreductase
Lachesis_group0__13_contigs__l.g5817	1113.282	20396.81	-4.19562	1.22E-10	5.34E-08	5838	- && P41820.1 RecName: Full=Brefeldin A resistance protein && PF06422:CDR ABC transporter|PF00005:ABC transporter|PF01061:ABC-2 type transporter|PF00656:Caspase domain|PF14510:ABC-transporter extracellular N-terminal
Lachesis_group0__13_contigs__l.g1604	68.76389	1274.903	-4.21571	4.07E-10	1.54E-07	3090	- && - && PF15979:Glycosyl hydrolase family 115
Lachesis_group0__13_contigs__l.g1951	15.65771	293.0657	-4.23178	4.06E-07	3.47E-05	459	- && - && PF16850:Peptidase inhibitor I66
Lachesis_group0__13_contigs__l.g9199	533.8933	11554.32	-4.43579	5.30E-11	3.23E-08	435	- && P0CW95.1 RecName: Full=Heat-stable 19 kDa antigen; AltName: Full=Coccidioides-specific antigen; Short=CS antigen; Short=CS-AG; AltName: Full=Precipitin antigen; AltName: Full=TP-AG;

							Flags: Precursor >E9CX44.1 RecName: Full=Heat-stable 19 kDa antigen; AltName: Full=Coccidioides-specific antigen; Short=CS antigen; Short=CS-AG; AltName: Full=Precipitin antigen; AltName: Full=TP-AG; Flags: Precursor && PF07249:Cerato-platanin
Lachesis_group0__13_contigs__l.g7910	138.7579	3242.363	-4.54679	0.000403	0.004795	1713	- && P49374.1 RecName: Full=High-affinity glucose transporter && PF00083:Sugar (and other) transporter
Lachesis_group0__13_contigs__l.g8423	19.40916	458.6174	-4.57072	7.89E-11	3.76E-08	900	- && Q9X248.1 RecName: Full=3-oxoacyl-[acyl-carrier-protein] reductase FabG; AltName: Full=3-ketoacyl-acyl carrier protein reductase; AltName: Full=Beta-Ketoacyl-acyl carrier protein reductase; AltName: Full=Beta-ketoacyl-ACP reductase && PF00106:short chain dehydrogenase
Lachesis_group0__13_contigs__l.g6349	76.0651	3017.569	-5.31084	6.02E-25	3.30E-21	1521	- && P0CP76.1 RecName: Full=Chitin deacetylase; Flags: Precursor && PF01522:Polysaccharide deacetylase

Log_2Fold change: values of transcripts upregulated in BaP addition is represented by positive numbers and downregulated is represented by negative numbers.

Padj: adjust *P*-value

Table S3 Expression of genes in the Brij30_BaP and BaP comparisons

gene_id	Brij30_BaP	BaP	log_2FoldChange	*P*value	padj	gene_length	gene_description
Lachesis_group0__13_contigs__l.g7738	173.7051	10.5059	4.054323	1.08E-09	1.13E-06	1077	- && P73138.1 RecName: Full=S-(hydroxymethyl)glutathione dehydrogenase; AltName: Full=Alcohol dehydrogenase class-3; AltName: Full=Alcohol dehydrogenase class-III; AltName: Full=Glutathione-dependent formaldehyde dehydrogenase; Short=FALDH; Short=FDH; Short=GSH-FDH && PF00107:Zinc-binding dehydrogenase丨PF08240:Alcohol dehydrogenase GroES-like domain
Lachesis_group0__13_contigs__l.g768	16020.95	1131.03	3.824302	0.000848	0.018173	1986	- && - && PF00004:ATPase family associated with various cellular activities (AAA)
Lachesis_group0__13_contigs__l.g6780	8137.322	718.9569	3.500657	0.002805	0.036752	1959	- && P30608.1 RecName: Full=Cytochrome P450 52A6; AltName: Full=Alkane-inducible P450-ALK3; AltName: Full=CYPLIIA6 && PF00067:Cytochrome P450
Lachesis_group0__13_contigs__l.g7729	2038.844	237.4297	3.102624	4.26E-08	1.74E-05	1071	- && G3XMC6.1 RecName: Full=Dehydrogenase azaJ; AltName: Full=Azaphilone biosynthesis cluster protein azaJ && PF08240:Alcohol dehydrogenase GroES-like domain丨PF00107:Zinc-binding dehydrogenase
Lachesis_group0__13_contigs__l.g2647	1880.057	235.9168	2.993938	3.80E-06	0.000457	1212	- && Q01752.1 RecName: Full=Aryl-alcohol dehydrogenase [NADP(+)]; Short=AAD && PF00248:Aldo/keto reductase family
Lachesis_group0__13_contigs__l.g9966	975.586	134.8293	2.857095	0.000105	0.004234	1728	- && Q4P460.1 RecName: Full=Sulfate adenylyltransferase; AltName: Full=ATP-sulfurylase; AltName: Full=Sulfate adenylate transferase; Short=SAT && PF01747:ATP-

							sulfurylase|PF01583:Adenylylsulphate kinase|PF14306:PUA-like domain
Lachesis_group0__13_contigs__l.g1072	44148.61	6784.69	2.702037	5.89E-05	0.002888	1713	- && P61871.1 RecName: Full=Lipase; AltName: Full=Lipase II; AltName: Full=RNL; AltName: Full=Triacylglycerol lipase; Flags: Precursor >P61872.1 RecName: Full=Lipase; AltName: Full=RDL; AltName: Full=Triacylglycerol lipase; Short=ROL; Flags: Precursor && PF01764:Lipase (class 3)
Lachesis_group0__13_contigs__l.g4059	141.9435	22.31177	2.672041	0.000517	0.012641	564	- && - && PF07883:Cupin domain
Lachesis_group0__13_contigs__l.g8358	1221.006	195.8114	2.641597	2.13E-12	2.00E-08	1050	- && Q91XV4.1 RecName: Full=L-xylulose reductase; Short=XR; AltName: Full=Dicarbonyl/L-xylulose reductase; AltName: Full=Sperm antigen P26h && PF00106:short chain dehydrogenase
Lachesis_group0__13_contigs__l.g10346	12565.57	2087.793	2.589435	1.77E-05	0.001245	1302	- && A8N4R7.1 RecName: Full=1,2-dihydroxy-3-keto-5-methylthiopentene dioxygenase 1; AltName: Full=Acireductone dioxygenase (Fe(2+)-requiring) 1; Short=ARD 1; Short=Fe-ARD 1 && PF03079:ARD/ARD' family
Lachesis_group0__13_contigs__l.g6791	5662.49	942.3104	2.587294	2.02E-05	0.001366	513	- && Q9JIM4.1 RecName: Full=Dual specificity protein phosphatase 12; AltName: Full=Glucokinase-associated dual specificity phosphatase; Short=GKAP && PF00782:Dual specificity phosphatase, catalytic domain
Lachesis_group0__13_contigs__l.g5413	14449.98	2405.909	2.586501	6.78E-07	0.000138	2343	- && Q54CQ8.1 RecName: Full=von Willebrand factor A domain-containing protein DDB_G0292740 && PF13768:von Willebrand factor type A domain|PF08487:Vault protein inter-alpha-trypsin domain

Lachesis_group0__13_contigs__l.g8098	2718.038	453.2877	2.584685	6.40E-07	0.000136	1320	- && P17576.1 RecName: Full=Polyporopepsin; AltName: Full=Aspartic proteinase && PF00026:Eukaryotic aspartyl protease
Lachesis_group0__13_contigs__l.g535	1584.268	273.2121	2.535833	5.86E-06	0.00061	1350	- && P23262.4 RecName: Full=Salicylate hydroxylase; AltName: Full=Salicylate 1-monooxygenase && PF01494:FAD binding domain
Lachesis_group0__13_contigs__l.g7176	3052.411	533.2226	2.517313	4.23E-06	0.000489	432	- && - && PF01172:Shwachman-Bodian-Diamond syndrome (SBDS) protein
Lachesis_group0__13_contigs__l.g7437	1850.367	329.7972	2.489178	1.56E-06	0.000239	948	- && Q8NBN7.2 RecName: Full=Retinol dehydrogenase 13; AltName: Full=Short chain dehydrogenase/reductase family 7C member 3 && PF00106:short chain dehydrogenase
Lachesis_group0__13_contigs__l.g7763	409.2854	74.88433	2.451825	0.001618	0.026804	1077	- && O31186.1 RecName: Full=Alcohol dehydrogenase && PF08240:Alcohol dehydrogenase GroES-like domain|PF00107:Zinc-binding dehydrogenase
Lachesis_group0__13_contigs__l.g7223	20806.32	3841.214	2.437452	2.35E-07	6.88E-05	546	- && P16934.1 RecName: Full=Fruiting body protein SC4; AltName: Full=Hydrophobin SC4; Flags: Precursor && PF01185:Fungal hydrophobin
Lachesis_group0__13_contigs__l.g4839	1083.104	201.5215	2.426664	1.98E-05	0.001364	1125	- && Q2HEW4.1 RecName: Full=Enoyl reductase CHGG_01240; AltName: Full=Chaetoglobosin biosynthesis protein CHGG_01240 && PF08240:Alcohol dehydrogenase GroES-like domain|PF00107:Zinc-binding dehydrogenase
Lachesis_group0__13_contigs__l.g3748	9155.529	1728.08	2.405588	0.000698	0.015942	3198	- && D4AUF1.1 RecName: Full=WSC domain-containing protein ARB_07867; Flags: Precursor && PF01822:WSC domain|PF07250:Glyoxal oxidase N-terminus|PF09118:Domain of unknown function (DUF1929)

Lachesis_group0__13_contigs__l.g486	65152.14	12335.11	2.401054	0.000124	0.004784	1758	- && Q54DY9.1 RecName: Full=Probable mitochondrial chaperone BCS1-B; AltName: Full=BCS1-like protein 2 && PF08740:BCS1 N terminal|PF00004:ATPase family associated with various cellular activities (AAA)
Lachesis_group0__13_contigs__l.g794	5571.003	1062.278	2.390846	2.43E-05	0.001526	1977	- && - && PF00004:ATPase family associated with various cellular activities (AAA)
Lachesis_group0__13_contigs__l.g7949	292.0375	58.13174	2.330649	0.000453	0.011527	1521	- && A9JPE2.1 RecName: Full=Cytochrome P450 monooxygenase atmQ; AltName: Full=Aflatrem synthesis protein Q && PF00067:Cytochrome P450
Lachesis_group0__13_contigs__l.g1725	361.0781	72.99683	2.307705	0.000265	0.008193	1023	- && Q09923.1 RecName: Full=Aldo-keto reductase yakc [NADP(+)] && PF00248:Aldo/keto reductase family
Lachesis_group0__13_contigs__l.g8360	543.5923	110.2198	2.300908	1.24E-05	0.000945	699	- && Q6NUE2.1 RecName: Full=Carbonyl reductase family member 4; AltName: Full=3-ketoacyl-[acyl-carrier-protein] reductase beta subunit; Short=KAR beta subunit; AltName: Full=3-oxoacyl-[acyl-carrier-protein] reductase; AltName: Full=Quinone reductase CBR4 && PF00106:short chain dehydrogenase
Lachesis_group0__13_contigs__l.g4973	12945.71	2630.628	2.298986	0.001433	0.025468	741	- && - && PF12766:Pyridoxamine 5'-phosphate oxidase
Lachesis_group0__13_contigs__l.g9130	917.0067	189.7129	2.273865	0.000226	0.00731	2238	- && - && PF00350:Dynamin family
Lachesis_group0__13_contigs__l.g717	2013.784	420.7029	2.259215	0.000274	0.008346	2310	- && Q942X8.1 RecName: Full=Probable potassium transporter 2; AltName: Full=OsHAK2 && PF02705:K+ potassium transporter
Lachesis_group0__13_contigs__l.g6878	1837.259	389.069	2.239615	0.000474	0.011894	1164	- && O74750.1 RecName: Full=UDP-N-acetylglucosamine transporter yea4 && PF08449:UAA transporter family

Lachesis_group0__13_contigs__l.g7267	310.5116	65.95913	2.237097	0.003131	0.039577	1887	- && P36619.2 RecName: Full=Leptomycin B resistance protein pmd1 && PF00005:ABC transporter
Lachesis_group0__13_contigs__l.g7222	6097.091	1300.382	2.229179	1.56E-05	0.001121	381	- && P16934.1 RecName: Full=Fruiting body protein SC4; AltName: Full=Hydrophobin SC4; Flags: Precursor && PF01185:Fungal hydrophobin
Lachesis_group0__13_contigs__l.g10103	793.954	171.1841	2.213821	0.001622	0.026804	1041	- && Q96NR8.3 RecName: Full=Retinol dehydrogenase 12; AltName: Full=All-trans and 9-cis retinol dehydrogenase; AltName: Full=Short chain dehydrogenase/reductase family 7C member 2 && PF00106:short chain dehydrogenase
Lachesis_group0__13_contigs__l.g1959	3139.465	700.4073	2.164323	1.08E-05	0.000909	873	- && - && PF13419:Haloacid dehalogenase-like hydrolase
Lachesis_group0__13_contigs__l.g10628	1553.061	348.039	2.158537	0.000197	0.006685	1728	- && - && PF00026:Eukaryotic aspartyl protease
Lachesis_group0__13_contigs__l.g9779	257.1282	59.6321	2.110118	0.00148	0.025764	3048	- && Q9M3B4.1 RecName: Full=Protein ROOT INITIATION DEFECTIVE 3; AltName: Full=Root initiation defective 3 protein; AltName: Full=WD-40 repeat-containing protein RID3 && PF00400:WD domain, G-beta repeat
Lachesis_group0__13_contigs__l.g530	2439.639	567.7317	2.10352	4.93E-05	0.002538	1044	- && Q9UT59.1 RecName: Full=Putative uncharacterized oxidoreductase C513.07 && PF01370:NAD dependent epimerase/dehydratase family
Lachesis_group0__13_contigs__l.g582	5836.294	1360.479	2.101058	1.61E-07	5.02E-05	636	- && - && PF05730:CFEM domain
Lachesis_group0__13_contigs__l.g3192	1099.135	260.6327	2.076545	0.004349	0.048845	789	- && - && PF13302:Acetyltransferase (GNAT) domain
Lachesis_group0__13_contigs__l.g3514	4145.831	1000.668	2.050922	0.000529	0.012805	1473	- && Q6UEF1.1 RecName: Full=Oxidoreductase AflY; AltName: Full=Aflatoxin biosynthesis protein Y && PF14027:Protein of unknown function (DUF4243)

Lachesis_group0__13_contigs__l.g446	430.1888	105.0372	2.036487	8.11E-06	0.000759	3243	- && A0A0D2YG01.1 RecName: Full=Non-canonical non-ribosomal peptide synthetase FUB8; AltName: Full=Fusaric acid biosynthesis protein 8 && PF07993:Male sterility protein|PF00501:AMP-binding enzyme
Lachesis_group0__13_contigs__l.g8361	63.09911	15.56413	2.026889	0.003078	0.039124	1023	- && - && PF01040:UbiA prenyltransferase family
Lachesis_group0__13_contigs__l.g9752	1716.527	438.1697	1.969969	0.001497	0.025804	375	- && O95563.1 RecName: Full=Mitochondrial pyruvate carrier 2; AltName: Full=Brain protein 44 && PF03650:Uncharacterised protein family (UPF0041)
Lachesis_group0__13_contigs__l.g4508	258.4731	66.00924	1.969658	3.40E-05	0.001909	903	- && - && PF05368:NmrA-like family
Lachesis_group0__13_contigs__l.g3311	5908.554	1518.655	1.959915	3.96E-07	0.0001	3147	- && Q9P6I2.2 RecName: Full=Cys-Gly metallodipeptidase dug1; AltName: Full=GSH degradosomal complex subunit DUG1 && PF07687:Peptidase dimerisation domain|PF01546:Peptidase family M20/M25/M40|PF02666:Phosphatidylserine decarboxylase
Lachesis_group0__13_contigs__l.g5414	221.1733	57.98133	1.936282	2.83E-05	0.001647	2406	- && Q54DU5.1 RecName: Full=von Willebrand factor A domain-containing protein DDB_G0292028 && PF13768:von Willebrand factor type A domain|PF08487:Vault protein inter-alpha-trypsin domain
Lachesis_group0__13_contigs__l.g9091	2283.594	602.1807	1.923424	5.58E-05	0.002753	1698	- && P53388.1 RecName: Full=Dicarboxylic amino acid permease && PF00324:Amino acid permease
Lachesis_group0__13_contigs__l.g8242	929.5056	248.4503	1.903556	0.000464	0.011739	1185	- && - && PF00652:Ricin-type beta-trefoil lectin domain
Lachesis_group0__13_contigs__l.g11175	2543.749	682.5963	1.897984	0.001239	0.02326	1080	- && - && PF13417:Glutathione S-transferase, N-terminal domain
Lachesis_group0__13_contigs__l.g6456	149.8348	40.59661	1.890279	0.000895	0.01893	903	- && - && PF08241:Methyltransferase domain

Lachesis_group0__13_contigs__l.g4498	19247.41	5212.87	1.884566	0.000268	0.008264	1056	- && Q9USW3.1 RecName: Full=Probable glycosidase C21B10.07 && PF00722:Glycosyl hydrolases family 16
Lachesis_group0__13_contigs__l.g3604	3738.334	1013.271	1.883656	0.000396	0.010569	1461	- && P53322.1 RecName: Full=High-affinity nicotinic acid transporter; AltName: Full=Nicotinic acid permease && PF07690:Major Facilitator Superfamily
Lachesis_group0__13_contigs__l.g11667	791.3014	215.4133	1.877453	0.000524	0.01275	864	- && Q5RDZ1.1 RecName: Full=Phenazine biosynthesis-like domain-containing protein && PF02567:Phenazine biosynthesis-like protein
Lachesis_group0__13_contigs__l.g2474	1894.94	519.4323	1.867209	0.000163	0.005918	1764	- && Q9FZ42.1 RecName: Full=Glucose and ribitol dehydrogenase homolog 1 && PF00106:short chain dehydrogenase
Lachesis_group0__13_contigs__l.g10211	2082.565	576.0918	1.854176	3.28E-05	0.00185	1995	- && D2XV59.1 RecName: Full=GTP-binding protein 1 && PF03144:Elongation factor Tu domain 2|PF00009:Elongation factor Tu GTP binding domain
Lachesis_group0__13_contigs__l.g1372	7252.389	2020.777	1.8437	0.00042	0.011018	2001	- && A2QEQ6.1 RecName: Full=Beta-glucuronidase; Short=GlcAase; AltName: Full=Beta-D-glucuronoside glucuronosohydrolase; Flags: Precursor && PF16862:Glycosyl hydrolase family 79 C-terminal beta domain
Lachesis_group0__13_contigs__l.g10952	654.7981	182.7954	1.841281	2.67E-05	0.001614	1830	- && P87024.1 RecName: Full=Beta-glucan synthesis-associated protein SKN1 && PF03935:Beta-glucan synthesis-associated protein (SKN1)
Lachesis_group0__13_contigs__l.g2225	4110.971	1147.217	1.841235	1.40E-06	0.000226	1257	- && Q01752.1 RecName: Full=Aryl-alcohol dehydrogenase [NADP(+)]; Short=AAD && PF00248:Aldo/keto reductase family
Lachesis_group0__13_contigs__l.g7520	8117.967	2266.482	1.84081	1.15E-05	0.000919	2304	- && P04842.2 RecName: Full=Alcohol oxidase 1; Short=AO 1; Short=AOX 1; AltName: Full=Methanol oxidase 1; Short=MOX 1 >F2QY27.1 RecName:

							Full=Alcohol oxidase 1; Short=AO 1; Short=AOX 1; AltName: Full=Methanol oxidase 1; Short=MOX 1 && PF00732:GMC oxidoreductase|PF05199:GMC oxidoreductase
Lachesis_group0__13_contigs__l.g1982	8706.871	2471.982	1.816473	0.0013	0.02406	330	- && - && PF12585:Protein of unknown function (DUF3759)
Lachesis_group0__13_contigs__l.g507	152.4613	43.67918	1.806962	0.000259	0.008054	1068	- && Q12535.1 RecName: Full=Pectinesterase; Short=PE; AltName: Full=Pectin methylesterase; Flags: Precursor && PF01095:Pectinesterase
Lachesis_group0__13_contigs__l.g2190	3604.419	1032.799	1.803456	1.51E-05	0.001108	747	- && - && PF04140:Isoprenylcysteine carboxyl methyltransferase (ICMT) family
Lachesis_group0__13_contigs__l.g6371	1770.668	507.5825	1.802879	0.00048	0.011978	1038	- && O14295.1 RecName: Full=Pyridoxal reductase; Short=PL reductase; Short=PL-red && PF00248:Aldo/keto reductase family
Lachesis_group0__13_contigs__l.g555	7746.631	2222.165	1.801698	2.98E-05	0.001701	2016	- && O74977.1 RecName: Full=VMS1 homolog C1827.04 && -
Lachesis_group0__13_contigs__l.g4086	742.3948	213.2045	1.799821	0.000481	0.011978	1626	- && O42615.1 RecName: Full=Threonine dehydratase, mitochondrial; AltName: Full=Threonine deaminase; Flags: Precursor && PF00291:Pyridoxal-phosphate dependent enzyme|PF00585:C-terminal regulatory domain of Threonine dehydratase
Lachesis_group0__13_contigs__l.g5415	19298.13	5556.654	1.796207	2.75E-05	0.001623	2916	- && Q54CQ8.1 RecName: Full=von Willebrand factor A domain-containing protein DDB_G0292740 && PF13768:von Willebrand factor type A domain|PF08487:Vault protein inter-alpha-trypsin domain

novel.1041	448.7607	129.4594	1.79511	0.000178	0.006271	879	- && P22579.2 RecName: Full=Transcriptional regulatory protein SIN3 && PF02671:Paired amphipathic helix repeat
Lachesis_group0__13_contigs__l.g55	245.8673	71.3334	1.788022	0.000213	0.007079	1023	- && P41838.1 RecName: Full=Poly(A)+ RNA export protein && -
Lachesis_group0__13_contigs__l.g3842	836.2325	243.5988	1.780337	6.54E-05	0.003093	1140	- && - && PF00498:FHA domain
Lachesis_group0__13_contigs__l.g2470	21990.51	6408.846	1.778783	0.000522	0.012733	2205	- && - && PF00004:ATPase family associated with various cellular activities (AAA)
Lachesis_group0__13_contigs__l.g2540	193.2128	56.8084	1.768789	0.001611	0.026751	1722	- && P59668.1 RecName: Full=Delta(12) fatty acid desaturase; AltName: Full=Delta-12 fatty acid desaturase && PF00487:Fatty acid desaturase
Lachesis_group0__13_contigs__l.g2924	122.9251	36.19256	1.76651	0.000355	0.009881	1719	- && - && PF15496:Domain of unknown function (DUF4646)
Lachesis_group0__13_contigs__l.g11363	4341.201	1276.493	1.766044	0.000584	0.013782	2277	- && Q9H4L5.1 RecName: Full=Oxysterol-binding protein-related protein 3; Short=ORP-3; Short=OSBP-related protein 3 && PF15409:Pleckstrin homology domain|PF01237:Oxysterol-binding protein
Lachesis_group0__13_contigs__l.g1862	417.2754	122.7962	1.763845	0.000364	0.01003	1551	- && Q0CJ57.1 RecName: Full=Cytochrome P450 monooxygenase atE; AltName: Full=Terreic acid biosynthesis cluster protein E && PF00067:Cytochrome P450
Lachesis_group0__13_contigs__l.g10095	9545.905	2829.227	1.754587	2.07E-05	0.001387	5748	- && P78811.2 RecName: Full=Probable UTP--glucose-1-phosphate uridylyltransferase; AltName: Full=UDP-glucose pyrophosphorylase; Short=UDPGP; Short=UGPase && PF08797:HIRAN domain|PF00176:SNF2 family N-terminal domain|PF00645:Poly(ADP-ribose) polymerase and DNA-Ligase Zn-finger region|PF01704:UTP--glucose-

							1-phosphate uridylyltransferase|PF00271:Helicase conserved C-terminal domain
Lachesis_group0__13_contigs__l.g4841	58.32864	17.37448	1.752169	0.001601	0.026693	978	- && Q8J0F9.1 RecName: Full=Deshydrogenase mlcG; AltName: Full=Compactin biosynthesis protein G; AltName: Full=Enoyl reductase && PF08240:Alcohol dehydrogenase GroES-like domain|PF00107:Zinc-binding dehydrogenase
Lachesis_group0__13_contigs__l.g6062	400.0581	119.2886	1.746229	0.003863	0.045231	837	- && Q7SDM8.1 RecName: Full=tRNA(His) guanylyltransferase; AltName: Full=tRNA-histidine guanylyltransferase && PF14413:Thg1 C terminal domain|PF04446:tRNAHis guanylyltransferase
Lachesis_group0__13_contigs__l.g4840	2528.555	755.3666	1.743229	9.43E-06	0.000842	1068	- && Q8FKG1.1 RecName: Full=S-(hydroxymethyl)glutathione dehydrogenase; AltName: Full=Alcohol dehydrogenase class-3; AltName: Full=Alcohol dehydrogenase class-III; AltName: Full=Glutathione-dependent formaldehyde dehydrogenase; Short=FALDH; Short=FDH; Short=GSH-FDH && PF00107:Zinc-binding dehydrogenase|PF08240:Alcohol dehydrogenase GroES-like domain
Lachesis_group0__13_contigs__l.g2205	7151.252	2144.38	1.737713	0.000114	0.004565	3927	- && B0D4J6.1 RecName: Full=Ubiquitin-related modifier 1 && PF09138:Urm1 (Ubiquitin related modifier)
Lachesis_group0__13_contigs__l.g599	574.5892	172.8303	1.734238	0.002563	0.034887	765	- && O14283.2 RecName: Full=Transcription factor prr1; AltName: Full=Pombe response regulator 1 && PF00447:HSF-type DNA-binding

Lachesis_group0__13_contigs__l.g2538	228.2252	68.95698	1.728332	0.001791	0.028386	1524	- && A1DA63.1 RecName: Full=Fumitremorgin C synthase; AltName: Full=Fumitremorgin biosynthesis protein E && PF00067:Cytochrome P450
Lachesis_group0__13_contigs__l.g4879	17845.05	5431.546	1.716086	0.000169	0.006029	738	- && O94476.1 RecName: Full=Eukaryotic translation initiation factor 6; Short=eIF-6 && PF01912:eIF-6 family
Lachesis_group0__13_contigs__l.g3853	66.08031	20.10123	1.715627	0.001971	0.030218	435	- && P16933.2 RecName: Full=Fruiting body protein SC3; AltName: Full=Hydrophobin SC3; Flags: Precursor && PF01185:Fungal hydrophobin
Lachesis_group0__13_contigs__l.g3567	508.6763	155.0722	1.71514	0.00147	0.025764	915	- && A8NWP2.2 RecName: Full=NAD-dependent protein deacylase; AltName: Full=Regulatory protein SIR2 homolog 5; Flags: Precursor && PF02146:Sir2 family
Lachesis_group0__13_contigs__l.g6866	167.8294	51.47132	1.703842	5.40E-05	0.002692	1893	- && - && PF13578:Methyltransferase domain|PF01501:Glycosyl transferase family 8
Lachesis_group0__13_contigs__l.g258	663.7079	203.8254	1.703747	0.001151	0.022282	4773	- && Q06625.1 RecName: Full=Glycogen debranching enzyme; AltName: Full=Glycogen debrancher; Includes: RecName: Full=4-alpha-glucanotransferase; AltName: Full=Oligo-1,4-1,4-glucantransferase; Includes: RecName: Full=Amylo-alpha-1,6-glucosidase; Short=Amylo-1,6-glucosidase; AltName: Full=Dextrin 6-alpha-D-glucosidase && PF06202:Amylo-alpha-1,6-glucosidase|PF14701:glucanotransferase domain of human glycogen debranching enzyme|PF14699:N-terminal domain from the human glycogen debranching enzyme|PF14702:central domain of human glycogen debranching enzyme
Lachesis_group0__13_contigs__l.g2907	456.543	141.7147	1.688877	0.000366	0.01003	1653	- && - && PF00011:Hsp20/alpha crystallin family

Lachesis_group0__13_contigs__l.g2713	920.7412	285.6345	1.688334	0.002976	0.038348	738	- && Q6P0H7.1 RecName: Full=Carbonyl reductase family member 4; AltName: Full=3-ketoacyl-[acyl-carrier-protein] reductase beta subunit; Short=KAR beta subunit; AltName: Full=3-oxoacyl-[acyl-carrier-protein] reductase; AltName: Full=Quinone reductase CBR4 && PF00106:short chain dehydrogenase
Lachesis_group0__13_contigs__l.g1113	7045.346	2188.058	1.686979	0.002296	0.032895	1857	- && Q2HXK9.2 RecName: Full=Respiratory burst oxidase homolog protein D; AltName: Full=NADPH oxidase RBOHD; AltName: Full=StRBOHD && PF08030:Ferric reductase NAD binding domain|PF01794:Ferric reductase like transmembrane component|PF08022:FAD-binding domain
Lachesis_group0__13_contigs__l.g3790	31176.93	9717.71	1.68182	0.002418	0.033962	2958	- && - && PF13632:Glycosyl transferase family group 2
Lachesis_group0__13_contigs__l.g1802	1166.277	363.6254	1.681063	0.001369	0.024857	1833	- && Q9U9A3.2 RecName: Full=Serine/threonine-protein phosphatase 6 catalytic subunit; Short=PP6C && PF00248:Aldo/keto reductase family
Lachesis_group0__13_contigs__l.g8366	81158.93	25505.34	1.669951	0.001962	0.030126	1593	- && P16934.1 RecName: Full=Fruiting body protein SC4; AltName: Full=Hydrophobin SC4; Flags: Precursor && PF01185:Fungal hydrophobin
Lachesis_group0__13_contigs__l.g4932	96.7143	31.46505	1.620757	0.001547	0.026227	1347	- && - && PF00069:Protein kinase domain
Lachesis_group0__13_contigs__l.g2537	271.9026	88.43091	1.618773	0.001328	0.024348	1293	- && P59668.1 RecName: Full=Delta(12) fatty acid desaturase; AltName: Full=Delta-12 fatty acid desaturase && PF00487:Fatty acid desaturase
Lachesis_group0__13_contigs__l.g3569	667.9259	219.1908	1.607548	0.001155	0.022308	3426	- && P34422.2 RecName: Full=Dipeptidyl peptidase family member 6 && PF00326:Prolyl oligopeptidase family

Lachesis_group0__13_contigs__l.g2204	45765.6	15031.17	1.606327	0.001729	0.027854	1914	- && P39992.1 RecName: Full=Uncharacterized protein YEL023C && PF09994:Uncharacterized alpha/beta hydrolase domain (DUF2235)
Lachesis_group0__13_contigs__l.g1471	1206.828	396.6718	1.605553	0.00074	0.016493	1383	- && - && PF00107:Zinc-binding dehydrogenase|PF16884:N-terminal domain of oxidoreductase
Lachesis_group0__13_contigs__l.g2917	288.1901	95.36379	1.596704	0.001621	0.026804	510	- && P22579.2 RecName: Full=Transcriptional regulatory protein SIN3 && PF02671:Paired amphipathic helix repeat
Lachesis_group0__13_contigs__l.g856	2480.835	821.4314	1.59492	0.000704	0.016047	2412	- && Q09922.1 RecName: Full=Uncharacterized transcriptional regulatory protein C1F7.11c && PF04082:Fungal specific transcription factor domain
Lachesis_group0__13_contigs__l.g2322	687.0362	228.7593	1.586476	0.00217	0.031913	915	- && I7LRH3.1 RecName: Full=Cytochrome P450 monooxygenase mpaDE; AltName: Full=Mycophenolic acid synthesis fusion protein DE; Flags: Precursor && PF00753:Metallo-beta-lactamase superfamily
Lachesis_group0__13_contigs__l.g1677	31744.77	10605.1	1.581777	7.00E-06	0.000698	468	- && - && PF16850:Peptidase inhibitor I66
Lachesis_group0__13_contigs__l.g5973	6578.61	2207.604	1.575305	3.71E-05	0.00203	3084	- && Q9ZVH4.1 RecName: Full=Cullin-3A; Short=AtCUL3a && PF13417:Glutathione S-transferase, N-terminal domain|PF00888:Cullin family|PF10557:Cullin protein neddylation domain
Lachesis_group0__13_contigs__l.g7966	287.2603	96.5396	1.57455	0.000956	0.019774	2697	- && Q13546.3 RecName: Full=Receptor-interacting serine/threonine-protein kinase 1; AltName: Full=Cell death protein RIP; AltName: Full=Receptor-interacting protein 1; Short=RIP-1; AltName: Full=Serine/threonine-protein kinase RIP && PF07714:Protein tyrosine kinase

Lachesis_group0__13_contigs__l.g8875	620.9468	208.7047	1.573673	4.27E-05	0.002328	1626	- && Q06402.1 RecName: Full=1-aminocyclopropane-1-carboxylate synthase 2; Short=ACC synthase 2; AltName: Full=S-adenosyl-L-methionine methylthioadenosine-lyase 2 && PF00155:Aminotransferase class I and II
Lachesis_group0__13_contigs__l.g8542	514.496	174.4196	1.561811	0.000279	0.008463	2796	- && Q9QZ81.2 RecName: Full=Protein argonaute-2; Short=Argonaute2; AltName: Full=Argonaute RISC catalytic component 2; AltName: Full=Eukaryotic translation initiation factor 2C 2; Short=eIF-2C 2; Short=eIF2C 2; AltName: Full=Golgi ER protein 95 kDa; Short=GERp95; AltName: Full=Protein slicer && PF16486:N-terminal domain of argonaute|PF16487:Mid domain of argonaute|PF02171:Piwi domain|PF08699:Argonaute linker 1 domain|PF02170:PAZ domain
Lachesis_group0__13_contigs__l.g541	1811.531	614.352	1.560374	0.000734	0.016402	2256	- && O74396.3 RecName: Full=Vacuolar protein sorting-associated protein 9a; AltName: Full=Vacuolar protein-targeting protein 9a && PF02204:Vacuolar sorting protein 9 (VPS9) domain|PF02845:CUE domain
Lachesis_group0__13_contigs__l.g7421	5864.95	2005.113	1.548561	0.001844	0.028895	408	- && Q91W82.1 RecName: Full=Ubiquitin-conjugating enzyme E2 E2; AltName: Full=E2 ubiquitin-conjugating enzyme E2; AltName: Full=Ubiquitin carrier protein E2; AltName: Full=Ubiquitin-protein ligase E2 && PF00179:Ubiquitin-conjugating enzyme
Lachesis_group0__13_contigs__l.g8166	221.3645	75.88143	1.546374	0.000904	0.018959	714	- && O94628.1 RecName: Full=Uncharacterized methyltransferase C1347.09 && PF13489:Methyltransferase domain

Lachesis_group0__13_contigs__l.g485	1794.125	615.2175	1.544504	0.004267	0.04834	732	- && Q9C2M6.1 RecName: Full=Putative dolichyldiphosphatase; AltName: Full=Dolichyl pyrophosphate phosphatase && PF01569:PAP2 superfamily
Lachesis_group0__13_contigs__l.g10938	1182.977	406.5832	1.541035	0.001662	0.027129	9411	- && Q9P7V2.1 RecName: Full=ATP-binding cassette transporter abc4; Short=ABC transporter abc4; AltName: Full=ATP-energized glutathione S-conjugate pump abc4; AltName: Full=Glutathione S-conjugate-transporting ATPase abc4 && PF00664:ABC transporter transmembrane region|PF00005:ABC transporter
Lachesis_group0__13_contigs__l.g8840	683.3567	235.5304	1.537026	3.82E-06	0.000457	537	- && Q5I0K7.1 RecName: Full=UDP-N-acetylglucosamine transferase subunit ALG13 homolog; AltName: Full=Glycosyltransferase 28 domain-containing protein 1 && PF04101:Glycosyltransferase family 28 C-terminal domain
Lachesis_group0__13_contigs__l.g4512	655.7518	226.944	1.532068	0.002547	0.034806	1791	- && Q9SYK0.1 RecName: Full=Beta-hexosaminidase 2; AltName: Full=Beta-GlcNAcase 2; AltName: Full=Beta-N-acetylhexosaminidase 2; AltName: Full=Beta-hexosaminidase 3; Short=AtHEX3; AltName: Full=N-acetyl-beta-glucosaminidase 2; Flags: Precursor && PF00728:Glycosyl hydrolase family 20, catalytic domain|PF14845:beta-acetyl hexosaminidase like
Lachesis_group0__13_contigs__l.g8128	5641.382	1961.498	1.524138	0.002897	0.037553	1020	- && Q8CG76.3 RecName: Full=Aflatoxin B1 aldehyde reductase member 2; AltName: Full=Succinic semialdehyde reductase; Short=SSA reductase && PF00248:Aldo/keto reductase family

Lachesis_group0__13_contigs__l.g3608	29725.56	10426.54	1.511452	0.001497	0.025804	2283	- && - && PF11443:Domain of unknown function (DUF2828)
Lachesis_group0__13_contigs__l.g147	1011.06	357.7212	1.498888	0.000437	0.011312	1377	- && Q9UT44.1 RecName: Full=Probable gamma-glutamyl phosphate reductase; Short=GPR; AltName: Full=Glutamate-5-semialdehyde dehydrogenase; Short=GSA dehydrogenase; AltName: Full=Glutamyl-gamma-semialdehyde dehydrogenase && PF00171:Aldehyde dehydrogenase family
Lachesis_group0__13_contigs__l.g2505	7118.971	2523.367	1.496319	0.000132	0.005006	1947	- && - && PF01565:FAD binding domain
Lachesis_group0__13_contigs__l.g5496	767.823	274.5737	1.484545	0.001824	0.028623	1872	- && Q06466.1 RecName: Full=Putative vacuolar protein sorting-associated protein TDA6; AltName: Full=Topoisomerase I damage affected protein 6 && PF06101:Plant protein of unknown function (DUF946)
Lachesis_group0__13_contigs__l.g5110	19611.89	7028.303	1.480492	0.001365	0.024825	744	- && - && PF13430:Domain of unknown function (DUF4112)
Lachesis_group0__13_contigs__l.g10443	658.2803	236.4358	1.47797	0.003506	0.042972	1341	- && Q53552.1 RecName: Full=Salicylate hydroxylase; AltName: Full=Salicylate 1-monooxygenase && PF01494:FAD binding domain
Lachesis_group0__13_contigs__l.g11592	4229.839	1533.046	1.464186	0.000135	0.005071	1065	- && Q550W6.1 RecName: Full=GDP-fucose transporter 1; AltName: Full=Solute carrier family 35 member C1 homolog && PF03151:Triose-phosphate Transporter family
Lachesis_group0__13_contigs__l.g10094	2186.274	803.4076	1.444667	0.001752	0.027915	1629	- && - && PF00149:Calcineurin-like phosphoesterase
Lachesis_group0__13_contigs__l.g11223	1320.163	493.3524	1.420653	0.000388	0.010428	3888	- && Q9M8Y0.1 RecName: Full=Probable UDP-N-acetylglucosamine--peptide N-acetylglucosaminyltransferase SEC; AltName: Full=Protein SECRET AGENT && PF13844:Glycosyl transferase family 41|PF13424:Tetratricopeptide repeat

Lachesis_group0__13_contigs__l.g5202	2302.77	870.865	1.403104	0.000125	0.004784	1689	- && - && PF01435:Peptidase family M48
Lachesis_group0__13_contigs__l.g9601	412.9007	156.3878	1.401658	0.000716	0.016088	765	- && O74758.1 RecName: Full=Uncharacterized protein C17D11.03c && -
Lachesis_group0__13_contigs__l.g7235	282.218	107.512	1.393711	0.000477	0.011953	741	- && - && PF09747:Coiled-coil domain containing protein (DUF2052)
Lachesis_group0__13_contigs__l.g9610	6049.729	2321.969	1.38151	0.000101	0.004143	1476	- && Q9US44.1 RecName: Full=Uncharacterized transporter C1002.16c && PF07690:Major Facilitator Superfamily
Lachesis_group0__13_contigs__l.g1329	227.5635	88.47321	1.365689	0.003476	0.042856	1680	- && - && PF01728:FtsJ-like methyltransferase
Lachesis_group0__13_contigs__l.g8732	526.9703	204.5751	1.364749	0.003169	0.039954	1620	- && Q04792.1 RecName: Full=Glutamate decarboxylase; Short=GAD && PF00282:Pyridoxal-dependent decarboxylase conserved domain
Lachesis_group0__13_contigs__l.g2640	146.1599	57.45716	1.35097	0.003419	0.042426	1311	- && A5H0J3.1 RecName: Full=Uracil catabolism protein 4; AltName: Full=Pyrimidine-degrading protein 14 && PF07958:Protein of unknown function (DUF1688)
Lachesis_group0__13_contigs__l.g3191	119.6982	47.10623	1.3478	0.002662	0.035682	789	- && - && PF13302:Acetyltransferase (GNAT) domain
Lachesis_group0__13_contigs__l.g9855	18954.21	7473.795	1.342579	0.00218	0.031964	366	- && P16934.1 RecName: Full=Fruiting body protein SC4; AltName: Full=Hydrophobin SC4; Flags: Precursor && PF01185:Fungal hydrophobin
Lachesis_group0__13_contigs__l.g7796	6127.37	2425.136	1.337295	0.003072	0.03912	1215	- && Q58EJ9.1 RecName: Full=Mitochondrial amidoxime-reducing component 1; Short=mARC1 && PF03476:MOSC N-terminal beta barrel domain|PF03473:MOSC domain
Lachesis_group0__13_contigs__l.g6455	1348.31	534.2622	1.335436	0.002233	0.032381	1674	- && - && PF02458:Transferase family
Lachesis_group0__13_contigs__l.g376	736.4146	291.9576	1.334832	6.49E-05	0.003084	822	- && - && PF10021:Uncharacterized protein conserved in bacteria (DUF2263)

Lachesis_group0__13_contigs__l.g8641	2724.832	1082.337	1.332304	0.003733	0.04416	2319	- && Q8VDZ4.1 RecName: Full=Palmitoyltransferase ZDHHC5; AltName: Full=Zinc finger DHHC domain-containing protein 5; Short=DHHC-5 && PF01529:DHHC palmitoyltransferase
Lachesis_group0__13_contigs__l.g9566	9368.621	3798.611	1.302358	0.003305	0.041279	4569	- && B0D360.1 RecName: Full=Adenylate kinase; AltName: Full=ATP-AMP transphosphorylase; AltName: Full=ATP:AMP phosphotransferase; AltName: Full=Adenylate kinase cytosolic and mitochondrial; AltName: Full=Adenylate monophosphate kinase && PF00005:ABC transporter|PF05191:Adenylate kinase, active site lid|PF00406:Adenylate kinase|PF01061:ABC-2 type transporter
Lachesis_group0__13_contigs__l.g2028	1568.703	639.4038	1.295214	0.002039	0.030999	4899	- && Q9P7V2.1 RecName: Full=ATP-binding cassette transporter abc4; Short=ABC transporter abc4; AltName: Full=ATP-energized glutathione S-conjugate pump abc4; AltName: Full=Glutathione S-conjugate-transporting ATPase abc4 && PF00005:ABC transporter|PF00664:ABC transporter transmembrane region
Lachesis_group0__13_contigs__l.g4392	1955.92	802.2756	1.285628	0.000247	0.00784	1056	- && Q9V3D2.1 RecName: Full=Oxygen-dependent coproporphyrinogen-III oxidase; Short=COX; Short=Coprogen oxidase; Short=Coproporphyrinogenase && PF01218:Coproporphyrinogen III oxidase
Lachesis_group0__13_contigs__l.g9965	1183.545	487.5326	1.27976	0.002501	0.0343	1008	- && Q09923.1 RecName: Full=Aldo-keto reductase yakc [NADP(+)] && PF00248:Aldo/keto reductase family

Lachesis_group0__13_contigs__l.g2107	1126.072	467.4024	1.269104	0.000628	0.014635	1377	- && Q9VVU5.1 RecName: Full=COP9 signalosome complex subunit 1b; Short=Dch1-2; Short=Signalosome subunit 1b && PF10602:26S proteasome subunit RPN7|PF01399:PCI domain
Lachesis_group0__13_contigs__l.g11144	2466.188	1025.713	1.265755	0.0032	0.040242	2019	- && Q6MG06.1 RecName: Full=Guanine nucleotide-binding protein-like 1 && PF01926:50S ribosome-binding GTPase
Lachesis_group0__13_contigs__l.g4543	964.4284	401.7545	1.263607	0.001509	0.025804	3582	- && Q0CL79.2 RecName: Full=Cytokinesis protein sepH; AltName: Full=Serine/threonine-protein kinase sepH && PF00069:Protein kinase domain
Lachesis_group0__13_contigs__l.g1803	470.2406	198.7196	1.242669	0.000369	0.01003	1038	- && B9WYE6.1 RecName: Full=Versiconal hemiacetal acetate reductase; AltName: Full=VHA reductase && PF00248:Aldo/keto reductase family
Lachesis_group0__13_contigs__l.g10325	3907.411	1652.629	1.241567	0.001316	0.024267	2175	- && P0CQ68.1 RecName: Full=Protein arginine N-methyltransferase 2; AltName: Full=Protein-arginine N5-methyltransferase; AltName: Full=Type IV protein arginine N-methyltransferase; Short=Type IV PRMT >P0CQ69.1 RecName: Full=Protein arginine N-methyltransferase 2; AltName: Full=Protein-arginine N5-methyltransferase; AltName: Full=Type IV protein arginine N-methyltransferase; Short=Type IV PRMT && -
Lachesis_group0__13_contigs__l.g8171	78253.96	33147.85	1.239256	0.002079	0.031115	1608	- && P39992.1 RecName: Full=Uncharacterized protein YEL023C && PF09994:Uncharacterized alpha/beta hydrolase domain (DUF2235)
Lachesis_group0__13_contigs__l.g2596	215.1004	91.47127	1.234754	0.001087	0.021612	1221	- && P32386.2 RecName: Full=ATP-dependent bile acid permease && PF00005:ABC transporter

Lachesis_group0__13_contigs__l.g9370	31015.55	13245.25	1.227524	0.000252	0.007951	1314	- && Q6FJN0.1 RecName: Full=Multiprotein-bridging factor 1 && PF01381:Helix-turn-helix|PF08523:Multiprotein bridging factor 1
Lachesis_group0__13_contigs__l.g3898	4812.054	2062.566	1.222269	0.003996	0.045892	2256	- && - && PF11901:Protein of unknown function (DUF3421)|PF00561:alpha/beta hydrolase fold
Lachesis_group0__13_contigs__l.g2327	1845.028	793.1559	1.218082	0.001477	0.025764	858	- && - && PF01424:R3H domain
Lachesis_group0__13_contigs__l.g6564	598.7725	259.6895	1.206353	0.00434	0.048832	1521	- && - && PF13229:Right handed beta helix region
Lachesis_group0__13_contigs__l.g8351	812.5481	356.9993	1.187281	0.003997	0.045892	2157	- && - && PF13417:Glutathione S-transferase, N-terminal domain
Lachesis_group0__13_contigs__l.g8150	725.8743	319.4826	1.184277	0.001319	0.024268	1389	- && Q96V64.1 RecName: Full=Glucan 1,3-beta-glucosidase; AltName: Full=Exo-1,3-beta-glucanase; Flags: Precursor && PF00150:Cellulase (glycosyl hydrolase family 5)
Lachesis_group0__13_contigs__l.g8168	600900.8	267476.6	1.167714	0.001926	0.029865	3435	- && Q4WY82.2 RecName: Full=Linoleate 10R-lipoxygenase; AltName: Full=Cyclooxygenase-like fatty acid oxygenase; AltName: Full=Fatty acid oxygenase ppoC; AltName: Full=Linoleate 10R-dioxygenase; Short=10R-DOX; AltName: Full=Psi-producing oxygenase C; Short=AfPpoC && PF03098:Animal haem peroxidase|PF00067:Cytochrome P450
Lachesis_group0__13_contigs__l.g8513	1881.205	852.0801	1.142602	0.000443	0.011373	648	- && P92963.1 RecName: Full=Ras-related protein RABB1c; Short=AtRABB1c; AltName: Full=Ras-related protein Rab2A; Short=AtRab2A && PF00071:Ras family
Lachesis_group0__13_contigs__l.g10472	5148.343	2334.648	1.140932	0.001211	0.022921	2484	- && A3KMV8.1 RecName: Full=Ran-binding protein 10; Short=RanBP10 && PF00622:SPRY domain|PF10607:CTLH/CRA C-terminal to LisH motif domain

Lachesis_group0__13_contigs__l.g2551	666.0821	302.2247	1.140444	0.002443	0.033962	1098	- && B8M9K5.1 RecName: Full=2-oxoglutarate-dependent dioxygenase tropC; AltName: Full=Tropolone synthesis protein C && PF14226:non-haem dioxygenase in morphine synthesis N-terminal|PF03171:2OG-Fe(II) oxygenase superfamily
Lachesis_group0__13_contigs__l.g7155	2602.697	1183.263	1.137355	0.002488	0.03423	1293	- && Q12042.1 RecName: Full=Vacuolar membrane protein YPL162C && PF12400:Vaculolar membrane protein
Lachesis_group0__13_contigs__l.g4838	584.7067	266.2131	1.135007	0.003128	0.039577	1986	- && Q8J0F9.1 RecName: Full=Deshydrogenase mlcG; AltName: Full=Compactin biosynthesis protein G; AltName: Full=Enoyl reductase && PF00107:Zinc-binding dehydrogenase|PF08240:Alcohol dehydrogenase GroES-like domain
Lachesis_group0__13_contigs__l.g6373	1405.912	641.19	1.132652	0.00118	0.022597	1047	- && O14295.1 RecName: Full=Pyridoxal reductase; Short=PL reductase; Short=PL-red && PF00248:Aldo/keto reductase family
Lachesis_group0__13_contigs__l.g9004	1015.125	464.9459	1.126746	0.002383	0.033877	813	- && Q5B5P1.1 RecName: Full=Probable rhamnogalacturonate lyase C; Flags: Precursor && PF00149:Calcineurin-like phosphoesterase
Lachesis_group0__13_contigs__l.g1892	9426.698	4320.509	1.125539	0.001738	0.027854	4812	- && P39531.4 RecName: Full=Recyclin-1 && PF12449:Protein of unknown function (DUF3684)|PF07393:Exocyst complex component Sec10
Lachesis_group0__13_contigs__l.g787	1906.786	875.9689	1.122346	0.001634	0.026859	1779	- && P79106.1 RecName: Full=Platelet-activating factor acetylhydrolase 2, cytoplasmic; AltName: Full=Serine-dependent phospholipase A2; Short=SD-PLA2 && PF03403:Platelet-activating factor acetylhydrolase, isoform II

Lachesis_group0__13_contigs__l.g10708	512.7933	235.7357	1.121433	0.002552	0.034806	1914	- && Q3SZH7.3 RecName: Full=Leukotriene A-4 hydrolase; Short=LTA-4 hydrolase; AltName: Full=Leukotriene A(4) hydrolase && PF01433:Peptidase family M1|PF09127:Leukotriene A4 hydrolase, C-terminal
Lachesis_group0__13_contigs__l.g3241	3737.54	1730.529	1.111017	0.004175	0.047497	1587	- && Q99NF1.1 RecName: Full=Beta,beta-carotene 9',10'-oxygenase; AltName: Full=B-diox-II; AltName: Full=Beta-carotene dioxygenase 2 && PF03055:Retinal pigment epithelial membrane protein
Lachesis_group0__13_contigs__l.g2884	2336.326	1081.837	1.110785	0.002335	0.033285	1236	- && Q9Y7K9.3 RecName: Full=PARP-type zinc finger-containing protein C2A9.07c && PF00645:Poly(ADP-ribose) polymerase and DNA-Ligase Zn-finger region
Lachesis_group0__13_contigs__l.g8709	14089.5	6553.469	1.104313	0.001649	0.027035	615	- && P33723.1 RecName: Full=GTP-binding protein ypt1 && PF00071:Ras family
Lachesis_group0__13_contigs__l.g8163	2068.567	979.2101	1.079202	0.003431	0.042521	1107	- && - && PF00651:BTB/POZ domain
Lachesis_group0__13_contigs__l.g10129	478.3163	227.3035	1.073928	0.002987	0.038349	666	- && Q5F389.2 RecName: Full=WW domain-containing oxidoreductase && PF00106:short chain dehydrogenase
Lachesis_group0__13_contigs__l.g11070	2396.744	1144.121	1.06698	0.001719	0.02776	3867	- && Q5F418.1 RecName: Full=26S proteasome non-ATPase regulatory subunit 1; AltName: Full=26S proteasome regulatory subunit RPN2; AltName: Full=26S proteasome regulatory subunit S1 && PF02826:D-isomer specific 2-hydroxyacid dehydrogenase, NAD binding domain|PF13646:HEAT repeats|PF00389:D-isomer specific 2-hydroxyacid dehydrogenase, catalytic domain|PF01851:Proteasome/cyclosome repeat

Lachesis_group0__13_contigs__l.g4638	488.7191	235.0844	1.056071	0.002487	0.03423	3033	- && O13992.1 RecName: Full=Meiotically up-regulated gene 56 protein && PF15404:Pleckstrin homology domain
Lachesis_group0__13_contigs__l.g10615	1727.569	834.9073	1.04917	0.002421	0.033962	2931	- && Q9EQH3.1 RecName: Full=Vacuolar protein sorting-associated protein 35; AltName: Full=Maternal-embryonic 3; AltName: Full=Vesicle protein sorting 35 && PF03635:Vacuolar protein sorting-associated protein 35
Lachesis_group0__13_contigs__l.g6026	334.3498	161.7083	1.04865	0.003659	0.043921	2487	- && P0CM47.1 RecName: Full=pH-response regulator protein palA/RIM20 >P0CM46.1 RecName: Full=pH-response regulator protein palA/RIM20 && PF03097:BRO1-like domain|PF13949:ALIX V-shaped domain binding to HIV
Lachesis_group0__13_contigs__l.g8524	5234.401	2535.39	1.045773	0.004456	0.049749	2562	- && P06208.1 RecName: Full=2-isopropylmalate synthase; AltName: Full=Alpha-IPM synthase; AltName: Full=Alpha-isopropylmalate synthase && PF00682:HMGL-like|PF08502:LeuA allosteric (dimerisation) domain
Lachesis_group0__13_contigs__l.g9369	1495.585	725.9296	1.043126	0.002821	0.036909	840	- && O14172.1 RecName: Full=Siroheme biosynthesis protein met8; Includes: RecName: Full=Precorrin-2 dehydrogenase; Includes: RecName: Full=Sirohydrochlorin ferrochelatase && PF14823:Sirohaem biosynthesis protein C-terminal|PF13241:Putative NAD(P)-binding|PF14824:Sirohaem biosynthesis protein central
Lachesis_group0__13_contigs__l.g2877	668.6502	328.5699	1.025117	0.00287	0.037332	1311	- && - && PF04511:Der1-like family

Lachesis_group0__13_contigs__l.g4700	1347.21	664.0582	1.020746	0.002202	0.032175	2991	- && P87235.1 RecName: Full=DNA repair protein rhp42 && PF03835:Rad4 transglutaminase-like domain|PF10405:Rad4 beta-hairpin domain 3|PF10404:Rad4 beta-hairpin domain 2|PF10403:Rad4 beta-hairpin domain 1|PF01841:Transglutaminase-like superfamily
Lachesis_group0__13_contigs__l.g4837	1412.727	700.0937	1.01291	0.003552	0.043293	2142	- && Q8J0F9.1 RecName: Full=Deshydrogenase mlcG; AltName: Full=Compactin biosynthesis protein G; AltName: Full=Enoyl reductase && PF08240:Alcohol dehydrogenase GroES-like domain|PF00107:Zinc-binding dehydrogenase
Lachesis_group0__13_contigs__l.g8079	1763.616	3618.462	-1.03694	0.002757	0.036178	1650	- && - && PF11735:Cryptococcal mannosyltransferase 1
Lachesis_group0__13_contigs__l.g8182	611.4612	1270.005	-1.05478	0.002711	0.035948	2562	- && - && PF04082:Fungal specific transcription factor domain
Lachesis_group0__13_contigs__l.g10787	1914.28	3981.504	-1.05661	0.004362	0.04894	2169	- && Q9Z8P2.1 RecName: Full=3-oxoacyl-[acyl-carrier-protein] reductase FabG; AltName: Full=3-ketoacyl-acyl carrier protein reductase; AltName: Full=Beta-Ketoacyl-acyl carrier protein reductase; AltName: Full=Beta-ketoacyl-ACP reductase && PF00106:short chain dehydrogenase
Lachesis_group0__13_contigs__l.g5734	3337.29	7009.73	-1.07071	0.00335	0.041679	1470	- && Q9UQW9.1 RecName: Full=Ornithine decarboxylase; Short=ODC && PF02784:Pyridoxal-dependent decarboxylase, pyridoxal binding domain|PF00278:Pyridoxal-dependent decarboxylase, C-terminal sheet domain

Lachesis_group0__13_contigs__l.g6232	173.5222	365.5259	-1.07522	0.00175	0.027915	6039	- && - && PF11707:Ribosome 60S biogenesis N-terminal|PF16201:Nucleolar pre-ribosomal-associated protein 1
Lachesis_group0__13_contigs__l.g6012	803.9085	1719.23	-1.09682	0.003678	0.043921	2781	- && P87320.2 RecName: Full=Protein msp1, mitochondrial; Flags: Precursor && PF01031:Dynamin central region|PF00350:Dynamin family
Lachesis_group0__13_contigs__l.g1284	646.6349	1383.87	-1.09791	0.002391	0.033938	768	- && Q4PHE4.1 RecName: Full=Histone H2A.Z && PF16211:C-terminus of histone H2A|PF00125:Core histone H2A/H2B/H3/H4
Lachesis_group0__13_contigs__l.g9080	154.1983	332.4013	-1.10897	0.003578	0.043293	693	- && - && PF04930:FUN14 family
Lachesis_group0__13_contigs__l.g6435	519.0596	1124.69	-1.11594	0.00381	0.044675	2898	- && P87177.1 RecName: Full=Uncharacterized WD repeat-containing protein C3D6.12 && PF00400:WD domain, G-beta repeat|PF04003:Dip2/Utp12 Family
Lachesis_group0__13_contigs__l.g2248	515.8253	1123.958	-1.12365	0.001948	0.030072	5520	- && A3KMH1.2 RecName: Full=von Willebrand factor A domain-containing protein 8; Flags: Precursor && PF07728:AAA domain (dynein-related subfamily)|PF00249:Myb-like DNA-binding domain
Lachesis_group0__13_contigs__l.g10677	1278.511	2809.159	-1.13579	0.002049	0.031035	1863	- && P0CQ78.1 RecName: Full=ATP-dependent RNA helicase DBP3 && PF00270:DEAD/DEAH box helicase|PF00271:Helicase conserved C-terminal domain
Lachesis_group0__13_contigs__l.g1293	548.9393	1206.184	-1.136	0.003507	0.042972	1245	- && Q92925.3 RecName: Full=SWI/SNF-related matrix-associated actin-dependent regulator of chromatin subfamily D member 2; AltName: Full=60 kDa BRG-1/Brm-associated factor subunit B; AltName: Full=BRG1-associated factor 60B; Short=BAF60B && PF02201:SWIB/MDM2 domain

Lachesis_group0__13_contigs__l.g6183	402.6189	885.5784	-1.13749	0.004479	0.049896	1503	- && P0CR27.1 RecName: Full=RuvB-like helicase 1 >P0CR26.1 RecName: Full=RuvB-like helicase 1 && PF06068:TIP49 C-terminus
Lachesis_group0__13_contigs__l.g297	3119.874	6874.106	-1.13975	0.002721	0.035948	4278	- && P46056.1 RecName: Full=Carbamoyl-phosphate synthase arginine-specific large chain; AltName: Full=Arginine-specific carbamoyl-phosphate synthetase, ammonia chain && PF02786:Carbamoyl-phosphate synthase L chain, ATP binding domain|PF02787:Carbamoyl-phosphate synthetase large chain, oligomerisation domain
Lachesis_group0__13_contigs__l.g2106	777.175	1718.238	-1.14487	0.003239	0.04062	1431	- && Q9UUI4.1 RecName: Full=Uncharacterized protein C22F8.09 && PF07767:Nop53 (60S ribosomal biogenesis)
Lachesis_group0__13_contigs__l.g11229	3243.809	7175.404	-1.14541	0.002059	0.031035	3549	- && Q9UTF7.1 RecName: Full=Putative elongation of fatty acids protein 1; AltName: Full=3-keto acyl-CoA synthase SPAC1B2.03c; AltName: Full=Very-long-chain 3-oxoacyl-CoA synthase 1 && PF00168:C2 domain|PF00616:GTPase-activator protein for Ras-like GTPase|PF01151:GNS1/SUR4 family
Lachesis_group0__13_contigs__l.g5090	508.4679	1126.156	-1.14729	0.000806	0.017474	915	- && O94511.1 RecName: Full=Putative enoyl reductase C646.07c && PF02544:3-oxo-5-alpha-steroid 4-dehydrogenase
Lachesis_group0__13_contigs__l.g3442	1059.008	2348.533	-1.14915	0.002873	0.037332	2256	- && O42945.1 RecName: Full=Probable pre-mRNA-splicing factor ATP-dependent RNA helicase prp43 && PF07717:Oligonucleotide/oligosaccharide-binding (OB)-fold|PF00271:Helicase conserved C-terminal domain|PF00270:DEAD/DEAH box helicase|PF04408:Helicase associated domain (HA2)

Lachesis_group0__13_contigs__l.g8681	2272.83	5057.463	-1.15399	0.001553	0.026227	2808	- && Q8WZJ7.1 RecName: Full=C-1-tetrahydrofolate synthase, cytoplasmic; Short=C1-THF synthase; Includes: RecName: Full=Methylenetetrahydrofolate dehydrogenase; Includes: RecName: Full=Methenyltetrahydrofolate cyclohydrolase; Includes: RecName: Full=Formyltetrahydrofolate synthetase && PF01268:Formate--tetrahydrofolate ligase|PF02882:Tetrahydrofolate dehydrogenase/cyclohydrolase, NAD(P)-binding domain|PF00763:Tetrahydrofolate dehydrogenase/cyclohydrolase, catalytic domain
Lachesis_group0__13_contigs__l.g10296	1423.519	3182.178	-1.16058	0.003243	0.04062	2952	- && Q4P3W3.1 RecName: Full=ATP-dependent RNA helicase DBP10 && PF00270:DEAD/DEAH box helicase|PF08147:DBP10CT (NUC160) domain|PF00271:Helicase conserved C-terminal domain
Lachesis_group0__13_contigs__l.g2413	146.5505	327.7905	-1.1619	0.003928	0.04554	2898	- && P73505.1 RecName: Full=Isoleucine--tRNA ligase; AltName: Full=Isoleucyl-tRNA synthetase; Short=IleRS && PF08264:Anticodon-binding domain of tRNA|PF00133:tRNA synthetases class I (I, L, M and V)
Lachesis_group0__13_contigs__l.g2197	1167.069	2612.037	-1.16234	0.002986	0.038349	2337	- && - && PF13813:Membrane bound O-acyl transferase family
Lachesis_group0__13_contigs__l.g778	91.17238	204.3636	-1.1652	0.002729	0.036006	1626	- && - && PF02636:Putative S-adenosyl-L-methionine-dependent methyltransferase
Lachesis_group0__13_contigs__l.g9548	1022.292	2298.588	-1.16907	0.001504	0.025804	990	- && Q5XGI0.1 RecName: Full=Ribose-phosphate pyrophosphokinase 2; AltName: Full=Phosphoribosyl pyrophosphate synthase II; Short=PRS-II && PF14572:Phosphoribosyl synthetase-associated

							domain|PF13793:N-terminal domain of ribose phosphate pyrophosphokinase
Lachesis_group0__13_contigs__l.g10078	249.9321	561.944	-1.16947	0.002735	0.036038	1992	- && Q02354.2 RecName: Full=U3 small nucleolar RNA-associated protein 6; Short=U3 snoRNA-associated protein 6; AltName: Full=U three protein 6 && PF08640:U3 small nucleolar RNA-associated protein 6
Lachesis_group0__13_contigs__l.g1536	587.6752	1321.831	-1.16948	0.003005	0.038509	4551	- && Q9US40.1 RecName: Full=Uncharacterized amino-acid permease C1039.01 && PF13086:AAA domain|PF13520:Amino acid permease|PF13087:AAA domain
Lachesis_group0__13_contigs__l.g9079	1064.845	2400.52	-1.1729	0.002217	0.032348	2244	- && O94268.1 RecName: Full=25S rRNA (cytosine-C(5))-methyltransferase nop2; AltName: Full=Nucleolar protein 2 && PF01189:16S rRNA methyltransferase RsmF|PF17125:N-terminal domain of 16S rRNA methyltransferase RsmF
Lachesis_group0__13_contigs__l.g10125	314.3739	709.5501	-1.17441	0.002069	0.031048	6384	- && Q9CQG2.1 RecName: Full=Methyltransferase-like protein 16; AltName: Full=Methyltransferase 10 domain-containing protein && PF05971:Protein of unknown function (DUF890)
Lachesis_group0__13_contigs__l.g4147	122.7809	278.1506	-1.18036	0.001327	0.024348	2001	- && Q5ZLG0.1 RecName: Full=Acetoacetyl-CoA synthetase && PF00501:AMP-binding enzyme
Lachesis_group0__13_contigs__l.g11041	1576.47	3573.718	-1.18075	0.000755	0.016732	2637	- && P0CS79.1 RecName: Full=AdoMet-dependent rRNA methyltransferase SPB1; AltName: Full=2'-O-ribose RNA methyltransferase; AltName: Full=S-adenosyl-L-methionine-dependent methyltransferase >P0CS78.1 RecName: Full=AdoMet-dependent rRNA methyltransferase SPB1; AltName:

							Full=2'-O-ribose RNA methyltransferase; AltName: Full=S-adenosyl-L-methionine-dependent methyltransferase && PF07780:Spb1 C-terminal domain|PF11861:Domain of unknown function (DUF3381)|PF01728:FtsJ-like methyltransferase
Lachesis_group0__13_contigs__l.g7106	172.169	391.1411	-1.18441	0.003948	0.045559	1545	- && P0CO97.1 RecName: Full=SWR1-complex protein 4 >P0CO96.1 RecName: Full=SWR1-complex protein 4 && PF05499:DNA methyltransferase 1-associated protein 1 (DMAP1)|PF16282:SANT/Myb-like domain of DAMP1
Lachesis_group0__13_contigs__l.g5719	116.9881	266.3568	-1.18644	0.003975	0.045749	3681	- && Q09830.1 RecName: Full=TBC domain-containing protein C4G8.04 && PF00566:Rab-GTPase-TBC domain
Lachesis_group0__13_contigs__l.g3160	66.72776	152.4698	-1.19287	0.002493	0.034238	1506	- && Q8K2I1.1 RecName: Full=Protein farnesyltransferase subunit beta; Short=FTase-beta; AltName: Full=CAAX farnesyltransferase subunit beta; AltName: Full=Ras proteins prenyltransferase subunit beta && PF00432:Prenyltransferase and squalene oxidase repeat
Lachesis_group0__13_contigs__l.g4645	301.5815	690.4256	-1.19515	0.001554	0.026227	759	- && Q7S910.2 RecName: Full=54S ribosomal protein L4, mitochondrial; Flags: Precursor && PF06984:Mitochondrial 39-S ribosomal protein L47 (MRP-L47)
Lachesis_group0__13_contigs__l.g9445	246.8224	564.9775	-1.19528	0.003635	0.043709	1851	- && O94250.1 RecName: Full=Probable hydroxyacylglutathione hydrolase C13B11.03c; AltName: Full=Glyoxalase II; Short=Glx II && PF16123:Hydroxyacylglutathione hydrolase C-terminus

Lachesis_group0__13_contigs__l.g9026	572.9142	1315.021	-1.19888	0.00265	0.035564	3831	- && O74490.1 RecName: Full=Vacuolar protein sorting-associated protein ist1 && PF03398:Regulator of Vps4 activity in the MVB pathway|PF00566:Rab-GTPase-TBC domain
Lachesis_group0__13_contigs__l.g1282	993.3978	2282.227	-1.2002	0.001584	0.026539	4227	- && P13433.2 RecName: Full=DNA-directed RNA polymerase, mitochondrial; Flags: Precursor && PF00940:DNA-dependent RNA polymerase|PF14700:DNA-directed RNA polymerase N-terminal
Lachesis_group0__13_contigs__l.g2997	118.1703	271.8386	-1.20343	0.004038	0.046248	1302	- && A8NZM5.2 RecName: Full=WD repeat-containing protein JIP5 && -
Lachesis_group0__13_contigs__l.g1377	205.9724	475.0486	-1.20601	0.000364	0.01003	3651	- && A0A0D1E9B9.1 RecName: Full=Spore development regulator umv1 && PF11754:Velvet factor
Lachesis_group0__13_contigs__l.g3875	1425.666	3291.52	-1.20725	0.00217	0.031913	891	- && P38988.1 RecName: Full=Mitochondrial GTP/GDP carrier protein 1 && PF00153:Mitochondrial carrier protein
Lachesis_group0__13_contigs__l.g1322	904.8309	2089.218	-1.20745	0.003567	0.043293	1497	- && Q7ZXV8.1 RecName: Full=BUB3-interacting and GLEBS motif-containing protein ZNF207; Short=BuGZ; Short=xBuGZ; AltName: Full=Zinc finger protein 207 && -
Lachesis_group0__13_contigs__l.g2176	372.7847	864.7358	-1.21428	0.003242	0.04062	3801	- && O60177.1 RecName: Full=Uncharacterized ATP-dependent helicase C23E6.02 && PF00176:SNF2 family N-terminal domain|PF13639:Ring finger domain|PF00271:Helicase conserved C-terminal domain
Lachesis_group0__13_contigs__l.g2869	445.8647	1035.649	-1.21582	0.001234	0.023219	618	- && Q92429.1 RecName: Full=Superoxide dismutase [Mn], mitochondrial; AltName: Full=Mn-SOD; Flags: Precursor && PF02777:Iron/manganese superoxide

							dismutases, C-terminal domain|PF00081:Iron/manganese superoxide dismutases, alpha-hairpin domain
Lachesis_group0__13_contigs__l.g6921	541.6693	1258.702	-1.2168	0.001048	0.021054	1242	- && O60071.1 RecName: Full=Uncharacterized protein C13G1.09 && PF05291:Bystin
Lachesis_group0__13_contigs__l.g10377	1092.308	2547.235	-1.22176	0.002715	0.035948	1317	- && P17707.2 RecName: Full=S-adenosylmethionine decarboxylase proenzyme; Short=AdoMetDC; Short=SAMDC; Contains: RecName: Full=S-adenosylmethionine decarboxylase alpha chain; Contains: RecName: Full=S-adenosylmethionine decarboxylase beta chain; Flags: Precursor && PF01536:Adenosylmethionine decarboxylase
Lachesis_group0__13_contigs__l.g11045	452.2691	1056.411	-1.22418	0.001502	0.025804	3510	- && Q1MTQ9.1 RecName: Full=60S ribosome subunit biogenesis protein nip7 && PF08314:Secretory pathway protein Sec39|PF03657:Uncharacterised protein family (UPF0113)
Lachesis_group0__13_contigs__l.g7901	972.5975	2275.002	-1.22611	0.001461	0.025735	3480	- && Q14562.1 RecName: Full=ATP-dependent RNA helicase DHX8; AltName: Full=DEAH box protein 8; AltName: Full=RNA helicase HRH1 && PF00271:Helicase conserved C-terminal domain|PF07717:Oligonucleotide/oligosaccharide-binding (OB)-fold|PF00575:S1 RNA binding domain|PF04408:Helicase associated domain (HA2)
Lachesis_group0__13_contigs__l.g9187	533.5615	1250.685	-1.2294	0.00302	0.038649	528	- && Q759D1.1 RecName: Full=Ribosome biogenesis protein RLP24 && PF01246:Ribosomal protein L24e
Lachesis_group0__13_contigs__l.g235	193.0783	453.6566	-1.23279	0.003628	0.043684	2604	- && Q9DE25.1 RecName: Full=Forkhead box C1-A && PF00250:Forkhead domain

Lachesis_group0__13_contigs__l.g8476	347.4619	819.3393	-1.23805	0.001229	0.023165	975	- && Q08235.2 RecName: Full=Ribosome biogenesis protein BRX1 && PF04427:Brix domain
Lachesis_group0__13_contigs__l.g11087	155.727	367.5717	-1.23967	0.000898	0.018931	891	- && - && PF07890:Rrp15p
Lachesis_group0__13_contigs__l.g2737	142.6589	336.7526	-1.24	0.002637	0.035514	2064	- && A8NZY7.1 RecName: Full=tRNA-dihydrouridine(47) synthase [NAD(P)(+)]; AltName: Full=tRNA-dihydrouridine synthase 3 && PF01207:Dihydrouridine synthase (Dus)
Lachesis_group0__13_contigs__l.g4701	175.6991	415.1074	-1.2413	0.001146	0.022278	2019	- && P0CS09.1 RecName: Full=tRNA (adenine(58)-N(1))-methyltransferase catalytic subunit TRM61; AltName: Full=tRNA(m1A58)-methyltransferase subunit TRM61; Short=tRNA(m1A58)MTase subunit TRM61 >P0CS08.1 RecName: Full=tRNA (adenine(58)-N(1))-methyltransferase catalytic subunit TRM61; AltName: Full=tRNA(m1A58)-methyltransferase subunit TRM61; Short=tRNA(m1A58)MTase subunit TRM61 && PF08704:tRNA methyltransferase complex GCD14 subunit
Lachesis_group0__13_contigs__l.g1681	278.1704	658.9669	-1.24498	0.001359	0.02482	3210	- && O60113.1 RecName: Full=Uncharacterized amino-acid permease C15C4.04c && PF13520:Amino acid permease
Lachesis_group0__13_contigs__l.g6920	907.1086	2152.36	-1.24678	0.002606	0.035227	1299	- && Q6GMI9.2 RecName: Full=UDP-glucuronic acid decarboxylase 1; AltName: Full=UDP-glucuronate decarboxylase 1; Short=UXS-1 && PF16363:GDP-mannose 4,6 dehydratase
Lachesis_group0__13_contigs__l.g5547	3936.522	9367.418	-1.25077	0.003892	0.045343	1065	- && O14321.1 RecName: Full=Sterol 24-C-methyltransferase erg6; AltName: Full=Delta(24)-sterol C-methyltransferase erg6; AltName: Full=Ergosterol

							biosynthesis protein 6 && PF13847:Methyltransferase domain|PF08498:Sterol methyltransferase C-terminal
Lachesis_group0__13_contigs__l.g11400	709.3711	1690.375	-1.25299	0.001634	0.026859	1701	- && Q4P6N0.2 RecName: Full=ATP-dependent RNA helicase HAS1 && PF13959:Domain of unknown function (DUF4217)|PF00270:DEAD/DEAH box helicase|PF00271:Helicase conserved C-terminal domain
Lachesis_group0__13_contigs__l.g3987	3893.55	9292.095	-1.25494	0.002921	0.037746	987	- && - && PF07716:Basic region leucine zipper
Lachesis_group0__13_contigs__l.g6296	247.8179	592.1536	-1.25735	0.002408	0.033962	2349	- && P0CM79.1 RecName: Full=Polynucleotide 5'-hydroxyl-kinase GRC3 >P0CM78.1 RecName: Full=Polynucleotide 5'-hydroxyl-kinase GRC3 && PF16575:mRNA cleavage and polyadenylation factor CLP1 P-loop
Lachesis_group0__13_contigs__l.g997	307.4788	737.9382	-1.26357	0.002439	0.033962	1095	- && O14180.1 RecName: Full=Brix domain-containing protein C4F8.04 && PF04427:Brix domain
Lachesis_group0__13_contigs__l.g8964	77.59577	186.4058	-1.26412	0.001132	0.022054	3264	- && P0CO92.1 RecName: Full=DNA mismatch repair protein MSH3; AltName: Full=MutS protein homolog 3 && PF01624:MutS domain I|PF05188:MutS domain II|PF00488:MutS domain V|PF05192:MutS domain III
Lachesis_group0__13_contigs__l.g4665	147.7199	355.3733	-1.26713	0.001101	0.021674	5991	- && Q92355.1 RecName: Full=Helicase sen1 && PF12726:SEN1 N terminal|PF13087:AAA domain|PF13086:AAA domain
Lachesis_group0__13_contigs__l.g9391	100.8717	242.7857	-1.26816	0.002988	0.038349	1275	- && P0CO07.1 RecName: Full=Histone acetyltransferase type B catalytic subunit >P0CO06.1 RecName: Full=Histone acetyltransferase type B catalytic subunit && PF10394:Histone acetyl transferase HAT1 N-terminus

Lachesis_group0__13_contigs__l.g9474	170.6404	411.8743	-1.27149	0.000271	0.008306	1383	- && B5XF11.1 RecName: Full=Lysine-specific demethylase 8; AltName: Full=JmjC domain-containing protein 5; AltName: Full=Jumonji domain-containing protein 5 [Salmo salar] && PF13621:Cupin-like domain
Lachesis_group0__13_contigs__l.g9043	340.4652	822.6552	-1.27324	0.000763	0.016781	1659	- && Q9UTB4.1 RecName: Full=Protein mss51 && PF13824:Zinc-finger of mitochondrial splicing suppressor 51
Lachesis_group0__13_contigs__l.g2918	413.8431	1001.133	-1.27488	0.003949	0.045559	3672	- && P22579.2 RecName: Full=Transcriptional regulatory protein SIN3 && PF16879:C-terminal domain of Sin3a protein|PF08295:Sin3 family co-repressor|PF02671:Paired amphipathic helix repeat
Lachesis_group0__13_contigs__l.g11334	1216.767	2944.357	-1.27507	0.0012	0.0229	1569	- && O14007.1 RecName: Full=H/ACA ribonucleoprotein complex subunit 4; AltName: Full=Centromere-binding factor 5 homolog && PF08068:DKCLD (NUC011) domain|PF01472:PUA domain|PF16198:tRNA pseudouridylate synthase B C-terminal domain|PF01509:TruB family pseudouridylate synthase (N terminal domain)
Lachesis_group0__13_contigs__l.g5723	1121.585	2716.853	-1.27644	0.0039	0.045389	1374	- && B3EAE0.1 RecName: Full=8-amino-7-oxononanoate synthase; Short=AONS; AltName: Full=7-keto-8-amino-pelargonic acid synthase; Short=7-KAP synthase; Short=KAPA synthase; AltName: Full=8-amino-7-ketopelargonate synthase && PF00155:Aminotransferase class I and II
Lachesis_group0__13_contigs__l.g5996	527.8261	1278.571	-1.27672	0.000835	0.017944	1068	- && O74978.1 RecName: Full=Uncharacterized RNA-binding protein C1827.05c && PF00076:RNA recognition motif. (a.k.a. RRM, RBD, or RNP domain)

Lachesis_group0__13_contigs__l.g3724	301.866	731.5035	-1.27727	0.001162	0.022395	1962	- && - && PF03235:Protein of unknown function DUF262
Lachesis_group0__13_contigs__l.g11390	1707.654	4139.538	-1.27759	0.002071	0.031048	2985	- && O74359.1 RecName: Full=Meiotically up-regulated gene 60 protein && PF00013:KH domain
Lachesis_group0__13_contigs__l.g11392	186.0212	451.392	-1.2794	0.002639	0.035514	1413	- && Q9SKC4.1 RecName: Full=Probable E3 ubiquitin-protein ligase ARI10; AltName: Full=ARIADNE-like protein ARI10; AltName: Full=Protein ariadne homolog 10; AltName: Full=RING-type E3 ubiquitin transferase ARI10 && PF05773:RWD domain|PF01485:IBR domain, a half RING-finger domain
Lachesis_group0__13_contigs__l.g9238	279.2876	680.1067	-1.28468	0.00271	0.035948	1980	- && A8NVW1.2 RecName: Full=Mitochondrial outer membrane protein IML2 && PF10300:Protein of unknown function (DUF3808)
Lachesis_group0__13_contigs__l.g5168	70.14058	171.6198	-1.29051	0.00368	0.043921	1347	- && P20397.3 RecName: Full=Nucleolin; AltName: Full=Protein C23 && PF14259:RNA recognition motif (a.k.a. RRM, RBD, or RNP domain)|PF00076:RNA recognition motif. (a.k.a. RRM, RBD, or RNP domain)
Lachesis_group0__13_contigs__l.g133	658.0892	1615.441	-1.29568	7.90E-05	0.003444	417	- && P0CO05.1 RecName: Full=Histone H3 >P0CO04.1 RecName: Full=Histone H3 && PF00125:Core histone H2A/H2B/H3/H4
Lachesis_group0__13_contigs__l.g4056	547.5354	1346.458	-1.29855	0.001444	0.025578	939	- && - && PF08790:LYAR-type C2HC zinc finger
Lachesis_group0__13_contigs__l.g11453	1031.061	2541.412	-1.30167	0.001806	0.028487	876	- && Q55ED1.1 RecName: Full=Probable nucleosome assembly protein && PF00956:Nucleosome assembly protein (NAP)
Lachesis_group0__13_contigs__l.g11420	329.5307	812.9292	-1.30316	0.002107	0.031301	681	- && Q5RKQ0.1 RecName: Full=Pre-mRNA-splicing factor SPF27; AltName: Full=Protein BCAS2 homolog && PF05700:Breast carcinoma amplified sequence 2 (BCAS2)

Lachesis_group0__13_contigs__l.g8819	1592.96	3934.566	-1.30459	0.001426	0.025394	1038	- && A1D3T4.1 RecName: Full=Probable arabinogalactan endo-beta-1,4-galactanase A; AltName: Full=Endo-1,4-beta-galactanase A; Short=Galactanase A; Flags: Precursor && PF07745:Glycosyl hydrolase family 53
Lachesis_group0__13_contigs__l.g7405	1877.356	4645.917	-1.30736	0.000759	0.016774	1146	- && P0CP98.1 RecName: Full=FK506-binding protein 4; AltName: Full=Histone proline isomerase; AltName: Full=Peptidyl-prolyl cis-trans isomerase; Short=PPIase; AltName: Full=Rotamase >P0CP99.1 RecName: Full=FK506-binding protein 4; AltName: Full=Histone proline isomerase; AltName: Full=Peptidyl-prolyl cis-trans isomerase; Short=PPIase; AltName: Full=Rotamase && PF00254:FKBP-type peptidyl-prolyl cis-trans isomerase
Lachesis_group0__13_contigs__l.g7781	186.4394	461.1871	-1.30759	0.002514	0.034426	963	- && Q14684.3 RecName: Full=Ribosomal RNA processing protein 1 homolog B; AltName: Full=RRP1-like protein B && PF05997:Nucleolar protein,Nop52
Lachesis_group0__13_contigs__l.g10494	115.1667	285.1505	-1.30914	0.003476	0.042856	4080	- && - && PF12738:twin BRCT domain
Lachesis_group0__13_contigs__l.g4034	267.6011	663.2692	-1.31011	0.00248	0.03423	1011	- && Q10248.2 RecName: Full=Uncharacterized mitochondrial carrier C4G9.20c && PF00153:Mitochondrial carrier protein
Lachesis_group0__13_contigs__l.g11194	386.9759	959.3554	-1.3104	0.001252	0.023417	2298	- && O13910.1 RecName: Full=U3 small nucleolar ribonucleoprotein protein mpp10 && PF04006:Mpp10 protein
Lachesis_group0__13_contigs__l.g1295	74.70645	185.5743	-1.31282	0.004492	0.049923	1803	- && Q6NRG5.1 RecName: Full=NADPH-dependent diflavin oxidoreductase 1; AltName: Full=NADPH-dependent FMN and FAD-containing oxidoreductase && PF00667:FAD binding

								domain|PF00258:Flavodoxin|PF00175:Oxidoreductase NAD-binding domain
Lachesis_group0__13_contigs__l.g9167	260.3707	647.8844	-1.31594		0.003889	0.045343	732	- && Q9CY21.1 RecName: Full=Probable 18S rRNA (guanine-N(7))-methyltransferase; AltName: Full=Bud site selection protein 23 homolog; AltName: Full=Williams-Beuren syndrome chromosomal region 22 protein homolog && PF12589:Methyltransferase involved in Williams-Beuren syndrome
Lachesis_group0__13_contigs__l.g2345	83.22058	207.3942	-1.31751		0.002338	0.033285	2796	- && O13396.1 RecName: Full=DNA mismatch repair protein msh-2 && PF01624:MutS domain I|PF05190:MutS family domain IV|PF00488:MutS domain V|PF05192:MutS domain III|PF05188:MutS domain II
Lachesis_group0__13_contigs__l.g5783	251.4212	626.9854	-1.31865		0.001071	0.021345	717	- && P35521.1 RecName: Full=Methylosome subunit pICln; AltName: Full=Chloride channel, nucleotide sensitive 1A; AltName: Full=Chloride conductance regulatory protein ICln; Short=I(Cln) && PF03517:Regulator of volume decrease after cellular swelling
Lachesis_group0__13_contigs__l.g9364	150.7638	376.2297	-1.31952		0.002422	0.033962	1035	- && Q10480.2 RecName: Full=Nuclease 1, mitochondrial; AltName: Full=SpNUC1; Flags: Precursor && PF01223:DNA/RNA non-specific endonuclease
Lachesis_group0__13_contigs__l.g1360	1512.369	3778.617	-1.32112		0.000399	0.010614	1701	- && P87058.1 RecName: Full=La protein homolog; AltName: Full=La autoantigen homolog; AltName: Full=La ribonucleoprotein && PF05383:La domain|PF00076:RNA recognition motif. (a.k.a. RRM, RBD, or RNP domain)

Lachesis_group0__13_contigs__l.g978	204.7969	512.5428	-1.32381	0.00029	0.008702	948	- && Q9UTE6.1 RecName: Full=Protein mak16 && PF01778:Ribosomal L28e protein family|PF04874:Mak16 protein C-terminal region
Lachesis_group0__13_contigs__l.g5246	105.6459	264.2306	-1.32384	0.00211	0.031301	711	- && P54168.1 RecName: Full=Uncharacterized protein YpgQ && -
Lachesis_group0__13_contigs__l.g10437	161.7573	404.7992	-1.32391	0.000556	0.013256	1173	- && - && PF00398:Ribosomal RNA adenine dimethylase
Lachesis_group0__13_contigs__l.g10133	211.2036	529.8383	-1.32709	0.00098	0.020086	960	- && Q09704.1 RecName: Full=Exosome complex component rrp4; AltName: Full=Ribosomal RNA-processing protein 4 && PF14382:Exosome complex exonuclease RRP4 N-terminal region|PF15985:KH domain
Lachesis_group0__13_contigs__l.g10764	711.5621	1785.195	-1.3271	0.003944	0.045559	3171	- && Q09833.1 RecName: Full=tRNA (uracil(54)-C(5))-methyltransferase && PF05958:tRNA (Uracil-5-)-methyltransferase
Lachesis_group0__13_contigs__l.g5743	55.4072	139.2412	-1.33028	0.002033	0.030999	1011	- && - && PF04005:Hus1-like protein
Lachesis_group0__13_contigs__l.g3894	3404.946	8620.379	-1.34018	0.001452	0.025672	2352	- && O59838.2 RecName: Full=Heat shock protein homolog pss1 && PF00012:Hsp70 protein|PF10712:NAD-specific glutamate dehydrogenase
Lachesis_group0__13_contigs__l.g1354	40.11223	101.5561	-1.34101	0.002626	0.035444	1632	- && P39932.2 RecName: Full=Sugar transporter STL1 && PF00083:Sugar (and other) transporter
Lachesis_group0__13_contigs__l.g4727	275.3781	698.4954	-1.34322	0.00027	0.008283	2781	- && O74832.1 RecName: Full=mRNA degradation protein pet127, mitochondrial; Flags: Precursor && PF08634:Mitochondrial protein Pet127
Lachesis_group0__13_contigs__l.g1294	526.0473	1335.313	-1.34412	0.001507	0.025804	2220	- && Q9UUD0.1 RecName: Full=Uncharacterized protein C19C2.10 && PF03114:BAR domain|PF14604:Variant SH3 domain

Lachesis_group0__13_contigs__l.g7787	2116.01	5384.351	-1.34745	0.000529	0.012805	312	- && P62792.2 RecName: Full=Histone H4 >P62793.2 RecName: Full=Histone H4 && PF15511:Centromere kinetochore component CENP-T histone fold
Lachesis_group0__13_contigs__l.g1461	1365.531	3484.348	-1.35149	0.001606	0.026728	609	- && - && PF10342:Ser-Thr-rich glycosyl-phosphatidyl-inositol-anchored membrane family
Lachesis_group0__13_contigs__l.g11275	510.0623	1302.466	-1.35292	0.003731	0.04416	1038	- && Q6DHC3.1 RecName: Full=Solute carrier family 25 member 40 && PF00153:Mitochondrial carrier protein
Lachesis_group0__13_contigs__l.g303	59.4406	151.7523	-1.35305	0.003164	0.039942	1896	- && Q10181.1 RecName: Full=Uncharacterized protein C3F10.06c && PF04179:Initiator tRNA phosphoribosyl transferase
Lachesis_group0__13_contigs__l.g8445	192.168	491.2365	-1.35436	0.001675	0.027257	1857	- && - && PF12937:F-box-like
Lachesis_group0__13_contigs__l.g9423	54.7702	140.1374	-1.35525	0.001478	0.025764	1491	- && - && PF07690:Major Facilitator Superfamily
Lachesis_group0__13_contigs__l.g6085	268.504	688.0754	-1.35759	0.000198	0.006685	2295	- && Q4V8D1.1 RecName: Full=Cytochrome P450 2U1 && PF00106:short chain dehydrogenase|PF00067:Cytochrome P450
Lachesis_group0__13_contigs__l.g11040	741.3897	1901.146	-1.35861	0.002061	0.031035	2682	- && Q9P7G8.1 RecName: Full=UPF0382 membrane protein C1782.12c; Flags: Precursor && PF04241:Protein of unknown function (DUF423)
Lachesis_group0__13_contigs__l.g10881	322.1599	826.7771	-1.36008	0.000536	0.012899	1215	- && Q9HE11.2 RecName: Full=U3 small nucleolar RNA-associated protein 5; Short=U3 snoRNA-associated protein 5; AltName: Full=U3 protein 5 required for transcription && PF04003:Dip2/Utp12 Family
Lachesis_group0__13_contigs__l.g6402	942.8312	2430.901	-1.36654	0.002194	0.032118	4827	- && Q09878.2 RecName: Full=Probable sulfite reductase [NADPH] flavoprotein component && PF00175:Oxidoreductase NAD-binding

							domain|PF00667:FAD binding domain|PF00759:Glycosyl hydrolase family 9
Lachesis_group0__13_contigs__l.g11297	588.2689	1516.803	-1.36679	0.002103	0.031301	729	- && Q4P4X6.2 RecName: Full=Eukaryotic translation initiation factor 3 subunit K; Short=eIF3k; AltName: Full=eIF-3 p25 && PF10075:CSN8/PSMD8/EIF3K family
Lachesis_group0__13_contigs__l.g2767	765.3907	1974.817	-1.36764	0.000142	0.005294	1815	- && Q9P7C0.1 RecName: Full=Uncharacterized WD repeat-containing protein C2E1P5.05 && -
Lachesis_group0__13_contigs__l.g4177	106.4367	274.334	-1.36767	0.002456	0.034085	1722	- && B7F9G7.1 RecName: Full=Heptahelical transmembrane protein ADIPOR1; AltName: Full=PAQR family protein ADIPOR1 && PF03006:Haemolysin-III related
Lachesis_group0__13_contigs__l.g3351	180.3852	465.5031	-1.36849	0.003197	0.040242	1011	- && Q10205.1 RecName: Full=Exosome complex component rrp43; AltName: Full=Ribosomal RNA-processing protein 43 && PF01138:3' exoribonuclease family, domain 1
Lachesis_group0__13_contigs__l.g5906	497.7522	1285.439	-1.369	0.002323	0.033178	834	- && - && PF05817:Oligosaccharyltransferase subunit Ribophorin II
Lachesis_group0__13_contigs__l.g1587	228.0273	589.268	-1.36953	0.002415	0.033962	3087	- && B0D4E6.1 RecName: Full=Phosphatidylethanolamine N-methyltransferase; Short=PE methyltransferase; Short=PEAMT; Short=PEMT && PF04191:Phospholipid methyltransferase
Lachesis_group0__13_contigs__l.g5292	306.2436	792.0214	-1.37116	0.002587	0.035108	1389	- && Q6NUA1.1 RecName: Full=Protein arginine N-methyltransferase 5; AltName: Full=Histone synthetic lethal 7 protein; Short=Hsl7; AltName: Full=Histone-arginine N-methyltransferase PRMT5 && PF05185:PRMT5 arginine-N-methyltransferase

Lachesis_group0__13_contigs__l.g2646	425.9187	1101.933	-1.37153	0.001206	0.022921	2985	- && O13671.2 RecName: Full=Importin-alpha re-exporter; AltName: Full=Cellular apoptosis susceptibility protein homolog && PF03378:CAS/CSE protein, C-terminus|PF08506:Cse1|PF03810:Importin-beta N-terminal domain
Lachesis_group0__13_contigs__l.g10512	3013.304	7798.628	-1.37192	0.000469	0.011803	3609	- && Q11119.2 RecName: Full=Ubiquitin carboxyl-terminal hydrolase 14; AltName: Full=Deubiquitinating enzyme 14; AltName: Full=UBA domain-containing protein 2; AltName: Full=Ubiquitin thioesterase 14; AltName: Full=Ubiquitin-specific-processing protease 14 && PF00956:Nucleosome assembly protein (NAP)|PF00627:UBA/TS-N domain|PF02148:Zn-finger in ubiquitin-hydrolases and other protein|PF00443:Ubiquitin carboxyl-terminal hydrolase
Lachesis_group0__13_contigs__l.g6316	179.4853	464.7692	-1.37288	0.000446	0.011385	3777	- && Q6L4S0.1 RecName: Full=DNA damage-binding protein 1; AltName: Full=UV-damaged DNA-binding protein 1; Short=OsUV-DDB1 && PF10433:Mono-functional DNA-alkylating methyl methanesulfonate N-term|PF03178:CPSF A subunit region
Lachesis_group0__13_contigs__l.g11397	242.3723	631.7441	-1.38262	0.002699	0.035948	300	- && P0CR99.1 RecName: Full=Mitochondrial import inner membrane translocase subunit TIM10 >P0CR98.1 RecName: Full=Mitochondrial import inner membrane translocase subunit TIM10 && PF02953:Tim10/DDP family zinc finger
Lachesis_group0__13_contigs__l.g6493	667.9087	1742.756	-1.3837	0.002442	0.033962	555	- && Q03020.1 RecName: Full=Iron sulfur cluster assembly protein 1, mitochondrial; AltName: Full=Iron

							sulfur cluster scaffold protein 1; Flags: Precursor && PF01592:NifU-like N terminal domain
Lachesis_group0__13_contigs__l.g5532	565.816	1478.445	-1.38595	0.001054	0.021054	2313	- && P0CQ91.1 RecName: Full=ATP-dependent RNA helicase MAK5 && PF00270:DEAD/DEAH box helicase|PF00271:Helicase conserved C-terminal domain
Lachesis_group0__13_contigs__l.g8180	218.0733	570.3042	-1.38757	0.001817	0.028603	1734	- && O43011.2 RecName: Full=Histidine--tRNA ligase, mitochondrial; AltName: Full=Histidyl-tRNA synthetase; Short=HisRS; Flags: Precursor && PF03129:Anticodon binding domain|PF13393:Histidyl-tRNA synthetase
Lachesis_group0__13_contigs__l.g4249	92.03268	241.3963	-1.39178	0.000888	0.018826	3876	- && - && PF13532:2OG-Fe(II) oxygenase superfamily
Lachesis_group0__13_contigs__l.g9133	30.50182	79.9657	-1.39186	0.003523	0.043082	2307	- && O60174.1 RecName: Full=Transcription factor tau subunit sfc6; AltName: Full=TFIIIC subunit sfc6; AltName: Full=Transcription factor C subunit 6 && -
Lachesis_group0__13_contigs__l.g9430	373.5233	979.9387	-1.39186	0.000295	0.008814	1398	- && O14206.2 RecName: Full=Brix domain-containing protein C1B9.03c && PF04427:Brix domain
Lachesis_group0__13_contigs__l.g139	113.4424	297.6513	-1.39268	0.00259	0.035108	588	- && Q63042.2 RecName: Full=FAD-linked sulfhydryl oxidase ALR; AltName: Full=Augmenter of liver regeneration && PF04777:Erv1 / Alr family
Lachesis_group0__13_contigs__l.g1807	50.19611	131.6278	-1.39336	0.001386	0.025024	1380	- && Q9Y8H5.2 RecName: Full=Delta(12) fatty acid desaturase; AltName: Full=Delta-12 fatty acid desaturase && PF00487:Fatty acid desaturase
Lachesis_group0__13_contigs__l.g6775	279.4959	735.6348	-1.39667	0.001436	0.025471	606	- && O14225.1 RecName: Full=Mitochondrial import receptor subunit tom20; AltName: Full=Mitochondrial 20 kDa outer membrane protein; AltName: Full=Translocase of outer membrane 20 kDa subunit && PF02064:MAS20 protein import receptor

Lachesis_group0__13_contigs__l.g2141	307.7372	811.4327	-1.39925	0.000418	0.011018	1203	- && - && PF15459:60S ribosome biogenesis protein Rrp14|PF04935:Surfeit locus protein 6
Lachesis_group0__13_contigs__l.g6964	222.9506	589.08	-1.40191	0.002053	0.031035	2184	- && Q9P7H1.1 RecName: Full=Tyrosine-protein phosphatase CDC14 homolog; AltName: Full=CDC fourteen-like phosphatase 1 && PF00782:Dual specificity phosphatase, catalytic domain|PF14671:Dual specificity protein phosphatase, N-terminal half
Lachesis_group0__13_contigs__l.g10983	80.95631	213.6264	-1.40223	0.0031	0.039297	1242	- && - && PF10453:Nuclear fragile X mental retardation-interacting protein 1 (NUFIP1)
Lachesis_group0__13_contigs__l.g10991	101.5206	269.9763	-1.4121	0.000853	0.018234	2340	- && A3M0B1.2 RecName: Full=Pre-rRNA-processing protein IPI1 && PF12333:Rix1 complex component involved in 60S ribosome maturation
Lachesis_group0__13_contigs__l.g2649	173.9128	462.9922	-1.41304	0.002228	0.032381	1830	- && O14059.1 RecName: Full=Probable histidinol-phosphatase; Short=HolPase && PF00111:2Fe-2S iron-sulfur cluster binding domain|PF02811:PHP domain
Lachesis_group0__13_contigs__l.g5539	181.7608	483.8561	-1.41337	0.001167	0.022443	594	- && Q4X0T4.1 RecName: Full=Required for respiratory growth protein 9, mitochondrial; Flags: Precursor >B0XSW5.1 RecName: Full=Required for respiratory growth protein 9, mitochondrial; Flags: Precursor && PF06413:Neugrin
Lachesis_group0__13_contigs__l.g10875	291.113	777.0278	-1.41712	0.000885	0.018789	1773	- && Q75AE1.1 RecName: Full=ATP-dependent RNA helicase ROK1 && PF00270:DEAD/DEAH box helicase|PF00271:Helicase conserved C-terminal domain
Lachesis_group0__13_contigs__l.g3848	337.5789	902.6977	-1.41938	0.000378	0.010208	3603	- && - && PF12937:F-box-like

Lachesis_group0__13_contigs__l.g9116	49.26561	132.172	-1.42586	0.001697	0.027469	936	- && Q2KIB9.1 RecName: Full=Ribonuclease P protein subunit p29 && PF01868:Domain of unknown function UPF0086
Lachesis_group0__13_contigs__l.g11445	471.8253	1267.918	-1.42642	0.000355	0.009881	1398	- && Q10276.1 RecName: Full=Putative choline kinase && PF01633:Choline/ethanolamine kinase
Lachesis_group0__13_contigs__l.g3244	53.36474	143.1858	-1.42706	0.002422	0.033962	1602	- && - && PF04305:Protein of unknown function (DUF455)|PF00355:Rieske [2Fe-2S] domain
Lachesis_group0__13_contigs__l.g9427	65.24912	175.1226	-1.42717	0.002405	0.033962	1128	- && O94684.1 RecName: Full=RNA polymerase II transcription factor B subunit 3; AltName: Full=CDK-activating kinase assembly factor MAT1 homolog; AltName: Full=RING finger protein pmh1; AltName: Full=RNA polymerase II transcription factor B 38 kDa subunit; AltName: Full=RNA polymerase II transcription factor B p38 subunit && PF06391:CDK-activating kinase assembly factor MAT1|PF17121:Zinc finger, C3HC4 type (RING finger)
Lachesis_group0__13_contigs__l.g249	826.9805	2225.163	-1.42816	0.000864	0.018442	669	- && O13917.1 RecName: Full=Hypoxanthine-guanine phosphoribosyltransferase; Short=HGPRT; Short=HGPRTase && -
Lachesis_group0__13_contigs__l.g460	468.9561	1264.967	-1.43121	0.000402	0.010663	2217	- && P38776.1 RecName: Full=Probable drug/proton antiporter YHK8 && PF07690:Major Facilitator Superfamily
Lachesis_group0__13_contigs__l.g442	600.1384	1621.839	-1.43461	0.000222	0.007243	1125	- && P46794.2 RecName: Full=Cystathionine beta-synthase; AltName: Full=Beta-thionase; AltName: Full=Serine sulfhydrase && PF00291:Pyridoxal-phosphate dependent enzyme
Lachesis_group0__13_contigs__l.g11301	699.2073	1895.037	-1.43869	0.000224	0.007293	1869	- && A8N1X3.1 RecName: Full=Pescadillo homolog; AltName: Full=Nucleolar protein 7 homolog &&

							PF16589:BRCT domain, a BRCA1 C-terminus domain|PF06732:Pescadillo N-terminus
Lachesis_group0__13_contigs__l.g11128	636.778	1727.496	-1.44016	0.000446	0.011385	4425	- && O74835.1 RecName: Full=rRNA biogenesis protein rrp5; AltName: Full=Ribosomal RNA-processing protein 5; AltName: Full=U3 small nucleolar RNA-associated protein rrp5; Short=U3 snoRNA-associated protein rrp5 && PF00575:S1 RNA binding domain
Lachesis_group0__13_contigs__l.g1957	515.8708	1400.982	-1.44165	0.000465	0.011739	1656	- && Q9USK3.1 RecName: Full=Uncharacterized transporter C4B3.13 && PF01554:MatE
Lachesis_group0__13_contigs__l.g6210	44.69751	121.5604	-1.44539	0.001394	0.02507	1011	- && P0CR37.1 RecName: Full=3-ketodihydrosphingosine reductase TSC10; AltName: Full=3-dehydrosphinganine reductase; AltName: Full=KDS reductase && PF00106:short chain dehydrogenase
Lachesis_group0__13_contigs__l.g6547	37.99883	103.8276	-1.45068	0.003485	0.042856	1080	- && - && PF00011:Hsp20/alpha crystallin family
Lachesis_group0__13_contigs__l.g6656	71.98291	196.6493	-1.45205	0.001903	0.029557	1707	- && P0CN19.1 RecName: Full=Diphthamide biosynthesis protein 1 >P0CN18.1 RecName: Full=Diphthamide biosynthesis protein 1 && PF01866:Putative diphthamide synthesis protein
Lachesis_group0__13_contigs__l.g9977	313.9699	859.6152	-1.45324	0.000921	0.019172	1269	- && - && PF07766:LETM1-like protein
Lachesis_group0__13_contigs__l.g9910	1621.297	4444.329	-1.4549	7.68E-05	0.003409	1863	- && - && PF04577:Protein of unknown function (DUF563)
Lachesis_group0__13_contigs__l.g6429	4857.879	13330.07	-1.45629	0.000634	0.014659	1953	- && O59714.1 RecName: Full=Uncharacterized membrane protein C3B8.06 && PF10348:Domain of unknown function (DUF2427)|PF10355:Protein of unknown function (Ytp1)

Lachesis_group0__13_contigs__l.g2025	36.04074	98.71019	-1.45724	0.001987	0.030369	1167	- && Q9LVH5.1 RecName: Full=Outer envelope protein 64, chloroplastic; AltName: Full=Translocon at the outer membrane of chloroplasts 64-III && PF13414:TPR repeat|PF07719:Tetratricopeptide repeat|PF13877:Potential Monad-binding region of RPAP3
Lachesis_group0__13_contigs__l.g6146	2421.466	6650.237	-1.45758	0.000827	0.017849	1731	- && P38861.3 RecName: Full=60S ribosomal export protein NMD3; AltName: Full=Nonsense-mediated mRNA decay protein 3 && PF04981:NMD3 family
Lachesis_group0__13_contigs__l.g5936	1127.696	3097.228	-1.45764	0.000716	0.016088	3354	- && P0CQ17.1 RecName: Full=Lon protease homolog, mitochondrial; Flags: Precursor && PF05362:Lon protease (S16) C-terminal proteolytic domain|PF02190:ATP-dependent protease La (LON) substrate-binding domain|PF00004:ATPase family associated with various cellular activities (AAA)
Lachesis_group0__13_contigs__l.g2580	24.46254	67.29542	-1.45894	0.001771	0.028174	4932	- && - && PF00400:WD domain, G-beta repeat|PF05729:NACHT domain
Lachesis_group0__13_contigs__l.g8600	23.0765	63.45526	-1.46163	0.004322	0.04872	1770	- && - && PF02469:Fasciclin domain
Lachesis_group0__13_contigs__l.g4298	885.9537	2450.505	-1.46775	0.00071	0.01606	1020	- && P0CN61.1 RecName: Full=Probable electron transfer flavoprotein subunit alpha, mitochondrial; Short=Alpha-ETF; Flags: Precursor >P0CN60.1 RecName: Full=Probable electron transfer flavoprotein subunit alpha, mitochondrial; Short=Alpha-ETF; Flags: Precursor && PF00766:Electron transfer flavoprotein FAD-binding domain|PF01012:Electron transfer flavoprotein domain

Lachesis_group0__13_contigs__l.g6885	369.183	1022.969	-1.47056	0.000209	0.006998	1770	- && P40186.3 RecName: Full=PHO85 cyclin-7; AltName: Full=PHO85-associated protein 1 && PF08613:Cyclin
Lachesis_group0__13_contigs__l.g4172	94.77414	262.6174	-1.47105	0.001132	0.022054	1209	- && Q8XJL8.1 RecName: Full=Probable serine/threonine-protein kinase CPE1738 && PF00069:Protein kinase domain
Lachesis_group0__13_contigs__l.g409	13158.61	36501.38	-1.47195	0.000167	0.00601	1188	- && - && PF07992:Pyridine nucleotide-disulphide oxidoreductase
Lachesis_group0__13_contigs__l.g7775	440.8238	1222.845	-1.47244	0.000512	0.012604	1590	- && Q1JQD2.1 RecName: Full=Glutamate-rich WD repeat-containing protein 1 && PF12265:Histone-binding protein RBBP4 or subunit C of CAF1 complex
Lachesis_group0__13_contigs__l.g3417	78.9945	219.3926	-1.47351	0.001508	0.025804	630	- && - && PF08641:Kinetochore protein Mis14 like
Lachesis_group0__13_contigs__l.g10620	157.0653	436.4349	-1.47431	0.00348	0.042856	4590	- && P08096.2 RecName: Full=DNA topoisomerase 2; AltName: Full=DNA topoisomerase II && PF00204:DNA gyrase B|PF00521:DNA gyrase/topoisomerase IV, subunit A|PF16898:C-terminal associated domain of TOPRIM|PF02518:Histidine kinase-, DNA gyrase B-, and HSP90-like ATPase|PF01751:Toprim domain
Lachesis_group0__13_contigs__l.g365	220.0329	611.4096	-1.47543	0.00083	0.017879	1506	- && Q6TNS2.1 RecName: Full=p21-activated protein kinase-interacting protein 1-like; AltName: Full=PAK1-interacting protein 1-like && PF00400:WD domain, G-beta repeat
Lachesis_group0__13_contigs__l.g5873	108.8421	302.5128	-1.47616	0.000572	0.013558	474	- && P0CR33.1 RecName: Full=Succinate dehydrogenase assembly factor 2, mitochondrial; Short=SDH assembly factor 2; Short=SDHAF2; Flags: Precursor >P0CR32.1 RecName: Full=Succinate dehydrogenase assembly factor 2, mitochondrial;

							Short=SDH assembly factor 2; Short=SDHAF2; Flags: Precursor && PF03937:Flavinator of succinate dehydrogenase
Lachesis_group0__13_contigs__l.g9241	162.7886	452.8794	-1.47637	0.000997	0.020307	2349	- && O75001.1 RecName: Full=DNA replication licensing factor mcm7; AltName: Full=Minichromosome maintenance protein 7 && PF00493:MCM2/3/5 family|PF14551:MCM N-terminal domain
Lachesis_group0__13_contigs__l.g8240	229.1445	638.6097	-1.47908	0.000305	0.00897	1209	- && - && PF00046:Homeobox domain
Lachesis_group0__13_contigs__l.g6908	276.6215	772.0361	-1.4814	0.001751	0.027915	1035	- && - && PF02777:Iron/manganese superoxide dismutases, C-terminal domain
Lachesis_group0__13_contigs__l.g1479	75.62574	211.088	-1.48154	0.002291	0.03288	1263	- && O94725.1 RecName: Full=Protein-glutamate O-methyltransferase C1393.13 && PF01937:Protein of unknown function DUF89
Lachesis_group0__13_contigs__l.g1025	341.2314	953.8015	-1.48292	0.002173	0.031913	6777	- && Q7S1P9.3 RecName: Full=DNA repair protein rad5; AltName: Full=Mutagen-sensitive protein 41 && PF00271:Helicase conserved C-terminal domain|PF00145:C-5 cytosine-specific DNA methylase|PF00176:SNF2 family N-terminal domain
Lachesis_group0__13_contigs__l.g5715	10625.78	29718.91	-1.48383	0.004156	0.047417	3639	- && Q96UH7.3 RecName: Full=Fructose-bisphosphate aldolase 1; Short=FBP aldolase 1; Short=FBPA 1; AltName: Full=Fructose-1,6-bisphosphate aldolase 1 && PF03069:Acetamidase/Formamidase family|PF01116:Fructose-bisphosphate aldolase class-II|PF03062:MBOAT, membrane-bound O-acyltransferase family
Lachesis_group0__13_contigs__l.g129	115.6536	323.3367	-1.48427	0.000304	0.008944	1779	- && P0CS53.1 RecName: Full=tRNA (guanine-N(7)-)-methyltransferase non-catalytic subunit TRM82;

							AltName: Full=Transfer RNA methyltransferase 82 && -
Lachesis_group0__13_contigs__l.g9051	1148.925	3216.262	-1.48514	6.31E-05	0.003042	6465	- && Q9C102.1 RecName: Full=Putative glutamate synthase [NADPH]; AltName: Full=NADPH-GOGAT && PF04898:Glutamate synthase central domain|PF00310:Glutamine amidotransferases class-II|PF01493:GXGXG motif|PF14691:Dihydroprymidine dehydrogenase domain II, 4Fe-4S cluster|PF01645:Conserved region in glutamate synthase|PF07992:Pyridine nucleotide-disulphide oxidoreductase
Lachesis_group0__13_contigs__l.g740	408.2632	1146.353	-1.48961	0.001479	0.025764	2013	- && Q00614.1 RecName: Full=Carnitine O-acetyltransferase, mitochondrial; Short=Carnitine acetylase; Flags: Precursor && PF00755:Choline/Carnitine o-acyltransferase
Lachesis_group0__13_contigs__l.g375	341.389	962.3651	-1.49566	0.000197	0.006685	1593	- && Q7ZXK9.1 RecName: Full=Notchless protein homolog 1 && PF08154:NLE (NUC135) domain|PF00400:WD domain, G-beta repeat
Lachesis_group0__13_contigs__l.g4481	115.5401	325.9633	-1.49643	0.000555	0.013256	1329	- && Q9HDX2.1 RecName: Full=Uncharacterized lactate 2-monooxygenase PB1A11.03 && PF01070:FMN-dependent dehydrogenase
Lachesis_group0__13_contigs__l.g4022	351.558	992.3629	-1.49761	0.001594	0.026616	1518	- && - && PF02845:CUE domain
Lachesis_group0__13_contigs__l.g2127	1709.371	4834.55	-1.49997	0.001249	0.0234	993	- && P47818.1 RecName: Full=Protein CCC1; AltName: Full=Cross-complementer of CSG1 protein 1 && PF01988:VIT family
Lachesis_group0__13_contigs__l.g9563	142.529	403.8223	-1.504	0.003692	0.043943	837	- && - && PF08208:DNA-directed RNA polymerase I subunit RPA34.5

Lachesis_group0__13_contigs__l.g7952	437.1153	1239.698	-1.50432	0.00033	0.009465	1236	- && Q553X7.1 RecName: Full=UDP-glucose 4-epimerase; AltName: Full=Galactowaldenase; AltName: Full=UDP-N-acetylglucosamine 4-epimerase; Short=UDP-GlcNAc 4-epimerase; AltName: Full=UDP-galactosamine 4-epimerase; Short=UDP-GalNAc 4-epimerase; AltName: Full=UDP-galactose 4-epimerase && PF16363:GDP-mannose 4,6 dehydratase
Lachesis_group0__13_contigs__l.g11398	1075.318	3050.217	-1.50432	0.000211	0.007026	2682	- && O60161.1 RecName: Full=U3 small nucleolar RNA-associated protein 4; Short=U3 snoRNA-associated protein 4; AltName: Full=U3 protein 4 required for transcription && -
Lachesis_group0__13_contigs__l.g2246	2875.734	8177.109	-1.50761	0.000796	0.017384	1671	- && P9WGQ4.1 RecName: Full=Uncharacterized oxidoreductase MT0954 >P9WGQ5.1 RecName: Full=Uncharacterized oxidoreductase Rv0927c && PF13561:Enoyl-(Acyl carrier protein) reductase|PF00106:short chain dehydrogenase|PF01596:O-methyltransferase
Lachesis_group0__13_contigs__l.g8691	163.0328	463.8924	-1.50951	0.000429	0.011142	1098	- && Q54S38.2 RecName: Full=Probable RNA 3'-terminal phosphate cyclase-like protein && PF01137:RNA 3'-terminal phosphate cyclase|PF05189:RNA 3'-terminal phosphate cyclase (RTC), insert domain
Lachesis_group0__13_contigs__l.g9202	40.84483	116.4049	-1.51072	0.000198	0.006685	2436	- && P41410.2 RecName: Full=DNA repair protein rhp54; AltName: Full=RAD54 homolog 1 && PF08658:Rad54 N terminal|PF00176:SNF2 family N-terminal domain|PF00271:Helicase conserved C-terminal domain

Lachesis_group0__13_contigs__l.g6933	155.4746	442.8372	-1.51085	0.000227	0.00734	1146	- && O74777.1 RecName: Full=KRR1 small subunit processome component homolog; AltName: Full=KRR-R motif-containing protein 1; AltName: Full=Ribosomal RNA assembly protein mis3 && -
Lachesis_group0__13_contigs__l.g6965	359.3582	1024.194	-1.51127	0.001471	0.025764	3237	- && P33520.1 RecName: Full=Cell division cycle-related protein res1/sct1; AltName: Full=p72sct1 && PF13637:Ankyrin repeats (many copies)|PF13857:Ankyrin repeats (many copies)
Lachesis_group0__13_contigs__l.g3902	617.2383	1759.73	-1.51181	0.000697	0.015942	4077	- && O14228.1 RecName: Full=Probable phosphoribosylformylglycinamidine synthase; Short=FGAM synthase; Short=FGAMS; AltName: Full=Formylglycinamide ribonucleotide amidotransferase; Short=FGAR amidotransferase; Short=FGAR-AT; AltName: Full=Formylglycinamide ribotide amidotransferase && PF13507:CobB/CobQ-like glutamine amidotransferase domain|PF02769:AIR synthase related protein, C-terminal domain
Lachesis_group0__13_contigs__l.g2385	75.67279	216.7156	-1.51719	0.000303	0.008944	1035	- && Q4R0J7.1 RecName: Full=D-arabinitol dehydrogenase 1; AltName: Full=NADP-dependent D-arabitol dehydrogenase && PF16912:Glucose dehydrogenase C-terminus|PF08240:Alcohol dehydrogenase GroES-like domain
Lachesis_group0__13_contigs__l.g10832	570.3131	1632.336	-1.51736	0.00012	0.00475	1794	- && P78750.3 RecName: Full=Probable U3 small nucleolar RNA-associated protein 18; Short=U3 snoRNA-associated protein 18 && -
Lachesis_group0__13_contigs__l.g10984	608.3792	1752.184	-1.52639	0.000566	0.013446	972	- && O74919.1 RecName: Full=RNA-binding protein rnc1; AltName: Full=RNA-binding protein that

							suppresses calcineurin deletion 1 && PF00013:KH domain
Lachesis_group0__13_contigs__l.g6720	1155.068	3331.528	-1.52838	0.000713	0.016088	2298	- && Q9UT19.1 RecName: Full=Probable 5-methyltetrahydropteroyltriglutamate--homocysteine methyltransferase; AltName: Full=Cobalamin-independent methionine synthase; AltName: Full=Methionine synthase, vitamin-B12 independent isozyme && PF08267:Cobalamin-independent synthase, N-terminal domain|PF01717:Cobalamin-independent synthase, Catalytic domain
Lachesis_group0__13_contigs__l.g5790	149.678	431.5852	-1.52872	0.001735	0.027854	1764	- && O13345.1 RecName: Full=O-methylsterigmatocystin oxidoreductase; Short=OMST oxidoreductase; AltName: Full=Aflatoxin B synthase; AltName: Full=Aflatoxin biosynthesis protein Q; AltName: Full=Cytochrome P450 64 && PF00067:Cytochrome P450
Lachesis_group0__13_contigs__l.g6672	110.3096	318.9973	-1.53284	0.000747	0.016582	1632	- && Q3UFB2.2 RecName: Full=Box C/D snoRNA protein 1; AltName: Full=Zinc finger HIT domain-containing protein 6 && PF04438:HIT zinc finger
Lachesis_group0__13_contigs__l.g1279	16.48983	47.80343	-1.54063	0.00426	0.048315	900	- && - && PF01328:Peroxidase, family 2
Lachesis_group0__13_contigs__l.g5549	41.56548	121.276	-1.54773	0.002106	0.031301	807	- && - && PF10197:N-terminal domain of CBF1 interacting co-repressor CIR
Lachesis_group0__13_contigs__l.g3907	140.5508	411.3351	-1.55054	0.001118	0.021898	1695	- && - && PF12937:F-box-like
Lachesis_group0__13_contigs__l.g5672	261.0913	765.2675	-1.55196	0.00105	0.021054	801	- && P0CS81.1 RecName: Full=tRNA (guanine-N(7)-)-methyltransferase; AltName: Full=Transfer RNA methyltransferase 8; AltName: Full=tRNA (guanine(46)-N(7))-methyltransferase; AltName: Full=tRNA(m7G46)-methyltransferase >P0CS80.1

							RecName: Full=tRNA (guanine-N(7)-)-methyltransferase; AltName: Full=Transfer RNA methyltransferase 8; AltName: Full=tRNA (guanine(46)-N(7))-methyltransferase; AltName: Full=tRNA(m7G46)-methyltransferase && PF02390:Putative methyltransferase
Lachesis_group0__13_contigs__l.g3554	60.58245	177.7111	-1.55282	0.004178	0.047497	753	- && Q9P7C8.1 RecName: Full=DNA replication complex GINS protein sld5 && PF16922:DNA replication complex GINS protein SLD5 C-terminus
Lachesis_group0__13_contigs__l.g4302	642.2169	1885.443	-1.55405	7.06E-05	0.00326	3756	- && Q12754.1 RecName: Full=Ribosomal RNA-processing protein 12 && PF08161:NUC173 domain
Lachesis_group0__13_contigs__l.g9455	4598.645	13510.52	-1.55481	0.000762	0.016781	1521	- && P78567.3 RecName: Full=Histone H2B && PF00125:Core histone H2A/H2B/H3/H4
Lachesis_group0__13_contigs__l.g8137	125.8644	369.832	-1.55649	0.000368	0.01003	1389	- && P0CS07.1 RecName: Full=tRNA (adenine(58)-N(1))-methyltransferase non-catalytic subunit TRM6; AltName: Full=tRNA(m1A58)-methyltransferase subunit TRM6; Short=tRNA(m1A58)MTase subunit TRM6 >P0CS06.1 RecName: Full=tRNA (adenine(58)-N(1))-methyltransferase non-catalytic subunit TRM6; AltName: Full=tRNA(m1A58)-methyltransferase subunit TRM6; Short=tRNA(m1A58)MTase subunit TRM6 && PF04189:Gcd10p family
Lachesis_group0__13_contigs__l.g3985	519.5361	1534.33	-1.56244	0.000592	0.013931	1209	- && - && PF09729:Gti1/Pac2 family
Lachesis_group0__13_contigs__l.g117	25.96956	76.90079	-1.56603	0.001985	0.030369	1779	- && Q828K5.1 RecName: Full=Glycerol kinase 1; AltName: Full=ATP:glycerol 3-phosphotransferase 1; AltName: Full=Glycerokinase 1; Short=GK 1 && PF02782:FGGY family of carbohydrate kinases, C-

							terminal domain丨PF00370:FGGY family of carbohydrate kinases, N-terminal domain
Lachesis_group0__13_contigs__l.g6280	129.8918	384.7747	-1.56825	0.000288	0.008697	588	- && - && PF14615:Ribosome-assembly protein 3
Lachesis_group0__13_contigs__l.g4114	84.20261	250.1047	-1.57122	0.003938	0.045559	1482	- && Q10475.1 RecName: Full=Eukaryotic translation initiation factor 4 gamma; Short=eIF-4-gamma; Short=eIF-4G && PF02854:MIF4G domain丨PF12152:Eukaryotic translation initiation factor 4G1
Lachesis_group0__13_contigs__l.g6892	1066.369	3173.925	-1.57374	0.000183	0.006428	2034	- && P40010.1 RecName: Full=Nuclear GTP-binding protein NUG1; AltName: Full=Nuclear GTPase 1 && PF01926:50S ribosome-binding GTPase丨PF08701:GNL3L/Grn1 putative GTPase
Lachesis_group0__13_contigs__l.g5917	2347.871	6999.222	-1.57588	4.02E-06	0.00047	1050	- && - && PF03036:Perilipin family
Lachesis_group0__13_contigs__l.g448	4341.377	12944.96	-1.57617	0.001284	0.023904	1776	- && Q9HGX4.3 RecName: Full=Histone H2A && PF00125:Core histone H2A/H2B/H3/H4丨PF14622:Ribonuclease-III-like丨PF16211:C-terminus of histone H2A丨PF00035:Double-stranded RNA binding motif
Lachesis_group0__13_contigs__l.g6835	916.6891	2733.887	-1.57656	0.000543	0.01305	1665	- && P28745.2 RecName: Full=Protein pim1; AltName: Full=Poly(A)+ RNA transport protein 2 && PF00415:Regulator of chromosome condensation (RCC1) repeat
Lachesis_group0__13_contigs__l.g9173	80.01462	239.8701	-1.58406	0.001869	0.029189	1332	- && Q6C656.1 RecName: Full=Chromosome segregation in meiosis protein 3 && PF07962:Replication Fork Protection Component Swi3
Lachesis_group0__13_contigs__l.g10811	2076.898	6226.551	-1.58411	0.000634	0.014659	1239	- && P47176.1 RecName: Full=Branched-chain-amino-acid aminotransferase, cytosolic; Short=BCAT;

							AltName: Full=Protein TWT2 && PF01063:Amino-transferase class IV
Lachesis_group0__13_contigs__l.g466	220.2166	660.3241	-1.58518	0.000318	0.009162	1467	- && Q4PHV3.1 RecName: Full=Pre-rRNA-processing protein IPI3 && -
Lachesis_group0__13_contigs__l.g3357	117.1225	351.3517	-1.58557	0.000688	0.015796	2286	- && - && PF00069:Protein kinase domain
Lachesis_group0__13_contigs__l.g9052	74.33679	223.325	-1.58838	0.000423	0.011039	1140	- && - && PF13878:zinc-finger of acetyl-transferase ESCO|PF13880:ESCO1/2 acetyl-transferase
Lachesis_group0__13_contigs__l.g8683	1021.431	3096.205	-1.60009	0.000387	0.010428	759	- && P38523.1 RecName: Full=GrpE protein homolog, mitochondrial; Flags: Precursor && PF01025:GrpE
Lachesis_group0__13_contigs__l.g932	412.5452	1251.099	-1.60065	4.98E-05	0.002538	801	- && - && PF13279:Thioesterase-like superfamily
Lachesis_group0__13_contigs__l.g9428	390.5605	1185.818	-1.60234	0.001806	0.028487	969	- && O74741.2 RecName: Full=4,5-DOPA dioxygenase extradiol-like protein && PF02900:Catalytic LigB subunit of aromatic ring-opening dioxygenase
Lachesis_group0__13_contigs__l.g138	565.2074	1719.992	-1.60594	0.000369	0.01003	4479	- && Q4P7M1.1 RecName: Full=ATP-dependent RNA helicase DBP9 && PF00270:DEAD/DEAH box helicase|PF00271:Helicase conserved C-terminal domain
Lachesis_group0__13_contigs__l.g1296	475.2896	1446.68	-1.60608	0.000278	0.008463	1386	- && C5PEI9.1 RecName: Full=Aspartic protease PEP3; Flags: Precursor && PF00026:Eukaryotic aspartyl protease
Lachesis_group0__13_contigs__l.g165	310.8304	946.2483	-1.60671	7.59E-05	0.003385	3171	- && O36021.1 RecName: Full=Uncharacterized protein C4F10.09c && PF03914:CBF/Mak21 family
Lachesis_group0__13_contigs__l.g2375	833.3991	2541.314	-1.60875	0.000418	0.011018	1932	- && O42644.1 RecName: Full=CTP synthase; AltName: Full=CTP synthetase; AltName: Full=UTP--ammonia ligase && PF06418:CTP synthase N-terminus|PF00117:Glutamine amidotransferase class-I
Lachesis_group0__13_contigs__l.g3516	1009.002	3079.805	-1.61003	0.00015	0.005572	1794	- && O94300.1 RecName: Full=Putative xanthine/uracil permease C887.17 && PF00860:Permease family

Lachesis_group0__13_contigs__l.g2514	453.4909	1385.401	-1.61139	4.40E-05	0.002353	1356	- && B0DWM8.1 RecName: Full=Ribosome biogenesis protein YTM1 && PF08154:NLE (NUC135) domain
Lachesis_group0__13_contigs__l.g4542	79.7189	244.1378	-1.61461	0.001939	0.030022	1317	- && O59790.2 RecName: Full=Serine/threonine-protein kinase ark1; AltName: Full=Aurora-related kinase 1 && PF00069:Protein kinase domain
Lachesis_group0__13_contigs__l.g5995	99.81048	305.8414	-1.61674	0.000499	0.012331	2304	- && P41889.2 RecName: Full=Anaphase-promoting complex subunit cut9; AltName: Full=20S cyclosome/APC complex protein cut9; AltName: Full=Cell untimely torn protein 9 && PF12895:Anaphase-promoting complex, cyclosome, subunit 3|PF13424:Tetratricopeptide repeat
Lachesis_group0__13_contigs__l.g10027	141.358	433.8672	-1.61815	0.000532	0.012853	2718	- && P40377.1 RecName: Full=DNA replication licensing factor mcm2; AltName: Full=Cell division control protein 19; AltName: Full=Minichromosome maintenance protein 2 && PF12619:Mini-chromosome maintenance protein 2|PF00493:MCM2/3/5 family|PF14551:MCM N-terminal domain
Lachesis_group0__13_contigs__l.g2057	117.655	361.7031	-1.61929	0.000117	0.004683	1098	- && - && PF05368:NmrA-like family
Lachesis_group0__13_contigs__l.g8222	738.8266	2276.862	-1.62356	1.68E-06	0.000247	2214	- && Q8TB72.2 RecName: Full=Pumilio homolog 2; Short=Pumilio-2 && -
Lachesis_group0__13_contigs__l.g11369	3063.992	9448.926	-1.62478	8.89E-05	0.003773	1920	- && Q9P7H3.1 RecName: Full=Prohibitin-1 && PF00076:RNA recognition motif. (a.k.a. RRM, RBD, or RNP domain)|PF01145:SPFH domain / Band 7 family
Lachesis_group0__13_contigs__l.g8645	776.6874	2395.515	-1.62511	0.0002	0.00673	381	- && P0CQ53.1 RecName: Full=13 kDa ribonucleoprotein-associated protein >P0CQ52.1 RecName: Full=13 kDa ribonucleoprotein-associated protein && PF01248:Ribosomal protein L7Ae/L30e/S12e/Gadd45 family

Lachesis_group0__13_contigs__l.g4599	48.24326	148.7744	-1.62581	0.001498	0.025804	1476	- && P38121.1 RecName: Full=DNA polymerase alpha subunit B; AltName: Full=DNA polymerase I subunit B; AltName: Full=DNA polymerase alpha:primase complex p86 subunit; Short=Pol alpha-primase complex p86 subunit; AltName: Full=DNA polymerase-primase complex p74 subunit && PF04042:DNA polymerase alpha/epsilon subunit B|PF08418:DNA polymerase alpha subunit B N-terminal
Lachesis_group0__13_contigs__l.g4533	281.7834	871.7198	-1.62912	0.002745	0.036066	816	- && Q9DCW4.3 RecName: Full=Electron transfer flavoprotein subunit beta; Short=Beta-ETF && PF01012:Electron transfer flavoprotein domain
Lachesis_group0__13_contigs__l.g11296	529.3742	1651.165	-1.64145	8.10E-06	0.000759	1842	- && - && PF01490:Transmembrane amino acid transporter protein
Lachesis_group0__13_contigs__l.g10934	216.5559	675.3659	-1.64163	1.83E-05	0.001271	3729	- && Q04217.1 RecName: Full=Probable ATP-dependent RNA helicase DHR1; AltName: Full=DEAH box RNA helicase DHR1; AltName: Full=Extracellular mutant protein 16 && PF07717:Oligonucleotide/oligosaccharide-binding (OB)-fold|PF00270:DEAD/DEAH box helicase|PF00271:Helicase conserved C-terminal domain|PF04408:Helicase associated domain (HA2)
Lachesis_group0__13_contigs__l.g8694	51.21698	160.0147	-1.64402	0.003665	0.043921	1278	- && O08760.2 RecName: Full=N-glycosylase/DNA lyase; Includes: RecName: Full=8-oxoguanine DNA glycosylase; Includes: RecName: Full=DNA-(apurinic or apyrimidinic site) lyase; Short=AP lyase && PF07934:8-oxoguanine DNA glycosylase, N-terminal

							domain|PF00730:HhH-GPD superfamily base excision DNA repair protein
Lachesis_group0__13_contigs__l.g8808	35.67034	111.6031	-1.6446	0.002292	0.03288	1119	- && Q09843.1 RecName: Full=Replication factor C subunit 2; Short=Replication factor C2; AltName: Full=Activator 1 41 kDa subunit && PF08542:Replication factor C C-terminal domain|PF00004:ATPase family associated with various cellular activities (AAA)
Lachesis_group0__13_contigs__l.g1178	53.72544	167.981	-1.64514	0.004477	0.049896	1881	- && - && PF01753:MYND finger
Lachesis_group0__13_contigs__l.g7977	206.227	646.3695	-1.64831	3.50E-05	0.001939	1389	- && O74731.1 RecName: Full=tRNA-dihydrouridine(20) synthase [NAD(P)+]; AltName: Full=tRNA-dihydrouridine synthase 2 && PF01207:Dihydrouridine synthase (Dus)
Lachesis_group0__13_contigs__l.g4810	37.86216	118.9384	-1.65219	0.001575	0.026446	1398	- && - && PF01231:Indoleamine 2,3-dioxygenase
Lachesis_group0__13_contigs__l.g6436	254.9878	802.8711	-1.65551	0.000299	0.008877	849	- && Q0VD01.1 RecName: Full=U3 small nucleolar ribonucleoprotein protein IMP4; Short=U3 snoRNP protein IMP4 && PF04427:Brix domain
Lachesis_group0__13_contigs__l.g362	96.48354	304.1346	-1.65762	0.000391	0.010476	1620	- && Q9HFE6.1 RecName: Full=Uncharacterized RNA-binding protein P16F5.06 && -
Lachesis_group0__13_contigs__l.g9084	50.12666	158.1534	-1.66006	0.001222	0.023072	3285	- && - && PF08457:Sfi1 spindle body protein
Lachesis_group0__13_contigs__l.g10689	123.0027	389.1893	-1.66262	7.58E-05	0.003385	1047	- && Q8YVU6.2 RecName: Full=Porphobilinogen deaminase; Short=PBG; AltName: Full=Hydroxymethylbilane synthase; Short=HMBS; AltName: Full=Pre-uroporphyrinogen synthase && PF03900:Porphobilinogen deaminase, C-terminal domain|PF01379:Porphobilinogen deaminase, dipyromethane cofactor binding domain

Lachesis_group0__13_contigs__l.g5780	422.9241	1339.878	-1.66375	0.001391	0.025053	2025	- && P42670.1 RecName: Full=Puromycin resistance protein pur8 && PF07690:Major Facilitator Superfamily
Lachesis_group0__13_contigs__l.g11270	696.5009	2209.559	-1.66585	0.000221	0.007243	1644	- && O74407.1 RecName: Full=Aspartate--tRNA ligase, cytoplasmic; AltName: Full=Aspartyl-tRNA synthetase; Short=AspRS && PF01336:OB-fold nucleic acid binding domain|PF00152:tRNA synthetases class II (D, K and N)
Lachesis_group0__13_contigs__l.g11244	50.88764	162.1925	-1.67182	0.000631	0.014659	3738	- && Q1MTN3.2 RecName: Full=Inner centromere protein-related protein pic1; Short=INCENP-related protein pic1 && PF03941:Inner centromere protein, ARK binding region
Lachesis_group0__13_contigs__l.g8419	19.86382	63.11099	-1.67299	0.001961	0.030126	363	- && P0CM85.1 RecName: Full=Cytochrome c oxidase assembly protein COX16, mitochondrial; Flags: Precursor >P0CM84.1 RecName: Full=Cytochrome c oxidase assembly protein COX16, mitochondrial; Flags: Precursor && PF14138:Cytochrome c oxidase assembly protein COX16
Lachesis_group0__13_contigs__l.g5204	284.4165	907.7498	-1.67474	2.19E-05	0.001435	1143	- && Q1MTN8.2 RecName: Full=Probable assembly chaperone of rpl4 && PF14559:Tetratricopeptide repeat
Lachesis_group0__13_contigs__l.g2258	117.239	374.6903	-1.67618	5.29E-05	0.00265	3666	- && P0CR92.1 RecName: Full=Topoisomerase 1-associated factor 1 && PF05029:Timeless protein C terminal region|PF04821:Timeless protein
Lachesis_group0__13_contigs__l.g10	6545.29	20925.79	-1.67675	0.002743	0.036066	1548	- && P07871.2 RecName: Full=3-ketoacyl-CoA thiolase B, peroxisomal; AltName: Full=Acetyl-CoA acyltransferase B; AltName: Full=Beta-ketothiolase B; AltName: Full=Peroxisomal 3-oxoacyl-CoA thiolase B;

							Flags: Precursor && PF00108:Thiolase, N-terminal domain|PF02803:Thiolase, C-terminal domain
Lachesis_group0__13_contigs__l.g2330	290.1429	928.7242	-1.67871	5.60E-06	0.000603	1503	- && Q874L4.1 RecName: Full=Vitamin B6 transporter TPN1; AltName: Full=Transport of pyridoxine protein 1 && PF02133:Permease for cytosine/purines, uracil, thiamine, allantoin
Lachesis_group0__13_contigs__l.g817	186.9316	599.0814	-1.68004	0.002276	0.032806	1779	- && Q70J59.1 RecName: Full=Tripeptidyl-peptidase sed2; AltName: Full=Sedolisin-B; Flags: Precursor && PF09286:Pro-kumamolisin, activation domain|PF00082:Subtilase family
Lachesis_group0__13_contigs__l.g7938	298.5988	956.7053	-1.68041	7.44E-05	0.003365	1764	- && O14360.1 RecName: Full=H/ACA ribonucleoprotein complex non-core subunit NAF1; AltName: Full=Nuclear assembly factor 1 && PF04410:Gar1/Naf1 RNA binding region
Lachesis_group0__13_contigs__l.g8375	15.34052	49.09317	-1.68103	0.003533	0.043156	1092	- && - && PF12697:Alpha/beta hydrolase family
Lachesis_group0__13_contigs__l.g5212	2018.678	6481.724	-1.68298	0.000348	0.009761	1251	- && O13982.1 RecName: Full=Uncharacterized WD repeat-containing protein C25H1.08c && PF00400:WD domain, G-beta repeat
Lachesis_group0__13_contigs__l.g10384	147.1681	472.6681	-1.68397	0.000373	0.010093	1980	- && Q99741.1 RecName: Full=Cell division control protein 6 homolog; AltName: Full=CDC6-related protein; AltName: Full=Cdc18-related protein; Short=HsCdc18; AltName: Full=p62(cdc6); Short=HsCDC6 && PF13401:AAA domain
Lachesis_group0__13_contigs__l.g1508	451.9293	1456.397	-1.68858	2.47E-05	0.001543	2034	- && Q3SYU1.1 RecName: Full=Nucleolar complex protein 2 homolog; Short=Protein NOC2 homolog; AltName: Full=NOC2-like protein; AltName: Full=Novel INHAT repressor && PF03715:Noc2p family

Lachesis_group0__13_contigs__l.g11694	32.71455	106.0267	-1.69408	0.000707	0.01606	741	- && - && PF00808:Histone-like transcription factor (CBF/NF-Y) and archaeal histone
Lachesis_group0__13_contigs__l.g7753	487.5982	1579.43	-1.69588	0.000196	0.006685	1677	- && P87153.1 RecName: Full=Probable T-complex protein 1 subunit eta; Short=TCP-1-eta; AltName: Full=CCT-eta && PF00118:TCP-1/cpn60 chaperonin family
Lachesis_group0__13_contigs__l.g5796	1083.509	3514.115	-1.69758	0.000357	0.0099	1803	- && Q05506.1 RecName: Full=Arginine--tRNA ligase, cytoplasmic; AltName: Full=Arginyl-tRNA synthetase; Short=ArgRS && PF00750:tRNA synthetases class I (R)|PF05746:DALR anticodon binding domain
Lachesis_group0__13_contigs__l.g9073	52.76208	171.8493	-1.70454	0.003603	0.043439	1287	- && - && PF12937:F-box-like
Lachesis_group0__13_contigs__l.g1021	42.70135	139.4972	-1.70536	0.001887	0.029418	1074	- && O14003.2 RecName: Full=Replication factor C subunit 3; Short=Replication factor C3 && PF00004:ATPase family associated with various cellular activities (AAA)|PF08542:Replication factor C C-terminal domain
Lachesis_group0__13_contigs__l.g10640	981.8913	3207.465	-1.70777	2.98E-06	0.000382	5436	- && O77033.1 RecName: Full=General transcriptional corepressor trfA && PF13414:TPR repeat
Lachesis_group0__13_contigs__l.g8083	314.4456	1028.608	-1.71035	0.000611	0.014308	978	- && Q9UU99.1 RecName: Full=CRAL-TRIO domain-containing protein C23B6.04c && PF00650:CRAL/TRIO domain
Lachesis_group0__13_contigs__l.g10018	13.73493	45.00309	-1.71293	0.001551	0.026227	1284	- && P18773.2 RecName: Full=Esterase && PF07859:alpha/beta hydrolase fold
Lachesis_group0__13_contigs__l.g6437	93.88547	307.5106	-1.71299	0.000118	0.004683	1407	- && O36014.1 RecName: Full=Aspartyl aminopeptidase 1 && PF02127:Aminopeptidase I zinc metalloprotease (M18)

Lachesis_group0__13_contigs__l.g5341	43.25472	143.0135	-1.72543	0.004391	0.04915	903	- && Q9P4T7.1 RecName: Full=Thymidylate synthase; Short=TS; Short=TSase && PF00303:Thymidylate synthase
Lachesis_group0__13_contigs__l.g10132	560.982	1855.328	-1.72596	5.27E-05	0.00265	2502	- && P0CQ95.1 RecName: Full=ATP-dependent RNA helicase DBP7 >P0CQ94.1 RecName: Full=ATP-dependent RNA helicase DBP7 && PF13959:Domain of unknown function (DUF4217)|PF00270:DEAD/DEAH box helicase|PF00271:Helicase conserved C-terminal domain
Lachesis_group0__13_contigs__l.g2011	326.631	1080.604	-1.72671	6.93E-05	0.003229	2781	- && Q10155.1 RecName: Full=Ribonuclease Z 1; Short=RNase Z 1; AltName: Full=tRNA 3 endonuclease 1; AltName: Full=tRNase Z 1 && PF12706:Beta-lactamase superfamily domain|PF13691:tRNase Z endonuclease
Lachesis_group0__13_contigs__l.g5564	80.89956	268.9306	-1.73158	0.001732	0.027854	1839	- && Q8NK72.2 RecName: Full=DNA repair and recombination protein rhm52; AltName: Full=RAD52 homolog && PF04098:Rad52/22 family double-strand break repair protein
Lachesis_group0__13_contigs__l.g5556	106.0506	354.8455	-1.7432	0.000709	0.01606	732	- && - && PF03070:TENA/THI-4/PQQC family
Lachesis_group0__13_contigs__l.g342	87.366	292.3537	-1.74422	8.61E-05	0.003701	2730	- && Q24K12.1 RecName: Full=G patch domain-containing protein 1 && PF07713:Protein of unknown function (DUF1604)|PF01585:G-patch domain
Lachesis_group0__13_contigs__l.g2754	26.66706	89.29641	-1.74463	0.000967	0.019961	1233	- && - && PF13414:TPR repeat
Lachesis_group0__13_contigs__l.g6208	283.5054	951.1281	-1.74697	0.000194	0.006685	1023	- && A8P7Y3.1 RecName: Full=S-methyl-5'-thioadenosine phosphorylase; AltName: Full=5'-methylthioadenosine phosphorylase; Short=MTA phosphorylase; Short=MTAP; Short=MTAPase && PF01048:Phosphorylase superfamily

Lachesis_group0__13_contigs__l.g2728	3837.218	12881.72	-1.74719	0.000219	0.007211	3708	- && - && PF00172:Fungal Zn(2)-Cys(6) binuclear cluster domain|PF04082:Fungal specific transcription factor domain
Lachesis_group0__13_contigs__l.g10720	13.41978	44.99739	-1.74873	0.003374	0.041914	903	- && - && PF00069:Protein kinase domain
Lachesis_group0__13_contigs__l.g10436	71.44672	241.05	-1.75465	0.000312	0.009125	3405	- && Q9C107.1 RecName: Full=Minichromosome loss protein 1; AltName: Full=DNA polymerase alpha accessory factor Mcl1 && PF12341:Minichromosome loss protein, Mcl1, middle region
Lachesis_group0__13_contigs__l.g1011	388.2843	1311.052	-1.75601	1.14E-05	0.000919	3585	- && O94372.1 RecName: Full=Uncharacterized protein C1604.06c && PF03914:CBF/Mak21 family
Lachesis_group0__13_contigs__l.g248	408.3497	1380.577	-1.75759	2.33E-05	0.001488	2244	- && - && PF12767:Transcriptional regulator of RNA polII, SAGA, subunit|PF05132:RNA polymerase III RPC4
Lachesis_group0__13_contigs__l.g8472	945.6683	3199.523	-1.75865	0.000167	0.00601	1500	- && Q51342.3 RecName: Full=Amidophosphoribosyltransferase; Short=ATase; AltName: Full=Glutamine phosphoribosylpyrophosphate amidotransferase; Short=GPATase && PF00156:Phosphoribosyl transferase domain|PF13522:Glutamine amidotransferase domain
Lachesis_group0__13_contigs__l.g302	69.70746	235.9537	-1.75928	0.000347	0.009761	1857	- && Q5AXT5.2 RecName: Full=ATP-dependent DNA helicase PIF1; AltName: Full=DNA repair and recombination helicase PIF1 && PF05970:PIF1-like helicase
Lachesis_group0__13_contigs__l.g10249	77.31647	262.3468	-1.76338	0.000229	0.007361	2199	- && - && PF13964:Kelch motif
Lachesis_group0__13_contigs__l.g11551	507.4254	1727.368	-1.76741	6.17E-05	0.002993	1401	- && P40510.1 RecName: Full=D-3-phosphoglycerate dehydrogenase 2; Short=3-PGDH 2 && PF00389:D-isomer specific 2-hydroxyacid dehydrogenase, catalytic

							domain|PF02826:D-isomer specific 2-hydroxyacid dehydrogenase, NAD binding domain
Lachesis_group0__13_contigs__l.g3864	83.03612	282.6378	-1.7679	6.36E-05	0.003042	1710	- && - && PF12937:F-box-like
Lachesis_group0__13_contigs__l.g8303	146.6005	500.8516	-1.77372	4.86E-05	0.002531	3084	- && P87137.1 RecName: Full=Uncharacterized protein C57A7.06 && PF04615:Utp14 protein
Lachesis_group0__13_contigs__l.g7510	464.7061	1589.867	-1.77481	1.23E-05	0.000945	1689	- && O94365.1 RecName: Full=U3 small nucleolar RNA-associated protein 15; Short=U3 snoRNA-associated protein 15; AltName: Full=U3 protein 15 required for transcription && PF09384:UTP15 C terminal
Lachesis_group0__13_contigs__l.g2257	233.7746	801.5374	-1.77847	2.10E-05	0.001392	2394	- && Q4P5U4.2 RecName: Full=ATP-dependent RNA helicase DBP4 && PF00270:DEAD/DEAH box helicase|PF13959:Domain of unknown function (DUF4217)|PF00271:Helicase conserved C-terminal domain
Lachesis_group0__13_contigs__l.g5314	108.3433	372.8195	-1.78317	1.18E-05	0.000937	2082	- && Q76PD3.1 RecName: Full=ATP-dependent RNA helicase dbp6 && PF00270:DEAD/DEAH box helicase|PF00271:Helicase conserved C-terminal domain
Lachesis_group0__13_contigs__l.g7937	580.8496	2002.032	-1.7854	6.91E-05	0.003229	1032	- && A8N2Z9.1 RecName: Full=Elongation factor Ts, mitochondrial; Short=EF-Ts; Short=EF-TsMt; Flags: Precursor && PF00889:Elongation factor TS
Lachesis_group0__13_contigs__l.g1359	1785.362	6179.811	-1.79136	0.002442	0.033962	2835	- && - && PF04082:Fungal specific transcription factor domain
Lachesis_group0__13_contigs__l.g6468	293.717	1017.119	-1.7923	6.76E-06	0.000687	876	- && O13774.1 RecName: Full=GTP cyclohydrolase 1; AltName: Full=GTP cyclohydrolase I; Short=GTP-CH-I && PF01227:GTP cyclohydrolase I

Lachesis_group0__13_contigs__l.g4415	72.89847	253.4279	-1.79857	0.000313	0.009125	1533	- && Q08220.1 RecName: Full=Glutathione synthetase; Short=GSH synthetase; Short=GSH-S; AltName: Full=Glutathione synthase && PF03917:Eukaryotic glutathione synthase, ATP binding domain|PF03199:Eukaryotic glutathione synthase
Lachesis_group0__13_contigs__l.g11358	72.78497	253.0088	-1.79877	0.000314	0.009125	3282	- && O13921.2 RecName: Full=MutS protein homolog 1 && PF00488:MutS domain V|PF05192:MutS domain III|PF01624:MutS domain I|PF05188:MutS domain II
Lachesis_group0__13_contigs__l.g2129	129.9165	452.3228	-1.80059	2.01E-05	0.001366	1668	- && Q10286.1 RecName: Full=Myo-inositol transporter 1 && PF00083:Sugar (and other) transporter
Lachesis_group0__13_contigs__l.g7974	23.99132	83.39364	-1.80175	0.000299	0.008877	2049	- && Q9P6S5.1 RecName: Full=DASH complex subunit ask1; AltName: Full=Associated with spindles and kinetochores protein 1; AltName: Full=Meiotically up-regulated gene 181 protein; AltName: Full=Outer kinetochore protein ask1 && PF08655:DASH complex subunit Ask1
Lachesis_group0__13_contigs__l.g4773	253.8191	885.1544	-1.80218	9.67E-05	0.004026	1614	- && Q9ZWB3.1 RecName: Full=Casein kinase 1-like protein 9; AltName: Full=Dual specificity kinase 1; AltName: Full=Protein CASEIN KINASE I-LIKE 9; AltName: Full=Protein CASEIN KINASE I-LIKE 9 ALPHA; AltName: Full=Protein CASEIN KINASE I-LIKE 9 BETA && PF00069:Protein kinase domain
Lachesis_group0__13_contigs__l.g6809	676.3616	2372.455	-1.81078	7.70E-06	0.000744	5298	- && P15398.2 RecName: Full=DNA-directed RNA polymerase I subunit rpa1; AltName: Full=DNA-directed RNA polymerase I 190 kDa polypeptide; AltName: Full=DNA-directed RNA polymerase I largest subunit && PF04998:RNA polymerase Rpb1,

							domain 5|PF04983:RNA polymerase Rpb1, domain 3|PF04997:RNA polymerase Rpb1, domain 1|PF00623:RNA polymerase Rpb1, domain 2|PF05000:RNA polymerase Rpb1, domain 4
Lachesis_group0__13_contigs__l.g6381	72.16364	253.2934	-1.81085	0.000956	0.019774	1347	- && Q75JQ3.1 RecName: Full=Peroxisome biogenesis factor 2; AltName: Full=Peroxin-2 && PF04757:Pex2 / Pex12 amino terminal region
Lachesis_group0__13_contigs__l.g679	137.6861	483.0872	-1.81152	9.63E-05	0.004026	2040	- && - && PF08615:Ribonuclease H2 non-catalytic subunit (Ylr154p-like)|PF08325:WLM domain|PF00240:Ubiquitin family
Lachesis_group0__13_contigs__l.g5708	187.24	667.4837	-1.83441	1.95E-06	0.000272	2418	- && O14325.1 RecName: Full=Uncharacterized AAA domain-containing protein C16E9.10c && PF00004:ATPase family associated with various cellular activities (AAA)
Lachesis_group0__13_contigs__l.g2013	66.86368	239.0437	-1.83842	2.57E-05	0.001575	2442	- && - && PF07814:Wings apart-like protein regulation of heterochromatin
Lachesis_group0__13_contigs__l.g3885	720.0405	2575.634	-1.83903	2.36E-05	0.001495	1755	- && Q9UUE6.1 RecName: Full=Lysine--tRNA ligase, cytoplasmic; AltName: Full=Lysyl-tRNA synthetase; Short=LysRS && PF01336:OB-fold nucleic acid binding domain|PF00152:tRNA synthetases class II (D, K and N)
Lachesis_group0__13_contigs__l.g676	38.63135	138.1435	-1.83946	0.000122	0.004764	2580	- && - && PF11559:Afadin- and alpha -actinin-Binding
Lachesis_group0__13_contigs__l.g6922	185.9637	666.9448	-1.84316	2.28E-05	0.001473	2373	- && A8P7F7.1 RecName: Full=Nucleolar protein 9; AltName: Full=Pumilio domain-containing protein NOP9 && -
Lachesis_group0__13_contigs__l.g7204	407.8031	1463.593	-1.84397	1.23E-05	0.000945	1422	- && Q9UTK0.1 RecName: Full=20S-pre-rRNA D-site endonuclease nob1; AltName: Full=Pre-rRNA-

							processing endonuclease nob1 && PF08772:Nin one binding (NOB1) Zn-ribbon like
Lachesis_group0__13_contigs__l.g10511	1474.98	5307.411	-1.84745	3.45E-05	0.001923	1890	- && P87142.3 RecName: Full=Heat shock protein 70 homolog C57A7.12 && PF00012:Hsp70 protein|PF10712:NAD-specific glutamate dehydrogenase
Lachesis_group0__13_contigs__l.g10121	82.46335	297.3292	-1.85064	0.002284	0.032866	1155	- && - && PF00248:Aldo/keto reductase family
Lachesis_group0__13_contigs__l.g8058	571.2332	2064.914	-1.85417	1.43E-06	0.000227	2850	- && Q5U2W5.1 RecName: Full=Transducin beta-like protein 3 && PF08625:Utp13 specific WD40 associated domain
Lachesis_group0__13_contigs__l.g10960	354.5938	1286.927	-1.85975	0.003794	0.044675	1830	- && A1CFL2.1 RecName: Full=Dehydrogenase patE; AltName: Full=Patulin synthesis protein E; Flags: Precursor && PF00732:GMC oxidoreductase|PF05199:GMC oxidoreductase
Lachesis_group0__13_contigs__l.g4540	66.80394	243.0921	-1.86488	0.000263	0.008171	921	- && Q6ME51.1 RecName: Full=50S ribosomal protein L5 && PF00673:ribosomal L5P family C-terminus
Lachesis_group0__13_contigs__l.g4764	461.0947	1680.487	-1.86599	4.56E-06	0.000521	2367	- && O74879.1 RecName: Full=Ribosome biogenesis protein enp2 homolog && PF08159:NUC153 domain
Lachesis_group0__13_contigs__l.g5609	133.3965	487.5559	-1.86962	7.88E-05	0.003444	2427	- && A6QPL4.2 RecName: Full=Kinesin-like protein KIF22 && PF00225:Kinesin motor domain
Lachesis_group0__13_contigs__l.g675	65.86448	241.9297	-1.87982	0.000367	0.01003	1080	- && O14242.2 RecName: Full=Putative pyridoxal kinase C6F6.11c && PF08543:Phosphomethylpyrimidine kinase
Lachesis_group0__13_contigs__l.g11653	235.2442	866.715	-1.88198	1.33E-05	0.001003	3237	- && Q99385.1 RecName: Full=Vacuolar calcium ion transporter; AltName: Full=High copy number undoes manganese protein 1; AltName: Full=Manganese resistance 1 protein; AltName: Full=Vacuolar

							Ca(2+)/H(+) exchanger && PF02146:Sir2 family|PF01699:Sodium/calcium exchanger protein
Lachesis_group0__13_contigs__l.g6430	449.4433	1656.454	-1.88201	2.22E-05	0.001445	2172	- && O43000.2 RecName: Full=Pantothenate transporter liz1 && -
Lachesis_group0__13_contigs__l.g7875	183.3484	679.9375	-1.89067	3.28E-06	0.000408	1470	- && Q8C2P3.1 RecName: Full=tRNA-dihydrouridine(16/17) synthase [NAD(P)(+)]-like; AltName: Full=tRNA-dihydrouridine synthase 1-like && PF01207:Dihydrouridine synthase (Dus)
Lachesis_group0__13_contigs__l.g8301	146.7932	544.5363	-1.89196	0.0001	0.004133	1839	- && O74468.1 RecName: Full=Probable uroporphyrinogen-III C-methyltransferase && PF14823:Sirohaem biosynthesis protein C-terminal|PF13241:Putative NAD(P)-binding|PF00590:Tetrapyrrole (Corrin/Porphyrin) Methylases|PF14824:Sirohaem biosynthesis protein central
Lachesis_group0__13_contigs__l.g3366	148.4316	550.84	-1.89245	2.71E-05	0.00162	963	- && Q6BDR8.1 RecName: Full=Non-structural maintenance of chromosome element 4; Short=Non-SMC element 4; AltName: Full=DNA repair protein rad62 && PF08743:Nse4 C-terminal|PF15412:Binding domain of Nse4/EID3 to Nse3-MAGE
Lachesis_group0__13_contigs__l.g6517	151.9975	569.0504	-1.90404	0.00025	0.007911	540	- && Q6MVL2.1 RecName: Full=Deoxyuridine 5'-triphosphate nucleotidohydrolase; Short=dUTPase; AltName: Full=dUTP pyrophosphatase && PF00692:dUTPase
Lachesis_group0__13_contigs__l.g8723	23.71127	88.87668	-1.90607	7.25E-05	0.003329	3117	- && - && PF10193:Telomere length regulation protein
Lachesis_group0__13_contigs__l.g3687	85.41165	320.0975	-1.90748	6.98E-05	0.003238	372	- && Q09723.1 RecName: Full=Multifunctional methyltransferase subunit trm112; AltName: Full=eRF1 methyltransferase subunit trm112; Short=eRF1 MTase

							subunit trm112; AltName: Full=tRNA methyltransferase 112 homolog && PF03966:Trm112p-like protein
Lachesis_group0__13_contigs__l.g623	866.0054	3263.618	-1.91421	0.00029	0.008702	1839	- && P84285.1 RecName: Full=Sterol O-acyltransferase 2; AltName: Full=ASAT; AltName: Full=Sterol-ester synthase && PF03062:MBOAT, membrane-bound O-acyltransferase family
Lachesis_group0__13_contigs__l.g11036	245.2475	924.6617	-1.91478	0.000341	0.009657	1863	- && - && PF10297:Minimal binding motif of Hap4 for binding to Hap2/3/5
Lachesis_group0__13_contigs__l.g11157	104.3607	393.3342	-1.9154	2.35E-06	0.000319	1179	- && Q8Z8W3.1 RecName: Full=2-dehydropantoate 2-reductase; AltName: Full=Ketopantoate reductase; Short=KPA reductase; Short=KPR && PF02558:Ketopantoate reductase PanE/ApbA|PF08546:Ketopantoate reductase PanE/ApbA C terminal
Lachesis_group0__13_contigs__l.g7294	37.93235	143.4478	-1.92245	0.000124	0.004784	4167	- && - && PF12770:CHAT domain
Lachesis_group0__13_contigs__l.g2706	34.09088	129.0153	-1.9231	0.000172	0.006132	645	- && - && PF16015:Promethin
Lachesis_group0__13_contigs__l.g7942	20.29024	77.21297	-1.92541	8.40E-05	0.003628	3114	- && P97929.2 RecName: Full=Breast cancer type 2 susceptibility protein homolog; AltName: Full=Fanconi anemia group D1 protein homolog && PF09103:BRCA2, oligonucleotide/oligosaccharide-binding, domain 1
Lachesis_group0__13_contigs__l.g9064	193.8602	736.9571	-1.92756	4.58E-05	0.002424	1929	- && Q4P9E5.2 RecName: Full=ATP-dependent rRNA helicase SPB4 && PF00270:DEAD/DEAH box helicase|PF00271:Helicase conserved C-terminal domain|PF13959:Domain of unknown function (DUF4217)

Lachesis_group0__13_contigs__l.g9486	93.22755	356.7381	-1.93696	4.98E-05	0.002538	1944	- && O94481.2 RecName: Full=Transcription factor TFIIIB component B''; AltName: Full=Transcription factor TFIIIB complex subunit bdp1 && PF15963:Myb DNA-binding like
Lachesis_group0__13_contigs__l.g4546	561.3181	2160.843	-1.94495	7.62E-07	0.000145	1956	- && Q9UT32.1 RecName: Full=Putative ribosome biogenesis protein C8F11.04; AltName: Full=U3 snoRNP-associated protein C8F11.04 && PF00687:Ribosomal protein L1p/L10e family
Lachesis_group0__13_contigs__l.g5328	24.90464	96.24862	-1.94779	0.001004	0.020393	2970	- && Q10213.1 RecName: Full=ATP-dependent DNA helicase srs2 && PF13361:UvrD-like helicase C-terminal domain|PF00580:UvrD/REP helicase N-terminal domain
Lachesis_group0__13_contigs__l.g4900	263.1345	1022.159	-1.9581	1.20E-06	0.0002	1722	- && Q9P775.1 RecName: Full=Uncharacterized WD repeat-containing protein C17D11.16 && -
Lachesis_group0__13_contigs__l.g2228	187.3415	728.0367	-1.95881	2.72E-05	0.00162	1173	- && Q01752.1 RecName: Full=Aryl-alcohol dehydrogenase [NADP(+)]; Short=AAD && PF00248:Aldo/keto reductase family
Lachesis_group0__13_contigs__l.g4250	181.8332	713.0183	-1.97139	6.75E-06	0.000687	1431	- && O94636.2 RecName: Full=tRNA (guanine(10)-N2)-methyltransferase; AltName: Full=tRNA guanosine-2'-O-methyltransferase TRM11; Short=tRNA methylase 11 && PF13659:Methyltransferase domain
Lachesis_group0__13_contigs__l.g6510	313.9004	1231.082	-1.97218	5.78E-06	0.00061	4026	- && Q9Y767.2 RecName: Full=DNA polymerase gamma, mitochondrial; AltName: Full=Mitochondrial DNA polymerase catalytic subunit; Flags: Precursor && PF00476:DNA polymerase family A
Lachesis_group0__13_contigs__l.g9214	661.9822	2599.091	-1.97328	5.44E-05	0.002698	1455	- && P38680.2 RecName: Full=N amino acid transport system protein; AltName: Full=Methyltryptophan

							resistance protein && PF01490:Transmembrane amino acid transporter protein
Lachesis_group0__13_contigs__l.g5291	177.7319	701.5235	-1.9811	7.19E-06	0.000702	2406	- && P78963.2 RecName: Full=Protein arginine N-methyltransferase skb1; AltName: Full=Shk1 kinase-binding protein 1; AltName: Full=Type II protein arginine N-methyltransferase; Short=Type II PRMT && PF05185:PRMT5 arginine-N-methyltransferase|PF13857:Ankyrin repeats (many copies)
Lachesis_group0__13_contigs__l.g11370	55.45275	219.2845	-1.98561	7.97E-05	0.003457	1788	- && O74397.1 RecName: Full=Asparagine synthetase domain-containing protein C4F6.11c && PF00733:Asparagine synthase
Lachesis_group0__13_contigs__l.g2733	592.0425	2348.143	-1.98795	4.57E-05	0.002424	1989	- && Q5AKA5.1 RecName: Full=Cys-Gly metallodipeptidase DUG1; AltName: Full=Deficient in utilization of glutathione protein 1; AltName: Full=GSH degradosomal complex subunit DUG1 && PF07687:Peptidase dimerisation domain|PF01546:Peptidase family M20/M25/M40
Lachesis_group0__13_contigs__l.g8807	52.94206	210.7749	-1.9925	0.000316	0.009132	2754	- && - && PF12253:Chromatin assembly factor 1 subunit A
Lachesis_group0__13_contigs__l.g9612	17.40464	68.90354	-1.99275	0.001286	0.023904	291	- && - && PF05730:CFEM domain
Lachesis_group0__13_contigs__l.g148	104.3861	417.1962	-1.9991	2.51E-05	0.001555	1245	- && P49321.2 RecName: Full=Nuclear autoantigenic sperm protein; Short=NASP && PF10516:SHNi-TPR
Lachesis_group0__13_contigs__l.g9851	32.75786	131.4061	-2.00576	0.000653	0.015071	1155	- && Q14AI0.1 RecName: Full=Sister chromatid cohesion protein DCC1; AltName: Full=Defective in sister chromatid cohesion protein 1 homolog && PF09724:Uncharacterized conserved protein (DUF2036)

Lachesis_group0__13_contigs__l.g9329	55.67082	223.0857	-2.00582	0.000152	0.00561	1620	- && Q9UT75.1 RecName: Full=tRNA dimethylallyltransferase, mitochondrial; AltName: Full=Isopentenyl-diphosphate: tRNA isopentenyltransferase; Short=IPP transferase; Short=IPPT; AltName: Full=tRNA isopentenyltransferase; Short=IPTase && PF01715:IPP transferase
Lachesis_group0__13_contigs__l.g7387	13.01278	52.22069	-2.00632	0.001409	0.025242	1059	- && Q9P7F4.1 RecName: Full=Zinc-type alcohol dehydrogenase-like protein C2E1P3.01 && PF00107:Zinc-binding dehydrogenase
Lachesis_group0__13_contigs__l.g2096	99.98075	402.4498	-2.00896	4.57E-07	0.000113	3864	- && - && PF00498:FHA domain
Lachesis_group0__13_contigs__l.g5464	1776.883	7162.729	-2.01119	1.09E-05	0.000909	873	- && - && PF00652:Ricin-type beta-trefoil lectin domain
Lachesis_group0__13_contigs__l.g2229	370.3375	1497.433	-2.01596	2.68E-06	0.000354	1362	- && Q7ZYQ6.1 RecName: Full=DDB1- and CUL4-associated factor 13; AltName: Full=WD repeat and SOF domain-containing protein 1 && PF04158:Sof1-like domain|PF00400:WD domain, G-beta repeat
Lachesis_group0__13_contigs__l.g5027	43.12328	175.4765	-2.02588	0.000257	0.008015	1047	- && - && PF05368:NmrA-like family
Lachesis_group0__13_contigs__l.g5028	48.05657	197.0802	-2.03874	1.50E-05	0.001108	540	- && - && PF05368:NmrA-like family
Lachesis_group0__13_contigs__l.g452	179.9737	746.2492	-2.05196	1.20E-05	0.000944	2142	- && Q9UT59.1 RecName: Full=Putative uncharacterized oxidoreductase C513.07 && PF01370:NAD dependent epimerase/dehydratase family
Lachesis_group0__13_contigs__l.g983	182.7704	759.2098	-2.05557	7.12E-06	0.000702	1497	- && O13724.1 RecName: Full=Zinc finger protein zpr1 && PF03367:ZPR1 zinc-finger domain
Lachesis_group0__13_contigs__l.g262	251.9857	1049.201	-2.0583	9.39E-06	0.000842	978	- && Q6C7A6.1 RecName: Full=Cytosolic Fe-S cluster assembly factor NBP35; AltName: Full=Nucleotide-binding protein 35 && PF10609:NUBPL iron-transfer P-loop NTPase

Lachesis_group0__13_contigs__l.g8965	326.7108	1364.147	-2.06238	5.18E-07	0.000123	3585	- && O60094.2 RecName: Full=DNA polymerase V; Short=POL V && PF04931:DNA polymerase phi
Lachesis_group0__13_contigs__l.g9128	70.01513	292.244	-2.06267	0.000129	0.00492	1182	- && Q601X8.2 RecName: Full=Chaperone protein DnaJ && PF00226:DnaJ domain
Lachesis_group0__13_contigs__l.g2446	36.26179	151.7329	-2.06731	2.14E-05	0.00141	1794	- && Q9UUA2.1 RecName: Full=ATP-dependent DNA helicase pfh1; AltName: Full=DNA repair and recombination helicase pfh1; AltName: Full=PIF1 helicase homolog; AltName: Full=RRM3/PIF1 homolog 1; Flags: Precursor && PF05970:PIF1-like helicase
Lachesis_group0__13_contigs__l.g5290	808.6992	3401.382	-2.07241	1.09E-06	0.000189	2961	- && Q01371.2 RecName: Full=White collar 1 protein; Short=WC1 && PF00320:GATA zinc finger
Lachesis_group0__13_contigs__l.g6821	302.8906	1279.034	-2.07832	1.01E-05	0.000882	3051	- && Q5AT15.1 RecName: Full=Serine O-acetyltransferase; Flags: Precursor && PF00561:alpha/beta hydrolase fold
Lachesis_group0__13_contigs__l.g5596	79.65616	338.0271	-2.08666	4.71E-06	0.000532	1395	- && O13632.1 RecName: Full=Tyrosine-protein phosphatase yvh1; Short=PTPase yvh1 && PF00782:Dual specificity phosphatase, catalytic domain
Lachesis_group0__13_contigs__l.g136	2617.958	11153.69	-2.091	0.001574	0.026446	1302	- && - && PF12937:F-box-like
Lachesis_group0__13_contigs__l.g11437	132.9858	567.0575	-2.09274	5.41E-06	0.000589	2559	- && Q9W032.1 RecName: Full=Protein ecdysoneless && PF07093:SGT1 protein
Lachesis_group0__13_contigs__l.g6400	59.94394	255.4221	-2.09444	2.62E-05	0.001594	990	- && P0CL97.1 RecName: Full=Pre-rRNA-processing protein ESF2; AltName: Full=18S rRNA factor 2 >P0CL96.1 RecName: Full=Pre-rRNA-processing protein ESF2; AltName: Full=18S rRNA factor 2 && -
Lachesis_group0__13_contigs__l.g2039	305.3677	1311.468	-2.10261	0.001416	0.025314	810	- && - && PF12697:Alpha/beta hydrolase family
Lachesis_group0__13_contigs__l.g10180	39.66938	170.2908	-2.10396	0.000338	0.009589	1005	- && - && PF09468:Ydr279p protein family (RNase H2 complex component)

Lachesis_group0__13_contigs__l.g4067	230.4895	995.7765	-2.11178	1.58E-06	0.000239	2370	- && O13956.1 RecName: Full=Ribosome biogenesis protein tsr1 && PF04950:40S ribosome biogenesis protein Tsr1 and BMS1 C-terminal|PF08142:AARP2CN (NUC121) domain
Lachesis_group0__13_contigs__l.g6442	448.9675	1945.736	-2.11585	3.31E-06	0.000408	1218	- && Q9C167.2 RecName: Full=Ribonucleoside-diphosphate reductase small chain; AltName: Full=Ribonucleotide reductase small subunit && PF00268:Ribonucleotide reductase, small chain
Lachesis_group0__13_contigs__l.g9457	398.5915	1739.448	-2.1257	1.36E-05	0.001016	3606	- && - && PF03177:Non-repetitive/WGA-negative nucleoporin C-terminal|PF08801:Nup133 N terminal like
Lachesis_group0__13_contigs__l.g6389	111.715	489.496	-2.13174	6.33E-05	0.003042	3042	- && Q8K2V6.1 RecName: Full=Importin-11; Short=Imp11; AltName: Full=Ran-binding protein 11; Short=RanBP11 && -
Lachesis_group0__13_contigs__l.g135	38.70529	170.7933	-2.14262	1.07E-05	0.000909	1794	- && - && PF00270:DEAD/DEAH box helicase
novel.1277	129.0368	574.8531	-2.15448	0.001534	0.026182	17215	- && P03875.2 RecName: Full=Putative COX1/OXI3 intron 1 protein && PF00961:LAGLIDADG endonuclease|PF01348:Type II intron maturase
Lachesis_group0__13_contigs__l.g11644	44.47048	197.6362	-2.15482	0.000125	0.004784	1089	- && O94449.1 RecName: Full=Replication factor C subunit 4; Short=Replication factor C4 && PF00004:ATPase family associated with various cellular activities (AAA)|PF08542:Replication factor C C-terminal domain
Lachesis_group0__13_contigs__l.g4744	44.99922	200.8213	-2.1589	0.000121	0.00475	2280	- && Q9BY08.1 RecName: Full=Emopamil-binding protein-like; AltName: Full=Emopamil-binding-related protein && PF05241:Emopamil binding protein
Lachesis_group0__13_contigs__l.g6385	32.03796	143.0045	-2.1597	0.000513	0.012604	1836	- && - && PF09507:DNA polymerase subunit Cdc27

Lachesis_group0__13_contigs__l.g6980	302.6942	1353.31	-2.16058	1.62E-08	1.17E-05	1572	- && Q0CJ61.1 RecName: Full=Efflux pump atB; AltName: Full=Terreic acid biosynthesis cluster protein B && PF07690:Major Facilitator Superfamily
Lachesis_group0__13_contigs__l.g6693	154.9458	693.5653	-2.16309	5.50E-07	0.000126	3240	- && O74429.1 RecName: Full=Inositol hexakisphosphate and diphosphoinositol-pentakisphosphate kinase; AltName: Full=Cortical actin cytoskeleton protein asp1; AltName: Full=InsP6 and PP-IP5 kinase && PF00328:Histidine phosphatase superfamily (branch 2)
Lachesis_group0__13_contigs__l.g5321	695.1523	3164.859	-2.18677	1.08E-05	0.000909	651	- && P42769.1 RecName: Full=Glutathione S-transferase PM239X14; AltName: Full=GST class-phi && PF02798:Glutathione S-transferase, N-terminal domain|PF00043:Glutathione S-transferase, C-terminal domain
Lachesis_group0__13_contigs__l.g3156	24.68658	112.1773	-2.18743	3.86E-06	0.000457	1335	- && Q6NU95.1 RecName: Full=RNA polymerase II-associated protein 3 && PF13414:TPR repeat
Lachesis_group0__13_contigs__l.g8861	281.0396	1281.95	-2.18974	2.39E-06	0.00032	1833	- && Q92372.1 RecName: Full=Replication factor A protein 1; AltName: Full=Single-stranded DNA-binding protein p68 subunit && PF01336:OB-fold nucleic acid binding domain|PF04057:Replication factor-A protein 1, N-terminal domain|PF08646:Replication factor-A C terminal domain|PF16900:Replication protein A OB domain
Lachesis_group0__13_contigs__l.g1337	71.99487	328.4986	-2.19017	0.00024	0.007703	855	- && O74628.1 RecName: Full=Uncharacterized oxidoreductase C162.03 && PF00106:short chain dehydrogenase
Lachesis_group0__13_contigs__l.g5207	48.13498	221.6625	-2.20239	0.000198	0.006685	954	- && Q5U209.1 RecName: Full=Ribonuclease H2 subunit A; Short=RNase H2 subunit A; AltName:

								Full=Ribonuclease HI large subunit; Short=RNase HI large subunit; AltName: Full=Ribonuclease HI subunit A && PF01351:Ribonuclease HII
Lachesis_group0__13_contigs__l.g470	1487.449	6892.205	-2.21211		0.000185	0.006463	1044	- && Q9UT59.1 RecName: Full=Putative uncharacterized oxidoreductase C513.07 && PF01370:NAD dependent epimerase/dehydratase family
Lachesis_group0__13_contigs__l.g7689	48.12602	223.7386	-2.2197		9.01E-06	0.00082	954	- && O94480.1 RecName: Full=25S rRNA (uridine-N(3))-methyltransferase && PF10354:Domain of unknown function (DUF2431)
Lachesis_group0__13_contigs__l.g8566	69.34078	325.9575	-2.23314		5.31E-06	0.000586	1047	- && - && PF07574:Nse1 non-SMC component of SMC5-6 complex|PF08746:RING-like domain
Lachesis_group0__13_contigs__l.g6690	123.1827	580.2773	-2.23471		3.65E-08	1.71E-05	1764	- && P94547.1 RecName: Full=Long-chain-fatty-acid--CoA ligase; AltName: Full=Long-chain acyl-CoA synthetase && PF13193:AMP-binding enzyme C-terminal domain|PF00501:AMP-binding enzyme
Lachesis_group0__13_contigs__l.g9498	61.908	292.0583	-2.23648		5.71E-07	0.000127	2595	- && Q0CAF5.1 RecName: Full=Probable beta-glucosidase I; AltName: Full=Beta-D-glucoside glucohydrolase I; AltName: Full=Cellobiase I; AltName: Full=Gentiobiase I && PF01915:Glycosyl hydrolase family 3 C-terminal domain|PF14310:Fibronectin type III-like domain|PF07691:PA14 domain|PF00933:Glycosyl hydrolase family 3 N terminal domain
Lachesis_group0__13_contigs__l.g1572	156.2146	738.4438	-2.242		3.34E-08	1.65E-05	1032	- && Q9Y6F1.3 RecName: Full=Poly [ADP-ribose] polymerase 3; Short=PARP-3; Short=hPARP-3; AltName: Full=ADP-ribosyltransferase diphtheria toxin-like 3; Short=ARTD3; AltName: Full=IRT1;

							AltName: Full=NAD(+) ADP-ribosyltransferase 3; Short=ADPRT-3; AltName: Full=Poly[ADP-ribose] synthase 3; Short=pADPRT-3 && PF00644:Poly(ADP-ribose) polymerase catalytic domain
Lachesis_group0__13_contigs__l.g6805	29.96637	142.9605	-2.25948	9.81E-05	0.004066	906	- && - && PF08167:rRNA processing/ribosome biogenesis
Lachesis_group0__13_contigs__l.g6392	165.8049	797.1579	-2.26527	0.000422	0.011039	1167	- && O13991.3 RecName: Full=Uncharacterized oxidoreductase C26H5.09c && PF01408:Oxidoreductase family, NAD-binding Rossmann fold
Lachesis_group0__13_contigs__l.g5558	252.1217	1220.835	-2.27608	3.65E-07	9.77E-05	2619	- && Q9C1X1.1 RecName: Full=Periodic tryptophan protein 2 homolog && PF00400:WD domain, G-beta repeat|PF04003:Dip2/Utp12 Family
Lachesis_group0__13_contigs__l.g10926	22.30134	108.968	-2.29463	4.95E-05	0.002538	1524	- && P36146.1 RecName: Full=Protein LAS1 && PF04031:Las1-like
Lachesis_group0__13_contigs__l.g4760	55.73506	277.4413	-2.31456	0.001676	0.027257	903	- && O74628.1 RecName: Full=Uncharacterized oxidoreductase C162.03 && PF00106:short chain dehydrogenase
Lachesis_group0__13_contigs__l.g4468	214.09	1087.556	-2.34531	7.33E-07	0.000143	1083	- && O13648.3 RecName: Full=Ribosomal protein arginine N-methyltransferase rmt3 && PF06325:Ribosomal protein L11 methyltransferase (PrmA)
Lachesis_group0__13_contigs__l.g3663	81.75315	415.3968	-2.34579	1.24E-05	0.000945	831	- && D4AK18.2 RecName: Full=Uncharacterized secreted protein ARB_06907; Flags: Precursor && -
Lachesis_group0__13_contigs__l.g5251	37.10791	189.6808	-2.35291	0.000158	0.005761	1077	- && Q8X082.1 RecName: Full=Replication factor C subunit 5; Short=Replication factor C5; AltName: Full=Probable activator 1 subunit 5 &&

							PF08542:Replication factor C C-terminal domain|PF13177:DNA polymerase III, delta subunit
Lachesis_group0__13_contigs__l.g2881	22.55151	116.3042	-2.3716	2.86E-06	0.000373	1287	- && P40481.1 RecName: Full=Histidine protein methyltransferase 1; AltName: Full=Mitotic exit network interactor 1 && -
Lachesis_group0__13_contigs__l.g5709	26.89931	144.57	-2.42454	1.78E-05	0.001247	2832	- && - && PF00533:BRCA1 C Terminus (BRCT) domain|PF12738:twin BRCT domain
Lachesis_group0__13_contigs__l.g3189	46.64588	251.9903	-2.43634	8.69E-07	0.000156	1437	- && B5FXE5.1 RecName: Full=Apoptosis-inducing factor 2 && PF07992:Pyridine nucleotide-disulphide oxidoreductase
Lachesis_group0__13_contigs__l.g4640	114.1742	629.8872	-2.46535	7.74E-07	0.000145	1881	- && - && PF12937:F-box-like
Lachesis_group0__13_contigs__l.g10399	16.08956	89.41688	-2.47658	0.001628	0.026843	1371	- && Q06816.2 RecName: Full=Putative epoxide hydrolase; AltName: Full=Epoxide hydratase; Flags: Precursor && PF06441:Epoxide hydrolase N terminus
Lachesis_group0__13_contigs__l.g4098	607.0298	3450.466	-2.50692	2.34E-05	0.001488	1161	- && Q7SDV9.1 RecName: Full=Cytochrome c peroxidase, mitochondrial; Short=CCP; Flags: Precursor && PF00141:Peroxidase
Lachesis_group0__13_contigs__l.g201	189.1413	1120.864	-2.56693	1.59E-05	0.001136	1947	- && - && PF08618:Transcription factor Opi1
Lachesis_group0__13_contigs__l.g5623	254.2291	1528.701	-2.58803	1.11E-05	0.000919	3048	- && Q01371.2 RecName: Full=White collar 1 protein; Short=WC1 && PF08447:PAS fold|PF13426:PAS domain
Lachesis_group0__13_contigs__l.g1463	48.77124	293.6813	-2.59116	2.47E-08	1.45E-05	2145	- && O94564.1 RecName: Full=Zinc-type alcohol dehydrogenase-like protein C1773.06c && PF00107:Zinc-binding dehydrogenase|PF08240:Alcohol dehydrogenase GroES-like domain

Lachesis_group0__13_contigs__l.g4398	77.08646	466.3308	-2.59732	2.86E-08	1.49E-05	1752	- && D7PHY8.1 RecName: Full=Efflux pump vrtL; AltName: Full=Viridicatumtoxin synthesis protein L && PF07690:Major Facilitator Superfamily
Lachesis_group0__13_contigs__l.g5348	72.70581	441.4284	-2.60217	4.85E-08	1.89E-05	3462	- && Q06680.2 RecName: Full=Condensin complex subunit 3; AltName: Full=CAPG homolog && PF13646:HEAT repeats|PF12719:Nuclear condensing complex subunits, C-term domain
Lachesis_group0__13_contigs__l.g1944	548.5339	3339.488	-2.60594	6.93E-07	0.000138	1350	- && Q55DV9.1 RecName: Full=Cystathionine gamma-lyase; AltName: Full=Gamma-cystathionase && PF01053:Cys/Met metabolism PLP-dependent enzyme
Lachesis_group0__13_contigs__l.g1338	67.46262	411.928	-2.61026	2.03E-05	0.001366	855	- && O74628.1 RecName: Full=Uncharacterized oxidoreductase C162.03 && PF00106:short chain dehydrogenase
Lachesis_group0__13_contigs__l.g4765	164.3636	1005.293	-2.61333	8.47E-06	0.000778	1059	- && Q8K2T1.1 RecName: Full=NmrA-like family domain-containing protein 1 && PF05368:NmrA-like family
Lachesis_group0__13_contigs__l.g10188	38.06753	240.5988	-2.65932	5.88E-08	2.12E-05	1290	- && Q5BK68.1 RecName: Full=snRNA-activating protein complex subunit 3; Short=SNAPc subunit 3; AltName: Full=Small nuclear RNA-activating complex polypeptide 3 && PF12251:snRNA-activating protein of 50kDa MW C terminal
Lachesis_group0__13_contigs__l.g9938	1463.005	9261.775	-2.66231	9.21E-10	1.13E-06	3204	- && Q9Y8G7.1 RecName: Full=Bifunctional cytochrome P450/NADPH--P450 reductase; AltName: Full=Cytochrome P450foxy; AltName: Full=Fatty acid omega-hydroxylase; AltName: Full=P450foxy; Includes: RecName: Full=Cytochrome P450 505; Includes: RecName: Full=NADPH--cytochrome P450 reductase && PF00067:Cytochrome

							P450|PF00258:Flavodoxin|PF00667:FAD binding domain|PF00175:Oxidoreductase NAD-binding domain
Lachesis_group0__13_contigs__l.g6847	321.2773	2094.006	-2.7043	9.76E-06	0.000863	771	- && Q12335.1 RecName: Full=Protoplast secreted protein 2; Flags: Precursor && PF03358:NADPH-dependent FMN reductase
Lachesis_group0__13_contigs__l.g10956	40.89337	269.4765	-2.71881	4.02E-08	1.73E-05	1035	- && - && PF06775:Putative adipose-regulatory protein (Seipin)
Lachesis_group0__13_contigs__l.g359	18.33066	120.4386	-2.72514	3.14E-06	0.000398	960	- && O59952.1 RecName: Full=Lipase; AltName: Full=Triacylglycerol lipase; Flags: Precursor && PF01764:Lipase (class 3)
Lachesis_group0__13_contigs__l.g6519	9339.331	63585.87	-2.76731	5.44E-08	2.04E-05	1803	- && Q09653.3 RecName: Full=Putative cytochrome P450 CYP13A10 && PF00067:Cytochrome P450
Lachesis_group0__13_contigs__l.g1020	12.73573	87.45131	-2.78412	1.53E-05	0.001111	924	- && Q9UT28.1 RecName: Full=Protein N-methyltransferase nnt1; AltName: Full=Nicotinamide N-methyltransferase-like protein 1 && PF10294:Lysine methyltransferase
Lachesis_group0__13_contigs__l.g1968	824.4631	6036.214	-2.87218	9.90E-08	3.44E-05	1593	- && G3Y416.1 RecName: Full=Cytochrome P450 monooxygenase yanC; AltName: Full=Yanuthone D synthesis protein C; Flags: Precursor && PF00067:Cytochrome P450
Lachesis_group0__13_contigs__l.g1398	288.0803	2152.149	-2.90121	1.45E-08	1.13E-05	1689	- && S0EEY7.1 RecName: Full=Efflux pump FUS6; AltName: Full=Fusarin biosynthesis protein 6 && PF07690:Major Facilitator Superfamily
Lachesis_group0__13_contigs__l.g5326	128.2616	998.0624	-2.95981	2.57E-09	2.41E-06	1788	- && Q870L3.1 RecName: Full=Siderophore iron transporter mirC; AltName: Full=Major facilitator iron-regulated transporter C && -

Lachesis_group0__13_contigs__l.g5502	31.84006	252.309	-2.99116	2.70E-08	1.49E-05	1629	- && Q09887.1 RecName: Full=Uncharacterized amino-acid permease C584.13 && PF13520:Amino acid permease
Lachesis_group0__13_contigs__l.g1462	210.5674	1881.944	-3.15973	7.44E-09	6.34E-06	1110	- && O94564.1 RecName: Full=Zinc-type alcohol dehydrogenase-like protein C1773.06c && PF00107:Zinc-binding dehydrogenase
Lachesis_group0__13_contigs__l.g10724	379.8038	3521.268	-3.21282	1.81E-07	5.48E-05	1872	- && P11182.3 RecName: Full=Lipoamide acyltransferase component of branched-chain alpha-keto acid dehydrogenase complex, mitochondrial; AltName: Full=52 kDa mitochondrial autoantigen of primary biliary cirrhosis; AltName: Full=Branched chain 2-oxo-acid dehydrogenase complex component E2; Short=BCOADC-E2; AltName: Full=Branched-chain alpha-keto acid dehydrogenase complex component E2; Short=BCKAD-E2; Short=BCKADE2; AltName: Full=Dihydrolipoamide acetyltransferase component of branched-chain alpha-keto acid dehydrogenase complex; AltName: Full=Dihydrolipoamide branched chain transacylase; AltName: Full=Dihydrolipoyllysine-residue (2-methylpropanoyl)transferase; Flags: Precursor && PF00364:Biotin-requiring enzyme|PF00198:2-oxoacid dehydrogenases acyltransferase (catalytic domain)|PF02817:e3 binding domain
Lachesis_group0__13_contigs__l.g344	179.7168	1670.468	-3.21732	1.03E-09	1.13E-06	1665	- && P53388.1 RecName: Full=Dicarboxylic amino acid permease && PF00324:Amino acid permease
Lachesis_group0__13_contigs__l.g7108	19.00725	179.0706	-3.23712	1.17E-06	0.000199	1278	- && P41816.2 RecName: Full=NADPH dehydrogenase 3; AltName: Full=Old yellow enzyme 3 &&

							PF00724:NADH:flavin oxidoreductase / NADH oxidase family
Lachesis_group0__13_contigs__l.g10002	35.99294	345.5829	-3.26772	3.95E-07	0.0001	1059	- && A8DRH7.1 RecName: Full=L-threo-3-deoxy-hexylosonate aldolase; AltName: Full=L-threo-3-deoxy-hexulosonate aldolase && PF00701:Dihydrodipicolinate synthetase family
Lachesis_group0__13_contigs__l.g9890	46.26875	479.7935	-3.3739	1.60E-11	5.00E-08	1824	- && Q9C0V0.1 RecName: Full=Probable amino-acid permease PB1C11.02 && PF00324:Amino acid permease
Lachesis_group0__13_contigs__l.g9594	49.10656	825.2781	-4.07103	2.16E-08	1.44E-05	1008	- && Q5XGR8.1 RecName: Full=Endoribonuclease LACTB2; AltName: Full=Beta-lactamase-like protein 2 && PF00753:Metallo-beta-lactamase superfamily

Log_2Fold change: values of transcripts upregulated in BaP and Brij 30 addition is represented by positive numbers and downregulated is represented by negative numbers.

Padj: adjust *P*-value

Table S4 Expression of genes in the Brij30_BaP and NoBaP comparisons

gene_id	Brij30_BaP	NoBaP	log_2FoldChange	*P*value	padj	gene_length	gene_description
Lachesis_group0__13_contigs__l.g8359	1726.933	24.88162	6.122587	2.09E-28	7.30E-25	789	- && Q91XV4.1 RecName: Full=L-xylulose reductase; Short=XR; AltName: Full=Dicarbonyl/L-xylulose reductase; AltName: Full=Sperm antigen P26h && PF00106:short chain dehydrogenase
Lachesis_group0__13_contigs__l.g8360	432.3769	10.37399	5.365414	4.76E-17	4.13E-14	699	- && Q6NUE2.1 RecName: Full=Carbonyl reductase family member 4; AltName: Full=3-ketoacyl-[acyl-carrier-protein] reductase beta subunit; Short=KAR beta subunit; AltName: Full=3-oxoacyl-[acyl-carrier-protein] reductase; AltName: Full=Quinone reductase CBR4 && PF00106:short chain dehydrogenase
Lachesis_group0__13_contigs__l.g2225	3278.043	80.51738	5.344839	3.78E-17	3.59E-14	1257	- && Q01752.1 RecName: Full=Aryl-alcohol dehydrogenase [NADP(+)]; Short=AAD && PF00248:Aldo/keto reductase family
Lachesis_group0__13_contigs__l.g7729	1622.787	61.01763	4.730985	5.76E-21	1.00E-17	1071	- && G3XMC6.1 RecName: Full=Dehydrogenase azaJ; AltName: Full=Azaphilone biosynthesis cluster protein azaJ && PF08240:Alcohol dehydrogenase GroES-like domain|PF00107:Zinc-binding dehydrogenase
Lachesis_group0__13_contigs__l.g2647	1495.671	79.94657	4.223604	1.69E-11	6.08E-09	1212	- && Q01752.1 RecName: Full=Aryl-alcohol dehydrogenase [NADP(+)]; Short=AAD && PF00248:Aldo/keto reductase family

Lachesis_group0__13_contigs__l.g9363	36.06895	2.014527	4.108947	2.52E-05	0.000916	1479	- && O69763.1 RecName: Full=Vanillin dehydrogenase && PF00171:Aldehyde dehydrogenase family
Lachesis_group0__13_contigs__l.g8361	50.19522	3.463245	3.835509	4.54E-05	0.001421	1023	- && - && PF01040:UbiA prenyltransferase family
Lachesis_group0__13_contigs__l.g3707	2729.876	207.452	3.717506	6.94E-06	0.00033	1305	- && Q70LM3.1 RecName: Full=Manganese peroxidase 2; Short=MnP2; AltName: Full=Manganese peroxidase isozyme 2; Flags: Precursor && PF00141:Peroxidase|PF11895:Domain of unknown function (DUF3415)
Lachesis_group0__13_contigs__l.g3514	3311.474	266.9033	3.633228	2.13E-09	4.27E-07	1473	- && Q6UEF1.1 RecName: Full=Oxidoreductase AflY; AltName: Full=Aflatoxin biosynthesis protein Y && PF14027:Protein of unknown function (DUF4243)
Lachesis_group0__13_contigs__l.g794	4455.696	445.4461	3.322953	2.70E-07	2.45E-05	1977	- && - && PF00004:ATPase family associated with various cellular activities (AAA)
Lachesis_group0__13_contigs__l.g6791	4529.458	490.1738	3.207721	1.54E-06	9.67E-05	513	- && Q9JIM4.1 RecName: Full=Dual specificity protein phosphatase 12; AltName: Full=Glucokinase-associated dual specificity phosphatase; Short=GKAP && PF00782:Dual specificity phosphatase, catalytic domain
Lachesis_group0__13_contigs__l.g4699	20349.98	2217.834	3.197853	1.80E-10	4.59E-08	1041	- && - && PF00702:haloacid dehalogenase-like hydrolase
Lachesis_group0__13_contigs__l.g4508	205.8208	23.09061	3.166602	1.96E-06	0.00012	903	- && - && PF05368:NmrA-like family
Lachesis_group0__13_contigs__l.g8358	974.4781	116.7031	3.058828	2.79E-09	5.34E-07	1050	- && Q91XV4.1 RecName: Full=L-xylulose reductase; Short=XR; AltName: Full=Dicarbonyl/L-

							xylulose reductase; AltName: Full=Sperm antigen P26h && PF00106:short chain dehydrogenase
Lachesis_group0__13_contigs__l.g768	12812.97	1633.442	2.971768	8.42E-06	0.000381	1986	- && - && PF00004:ATPase family associated with various cellular activities (AAA)
Lachesis_group0__13_contigs__l.g4840	2015.461	262.4409	2.941662	2.94E-11	9.92E-09	1068	- && Q8FKG1.1 RecName: Full=S-(hydroxymethyl)glutathione dehydrogenase; AltName: Full=Alcohol dehydrogenase class-3; AltName: Full=Alcohol dehydrogenase class-III; AltName: Full=Glutathione-dependent formaldehyde dehydrogenase; Short=FALDH; Short=FDH; Short=GSH-FDH && PF00107:Zinc-binding dehydrogenase|PF08240:Alcohol dehydrogenase GroES-like domain
Lachesis_group0__13_contigs__l.g535	1260.453	168.7143	2.900208	3.86E-08	5.11E-06	1350	- && P23262.4 RecName: Full=Salicylate hydroxylase; AltName: Full=Salicylate 1-monooxygenase && PF01494:FAD binding domain
Lachesis_group0__13_contigs__l.g4839	861.4914	115.7918	2.895459	9.38E-08	1.04E-05	1125	- && Q2HEW4.1 RecName: Full=Enoyl reductase CHGG_01240; AltName: Full=Chaetoglobosin biosynthesis protein CHGG_01240 && PF08240:Alcohol dehydrogenase GroES-like domain|PF00107:Zinc-binding dehydrogenase
Lachesis_group0__13_contigs__l.g4741	116.7205	16.27497	2.837363	1.18E-07	1.28E-05	1152	- && - && PF00326:Prolyl oligopeptidase family|PF07859:alpha/beta hydrolase fold
Lachesis_group0__13_contigs__l.g2226	1519.396	216.3284	2.813125	3.22E-06	0.000181	1158	- && Q01752.1 RecName: Full=Aryl-alcohol dehydrogenase [NADP(+)]; Short=AAD && PF00248:Aldo/keto reductase family

Lachesis_group0__13_contigs__l.g9965	945.8727	138.9837	2.767307	4.64E-12	1.86E-09	1008	- && Q09923.1 RecName: Full=Aldo-keto reductase yakc [NADP(+)] && PF00248:Aldo/keto reductase family
Lachesis_group0__13_contigs__l.g10392	443.6222	65.67012	2.752369	7.67E-05	0.002137	3291	- && Q04336.1 RecName: Full=Uncharacterized protein YMR196W && PF03200:Glycosyl hydrolase family 63 C-terminal domain
Lachesis_group0__13_contigs__l.g7939	179.9171	27.86692	2.697168	8.15E-05	0.002242	1812	- && - && PF15275:PEHE domain
Lachesis_group0__13_contigs__l.g3942	40.66893	6.274201	2.685897	0.004199	0.03936	1599	- && - && PF12937:F-box-like
Lachesis_group0__13_contigs__l.g10103	634.6469	99.05187	2.678008	9.72E-05	0.002552	1041	- && Q96NR8.3 RecName: Full=Retinol dehydrogenase 12; AltName: Full=All-trans and 9-cis retinol dehydrogenase; AltName: Full=Short chain dehydrogenase/reductase family 7C member 2 && PF00106:short chain dehydrogenase
Lachesis_group0__13_contigs__l.g11522	55.2368	8.566259	2.668078	0.005705	0.048918	2631	- && Q96Q89.3 RecName: Full=Kinesin-like protein KIF20B; AltName: Full=Cancer/testis antigen 90; Short=CT90; AltName: Full=Kinesin family member 20B; AltName: Full=Kinesin-related motor interacting with PIN1; AltName: Full=M-phase phosphoprotein 1; Short=MPP1 && PF00225:Kinesin motor domain
Lachesis_group0__13_contigs__l.g1269	52.78292	8.387732	2.658844	0.002254	0.02437	954	- && O74959.1 RecName: Full=Uncharacterized oxidoreductase C736.13 && PF00106:short chain dehydrogenase
Lachesis_group0__13_contigs__l.g4879	14259.95	2275.556	2.647785	4.06E-07	3.37E-05	738	- && O94476.1 RecName: Full=Eukaryotic translation initiation factor 6; Short=eIF-6 && PF01912:eIF-6 family

Lachesis_group0__13_contigs__l.g8469	544.7839	90.69088	2.584224	1.40E-08	2.12E-06	2790	- && Q9Y804.1 RecName: Full=Fanconi-associated nuclease 1 homolog && PF08774:VRR-NUC domain
Lachesis_group0__13_contigs__l.g5967	14301.78	2464.648	2.536859	1.33E-05	0.000548	474	- && - && PF01185:Fungal hydrophobin
Lachesis_group0__13_contigs__l.g2537	216.6505	37.54767	2.526825	2.49E-05	0.000912	1293	- && P59668.1 RecName: Full=Delta(12) fatty acid desaturase; AltName: Full=Delta-12 fatty acid desaturase && PF00487:Fatty acid desaturase
Lachesis_group0__13_contigs__l.g6383	36.86763	6.42738	2.49802	0.004771	0.043106	1746	- && - && PF12937:F-box-like
Lachesis_group0__13_contigs__l.g4696	8273.53	1487.498	2.475742	4.45E-07	3.58E-05	798	- && O23522.2 RecName: Full=Phospholipase A1-Ibeta2, chloroplastic; Flags: Precursor && PF01764:Lipase (class 3)
Lachesis_group0__13_contigs__l.g10095	7631.37	1407.814	2.438541	9.68E-11	2.73E-08	5748	- && P78811.2 RecName: Full=Probable UTP--glucose-1-phosphate uridylyltransferase; AltName: Full=UDP-glucose pyrophosphorylase; Short=UDPGP; Short=UGPase && PF08797:HIRAN domain|PF00176:SNF2 family N-terminal domain|PF00645:Poly(ADP-ribose) polymerase and DNA-Ligase Zn-finger region|PF01704:UTP--glucose-1-phosphate uridylyltransferase|PF00271:Helicase conserved C-terminal domain
Lachesis_group0__13_contigs__l.g9037	299.6363	55.241	2.437353	0.000172	0.003788	1494	- && P0CS57.1 RecName: Full=DNA damage-binding protein CMR1 >P0CS56.1 RecName: Full=DNA damage-binding protein CMR1 && -
Lachesis_group0__13_contigs__l.g4605	57.9835	10.78258	2.410028	0.003564	0.034652	1251	- && Q24168.2 RecName: Full=Origin recognition complex subunit 2; Short=DmORC2 && PF04084:Origin recognition complex subunit 2

Lachesis_group0__13_contigs__l.g6373	1120.956	212.3572	2.401441	2.47E-06	0.000146	1047	- && O14295.1 RecName: Full=Pyridoxal reductase; Short=PL reductase; Short=PL-red && PF00248:Aldo/keto reductase family
Lachesis_group0__13_contigs__l.g4688	620.0326	119.6904	2.374734	7.15E-09	1.17E-06	822	- && Q96DG6.1 RecName: Full=Carboxymethylenebutenolidase homolog && PF01738:Dienelactone hydrolase family
Lachesis_group0__13_contigs__l.g737	123.9889	24.10692	2.366945	0.003377	0.033304	867	- && - && PF07264:Etoposide-induced protein 2.4 (EI24)
Lachesis_group0__13_contigs__l.g6455	1077.361	212.7184	2.340049	1.86E-07	1.88E-05	1674	- && - && PF02458:Transferase family
Lachesis_group0__13_contigs__l.g1802	927.5551	184.7381	2.327627	2.85E-05	0.001001	1833	- && Q9U9A3.2 RecName: Full=Serine/threonine-protein phosphatase 6 catalytic subunit; Short=PP6C && PF00248:Aldo/keto reductase family
Lachesis_group0__13_contigs__l.g9876	1526.854	307.7247	2.310997	0.000484	0.008326	1125	- && - && PF01040:UbiA prenyltransferase family
Lachesis_group0__13_contigs__l.g1959	2509.306	511.2431	2.295405	9.93E-05	0.002578	873	- && - && PF13419:Haloacid dehalogenase-like hydrolase
Lachesis_group0__13_contigs__l.g2849	62.51274	12.82747	2.283704	0.002323	0.024979	1881	- && - && PF12937:F-box-like
Lachesis_group0__13_contigs__l.g5006	118.8948	24.59447	2.267691	1.23E-05	0.000513	825	- && - && PF01138:3' exoribonuclease family, domain 1
Lachesis_group0__13_contigs__l.g829	540.2873	112.4939	2.262236	2.89E-05	0.001011	1440	- && - && PF01738:Dienelactone hydrolase family
Lachesis_group0__13_contigs__l.g7345	78.64017	16.49541	2.241323	0.004561	0.041714	336	- && - && PF07019:Rab5-interacting protein (Rab5ip)
Lachesis_group0__13_contigs__l.g2505	5680.178	1241.807	2.193293	0.000104	0.00263	1947	- && - && PF01565:FAD binding domain
Lachesis_group0__13_contigs__l.g5553	4706.171	1041.065	2.176335	0.000327	0.006273	1230	- && Q00859.1 RecName: Full=Mitogen-activated protein kinase; AltName: Full=FsMAPK && PF00069:Protein kinase domain

Lachesis_group0__13_contigs__l.g10646	55.37787	12.43447	2.15249	0.001446	0.017806	750	- && B6JGU7.1 RecName: Full=ATP-dependent Clp protease proteolytic subunit; AltName: Full=Endopeptidase Clp && PF00574:Clp protease
Lachesis_group0__13_contigs__l.g2817	49.16137	11.181	2.139697	0.000666	0.010376	2400	- && - && PF04082:Fungal specific transcription factor domain
Lachesis_group0__13_contigs__l.g486	52080.77	12210.91	2.092596	0.000214	0.00453	1758	- && Q54DY9.1 RecName: Full=Probable mitochondrial chaperone BCS1-B; AltName: Full=BCS1-like protein 2 && PF08740:BCS1 N terminal|PF00004:ATPase family associated with various cellular activities (AAA)
Lachesis_group0__13_contigs__l.g5973	5254.939	1243.02	2.080091	8.49E-05	0.002305	3084	- && Q9ZVH4.1 RecName: Full=Cullin-3A; Short=AtCUL3a && PF13417:Glutathione S-transferase, N-terminal domain|PF00888:Cullin family|PF10557:Cullin protein neddylation domain
Lachesis_group0__13_contigs__l.g3748	7297.25	1736.817	2.070723	2.30E-05	0.000862	3198	- && D4AUF1.1 RecName: Full=WSC domain-containing protein ARB_07867; Flags: Precursor && PF01822:WSC domain|PF07250:Glyoxal oxidase N-terminus|PF09118:Domain of unknown function (DUF1929)
Lachesis_group0__13_contigs__l.g1086	1122.422	267.6793	2.069163	3.61E-06	0.000197	2319	- && Q80Z60.2 RecName: Full=Endothelin-converting enzyme 2; Short=ECE-2; Includes: RecName: Full=Methyltransferase-like region; Includes: RecName: Full=Endothelin-converting enzyme 2 region && PF08241:Methyltransferase domain

Lachesis_group0__13_contigs__l.g3306	58.26505	14.00964	2.05699	0.00057	0.009377	306	- && Q4P8G2.1 RecName: Full=Diphthamide biosynthesis protein 3 && PF05207:CSL zinc finger
Lachesis_group0__13_contigs__l.g9807	1022.162	247.5099	2.046931	2.76E-06	0.000158	1260	- && - && PF14976:FAM72 protein
Lachesis_group0__13_contigs__l.g1758	1033.572	253.7354	2.02573	7.56E-06	0.000351	1032	- && Q09923.1 RecName: Full=Aldo-keto reductase yakc [NADP(+)] && PF00248:Aldo/keto reductase family
Lachesis_group0__13_contigs__l.g1803	375.2914	92.93757	2.011814	1.34E-06	8.79E-05	1038	- && B9WYE6.1 RecName: Full=Versiconal hemiacetal acetate reductase; AltName: Full=VHA reductase && PF00248:Aldo/keto reductase family
Lachesis_group0__13_contigs__l.g152	430.0907	106.9808	2.010327	4.44E-05	0.001406	1329	- && Q08980.1 RecName: Full=Probable transport protein YPL264C && PF00892:EamA-like transporter family
Lachesis_group0__13_contigs__l.g7222	4854.499	1221.51	1.990881	0.005208	0.045905	381	- && P16934.1 RecName: Full=Fruiting body protein SC4; AltName: Full=Hydrophobin SC4; Flags: Precursor && PF01185:Fungal hydrophobin
Lachesis_group0__13_contigs__l.g10100	4187.574	1054.561	1.989448	0.00364	0.035332	1488	- && Q9LK73.1 RecName: Full=UDP-glycosyltransferase 88A1 && PF00201:UDP-glucoronosyl and UDP-glucosyl transferase
Lachesis_group0__13_contigs__l.g6869	131.7109	33.66374	1.974883	0.000418	0.007523	978	- && Q7XA87.1 RecName: Full=Folate transporter 1, chloroplastic; Short=AtFOLT1 && PF00153:Mitochondrial carrier protein
Lachesis_group0__13_contigs__l.g6629	1294.661	330.1164	1.971978	1.93E-07	1.92E-05	483	- && O74866.1 RecName: Full=Riboflavin kinase; AltName: Full=ATP:riboflavin 5'-phosphotransferase; AltName: Full=Flavin mononucleotide kinase 1; AltName: Full=Flavokinase && PF01687:Riboflavin kinase

Lachesis_group0__13_contigs__l.g2190	2877.781	737.255	1.965207	0.000342	0.006464	747	- && - && PF04140:Isoprenylcysteine carboxyl methyltransferase (ICMT) family
Lachesis_group0__13_contigs__l.g27	125.0362	32.02257	1.959621	0.000578	0.009477	918	- && - && PF06221:Putative zinc finger motif, C2HC5-type
Lachesis_group0__13_contigs__l.g2089	661.6419	171.3743	1.949725	0.000124	0.002991	570	- && B0D4L0.1 RecName: Full=Signal peptidase complex catalytic subunit SEC11; AltName: Full=Signal peptidase I && PF00717:Peptidase S24-like
Lachesis_group0__13_contigs__l.g8088	219.7211	57.71943	1.925937	8.07E-05	0.002226	1734	- && O94725.1 RecName: Full=Protein-glutamate O-methyltransferase C1393.13 && PF01937:Protein of unknown function DUF89
Lachesis_group0__13_contigs__l.g11288	220.4832	58.17745	1.921254	4.35E-06	0.000229	681	- && Q8VY97.1 RecName: Full=ER membrane protein complex subunit 7 homolog; Flags: Precursor && PF09430:Protein of unknown function (DUF2012)
Lachesis_group0__13_contigs__l.g8052	329.0748	87.02061	1.918427	0.002976	0.030315	2613	- && - && PF12937:F-box-like
Lachesis_group0__13_contigs__l.g4387	765.8853	202.7471	1.917682	1.45E-07	1.55E-05	2463	- && Q5F389.2 RecName: Full=WW domain-containing oxidoreductase && PF00106:short chain dehydrogenase
Lachesis_group0__13_contigs__l.g9875	12825.37	3414.239	1.90946	0.000356	0.00664	1671	- && P64264.1 RecName: Full=Uncharacterized GMC-type oxidoreductase Mb1310 >P9WMV4.1 RecName: Full=Uncharacterized GMC-type oxidoreductase MT1316 >P9WMV5.1 RecName: Full=Uncharacterized GMC-type oxidoreductase Rv1279 && PF00732:GMC oxidoreductase|PF05199:GMC oxidoreductase

Lachesis_group0__13_contigs__l.g2166	12661.91	3383.449	1.903891	2.68E-05	0.000959	1437	- && Q12618.1 RecName: Full=Acyl-CoA desaturase; AltName: Full=Delta(9)-desaturase; Short=Delta-9 desaturase; AltName: Full=Fatty acid desaturase; AltName: Full=Stearoyl-CoA desaturase && PF00487:Fatty acid desaturase|PF00173:Cytochrome b5-like Heme/Steroid binding domain
Lachesis_group0__13_contigs__l.g10381	186.7591	50.2668	1.896207	0.000979	0.013778	1665	- && - && PF07535:DBF zinc finger|PF08630:Dfp1/Him1, central region
Lachesis_group0__13_contigs__l.g4951	89.92738	24.08688	1.893885	0.001362	0.017029	690	- && - && PF09725:Folate-sensitive fragile site protein Fra10Ac1
Lachesis_group0__13_contigs__l.g10628	1243.762	337.3371	1.88193	0.000836	0.012309	1728	- && - && PF00026:Eukaryotic aspartyl protease
Lachesis_group0__13_contigs__l.g4768	14819.08	4095.076	1.855553	3.60E-06	0.000197	1335	- && P59668.1 RecName: Full=Delta(12) fatty acid desaturase; AltName: Full=Delta-12 fatty acid desaturase && PF00487:Fatty acid desaturase
Lachesis_group0__13_contigs__l.g3389	650.1304	180.0676	1.85061	7.65E-05	0.002137	1875	- && O94528.1 RecName: Full=Caffeine resistance protein 5 && PF07690:Major Facilitator Superfamily
Lachesis_group0__13_contigs__l.g637	190.3496	52.99143	1.848165	0.000104	0.002628	291	- && - && PF10270:Membrane magnesium transporter
Lachesis_group0__13_contigs__l.g7233	320.0375	89.49748	1.835882	0.001658	0.019514	477	- && Q9UTI3.1 RecName: Full=N-terminal acetyltransferase A complex catalytic subunit ard1; Short=NatA complex subunit ARD1 && PF00583:Acetyltransferase (GNAT) family
Lachesis_group0__13_contigs__l.g6036	382.4533	107.5154	1.831006	1.90E-05	0.000737	3510	- && P28706.2 RecName: Full=DNA repair protein rad13 && PF00752:XPG N-terminal domain|PF00867:XPG I-region

Lachesis_group0__13_contigs__l.g8732	419.5861	118.2812	1.825608	0.000461	0.008023	1620	- && Q04792.1 RecName: Full=Glutamate decarboxylase; Short=GAD && PF00282:Pyridoxal-dependent decarboxylase conserved domain
Lachesis_group0__13_contigs__l.g6559	620.9238	175.6802	1.820771	0.000246	0.005127	1413	- && Q9V345.1 RecName: Full=COP9 signalosome complex subunit 4; Short=Dch4; Short=Signalosome subunit 4 && PF01399:PCI domain
Lachesis_group0__13_contigs__l.g7	19154.05	5436.266	1.816978	0.00248	0.026342	1914	- && - && PF00320:GATA zinc finger
Lachesis_group0__13_contigs__l.g2552	527.8103	150.7904	1.807093	3.75E-06	0.000202	1320	- && Q8VYR0.1 RecName: Full=Calcium uniporter protein 5, mitochondrial; Flags: Precursor && PF04678:Mitochondrial calcium uniporter
Lachesis_group0__13_contigs__l.g2237	299.4691	85.48982	1.805799	0.000793	0.011786	1740	- && - && PF12937:F-box-like
Lachesis_group0__13_contigs__l.g10104	289.3798	83.02634	1.79989	0.005269	0.046211	1041	- && Q8TC12.2 RecName: Full=Retinol dehydrogenase 11; AltName: Full=Androgen-regulated short-chain dehydrogenase/reductase 1; AltName: Full=HCV core-binding protein HCBP12; AltName: Full=Prostate short-chain dehydrogenase/reductase 1; AltName: Full=Retinal reductase 1; Short=RalR1; AltName: Full=Short chain dehydrogenase/reductase family 7C member 1 && PF00106:short chain dehydrogenase
Lachesis_group0__13_contigs__l.g1964	2464.891	711.2249	1.793588	0.000912	0.013161	1575	- && - && PF01062:Bestrophin, RFP-TM, chloride channel
Lachesis_group0__13_contigs__l.g4275	423.7915	122.575	1.787141	0.000139	0.003262	492	- && - && PF09451:Autophagy-related protein 27
Lachesis_group0__13_contigs__l.g7946	1607.337	469.0463	1.777674	0.003174	0.031721	1371	- && Q54EW2.1 RecName: Full=Putative bifunctional amine oxidase DDB_G0291301; Includes: RecName: Full=Putative sarcosine oxidase;

							Short=PSO; Includes: RecName: Full=Putative L-amino-acid oxidase && PF01266:FAD dependent oxidoreductase
Lachesis_group0__13_contigs__l.g10005	68.90174	20.1397	1.77166	0.005022	0.044677	882	- && - && PF12695:Alpha/beta hydrolase family
Lachesis_group0__13_contigs__l.g1154	111.838	32.79825	1.768979	0.00102	0.014103	1797	- && - && PF12937:F-box-like
Lachesis_group0__13_contigs__l.g6365	259.3775	76.68904	1.760992	0.00106	0.014389	609	- && Q9P7I9.1 RecName: Full=Endosomal protein P24B; Flags: Precursor && PF01105:emp24/gp25L/p24 family/GOLD
Lachesis_group0__13_contigs__l.g10605	766.2658	227.1046	1.755705	6.03E-05	0.001765	1161	- && Q9UT34.1 RecName: Full=Uncharacterized protein C824.09c && PF01412:Putative GTPase activating protein for Arf
Lachesis_group0__13_contigs__l.g2419	89.51813	26.60495	1.742584	0.003655	0.035407	1119	- && P0CS31.1 RecName: Full=Probable cytosolic iron-sulfur protein assembly protein 1 >P0CS30.1 RecName: Full=Probable cytosolic iron-sulfur protein assembly protein 1 && PF00400:WD domain, G-beta repeat
Lachesis_group0__13_contigs__l.g8820	826.0265	247.7378	1.737816	0.000122	0.002955	543	- && Q9D187.1 RecName: Full=Mitotic spindle-associated MMXD complex subunit MIP18; AltName: Full=Protein FAM96B && PF01883:Domain of unknown function DUF59
Lachesis_group0__13_contigs__l.g1070	265.1424	80.19548	1.727823	0.000536	0.008957	1710	- && - && PF12937:F-box-like
Lachesis_group0__13_contigs__l.g2642	1027.078	310.3598	1.727471	0.000515	0.008676	1029	- && Q06466.1 RecName: Full=Putative vacuolar protein sorting-associated protein TDA6; AltName: Full=Topoisomerase I damage affected protein 6 && PF06101:Plant protein of unknown function (DUF946)

Lachesis_group0__13_contigs__l.g3567	406.0879	123.0942	1.723056	0.004194	0.03936	915	- && A8NWP2.2 RecName: Full=NAD-dependent protein deacylase; AltName: Full=Regulatory protein SIR2 homolog 5; Flags: Precursor && PF02146:Sir2 family
Lachesis_group0__13_contigs__l.g2527	4678.737	1421.345	1.719138	0.004887	0.043815	891	- && - && PF04140:Isoprenylcysteine carboxyl methyltransferase (ICMT) family
Lachesis_group0__13_contigs__l.g6206	3423.678	1044.766	1.712582	3.79E-05	0.001227	1089	- && Q6IE26.2 RecName: Full=Epoxide hydrolase 4; AltName: Full=Abhydrolase domain-containing protein 7; AltName: Full=Epoxide hydrolase-related protein && PF12697:Alpha/beta hydrolase family
Lachesis_group0__13_contigs__l.g9340	259.8817	79.43204	1.70704	0.000151	0.003459	888	- && Q9Y7M5.2 RecName: Full=54S ribosomal protein L10, mitochondrial; Flags: Precursor && PF00828:Ribosomal protein L18e/L15
Lachesis_group0__13_contigs__l.g3984	940.2132	288.2868	1.70618	6.89E-05	0.001975	612	- && Q5U3N0.1 RecName: Full=Pleckstrin homology domain-containing family A member 8; Short=PH domain-containing family A member 8 && PF08718:Glycolipid transfer protein (GLTP)
Lachesis_group0__13_contigs__l.g1296	379.2958	117.0427	1.69439	0.000126	0.003006	1386	- && C5PEI9.1 RecName: Full=Aspartic protease PEP3; Flags: Precursor && PF00026:Eukaryotic aspartyl protease
Lachesis_group0__13_contigs__l.g2357	1091.156	339.8315	1.683267	0.000153	0.003478	1101	- && Q4P804.1 RecName: Full=COP9 signalosome complex subunit 5 && PF01398:JAB1/Mov34/MPN/PAD-1 ubiquitin protease
Lachesis_group0__13_contigs__l.g7697	5662.127	1763.062	1.683186	5.53E-05	0.001659	4458	- && P41820.1 RecName: Full=Brefeldin A resistance protein && PF14510:ABC-transporter extracellular

							N-terminal丨PF00005:ABC transporter丨PF06422:CDR ABC transporter丨PF01061:ABC-2 type transporter
Lachesis_group0__13_contigs__l.g7479	294.1218	91.94482	1.675025	0.004674	0.042378	1509	- && - && PF02205:WH2 motif
Lachesis_group0__13_contigs__l.g2350	155.1829	48.72311	1.674329	0.000325	0.006264	1329	- && P53011.1 RecName: Full=Nucleoporin SEH1; AltName: Full=Nuclear pore protein SEH1; AltName: Full=SEC13 homolog 1 && -
Lachesis_group0__13_contigs__l.g1481	538.9994	169.8691	1.664628	7.92E-05	0.00219	1416	- && C5G0A8.1 RecName: Full=Probable leucine aminopeptidase MCYG_08380; AltName: Full=Leucyl aminopeptidase MCYG_08380; Flags: Precursor && PF04389:Peptidase family M28
Lachesis_group0__13_contigs__l.g55	196.3604	63.04736	1.642066	0.001031	0.014202	1023	- && P41838.1 RecName: Full=Poly(A)+ RNA export protein && -
Lachesis_group0__13_contigs__l.g11223	1054.526	338.0285	1.641299	5.95E-06	0.000293	3888	- && Q9M8Y0.1 RecName: Full=Probable UDP-N-acetylglucosamine--peptide N-acetylglucosaminyltransferase SEC; AltName: Full=Protein SECRET AGENT && PF13844:Glycosyl transferase family 41丨PF13424:Tetratricopeptide repeat
Lachesis_group0__13_contigs__l.g6674	95.96958	30.83218	1.638418	0.000986	0.01384	1935	- && - && PF04129:Vps52 / Sac2 family
Lachesis_group0__13_contigs__l.g1677	25368.66	8155.93	1.637159	0.000215	0.004543	468	- && - && PF16850:Peptidase inhibitor I66
Lachesis_group0__13_contigs__l.g7346	768.839	247.8295	1.633536	7.22E-05	0.002035	1116	- && P78780.2 RecName: Full=Probable aspartate-semialdehyde dehydrogenase; Short=ASA dehydrogenase; Short=ASADH; AltName: Full=Aspartate-beta-semialdehyde dehydrogenase && PF01118:Semialdehyde dehydrogenase, NAD

							binding domain|PF02774:Semialdehyde dehydrogenase, dimerisation domain
Lachesis_group0__13_contigs__l.g6371	1412.999	455.7202	1.632689	0.001023	0.014113	1038	- && O14295.1 RecName: Full=Pyridoxal reductase; Short=PL reductase; Short=PL-red && PF00248:Aldo/keto reductase family
Lachesis_group0__13_contigs__l.g5704	668.8047	215.8851	1.632669	0.00045	0.007899	2973	- && - && PF00498:FHA domain
Lachesis_group0__13_contigs__l.g9964	2903.463	936.6558	1.632306	0.003504	0.034204	1026	- && - && PF04909:Amidohydrolase
Lachesis_group0__13_contigs__l.g5966	7541.137	2438.548	1.62889	0.000595	0.00962	324	- && - && PF01185:Fungal hydrophobin
Lachesis_group0__13_contigs__l.g10211	1662.756	537.7866	1.62844	0.000584	0.009507	1995	- && D2XV59.1 RecName: Full=GTP-binding protein 1 && PF03144:Elongation factor Tu domain 2|PF00009:Elongation factor Tu GTP binding domain
Lachesis_group0__13_contigs__l.g3643	884.9014	289.0368	1.613883	0.000205	0.004374	1389	- && P47154.1 RecName: Full=CAAX prenyl protease 1; AltName: Full=A-factor-converting enzyme; AltName: Full=Prenyl protein-specific endoprotease 1; Short=PPSEP 1 && PF16491:CAAX prenyl protease N-terminal, five membrane helices|PF01435:Peptidase family M48
Lachesis_group0__13_contigs__l.g2908	2906.243	950.7543	1.61209	0.000161	0.003628	1992	- && O42773.1 RecName: Full=Serine/threonine-protein phosphatase 2B catalytic subunit A1; AltName: Full=Calcineurin A1 && PF00149:Calcineurin-like phosphoesterase
Lachesis_group0__13_contigs__l.g11636	7407.67	2426.974	1.609955	9.00E-05	0.002405	1569	- && A1CFL5.1 RecName: Full=Cytochrome P450 monooxygenase patH; AltName: Full=Patulin synthesis protein H; AltName: Full=m-cresol

							hydrolase; Flags: Precursor && PF00067:Cytochrome P450
Lachesis_group0__13_contigs__l.g8905	2970.764	974.2429	1.608618	0.000865	0.012669	1686	- && Q01772.1 RecName: Full=Aldehyde oxidase GLOX; AltName: Full=Glyoxal oxidase; Short=GLOX; Flags: Precursor && PF07250:Glyoxal oxidase N-terminus|PF09118:Domain of unknown function (DUF1929)
Lachesis_group0__13_contigs__l.g8171	62472.28	20546.15	1.604354	0.000115	0.002841	1608	- && P39992.1 RecName: Full=Uncharacterized protein YEL023C && PF09994:Uncharacterized alpha/beta hydrolase domain (DUF2235)
Lachesis_group0__13_contigs__l.g114	419.3273	138.1474	1.601034	3.39E-05	0.001137	1272	- && - && PF01694:Rhomboid family
Lachesis_group0__13_contigs__l.g4690	403.0854	133.2341	1.598896	0.001565	0.018697	981	- && - && PF01738:Dienelactone hydrolase family
Lachesis_group0__13_contigs__l.g9881	376.7301	124.3812	1.596793	0.00263	0.027515	1173	- && O74995.1 RecName: Full=TFIIH basal transcription factor complex p47 subunit; AltName: Full=Suppressor of stem-loop protein 1 homolog; Short=SSL1 homolog && PF04056:Ssl1-like
Lachesis_group0__13_contigs__l.g82	9238.004	3055.181	1.596342	5.63E-06	0.000279	1479	- && - && PF01823:MAC/Perforin domain
Lachesis_group0__13_contigs__l.g1963	449.8518	149.5128	1.589358	5.50E-05	0.001657	1452	- && - && PF04577:Protein of unknown function (DUF563)
Lachesis_group0__13_contigs__l.g4001	449.7551	149.7124	1.587862	0.000264	0.005358	423	- && - && PF13380:CoA binding domain
Lachesis_group0__13_contigs__l.g11566	349.9125	116.4378	1.587268	0.005806	0.049628	1155	- && P53871.1 RecName: Full=Probable glutamine amidotransferase DUG3; AltName: Full=Deficient in utilization of glutathione protein 3; AltName: Full=GSH degradosomal complex subunit DUG3 && PF13230:Glutamine amidotransferases class-II

Lachesis_group0__13_contigs__l.g4004	3574.046	1193.109	1.582811	9.98E-05	0.002578	663	- && O94255.2 RecName: Full=Carbonic anhydrase; AltName: Full=Carbonate dehydratase && PF00484:Carbonic anhydrase
Lachesis_group0__13_contigs__l.g1116	547.9249	183.0762	1.581683	3.63E-05	0.001188	1038	- && - && PF13383:Methyltransferase domain
Lachesis_group0__13_contigs__l.g5900	4476.047	1497.849	1.579401	6.34E-06	0.000305	2928	- && Q03516.1 RecName: Full=Uncharacterized protein RSN1; AltName: Full=Rescuer of SRO7 at high Nacl protein 1 && PF02714:Calcium-dependent channel, 7TM region, putative phosphate|PF13967:Late exocytosis, associated with Golgi transport|PF12621:Extracellular tail, of 10TM putative phosphate transporter|PF14703:Cytosolic domain of 10TM putative phosphate transporter
Lachesis_group0__13_contigs__l.g8696	226.5399	75.77202	1.578951	0.000274	0.005481	1659	- && P34809.3 RecName: Full=Glycylpeptide N-tetradecanoyltransferase; AltName: Full=Myristoyl-CoA:protein N-myristoyltransferase; Short=NMT; AltName: Full=Peptide N-myristoyltransferase && PF01233:Myristoyl-CoA:protein N-myristoyltransferase, N-terminal domain|PF02799:Myristoyl-CoA:protein N-myristoyltransferase, C-terminal domain
Lachesis_group0__13_contigs__l.g6386	433.8141	145.1652	1.5788	0.000124	0.002996	2019	- && - && PF13041:PPR repeat family
Lachesis_group0__13_contigs__l.g11214	149.412	50.48837	1.570059	0.00141	0.017525	1137	- && P42023.1 RecName: Full=Actin-2; AltName: Full=Actin II; AltName: Full=Centractin-like protein [Pneumocystis carinii] && PF00022:Actin
Lachesis_group0__13_contigs__l.g11530	1815.246	611.5205	1.57	2.72E-05	0.000969	1911	- && A8N2M6.1 RecName: Full=Amino-acid acetyltransferase, mitochondrial; AltName:

							Full=Arginine-requiring protein 2; AltName: Full=Glutamate N-acetyltransferase; AltName: Full=N-acetylglutamate synthase; Short=AGS; Short=NAGS; Flags: Precursor && PF04768:NAT, N-acetyltransferase, of N-acetylglutamate synthase
Lachesis_group0__13_contigs__l.g171	5996.916	2026.979	1.564977	0.001129	0.014919	6705	- && P78820.2 RecName: Full=Acetyl-CoA carboxylase; Short=ACC; AltName: Full=Cell untimely torn protein 6; Includes: RecName: Full=Biotin carboxylase && PF01039:Carboxyl transferase domain|PF02786:Carbamoyl-phosphate synthase L chain, ATP binding domain|PF08326:Acetyl-CoA carboxylase, central region|PF02785:Biotin carboxylase C-terminal domain|PF00289:Carbamoyl-phosphate synthase L chain, N-terminal domain|PF00364:Biotin-requiring enzyme
Lachesis_group0__13_contigs__l.g2416	337.0874	114.4153	1.561166	0.00104	0.014228	3228	- && Q9P7S5.1 RecName: Full=Probable ubiquitin carboxyl-terminal hydrolase 7; AltName: Full=Deubiquitinating enzyme 7; AltName: Full=Ubiquitin thioesterase 7; AltName: Full=Ubiquitin-specific-processing protease 7 && PF00443:Ubiquitin carboxyl-terminal hydrolase
Lachesis_group0__13_contigs__l.g7696	168.598	57.30472	1.559015	0.000858	0.012572	1758	- && Q0UG00.1 RecName: Full=ATP-dependent RNA helicase MSS116, mitochondrial; Flags: Precursor && PF00271:Helicase conserved C-

							terminal domain|PF00270:DEAD/DEAH box helicase
Lachesis_group0__13_contigs__l.g10606	739.4234	251.0986	1.55879	0.000102	0.002611	996	- && - && PF08690:GET complex subunit GET2
Lachesis_group0__13_contigs__l.g6749	282.6548	96.79323	1.549171	0.003766	0.036285	2934	- && P0CN25.1 RecName: Full=DNA polymerase epsilon subunit B; AltName: Full=DNA polymerase II subunit 2 >P0CN24.1 RecName: Full=DNA polymerase epsilon subunit B; AltName: Full=DNA polymerase II subunit 2 && PF00583:Acetyltransferase (GNAT) family|PF04042:DNA polymerase alpha/epsilon subunit B
Lachesis_group0__13_contigs__l.g9344	131.7892	45.3096	1.542613	0.001235	0.015782	1707	- && - && PF09739:Mini-chromosome maintenance replisome factor
Lachesis_group0__13_contigs__l.g8023	1501.343	516.4172	1.539976	0.000114	0.002824	1893	- && - && PF01544:CorA-like Mg2+ transporter protein
Lachesis_group0__13_contigs__l.g7342	139.7942	48.26397	1.536895	0.001946	0.021966	534	- && Q5KPF3.1 RecName: Full=Inosine triphosphate pyrophosphatase; Short=ITPase; Short=Inosine triphosphatase; AltName: Full=Non-canonical purine NTP pyrophosphatase; AltName: Full=Non-standard purine NTP pyrophosphatase; AltName: Full=Nucleoside-triphosphate diphosphatase; AltName: Full=Nucleoside-triphosphate pyrophosphatase; Short=NTPase && PF01725:Ham1 family
Lachesis_group0__13_contigs__l.g4220	3299.989	1138.335	1.535664	2.63E-05	0.000953	1239	- && O74803.1 RecName: Full=UV excision repair protein rhp23; AltName: Full=RAD23 homolog &&

							PF09280:XPC-binding domain|PF00627:UBA/TS-N domain|PF00240:Ubiquitin family
Lachesis_group0__13_contigs__l.g8795	3163.294	1094.312	1.531224	8.35E-05	0.002278	2028	- && Q2TZ06.1 RecName: Full=Protein sip5 && -
Lachesis_group0__13_contigs__l.g118	495.5396	171.42	1.531048	0.000344	0.006491	3423	- && Q27516.2 RecName: Full=Putative cytochrome P450 CYP13A8 && PF00067:Cytochrome P450
Lachesis_group0__13_contigs__l.g8222	590.5156	204.2054	1.530872	0.000366	0.006807	2214	- && Q8TB72.2 RecName: Full=Pumilio homolog 2; Short=Pumilio-2 && -
Lachesis_group0__13_contigs__l.g7406	450.4971	156.2042	1.528131	4.49E-05	0.001411	510	- && P59998.3 RecName: Full=Actin-related protein 2/3 complex subunit 4; AltName: Full=Arp2/3 complex 20 kDa subunit; Short=p20-ARC >P59999.3 RecName: Full=Actin-related protein 2/3 complex subunit 4; AltName: Full=Arp2/3 complex 20 kDa subunit; Short=p20-ARC >Q148J6.3 RecName: Full=Actin-related protein 2/3 complex subunit 4; AltName: Full=Arp2/3 complex 20 kDa subunit; Short=p20-ARC && PF05856:ARP2/3 complex 20 kDa subunit (ARPC4)
Lachesis_group0__13_contigs__l.g1372	5798.178	2021.351	1.520461	0.002543	0.026786	2001	- && A2QEQ6.1 RecName: Full=Beta-glucuronidase; Short=GlcAase; AltName: Full=Beta-D-glucuronoside glucuronosohydrolase; Flags: Precursor && PF16862:Glycosyl hydrolase family 79 C-terminal beta domain
Lachesis_group0__13_contigs__l.g545	2501.982	873.9235	1.517799	0.001852	0.021159	1437	- && P54872.2 RecName: Full=Hydroxymethylglutaryl-CoA synthase A; Short=HMG-CoA synthase A; AltName: Full=3-hydroxy-3-methylglutaryl coenzyme A synthase A

							&& PF01154:Hydroxymethylglutaryl-coenzyme A synthase N terminal|PF08540:Hydroxymethylglutaryl-coenzyme A synthase C terminal
Lachesis_group0__13_contigs__l.g8140	304.8121	106.374	1.517242	0.001282	0.016247	1428	- && Q10209.1 RecName: Full=Uncharacterized J domain-containing protein C4H3.01 && PF00226:DnaJ domain|PF14308:X-domain of DnaJ-containing
Lachesis_group0__13_contigs__l.g4084	2783.363	972.922	1.516515	0.000608	0.009747	1869	- && Q9P381.1 RecName: Full=Putative phosphatidate cytidylyltransferase; AltName: Full=CDP-DAG synthase; AltName: Full=CDP-DG synthase; AltName: Full=CDP-diacylglycerol synthase; Short=CDS; AltName: Full=CDP-diglyceride pyrophosphorylase; AltName: Full=CDP-diglyceride synthase; AltName: Full=CTP:phosphatidate cytidylyltransferase && PF01148:Cytidylyltransferase family
Lachesis_group0__13_contigs__l.g6623	202.3037	70.85786	1.510871	0.001892	0.021517	1038	- && - && PF10180:Uncharacterised conserved protein (DUF2373)
Lachesis_group0__13_contigs__l.g9921	4844.842	1701.363	1.509681	0.000265	0.005362	2154	- && - && PF04082:Fungal specific transcription factor domain
Lachesis_group0__13_contigs__l.g5933	373.3503	131.415	1.508696	0.001618	0.019161	1854	- && - && PF08202:Mis12-Mtw1 protein family
Lachesis_group0__13_contigs__l.g11241	1159.757	407.8841	1.507034	0.003818	0.036581	1833	- && D4AS41.2 RecName: Full=Uncharacterized FAD-linked oxidoreductase ARB_02478; Flags: Precursor && PF01565:FAD binding domain

Lachesis_group0__13_contigs__l.g11144	1971.118	695.3074	1.50366	0.002141	0.023581	2019	- && Q6MG06.1 RecName: Full=Guanine nucleotide-binding protein-like 1 && PF01926:50S ribosome-binding GTPase
Lachesis_group0__13_contigs__l.g5958	4665.7	1646.144	1.503155	0.000126	0.003006	432	- && P47813.2 RecName: Full=Eukaryotic translation initiation factor 1A, X-chromosomal; Short=eIF-1A X isoform; AltName: Full=Eukaryotic translation initiation factor 4C; Short=eIF-4C >Q5RA42.3 RecName: Full=Eukaryotic translation initiation factor 1A, X-chromosomal; Short=eIF-1A X isoform; AltName: Full=Eukaryotic translation initiation factor 4C; Short=eIF-4C >Q8BMJ3.3 RecName: Full=Eukaryotic translation initiation factor 1A, X-chromosomal; Short=eIF-1A X isoform; AltName: Full=Eukaryotic translation initiation factor 4C; Short=eIF-4C && PF01176:Translation initiation factor 1A / IF-1
Lachesis_group0__13_contigs__l.g9031	579.6021	204.7559	1.502138	0.001668	0.019552	1389	- && P50514.1 RecName: Full=Probable argininosuccinate lyase; Short=ASAL; AltName: Full=Arginosuccinase && PF00206:Lyase|PF14698:Argininosuccinate lyase C-terminal
Lachesis_group0__13_contigs__l.g7152	2170.302	766.7634	1.500884	0.001077	0.0145	720	- && O42901.3 RecName: Full=Uncharacterized protein C119.09c && PF04061:ORMDL family
Lachesis_group0__13_contigs__l.g9953	1947.253	688.0647	1.500832	0.001233	0.015777	1542	- && Q5AR55.1 RecName: Full=Cyrochrome P450 monooxygenase asqL; AltName: Full=4'-methoxyviridicatin/aspoquinolone biosynthesis

							cluster protein asqL; AltName: Full=Aspoquinolone biosynthesis protein L && PF00067:Cytochrome P450
Lachesis_group0__13_contigs__l.g7920	1731.075	612.3828	1.499364	0.001137	0.014994	2034	- && Q9Y7J5.1 RecName: Full=TBC domain-containing protein C1778.09 && PF00566:Rab-GTPase-TBC domain
Lachesis_group0__13_contigs__l.g1552	206.6003	73.18419	1.498752	0.000577	0.009477	993	- && Q6K9N1.1 RecName: Full=Casein kinase 1; Short=OsCKI1; AltName: Full=Protein HYBRID BREAKDOWN 2; AltName: Full=Protein LOW TEMPERATURE GROWTH 1 && PF00069:Protein kinase domain
Lachesis_group0__13_contigs__l.g7191	1234.434	438.6357	1.493198	0.001406	0.017496	894	- && Q4PI88.1 RecName: Full=Eukaryotic translation initiation factor 3 subunit F; Short=eIF3f && PF01398:JAB1/Mov34/MPN/PAD-1 ubiquitin protease|PF13012:Maintenance of mitochondrial structure and function
Lachesis_group0__13_contigs__l.g2871	2007.493	713.765	1.491902	0.000104	0.002628	5430	- && Q04781.1 RecName: Full=E3 ubiquitin-protein ligase listerin; AltName: Full=RING domain mutant killed by rtf1 deletion protein 1 && -
Lachesis_group0__13_contigs__l.g2110	1421.323	505.7448	1.491423	0.002735	0.028308	1356	- && - && PF11735:Cryptococcal mannosyltransferase 1
Lachesis_group0__13_contigs__l.g6950	657.7837	236.3397	1.476676	0.001128	0.014919	1848	- && O94547.1 RecName: Full=Serine/threonine-protein kinase srk1; AltName: Full=Sty1-regulated kinase 1 && PF00069:Protein kinase domain
Lachesis_group0__13_contigs__l.g8957	303.9166	109.542	1.474594	0.004181	0.039336	2202	- && O60070.1 RecName: Full=Set1 complex component ash2; Short=Set1C component ash2;

							AltName: Full=COMPASS component ash2; AltName: Full=Complex proteins associated with set1 protein ash2; AltName: Full=Lid2 complex component ash2; Short=Lid2C component ash2 && PF00622:SPRY domain
Lachesis_group0__13_contigs__l.g11359	301.3355	108.6768	1.472283	0.000407	0.007367	2445	- && Q9P7D4.3 RecName: Full=Homocitrate dehydratase, mitochondrial; AltName: Full=Aconitase 2; Flags: Precursor && PF00694:Aconitase C-terminal domain|PF00330:Aconitase family (aconitate hydratase)
Lachesis_group0__13_contigs__l.g2107	899.9448	325.1215	1.468864	0.000697	0.010692	1377	- && Q9VVU5.1 RecName: Full=COP9 signalosome complex subunit 1b; Short=Dch1-2; Short=Signalosome subunit 1b && PF10602:26S proteasome subunit RPN7|PF01399:PCI domain
Lachesis_group0__13_contigs__l.g2839	209.608	75.7641	1.466818	0.001443	0.017806	2688	- && - && PF12937:F-box-like
Lachesis_group0__13_contigs__l.g6398	568.5269	206.2377	1.464068	0.00094	0.013441	4278	- && P87323.1 RecName: Full=Response regulator mcs4; AltName: Full=Mitotic catastrophe suppressor 4 && PF00072:Response regulator receiver domain
Lachesis_group0__13_contigs__l.g4034	213.4855	77.71445	1.459318	0.001718	0.020004	1011	- && Q10248.2 RecName: Full=Uncharacterized mitochondrial carrier C4G9.20c && PF00153:Mitochondrial carrier protein
Lachesis_group0__13_contigs__l.g8164	278.0378	101.3313	1.457896	0.000876	0.012765	1341	- && P39992.1 RecName: Full=Uncharacterized protein YEL023C && PF09994:Uncharacterized alpha/beta hydrolase domain (DUF2235)

Lachesis_group0__13_contigs__l.g8413	397.7163	145.2027	1.45244	0.000397	0.007271	1290	- && Q9P7S2.1 RecName: Full=Probable 26S proteasome regulatory subunit rpn6 && PF01399:PCI domain
Lachesis_group0__13_contigs__l.g6782	2364.967	868.8974	1.444555	5.54E-05	0.001659	2532	- && Q9Y7Y8.1 RecName: Full=Probable glutamine--tRNA ligase; AltName: Full=Glutaminyl-tRNA synthetase; Short=GlnRS && PF03950:tRNA synthetases class I (E and Q), anti-codon binding domain|PF04557:Glutaminyl-tRNA synthetase, non-specific RNA binding region part 2|PF00749:tRNA synthetases class I (E and Q), catalytic domain|PF04558:Glutaminyl-tRNA synthetase, non-specific RNA binding region part 1
Lachesis_group0__13_contigs__l.g2914	417.0924	153.6032	1.441881	0.000205	0.004374	942	- && Q9RA05.1 RecName: Full=(-)-trans-carveol dehydrogenase; AltName: Full=(4R,6S)-carveol dehydrogenase; Short=CDH; Short=Carveol dehydrogenase && PF00106:short chain dehydrogenase
Lachesis_group0__13_contigs__l.g815	1128.989	417.1465	1.436692	0.000691	0.010646	504	- && Q9CWZ3.3 RecName: Full=RNA-binding protein 8A; AltName: Full=RNA-binding motif protein 8A; AltName: Full=Ribonucleoprotein RBM8A && PF00076:RNA recognition motif. (a.k.a. RRM, RBD, or RNP domain)
Lachesis_group0__13_contigs__l.g5679	261.609	96.83419	1.431332	0.001021	0.014103	675	- && Q9ZPW2.2 RecName: Full=Anaphase-promoting complex subunit 10; Short=APC10; AltName: Full=Cyclosome subunit 10 &&

							PF03256:Anaphase-promoting complex, subunit 10 (APC10)
Lachesis_group0__13_contigs__l.g5871	1794.447	665.8564	1.430011	0.001166	0.015186	999	- && P20654.1 RecName: Full=Serine/threonine-protein phosphatase PP1 && PF16891:Serine-threonine protein phosphatase N-terminal domain|PF00149:Calcineurin-like phosphoesterase
Lachesis_group0__13_contigs__l.g7520	6487.26	2407.816	1.429799	0.004581	0.041747	2304	- && P04842.2 RecName: Full=Alcohol oxidase 1; Short=AO 1; Short=AOX 1; AltName: Full=Methanol oxidase 1; Short=MOX 1 >F2QY27.1 RecName: Full=Alcohol oxidase 1; Short=AO 1; Short=AOX 1; AltName: Full=Methanol oxidase 1; Short=MOX 1 && PF00732:GMC oxidoreductase|PF05199:GMC oxidoreductase
Lachesis_group0__13_contigs__l.g10635	643.7551	239.0441	1.429201	0.005261	0.046211	1791	- && P38213.2 RecName: Full=Protein DSF2; AltName: Full=Deletion suppressor of MPT5 mutation protein 2 && -
Lachesis_group0__13_contigs__l.g10264	771.0577	288.7144	1.417796	0.000482	0.008303	1050	- && P25605.2 RecName: Full=Acetolactate synthase small subunit, mitochondrial; AltName: Full=Acetohydroxy-acid synthase small subunit; Short=AHAS; Short=ALS; Flags: Precursor && PF10369:Small subunit of acetolactate synthase|PF01842:ACT domain
Lachesis_group0__13_contigs__l.g8685	411.9874	154.4791	1.41625	0.000538	0.00898	1188	- && - && PF13324:Grap2 and cyclin-D-interacting
Lachesis_group0__13_contigs__l.g5538	135.8472	50.99223	1.412861	0.005021	0.044677	1629	- && O23051.1 RecName: Full=Ent-kaurenoic acid oxidase 1; Short=AtKAO1; AltName:

							Full=Cytochrome P450 88A3 && PF00067:Cytochrome P450
Lachesis_group0__13_contigs__l.g2249	558.8529	209.9888	1.411792	0.000261	0.005311	894	- && Q6DH23.1 RecName: Full=Vacuolar protein sorting-associated protein 26B-like; AltName: Full=Vesicle protein sorting 26B-like && PF03643:Vacuolar protein sorting-associated protein 26
Lachesis_group0__13_contigs__l.g378	655.6125	246.732	1.409606	0.000854	0.012545	3030	- && C8VDQ4.1 RecName: Full=Putative GTPase-activating protein AN11010 && PF00566:Rab-GTPase-TBC domain
Lachesis_group0__13_contigs__l.g4736	717.8925	270.3193	1.409596	0.000223	0.0047	1344	- && Q99078.2 RecName: Full=Dual specificity protein kinase FUZ7 && PF00069:Protein kinase domain
Lachesis_group0__13_contigs__l.g9227	416.0254	157.0412	1.406398	0.000603	0.009716	1623	- && P87041.3 RecName: Full=UDP-galactose transporter; AltName: Full=Golgi UDP-Gal transporter && PF04142:Nucleotide-sugar transporter
Lachesis_group0__13_contigs__l.g9583	153.0704	57.79365	1.405011	0.0035	0.034204	1017	- && A8WFS8.1 RecName: Full=Post-GPI attachment to proteins factor 3; AltName: Full=PER1-like domain-containing protein 1; Flags: Precursor && PF04080:Per1-like
Lachesis_group0__13_contigs__l.g376	588.1287	222.7927	1.40135	0.000393	0.007243	822	- && - && PF10021:Uncharacterized protein conserved in bacteria (DUF2263)
Lachesis_group0__13_contigs__l.g8611	957.261	362.5867	1.400937	0.001034	0.014217	495	- && P53937.1 RecName: Full=37S ribosomal protein SWS2, mitochondrial; AltName: Full=Sick without

							securin protein 2 && PF00416:Ribosomal protein S13/S18
Lachesis_group0__13_contigs__l.g5734	2665.308	1014.841	1.393068	0.00035	0.006563	1470	- && Q9UQW9.1 RecName: Full=Ornithine decarboxylase; Short=ODC && PF02784:Pyridoxal-dependent decarboxylase, pyridoxal binding domain|PF00278:Pyridoxal-dependent decarboxylase, C-terminal sheet domain
Lachesis_group0__13_contigs__l.g7163	545.4835	207.895	1.392073	0.005631	0.048478	1977	- && Q2KIH4.1 RecName: Full=Ribosome biogenesis regulatory protein homolog && PF04176:TIP41-like family|PF04939:Ribosome biogenesis regulatory protein (RRS1)
Lachesis_group0__13_contigs__l.g11602	304.0094	115.7324	1.391233	0.005176	0.045732	2058	- && - && PF05920:Homeobox KN domain
Lachesis_group0__13_contigs__l.g11442	770.6117	294.9735	1.385282	0.000148	0.003425	1032	- && Q8TG13.1 RecName: Full=Casein kinase II subunit alpha; Short=CK II subunit alpha && PF00069:Protein kinase domain
Lachesis_group0__13_contigs__l.g1615	565.9436	216.9291	1.383587	0.004682	0.042381	1779	- && O42922.1 RecName: Full=Uncharacterized MFS-type transporter C16A3.17c && PF07690:Major Facilitator Superfamily
Lachesis_group0__13_contigs__l.g1329	181.9479	69.74071	1.382099	0.002624	0.027479	1680	- && - && PF01728:FtsJ-like methyltransferase
Lachesis_group0__13_contigs__l.g9040	180.3119	69.64212	1.372959	0.003029	0.030686	1629	- && - && PF13532:2OG-Fe(II) oxygenase superfamily
Lachesis_group0__13_contigs__l.g1503	2893.173	1119.137	1.370204	0.001342	0.016802	1323	- && Q4PBZ9.2 RecName: Full=Non-histone chromosomal protein 6 && PF00505:HMG (high mobility group) box|PF01975:Survival protein SurE
Lachesis_group0__13_contigs__l.g7460	149.2647	57.63682	1.369767	0.004074	0.038526	597	- && P0CR81.1 RecName: Full=Translation machinery-associated protein 22 >P0CR80.1

							RecName: Full=Translation machinery-associated protein 22 && PF01253:Translation initiation factor SUI1
Lachesis_group0__13_contigs__l.g11070	1913.763	742.9762	1.365129	0.00152	0.018476	3867	- && Q5F418.1 RecName: Full=26S proteasome non-ATPase regulatory subunit 1; AltName: Full=26S proteasome regulatory subunit RPN2; AltName: Full=26S proteasome regulatory subunit S1 && PF02826:D-isomer specific 2-hydroxyacid dehydrogenase, NAD binding domain|PF13646:HEAT repeats|PF00389:D-isomer specific 2-hydroxyacid dehydrogenase, catalytic domain|PF01851:Proteasome/cyclosome repeat
Lachesis_group0__13_contigs__l.g2112	1578.104	613.6116	1.363107	0.000327	0.006273	735	- && - && PF11807:Domain of unknown function (DUF3328)
Lachesis_group0__13_contigs__l.g6652	3785.219	1472.255	1.362322	0.000818	0.012111	3060	- && O94609.1 RecName: Full=Ubiquitin-activating enzyme E1 1; AltName: Full=Poly(A)+ RNA transport protein 3 && PF16190:Ubiquitin-activating enzyme E1 FCCH domain|PF10585:Ubiquitin-activating enzyme active site|PF16191:Ubiquitin-activating enzyme E1 four-helix bundle|PF09358:Ubiquitin fold domain|PF00899:ThiF family
Lachesis_group0__13_contigs__l.g4714	192.5955	74.84776	1.361698	0.002196	0.024011	1575	- && Q86XW9.1 RecName: Full=Thioredoxin domain-containing protein 6; AltName: Full=Thioredoxin-like protein 2; Short=Txl-2 && PF00334:Nucleoside diphosphate kinase

Lachesis_group0__13_contigs__l.g5273	1463.525	569.7597	1.361099	0.000653	0.010202	2817	- && P73177.1 RecName: Full=Uncharacterized ribonuclease sll1290 && PF00773:RNB domain
Lachesis_group0__13_contigs__l.g9351	350.3431	136.765	1.358079	0.000582	0.009498	2529	- && - && PF05622:HOOK protein
Lachesis_group0__13_contigs__l.g6658	546.9104	213.6369	1.35624	0.001099	0.014667	795	- && Q1MTR2.2 RecName: Full=RNA-binding protein with serine-rich domain 1 homolog && PF00076:RNA recognition motif. (a.k.a. RRM, RBD, or RNP domain)
Lachesis_group0__13_contigs__l.g10889	377.3457	147.6476	1.353731	0.001109	0.014755	939	- && Q06143.1 RecName: Full=Mitochondrial dicarboxylate transporter; AltName: Full=DTP; AltName: Full=Dicarboxylate carrier 1 && PF00153:Mitochondrial carrier protein
Lachesis_group0__13_contigs__l.g7955	254.8787	99.77412	1.352723	0.001434	0.017716	957	- && P25416.2 RecName: Full=Protein qutG && PF00459:Inositol monophosphatase family
Lachesis_group0__13_contigs__l.g2037	882.2064	345.9438	1.350038	0.003783	0.036412	2976	- && Q7S8R8.2 RecName: Full=26S proteasome regulatory subunit rpn-1 && -
Lachesis_group0__13_contigs__l.g5552	4537.387	1784.324	1.346696	0.001672	0.019552	1521	- && O94260.1 RecName: Full=Putative G3BP-like protein && PF02136:Nuclear transport factor 2 (NTF2) domain|PF00076:RNA recognition motif. (a.k.a. RRM, RBD, or RNP domain)
Lachesis_group0__13_contigs__l.g6054	414.1983	163.4807	1.339564	0.001737	0.020194	1248	- && O74480.1 RecName: Full=Uncharacterized protein C1840.07c; Flags: Precursor && PF00149:Calcineurin-like phosphoesterase
Lachesis_group0__13_contigs__l.g9111	673.8232	266.1321	1.339502	0.002225	0.024194	891	- && Q5ZIM5.1 RecName: Full=Methionine aminopeptidase 1; Short=MAP 1; Short=MetAP 1; AltName: Full=Peptidase M 1 && PF00557:Metallopeptidase family M24

Lachesis_group0__13_contigs__l.g10801	762.8114	301.5412	1.339186	0.00115	0.01511	903	- && A5GFW7.1 RecName: Full=Protein RTF2 homolog; AltName: Full=Replication termination factor 2 domain-containing protein 1 && PF04641:Rtf2 RING-finger
Lachesis_group0__13_contigs__l.g10617	815.7695	322.7339	1.337384	0.000443	0.007829	1941	- && Q0CJV3.1 RecName: Full=Vacuolar protein sorting-associated protein 27 && PF01363:FYVE zinc finger|PF03127:GAT domain|PF00790:VHS domain
Lachesis_group0__13_contigs__l.g4614	968.1334	384.0392	1.333793	0.001157	0.015153	687	- && Q8WUJ0.1 RecName: Full=Serine/threonine/tyrosine-interacting protein; AltName: Full=Protein tyrosine phosphatase-like protein && PF00782:Dual specificity phosphatase, catalytic domain
Lachesis_group0__13_contigs__l.g11708	7303.999	2904.356	1.330513	0.001004	0.014013	4278	- && Q9UUF7.1 RecName: Full=Coatomer subunit beta; AltName: Full=Beta-coat protein; Short=Beta-COP && PF07718:Coatomer beta C-terminal region|PF02133:Permease for cytosine/purines, uracil, thiamine, allantoin|PF14806:Coatomer beta subunit appendage platform|PF01602:Adaptin N terminal region
Lachesis_group0__13_contigs__l.g8667	268.3843	106.812	1.329635	0.003375	0.033304	1887	- && - && PF00096:Zinc finger, C2H2 type
Lachesis_group0__13_contigs__l.g975	380.5185	151.6778	1.327707	0.000626	0.009951	612	- && Q64520.2 RecName: Full=Guanylate kinase; AltName: Full=GMP kinase && PF00625:Guanylate kinase
Lachesis_group0__13_contigs__l.g5846	933.1547	372.512	1.325563	0.002686	0.027908	1311	- && Q10234.1 RecName: Full=Probable 37S ribosomal protein S5, mitochondrial && PF00333:Ribosomal protein S5, N-terminal

							domain|PF03719:Ribosomal protein S5, C-terminal domain
Lachesis_group0__13_contigs__l.g7966	228.9384	91.26283	1.324725	0.004204	0.03936	2697	- && Q13546.3 RecName: Full=Receptor-interacting serine/threonine-protein kinase 1; AltName: Full=Cell death protein RIP; AltName: Full=Receptor-interacting protein 1; Short=RIP-1; AltName: Full=Serine/threonine-protein kinase RIP && PF07714:Protein tyrosine kinase
Lachesis_group0__13_contigs__l.g10060	702.2947	281.2041	1.321401	0.001038	0.014228	720	- && - && PF00240:Ubiquitin family|PF02179:BAG domain
Lachesis_group0__13_contigs__l.g5017	266.9758	107.7871	1.308158	0.001261	0.01605	1770	- && - && PF00566:Rab-GTPase-TBC domain
Lachesis_group0__13_contigs__l.g3582	358.505	145.012	1.307942	0.003671	0.035498	777	- && - && PF13489:Methyltransferase domain
Lachesis_group0__13_contigs__l.g3735	490.5714	198.4827	1.305592	0.000909	0.013149	1905	- && Q9UNZ2.2 RecName: Full=NSFL1 cofactor p47; AltName: Full=UBX domain-containing protein 2C; AltName: Full=p97 cofactor p47 >Q5RBG3.1 RecName: Full=NSFL1 cofactor p47; AltName: Full=p97 cofactor p47 && PF00789:UBX domain|PF08059:SEP domain
Lachesis_group0__13_contigs__l.g1502	3953.293	1601.988	1.303105	0.000721	0.010963	1191	- && P0C199.1 RecName: Full=Protein KES1 && PF01237:Oxysterol-binding protein
Lachesis_group0__13_contigs__l.g4161	525.6766	213.8049	1.298987	0.003084	0.031055	1437	- && Q4P4G8.1 RecName: Full=NKAP family protein UM04995 && PF06047:Ras-induced vulval development antagonist
Lachesis_group0__13_contigs__l.g6229	1085.568	441.3464	1.298865	0.000952	0.013515	3714	- && O14306.2 RecName: Full=Probable inositol polyphosphate 5-phosphatase C9G1.10c &&

							PF03372:Endonuclease/Exonuclease/phosphatase family
Lachesis_group0__13_contigs__l.g643	1414.386	575.4757	1.297659	0.00178	0.020516	1695	- && - && PF12937:F-box-like
Lachesis_group0__13_contigs__l.g3337	5435.765	2214.234	1.295717	0.004967	0.044415	1725	- && - && PF00106:short chain dehydrogenase
Lachesis_group0__13_contigs__l.g9869	1155.675	471.6734	1.292822	0.001809	0.020764	771	- && Q08954.1 RecName: Full=Smr domain-containing protein YPL199C && PF01713:Smr domain|PF08590:Domain of unknown function (DUF1771)
Lachesis_group0__13_contigs__l.g10152	1438.91	588.9434	1.28893	0.001007	0.014013	1584	- && Q9Y884.1 RecName: Full=MAP kinase kinase skh1/pek1 && PF00069:Protein kinase domain
Lachesis_group0__13_contigs__l.g6744	812.6641	334.2516	1.282046	0.002552	0.026836	2769	- && Q9UKV8.3 RecName: Full=Protein argonaute-2; Short=Argonaute2; Short=hAgo2; AltName: Full=Argonaute RISC catalytic component 2; AltName: Full=Eukaryotic translation initiation factor 2C 2; Short=eIF-2C 2; Short=eIF2C 2; AltName: Full=PAZ Piwi domain protein; Short=PPD; AltName: Full=Protein slicer && PF02171:Piwi domain|PF16487:Mid domain of argonaute|PF02170:PAZ domain|PF08699:Argonaute linker 1 domain|PF16488:Argonaute linker 2 domain|PF16486:N-terminal domain of argonaute
Lachesis_group0__13_contigs__l.g1926	854.2577	351.5513	1.280559	0.00334	0.033127	1338	- && A6H7B5.1 RecName: Full=COP9 signalosome complex subunit 3; Short=SGN3; Short=Signalosome subunit 3 && -

Lachesis_group0__13_contigs__l.g4543	771.1341	318.5828	1.275148	0.004076	0.038526	3582	- && Q0CL79.2 RecName: Full=Cytokinesis protein sepH; AltName: Full=Serine/threonine-protein kinase sepH && PF00069:Protein kinase domain
Lachesis_group0__13_contigs__l.g9250	218.1452	90.08204	1.274211	0.004061	0.038446	1839	- && O74927.2 RecName: Full=Methylenetetrahydrofolate reductase 2 && PF02219:Methylenetetrahydrofolate reductase
Lachesis_group0__13_contigs__l.g8958	399.91	165.313	1.273757	0.00231	0.024872	438	- && P35129.1 RecName: Full=Ubiquitin-conjugating enzyme E2 2; AltName: Full=E2 ubiquitin-conjugating enzyme 2; AltName: Full=Lethal protein 70; AltName: Full=Ubiquitin carrier protein 2; AltName: Full=Ubiquitin-protein ligase 2 && PF00179:Ubiquitin-conjugating enzyme
Lachesis_group0__13_contigs__l.g11550	590.8525	244.5743	1.273188	0.000956	0.013546	1680	- && O94303.3 RecName: Full=Imidazole glycerol phosphate synthase hisHF; Short=IGP synthase; Short=IGPS; Short=ImGP synthase; Includes: RecName: Full=Glutamine amidotransferase; Includes: RecName: Full=Cyclase && PF00117:Glutamine amidotransferase class-I|PF00977:Histidine biosynthesis protein
Lachesis_group0__13_contigs__l.g283	365.3965	151.5107	1.271774	0.00252	0.026663	954	- && Q5RFR8.1 RecName: Full=Coatomer subunit epsilon; AltName: Full=Epsilon-coat protein; Short=Epsilon-COP && PF04733:Coatomer epsilon subunit
Lachesis_group0__13_contigs__l.g243	2698.205	1119.136	1.269654	0.00038	0.007045	546	- && P34737.1 RecName: Full=40S ribosomal protein S15; AltName: Full=S12 && PF00203:Ribosomal protein S19

Lachesis_group0__13_contigs__l.g4735	1612.014	669.9327	1.266693	0.001018	0.014103	2649	- && P25375.1 RecName: Full=Saccharolysin; AltName: Full=Oligopeptidase YSCD; AltName: Full=Protease D; AltName: Full=Proteinase yscD && PF01432:Peptidase family M3
Lachesis_group0__13_contigs__l.g2428	2377.243	989.6377	1.264569	0.001511	0.018391	1437	- && O23254.1 RecName: Full=Serine hydroxymethyltransferase 4; Short=AtSHMT4; AltName: Full=Glycine hydroxymethyltransferase 4; AltName: Full=Serine methylase 4 && PF00464:Serine hydroxymethyltransferase
Lachesis_group0__13_contigs__l.g2759	1062.599	443.094	1.262121	0.001244	0.015873	966	- && Q9UUI8.1 RecName: Full=Uncharacterized transporter C22F8.04 && PF03151:Triose-phosphate Transporter family
Lachesis_group0__13_contigs__l.g5606	530.2103	221.9358	1.257213	0.00249	0.026401	375	- && Q9VU02.1 RecName: Full=Probable small nuclear ribonucleoprotein Sm D1; Short=Sm-D1; AltName: Full=snRNP core protein D1 && PF01423:LSM domain
Lachesis_group0__13_contigs__l.g11075	2282.863	957.597	1.253547	0.000675	0.010459	1599	- && G5EB89.1 RecName: Full=Importin subunit alpha; AltName: Full=Karyopherin alpha && PF16186:Atypical Arm repeat|PF01749:Importin beta binding domain|PF00514:Armadillo/beta-catenin-like repeat
Lachesis_group0__13_contigs__l.g8074	498.032	209.1266	1.252498	0.002236	0.024194	684	- && Q5RBL6.1 RecName: Full=Golgi SNAP receptor complex member 1; AltName: Full=28 kDa Golgi SNARE protein; AltName: Full=28 kDa cis-Golgi SNARE p28 && -

Lachesis_group0__13_contigs__l.g267	1048.426	440.4509	1.251318	0.002078	0.023061	1896	- && P0C155.1 PUTATIVE PSEUDOGENE: RecName: Full=Putative carboxypeptidase YOL153C && PF07687:Peptidase dimerisation domain|PF01546:Peptidase family M20/M25/M40
Lachesis_group0__13_contigs__l.g1371	3733.3	1571.141	1.248596	0.003703	0.035736	4026	- && Q4PBP6.2 RecName: Full=Stress response protein NST1 && PF13945:Salt tolerance down-regulator
Lachesis_group0__13_contigs__l.g819	351.3387	148.6066	1.241554	0.001771	0.020458	1524	- && - && PF13923:Zinc finger, C3HC4 type (RING finger)
Lachesis_group0__13_contigs__l.g11282	1880.715	797.7742	1.236981	0.000913	0.013161	1032	- && B0CQN7.1 RecName: Full=NADH-cytochrome b5 reductase 1; AltName: Full=Microsomal cytochrome b reductase && PF00175:Oxidoreductase NAD-binding domain|PF00970:Oxidoreductase FAD-binding domain
Lachesis_group0__13_contigs__l.g9225	1295.699	554.746	1.223869	0.001683	0.019632	3231	- && Q92900.2 RecName: Full=Regulator of nonsense transcripts 1; AltName: Full=ATP-dependent helicase RENT1; AltName: Full=Nonsense mRNA reducing factor 1; Short=NORF1; AltName: Full=Up-frameshift suppressor 1 homolog; Short=hUpf1 && PF13086:AAA domain|PF13087:AAA domain|PF13245:Part of AAA domain|PF09416:RNA helicase (UPF2 interacting domain)

Lachesis_group0__13_contigs__l.g6447	620.4853	266.4594	1.220406	0.004925	0.044078	2241	- && Q9P787.1 RecName: Full=ER membrane protein complex subunit 3 && PF01956:Integral membrane protein DUF106
Lachesis_group0__13_contigs__l.g4870	303.5815	130.5318	1.218114	0.001581	0.0188	2730	- && P78810.2 RecName: Full=Vacuolar transporter chaperone 4 && PF02656:Domain of unknown function (DUF202)|PF09359:VTC domain|PF03105:SPX domain
Lachesis_group0__13_contigs__l.g1729	1622.328	699.6216	1.213715	0.004384	0.040594	2592	- && O13864.1 RecName: Full=Importin subunit beta-1; AltName: Full=Importin-95; AltName: Full=Karyopherin subunit beta-1; AltName: Full=Karyopherin-95 && PF13513:HEAT-like repeat|PF03810:Importin-beta N-terminal domain
Lachesis_group0__13_contigs__l.g4837	1126.668	486.8059	1.21046	0.002834	0.029231	2142	- && Q8J0F9.1 RecName: Full=Deshydrogenase mlcG; AltName: Full=Compactin biosynthesis protein G; AltName: Full=Enoyl reductase && PF08240:Alcohol dehydrogenase GroES-like domain|PF00107:Zinc-binding dehydrogenase
Lachesis_group0__13_contigs__l.g5989	2009.944	871.3798	1.205967	0.002846	0.029313	2325	- && P33277.1 RecName: Full=GTPase-activating protein; AltName: Full=Ras GTPase-activating protein && PF03836:RasGAP C-terminus|PF00616:GTPase-activator protein for Ras-like GTPase
Lachesis_group0__13_contigs__l.g10253	1127.619	489.5318	1.203945	0.002758	0.028512	3396	- && O60111.2 RecName: Full=ABC1 family protein MCP2 homolog; Flags: Precursor && PF03109:ABC1 family|PF00566:Rab-GTPase-TBC domain

Lachesis_group0__13_contigs__l.g2150	1271.663	552.3269	1.203262	0.000673	0.010449	357	- && Q8QG64.2 RecName: Full=RING-box protein 1; Short=Rbx1; AltName: Full=Hyperosmotic protein 21; Short=sHOP21 && PF12678:RING-H2 zinc finger
Lachesis_group0__13_contigs__l.g7293	1118.796	487.4431	1.198713	0.001196	0.01544	1956	- && Q9USP8.2 RecName: Full=Isocitrate dehydrogenase [NAD] subunit 2, mitochondrial; AltName: Full=Isocitric dehydrogenase; AltName: Full=NAD(+)-specific ICDH; Flags: Precursor && PF03357:Snf7|PF00180:Isocitrate/isopropylmalate dehydrogenase
Lachesis_group0__13_contigs__l.g8560	3783.262	1650.196	1.197035	0.000607	0.009747	3309	- && Q9UTT1.2 RecName: Full=Ubiquitin carboxyl-terminal hydrolase 21; AltName: Full=Deubiquitinating enzyme 21; AltName: Full=Ubiquitin thioesterase 21; AltName: Full=Ubiquitin-specific-processing protease 21 && PF14533:Ubiquitin-specific protease C-terminal|PF00917:MATH domain|PF12436:ICP0-binding domain of Ubiquitin-specific protease 7|PF00443:Ubiquitin carboxyl-terminal hydrolase
Lachesis_group0__13_contigs__l.g5380	667.7744	291.6421	1.195287	0.001934	0.021857	1911	- && Q6PD26.3 RecName: Full=GPI transamidase component PIG-S; AltName: Full=Phosphatidylinositol-glycan biosynthesis class S protein && PF10510:Phosphatidylinositol-glycan biosynthesis class S protein
Lachesis_group0__13_contigs__l.g8559	1316.659	576.0912	1.192641	0.002937	0.030062	756	- && Q9UUA4.1 RecName: Full=Translocation protein sec66 && PF09802:Preprotein translocase subunit Sec66

Lachesis_group0__13_contigs__l.g4149	1974.479	868.2063	1.185613	0.001541	0.018585	2370	- && B0D0N9.1 RecName: Full=Protein SEY1 && PF05879:Root hair defective 3 GTP-binding protein (RHD3)
Lachesis_group0__13_contigs__l.g8591	434.3569	192.165	1.17691	0.002203	0.024034	2670	- && A5PJS6.1 RecName: Full=Ubiquitin carboxyl-terminal hydrolase 10; AltName: Full=Deubiquitinating enzyme 10; AltName: Full=Ubiquitin thioesterase 10; AltName: Full=Ubiquitin-specific-processing protease 10 && PF00443:Ubiquitin carboxyl-terminal hydrolase
Lachesis_group0__13_contigs__l.g4700	1076.144	476.8591	1.174617	0.002032	0.022745	2991	- && P87235.1 RecName: Full=DNA repair protein rhp42 && PF03835:Rad4 transglutaminase-like domain|PF10405:Rad4 beta-hairpin domain 3|PF10404:Rad4 beta-hairpin domain 2|PF10403:Rad4 beta-hairpin domain 1|PF01841:Transglutaminase-like superfamily
Lachesis_group0__13_contigs__l.g6928	715.7834	318.0189	1.170866	0.004442	0.040946	1590	- && - && PF09816:RNA polymerase II transcription elongation factor
Lachesis_group0__13_contigs__l.g6045	4109.144	1828.733	1.168118	0.003797	0.036509	1113	- && O42964.1 RecName: Full=Transcription factor iws1 && PF08711:TFIIS helical bundle-like domain
Lachesis_group0__13_contigs__l.g5528	2563.612	1148.223	1.158982	0.001988	0.022348	2847	- && Q4P9K4.1 RecName: Full=Protein transport protein SEC24 && PF04810:Sec23/Sec24 zinc finger|PF00626:Gelsolin repeat|PF04811:Sec23/Sec24 trunk domain|PF00397:WW domain|PF04815:Sec23/Sec24 helical

							domain丨PF08033:Sec23/Sec24 beta-sandwich domain
Lachesis_group0__13_contigs__l.g10159	1362.408	611.7225	1.155196		0.001209	0.015529	1725	- && B0D0T8.1 RecName: Full=Transcription activator of gluconeogenesis ERT1 && -
Lachesis_group0__13_contigs__l.g3581	3014.778	1353.819	1.155113		0.002993	0.030423	1341	- && Q10142.1 RecName: Full=Inositol phosphorylceramide synthase catalytic subunit aur1; Short=IPC synthase catalytic subunit aur1; AltName: Full=Aureobasidin A resistance protein homolog; AltName: Full=Phosphatidylinositol:ceramide phosphoinositol transferase && PF14378:PAP2 superfamily
Lachesis_group0__13_contigs__l.g5202	1837.572	828.7136	1.148969		0.001976	0.022239	1689	- && - && PF01435:Peptidase family M48
Lachesis_group0__13_contigs__l.g1509	5202.794	2352.072	1.145275		0.004127	0.038917	3957	- && Q90YK3.1 RecName: Full=L-gulonolactone oxidase; Short=LGO; AltName: Full=L-gulono-gamma-lactone oxidase; Short=GLO && PF04030:D-arabinono-1,4-lactone oxidase丨PF01565:FAD binding domain
Lachesis_group0__13_contigs__l.g6034	1169.905	531.355	1.138446		0.00132	0.016625	1566	- && B6H9U8.1 RecName: Full=Secondary metabolism regulator laeA; AltName: Full=Methyltransferase laeA; AltName: Full=Velvet complex subunit laeA && PF12847:Methyltransferase domain
Lachesis_group0__13_contigs__l.g2245	1207.779	557.7968	1.114645		0.001672	0.019552	669	- && Q08971.1 RecName: Full=Protein PBDC1 homolog && PF04669:Polysaccharide biosynthesis

Lachesis_group0__13_contigs__l.g8883	1237.069	572.4707	1.11161		0.005756	0.049281	1230	- && P78774.3 RecName: Full=Actin-related protein 2/3 complex subunit 1; AltName: Full=Arp2/3 complex 41 kDa subunit; AltName: Full=p41-ARC && -
Lachesis_group0__13_contigs__l.g7917	262.4117	121.6686	1.109448		0.005453	0.047497	1338	- && Q9HD43.3 RecName: Full=Receptor-type tyrosine-protein phosphatase H; Short=R-PTP-H; AltName: Full=Stomach cancer-associated protein tyrosine phosphatase 1; Short=SAP-1; AltName: Full=Transmembrane-type protein-tyrosine phosphatase type H; Flags: Precursor && PF00102:Protein-tyrosine phosphatase
Lachesis_group0__13_contigs__l.g11295	4014.522	1864.97	1.10614		0.002997	0.030423	2796	- && P53125.1 RecName: Full=Imitation switch two complex protein 1 && PF02791:DDT domain|PF10537:ATP-utilising chromatin assembly and remodelling N-terminal|PF15614:WSTF, HB1, Itc1p, MBD9 motif 3
Lachesis_group0__13_contigs__l.g6309	894.5195	415.7573	1.105682		0.005142	0.045514	1812	- && Q6DRJ9.1 RecName: Full=Synembryn-B; AltName: Full=Protein Ric-8B; AltName: Full=Synembryn-like && PF10165:Guanine nucleotide exchange factor synembryn
Lachesis_group0__13_contigs__l.g3333	1806.021	840.1524	1.104182		0.001531	0.018561	303	- && P55853.1 RecName: Full=Small ubiquitin-related modifier; Short=SUMO; AltName: Full=Ubiquitin-like protein SMT3; Flags: Precursor && PF11976:Ubiquitin-2 like Rad60 SUMO-like

Lachesis_group0__13_contigs__l.g5243	1414.263	659.0152	1.101334	0.003879	0.037065	1530	- && P87035.1 RecName: Full=Guanine nucleotide-binding protein alpha-4 subunit && PF00503:G-protein alpha subunit
Lachesis_group0__13_contigs__l.g1351	1033.81	485.6751	1.090396	0.004585	0.041747	2631	- && Q5AP95.1 RecName: Full=Suppressor of ferric uptake 1 && PF08550:Fungal protein of unknown function (DUF1752)|PF00320:GATA zinc finger
Lachesis_group0__13_contigs__l.g572	1230.299	578.4405	1.089106	0.003653	0.035407	3960	- && B2GUZ1.1 RecName: Full=Ubiquitin carboxyl-terminal hydrolase 4; AltName: Full=Deubiquitinating enzyme 4; AltName: Full=Ubiquitin thioesterase 4; AltName: Full=Ubiquitin-specific-processing protease 4 && PF06337:DUSP domain|PF00443:Ubiquitin carboxyl-terminal hydrolase
Lachesis_group0__13_contigs__l.g10985	2248.148	1059.707	1.085257	0.004605	0.041858	603	- && Q9P720.1 RecName: Full=60S ribosomal protein L16; AltName: Full=Cytoplasmic ribosomal protein 46 && PF00572:Ribosomal protein L13
Lachesis_group0__13_contigs__l.g4547	351.7648	166.6885	1.07776	0.004683	0.042381	1866	- && Q9HGM6.1 RecName: Full=Putative transporter C543.05c && PF00955:HCO3-transporter family
Lachesis_group0__13_contigs__l.g2549	824.9005	394.7509	1.063172	0.005467	0.047539	1923	- && Q4IQ08.1 RecName: Full=GPI-anchored wall transfer protein 1 && PF06423:GWT1
Lachesis_group0__13_contigs__l.g2710	1052.643	506.6699	1.054962	0.005502	0.047726	3306	- && Q92540.2 RecName: Full=Protein SMG7; AltName: Full=EST1-like protein C; AltName: Full=SMG-7 homolog; Short=hSMG-7 && PF10373:Est1 DNA/RNA binding domain|PF10374:Telomerase activating protein Est1

Lachesis_group0__13_contigs__l.g3188	1488.749	717.9258	1.052026	0.004851	0.043523	2589	- && P46592.3 RecName: Full=Glycolipid 2-alpha-mannosyltransferase 2; AltName: Full=Alpha-1,2-mannosyltransferase 2 && PF01793:Glycolipid 2-alpha-mannosyltransferase
Lachesis_group0__13_contigs__l.g8738	957.2167	475.6837	1.008727	0.004049	0.03841	966	- && Q5ZL33.2 RecName: Full=Serine-threonine kinase receptor-associated protein && PF00400:WD domain, G-beta repeat
Lachesis_group0__13_contigs__l.g5320	995.9752	496.0033	1.005453	0.005305	0.046398	1296	- && P78712.2 RecName: Full=Actin-related protein 3; AltName: Full=Actin-like protein 3 && PF00022:Actin
Lachesis_group0__13_contigs__l.g4981	21738.64	45259.5	-1.05796	0.0038	0.036509	4215	- && Q7S4N5.2 RecName: Full=Metacaspase-1B; Flags: Precursor && PF00656:Caspase domain
Lachesis_group0__13_contigs__l.g7325	1690.514	3525.364	-1.06036	0.003413	0.033628	1116	- && Q00319.1 RecName: Full=Peroxisomal membrane protein PMP47B && PF00153:Mitochondrial carrier protein
Lachesis_group0__13_contigs__l.g2842	1612.112	3369.659	-1.06362	0.002232	0.024194	1167	- && Q9Y7N4.1 RecName: Full=D-amino-acid oxidase; Short=DAAO; Short=DAMOX; Short=DAO && PF01266:FAD dependent oxidoreductase
Lachesis_group0__13_contigs__l.g7876	816.9356	1722.522	-1.07612	0.003464	0.033959	669	- && - && PF15370:Domain of unknown function (DUF4598)
Lachesis_group0__13_contigs__l.g5452	6292.551	13276.01	-1.07711	0.00457	0.041747	1326	- && - && PF03547:Membrane transport protein
Lachesis_group0__13_contigs__l.g1069	342.4022	750.8856	-1.13268	0.004206	0.03936	2463	- && O74402.1 RecName: Full=Heat shock protein 78, mitochondrial; Flags: Precursor && PF07724:AAA domain (Cdc48 subfamily)|PF00004:ATPase family associated with

							various cellular activities (AAA)|PF10431:C-terminal, D2-small domain, of ClpB protein
novel.736	798.3081	1769.036	-1.14782	0.004524	0.041555	7168	- && P55080.1 RecName: Full=Microfibrillar-associated protein 1; AltName: Full=Associated microfibril protein; Short=AMF && PF06991:Microfibril-associated/Pre-mRNA processing
Lachesis_group0__13_contigs__l.g6883	1311.283	2914.918	-1.15237	0.003057	0.030879	3471	- && P0CT24.1 RecName: Full=Glucosidase 2 subunit beta; AltName: Full=Alpha-glucosidase 2 subunit beta; Flags: Precursor && PF12999:Glucosidase II beta subunit-like|PF13015:Glucosidase II beta subunit-like protein
Lachesis_group0__13_contigs__l.g11405	727.1338	1625.335	-1.16065	0.004322	0.040123	906	- && - && PF05705:Eukaryotic protein of unknown function (DUF829)
Lachesis_group0__13_contigs__l.g2805	233.9302	523.9479	-1.16287	0.00265	0.027675	6588	- && Q8VD65.3 RecName: Full=Phosphoinositide 3-kinase regulatory subunit 4; Short=PI3-kinase regulatory subunit 4 && PF00400:WD domain, G-beta repeat|PF00069:Protein kinase domain
Lachesis_group0__13_contigs__l.g7712	186.1449	418.1987	-1.16787	0.003725	0.035917	852	- && G4V4T7.1 RecName: Full=Enoyl-CoA-hydratase && PF00378:Enoyl-CoA hydratase/isomerase
Lachesis_group0__13_contigs__l.g7264	1533.555	3452.655	-1.17089	0.002157	0.023733	1572	- && Q96HV5.1 RecName: Full=Transmembrane protein 41A; Flags: Precursor && PF09335:SNARE associated Golgi protein
Lachesis_group0__13_contigs__l.g6863	1060.088	2405.487	-1.18209	0.000951	0.013515	837	- && - && PF00505:HMG (high mobility group) box

Lachesis_group0__13_contigs__l.g6591	688.0765	1561.637	-1.18234	0.003057	0.030879	3840	- && - && PF04937:Protein of unknown function (DUF 659)|PF05615:Tho complex subunit 7|PF05699:hAT family C-terminal dimerisation region
Lachesis_group0__13_contigs__l.g11231	328.2643	746.8861	-1.18636	0.004243	0.039585	9282	- && Q07878.1 RecName: Full=Vacuolar protein sorting-associated protein 13; AltName: Full=Suppression of the onset of impotence protein 1; AltName: Full=Vacuolar protein-targeting protein 2 && PF09333:Autophagy-related protein C terminal domain|PF06650:SHR-binding domain of vacuolar-sorting associated protein 13|PF12624:N-terminal region of Chorein or VPS13|PF16910:Repeating coiled region of VPS13|PF16909:Vacuolar-sorting-associated 13 protein C-terminal|PF16908:Vacuolar sorting-associated protein 13, N-terminal
Lachesis_group0__13_contigs__l.g10514	121.4262	276.3476	-1.18665	0.005572	0.048112	1143	- && Q9HTE3.1 RecName: Full=Glutathione-independent formaldehyde dehydrogenase; Short=FALDH; Short=FDH && PF08240:Alcohol dehydrogenase GroES-like domain|PF00107:Zinc-binding dehydrogenase
Lachesis_group0__13_contigs__l.g458	251.5389	572.5149	-1.18678	0.004329	0.040152	6915	- && P9WQE7.1 RecName: Full=Phthiocerol synthesis polyketide synthase type I PpsA; AltName: Full=Beta-ketoacyl-acyl-carrier-protein synthase I && PF00698:Acyl transferase domain|PF02801:Beta-ketoacyl synthase, C-terminal domain|PF08659:KR

							domain|PF07993:Male sterility protein|PF14765:Polyketide synthase dehydratase|PF00109:Beta-ketoacyl synthase, N-terminal domain
Lachesis_group0__13_contigs__l.g3882	416.8991	966.4423	-1.21295	0.00244	0.025975	1389	- && O15975.1 RecName: Full=Guanine nucleotide-binding protein G(q) subunit alpha; AltName: Full=Guanine nucleotide-binding protein alpha-q && PF00503:G-protein alpha subunit
Lachesis_group0__13_contigs__l.g638	748.3945	1735.702	-1.21365	0.002297	0.024758	1788	- && - && PF15786:PET assembly of cytochrome c oxidase, mitochondrial
Lachesis_group0__13_contigs__l.g5913	133.9406	311.945	-1.21977	0.004397	0.040677	1671	- && Q0D076.1 RecName: Full=Probable mannosyl-oligosaccharide alpha-1,2-mannosidase 1B; AltName: Full=Class I alpha-mannosidase 1B; AltName: Full=Man(9)-alpha-mannosidase 1B; Flags: Precursor && PF01532:Glycosyl hydrolase family 47
Lachesis_group0__13_contigs__l.g8446	3192.53	7438.82	-1.22042	0.002561	0.026905	4347	- && Q09891.1 RecName: Full=Putative phospholipid-transporting ATPase C24B11.12c && PF12710:haloacid dehalogenase-like hydrolase|PF16212:Phospholipid-translocating P-type ATPase C-terminal|PF16209:Phospholipid-translocating ATPase N-terminal|PF13246:Cation transport ATPase (P-type)
Lachesis_group0__13_contigs__l.g10137	752.0606	1753.537	-1.22153	0.005642	0.0485	1158	- && O13437.1 RecName: Full=Formate dehydrogenase; Short=FDH; AltName: Full=NAD-dependent formate dehydrogenase && PF00389:D-

							isomer specific 2-hydroxyacid dehydrogenase, catalytic domain|PF02826:D-isomer specific 2-hydroxyacid dehydrogenase, NAD binding domain
Lachesis_group0__13_contigs__l.g8852	1076.344	2519.584	-1.22719	0.002264	0.024453	1977	- && Q8K194.1 RecName: Full=U4/U6.U5 small nuclear ribonucleoprotein 27 kDa protein; Short=U4/U6.U5 snRNP 27 kDa protein; Short=U4/U6.U5-27K; AltName: Full=U4/U6.U5 tri-snRNP-associated protein 3 && PF08648:Protein of unknown function (DUF1777)
Lachesis_group0__13_contigs__l.g5914	4489.463	10527.79	-1.2296	0.001056	0.014359	3783	- && Q5BGR2.2 RecName: Full=Protein mesA && PF07792:Docking domain of Afi1 for Arf3 in vesicle trafficking|PF00656:Caspase domain
Lachesis_group0__13_contigs__l.g9741	296.4276	697.8437	-1.23503	0.001072	0.014484	1923	- && O34718.1 RecName: Full=Major myo-inositol transporter IolT && PF00083:Sugar (and other) transporter
Lachesis_group0__13_contigs__l.g8095	103.9349	245.0645	-1.23844	0.005291	0.046319	3261	- && - && PF08596:Lethal giant larvae(Lgl) like, C-terminal
Lachesis_group0__13_contigs__l.g4691	1509.72	3570.262	-1.24179	0.005689	0.048832	1806	- && S0EEY7.1 RecName: Full=Efflux pump FUS6; AltName: Full=Fusarin biosynthesis protein 6 && PF07690:Major Facilitator Superfamily
Lachesis_group0__13_contigs__l.g2906	248.9748	589.7884	-1.24416	0.004519	0.041555	1617	- && P17549.1 RecName: Full=Benzoate 4-monooxygenase; AltName: Full=Benzoate-para-hydroxylase; Short=BpH; AltName: Full=Cytochrome P450 53 && PF00067:Cytochrome P450

Lachesis_group0__13_contigs__l.g11653	187.5951	445.9684	-1.24985	0.002695	0.027966	3237	- && Q99385.1 RecName: Full=Vacuolar calcium ion transporter; AltName: Full=High copy number undoes manganese protein 1; AltName: Full=Manganese resistance 1 protein; AltName: Full=Vacuolar Ca(2+)/H(+) exchanger && PF02146:Sir2 family|PF01699:Sodium/calcium exchanger protein
novel.1007	1160.193	2786.709	-1.26426	0.003322	0.033005	5725	- && P65824.1 RecName: Full=Putative hydrolase Mb2248c; Flags: Precursor >P9WHR2.1 RecName: Full=Carboxylesterase A; Flags: Precursor >P9WHR3.1 RecName: Full=Carboxylesterase A; Flags: Precursor && -
Lachesis_group0__13_contigs__l.g8262	240.0698	580.0059	-1.27209	0.003139	0.031488	5529	- && - && PF13191:AAA ATPase domain
Lachesis_group0__13_contigs__l.g5773	97.3698	235.3445	-1.2741	0.002379	0.025527	2262	- && Q7Z9M8.1 RecName: Full=Xyloglucanase; Short=XG; AltName: Full=Cel74a; Flags: Precursor && -
Lachesis_group0__13_contigs__l.g8350	401.318	971.7653	-1.27584	0.000416	0.007501	3249	- && O43122.1 RecName: Full=Hydrophobin-B; Flags: Precursor [Agaricus bisporus] && PF00106:short chain dehydrogenase|PF01185:Fungal hydrophobin|PF12697:Alpha/beta hydrolase family
Lachesis_group0__13_contigs__l.g6108	127.3365	309.7441	-1.28238	0.001097	0.014667	2496	- && P55487.1 RecName: Full=Uncharacterized monooxygenase y4iD && PF13738:Pyridine nucleotide-disulphide oxidoreductase

Lachesis_group0__13_contigs__l.g2384	176.142	429.8537	-1.28718	0.002942	0.030062	1905	- && O31826.1 RecName: Full=Putative acyl-CoA synthetase YngI && PF13193:AMP-binding enzyme C-terminal domain|PF00501:AMP-binding enzyme
Lachesis_group0__13_contigs__l.g8297	57.75286	141.394	-1.29199	0.00569	0.048832	1128	- && O14082.2 RecName: Full=Uncharacterized protein UNK4.17 && PF01408:Oxidoreductase family, NAD-binding Rossmann fold|PF08635:Putative oxidoreductase C terminal
Lachesis_group0__13_contigs__l.g9826	161.2957	395.0779	-1.29232	0.000946	0.013469	2910	- && O00061.1 RecName: Full=Cytochrome P450 67; AltName: Full=CYPLXVII; AltName: Full=Planta-induced rust protein 16, partial [Uromyces viciae-fabae] && PF00067:Cytochrome P450
Lachesis_group0__13_contigs__l.g5303	148.3706	363.6807	-1.29313	0.002016	0.022589	2649	- && Q01373.1 RecName: Full=Peroxisomal hydratase-dehydrogenase-epimerase; Short=HDE; AltName: Full=Multifunctional beta-oxidation protein; Short=MFP; Includes: RecName: Full=2-enoyl-CoA hydratase; Includes: RecName: Full=(3R)-3-hydroxyacyl-CoA dehydrogenase && PF00106:short chain dehydrogenase|PF01575:MaoC like domain
Lachesis_group0__13_contigs__l.g6679	391.8194	970.0999	-1.30776	0.00094	0.013441	1143	- && - && PF03227:Gamma interferon inducible lysosomal thiol reductase (GILT)|PF09774:Caffeine-induced death protein 2
Lachesis_group0__13_contigs__l.g8291	19422.83	48200.91	-1.31131	0.000163	0.003649	5076	- && Q0DV32.2 RecName: Full=4-coumarate--CoA ligase-like 1 && PF00501:AMP-binding enzyme|PF00441:Acyl-CoA dehydrogenase, C-terminal domain|PF02353:Mycolic acid

							cyclopropane synthetase|PF02770:Acyl-CoA dehydrogenase, middle domain|PF13193:AMP-binding enzyme C-terminal domain
Lachesis_group0__13_contigs__l.g2807	27296.84	68538.13	-1.32817	0.000732	0.01108	570	- && - && PF00011:Hsp20/alpha crystallin family
Lachesis_group0__13_contigs__l.g3340	177.9998	449.7558	-1.33745	0.005213	0.045908	780	- && - && PF00106:short chain dehydrogenase
Lachesis_group0__13_contigs__l.g932	329.4322	833.1672	-1.33858	0.000997	0.013946	801	- && - && PF13279:Thioesterase-like superfamily
Lachesis_group0__13_contigs__l.g11378	288.8268	731.5499	-1.34049	0.003068	0.030927	1128	- && - && PF01485:IBR domain, a half RING-finger domain
Lachesis_group0__13_contigs__l.g9829	467.8157	1202.307	-1.36169	0.000146	0.003375	1815	- && Q08970.2 RecName: Full=Mitochondrial metal transporter 2; Flags: Precursor && PF01545:Cation efflux family
Lachesis_group0__13_contigs__l.g7505	209.2177	540.8106	-1.37006	0.001637	0.019315	831	- && - && PF00651:BTB/POZ domain
Lachesis_group0__13_contigs__l.g4295	134.6192	348.8251	-1.37316	0.002867	0.029464	1776	- && B2SH83.1 RecName: Full=Dihydroxy-acid dehydratase; Short=DAD && PF00920:Dehydratase family
Lachesis_group0__13_contigs__l.g11329	350.7165	908.6175	-1.37343	0.001904	0.021634	1542	- && P09437.2 RecName: Full=Cytochrome b2, mitochondrial; AltName: Full=L-lactate dehydrogenase [Cytochrome]; AltName: Full=L-lactate ferricytochrome C oxidoreductase; Short=L-LCR; Flags: Precursor && PF00173:Cytochrome b5-like Heme/Steroid binding domain|PF01070:FMN-dependent dehydrogenase
Lachesis_group0__13_contigs__l.g590	2052.046	5323.512	-1.37538	0.002067	0.022961	5571	- && Q5B4Z3.2 RecName: Full=Cytokinesis protein sepH; AltName: Full=Serine/threonine-protein kinase sepH && PF00069:Protein kinase domain|PF00307:Calponin homology (CH)

							domain|PF00130:Phorbol esters/diacylglycerol binding domain (C1 domain)
Lachesis_group0__13_contigs__l.g677	154.5252	401.1377	-1.37629	0.00115	0.01511	1878	- && P15245.3 RecName: Full=Phenol 2-monooxygenase; AltName: Full=Phenol hydroxylase && PF07976:Phenol hydroxylase, C-terminal dimerisation domain|PF01494:FAD binding domain
Lachesis_group0__13_contigs__l.g9474	136.205	353.8482	-1.37821	0.004575	0.041747	1383	- && B5XF11.1 RecName: Full=Lysine-specific demethylase 8; AltName: Full=JmjC domain-containing protein 5; AltName: Full=Jumonji domain-containing protein 5 [Salmo salar] && PF13621:Cupin-like domain
Lachesis_group0__13_contigs__l.g9104	66.2972	172.9759	-1.3832	0.00303	0.030686	2031	- && - && PF12862:Anaphase-promoting complex subunit 5
Lachesis_group0__13_contigs__l.g4035	1159.525	3025.172	-1.38348	0.001871	0.021331	2631	- && Q9DCN1.1 RecName: Full=Peroxisomal NADH pyrophosphatase NUDT12; AltName: Full=Nucleoside diphosphate-linked moiety X motif 12; Short=Nudix motif 12 && PF00293:NUDIX domain|PF09296:NADH pyrophosphatase-like rudimentary NUDIX domain
Lachesis_group0__13_contigs__l.g5954	420.0407	1096.135	-1.38359	0.00018	0.003936	702	- && Q6PAV8.1 RecName: Full=O-acetyl-ADP-ribose deacetylase MACROD2; AltName: Full=MACRO domain-containing protein 2; AltName: Full=[Protein ADP-ribosylglutamate] hydrolase && PF01661:Macro domain

Lachesis_group0__13_contigs__l.g7430	117.4483	306.5026	-1.38462	0.003291	0.032763	1614	- && Q9C0V8.1 RecName: Full=Uncharacterized transporter PB10D8.01 && PF07690:Major Facilitator Superfamily
Lachesis_group0__13_contigs__l.g7442	179.569	473.4254	-1.39945	0.00287	0.029469	1104	- && Q148L6.1 RecName: Full=Trans-1,2-dihydrobenzene-1,2-diol dehydrogenase; AltName: Full=D-xylose 1-dehydrogenase; AltName: Full=D-xylose-NADP dehydrogenase; AltName: Full=Dimeric dihydrodiol dehydrogenase && PF01408:Oxidoreductase family, NAD-binding Rossmann fold
Lachesis_group0__13_contigs__l.g6553	317.1587	838.707	-1.40308	0.000296	0.005823	10668	- && P38811.1 RecName: Full=Transcription-associated protein 1; AltName: Full=p400 kDa component of SAGA && PF02259:FAT domain|PF00454:Phosphatidylinositol 3- and 4-kinase
Lachesis_group0__13_contigs__l.g2129	103.6643	273.934	-1.40316	0.003374	0.033304	1668	- && Q10286.1 RecName: Full=Myo-inositol transporter 1 && PF00083:Sugar (and other) transporter
Lachesis_group0__13_contigs__l.g5603	121.6808	322.1497	-1.40521	0.000603	0.009716	1152	- && - && PF07818:HCNGP-like protein
Lachesis_group0__13_contigs__l.g4892	502.3382	1335.928	-1.41099	0.000563	0.009299	1104	- && Q5ZID0.1 RecName: Full=NmrA-like family domain-containing protein 1 && PF05368:NmrA-like family
Lachesis_group0__13_contigs__l.g10516	366.25	976.1422	-1.41463	0.000578	0.009478	1605	- && Q99252.1 RecName: Full=Protein ECM3; AltName: Full=Extracellular mutant protein 3 && -
Lachesis_group0__13_contigs__l.g489	202.2622	544.8562	-1.42982	0.000254	0.005214	4179	- && - && PF12937:F-box-like

Lachesis_group0__13_contigs__l.g2006	129.5553	349.3409	-1.43136	0.000549	0.009098	1635	- && P08092.2 RecName: Full=Negative regulator of sexual conjugation and meiosis && PF00069:Protein kinase domain
Lachesis_group0__13_contigs__l.g3650	100.5589	271.7616	-1.43415	0.000653	0.010202	1347	- && A2QBQ3.1 RecName: Full=Probable endo-1,3(4)-beta-glucanase An02g00850; AltName: Full=Mixed-linked glucanase An02g00850; Flags: Precursor && PF00652:Ricin-type beta-trefoil lectin domain
Lachesis_group0__13_contigs__l.g3651	284.1852	772.9146	-1.44387	0.000342	0.006464	1317	- && P75791.1 RecName: Full=Uncharacterized protein YbiU && PF07350:Protein of unknown function (DUF1479)
Lachesis_group0__13_contigs__l.g1318	3649.528	9939.102	-1.44545	0.001701	0.019823	1995	- && A2QEQ6.1 RecName: Full=Beta-glucuronidase; Short=GlcAase; AltName: Full=Beta-D-glucuronoside glucuronosohydrolase; Flags: Precursor && PF16862:Glycosyl hydrolase family 79 C-terminal beta domain
Lachesis_group0__13_contigs__l.g11123	34005.55	92687.56	-1.4466	0.000149	0.003426	2772	- && - && PF00564:PB1 domain
Lachesis_group0__13_contigs__l.g1534	550.2828	1500.837	-1.44767	0.00021	0.004454	5208	- && Q9UUG9.1 RecName: Full=Tuberous sclerosis 2 protein homolog && PF03542:Tuberin|PF02145:Rap/ran-GAP
Lachesis_group0__13_contigs__l.g765	257.4852	702.7854	-1.44889	0.004791	0.043207	1395	- && P25390.2 RecName: Full=Serine/threonine-protein kinase SSK22; AltName: Full=MAP kinase kinase kinase SSK22; AltName: Full=Suppressor of sensor kinase 22 && PF07714:Protein tyrosine kinase
Lachesis_group0__13_contigs__l.g11203	298.2343	814.7244	-1.44958	0.001233	0.015777	681	- && - && PF01975:Survival protein SurE
Lachesis_group0__13_contigs__l.g8784	124.2454	339.8703	-1.45229	0.001758	0.020358	4407	- && - && PF05729:NACHT domain

Lachesis_group0__13_contigs__l.g6429	3878.048	10619.87	-1.45338	0.00187	0.021331	1953	- && O59714.1 RecName: Full=Uncharacterized membrane protein C3B8.06 && PF10348:Domain of unknown function (DUF2427)|PF10355:Protein of unknown function (Ytp1)
Lachesis_group0__13_contigs__l.g10550	159.7294	439.0623	-1.45883	0.000184	0.004005	2910	- && P29064.1 RecName: Full=Alpha-glucosidase; AltName: Full=Maltase; Contains: RecName: Full=Alpha-glucosidase subunit 1; Contains: RecName: Full=Alpha-glucosidase subunit 2; Flags: Precursor && PF16863:N-terminal barrel of NtMGAM and CtMGAM, maltase-glucoamylase|PF01055:Glycosyl hydrolases family 31
Lachesis_group0__13_contigs__l.g571	126.9846	351.6573	-1.47042	0.001151	0.01511	1833	- && O13902.1 RecName: Full=Dihydroxyacetone kinase 1; Short=DHA kinase 1; AltName: Full=Glycerone kinase 1; AltName: Full=Triokinase 1; AltName: Full=Triose kinase 1 && PF02734:DAK2 domain|PF02733:Dak1 domain
Lachesis_group0__13_contigs__l.g678	1455.633	4056.315	-1.47854	0.000125	0.003006	1725	- && P15245.3 RecName: Full=Phenol 2-monooxygenase; AltName: Full=Phenol hydroxylase && PF07976:Phenol hydroxylase, C-terminal dimerisation domain|PF01494:FAD binding domain
Lachesis_group0__13_contigs__l.g409	10506.63	29531.23	-1.49094	2.15E-05	0.000814	1188	- && - && PF07992:Pyridine nucleotide-disulphide oxidoreductase
Lachesis_group0__13_contigs__l.g9060	410.4988	1156.636	-1.49458	0.000258	0.005273	1677	- && P55809.1 RecName: Full=Succinyl-CoA:3-ketoacid coenzyme A transferase 1, mitochondrial;

							AltName: Full=3-oxoacid CoA-transferase 1; AltName: Full=Somatic-type succinyl-CoA:3-oxoacid CoA-transferase; Short=SCOT-s; Flags: Precursor && PF01144:Coenzyme A transferase
Lachesis_group0__13_contigs__l.g11709	2094.881	5923.524	-1.49962	0.001757	0.020358	2859	- && - && PF07217:Heterokaryon incompatibility protein Het-C
Lachesis_group0__13_contigs__l.g2843	7554.792	21383.19	-1.50102	0.000477	0.008254	1518	- && Q9URW6.1 RecName: Full=SH3 domain-containing protein PJ696.02 && PF04366:Las17-binding protein actin regulator|PF00018:SH3 domain
Lachesis_group0__13_contigs__l.g7258	3964.752	11227.68	-1.50179	0.000164	0.003649	3924	- && P53721.1 RecName: Full=Respiratory supercomplex factor 2, mitochondrial; AltName: Full=Altered inheritance of mitochondria protein 38 && -
Lachesis_group0__13_contigs__l.g10877	884.0265	2509.528	-1.50546	0.001534	0.018579	1512	- && O13820.3 RecName: Full=Cytochrome P450 61; AltName: Full=C-22 sterol desaturase && PF00067:Cytochrome P450
Lachesis_group0__13_contigs__l.g6467	573.8029	1630.045	-1.50648	0.000608	0.009747	1848	- && Q3KJ31.1 RecName: Full=Lipid A export ATP-binding/permease protein MsbA && PF00005:ABC transporter
Lachesis_group0__13_contigs__l.g2911	409.5011	1167.121	-1.5113	0.003665	0.035475	2112	- && - && PF00010:Helix-loop-helix DNA-binding domain
Lachesis_group0__13_contigs__l.g9163	88.95591	254.1657	-1.51519	0.001169	0.015186	4614	- && - && PF02373:JmjC domain, hydroxylase
Lachesis_group0__13_contigs__l.g1720	43.90479	125.4115	-1.51604	0.002174	0.023896	885	- && B8NM67.1 RecName: Full=Oxidase ustYa; AltName: Full=Ustiloxin B biosynthesis protein Ya

							&& PF11807:Domain of unknown function (DUF3328)
Lachesis_group0__13_contigs__l.g11062	2374.503	6801.353	-1.51824	0.001643	0.019357	2691	- && - && PF13886:Domain of unknown function (DUF4203)
Lachesis_group0__13_contigs__l.g5699	277.7588	796.9306	-1.52024	0.00327	0.032585	1209	- && Q7SDR1.1 RecName: Full=Endoglucanase gh5-1; AltName: Full=Cellulase gh5-1; AltName: Full=Endo-1,4-beta-glucanase gh5-1; Flags: Precursor && PF00734:Fungal cellulose binding domain|PF00150:Cellulase (glycosyl hydrolase family 5)
Lachesis_group0__13_contigs__l.g2033	135.9752	390.1958	-1.52164	0.001432	0.017716	5091	- && Q9P7V2.1 RecName: Full=ATP-binding cassette transporter abc4; Short=ABC transporter abc4; AltName: Full=ATP-energized glutathione S-conjugate pump abc4; AltName: Full=Glutathione S-conjugate-transporting ATPase abc4 && PF00005:ABC transporter|PF00664:ABC transporter transmembrane region
Lachesis_group0__13_contigs__l.g10091	392.1955	1126.249	-1.52205	0.002291	0.024717	2415	- && Q59KI0.1 RecName: Full=UTP--glucose-1-phosphate uridylyltransferase; AltName: Full=UDP-glucose pyrophosphorylase; Short=UDPGP; Short=UGPase && PF00106:short chain dehydrogenase|PF01704:UTP--glucose-1-phosphate uridylyltransferase
Lachesis_group0__13_contigs__l.g9589	30.62713	88.11057	-1.52233	0.003527	0.034359	819	- && B5ZA76.1 RecName: Full=Peptidoglycan deacetylase; Short=PG deacetylase; AltName:

							Full=Acetylxylan esterase && PF01522:Polysaccharide deacetylase
Lachesis_group0__13_contigs__l.g9971	798.929	2297.518	-1.52399	0.003034	0.030705	1428	- && - && PF03595:Voltage-dependent anion channel
Lachesis_group0__13_contigs__l.g8439	1602.94	4618.67	-1.52676	0.001841	0.021057	1104	- && - && PF00134:Cyclin, N-terminal domain
Lachesis_group0__13_contigs__l.g2385	60.44586	175.161	-1.5354	0.001008	0.014013	1035	- && Q4R0J7.1 RecName: Full=D-arabinitol dehydrogenase 1; AltName: Full=NADP-dependent D-arabitol dehydrogenase && PF16912:Glucose dehydrogenase C-terminus|PF08240:Alcohol dehydrogenase GroES-like domain
Lachesis_group0__13_contigs__l.g10169	31.32572	90.8491	-1.53558	0.002391	0.025554	1794	- && - && PF00646:F-box domain
Lachesis_group0__13_contigs__l.g6803	95.19066	276.4382	-1.53777	0.00111	0.014755	933	- && Q9JLZ3.1 RecName: Full=Methylglutaconyl-CoA hydratase, mitochondrial; AltName: Full=AU-specific RNA-binding enoyl-CoA hydratase; Short=AU-binding enoyl-CoA hydratase; Short=muAUH; Flags: Precursor && PF00378:Enoyl-CoA hydratase/isomerase
Lachesis_group0__13_contigs__l.g7261	37.94469	110.674	-1.54438	0.002998	0.030423	1872	- && - && PF01965:DJ-1/PfpI family
Lachesis_group0__13_contigs__l.g1377	164.3615	480.1402	-1.54735	0.00049	0.00838	3651	- && A0A0D1E9B9.1 RecName: Full=Spore development regulator umv1 && PF11754:Velvet factor
Lachesis_group0__13_contigs__l.g1998	60.40709	177.3273	-1.55282	0.000595	0.00962	1698	- && P08158.1 RecName: Full=Acetamidase && PF01425:Amidase
Lachesis_group0__13_contigs__l.g11040	592.2398	1738.084	-1.553	0.001097	0.014667	2682	- && Q9P7G8.1 RecName: Full=UPF0382 membrane protein C1782.12c; Flags: Precursor && PF04241:Protein of unknown function (DUF423)

Lachesis_group0__13_contigs__l.g8748	133.4021	391.4588	-1.55363	0.002381	0.025527	510	- && - && PF08583:Cytochrome c oxidase biogenesis protein Cmc1 like
Lachesis_group0__13_contigs__l.g10906	66.3884	195.4723	-1.55623	0.001681	0.019631	867	- && P25170.1 RecName: Full=Multifunctional tryptophan biosynthesis protein; Includes: RecName: Full=Anthranilate synthase component 2; Short=AS; AltName: Full=Anthranilate synthase, glutamine amidotransferase component; Includes: RecName: Full=Indole-3-glycerol phosphate synthase; Short=IGPS; Includes: RecName: Full=N-(5'-phosphoribosyl)anthranilate isomerase; Short=PRAI && PF00218:Indole-3-glycerol phosphate synthase
Lachesis_group0__13_contigs__l.g9385	464.4794	1366.658	-1.55688	0.001607	0.019088	870	- && P06632.3 RecName: Full=2,5-diketo-D-gluconic acid reductase A; Short=2,5-DKG reductase A; Short=2,5-DKGR A; Short=25DKGR-A; AltName: Full=AKR5C && PF00248:Aldo/keto reductase family
Lachesis_group0__13_contigs__l.g4384	47.47793	139.8204	-1.56086	0.003466	0.033959	1506	- && Q4WAZ6.1 RecName: Full=Multifunctional cytochrome P450 monooxygenase af510; AltName: Full=Fumagillin bioynthesis cluster P450 monooxygenase; Short=Fma-P450 && PF00067:Cytochrome P450
Lachesis_group0__13_contigs__l.g6900	1045.742	3095.836	-1.56593	0.001317	0.01661	1515	- && Q92407.1 RecName: Full=Glucokinase; AltName: Full=Glucose kinase; Short=GLK && PF00349:Hexokinase|PF03727:Hexokinase

Lachesis_group0__13_contigs__l.g452	143.756	425.8493	-1.56611	0.000953	0.013515	2142	- && Q9UT59.1 RecName: Full=Putative uncharacterized oxidoreductase C513.07 && PF01370:NAD dependent epimerase/dehydratase family
Lachesis_group0__13_contigs__l.g5941	121.5189	361.0235	-1.57052	0.000126	0.003012	1608	- && Q4U3U4.1 RecName: Full=Quinate permease; AltName: Full=Quinate transporter && PF00083:Sugar (and other) transporter
Lachesis_group0__13_contigs__l.g10701	63.26335	188.3674	-1.57577	0.001482	0.018102	2664	- && Q09803.1 RecName: Full=Suppressor protein of bem1/bed5 double mutants && PF04212:MIT (microtubule interacting and transport) domain|PF07714:Protein tyrosine kinase
Lachesis_group0__13_contigs__l.g483	36.68808	109.6449	-1.57663	0.005309	0.046398	1689	- && - && PF12937:F-box-like
Lachesis_group0__13_contigs__l.g7481	1673.13	5006.1	-1.58118	0.001391	0.017335	2475	- && - && PF04082:Fungal specific transcription factor domain
Lachesis_group0__13_contigs__l.g5150	848.4803	2542.549	-1.58323	0.000637	0.010051	2967	- && P0CS23.1 RecName: Full=Urease; AltName: Full=Urea amidohydrolase >P0CS22.1 RecName: Full=Urease; AltName: Full=Urea amidohydrolase && PF00547:Urease, gamma subunit|PF01979:Amidohydrolase family|PF00449:Urease alpha-subunit, N-terminal domain|PF00699:Urease beta subunit
Lachesis_group0__13_contigs__l.g1062	3239.096	9710.574	-1.58398	5.81E-05	0.001715	4593	- && P55201.2 RecName: Full=Peregrin; AltName: Full=Bromodomain and PHD finger-containing protein 1; AltName: Full=Protein Br140 && PF10513:Enhancer of polycomb-like|PF13831:PHD-

							finger|PF00439:Bromodomain|PF13832:PHD-zinc-finger like domain|PF00855:PWWP domain
Lachesis_group0__13_contigs__l.g108	418.9801	1257.22	-1.58515	0.001906	0.021635	945	- && - && PF04479:RTA1 like protein
Lachesis_group0__13_contigs__l.g3045	85.12486	255.6026	-1.5859	0.000719	0.010959	975	- && Q00023.1 RecName: Full=Cellulose-growth-specific protein; Flags: Precursor && PF03443:Glycosyl hydrolase family 61|PF00734:Fungal cellulose binding domain
Lachesis_group0__13_contigs__l.g9100	50.67244	152.3849	-1.58851	0.000546	0.00908	687	- && Q00719.1 RecName: Full=O-methyltransferase MdmC && PF01596:O-methyltransferase
Lachesis_group0__13_contigs__l.g3729	92.23263	277.1632	-1.58855	0.0027	0.027975	1599	- && O74969.1 RecName: Full=High-affinity glucose transporter ght2; AltName: Full=Hexose transporter 2 && PF00083:Sugar (and other) transporter
Lachesis_group0__13_contigs__l.g7355	289.1653	871.7863	-1.59219	3.28E-05	0.001111	930	- && - && PF00106:short chain dehydrogenase
Lachesis_group0__13_contigs__l.g7319	308.1243	933.2312	-1.5987	0.00071	0.010846	1113	- && P9WMU2.1 RecName: Full=Probable glycerophosphoryl diester phosphodiesterase 1; Short=Glycerophosphodiester phosphodiesterase 1 >P9WMU3.1 RecName: Full=Probable glycerophosphoryl diester phosphodiesterase 1; Short=Glycerophosphodiester phosphodiesterase 1 && PF03009:Glycerophosphoryl diester phosphodiesterase family
Lachesis_group0__13_contigs__l.g710	47.95216	145.2018	-1.59989	0.004528	0.041556	2427	- && - && PF00069:Protein kinase domain
Lachesis_group0__13_contigs__l.g3645	2511.129	7623.322	-1.60214	0.004827	0.043412	423	- && - && PF14200:Ricin-type beta-trefoil lectin domain-like
Lachesis_group0__13_contigs__l.g5509	433.0234	1315.445	-1.60306	0.001373	0.017153	1146	- && P0DMQ6.1 RecName: Full=Sorbitol dehydrogenase; AltName: Full=L-iditol 2-

							dehydrogenase && PF08240:Alcohol dehydrogenase GroES-like domain|PF00107:Zinc-binding dehydrogenase
Lachesis_group0__13_contigs__l.g11460	445.3839	1353.386	-1.60319	2.50E-05	0.000914	651	- && - && PF00385:Chromo (CHRromatin Organisation MOdifier) domain
Lachesis_group0__13_contigs__l.g6053	1346.441	4093.93	-1.60438	3.73E-05	0.001215	660	- && P40581.1 RecName: Full=Peroxiredoxin HYR1; AltName: Full=Glutathione peroxidase 3; AltName: Full=Hydrogen peroxide resistance protein 1; AltName: Full=Oxidant receptor peroxidase 1; AltName: Full=Phospholipid hydroperoxide glutathione peroxidase 3; Short=PHGPx3 && PF00255:Glutathione peroxidase
Lachesis_group0__13_contigs__l.g8747	179.7883	546.5731	-1.60464	0.000817	0.012111	2403	- && P31434.2 RecName: Full=Alpha-xylosidase && PF13802:Galactose mutarotase-like|PF01055:Glycosyl hydrolases family 31
Lachesis_group0__13_contigs__l.g5843	204.464	623.3614	-1.60829	0.005857	0.049934	1467	- && Q4WCZ8.2 RecName: Full=Probable exopolygalacturonase X; Short=ExoPG; AltName: Full=Galacturan 1,4-alpha-galacturonidase; AltName: Full=Poly(1,4-alpha-D-galacturonide)galacturonohydrolase; Flags: Precursor >B0YDE8.2 RecName: Full=Probable exopolygalacturonase X; Short=ExoPG; AltName: Full=Galacturan 1,4-alpha-galacturonidase; AltName: Full=Poly(1,4-alpha-D-galacturonide)galacturonohydrolase; Flags:

							Precursor && PF00295:Glycosyl hydrolases family 28
Lachesis_group0__13_contigs__l.g3904	475.5136	1457.084	-1.61541	0.001391	0.017335	2031	- && - && PF00561:alpha/beta hydrolase fold
Lachesis_group0__13_contigs__l.g4928	9836.645	30149.48	-1.6159	0.000446	0.00787	4515	- && Q9HDW7.1 RecName: Full=Calcium-transporting ATPase 2 && PF13246:Cation transport ATPase (P-type)|PF00122:E1-E2 ATPase|PF00690:Cation transporter/ATPase, N-terminus|PF00689:Cation transporting ATPase, C-terminus
Lachesis_group0__13_contigs__l.g721	211.078	650.1902	-1.62308	0.002367	0.025431	2298	- && O22881.2 RecName: Full=Potassium transporter 2; Short=AtKT2; Short=AtKUP2; Short=AtPOT2 && PF02705:K+ potassium transporter
Lachesis_group0__13_contigs__l.g11148	155.9318	480.6618	-1.62343	0.00163	0.019277	882	- && Q6P8M1.1 RecName: Full=Putative deoxyribonuclease TATDN1 && PF01026:TatD related DNase
Lachesis_group0__13_contigs__l.g4565	47.51864	146.2261	-1.62369	0.002675	0.027839	1416	- && Q99252.1 RecName: Full=Protein ECM3; AltName: Full=Extracellular mutant protein 3 && PF03547:Membrane transport protein
Lachesis_group0__13_contigs__l.g10294	1425.021	4418.429	-1.63264	6.89E-05	0.001975	699	- && Q9UUM7.1 RecName: Full=Protein hob3; AltName: Full=Homolog of Bin3 && PF03114:BAR domain
Lachesis_group0__13_contigs__l.g11461	248.5167	771.4258	-1.63464	0.000193	0.004167	2325	- && Q9C168.2 RecName: Full=Catalase-1 && PF01965:DJ-1/PfpI family|PF06628:Catalase-related immune-responsive|PF00199:Catalase
Lachesis_group0__13_contigs__l.g107	689.0954	2141.833	-1.63621	0.000356	0.00664	933	- && - && PF04479:RTA1 like protein

Lachesis_group0__13_contigs__l.g1568	38.75077	120.3385	-1.63726	0.002505	0.026531	894	- && - && PF08719:Domain of unknown function (DUF1768)
Lachesis_group0__13_contigs__l.g5830	87.70083	272.5914	-1.63734	0.000325	0.006264	951	- && Q3ZFI7.1 RecName: Full=D-galacturonate reductase; AltName: Full=D-galacturonic acid reductase && PF00248:Aldo/keto reductase family
Lachesis_group0__13_contigs__l.g10162	355.1364	1107.601	-1.64104	0.002097	0.023151	2127	- && Q752X0.5 RecName: Full=Peroxisomal targeting signal receptor; Short=PTS1 receptor; Short=PTS1R; AltName: Full=Peroxin-5 && PF13414:TPR repeat
Lachesis_group0__13_contigs__l.g2411	37.32295	116.2828	-1.64165	0.0018	0.020697	2346	- && P33363.2 RecName: Full=Periplasmic beta-glucosidase; AltName: Full=Beta-D-glucoside glucohydrolase; AltName: Full=Cellobiase; AltName: Full=Gentiobiase; Flags: Precursor && PF14310:Fibronectin type III-like domain|PF01915:Glycosyl hydrolase family 3 C-terminal domain|PF00933:Glycosyl hydrolase family 3 N terminal domain
Lachesis_group0__13_contigs__l.g8968	56.63703	177.0571	-1.64299	0.000544	0.009077	1074	- && Q96HA8.2 RecName: Full=Protein N-terminal glutamine amidohydrolase; AltName: Full=Protein NH2-terminal glutamine deamidase; Short=N-terminal Gln amidase; Short=Nt(Q)-amidase; AltName: Full=WDYHV motif-containing protein 1 && PF09764:N-terminal glutamine amidase
Lachesis_group0__13_contigs__l.g1354	31.98459	99.85778	-1.64406	0.00134	0.016795	1632	- && P39932.2 RecName: Full=Sugar transporter STL1 && PF00083:Sugar (and other) transporter

Lachesis_group0__13_contigs__l.g3872	41.33538	129.8783	-1.6511	0.000989	0.013859	2193	- && Q54PX0.1 RecName: Full=Probable serine/threonine-protein kinase DDB_G0284251 && PF07714:Protein tyrosine kinase
Lachesis_group0__13_contigs__l.g441	274.8579	864.4585	-1.65371	0.0009	0.013072	507	- && P56739.1 RecName: Full=Uncharacterized 21.2 kDa protein && PF08883:Dopa 4,5-dioxygenase family
Lachesis_group0__13_contigs__l.g4068	23.70291	74.62805	-1.65711	0.005089	0.045129	885	- && - && PF12695:Alpha/beta hydrolase family
Lachesis_group0__13_contigs__l.g2516	2131.866	6739.914	-1.66062	0.00206	0.022937	2052	- && P39992.1 RecName: Full=Uncharacterized protein YEL023C && PF09994:Uncharacterized alpha/beta hydrolase domain (DUF2235)
Lachesis_group0__13_contigs__l.g10337	158.4774	502.1393	-1.66417	0.000945	0.013469	555	- && Q9UTI7.1 RecName: Full=Probable thymidylate synthase; Short=TS; Short=TSase && PF02441:Flavoprotein
Lachesis_group0__13_contigs__l.g568	17546.62	55701.99	-1.66654	0.000105	0.002646	1626	- && Q12300.1 RecName: Full=High-affinity glucose transporter RGT2 && PF00083:Sugar (and other) transporter
Lachesis_group0__13_contigs__l.g4102	1015.743	3257.284	-1.68111	0.000617	0.009812	1629	- && Q05567.1 RecName: Full=Sphingosine-1-phosphate lyase; Short=S1PL; Short=SP-lyase; Short=ySPL; AltName: Full=Bestowed of sphingosine tolerance 1; AltName: Full=Sphingosine-1-phosphate aldolase && PF00282:Pyridoxal-dependent decarboxylase conserved domain
Lachesis_group0__13_contigs__l.g3733	19753.4	63358.62	-1.68145	3.45E-05	0.001146	3861	- && O74969.1 RecName: Full=High-affinity glucose transporter ght2; AltName: Full=Hexose transporter 2 && PF00083:Sugar (and other) transporter

Lachesis_group0__13_contigs__l.g2242	513.5323	1657.693	-1.69082	8.76E-06	0.00039	4188	- && O80327.1 RecName: Full=Thaumatin-like protein 1; Flags: Precursor && PF00314:Thaumatin family|PF08386:TAP-like protein|PF00561:alpha/beta hydrolase fold
Lachesis_group0__13_contigs__l.g2585	70.43915	227.3959	-1.69251	0.000593	0.009616	1092	- && Q7LL04.2 RecName: Full=UPF0676 protein C1494.01 && PF03171:2OG-Fe(II) oxygenase superfamily|PF14226:non-haem dioxygenase in morphine synthesis N-terminal
Lachesis_group0__13_contigs__l.g4906	17464.53	56478.88	-1.69329	8.34E-05	0.002278	2271	- && Q9P326.1 RecName: Full=Transcriptional regulatory protein pro1; AltName: Full=Arrested development protein 1 && PF11951:Fungal specific transcription factor domain
Lachesis_group0__13_contigs__l.g7732	62.98345	203.7426	-1.69369	0.004524	0.041555	1740	- && Q6BZP5.1 RecName: Full=2-methylisocitrate lyase, mitochondrial; Flags: Precursor && PF00463:Isocitrate lyase family
Lachesis_group0__13_contigs__l.g3858	128.8694	418.4683	-1.6991	0.002656	0.027712	1173	- && - && PF01408:Oxidoreductase family, NAD-binding Rossmann fold
Lachesis_group0__13_contigs__l.g11296	422.0943	1370.345	-1.69915	7.82E-06	0.000358	1842	- && - && PF01490:Transmembrane amino acid transporter protein
Lachesis_group0__13_contigs__l.g11140	232.9138	756.7071	-1.70004	1.14E-05	0.000481	2277	- && - && PF02838:Glycosyl hydrolase family 20, domain 2
Lachesis_group0__13_contigs__l.g4100	5784.685	18795.72	-1.7001	0.000678	0.010476	2820	- && Q09729.1 RecName: Full=Arrestin domain-containing protein C31A2.12 && PF02752:Arrestin (or S-antigen), C-terminal domain|PF00339:Arrestin (or S-antigen), N-terminal domain

Lachesis_group0__13_contigs__l.g2214	30.18802	98.0456	-1.70114	0.001117	0.014801	1140	- && Q01752.1 RecName: Full=Aryl-alcohol dehydrogenase [NADP(+)]; Short=AAD && PF00248:Aldo/keto reductase family
Lachesis_group0__13_contigs__l.g3145	657.8777	2139.997	-1.70177	0.001012	0.014054	1869	- && A1CPX0.1 RecName: Full=Probable quinate permease; AltName: Full=Quinate transporter && PF00083:Sugar (and other) transporter
Lachesis_group0__13_contigs__l.g2888	452.0916	1470.869	-1.70193	0.000492	0.008383	3771	- && P0CU32.1 RecName: Full=FAD-linked oxidoreductase CHGG_01242-2; AltName: Full=Chaetoglobosin biosynthesis protein CHGG_01242-2 && PF08031:Berberine and berberine like|PF01565:FAD binding domain|PF00069:Protein kinase domain
Lachesis_group0__13_contigs__l.g11052	7232.596	23585.67	-1.70532	0.003219	0.032142	1653	- && A0A0B5EMG9.1 RecName: Full=Efflux pump FUBT; AltName: Full=Fusaric acid biosynthesis protein T; AltName: Full=Fusaric acid transporter && PF07690:Major Facilitator Superfamily
Lachesis_group0__13_contigs__l.g2503	78.79596	257.9876	-1.70985	0.000457	0.007993	969	- && P84675.2 RecName: Full=Putative fungistatic metabolite && PF01822:WSC domain
Lachesis_group0__13_contigs__l.g8405	377.8218	1237.639	-1.71183	0.000129	0.003059	3213	- && O14283.2 RecName: Full=Transcription factor prr1; AltName: Full=Pombe response regulator 1 && PF00447:HSF-type DNA-binding
Lachesis_group0__13_contigs__l.g4708	1494.953	4897.712	-1.71201	0.00281	0.029023	1944	- && Q00614.1 RecName: Full=Carnitine O-acetyltransferase, mitochondrial; Short=Carnitine acetylase; Flags: Precursor && PF00755:Choline/Carnitine o-acyltransferase

Lachesis_group0__13_contigs__l.g5321	555.1522	1823.231	-1.71561	0.005191	0.04582	651	- && P42769.1 RecName: Full=Glutathione S-transferase PM239X14; AltName: Full=GST class-phi && PF02798:Glutathione S-transferase, N-terminal domain|PF00043:Glutathione S-transferase, C-terminal domain
Lachesis_group0__13_contigs__l.g8408	710.8845	2347.556	-1.72365	0.000171	0.00378	1881	- && O14283.2 RecName: Full=Transcription factor prr1; AltName: Full=Pombe response regulator 1 && PF00447:HSF-type DNA-binding
Lachesis_group0__13_contigs__l.g1999	47.15154	156.2177	-1.72976	0.000669	0.010411	888	- && Q49WS9.1 RecName: Full=Uncharacterized oxidoreductase SSP1627 && PF00106:short chain dehydrogenase
Lachesis_group0__13_contigs__l.g3866	68.34416	226.863	-1.73019	0.000705	0.010807	2367	- && P80235.2 RecName: Full=Putative mitochondrial carnitine O-acetyltransferase && PF00755:Choline/Carnitine o-acyltransferase
Lachesis_group0__13_contigs__l.g10260	60.36147	200.6676	-1.73207	0.000935	0.013408	2925	- && Q804S5.1 RecName: Full=E3 ubiquitin-protein ligase mib1; AltName: Full=Protein mind bomb && PF00569:Zinc finger, ZZ type
Lachesis_group0__13_contigs__l.g8349	165.5696	550.3808	-1.73353	0.000421	0.00756	756	- && P0A2C9.1 RecName: Full=3-oxoacyl-[acyl-carrier-protein] reductase FabG; AltName: Full=3-ketoacyl-acyl carrier protein reductase; AltName: Full=Beta-Ketoacyl-acyl carrier protein reductase; AltName: Full=Beta-ketoacyl-ACP reductase >P0A2D0.1 RecName: Full=3-oxoacyl-[acyl-carrier-protein] reductase FabG; AltName: Full=3-ketoacyl-acyl carrier protein reductase; AltName: Full=Beta-Ketoacyl-acyl carrier protein

							reductase; AltName: Full=Beta-ketoacyl-ACP reductase && PF00106:short chain dehydrogenase
Lachesis_group0__13_contigs__l.g8721	4507.041	15004.46	-1.73515	0.000119	0.002909	1617	- && P25390.2 RecName: Full=Serine/threonine-protein kinase SSK22; AltName: Full=MAP kinase kinase kinase SSK22; AltName: Full=Suppressor of sensor kinase 22 && PF00069:Protein kinase domain
Lachesis_group0__13_contigs__l.g6781	628.8986	2093.997	-1.73542	0.000462	0.008023	1869	- && P30608.1 RecName: Full=Cytochrome P450 52A6; AltName: Full=Alkane-inducible P450-ALK3; AltName: Full=CYPLIIA6 && PF00067:Cytochrome P450
Lachesis_group0__13_contigs__l.g10940	78.82481	262.4524	-1.73676	0.000353	0.006619	1410	- && - && PF03009:Glycerophosphoryl diester phosphodiesterase family
Lachesis_group0__13_contigs__l.g8668	1102.298	3675.648	-1.73751	0.001815	0.020802	1032	- && Q43139.2 RecName: Full=Fructose-1,6-bisphosphatase, cytosolic; Short=FBPase; AltName: Full=D-fructose-1,6-bisphosphate 1-phosphohydrolase && PF00316:Fructose-1-6-bisphosphatase
Lachesis_group0__13_contigs__l.g10448	2662.432	8912.091	-1.74303	4.47E-05	0.001411	2994	- && Q9PFB0.1 RecName: Full=Beta-lactamase hydrolase-like protein; Short=BLH && PF00753:Metallo-beta-lactamase superfamily
Lachesis_group0__13_contigs__l.g4081	68.44595	230.314	-1.75089	0.00218	0.023934	1920	- && Q20YI1.1 RecName: Full=Homoserine O-acetyltransferase; AltName: Full=Homoserine O-trans-acetylase; Short=HTA; Short=Homoserine transacetylase && PF00561:alpha/beta hydrolase fold

Lachesis_group0__13_contigs__l.g275	161.3471	543.352	-1.75104	0.00061	0.009749	2625	- && - && PF17107:N-terminal domain on NACHT_NTPase and P-loop NTPases
Lachesis_group0__13_contigs__l.g6118	130.5967	439.8999	-1.75216	0.000167	0.003697	2061	- && A1CFL2.1 RecName: Full=Dehydrogenase patE; AltName: Full=Patulin synthesis protein E; Flags: Precursor && PF05199:GMC oxidoreductase|PF00732:GMC oxidoreductase
Lachesis_group0__13_contigs__l.g7226	237.1101	801.2279	-1.75683	0.00063	0.009973	678	- && - && PF01965:DJ-1/PfpI family
Lachesis_group0__13_contigs__l.g4634	3939.51	13340.77	-1.75975	1.61E-05	0.000645	1077	- && Q8NKE2.2 RecName: Full=Alternative oxidase, mitochondrial; Flags: Precursor && PF01786:Alternative oxidase
Lachesis_group0__13_contigs__l.g7745	405.0382	1374.413	-1.76283	0.001056	0.014359	3006	- && - && PF12937:F-box-like
Lachesis_group0__13_contigs__l.g6944	111.0269	377.8014	-1.76696	0.000405	0.007346	1722	- && P08157.2 RecName: Full=Aldehyde dehydrogenase; Short=ALDDH; Short=ALDH && PF00171:Aldehyde dehydrogenase family
Lachesis_group0__13_contigs__l.g370	405.0775	1380.367	-1.76916	0.001174	0.015188	1896	- && Q0CJ61.1 RecName: Full=Efflux pump atB; AltName: Full=Terreic acid biosynthesis cluster protein B && PF07690:Major Facilitator Superfamily
Lachesis_group0__13_contigs__l.g1572	124.5246	427.9008	-1.78141	0.000589	0.009565	1032	- && Q9Y6F1.3 RecName: Full=Poly [ADP-ribose] polymerase 3; Short=PARP-3; Short=hPARP-3; AltName: Full=ADP-ribosyltransferase diphtheria toxin-like 3; Short=ARTD3; AltName: Full=IRT1; AltName: Full=NAD(+) ADP-ribosyltransferase 3; Short=ADPRT-3; AltName: Full=Poly[ADP-ribose] synthase 3; Short=pADPRT-3 && PF00644:Poly(ADP-ribose) polymerase catalytic domain

Lachesis_group0__13_contigs__l.g10730	86.09449	296.0522	-1.78269	0.000224	0.004712	2748	- && Q2U4L7.2 RecName: Full=Glutaminase A; Flags: Precursor && PF08760:Domain of unknown function (DUF1793)|PF16335:Domain of unknown function (DUF4965)
Lachesis_group0__13_contigs__l.g9068	8196.162	28316.24	-1.7886	0.005538	0.047919	1962	- && Q01877.1 RecName: Full=Heat shock protein HSS1 && PF00012:Hsp70 protein|PF10712:NAD-specific glutamate dehydrogenase
Lachesis_group0__13_contigs__l.g2246	2299.746	7946.352	-1.78881	0.000164	0.003649	1671	- && P9WGQ4.1 RecName: Full=Uncharacterized oxidoreductase MT0954 >P9WGQ5.1 RecName: Full=Uncharacterized oxidoreductase Rv0927c && PF13561:Enoyl-(Acyl carrier protein) reductase|PF00106:short chain dehydrogenase|PF01596:O-methyltransferase
Lachesis_group0__13_contigs__l.g5114	32.18667	111.2549	-1.78995	0.000706	0.010808	1635	- && Q9P6J5.1 RecName: Full=Uncharacterized permease C1683.05 && PF02133:Permease for cytosine/purines, uracil, thiamine, allantoin
Lachesis_group0__13_contigs__l.g3641	32.93783	114.1426	-1.7959	0.000638	0.010051	2061	- && - && PF00069:Protein kinase domain
Lachesis_group0__13_contigs__l.g8522	22.17835	77.26104	-1.80223	0.004282	0.039859	1161	- && - && PF13926:Domain of unknown function (DUF4211)
Lachesis_group0__13_contigs__l.g3103	189.7891	662.7602	-1.80366	9.03E-05	0.002408	3453	- && Q9Y6Z9.1 RecName: Full=Sorbose reductase sou1; AltName: Full=Sorbitol utilization protein sou1 && PF00106:short chain dehydrogenase
Lachesis_group0__13_contigs__l.g9495	2639.816	9216.351	-1.80382	0.000251	0.005176	738	- && - && PF00646:F-box domain
Lachesis_group0__13_contigs__l.g8618	30.21117	105.6307	-1.8086	0.000749	0.011265	1419	- && P49374.1 RecName: Full=High-affinity glucose transporter && PF00083:Sugar (and other) transporter

Lachesis_group0__13_contigs__l.g11158	106.3583	374.5071	-1.81634	0.000449	0.007899	984	- && Q5BGA7.1 RecName: Full=Probable NAD(P)H-dependent D-xylose reductase xyl1; Short=XR && PF00248:Aldo/keto reductase family
Lachesis_group0__13_contigs__l.g7189	45.14998	159.5885	-1.81927	0.000166	0.003693	2550	- && B0Y3M6.1 RecName: Full=Probable beta-glucosidase I; AltName: Full=Beta-D-glucoside glucohydrolase I; AltName: Full=Cellobiase I; AltName: Full=Gentiobiase I && PF14310:Fibronectin type III-like domain|PF00933:Glycosyl hydrolase family 3 N terminal domain|PF01915:Glycosyl hydrolase family 3 C-terminal domain
Lachesis_group0__13_contigs__l.g6865	233.4181	824.621	-1.82041	0.00045	0.007899	1032	- && P25148.2 RecName: Full=General stress protein A && PF01501:Glycosyl transferase family 8
Lachesis_group0__13_contigs__l.g845	2348.705	8328.432	-1.82618	0.000873	0.012745	4587	- && - && PF08229:ER membrane protein SH3
Lachesis_group0__13_contigs__l.g10640	784.423	2787.061	-1.82896	5.79E-05	0.001713	5436	- && O77033.1 RecName: Full=General transcriptional corepressor trfA && PF13414:TPR repeat
Lachesis_group0__13_contigs__l.g8631	397.6969	1412.915	-1.82903	0.002205	0.024034	1407	- && P0CO61.1 RecName: Full=Putative lipase ATG15; AltName: Full=Autophagy-related protein 15 && PF01764:Lipase (class 3)
Lachesis_group0__13_contigs__l.g2264	439.7426	1565.457	-1.83197	4.04E-05	0.001304	2058	- && E9R876.1 RecName: Full=MFS gliotoxin efflux transporter gliA; AltName: Full=Gliotoxin biosynthesis protein A && PF07690:Major Facilitator Superfamily
Lachesis_group0__13_contigs__l.g1026	125.8817	448.1859	-1.83224	3.07E-05	0.001055	1722	- && Q86UW6.2 RecName: Full=NEDD4-binding protein 2; Short=N4BP2; AltName: Full=BCL-3-

							binding protein && PF08590:Domain of unknown function (DUF1771)|PF01713:Smr domain
Lachesis_group0__13_contigs__l.g11147	1709.201	6100.697	-1.83561	0.000312	0.006083	1140	- && Q56WD9.2 RecName: Full=3-ketoacyl-CoA thiolase 2, peroxisomal; AltName: Full=Acetyl-CoA acyltransferase 2; AltName: Full=Beta-ketothiolase 2; AltName: Full=Peroxisomal 3-oxoacyl-CoA thiolase 2; AltName: Full=Peroxisome defective protein 1; Flags: Precursor && PF02803:Thiolase, C-terminal domain|PF00108:Thiolase, N-terminal domain
Lachesis_group0__13_contigs__l.g10680	3257.63	11644.2	-1.83776	0.000139	0.003262	1899	- && Q12581.1 RecName: Full=Cytochrome P450 52A5; AltName: Full=Alkane-inducible P450-ALK2-A; AltName: Full=CYPLIIA5 [Candida maltosa] && PF00067:Cytochrome P450
Lachesis_group0__13_contigs__l.g10682	7738.092	27669.58	-1.83826	1.16E-06	7.84E-05	1206	- && Q9UUA1.2 RecName: Full=Oxysterol-binding protein homolog C23B6.01c && PF01237:Oxysterol-binding protein
Lachesis_group0__13_contigs__l.g2200	108.7246	390.1199	-1.8435	0.001478	0.018081	1155	- && - && PF00150:Cellulase (glycosyl hydrolase family 5)
Lachesis_group0__13_contigs__l.g4651	1358.732	4877.538	-1.84392	6.42E-06	0.000308	3114	- && P40317.2 RecName: Full=Protein SOK1 && PF05794:T-complex protein 11
Lachesis_group0__13_contigs__l.g5402	75.38753	270.5487	-1.84462	0.000492	0.008383	2304	- && Q29496.1 RecName: Full=Cytochrome P450 3A24; AltName: Full=CYPIIIA24 && PF00067:Cytochrome P450
Lachesis_group0__13_contigs__l.g2061	42.20981	151.7577	-1.84469	0.003093	0.031092	759	- && P38310.2 RecName: Full=Iron transporter FTH1 && PF03239:Iron permease FTR1 family

Lachesis_group0__13_contigs__l.g2059	86.70378	312.9707	-1.85172	3.63E-06	0.000197	1734	- && P70786.1 RecName: Full=Putative tartrate transporter && PF07690:Major Facilitator Superfamily
Lachesis_group0__13_contigs__l.g11221	45.1746	163.6378	-1.85771	0.002661	0.027733	1029	- && Q8Y014.1 RecName: Full=Transaldolase && PF00923:Transaldolase
Lachesis_group0__13_contigs__l.g6881	1463.154	5306.377	-1.8586	0.000149	0.003426	1152	- && - && PF00134:Cyclin, N-terminal domain
Lachesis_group0__13_contigs__l.g8616	30.50488	110.9512	-1.86497	0.000972	0.013692	4218	- && A2Q8R2.2 RecName: Full=ATP-dependent DNA helicase mph1; AltName: Full=FANCM-like protein 1 && PF00271:Helicase conserved C-terminal domain|PF04851:Type III restriction enzyme, res subunit
Lachesis_group0__13_contigs__l.g5096	86.8821	317.2578	-1.86799	0.000114	0.002824	1293	- && - && PF00339:Arrestin (or S-antigen), N-terminal domain
Lachesis_group0__13_contigs__l.g5403	61005.61	223216.8	-1.87144	0.000394	0.007243	1074	- && P43067.1 RecName: Full=Alcohol dehydrogenase 1; AltName: Full=40 kDa allergen; AltName: Full=Allergen Can a 1; AltName: Full=Allergen Can a I; AltName: Allergen=Cand a 1 && PF08240:Alcohol dehydrogenase GroES-like domain|PF00107:Zinc-binding dehydrogenase
Lachesis_group0__13_contigs__l.g9431	551.4582	2019.635	-1.87271	0.000346	0.006522	744	- && O13962.1 RecName: Full=Putative cytochrome c1 heme lyase; Short=CC1HL && PF01265:Cytochrome c/c1 heme lyase
Lachesis_group0__13_contigs__l.g10515	75.98686	278.6633	-1.87451	0.004272	0.039801	1662	- && - && PF00501:AMP-binding enzyme
Lachesis_group0__13_contigs__l.g9562	1435.624	5275.039	-1.87758	0.000108	0.002701	423	- && D4ALV6.1 RecName: Full=Allergen Asp f 15 homolg && PF07249:Cerato-platanin
Lachesis_group0__13_contigs__l.g5514	131.8187	485.6057	-1.88097	0.000103	0.002617	840	- && - && PF09458:H-type lectin domain

Lachesis_group0__13_contigs__l.g10242	60.70936	225.1981	-1.89164	7.27E-05	0.002043	1716	- && P39992.1 RecName: Full=Uncharacterized protein YEL023C && PF09994:Uncharacterized alpha/beta hydrolase domain (DUF2235)
Lachesis_group0__13_contigs__l.g2892	437.9319	1630.492	-1.89635	0.000154	0.003493	1668	- && P0CU32.1 RecName: Full=FAD-linked oxidoreductase CHGG_01242-2; AltName: Full=Chaetoglobosin biosynthesis protein CHGG_01242-2 && PF01565:FAD binding domain
Lachesis_group0__13_contigs__l.g5617	71.68017	267.6102	-1.90143	0.000761	0.011385	939	- && P02723.1 RecName: Full=ADP,ATP carrier protein; AltName: Full=ADP/ATP translocase; AltName: Full=Adenine nucleotide translocator; Short=ANT && PF00153:Mitochondrial carrier protein
Lachesis_group0__13_contigs__l.g7531	27.55415	103.0335	-1.90332	0.004583	0.041747	963	- && E1U332.1 RecName: Full=Isoflavone reductase-like protein; AltName: Full=Pollen allergen Ole e 12.01; AltName: Allergen=Ole e 12.01 && PF05368:NmrA-like family
Lachesis_group0__13_contigs__l.g993	51.33822	193.0739	-1.91212	2.07E-05	0.000788	1593	- && B8NHY4.1 RecName: Full=O-methylsterigmatocystin oxidoreductase; Short=OMST oxidoreductase; AltName: Full=Aflatoxin B synthase; AltName: Full=Aflatoxin biosynthesis protein Q; AltName: Full=Cytochrome P450 64 >P0CT93.1 RecName: Full=O-methylsterigmatocystin oxidoreductase; Short=OMST oxidoreductase; AltName: Full=Aflatoxin B synthase; AltName: Full=Aflatoxin

							biosynthesis protein Q; AltName: Full=Cytochrome P450 64 && PF00067:Cytochrome P450
Lachesis_group0__13_contigs__l.g9768	90.12265	339.402	-1.91383	3.46E-05	0.001146	2799	- && Q2U4L7.2 RecName: Full=Glutaminase A; Flags: Precursor && PF16335:Domain of unknown function (DUF4965)|PF08760:Domain of unknown function (DUF1793)
Lachesis_group0__13_contigs__l.g1530	7583.489	28613.56	-1.91578	5.26E-07	4.13E-05	4059	- && P40406.1 RecName: Full=Beta-hexosaminidase; AltName: Full=Beta-N-acetylhexosaminidase; AltName: Full=N-acetyl-beta-glucosaminidase; AltName: Full=N-acetylglucosaminidase; AltName: Full=ORF1; Flags: Precursor && PF03127:GAT domain|PF00790:VHS domain|PF00933:Glycosyl hydrolase family 3 N terminal domain
Lachesis_group0__13_contigs__l.g597	3217.564	12145.82	-1.91644	4.81E-05	0.001473	966	- && O14283.2 RecName: Full=Transcription factor prr1; AltName: Full=Pombe response regulator 1 && PF00447:HSF-type DNA-binding
Lachesis_group0__13_contigs__l.g5178	170.4259	643.4233	-1.91729	3.39E-06	0.000189	1485	- && O14039.3 RecName: Full=25S rRNA (cytosine-C(5))-methyltransferase rcm1; AltName: Full=rRNA m(5)C methyltransferase 1 && PF01189:16S rRNA methyltransferase RsmF
Lachesis_group0__13_contigs__l.g4367	26.41578	100.7264	-1.93104	0.000682	0.010532	3351	- && B8NHY4.1 RecName: Full=O-methylsterigmatocystin oxidoreductase; Short=OMST oxidoreductase; AltName: Full=Aflatoxin B synthase; AltName: Full=Aflatoxin biosynthesis protein Q; AltName: Full=Cytochrome P450 64 >P0CT93.1 RecName: Full=O-

							methylsterigmatocystin oxidoreductase; Short=OMST oxidoreductase; AltName: Full=Aflatoxin B synthase; AltName: Full=Aflatoxin biosynthesis protein Q; AltName: Full=Cytochrome P450 64 && PF00067:Cytochrome P450
Lachesis_group0__13_contigs__l.g5916	14.47309	55.21273	-1.93309	0.003433	0.033728	2223	- && - && PF00271:Helicase conserved C-terminal domain|PF00270:DEAD/DEAH box helicase
Lachesis_group0__13_contigs__l.g8282	80.11779	307.1913	-1.9404	5.74E-05	0.001706	1662	- && F4JY37.1 RecName: Full=Serine/threonine-protein kinase RUNKEL; AltName: Full=Protein EMBRYO DEFECTIVE 3013; AltName: Full=Protein RUNKEL && PF07714:Protein tyrosine kinase
Lachesis_group0__13_contigs__l.g9594	39.20872	151.0003	-1.94518	0.000239	0.00499	1008	- && Q5XGR8.1 RecName: Full=Endoribonuclease LACTB2; AltName: Full=Beta-lactamase-like protein 2 && PF00753:Metallo-beta-lactamase superfamily
Lachesis_group0__13_contigs__l.g3589	35.34568	136.4506	-1.94714	0.000279	0.005536	3213	- && A0A0D2YG01.1 RecName: Full=Non-canonical non-ribosomal peptide synthetase FUB8; AltName: Full=Fusaric acid biosynthesis protein 8 && PF07993:Male sterility protein|PF00501:AMP-binding enzyme
Lachesis_group0__13_contigs__l.g7558	29.63967	114.1463	-1.94805	0.001974	0.02223	1578	- && Q10087.1 RecName: Full=Uncharacterized amino-acid permease C11D3.08c && PF13520:Amino acid permease
Lachesis_group0__13_contigs__l.g5374	16.83672	65.05724	-1.95204	0.005846	0.049883	1557	- && G3Y416.1 RecName: Full=Cytochrome P450 monooxygenase yanC; AltName: Full=Yanuthone D synthesis protein C; Flags: Precursor && PF00067:Cytochrome P450

Lachesis_group0__13_contigs__l.g305	7708.734	30008.38	-1.96082	0.000283	0.0056	1650	- && Q3MIF4.1 RecName: Full=Xylulose kinase; Short=Xylulokinase && PF00370:FGGY family of carbohydrate kinases, N-terminal domain|PF02782:FGGY family of carbohydrate kinases, C-terminal domain
Lachesis_group0__13_contigs__l.g10787	1525.701	5941.727	-1.96146	1.47E-06	9.39E-05	2169	- && Q9Z8P2.1 RecName: Full=3-oxoacyl-[acyl-carrier-protein] reductase FabG; AltName: Full=3-ketoacyl-acyl carrier protein reductase; AltName: Full=Beta-Ketoacyl-acyl carrier protein reductase; AltName: Full=Beta-ketoacyl-ACP reductase && PF00106:short chain dehydrogenase
Lachesis_group0__13_contigs__l.g3352	132.3401	516.3458	-1.96377	8.08E-06	0.000369	1416	- && A1CTI3.1 RecName: Full=Probable glucan 1,3-beta-glucosidase D; AltName: Full=Exo-1,3-beta-glucanase D && PF00150:Cellulase (glycosyl hydrolase family 5)
Lachesis_group0__13_contigs__l.g282	14.30406	55.9394	-1.96934	0.003806	0.036529	1587	- && O60020.1 RecName: Full=Aspartic protease; Flags: Precursor && PF00026:Eukaryotic aspartyl protease
Lachesis_group0__13_contigs__l.g2610	360.3108	1412.834	-1.97143	0.000269	0.005413	1341	- && P51660.3 RecName: Full=Peroxisomal multifunctional enzyme type 2; Short=MFE-2; AltName: Full=17-beta-hydroxysteroid dehydrogenase 4; Short=17-beta-HSD 4; AltName: Full=D-bifunctional protein; Short=DBP; AltName: Full=Multifunctional protein 2; Short=MPF-2; Contains: RecName: Full=(3R)-hydroxyacyl-CoA dehydrogenase; Contains: RecName: Full=Enoyl-

							CoA hydratase 2; AltName: Full=3-alpha,7-alpha,12-alpha-trihydroxy-5-beta-cholest-24-enoyl-CoA hydratase && PF01575:MaoC like domain
Lachesis_group0__13_contigs__l.g8157	1698.977	6683.555	-1.97603	9.24E-06	0.000406	3306	- && - && PF03370:Carbohydrate/starch-binding module (family 21)
Lachesis_group0__13_contigs__l.g1697	1998.391	7963.659	-1.99461	3.32E-07	2.94E-05	2661	- && Q7ZUW2.1 RecName: Full=Hypoxia up-regulated protein 1; Flags: Precursor && PF00012:Hsp70 protein
Lachesis_group0__13_contigs__l.g11192	71.14871	283.9119	-1.99649	4.15E-05	0.001333	2703	- && - && PF08124:Polysaccharide lyase family 8, N terminal alpha-helical domain|PF02278:Polysaccharide lyase family 8, super-sandwich domain|PF02884:Polysaccharide lyase family 8, C-terminal beta-sandwich domain
Lachesis_group0__13_contigs__l.g1724	2859.92	11441.73	-2.00024	0.003353	0.033157	1755	- && - && PF02179:BAG domain
Lachesis_group0__13_contigs__l.g5534	545.2601	2183.021	-2.00135	6.05E-06	0.000296	1626	- && P07921.1 RecName: Full=Lactose permease && PF00083:Sugar (and other) transporter
Lachesis_group0__13_contigs__l.g10144	85.41559	342.1686	-2.00271	0.000267	0.005395	1458	- && - && PF07690:Major Facilitator Superfamily
Lachesis_group0__13_contigs__l.g10128	18.16137	72.76072	-2.0038	0.001001	0.013994	2388	- && Q551H4.1 RecName: Full=Serine/threonine-protein kinase fray2; AltName: Full=STE20-like kinase fray2 && PF00069:Protein kinase domain
Lachesis_group0__13_contigs__l.g2771	111.008	451.2754	-2.0238	0.000833	0.012277	1935	- && Q8NK92.2 RecName: Full=Aorsin; Flags: Precursor && PF09286:Pro-kumamolisin, activation domain
Lachesis_group0__13_contigs__l.g6060	61.67407	251.8017	-2.02985	7.03E-05	0.001998	1308	- && O34714.1 RecName: Full=Oxalate decarboxylase OxdC && PF00190:Cupin

Lachesis_group0__13_contigs__l.g10552	34.08804	138.9952	-2.03014	0.000429	0.00766	1032	- && Q9UVP6.1 RecName: Full=Versatile peroxidase VPS1; AltName: Full=Versatile solid phase peroxidase 1; Flags: Precursor && PF00141:Peroxidase|PF11895:Domain of unknown function (DUF3415)
Lachesis_group0__13_contigs__l.g623	689.3586	2822.644	-2.03395	3.31E-05	0.001116	1839	- && P84285.1 RecName: Full=Sterol O-acyltransferase 2; AltName: Full=ASAT; AltName: Full=Sterol-ester synthase && PF03062:MBOAT, membrane-bound O-acyltransferase family
Lachesis_group0__13_contigs__l.g4553	280.9105	1153.885	-2.03817	3.40E-07	2.99E-05	786	- && D2WKD9.2 RecName: Full=Farnesol dehydrogenase; AltName: Full=NADP+-dependent farnesol dehydrogenase 1; Short=AaSDR-1 && PF00106:short chain dehydrogenase
Lachesis_group0__13_contigs__l.g8548	99.89986	412.2567	-2.0454	4.69E-06	0.000241	2457	- && D4ATR3.1 RecName: Full=Uncharacterized secreted glycosidase ARB_07629; Flags: Precursor && PF07971:Glycosyl hydrolase family 92
Lachesis_group0__13_contigs__l.g11054	34.58445	143.7037	-2.05564	0.000308	0.006037	1509	- && Q4WAZ6.1 RecName: Full=Multifunctional cytochrome P450 monooxygenase af510; AltName: Full=Fumagillin bioynthesis cluster P450 monooxygenase; Short=Fma-P450 && PF00067:Cytochrome P450
Lachesis_group0__13_contigs__l.g990	117.5925	489.2842	-2.05641	2.28E-05	0.000856	2844	- && Q4WAZ6.1 RecName: Full=Multifunctional cytochrome P450 monooxygenase af510; AltName: Full=Fumagillin bioynthesis cluster P450 monooxygenase; Short=Fma-P450 && PF00067:Cytochrome P450

Lachesis_group0__13_contigs__l.g9874	25.38319	105.9169	-2.06195	0.000258	0.005273	1044	- && - && PF00230:Major intrinsic protein
Lachesis_group0__13_contigs__l.g3393	714.4384	2994.019	-2.06717	6.34E-05	0.001842	2451	- && Q0CEF3.1 RecName: Full=Probable beta-glucosidase L; AltName: Full=Beta-D-glucoside glucohydrolase L; AltName: Full=Cellobiase L; AltName: Full=Gentiobiase L; Flags: Precursor && PF01915:Glycosyl hydrolase family 3 C-terminal domain|PF14310:Fibronectin type III-like domain|PF00933:Glycosyl hydrolase family 3 N terminal domain
Lachesis_group0__13_contigs__l.g1947	16.37331	68.97738	-2.07637	0.005099	0.045172	1215	- && A6TBU6.1 RecName: Full=2-keto-3-deoxy-L-rhamnonate aldolase; Short=KDR aldolase; AltName: Full=2-dehydro-3-deoxyrhamnonate aldolase && PF03328:HpcH/HpaI aldolase/citrate lyase family
Lachesis_group0__13_contigs__l.g2185	53.92062	227.4638	-2.07862	0.000722	0.010963	1935	- && - && PF00856:SET domain
Lachesis_group0__13_contigs__l.g1248	78.92864	333.8246	-2.08182	6.97E-05	0.001991	2037	- && - && PF05592:Bacterial alpha-L-rhamnosidase
Lachesis_group0__13_contigs__l.g4262	25.90412	110.5352	-2.09377	0.000931	0.013367	975	- && B8NM62.1 RecName: Full=Pyridoxamine 5'-phosphate oxidase family protein ustO; AltName: Full=Ustiloxin B biosynthesis protein O && -
Lachesis_group0__13_contigs__l.g4344	17.79882	75.79237	-2.0946	0.000732	0.01108	1152	- && O74957.1 RecName: Full=Protein argonaute; AltName: Full=Cell cycle control protein ago1; AltName: Full=Eukaryotic translation initiation factor 2C 2-like protein ago1; AltName: Full=PAZ Piwi domain protein ago1; AltName: Full=Protein slicer; AltName: Full=RNA interference pathway protein ago1 && PF02171:Piwi domain

Lachesis_group0__13_contigs__l.g9792	54.95141	234.5071	-2.09547	0.001008	0.014013	1719	- && Q756A9.2 RecName: Full=Acyl-coenzyme A oxidase; Short=Acyl-CoA oxidase && -
Lachesis_group0__13_contigs__l.g7330	49.13884	210.0616	-2.09654	2.40E-06	0.000144	1320	- && A1DDK1.1 RecName: Full=Aspartic protease pep1; AltName: Full=Aspergillopepsin A; AltName: Full=Aspergillopepsin I; Flags: Precursor && PF00026:Eukaryotic aspartyl protease
Lachesis_group0__13_contigs__l.g8727	142.8247	611.8908	-2.09872	1.25E-06	8.34E-05	2337	- && Q9URY8.1 RecName: Full=Probable sulfate permease C869.05c && PF01740:STAS domain|PF00916:Sulfate permease family
Lachesis_group0__13_contigs__l.g10950	6370.955	27334.53	-2.10116	0.000187	0.004048	981	- && Q876L8.1 RecName: Full=NAD(P)H-dependent D-xylose reductase xyl1; Short=XR && PF00248:Aldo/keto reductase family
Lachesis_group0__13_contigs__l.g8672	15.07447	64.8084	-2.10366	0.001547	0.018585	1674	- && P42251.3 RecName: Full=Alkaline phosphatase D; Short=APaseD; Flags: Precursor && PF09423:PhoD-like phosphatase|PF16655:PhoD-like phosphatase, N-terminal domain
Lachesis_group0__13_contigs__l.g2685	174.7439	753.1194	-2.10778	1.10E-06	7.52E-05	897	- && Q1ZXF1.1 RecName: Full=Probable enoyl-CoA hydratase, mitochondrial; AltName: Full=Enoyl-CoA hydratase 1; AltName: Full=Short-chain enoyl-CoA hydratase; Short=SCEH; Flags: Precursor && PF00378:Enoyl-CoA hydratase/isomerase
Lachesis_group0__13_contigs__l.g7972	51.58448	222.7892	-2.10972	8.41E-06	0.000381	963	- && P18631.1 RecName: Full=Low-affinity glucose transporter; AltName: Full=Hexose transporter 1 && PF00083:Sugar (and other) transporter
Lachesis_group0__13_contigs__l.g2607	20.48101	88.78328	-2.11386	0.002048	0.022852	756	- && O64519.1 RecName: Full=Metacaspase-6; Short=AtMC6; AltName: Full=Metacaspase 2c;

							Short=AtMCP2c; AltName: Full=Metacaspase-5 && PF00656:Caspase domain
Lachesis_group0__13_contigs__l.g2517	28.26478	122.8177	-2.12055	0.000297	0.00583	1395	- && O34371.1 RecName: Full=Putative oxidoreductase YteT; Flags: Precursor && PF01408:Oxidoreductase family, NAD-binding Rossmann fold|PF02894:Oxidoreductase family, C-terminal alpha/beta domain
Lachesis_group0__13_contigs__l.g10317	126.8623	551.5857	-2.12106	6.91E-07	5.05E-05	2376	- && O94556.2 RecName: Full=Anaphase-promoting complex subunit 8; AltName: Full=20S cyclosome/APC complex protein apc8; AltName: Full=Cell untimely torn protein 23 && PF04049:Anaphase promoting complex subunit 8 / Cdc23|PF13414:TPR repeat
Lachesis_group0__13_contigs__l.g273	2348.081	10221.66	-2.12212	5.37E-07	4.19E-05	1488	- && - && PF05057:Putative serine esterase (DUF676)
Lachesis_group0__13_contigs__l.g714	169.2153	740.6152	-2.12964	6.29E-07	4.63E-05	1824	- && Q00730.2 RecName: Full=Putative sterigmatocystin biosynthesis monooxygenase stcW && PF13738:Pyridine nucleotide-disulphide oxidoreductase
Lachesis_group0__13_contigs__l.g7662	63.6045	278.0359	-2.12999	3.00E-05	0.001035	2667	- && P08159.2 RecName: Full=6-hydroxy-D-nicotine oxidase; Short=6-HDNO && PF08031:Berberine and berberine like|PF01565:FAD binding domain
Lachesis_group0__13_contigs__l.g1463	38.93311	171.0676	-2.1369	0.000492	0.008383	2145	- && O94564.1 RecName: Full=Zinc-type alcohol dehydrogenase-like protein C1773.06c && PF00107:Zinc-binding

							dehydrogenase|PF08240:Alcohol dehydrogenase GroES-like domain
Lachesis_group0__13_contigs__l.g10102	9.481662	42.31954	-2.15787	0.00154	0.018585	1599	- && Q9M156.1 RecName: Full=UDP-glycosyltransferase 72B1; AltName: Full=Arbutin synthase; AltName: Full=Probable hydroquinone glucosyltransferase && PF00201:UDP-glucoronosyl and UDP-glucosyl transferase
Lachesis_group0__13_contigs__l.g10002	28.59723	127.4397	-2.15972	0.00515	0.045545	1059	- && A8DRH7.1 RecName: Full=L-threo-3-deoxy-hexylosonate aldolase; AltName: Full=L-threo-3-deoxy-hexulosonate aldolase && PF00701:Dihydrodipicolinate synthetase family
Lachesis_group0__13_contigs__l.g1173	310.9804	1389.558	-2.16017	0.003351	0.033157	1239	- && O43050.1 RecName: Full=Sterol-4-alpha-carboxylate 3-dehydrogenase, decarboxylating && PF01073:3-beta hydroxysteroid dehydrogenase/isomerase family
Lachesis_group0__13_contigs__l.g4931	92.20732	415.5339	-2.17262	7.22E-05	0.002035	834	- && - && PF12695:Alpha/beta hydrolase family
Lachesis_group0__13_contigs__l.g8917	315.6984	1426.5	-2.17615	8.33E-05	0.002278	1029	- && O95154.2 RecName: Full=Aflatoxin B1 aldehyde reductase member 3; AltName: Full=AFB1 aldehyde reductase 2; Short=AFB1-AR 2 && PF00248:Aldo/keto reductase family
Lachesis_group0__13_contigs__l.g10661	14.9419	67.60725	-2.17918	0.000336	0.00639	6573	- && Q8BV79.3 RecName: Full=TPR and ankyrin repeat-containing protein 1; AltName: Full=Lupus brain antigen 1 && PF13361:UvrD-like helicase C-terminal domain|PF00580:UvrD/REP helicase N-terminal domain

Lachesis_group0__13_contigs__l.g10170	53.68053	243.2156	-2.18039	0.001446	0.017806	1029	- && P79213.1 RecName: Full=Aquaporin-2; Short=AQP-2; AltName: Full=ADH water channel; AltName: Full=Aquaporin-CD; Short=AQP-CD; AltName: Full=Collecting duct water channel protein; AltName: Full=WCH-CD; AltName: Full=Water channel protein for renal collecting duct && PF00230:Major intrinsic protein
Lachesis_group0__13_contigs__l.g1239	71.81856	326.2024	-2.18321	2.19E-07	2.12E-05	4284	- && Q5UQ40.1 RecName: Full=Probable bifunctional E2/E3 enzyme R795; Includes: RecName: Full=E3 ubiquitin-protein ligase; AltName: Full=RING-type E3 ubiquitin transferase; Includes: RecName: Full=Ubiquitin-conjugating enzyme E2; AltName: Full=E2 ubiquitin-conjugating enzyme && PF00179:Ubiquitin-conjugating enzyme
Lachesis_group0__13_contigs__l.g10	5233.016	23867.48	-2.18933	7.54E-05	0.002112	1548	- && P07871.2 RecName: Full=3-ketoacyl-CoA thiolase B, peroxisomal; AltName: Full=Acetyl-CoA acyltransferase B; AltName: Full=Beta-ketothiolase B; AltName: Full=Peroxisomal 3-oxoacyl-CoA thiolase B; Flags: Precursor && PF00108:Thiolase, N-terminal domain|PF02803:Thiolase, C-terminal domain
Lachesis_group0__13_contigs__l.g8910	65.20768	299.0495	-2.197	0.000522	0.008756	1701	- && Q7Z9I0.2 RecName: Full=Uncharacterized MFS-type transporter SPBC409.08 && PF07690:Major Facilitator Superfamily

Lachesis_group0__13_contigs__l.g1014	117.5769	539.3948	-2.19801	3.99E-06	0.000213	1542	- && Q9US44.1 RecName: Full=Uncharacterized transporter C1002.16c && PF07690:Major Facilitator Superfamily
Lachesis_group0__13_contigs__l.g8571	22.92429	105.5073	-2.20344	2.59E-05	0.000939	1551	- && P0CO49.1 RecName: Full=Kynurenine 3-monooxygenase; AltName: Full=Biosynthesis of nicotinic acid protein 4; AltName: Full=Kynurenine 3-hydroxylase && PF01494:FAD binding domain
Lachesis_group0__13_contigs__l.g5093	27.32617	125.8339	-2.2036	0.000274	0.005481	1140	- && I1S5P3.1 RecName: Full=Global transcription regulator FGP1 && PF09729:Gti1/Pac2 family
Lachesis_group0__13_contigs__l.g3353	79.00065	364.8907	-2.20768	0.000145	0.003375	1422	- && Q10444.1 RecName: Full=Glucan 1,3-beta-glucosidase 2; AltName: Full=Exo-1,3-beta-glucanase 2; Flags: Precursor && PF00150:Cellulase (glycosyl hydrolase family 5)
Lachesis_group0__13_contigs__l.g3871	40.58774	187.4775	-2.20879	1.58E-05	0.000635	2310	- && C5PFX0.2 RecName: Full=Nuclear distribution protein PAC1; AltName: Full=Lissencephaly-1 homolog; Short=LIS-1; AltName: Full=nudF homolog && PF00400:WD domain, G-beta repeat
Lachesis_group0__13_contigs__l.g1287	75.78386	351.0323	-2.21061	0.000101	0.002597	615	- && - && PF01822:WSC domain
Lachesis_group0__13_contigs__l.g6438	59.16337	274.2103	-2.21424	0.001111	0.014755	1077	- && Q76NT9.1 RecName: Full=1-aminocyclopropane-1-carboxylate oxidase; Short=ACC oxidase; Short=Ddaco; AltName: Full=Ethylene-forming enzyme; Short=EFE >A6BM06.1 RecName: Full=1-aminocyclopropane-1-carboxylate oxidase; Short=ACC oxidase; Short=Dmaco; AltName: Full=Ethylene-forming enzyme; Short=EFE &&

							PF03171:2OG-Fe(II) oxygenase superfamily|PF14226:non-haem dioxygenase in morphine synthesis N-terminal
Lachesis_group0__13_contigs__l.g8031	549.3762	2552.96	-2.21647	3.09E-07	2.76E-05	2988	- && Q06681.1 RecName: Full=Membrane-anchored lipid-binding protein YSP2; AltName: Full=Lipid transfer at contact site protein 4; AltName: Full=Lipid transfer protein anchored at membrane contact sites 3; AltName: Full=Yeast suicide protein 2 && PF16016:Domain of unknown function (DUF4782)|PF02893:GRAM domain
Lachesis_group0__13_contigs__l.g4834	655.9588	3068.556	-2.22605	2.79E-07	2.51E-05	1062	- && Q9P7F4.1 RecName: Full=Zinc-type alcohol dehydrogenase-like protein C2E1P3.01 && PF08240:Alcohol dehydrogenase GroES-like domain|PF00107:Zinc-binding dehydrogenase
Lachesis_group0__13_contigs__l.g7661	23.09186	108.9751	-2.23781	4.87E-05	0.001489	1476	- && P25737.5 RecName: Full=Lysine-specific permease && PF00324:Amino acid permease
Lachesis_group0__13_contigs__l.g5917	1874.263	8883.123	-2.24475	1.51E-10	3.94E-08	1050	- && - && PF03036:Perilipin family
Lachesis_group0__13_contigs__l.g6708	104.4564	496.2827	-2.2483	1.09E-08	1.72E-06	1128	- && A1CFY8.2 RecName: Full=Probable D-xylulose reductase A; AltName: Full=Xylitol dehydrogenase A && PF08240:Alcohol dehydrogenase GroES-like domain|PF00107:Zinc-binding dehydrogenase
Lachesis_group0__13_contigs__l.g3061	36.5207	174.7097	-2.25741	5.37E-06	0.00027	1419	- && - && PF00172:Fungal Zn(2)-Cys(6) binuclear cluster domain
Lachesis_group0__13_contigs__l.g281	95.93211	459.7407	-2.26117	0.001083	0.014534	2409	- && - && PF17107:N-terminal domain on NACHT_NTPase and P-loop NTPases

Lachesis_group0__13_contigs__l.g5624	695.8693	3350.607	-2.26768	1.07E-05	0.000457	1332	- && Q9P6P9.1 RecName: Full=[Pyruvate dehydrogenase (acetyl-transferring)] kinase, mitochondrial; Short=Pyruvate dehydrogenase kinase; Flags: Precursor && PF10436:Mitochondrial branched-chain alpha-ketoacid dehydrogenase kinase|PF02518:Histidine kinase-, DNA gyrase B-, and HSP90-like ATPase
Lachesis_group0__13_contigs__l.g8190	131.2531	631.7578	-2.26775	1.69E-05	0.000668	1329	- && - && PF00652:Ricin-type beta-trefoil lectin domain
Lachesis_group0__13_contigs__l.g3254	131.0556	641.5814	-2.29103	1.13E-05	0.000478	3033	- && - && PF00004:ATPase family associated with various cellular activities (AAA)
Lachesis_group0__13_contigs__l.g7108	15.17121	74.27791	-2.29355	9.81E-05	0.002563	1278	- && P41816.2 RecName: Full=NADPH dehydrogenase 3; AltName: Full=Old yellow enzyme 3 && PF00724:NADH:flavin oxidoreductase / NADH oxidase family
Lachesis_group0__13_contigs__l.g7087	298.8659	1467.308	-2.29574	0.000262	0.005311	2643	- && B7GFR1.1 RecName: Full=Aspartate--tRNA(Asp/Asn) ligase; AltName: Full=Aspartyl-tRNA synthetase; Short=AspRS; AltName: Full=Non-discriminating aspartyl-tRNA synthetase; Short=ND-AspRS && PF00152:tRNA synthetases class II (D, K and N)
Lachesis_group0__13_contigs__l.g1952	36.19839	177.9918	-2.29623	0.000433	0.007725	444	- && - && PF16850:Peptidase inhibitor I66
Lachesis_group0__13_contigs__l.g8786	192.5439	948.8759	-2.30127	9.32E-08	1.04E-05	1053	- && O42909.1 RecName: Full=Zinc-type alcohol dehydrogenase-like protein C16A3.02c && PF13602:Zinc-binding dehydrogenase

Lachesis_group0__13_contigs__l.g1953	20.48362	101.4558	-2.31074	0.000394	0.007243	1104	- && - && PF00149:Calcineurin-like phosphoesterase
Lachesis_group0__13_contigs__l.g317	78.2483	389.2061	-2.314	0.000101	0.002597	1626	- && - && PF00172:Fungal Zn(2)-Cys(6) binuclear cluster domain
Lachesis_group0__13_contigs__l.g11168	38.23249	192.0309	-2.33049	4.65E-05	0.001443	1026	- && Q8ZTE1.1 RecName: Full=Ketol-acid reductoisomerase (NADP(+)); Short=KARI; AltName: Full=Acetohydroxy-acid isomeroreductase; Short=AHIR; AltName: Full=Alpha-keto-beta-hydroxylacyl reductoisomerase; AltName: Full=Ketol-acid reductoisomerase type 1; AltName: Full=Ketol-acid reductoisomerase type I && PF07991:Acetohydroxy acid isomeroreductase, NADPH-binding domain|PF01450:Acetohydroxy acid isomeroreductase, catalytic domain
Lachesis_group0__13_contigs__l.g10551	38.12325	191.7842	-2.33303	0.001065	0.01444	1887	- && Q9FVQ0.1 RecName: Full=Probable indole-3-pyruvate monooxygenase YUCCA10; AltName: Full=Flavin-containing monooxygenase YUCCA10 && PF13738:Pyridine nucleotide-disulphide oxidoreductase
Lachesis_group0__13_contigs__l.g4414	8.774312	44.25679	-2.34061	0.00398	0.037921	1326	- && O94343.1 RecName: Full=Uncharacterized MFS-type transporter C1271.10c && PF07690:Major Facilitator Superfamily
Lachesis_group0__13_contigs__l.g6432	79.12319	401.3241	-2.34253	4.74E-09	8.12E-07	621	- && - && PF00076:RNA recognition motif. (a.k.a. RRM, RBD, or RNP domain)
Lachesis_group0__13_contigs__l.g9821	859.3987	4380.599	-2.34986	6.17E-07	4.60E-05	4662	- && - && PF00069:Protein kinase domain

Lachesis_group0__13_contigs__l.g4810	30.24156	155.7144	-2.36512	0.000488	0.00836	1398	- && - && PF01231:Indoleamine 2,3-dioxygenase
Lachesis_group0__13_contigs__l.g5020	465.0949	2408.618	-2.37269	7.77E-07	5.64E-05	1728	- && Q47944.1 RecName: Full=L-sorbose 1-dehydrogenase; Short=SDH && PF00732:GMC oxidoreductase|PF05199:GMC oxidoreductase
Lachesis_group0__13_contigs__l.g3662	8.671326	44.85142	-2.37512	0.000415	0.007499	798	- && - && PF11807:Domain of unknown function (DUF3328)
Lachesis_group0__13_contigs__l.g1698	29.37246	153.7931	-2.38649	1.20E-05	0.000505	1695	- && - && PF00651:BTB/POZ domain
Lachesis_group0__13_contigs__l.g4961	11.75609	61.38388	-2.39107	0.001561	0.018673	1107	- && Q9HEZ1.1 RecName: Full=Endo-1,4-beta-xylanase A; Short=Xylanase A; AltName: Full=1,4-beta-D-xylan xylanohydrolase A; Flags: Precursor && PF00331:Glycosyl hydrolase family 10
Lachesis_group0__13_contigs__l.g5294	20.366	106.8336	-2.39219	0.001274	0.016163	975	- && - && PF05577:Serine carboxypeptidase S28
Lachesis_group0__13_contigs__l.g6589	27.75033	146.6522	-2.40139	5.71E-05	0.0017	1104	- && O13629.1 RecName: Full=Putative lipoate-protein ligase A && -
Lachesis_group0__13_contigs__l.g2810	17.43105	92.02063	-2.40186	0.001474	0.018069	882	- && P24665.1 RecName: Full=Aspergillopepsin-2; AltName: Full=Acid protease A; AltName: Full=Aspergillopepsin II; AltName: Full=Proctase A; Contains: RecName: Full=Aspergillopepsin-2 light chain; AltName: Full=Aspergillopepsin II light chain; Contains: RecName: Full=Aspergillopepsin-2 heavy chain; AltName: Full=Aspergillopepsin II heavy chain; Flags: Precursor && PF01828:Peptidase A4 family
Lachesis_group0__13_contigs__l.g4940	1601.806	8472.739	-2.40311	0.000198	0.004251	1140	- && Q06497.1 RecName: Full=Peroxisomal adenine nucleotide transporter 1 && PF00153:Mitochondrial carrier protein

Lachesis_group0__13_contigs__l.g4939	10.07746	53.4017	-2.40571	0.000279	0.005536	1026	- && - && PF10294:Lysine methyltransferase
Lachesis_group0__13_contigs__l.g5078	8854.321	46941.4	-2.40642	0.000121	0.002932	2307	- && O74770.1 RecName: Full=Probable phosphoketolase && PF09363:XFP C-terminal domain|PF09364:XFP N-terminal domain|PF03894:D-xylulose 5-phosphate/D-fructose 6-phosphate phosphoketolase
Lachesis_group0__13_contigs__l.g7337	8.038406	42.75057	-2.41253	0.000714	0.010899	1815	- && - && PF00076:RNA recognition motif. (a.k.a. RRM, RBD, or RNP domain)
Lachesis_group0__13_contigs__l.g10375	2445.144	13129.39	-2.42482	3.17E-08	4.42E-06	510	- && - && PF02755:RPEL repeat
novel.1164	248.4465	1334.225	-2.42516	1.93E-07	1.92E-05	2041	- && - && PF10164:Uncharacterized conserved protein (DUF2367)
Lachesis_group0__13_contigs__l.g5712	31.93464	171.2231	-2.426	2.99E-05	0.001033	2346	- && - && PF00069:Protein kinase domain
Lachesis_group0__13_contigs__l.g8160	38.73036	208.1735	-2.42754	0.000145	0.003375	1380	- && P23262.4 RecName: Full=Salicylate hydroxylase; AltName: Full=Salicylate 1-monooxygenase && PF13450:NAD(P)-binding Rossmann-like domain|PF01494:FAD binding domain
Lachesis_group0__13_contigs__l.g7082	436.2204	2352.977	-2.4314	1.66E-05	0.000662	1368	- && Q8RWZ3.1 RecName: Full=Probable acyl-CoA dehydrogenase IBR3; AltName: Full=Protein INDOLE-3-BUTYRIC ACID RESPONSE 3 && PF00441:Acyl-CoA dehydrogenase, C-terminal domain|PF02770:Acyl-CoA dehydrogenase, middle domain|PF02771:Acyl-CoA dehydrogenase, N-terminal domain
Lachesis_group0__13_contigs__l.g6641	712.4141	3843.506	-2.43159	3.38E-11	1.11E-08	2220	- && Q95M17.1 RecName: Full=Acidic mammalian chitinase; Short=AMCase; AltName: Full=Chitin-

							binding protein b04; Short=CBPb04; Flags: Precursor && PF00704:Glycosyl hydrolases family 18
Lachesis_group0__13_contigs__l.g1146	151.9484	820.1145	-2.43271	2.88E-08	4.07E-06	2244	- && P32816.1 RecName: Full=Glycerol dehydrogenase; Short=GDH; Short=GLDH; Short=GlyDH && PF00465:Iron-containing alcohol dehydrogenase
Lachesis_group0__13_contigs__l.g6191	56.23651	305.2065	-2.43992	9.39E-08	1.04E-05	1002	- && Q9UTA1.2 RecName: Full=Zinc finger protein C25B8.19c && -
Lachesis_group0__13_contigs__l.g11178	757.3722	4146.339	-2.45283	3.85E-08	5.11E-06	936	- && O13780.1 RecName: Full=Uncharacterized protein C17G6.02c && PF04479:RTA1 like protein
Lachesis_group0__13_contigs__l.g10899	452.7788	2480.042	-2.45338	0.000209	0.004444	1500	- && Q54HD2.1 RecName: Full=Probable serine/threonine-protein kinase ndrD; AltName: Full=Nuclear DBF2-related kinase D && PF00069:Protein kinase domain
Lachesis_group0__13_contigs__l.g5100	426.243	2365.045	-2.47231	1.15E-07	1.27E-05	537	- && B0DN41.1 RecName: Full=Cyanate hydratase; Short=Cyanase; AltName: Full=Cyanate hydrolase; AltName: Full=Cyanate lyase && PF02560:Cyanate lyase C-terminal domain
Lachesis_group0__13_contigs__l.g4541	19.12166	106.0708	-2.47518	0.000325	0.006264	1155	- && Q8RXK7.2 RecName: Full=Probable pectinesterase/pectinesterase inhibitor 41; Includes: RecName: Full=Pectinesterase inhibitor 41; AltName: Full=Pectin methylesterase inhibitor 41; Includes: RecName: Full=Pectinesterase 41; Short=PE 41; AltName: Full=AtPMEpcrB; AltName: Full=Pectin methylesterase 41; Short=AtPME41; Flags: Precursor && PF01095:Pectinesterase

Lachesis_group0__13_contigs__l.g3548	64.28439	360.9824	-2.49081	9.62E-05	0.002533	3453	- && A0A0D2YG01.1 RecName: Full=Non-canonical non-ribosomal peptide synthetase FUB8; AltName: Full=Fusaric acid biosynthesis protein 8 && PF00501:AMP-binding enzyme|PF07993:Male sterility protein
Lachesis_group0__13_contigs__l.g5028	38.30985	215.1849	-2.49111	0.001775	0.020485	540	- && - && PF05368:NmrA-like family
Lachesis_group0__13_contigs__l.g4400	8.904758	50.05278	-2.49279	0.000736	0.01111	1845	- && Q47944.1 RecName: Full=L-sorbose 1-dehydrogenase; Short=SDH && PF05199:GMC oxidoreductase|PF00732:GMC oxidoreductase
Lachesis_group0__13_contigs__l.g8679	15.26927	85.73351	-2.49353	0.000766	0.011436	1776	- && P81898.2 RecName: Full=Peptide-N4-(N-acetyl-beta-glucosaminyl)asparagine amidase A; Short=PNGase A; AltName: Full=Glycopeptide N-glycosidase; AltName: Full=N-glycanase; Contains: RecName: Full=Peptide-N4-(N-acetyl-beta-glucosaminyl)asparagine amidase A light chain; AltName: Full=PNGase A small chain; AltName: Full=PNGase A subunit B; Contains: RecName: Full=Peptide-N4-(N-acetyl-beta-glucosaminyl)asparagine amidase A heavy chain; AltName: Full=PNGase A large chain; Short=PNGase A subunit A [Prunus dulcis] && PF12222:Peptide N-acetyl-beta-D-glucosaminyl asparaginase amidase A
Lachesis_group0__13_contigs__l.g9432	2631.392	14868.48	-2.49835	0.000277	0.005516	1062	- && O06179.1 RecName: Full=Putative monooxygenase Rv1533 && PF03060:Nitronate monooxygenase

Lachesis_group0__13_contigs__l.g8991	69.69668	394.5386	-2.5009	9.22E-05	0.002453	1092	- && Q6GBM4.1 RecName: Full=Alcohol dehydrogenase; Short=ADH >Q6GJ63.1 RecName: Full=Alcohol dehydrogenase; Short=ADH >Q8NXU1.1 RecName: Full=Alcohol dehydrogenase; Short=ADH >Q5HI63.1 RecName: Full=Alcohol dehydrogenase; Short=ADH >Q2G0G1.1 RecName: Full=Alcohol dehydrogenase; Short=ADH >Q2FJ31.1 RecName: Full=Alcohol dehydrogenase; Short=ADH >Q2YSX0.1 RecName: Full=Alcohol dehydrogenase; Short=ADH && PF00107:Zinc-binding dehydrogenase
Lachesis_group0__13_contigs__l.g10011	58.26912	330.0055	-2.50289	0.00064	0.010056	2391	- && D4B0V1.1 RecName: Full=Probable glucan endo-1,3-beta-glucosidase ARB_02077; AltName: Full=(1->3)-beta-glucan endohydrolase ARB_02077; Short=(1->3)-beta-glucanase ARB_02077; Flags: Precursor && PF12708:Pectate lyase superfamily protein
Lachesis_group0__13_contigs__l.g3509	53.60493	309.6902	-2.5317	0.0012	0.015448	1569	- && Q01679.2 RecName: Full=Laccase; AltName: Full=Benzenediol:oxygen oxidoreductase; AltName: Full=Diphenol oxidase; AltName: Full=Ligninolytic phenoloxidase; AltName: Full=Urishiol oxidase; Flags: Precursor && PF00394:Multicopper oxidase|PF07732:Multicopper oxidase|PF07731:Multicopper oxidase

Lachesis_group0__13_contigs__l.g4886	358.301	2076.712	-2.53532	3.77E-07	3.20E-05	1851	- && O23461.1 RecName: Full=L-arabinokinase; Short=AtISA1 && PF13528:Glycosyl transferase family 1
Lachesis_group0__13_contigs__l.g9423	43.71851	253.5164	-2.53679	2.55E-06	0.000148	1491	- && - && PF07690:Major Facilitator Superfamily
Lachesis_group0__13_contigs__l.g5530	184.2234	1089.095	-2.56395	0.000235	0.004926	1578	- && P07921.1 RecName: Full=Lactose permease && PF00083:Sugar (and other) transporter
Lachesis_group0__13_contigs__l.g4273	9.874096	59.42771	-2.58895	0.000588	0.009562	1374	- && - && PF03595:Voltage-dependent anion channel
Lachesis_group0__13_contigs__l.g8558	449.2223	2705.973	-2.5907	1.06E-06	7.27E-05	1398	- && Q50I20.1 RecName: Full=2-methylcitrate synthase, mitochondrial; Short=Methylcitrate synthase; AltName: Full=(2S,3S)-2-methylcitrate synthase; AltName: Full=Citrate synthase 1; Flags: Precursor >B0YD89.1 RecName: Full=2-methylcitrate synthase, mitochondrial; Short=Methylcitrate synthase; AltName: Full=(2S,3S)-2-methylcitrate synthase; AltName: Full=Citrate synthase 2; Flags: Precursor && PF00285:Citrate synthase
Lachesis_group0__13_contigs__l.g8376	429.9717	2596.688	-2.59432	0.000223	0.0047	339	- && - && PF01185:Fungal hydrophobin
Lachesis_group0__13_contigs__l.g7748	1573.011	9706.311	-2.62537	2.07E-05	0.000788	849	- && - && PF08325:WLM domain
Lachesis_group0__13_contigs__l.g3661	7.279163	45.0613	-2.63628	0.001618	0.019161	792	- && - && PF11807:Domain of unknown function (DUF3328)
Lachesis_group0__13_contigs__l.g9472	34.79686	216.1817	-2.63661	0.000111	0.002768	1485	- && A1CFL6.2 RecName: Full=Cytochrome P450 monooxygenase patI; AltName: Full=Patulin synthesis protein I; AltName: Full=m-

							hydroxybenzyl alcohol hydroxylase; Flags: Precursor && PF00067:Cytochrome P450
Lachesis_group0__13_contigs__l.g6519	7466.775	46805.6	-2.64812	2.34E-08	3.40E-06	1803	- && Q09653.3 RecName: Full=Putative cytochrome P450 CYP13A10 && PF00067:Cytochrome P450
Lachesis_group0__13_contigs__l.g5160	803.6134	5072.574	-2.65811	7.49E-10	1.67E-07	990	- && O23300.1 RecName: Full=Enoyl-CoA delta isomerase 3; AltName: Full=3-hydroxyacyl-CoA dehydratase 1; AltName: Full=Delta(3),Delta(2)-enoyl CoA isomerase 3; Short=AtECI3 && PF00378:Enoyl-CoA hydratase/isomerase
Lachesis_group0__13_contigs__l.g2276	57.0547	359.9755	-2.65813	8.58E-08	9.86E-06	1551	- && Q25BW4.1 RecName: Full=Beta-glucosidase 1B; AltName: Full=Cellobiase 1B && PF00232:Glycosyl hydrolase family 1
Lachesis_group0__13_contigs__l.g7950	23.40773	147.645	-2.65961	0.000154	0.003493	1371	- && A9JPE2.1 RecName: Full=Cytochrome P450 monooxygenase atmQ; AltName: Full=Aflatrem synthesis protein Q && PF00067:Cytochrome P450
novel.536	9.966571	62.92743	-2.66494	0.000152	0.003464	1339	- && Q0CJ60.1 RecName: Full=Cyclase atC; AltName: Full=Terreic acid biosynthesis cluster protein C; Flags: Precursor && PF00732:GMC oxidoreductase|PF05199:GMC oxidoreductase
Lachesis_group0__13_contigs__l.g3973	22.32783	141.9066	-2.66863	5.19E-06	0.000263	723	- && O94255.2 RecName: Full=Carbonic anhydrase; AltName: Full=Carbonate dehydratase && PF00484:Carbonic anhydrase
Lachesis_group0__13_contigs__l.g8423	64.90055	412.8734	-2.67	4.41E-07	3.57E-05	900	- && Q9X248.1 RecName: Full=3-oxoacyl-[acyl-carrier-protein] reductase FabG; AltName: Full=3-ketoacyl-acyl carrier protein reductase; AltName: Full=Beta-Ketoacyl-acyl carrier protein reductase;

							AltName: Full=Beta-ketoacyl-ACP reductase && PF00106:short chain dehydrogenase
Lachesis_group0__13_contigs__l.g11055	37.51234	240.5971	-2.68237	7.23E-08	8.69E-06	1554	- && Q4WAZ6.1 RecName: Full=Multifunctional cytochrome P450 monooxygenase af510; AltName: Full=Fumagillin bioynthesis cluster P450 monooxygenase; Short=Fma-P450 && PF00067:Cytochrome P450
Lachesis_group0__13_contigs__l.g552	45.96372	295.1983	-2.68273	2.57E-09	5.06E-07	1968	- && Q5G234.1 RecName: Full=Pyranose 2-oxidase; Short=P2Ox; Short=POD; Short=POx; Short=PROD; Short=Pyranose oxidase; AltName: Full=FAD-oxidoreductase; AltName: Full=Glucose 2-oxidase; AltName: Full=Pyranose:oxygen 2-oxidoreductase; Flags: Precursor && PF05199:GMC oxidoreductase
Lachesis_group0__13_contigs__l.g5591	44.91338	288.3699	-2.68441	9.63E-07	6.75E-05	1479	- && Q0QWS4.1 RecName: Full=L-galactonate dehydratase && PF13378:Enolase C-terminal domain-like|PF02746:Mandelate racemase / muconate lactonizing enzyme, N-terminal domain
Lachesis_group0__13_contigs__l.g1789	1289.746	8310.735	-2.68799	2.50E-06	0.000147	396	- && - && PF03330:Rare lipoprotein A (RlpA)-like double-psi beta-barrel
Lachesis_group0__13_contigs__l.g8704	120.0496	776.7698	-2.69425	1.99E-07	1.97E-05	1488	- && - && PF04616:Glycosyl hydrolases family 43
Lachesis_group0__13_contigs__l.g5077	846.2914	5511.083	-2.70325	1.81E-07	1.86E-05	1395	- && Q7SH17.3 RecName: Full=Probable acetate kinase; AltName: Full=Acetokinase >Q5B3G6.2 RecName: Full=Probable acetate kinase; AltName: Full=Acetokinase && PF00871:Acetokinase family

Lachesis_group0__13_contigs__l.g10845	14.1631	92.16281	-2.70603	0.00483	0.043412	966	- && O74959.1 RecName: Full=Uncharacterized oxidoreductase C736.13 && PF00106:short chain dehydrogenase
Lachesis_group0__13_contigs__l.g4534	93.07207	612.753	-2.71895	2.63E-10	6.25E-08	1710	- && P45598.1 RecName: Full=Arabinose-proton symporter; AltName: Full=Arabinose transporter && PF00083:Sugar (and other) transporter
Lachesis_group0__13_contigs__l.g7335	6.222274	41.05297	-2.72564	0.0012	0.015448	1587	- && P49374.1 RecName: Full=High-affinity glucose transporter && PF00083:Sugar (and other) transporter
Lachesis_group0__13_contigs__l.g7749	42.32308	281.8881	-2.73425	2.70E-07	2.45E-05	1068	- && P0CU27.1 RecName: Full=Protein-lysine N-methyltransferase EFM3; AltName: Full=Elongation factor methyltransferase 3 && PF10294:Lysine methyltransferase
Lachesis_group0__13_contigs__l.g7311	6.783078	45.17547	-2.73452	0.000873	0.012745	4488	- && - && PF08719:Domain of unknown function (DUF1768)
Lachesis_group0__13_contigs__l.g2057	94.02483	629.7902	-2.74329	7.78E-06	0.000358	1098	- && - && PF05368:NmrA-like family
Lachesis_group0__13_contigs__l.g11360	7668.589	51685.91	-2.75276	4.38E-10	1.02E-07	1017	- && A1CFL1.1 RecName: Full=Alcohol dehydrogenase patD; AltName: Full=Patulin synthesis protein D && PF08240:Alcohol dehydrogenase GroES-like domain|PF00107:Zinc-binding dehydrogenase
Lachesis_group0__13_contigs__l.g5507	317.3286	2138.648	-2.75282	2.55E-06	0.000148	708	- && P37769.2 RecName: Full=2-dehydro-3-deoxy-D-gluconate 5-dehydrogenase; AltName: Full=2-deoxy-D-gluconate 3-dehydrogenase; AltName: Full=2-keto-3-deoxygluconate 5-dehydrogenase; AltName: Full=2-keto-3-deoxygluconate

							oxidoreductase; Short=KDG oxidoreductase; AltName: Full=20-ketosteroid reductase && PF00106:short chain dehydrogenase
Lachesis_group0__13_contigs__l.g9498	49.50716	336.031	-2.76308	2.05E-05	0.000785	2595	- && Q0CAF5.1 RecName: Full=Probable beta-glucosidase I; AltName: Full=Beta-D-glucoside glucohydrolase I; AltName: Full=Cellobiase I; AltName: Full=Gentiobiase I && PF01915:Glycosyl hydrolase family 3 C-terminal domain|PF14310:Fibronectin type III-like domain|PF07691:PA14 domain|PF00933:Glycosyl hydrolase family 3 N terminal domain
Lachesis_group0__13_contigs__l.g5464	1417.77	9700.339	-2.77445	1.82E-05	0.000711	873	- && - && PF00652:Ricin-type beta-trefoil lectin domain
Lachesis_group0__13_contigs__l.g7290	36.74538	252.7586	-2.78295	1.36E-06	8.86E-05	1614	- && P51687.2 RecName: Full=Sulfite oxidase, mitochondrial; Flags: Precursor && PF03404:Mo-co oxidoreductase dimerisation domain|PF00174:Oxidoreductase molybdopterin binding domain
Lachesis_group0__13_contigs__l.g6301	125.2177	866.453	-2.79126	1.18E-05	0.000499	831	- && O13871.1 RecName: Full=Uncharacterized methyltransferase C1B3.06c && PF13847:Methyltransferase domain
Lachesis_group0__13_contigs__l.g10522	257.1241	1781.351	-2.79274	4.33E-06	0.000229	1503	- && O74187.1 RecName: Full=Aldehyde dehydrogenase; Short=ALDDH; Short=ALDH && PF00171:Aldehyde dehydrogenase family
Lachesis_group0__13_contigs__l.g4409	17.9207	126.0201	-2.81875	0.00048	0.008295	4737	- && G3Y416.1 RecName: Full=Cytochrome P450 monooxygenase yanC; AltName: Full=Yanuthone D

							synthesis protein C; Flags: Precursor && PF00067:Cytochrome P450 PF07993:Male sterility protein PF00501:AMP-binding enzyme
Lachesis_group0__13_contigs__l.g11056	52.2422	372.1217	-2.83108	6.98E-10	1.59E-07	1233	- && Q4WAZ6.1 RecName: Full=Multifunctional cytochrome P450 monooxygenase af510; AltName: Full=Fumagillin bioynthesis cluster P450 monooxygenase; Short=Fma-P450 && PF00067:Cytochrome P450
Lachesis_group0__13_contigs__l.g5508	76.15057	543.9732	-2.8372	7.02E-06	0.000331	423	- && P33207.2 RecName: Full=3-oxoacyl-[acyl-carrier-protein] reductase, chloroplastic; AltName: Full=3-ketoacyl-acyl carrier protein reductase; Flags: Precursor && PF00106:short chain dehydrogenase
Lachesis_group0__13_contigs__l.g1462	168.321	1216.209	-2.85301	2.08E-06	0.000127	1110	- && O94564.1 RecName: Full=Zinc-type alcohol dehydrogenase-like protein C1773.06c && PF00107:Zinc-binding dehydrogenase
Lachesis_group0__13_contigs__l.g9559	1430.563	10648.38	-2.89601	3.50E-05	0.001153	8046	- && P04323.1 RecName: Full=Retrovirus-related Pol polyprotein from transposon 17.6; Includes: RecName: Full=Protease; Includes: RecName: Full=Reverse transcriptase; Includes: RecName: Full=Endonuclease && PF00665:Integrase core domain PF00078:Reverse transcriptase (RNA-dependent DNA polymerase)
Lachesis_group0__13_contigs__l.g5019	104.2864	787.9412	-2.91722	9.50E-07	6.71E-05	1689	- && Q47944.1 RecName: Full=L-sorbose 1-dehydrogenase; Short=SDH && PF00732:GMC oxidoreductase PF05199:GMC oxidoreductase

Lachesis_group0__13_contigs__l.g8159	104.6259	804.8152	-2.94403	8.18E-08	9.61E-06	1365	- && P23262.4 RecName: Full=Salicylate hydroxylase; AltName: Full=Salicylate 1-monooxygenase && PF01494:FAD binding domain
Lachesis_group0__13_contigs__l.g4685	789.3318	6127.511	-2.9566	1.88E-06	0.000116	549	- && P0AE54.1 RecName: Full=Putative peroxiredoxin bcp; AltName: Full=Bacterioferritin comigratory protein; AltName: Full=Thioredoxin reductase >P0AE53.1 RecName: Full=Putative peroxiredoxin bcp; AltName: Full=Bacterioferritin comigratory protein; AltName: Full=Thioredoxin reductase >P0AE52.1 RecName: Full=Putative peroxiredoxin bcp; AltName: Full=Bacterioferritin comigratory protein; AltName: Full=Thioredoxin reductase >P0AE55.1 RecName: Full=Putative peroxiredoxin bcp; AltName: Full=Bacterioferritin comigratory protein; AltName: Full=Thioredoxin reductase && PF00578:AhpC/TSA family
Lachesis_group0__13_contigs__l.g268	159.695	1249.725	-2.96826	2.95E-09	5.40E-07	945	- && O74959.1 RecName: Full=Uncharacterized oxidoreductase C736.13 && PF00106:short chain dehydrogenase
Lachesis_group0__13_contigs__l.g6318	79.24175	628.3281	-2.98681	3.73E-08	5.07E-06	645	- && B0DK57.1 RecName: Full=Solute carrier family 25 member 38 homolog && PF00153:Mitochondrial carrier protein
Lachesis_group0__13_contigs__l.g7178	116.3223	946.5209	-3.02413	1.64E-07	1.71E-05	2577	- && A1CA51.1 RecName: Full=Probable beta-glucosidase I; AltName: Full=Beta-D-glucoside glucohydrolase I; AltName: Full=Cellobiase I; AltName: Full=Gentiobiase I && PF00933:Glycosyl

							hydrolase family 3 N terminal domain|PF01915:Glycosyl hydrolase family 3 C-terminal domain|PF14310:Fibronectin type III-like domain
Lachesis_group0__13_contigs__l.g2228	149.4514	1221.837	-3.03146	2.48E-05	0.000912	1173	- && Q01752.1 RecName: Full=Aryl-alcohol dehydrogenase [NADP(+)]; Short=AAD && PF00248:Aldo/keto reductase family
Lachesis_group0__13_contigs__l.g5099	27.39714	226.5965	-3.0474	7.65E-08	9.09E-06	1377	- && P75791.1 RecName: Full=Uncharacterized protein YbiU && PF07350:Protein of unknown function (DUF1479)
Lachesis_group0__13_contigs__l.g10575	1073.487	9211.816	-3.1013	1.21E-10	3.32E-08	3297	- && Q9XF67.1 RecName: Full=3-phosphoinositide-dependent protein kinase 1; Short=AtPDK1 && PF00069:Protein kinase domain
Lachesis_group0__13_contigs__l.g1711	27.7631	240.3467	-3.11318	6.55E-05	0.001892	1452	- && B7ID58.1 RecName: Full=8-amino-7-oxononanoate synthase; Short=AONS; AltName: Full=7-keto-8-amino-pelargonic acid synthase; Short=7-KAP synthase; Short=KAPA synthase; AltName: Full=8-amino-7-ketopelargonate synthase; AltName: Full=Alpha-oxoamine synthase && PF00155:Aminotransferase class I and II
Lachesis_group0__13_contigs__l.g10566	14.57754	126.0447	-3.11362	0.000482	0.008303	798	- && - && PF13417:Glutathione S-transferase, N-terminal domain
Lachesis_group0__13_contigs__l.g5285	4.45657	39.334	-3.14169	0.002524	0.02668	432	- && Q1E8D2.1 RecName: Full=Heat-stable 19 kDa antigen; AltName: Full=CS-AG; Flags: Precursor && PF07249:Cerato-platanin

Lachesis_group0__13_contigs__l.g9199	1140.138	10414.39	-3.19135	6.13E-09	1.03E-06	435	- && P0CW95.1 RecName: Full=Heat-stable 19 kDa antigen; AltName: Full=Coccidioides-specific antigen; Short=CS antigen; Short=CS-AG; AltName: Full=Precipitin antigen; AltName: Full=TP-AG; Flags: Precursor >E9CX44.1 RecName: Full=Heat-stable 19 kDa antigen; AltName: Full=Coccidioides-specific antigen; Short=CS antigen; Short=CS-AG; AltName: Full=Precipitin antigen; AltName: Full=TP-AG; Flags: Precursor && PF07249:Cerato-platanin
Lachesis_group0__13_contigs__l.g3453	5.634859	52.23147	-3.21443	8.62E-05	0.002334	600	- && - && PF01185:Fungal hydrophobin
Lachesis_group0__13_contigs__l.g654	47.95509	445.2973	-3.21499	1.84E-06	0.000114	2025	- && Q54DY9.1 RecName: Full=Probable mitochondrial chaperone BCS1-B; AltName: Full=BCS1-like protein 2 && PF00004:ATPase family associated with various cellular activities (AAA)|PF08740:BCS1 N terminal
Lachesis_group0__13_contigs__l.g1814	16.44561	155.7323	-3.23995	6.87E-09	1.14E-06	2169	- && P94593.2 RecName: Full=Uncharacterized ATP-dependent helicase YwqA && PF00176:SNF2 family N-terminal domain
Lachesis_group0__13_contigs__l.g4968	8.10214	76.5187	-3.24427	2.47E-05	0.000912	597	- && - && PF05368:NmrA-like family
Lachesis_group0__13_contigs__l.g9674	26.18666	249.7617	-3.25343	1.59E-06	9.96E-05	3369	- && Q4WRH9.1 RecName: Full=Probable alpha/beta-glucosidase agdC; Flags: Precursor >B0XNL6.1 RecName: Full=Probable alpha/beta-glucosidase agdC; Flags: Precursor && PF01055:Glycosyl hydrolases family 31|PF13802:Galactose mutarotase-like|PF16863:N-

							terminal barrel of NtMGAM and CtMGAM, maltase-glucoamylase
Lachesis_group0__13_contigs__l.g60	46.85916	456.43	-3.28461	6.20E-07	4.60E-05	1077	- && Q4WBW4.1 RecName: Full=Probable acetylxylan esterase A; Flags: Precursor && PF10503:Esterase PHB depolymerase
Lachesis_group0__13_contigs__l.g5942	130.2114	1286.377	-3.30514	1.91E-10	4.64E-08	1047	- && P43549.1 RecName: Full=Uncharacterized membrane protein YFL054C && PF00230:Major intrinsic protein
Lachesis_group0__13_contigs__l.g451	138.7053	1411.333	-3.34694	6.95E-16	4.85E-13	1038	- && Q9UT59.1 RecName: Full=Putative uncharacterized oxidoreductase C513.07 && PF01370:NAD dependent epimerase/dehydratase family
Lachesis_group0__13_contigs__l.g8243	233.2982	2391.83	-3.35791	3.29E-06	0.000184	882	- && - && PF00652:Ricin-type beta-trefoil lectin domain
Lachesis_group0__13_contigs__l.g992	9.224412	94.8704	-3.36239	2.53E-07	2.39E-05	1593	- && B8NHY4.1 RecName: Full=O-methylsterigmatocystin oxidoreductase; Short=OMST oxidoreductase; AltName: Full=Aflatoxin B synthase; AltName: Full=Aflatoxin biosynthesis protein Q; AltName: Full=Cytochrome P450 64 >P0CT93.1 RecName: Full=O-methylsterigmatocystin oxidoreductase; Short=OMST oxidoreductase; AltName: Full=Aflatoxin B synthase; AltName: Full=Aflatoxin biosynthesis protein Q; AltName: Full=Cytochrome P450 64 && PF00067:Cytochrome P450

Lachesis_group0__13_contigs__l.g8595	1536.997	15911.45	-3.37187	2.59E-06	0.000149	882	- && A3PXS9.1 RecName: Full=Uncharacterized oxidoreductase Mjls_1918 && PF00248:Aldo/keto reductase family
Lachesis_group0__13_contigs__l.g5121	640.61	6672.492	-3.38075	1.04E-15	6.80E-13	813	- && Q9ZNN8.1 RecName: Full=L-2,3-butanediol dehydrogenase; Short=L-BDH; AltName: Full=(S,S)-butanediol dehydrogenase; AltName: Full=Diacetyl reductase [(S)-acetoin forming] && PF00106:short chain dehydrogenase
Lachesis_group0__13_contigs__l.g6565	13.94856	145.6365	-3.39004	5.64E-06	0.000279	2814	- && Q0CI48.2 RecName: Full=Beta-mannosidase A; AltName: Full=Mannanase A; Short=Mannase A; Flags: Precursor && -
Lachesis_group0__13_contigs__l.g1739	139.0493	1461.017	-3.39377	1.82E-09	3.80E-07	1803	- && Q00922.1 RecName: Full=Alcohol oxidase; Short=AO; Short=AOX; AltName: Full=Methanol oxidase; Short=MOX && PF05199:GMC oxidoreductase|PF00732:GMC oxidoreductase
Lachesis_group0__13_contigs__l.g4108	14.38833	152.9878	-3.40841	1.03E-06	7.15E-05	657	- && - && PF00583:Acetyltransferase (GNAT) family
Lachesis_group0__13_contigs__l.g6317	714.7119	7938.483	-3.47337	1.42E-06	9.11E-05	1269	- && Q9DBL1.1 RecName: Full=Short/branched chain specific acyl-CoA dehydrogenase, mitochondrial; Short=SBCAD; AltName: Full=2-methyl branched chain acyl-CoA dehydrogenase; Short=2-MEBCAD; AltName: Full=2-methylbutyryl-coenzyme A dehydrogenase; Short=2-methylbutyryl-CoA dehydrogenase; Flags: Precursor && PF00441:Acyl-CoA dehydrogenase, C-terminal domain|PF02770:Acyl-CoA

							dehydrogenase, middle domain|PF02771:Acyl-CoA dehydrogenase, N-terminal domain
Lachesis_group0__13_contigs__l.g5245	482.3715	5399.433	-3.48447	2.79E-14	1.39E-11	2412	- && G0SGU4.1 RecName: Full=Formate dehydrogenase; Short=FDH; AltName: Full=NAD-dependent formate dehydrogenase && PF00389:D-isomer specific 2-hydroxyacid dehydrogenase, catalytic domain|PF02826:D-isomer specific 2-hydroxyacid dehydrogenase, NAD binding domain
Lachesis_group0__13_contigs__l.g150	18.46067	208.4003	-3.49629	5.79E-08	7.04E-06	1164	- && Q04835.1 RecName: Full=Uncharacterized membrane protein YMR253C && PF00892:EamA-like transporter family
Lachesis_group0__13_contigs__l.g808	26.48723	313.3431	-3.56556	2.58E-08	3.69E-06	1584	- && - && PF12937:F-box-like
Lachesis_group0__13_contigs__l.g5817	1467.393	18381.77	-3.64697	3.06E-09	5.42E-07	5838	- && P41820.1 RecName: Full=Brefeldin A resistance protein && PF06422:CDR ABC transporter|PF00005:ABC transporter|PF01061:ABC-2 type transporter|PF00656:Caspase domain|PF14510:ABC-transporter extracellular N-terminal
Lachesis_group0__13_contigs__l.g8740	1584.595	20501.44	-3.6935	6.86E-17	5.12E-14	1641	- && - && PF01936:NYN domain
Lachesis_group0__13_contigs__l.g2012	11.64406	155.4319	-3.74127	1.65E-07	1.71E-05	2025	- && Q0CTV2.1 RecName: Full=Probable alpha-L-arabinofuranosidase A; Short=ABF A; Short=Arabinosidase A; Flags: Precursor && PF06964:Alpha-L-arabinofuranosidase C-terminus

Lachesis_group0__13_contigs__l.g10523	272.0105	3846.091	-3.82181	2.60E-11	9.05E-09	1503	- && O74187.1 RecName: Full=Aldehyde dehydrogenase; Short=ALDDH; Short=ALDH && PF00171:Aldehyde dehydrogenase family
Lachesis_group0__13_contigs__l.g4683	32.30376	466.6105	-3.85257	7.64E-11	2.22E-08	2316	- && Q9Y6Z9.1 RecName: Full=Sorbose reductase sou1; AltName: Full=Sorbitol utilization protein sou1 && PF13561:Enoyl-(Acyl carrier protein) reductase|PF00106:short chain dehydrogenase
Lachesis_group0__13_contigs__l.g359	14.57097	215.2181	-3.88971	5.81E-07	4.47E-05	960	- && O59952.1 RecName: Full=Lipase; AltName: Full=Triacylglycerol lipase; Flags: Precursor && PF01764:Lipase (class 3)
Lachesis_group0__13_contigs__l.g4765	130.7802	2013.551	-3.94534	1.42E-13	6.76E-11	1059	- && Q8K2T1.1 RecName: Full=NmrA-like family domain-containing protein 1 && PF05368:NmrA-like family
Lachesis_group0__13_contigs__l.g9938	1169.427	19460.47	-4.05661	1.68E-23	3.50E-20	3204	- && Q9Y8G7.1 RecName: Full=Bifunctional cytochrome P450/NADPH--P450 reductase; AltName: Full=Cytochrome P450foxy; AltName: Full=Fatty acid omega-hydroxylase; AltName: Full=P450foxy; Includes: RecName: Full=Cytochrome P450 505; Includes: RecName: Full=NADPH--cytochrome P450 reductase && PF00067:Cytochrome P450|PF00258:Flavodoxin|PF00667:FAD binding domain|PF00175:Oxidoreductase NAD-binding domain

Lachesis_group0__13_contigs__l.g6980	241.5895	4329.065	-4.16352	5.07E-30	2.65E-26	1572	- && Q0CJ61.1 RecName: Full=Efflux pump atB; AltName: Full=Terreic acid biosynthesis cluster protein B && PF07690:Major Facilitator Superfamily
Lachesis_group0__13_contigs__l.g6766	130.0752	2359.34	-4.18081	9.92E-10	2.16E-07	2499	- && I2C092.1 RecName: Full=Beta-mannosidase B; AltName: Full=Mannanase B; Short=Mannase B && -
Lachesis_group0__13_contigs__l.g1398	230.1016	4624.51	-4.3289	1.06E-12	4.62E-10	1689	- && S0EEY7.1 RecName: Full=Efflux pump FUS6; AltName: Full=Fusarin biosynthesis protein 6 && PF07690:Major Facilitator Superfamily
Lachesis_group0__13_contigs__l.g1951	13.02638	266.1563	-4.35292	2.66E-07	2.45E-05	459	- && - && PF16850:Peptidase inhibitor I66
Lachesis_group0__13_contigs__l.g9406	37.80871	814.4248	-4.42969	5.46E-15	3.36E-12	2289	- && Q4WFI6.1 RecName: Full=Probable exo-1,4-beta-xylosidase bxlB; AltName: Full=1,4-beta-D-xylan xylohydrolase bxlB; AltName: Full=Beta-xylosidase bxlB; AltName: Full=Xylobiase bxlB; Flags: Precursor >B0Y0I4.1 RecName: Full=Probable exo-1,4-beta-xylosidase bxlB; AltName: Full=1,4-beta-D-xylan xylohydrolase bxlB; AltName: Full=Beta-xylosidase bxlB; AltName: Full=Xylobiase bxlB; Flags: Precursor && PF01915:Glycosyl hydrolase family 3 C-terminal domain|PF14310:Fibronectin type III-like domain|PF00933:Glycosyl hydrolase family 3 N terminal domain
Lachesis_group0__13_contigs__l.g7910	123.7557	2918.18	-4.55949	3.05E-09	5.42E-07	1713	- && P49374.1 RecName: Full=High-affinity glucose transporter && PF00083:Sugar (and other) transporter

Lachesis_group0__13_contigs__l.g6349	75.40546	2729.088	-5.17758	4.51E-25	1.18E-21	1521	- && P0CP76.1 RecName: Full=Chitin deacetylase; Flags: Precursor && PF01522:Polysaccharide deacetylase
Lachesis_group0__13_contigs__l.g6193	4.983045	186.2932	-5.21478	5.13E-18	5.96E-15	1662	- && A2QEQ6.1 RecName: Full=Beta-glucuronidase; Short=GlcAase; AltName: Full=Beta-D-glucuronoside glucuronosohydrolase; Flags: Precursor && PF16862:Glycosyl hydrolase family 79 C-terminal beta domain
Lachesis_group0__13_contigs__l.g5319	75.62431	3002.786	-5.311	4.38E-19	6.54E-16	2592	- && Q00922.1 RecName: Full=Alcohol oxidase; Short=AO; Short=AOX; AltName: Full=Methanol oxidase; Short=MOX && PF05199:GMC oxidoreductase|PF00732:GMC oxidoreductase
Lachesis_group0__13_contigs__l.g1604	21.07942	1148.626	-5.76773	1.29E-38	1.35E-34	3090	- && - && PF15979:Glycosyl hydrolase family 115

Log_2Fold change: values of transcripts upregulated in Brij 30 addition is represented by positive numbers and downregulated is represented by negative numbers.

Padj: adjust *P*-value

Table S5 Primers used for qPCR

Primer	Sequence (5'-3')	Product (bp)	NR description
g2225-F1	CGCGACCAGATTGTCATTGC	187	Aryl-alcohol dehydrogenase [NADP(+)]
g2225-R1	TTTCGTAGTCCCACCAGTGC		
g7729-F1	CCAAGCAAAGGGGAGGTTCT	169	Dehydrogenase
g7729-R1	CTACAGCAAGCGAGGTGACA		
g9953-F1	GCCCAATGAGTTGCACTTCG	184	Cyrochrome P450 monooxygenase
g9953-R1	CGAGAAGAGCGGGATCATGG		
g8591-F1	CTGTCCAGGTTCCTTCGTCC	140	Ubiquitin carboxyl-terminal hydrolase
g8591-R1	CTCGTTGCTTGGTGATGCTG		
g10392-F2	GAGAAGAAGGGGACGAAGGC	197	Glycosyl hydrolase family
g10392-R2	GGGACAAGCTGTGCGTAGAA		
g8359-F1	ACCTCAAGGACACGCAGAAC	143	Dicarbonyl/L-xylulose reductase
g8359-R1	AACGTCGAAAGCGTCTCTGT		
g6290-F1	TTGAACCCCAAGGCTAACCG	152	Actin
g6290-R1	GAGACACCATCACCGGAGTC		

Sequence information of functional genes

>Lachesis_group0__13_contigs__l.g2225
ATGAGCCTCTTCGCAGCACCCCCTGAACCCAAGACCAAGCTCGGTATCCACCGTAAACTTGCTCCCACCGCTGGCGTCCATGTCTCGCCCATCCAGCTCGGTGCAATGA
GCATCGGCGACAAGTGGGCGCAGTACGGCATGGGTGCGATGGACAAGGAGAGTAGCTTCAAGCTGCTCGATGCCTTCTACGAGGCGGGTGGTAACTTCATCGATACCG
CGAATGGATACCAGGATGAGAGCTCTGAAGAGTTCCTCGGCGAGTGGATGGAGACACGCGGCATCCGCGACCAGATTGTCATTGCAACCAAGTATTCGAGCAACTTC
AAGCGCGGAGCAAAGGACATCTCACAGAAAGGCACCTACGTCGGCAATCACCTGAAATCGCTGCACCTCTCCGTGGAAGCTTCGCTCAAGAAGCTCCGCACAGACTA
CATCGACATTCTGTACGTGCACTGGTGGGACTACGAAACGAGCATCGAAGAGGTCATGAACGGCCTGCACCACCTTGTTGCCCAGGGCAAAGTGCTCTACTTGGGAAT
TTCTGACACGCCCGCATGGATTGTCGCCAAAGCGAACAGCTACGCGCGATACAACGGCAAGACGCCCTTCATCATCTACCAGGGCGCATGGAACGTCATGCAGCGCG
ACTTCGAGCGCGAGATCATTCCCCTTGCGCGCAGCGAAGGTATGGCACTCGCACCATGGAACGTCCTTGCAGGAGGCAAGATCCGCACGGATGCAGAGGAGCAGAGG
CGGCGCGAGACGGGGGAGCTCGGCCGTCGCACCTTCAACCCAAACTGGGAGCGCACCGAAAACGAGCGCAAGGTCTGTGCCGCACTCGAGAAGGTTGCAGCAGAG
GTTGGCACGAAGAGTATCACTGCCGTGGCAATCGCGTATGTGATGCAGAAGACGCCATACGTGTTCCCCATCGTCGGGGGGCGCAAGGTCGAGCACCTGAACGACAA
CATCGAGGCACTCAGCATCGCTCTCACGCCCGAGCAAATCGCGTTCATAGAGAGCATTCTACCCTTCGACTCTGGCTTCCCTTCGAACTTTGTTGTGAGTGCTCGGTTTT
CTGTCTTCACGGCTCTTCTGGTCCTGCGTCTTCAGTCGAGATGTATGCGCTCGGGAACAATGATGCTGATGATGATGTTTTTTCCACAGGGTGACGGGTCGGACTATATCC
ATATCTACAAGGCGTCCGGGCACTTTGTGAAGTGGCCCCAGACACAGGCCATTCGCCCGGCTGCGAATTAG

>Lachesis_group0__13_contigs__l.g7729
ATGTCCATTACCGTCCCGACAAAACATAAGGTCCTTGTCATACCTGTCGAACACGCCCCGTTCGAGGTCCAGGAAATCGAAGTAACCAAGCCAAGCAAAGGGGAGGT
TCTTGTTCGTGCAGAGAGCGTCGGCCTCAACCCCGCCGACTGGCTGGTCCGCGAGTTCAACCCGTTCAAATGGGCTCATCCCTGTATTCTTGGCTGTGAAGCCGCCGGA
ACCGTCGTGGAGCTTGGCGAAGGTGTCACCTCGCTTGCTGTAGGCGACAAAGTGATGCACCCTGCGTGGAACGGCGATGGGTACACCGCGTTCAAGCAGTACAACAC
GACTCTCGCAGATGTCTGCGTGAAGGTACCTTCCAACCTTACTATTGACCAAGCGGCGTCCATCCCGTTAGCGTTTAACACGGCCCTCGTCGGATTATACTGCCCGCCCG
AAAACCGTGGCGGCGCGGGCCTCGCGCCCTTCTGGAAGGACGGCGGACGCGGTAAATACGCCGGACAGCCCATCCTCATCCTCGGCGGCTCCTCAGGTGTTGCACAA
GCTGCGATCCAATTCGCGCGCATCTCCGGCTTCTCGCCTATCATCACGACTGCTTCACTCAAGAACACCGACCTACTCACGTCGCTCGGTGCGACGCACGTCCTTGACC

GCAACCTCTCAGACGAGGCGTTTGCCGCCGCCGTGCGCGGTATCATAGACCCAGCATCACTGCGCGTGGTCTTCGACAGCATGTCCGACCCAGCGATCCAACGCGCGG
CGTTCGAGCTGCTCGCGCCCGGGGGGTCGTTCATCGGCTGCTCGGGCGGCCGCAACCTGACCCCGGAGCAGCACGCGAGCAAGGAGAAGCAGTTCGTCTTTCCGTAT
GGGAGCCCGTACTGGGAGGATCGCCGGGAGCTGTGCGCCTCGTTCTTCAAGCAGATGCCTGCGTTCTGTGAGAGCGGGGAGCTCAAGCCGAACAAGGTGGAAGTCCT
TCCTGGTGGATGGGATGGCGCTATCGCTGGGTTGAAGAGGATGAAGGAGACTGGCGTGAGCGCCGTGAAACTAGTCGTCCATCCTCAAGAGACGGCCTAG

>Lachesis_group0__13_contigs__l.g9953
ATGCCCTGGACACAGCTTGGGATCGTGCAGACCCTGTTCGTCGCGCTCGCCGTGCATCGTCTCTGGATCATTATCTACAATGCATTCTTCCACCCGCTCGCGGCGTTCCC
CGGGCCAGTGCTGGCGAAGGTGACTACCTTGTGGCGGGCGTACGTCGAGCTGTACGGTACCGAGAACTTTACGACCGACACGCTCGTGGCGTTGCACGCCCGATATGG
GGACGTGGTGCGGGTCGCGCCCAATGAGTTGCACTTCGCAACCCCGCAGGCGTACCACGACATATACAACGCGAAGAACCGGTGGGACAAGGACGCGAAGCTGTAC
AACGCCCTGCGCGGCTCCGACGGGCATGCCTCCTTCCACCTCGCGCGCTTCCGTGACGCGAAGCAGCGCCGCGAGCCCATGATCCCGCTCTTCTCGCGCTCGTCGATC
ATGAGCATGCAGCACCTGCTCAACGAGAACCTCGACACACTCTGCGCGCACCTCGCGCGGCGCGACGCGGCAGGCGAGACGACGGACCTCCTCCTCGCGATGCGCG
CCTTCGCGCTCGACACGGTGACCGCGTTCTGCTTCGGCACGCCCTCCGTGCACGCGCTCTCCGCGCCGGGCTTCGACGCGCCCCTCGGGCGCGTCATGACGCTCATCAT
GACGTTCGTGCCCTCGCTCAAGCACTTCCCCGTCCTCCACTGGATGCTCAATCACATGCCGCCCGCGCTCGTGCGCGCCGCGCGCCCCGAGCTGACGGTGTTCTACGAG
TTCCACGACATAATCGCGAAGCAGGTCGACCACGCGCTGAACGACCCCGACGCGCTCGCCGCCGCGCCGCACCCGACGGTCTACCACGCGCTTTTGGACCAGCCGCA
CGCGTCCGCCGCACCAGCAGCGGCACGCGCGGCCCCTGCGCTGAGCTTCCGCGAGCTGTGCGCCGAGGCGCACCTGCTCGCGTTCGCGGGCACGGACACGGCGAGC
AACACGCTCACGTACGGCGTGACGAGCGTGCTCAGCGACGCGCGCGTGCACCGGACGCTCGTCGCGGCCCTGCGCGACGCGTGGCCTGATCTCGCGAGCCCGCCGCG
GTACGAGGAGCTCGAGAAGGTGCCGTACTTGAAAGCGGTCGTGCGGGAGTCTCTCCGCTGCCTGCACGGCGTGCTCTCGCCCATGGACCGCGTCGTCCCGCCCGAGG
GCGCGGTCGTCGCGGGGTCGTTCATCCCCGGCGGCACCGTCGTGGGCATCAGCAACATCTTCGTGCACCTCTCGCCCGAAATCTTCCACGACCCGCTCGTGTTCCTCCC
TGAGCGCTGGCTCGCAGACGACGCCGACACGCTCGAGCCGTGGCTCGTCGCGTTCTCCAAGGGCCCGCGGAGCTGTCTCGGGATCAACTTGGGCTACTGTGAGCTCTT
CCTGCTCTTCGCACACCTGTTCAGGCGATTCGAGCTGTCTCTCGTCGACGTCAGCGCGAAGGACATGGTCTGGGACGAGTGCTACCTGCCGCACTACGTCGGCCCGTCG
CTGCAGATCCGCACGAAGCCCGTCGACTCGTGA

>Lachesis_group0__13_contigs__l.g8591
ATGGCGAACCCAAATCAACAGTACTCGCATCCCGGTCCATCCTACTATCATACACCACCCCCGCCGCCTCAGTTTACCTACGCTCCTGGCCCAGGTGGTATGGCTTACTA
TCCATATCCCATGAACCCTTTGAACGGACATAGTCCGCCTCACCAGCCCGCCTCGCCAAGAATGAACGGAGGTGGTCGCGGGGGGTATCATCCACACCGAGCAGGGG

GGCCCAGCTACCATCATTTCCACCATGTCCCACCACCGCACTACCTGCCGCCGCAACCTATGCCAGCTTCTCCAGTCCCTCCAGGGAGTCCTTACGCGCACCCACAAAA
ATACCCCCCACATCCACAGCAAATGCCATATCCGCATGCATATCATCCTCCGCCTAATGGGCCGTACAATAATTCATGGCCTGCTCAGACGTTGTCCCCCTTGCCAAAAC
AGCTTTCCATGCCTGTACAATTGCCAACTCCTACCTCAATATCGGGCTCTCCGTTGCCTCCACCTTCTGTGGTTATATCCTCAGAGTACGTTGAGCCGATTCTTGATACACC
GCTGGTCAAGGACGAATCGACTTTCTCTCCCCAACCCCAAGAACCTACTGAGCCTCCACCGCCCTCGCCGCCACCGCCTTCCTCGCCGCCGAAAGAGGATGCTATGTT
TATGCCCTCAGTTGAGACTACGCTGGAAGCTATGGACAGATCGCCGACACCCAAAAGCCCTGTTTCTACGACATCCCGGATTTCTCGGAGCTCTCCTAGCTCCTTCGTA
GTATGGTCTCGCAAGCCCCGAGATCCTTCAAAATCGGTGGGAGTCGCTATCTCGTCCAGAGCGCATCCACCTGCGAATATCATTGACGACGCTATGGACTTACCCACAC
CGCCTTCATCTCCAAAACAACGGCCAAAAGATATCACTATTTCCTCGTCCGCTCCCGTCAGTTCCACAGTCGAAATTTCTCCGGAAGCTGTCCAGGTTCCTTCGTCCTCT
GCTACAGAGACGACACCTCCGAGCTCTATGGCGCCAGATACCCCAGTCCCTAGTTCTCCGCTGTCCAGTCATACATCGGTCTCAGTTGCTGCTACGCCAGCATCACCAA
GCAACGAGACTAAACAGGGCCTATCTGCGCCAGCGCACGTTGCGCAAGAGCAGCCCAAGTCGAACGAGTCAGTTTCTGTCGTTGTAGAAGGTTCGAAAGATGCCGCG
ATTCCCCCCCTGCAGCCTGCCATGGAGGCAGCTGCAACTGCTTCAGTCGCCCCGGCGGCGGAAGTCAAGCCTGCGCCGCCAGTCAAGAAGTCGTGGGCTTCCCTCTTG
CAGCCATCTGGTGCTGCCTCCTCATCCAACTCGCGTTTGCCGACTTCATCAGTCGTCGGGTTCTCCATTCCAGCTGGCCTGCAGAACGGCACCTCCAAACAGTCTACTTC
GTCTCCAGCAGGCACCGTGCGCCCCGAGCTGCTCAACCTGATCAATGTCGGTCCCACGGGATACTTTGCGCCACCAAAGATTCGTCCCCGCGGACTTGTCAATACCGG
AAACATGTGCTTTGCAAATGCCGTGTTGCAGATCCTGGTCTACTGCCAGCCGTTCTACCGTCTGTTCACTGAGGTGGGGAAGTTTATCGGCGAACAAGGTTCCTCGAAA
GACGAACCCAATGCGACGCCGCTCGTGGACGCGATCATTCAGTTCCTGAAGGAGTTTGCGCCCAAAGAGCCGGAGTTTGACCCTAGATCGAAAGGCAAGGAGAGGC
AGGAAGATTTCGACGATCTCGACTCTTTCATTCCAACGTATGTATACGACGTGTTGAAAGAAAAGAAGCGTTTCGCCAGTATGGTGGGCGGACATCAAGAAGATGCTG
AAGAATTCTTGGGCTTTTTTCTTGACACCCTGGAGGACGAGCTACTTTCCCTCTCTCAGGCGCCGTCTTCTGTAAAGGAGAAGGAGTCACAAGAAAACGTACCGGACG
ATGAATGGATGGAAGTCGGAAAACGCAATCGTTTGGTCTCGACGCGTACCGCCAAAAGCACTGAGTCTCCTATTACACGGATCTTTGGTGGCAAGTTCCGCTCGACAC
TGAAGGCTCCACATCAAAGAGACTCCGTCACTGTAGAAGACTGGCGTTCGCTGCGACTGGATATCCAGCGCGAGCAAGTGCACACCATTCAGGACGCATTGCAGTTC
ATCTCCGCGCCGCATTCGGTAGAGATCACCTCGCCGACACGTCCTGGTGCCATCATCGAAGCCAGCCGACAAGAGCTCATTGAGAACTTACCCCCGGTCCTCGTTCTCC
ACCTAAAACGCTTCTTATACGATACGAACGTCGGCGACGTCGTGAAACTCGGGAAGCACGTTACCTTTGGACCTGACCTTGAAATCGGCTCTGACTTATTGACCCCGAC
GCGTCGTACATTACATCCCGTGAAGTATCAGCTCTTTGGAGCATTGTACCACCACGGACAATCTGCATCCGGTGGTCATTACACGCTGGATGTTCTGCACCCTAATCGCG
AAATGAACGACCGGCCACGGCAAGCTTGGATACGCATTGACGACGAGCTGGTATCCGACGTACGTCCCGAGGACGTCTTCGGCGACCAGGAACGGGACGACCGCTGT
GCATACCTGCTCTTCTACCGACGACTAGGTCCCGCGAAGACGTGGTCATCGGCCGCGTCGTAG

>Lachesis_group0__13_contigs__1.g10392

ATGTCGCGCGCCGAAAAGATTCGCGGAAAGGTCTTTGGCGGTGGTGACAAGGCCGCCGCCCCAGACGACGACGCCTCCGTTTCTCGTTCTCCAGATGCTCCCCTCACC
ATCAGCATCCCTAAGCCACCTCCAACCCAGCCCGCTCCTCCCCAAAAGTCTAAGCAATCCGACTCGTCGCCCGGACCTGCTGCCGACGCTCAACAGAGGGACGACAA
GTGCTTCCGCGAGCGACTTATCCACCGCCTCGGTGAAGACTACAACGGTGCTGAGCGTTACAGGCTGTCTCAGGATGCCCGCAAAGACTTGCATTGGAAGCGTTGGGG
TCCCTATTTGAGCGACCGTCAATGGGCAACTGTCCGGGAAGATTATTCTGCTGACGGGGACGCCTGGTCCCATTTCCCTCACGATCACGCCCGTTCTCGCGCGTACCGTT
GGGGTGAAGACGGTATCGCAGGCATCTCCGACAACCATCAACGCTTATGTCTTGCCCTTTCGCTTTGGAACGGCGAAGACCCTATCCTTAAGGAGCGCTTCTTCGGCGT
GACTGGCCACCAAGGCAATCACGGCGAGGACGTGAAGGAGCTCTATTATTACCTAGACTCAACGCCGACCCACTCGTACATGAAATTCTTGTACAAATACCCTCAGAG
GCGTTATCCTTACGAACAGCTCGTAAGGGAGAACGCGAACCGCGGGCGCGATGTTCCCGAATTCGAGATCTTAGACACCGACGCCTTCGATGACGATCGTTATTGGGAT
ATCTTTGTCGAGTATGCGAAGGATGAACAGAATCCCGAGAACATACACATGCGTATCACTACCTACAACCGCGGGCCGGAACCCGCCACGCTGCACGTCATCCCACAA
TTGTGGTTCCCTAACTCCTGGTCATGGGACCAGGACCACCCTCCAAAACCATCCATGTATGCGTCTGGCGACGGGGTGATTACTGCGAAACACGAGACGCTAGGACAA
TACCATCTATACTGCATACCATCCCCGCCGCCAGTTGATTCTCAGGGCGAGGAAGTCACCTCAGAGACGGATATGATCCAGCCGGAATTGCTCTTCACGGAGAACAACA
CAAACTACCACCGATTGTATGGAGGTCAGAACGATACACCATACGTGAAGGATGGCTTCCATGACCACATTGTTCCGTCTCATCGGCCCCCTGCCCCGGAAGAGGAGG
ATGAAAAGGGGTACTTTATGTCCAGAATACATTCTCAGACGAGTCCTCATCGCACAGTTCCCTCTGGCGTAGACGAATCGGAGACACCGCCTAACGAGCGTGAACAAG
GTCCTCGCACTCCATTCCCGGGCCCTACCTTCGTGAACCCAGAGAAGAAGGGGACGAAGGCGGCTGCACATTACACCTTCCGCGATGTGCCCCCGCGCGGTGGATGTG
CAGTTGCACGCTTGACGCTGACACCCGTGGGCCCAGAGGAGAACACGACCGTCGAGGATGAGGGCATCTTCGATGATACGGTCGAGGAAAGACGGGAGGAGGCGGA
TGAATTCTACGCACAGCTTGTCCCCGGACCCATCAGTGACGACTTCAGGCAGATTATGCGACAGGCGTTCGGTGGAATGTTGTGGACGAAGCAGTACTACCAATTCGTC
CAGAAGACCTGGATCGAGGGTGACCCGGCGCAGCCGCCCCCGCCACCTGAGAGGAAGTGGATTCGTAACAAGGATTGGAAGCATCTTTACATTGCGGACATTCTGTC
CATGCCGGATAAATGGGAATACCCGTTCTTCGCTGCTTGGGATACCGCATTCCACTGCATTCCCCTTGCCGTCGTCGACCCGGCATTTGCAAAGAGGCAGCTCGATCTAC
TCACTCGCGAGTGGTACATGAAGCCCGACGGACAGATTCCCGCATACGAATGGAACTTCGGAGATGTTAACCCTCCGGTCCACGCCTGGGCGACTTTCCGTGTGTTCA
AGATCGAGCGCAAGGTGCACGGTGTAGAGGATTTGCCGTTCCTTGAGCGTGTATTCCAGAAGCTCCTGTTGAACTTTACGTGGTGGGTCAATCGTAAGGATGCAGGCG
GTAACAATGTCTTTGAGGGTGGATTCCTTGGGTTGGATAACATCGGCGTGTTCAACCGCTCGGAGCCCCTTCCTACCGGCGGTCGTTTACGTCAAGCTGACGGTACGGC
CTGGATGGCATTCTACTGCCTGAACATGCTGAATATCGCCCTTGAACTCGCCAAACACAACCCTGTGTACGAGGACATTGCGTCCAAGTTCTTCGAGCACTTCATCTTCA

TCTCTGATGCAATGACCTACCGCTCTGGCGAGGATGAAGTCAGCCTTTGGAACGAGCAGGACGGCATGTACTACGACGCCATTGAATTTGGACCCGGCAACACAATGC
AGCTTCCTGTACGCAGCTTAGTTGGTCTCATCCCTCTCTATGCCACTCTCGTCCTCGAGCCCTCGGTCATCAACAAGCTACCGGGCTTCAAGAAACGTATGGAGTGGTTC
ATCGAGAACCGGCCTGAGATTTCGGAGCGCACAATGGCCAACATGAAAGTGAAGGGCCGTGGCGAGCGGCGGCTGCTGGCGTTGGCGAGTAAAGAACGGTTGATCA
GCATCTTGGAGAAAATGCTTGACGAAGACGAGTTCTTCAGCGAACACGGCATTCGCTCGCTGTCGAAGAAGCACAAAGATCAGCCGTGGGGTATCGATGTTCACGGG
GAGCACTACGAGGTCAACTACTGGCCCGGCGACTCGATGTCCGGCATGTTCGGTGGCAACTCCAACTGGCGTGGTCCTATCTGGCTGGCCGTGAACTTCCTGTTGATAG
AGTCTCTCCAAAGATTCTACCAGTACTACGGAGACGAGGTCCAGGTAGAATGCCCAACCGGCAGTGGGGATTACATGAACCTCGCAAACGTCGCTGAAGAGATCCAG
CACCGCATCATCCACATTTTCGGACGTGACCGTGAGGGTAAGCGGGCAACGAACGGTGGTAATGTCAAGCTGGACCGTGACCCGCACTTCCGTGACTACGTCTGGTTC
TATGAGTTCTTCCACGCTGACGATGGGCGCGGTCTTGGAGCATCGCACCAGACCGGCTGGTCTGGCCTCGTCGCGTACCATATCCTGCAGAGCGGGGCCAGCTGCCGTC
TTCCAAAGACACCAAAGACTCCTCGCAGCGTCGTCAACCACTACTTTGATGAAACGATGTCCGCATACGGAGATGGTGCAGAGTCTGCTTACAGCGCATATAGCGCAG
CAGACCACAACACCCTCGGAGACATTTCGCCGGATGCCTTGTAG

>Lachesis_group0__13_contigs__l.g8359
ATGGCCACACTCGAAGGCAAGAAGATCGTCATCATCGGCGGCTCGTCTGGCATAGGCTACTCCGTGGCCAAGGCCTCCCTGCTCAACCTCGCGGGACACGTCCTCGTC
GCGTCGTCCTCGAAAGCGAAGGTCGACGCAGCGATCACACGCCTTCTCGCGGAGCCCGCGTTGAAGGCACACTCACCCCAAGGACGCGTTAGCGGAGACGTCGTGG
ACCTCAAGGACACGCAGAACGTCGCCGCGTTCTTCGAGAAAATCGGCGAGATCGACCACCTCATCATCACCAGCGGCAGCATTGCGCGCACCATCGACTTCCGTACC
GAGGACCTGAGCAAGCACAGAGACGCTTTCGACGTTAGGTTCTGGGGCGCGGCCGTCGCTGCACAGAAGGCGAAAATCATGGAGGGAGGATCCATTACGTTCACCAT
CGGCGGCGCTTTGTTGACACCCGCACCCACCTGGTCGCTCATCGTTAGCGCTATCGGTGCCGTCGACGCCCTCACGCGTTCTCTCGCTATCGAACTCGCCCCGTTACGCG
TGAATGTTGTCAGCCCAGGTGCCGTCGATACGGAGCTCTGGGATGGTTTCTCGGAAGACGCGAAGGCCAAGACGCTTGCCGGATATGCTGCAAAGCTGCCCGTCAAGC
ACGTCGCTGGACCTGATGAAATTGCCGAGGCGTATCTCTTCCTCATGAAAAAAAGCGGCGGAAGCGAGGGCGATCTTTGGGAAGTGCCGGAGATGGCTATTGCCTTCG
TACGACTCGGTACTGCTTTGATTAACGAGGTATGA

>Lachesis_group0__13_contigs__l.g6290
ATGGAGGACGAGGTCGCTGCTCTCGTTATCGACAATGGCTCTGGAATGTGCAAGGCTGGATTTGCTGGTGACGATGCTCCTCGCGCAGTCTTCCCATCCATCGTCGGTC

GCCCTCGCCACCAGGGTGTAATGGTTGGAATGGGTCAGAAGGACTCATACGTTGGTGATGAGGCTCAGTCGAAGCGTGGTATCCTCACGCTCAAGTACCCCATCGAGC
ACGGTATCGTCACCAACTGGGACGACATGGAGAAGATCTGGCATCACACCTTCTACAACGAGCTCCGTGTCGCACCTGAGGAGCACCCAGTGCTGCTCACGGAGGCC
CCGTTGAACCCCAAGGCTAACCGTGAAAAGATGACACAGATCATGTTCGAGACGTTCAATGCGCCAGCGTTCTACGTCGCGATTCAGGCTGTGCTCTCGCTTTACGCGT
CCGGTCGTACGACTGGTATCGTTCTCGACTCCGGTGATGGTGTCTCTCACACTGTGCCCATCTACGAGGGTTTCGCACTGCCGCACGCCATCCTCCGTCTTGATCTCGCC
GGTCGTGACATCACAGACTTGCTCGTCAAGAACCTGATGGAGCGCGGTTACCCCTTCAACACTACGGCCGAGCGCGAAATTGTCCGTGACATCAAGGAGAAGCTTTGC
TATGTTGCTCTCGACTTCGAGCAGGAGCTCCAGACCGCCGCACACTCGTCTGCCATCGAGAAGTCCTACGAATTGCCCGACGGTCAGGTCATCACCATCGGCAACGAG
CGGTTCCGTGCCCCTGAGGCGCTCTTCCAGCCCGCCTTCCTCGGACTTGAGGCTGCCGGTGTCCATGAGACGACCTACAACTCCATCTACAAGTGTGATCTCGATATTCG
TCGTGATCTCTACGGCAACATCGTCCTTTCTGGCGGTACTACCATGTTCCCTGGTATTGCTGACCGTATGCAGAAGGAGCTCACCGCTCTTGCGCCCTCGAGCATGAAGG
TCAAGATCGTCGCTCCTCCCGAGAGGAAGTACTCCGTCTGGATTGGTGGTTCTATCCTCGCGTCGCTCTCCACGTTCCAGAACCTCTGGTGCTCCAAGCAGGAGTATGA
CGAGTCAGGCCCTGGTATCGTTCACCGCAAGTGCTTCTAA
